# DNA Binding of the Cell Cycle Transcriptional Regulator GcrA Depends on N6-Adenosine Methylation in *Caulobacter crescentus* and Other *Alphaproteobacteria*

**DOI:** 10.1371/journal.pgen.1003541

**Published:** 2013-05-30

**Authors:** Antonella Fioravanti, Coralie Fumeaux, Saswat S. Mohapatra, Coralie Bompard, Matteo Brilli, Antonio Frandi, Vincent Castric, Vincent Villeret, Patrick H. Viollier, Emanuele G. Biondi

**Affiliations:** 1 Interdisciplinary Research Institute USR3078, CNRS–Université Lille Nord de France, Villeneuve d'Ascq, France; 2 Department of Microbiology and Molecular Medicine, University of Geneva, Geneva, Switzerland; 3 Laboratoire de Biométrie et Biologie Evolutive UMR5558, CNRS–Université Lyon 1–INRIA, Villeurbanne, France; 4 Laboratoire GEPV UMR 8198, CNRS–Université Lille 1–Université Lille Nord de France, Villeneuve d'Ascq, France; Universidad de Sevilla, Spain

## Abstract

Several regulators are involved in the control of cell cycle progression in the bacterial model system *Caulobacter crescentus*, which divides asymmetrically into a vegetative G1-phase (swarmer) cell and a replicative S-phase (stalked) cell. Here we report a novel functional interaction between the enigmatic cell cycle regulator GcrA and the N6-adenosine methyltransferase CcrM, both highly conserved proteins among *Alphaproteobacteria*, that are activated early and at the end of S-phase, respectively. As no direct biochemical and regulatory relationship between GcrA and CcrM were known, we used a combination of ChIP (chromatin-immunoprecipitation), biochemical and biophysical experimentation, and genetics to show that GcrA is a dimeric DNA–binding protein that preferentially targets promoters harbouring CcrM methylation sites. After tracing CcrM-dependent N6-methyl-adenosine promoter marks at a genome-wide scale, we show that these marks recruit GcrA *in vitro* and *in vivo*. Moreover, we found that, in the presence of a methylated target, GcrA recruits the RNA polymerase to the promoter, consistent with its role in transcriptional activation. Since methylation-dependent DNA binding is also observed with GcrA orthologs from other *Alphaproteobacteria*, we conclude that GcrA is the founding member of a new and conserved class of transcriptional regulators that function as molecular effectors of a methylation-dependent (non-heritable) epigenetic switch that regulates gene expression during the cell cycle.

## Author Summary

Methylation of genomic DNA at a specific regulatory site can impact a myriad of processes in eukaryotic cells. In bacteria, methylation at the N6 position of adenosine (m6A) is known to mediate a non-adaptive immunity response to protect cells from foreign DNA. While m6A marks are not known to govern expression of cell cycle genes in *Gammaproteobacteria*, cell cycle transcription in the model alphaproteobacterium *Caulobacter crescentus* requires the m6A methyltransferase CcrM that introduces m6A marks at GAnTC sequences and the enigmatic factor GcrA. Investigating if a functional and biochemical relationship exists between CcrM and GcrA, we found that CcrM-dependent m6A marks recruit GcrA to the promoters of cell cycle genes *in vitro* and *in vivo* and is required for efficient transcription. GcrA interacts with RNA polymerase, explaining how cell cycle transcription is affected. Importantly, m6A-dependent binding is also seen in GcrA orthologs, indicating that this transcriptional regulatory mechanism by CcrM and GcrA is conserved in *Alphaproteobacteria.*

## Introduction

Epigenetic signals, such as methylation of DNA, play an important role in the regulation of gene expression in eukaryotes. Methylation of adenines in the N6 position (m6A) has been described in Bacteria, Archaea, Protists and Fungi. Though known for its protective role in bacterial restriction/modification systems [1], m6A also fulfills cellular functions in *Gammaproteobacteria*, including the initiation of DNA replication, transposition, mismatch repair, and virulence gene expression [2]–[4]. In the *Alphaproteobacteria* such as *Caulobacter crescentus*, *Sinorhizobium meliloti*, *Brucella abortus* and *Agrobacterium tumefaciens*, the solitary methyltranferase CcrM is required for efficient growth, presumably through gene expression control of critical cell cycle genes [5]–[8].

The cell cycle role of CcrM was originally described in *C. crescentus* [5], [7]. At each cell division, *C. crescentus* generates two different cells (stalked and swarmer) committed to specific stages of the cell cycle [9]. The stalked cell is able to replicate the DNA (S-phase) and it possesses an extension of the external envelope and membranes, called stalk. The swarmer cell is instead motile and non-replicative (G1-like) possessing a polar flagellum and several pili. Upon nutrient availability the swarmer cell differentiates in a stalked cell, resembling the eukaryotic G1→S transition. In this cyclical progression, a crucial role is played by CtrA, an essential transcriptional regulator that targets many cell cycle genes [10]. Its activity and abundance are precisely regulated in time and space through phosphorylation, proteolysis and transcription. In G1, CtrA∼P inhibits DNA replication by repression of the origin of replication [11] and only upon CtrA proteolysis or dephosphorylation, DnaA-mediated chromosome replication initiation occurs [12] committing cells to the S phase. The re-synthesis of CtrA requires transcription of *ctrA* that relies on the methylation-sensitive *ctrA*P1 promoter [13] whose activation depends on GcrA, an enigmatic factor that is encoded in the genomes of *Alphaproeobacteria* and several *Caulophages* [14], [15]. While in *Caulobacter* GcrA accumulates in early S phase and is confined to stalked cells [16] for the activation of *ctrA*P1, a second, auto-regulatory promoter, *ctrAP2*, reinforces *ctrA* transcription later in S-phase. Upon CtrA synthesis, an essential phosphorelay, composed of CckA and ChpT [17], phosphorylates CtrA that in turn activates transcription of the *ccrM* gene. After the introduction of m6A marks in the context of GAnTC sequences [5] CcrM is proteolyzed prior to cell division by the Lon protease [7]. How the m6A marks, introduced by CcrM, affect transcription is unclear, but the marks are transient as DNA replication converts the (full) methylation on both DNA strands to the hemi-methylated state, until strands are re-methylated in a distributive manner [18] once CcrM has accumulated at the end of S-phase. The time a given genomic locus spends in the hemi-methylated state is thus pre-determined by its physical proximity to the origin of replication [19], a feature that might be exploited to couple activation of certain promoters, such as *ctrA*P1, with replication progression [19]. GcrA and CcrM are implicated in the transcriptional regulation of *ctrA*P1, suggesting linked roles. While an underlying biochemical relationship is also hinted by the analysis of the gene occurrence pattern in the *Alphaproteobacterial* genomes [20], this link remains experimentally untested.

Here we use chromatin-immunoprecipitation, biochemical, genetic and biophysical methods to explore the basis of transcriptional activation by GcrA. We uncovered that GcrA binds preferentially m6A-marked DNA and that it associates with the RNA polymerase, presumably to facilitate transcription initiation at methylated promoters. To assess if this mechanism is specific for *Caulobacter* or instead evolutionarily conserved, we performed experiments with GcrA orthologs in other *Alphaproteobacteria*, observing essentially the same behaviour. We conclude that GcrA and CcrM define an important regulatory pair, which is evolutionarily conserved and has been appropriated for epigenetic control of cell cycle transcription in *Alphaproteobacteria*.

## Results/Discussion

### GcrA forms an elongated, partially unfolded dimer

Because GcrA is a conserved protein that lacks primary structural resemblances to known functional domains, we investigated the features of the primary structure of GcrA by bioinformatic prediction using SMART [21]; first, a non-significant homology (E-value equal to 734) to helix-turn-helix domains was detected (13–55 aa). Also GcrA has a high content of positively charged residues such as arginine and lysine mostly located in the central region (45–80 aa). Those features may support the ability of GcrA to bind DNA directly (see next sections) through the N-terminal part. Consistently, the N-terminal part is also the region of GcrA that is more conserved at the evolutionary level across orthologs of GcrA shown in the Figure S1. This conservation suggests an important functional role of the region, for example in the specific DNA binding and also putative interactions with other cellular factors.

With the goal of investigating the interactions of GcrA with DNA and its targets *in vivo* we purified an N-terminally hexa-histidine tagged variant of GcrA (His_6_-GcrA) from an *E. coli* overexpression strain by sequential affinity and gel filtration chromatography and characterized its biophysical properties (see Materials and Methods). SDS-PAGE (Figure S2) and dynamic light scattering (DLS) analysis (data not shown) indicated a highly pure (>95%) and monodisperse preparation of His_6_-GcrA. Prediction of unfolded regions using RONN suggest that regions 41–105 aa and 145–173 aa of GcrA are disordered, while the software SOPMA [22] suggested that GcrA is partially structured in the N-terminal region (Figure S3) with an organization in three predicted alpha helices suggesting a folded structure. To gain insight into the spatial organization, we conducted Small Angle X-ray Scattering (SAXS) analysis (Protocol S1 and Table S1) that allows the determination of shape, size and oligomerization status of macromolecules in solution (Figure S4A). SAXS estimates the molecular mass of His_6_-GcrA at 42 kDa, which corresponds to a dimeric organization. Also the dimensions of His_6_-GcrA, by using computed radius of gyration (Rg) and maximum dimension (Dmax) values, combined with the pair-distance distribution function, P(R), shape and the Kratky plot representation, are consistent with an elongated form and partially disordered conformation of His_6_-GcrA dimers in solution (See legend of Figure S4A for more technical details). Possibly the interaction of GcrA with other proteins and with DNA can induce a stabilization of the disordered regions.

Next, we performed limited proteolysis followed by MALDI-TOF mass spectrometry (MS) analysis in order to identify regions of His_6_-GcrA that were more resistant to proteolysis indicating its more compact (structured) nature. Two different proteases, Thermolysin and V8 (see Materials and Methods) were used and the most resistant fragments to proteolysis were analyzed by MS (Figure S4B). This analysis suggests that the N-terminal part of GcrA (from 1 to 115 ca.), although containing proteolytic sites for both proteases, was more stable than the C-terminal part, as also indicated by the prediction of alpha helical structures in the N-terminal portion of GcrA.

### Genome-wide occupancy of GcrA at promoters *in vivo*

In light of these structural features suggesting that the N-terminal domain of GcrA binds DNA, we sought specific *in vivo* targets of GcrA that could be used to probe DNA-binding of GcrA *in vitro*. Previous non-quantitative chromatin-immunoprecipitation (ChIP) experiments using polyclonal antibodies to GcrA, provided support for the notion that GcrA affects the transcription of cell cycle genes by, directly or indirectly, associating with specific chromosomal sites [16]. To illuminate the basis for this selectivity and the underlying mechanism of transcriptional regulation by GcrA in *Alphaproteobacteria*, we subjected ChIP samples from NA1000 wild-type cells to deep sequencing (ChIP-Seq) [23] (Protocol S2). By mapping the reads onto the genome, we determined the binding profile of GcrA on genomic regions.

**Figure 1:**
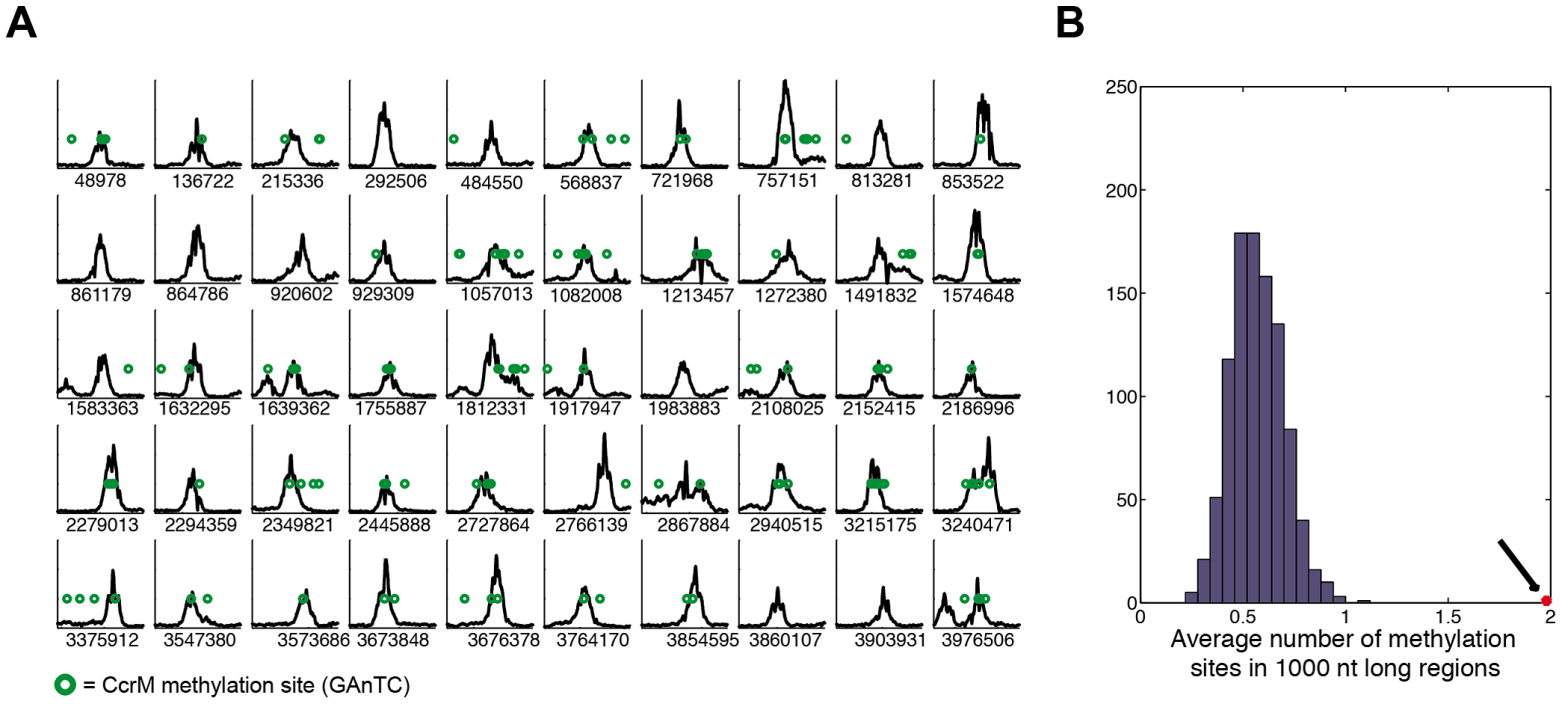
Chromatin Immunoprecipitation and deep sequencing (ChIP–Seq) of GcrA-bound genomic sequences are enriched in methylation sites. *(A)* Best 50 sequences 2 kbp long from Table S2 of the *C. crescentus* genome that are immunoprecipitated with GcrA antibodies. In each plot the number of reads per nucleotide in 2 kbp is shown. The number below the graphs corresponds to the position in the NA1000 genome of the middle of the 2 kbp region. Methylation sites of CcrM (GAnTC) are represented as green circles. *(B)* A random sampling of 1 kbp sequences reveals a distribution of methylation sites around 0.6, while the GcrA sequences have an average number of 2 (Black arrow).

First, we used a peak finding strategy to identify regions bound by GcrA with high affinity (Protocol S2); this analysis allowed to identify 218 peaks that were subsequently associated to the closest genes (Table S2). Inspection of the top 50 targets (Figure 1A) revealed wide peaks (*ca.* 1 kbp wide, data not shown). We found that half of these GcrA-bound sequences were significantly associated with CcrM methylation sites (GAnTC). To explore this association more in details, we calculated the average number of methylation sites in 1 kbp windows centered on the peaks and found it to be close to 2 (1.78), in comparison with 0.58 sites per random 1 kbp genomic regions (Figure 1B). These results clearly indicate a significant enrichment of methylation sites in genomic regions bound *in vivo* by GcrA, raising the possibility that methylation enhances GcrA binding to its targets.

Next, in all promoter regions, defined from 300 bp upstream to 100 bp downstream a gene's start codon, we calculated the number of ChIp-Seq reads (see Protocol S2) (Table S3). We obtained (Z-score ≥2) 161 GcrA-bound promoter regions, 89 of which also contained a GAnTC methylation site (data not shown). This list contained many known GcrA-controlled targets such as *mipZ*, encoding a division regulator [24], *podJ* encoding a polarity factor [25], [26] and *ctrA*. We observed a remarkably small overlap with the genes previously identified as GcrA-dependent by DNA microarrays [16]. Only 5 genes passed the threshold (Z score ≥2), including those encoding CCNA_01542 (ice nucleation protein), CCNA_01556 (LacI family transcriptional regulator), CCNA_01766 (hypothetical protein), CCNA_02005 (inosine-uridine preferring nucleoside hydrolase), CCNA_02086 (sporulation domain containing protein), CCNA_02246 (*mipZ*), CCNA_02401 (encoding a transcriptional regulator) and CCNA_03325 (encoding a hypothetical protein). Since microarrays detect both direct and indirect mRNA abundance changes, it is likely that many genes whose expression was affected by GcrA depletion were, in fact, indirect targets of GcrA presumably under the control of other transcription factors, such as CtrA.

### GcrA defines a new class of specific DNA–binding proteins

**Figure 2:**
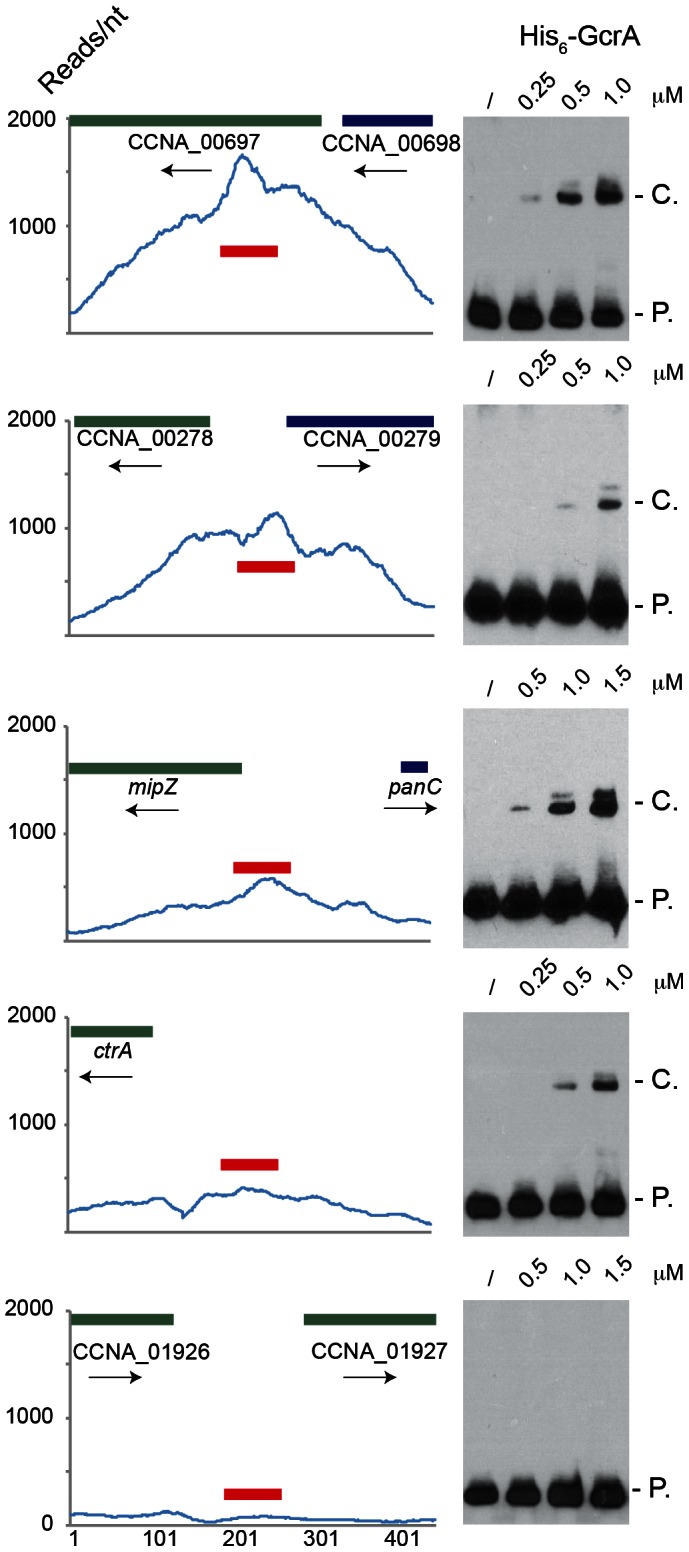
Electrophoresis mobility shift assay (EMSA) of 5 ChIP–Seq regions with His_6_-GcrA. EMSA results using increasing concentrations of purified GcrA using probes (red line) design in regions with high number of reads in ChIp-Seq results. From the top to the bottom: 1) the sequence with the maximum number of reads ChipSeq results, corresponding to the coding sequence of CCNA_00697; 2) the intergenic sequence between CCNA_00278 and CCNA_00279; 3) The promoter of *mipZ*; 4) The promoter *ctrA*P1 of *ctrA*; 5) A negative control, corresponding to the intergenic between CCNA_01926 and CCNA_01927. On the right, P. = Probe signal and C. = complex signal. Dissociation constants (Kd) of the positive probes are shown in Figure S9.

In order to test if GcrA binds DNA *in vitro*, we set up an electrophoretic mobility shift assay (EMSA) with His_6_-GcrA and regions identified as *in vivo* targets of GcrA by the ChIP-seq experiment described above. We selected 5 regions as EMSA probes, each with distinct features: 1) the preferred target (with the maximum number of reads in Table S2) corresponding to the N-terminal coding sequence of gene CCNA_00697 that has three GAnTC sites; 2) the intergenic sequence between CCNA_00278 and CCNA_00279 that is efficiently bound by GcrA *in vivo*, but has no predicted GAnTC methylation site; 3) the promoter of the gene *mipZ*, which was also discovered previously by microarray as a GcrA-dependent gene and has two juxtaposed GAnTC sites; 4), the P1 promoter of *ctrA* (*ctrAP1*) that has one GAnTC site (there is another GAnTC sequence after the transcription start site of the *ctrA*P2 promoter) and is thought to be activated by GcrA [16]; 5) a negative control, corresponding to the intergenic region between CCNA_01926 and CCNA_01927 which GcrA binds non-specifically *in vivo* based on ChIP-seq data. Probes were designed as 70-mer double stranded oligo-nucleotides, in which the central part corresponds to the genomic region with the highest number of ChIP-seq reads. The EMSA (Figure 2) showed that His_6_-GcrA shifted four out of five probes, indicating the formation of a His_6_-GcrA•DNA complex with sequences identified by ChIP-Seq, except for the intergenic sequence between CCNA_01926 and CCNA_01927 (i.e., the negative control). All positive probes gave rise to two distinct His_6_-GcrA•DNA complexes with similar migration properties, possibly reflecting different oligomeric states of His_6_-GcrA with different apparent affinities for the DNA (see below). In particular, probe CCNA_00697 was the most efficiently bound by His_6_-GcrA (Kd = 4±0.5 µM); probes *ctrA* (Kd = 6.5±0.5 µM) and *mipZ* (Kd = 8.5±0.5 µM) also showed DNA binding however the complex forms only at a higher concentration of His_6_-GcrA, mirroring, with the exception of the intergenic region between CCNA_00278 and CCNA_00279 (Kd>9 µM), the binding preference ChIP-seq *in vivo.* The EMSA results also demonstrate that His_6_-GcrA binds DNA in a specific fashion *in vitro*. Considering also that the conserved GcrA protein has no homology with known DNA binding proteins at the primary structure level, we conclude that GcrA defines a new class of alphaproteobacterial DNA binding proteins that directly interacts with target promoters to control transcription of many *Caulobacter* S-phase genes, including the gene encoding the master regulator CtrA. Although methylation sites are associated with GcrA-bound regions, GcrA apparently can also bind sequences that do not contain GAnTC methylation sites, based on the methylation-dependent binding experiments described below, we suggest that multiple DNA constrains exist in the GcrA specificity, perhaps involving m6A marks in different sequences contexts or a different type of methylation mark altogether. In this context, it is noteworthy that two putative cytosine methyltransferases are encoded in the *C. crescentus* genome [27].

### DNA binding of GcrA is enhanced by CcrM-dependent methylation

**Figure 3:**
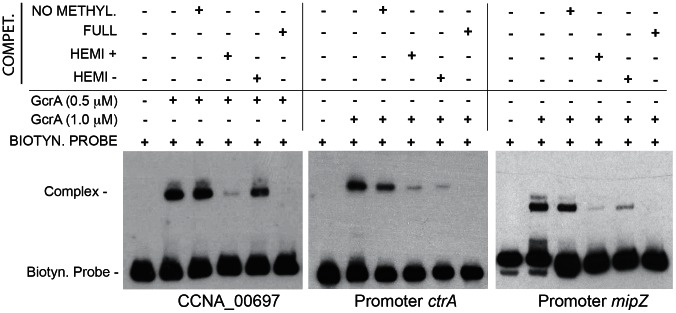
GcrA DNA binding depends on CcrM methylation state. Three regions (CCNA_0697, *ctrA*, *mipZ*) containing methylation sites of CcrM were tested in a competition experiment. Competitor DNA identical to the probe with various methylation states was mixed with biotinylated probes and GcrA in order to evaluate competition. For CCNA_00697 we used 0.375 µM of competitors, while for *ctrA* and *mipZ* promoters we used 1.25 µM. Dissociation constants (Kd) of the methylated probes of *ctrA* and *mipZ* probes are shown in Figure S9.

To test if GAnTC methylation modulates the binding of His_6_-GcrA to some of its targets *in vitro*, we conducted EMSA competition experiments with the un-methylated region of P*_mipZ_*, CCNA_00697 and *ctrA*P1 as biotinylated probes and double stranded synthetic oligos harboring N6-adenosine methylation at GAnTC on either one or both strands as competitors. In these experiments, His_6_-GcrA was pre-incubated with the unlabeled competitor DNA, followed by the addition of the biotinylated probe. The more the unlabeled competitor DNA reduces the abundance of the shifted His_6_-GcrA•DNA complex, the higher the affinity of His_6_-GcrA is for the unlabeled competitor. As shown in Figure 3, we observed a clear preference of GcrA for the methylated competitors over the un-methylated one, with those carrying methylation on both strands (“full”-methylation) competing better than either one harbouring the methylation on one of the two strands (“hemi”-methylation). Remarkably, in the case of the CCNA_00697 and *mipZ* competitors, hemi-methylation on the “sense” strand is a better competitor than the hemi-methylated competitor with the modification on the other strand. For promoters of *ctrA* and *mipZ*, the calculated Kds provided quantitative confirmation of the results shown in Figure 3 (Figure S9B and S9C).

**Figure 4:**
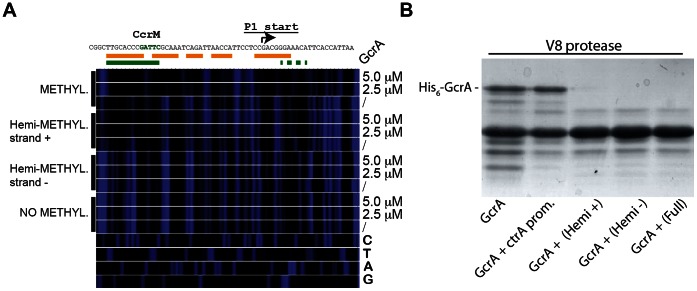
Binding of GcrA to DNA in different GAnTC methylation states and conformation al changes of GcrA induced by DNA. *(A)* DNase I footprinting of the *ctrA*P1 promoter by GcrA. The promoter region was tested in different methylation states with increasing concentrations of His_6_-GcrA. The nucleotide sequence of the *ctrA*P1 promoter with the start site and the CcrM methylation site (green)vis shown on top. In orange, the putative region, protected by GcrA in the fully methylated state. In green the putative region protected in the hemi-methylated strand plus. *(B)* Proteolytic digestion using V8 in presence of DNA. In particular 4 kinds of DNA corresponding to the *ctrA*P1 promoter were tested as reported in the bottom part.

In order to assess if methylation alters the disposition of GcrA on its target DNA, we conducted DNase I protection assay using fully and hemi-(GAnTC) methylated fluorescently-labeled *ctrAP1* promoter probes. As shown in Figure 4A, GcrA protects specific regions of the probe in a methylation-dependent manner, giving rise to a larger region of protection spanning the −35 to the −10 of the *ctrA*P1 promoter with the fully-methylated (*i.e.* on both strands) probe. By contrast, the protection of the hemi-methylated (on the plus strand) probe was confined to a region adjacent to the methylation site itself. Importantly, the un-methylated probe and the hemi-methylated probe carrying the modification on the minus strand did not show protection by His_6_-GcrA at any concentration. We conclude that methylation induces different oligomerization or conformational states in strand-specific manner.

Next, we complemented the DNase I protection experiments of the target, with protection experiments of His_6_-GcrA by limited proteolysis in the presence or absence of the various methylated *ctrA*P1 probes (Figure 4B). We found that efficiency of proteolysis is accelerated in the presence of methylated probes, suggesting that conformational rearrangements are induced by the methylated probe to expose protease hypersensitive sites, akin to the DNase I hypersensitive sites of the target DNA that become exposed only when GcrA associates with a methylated target, but not in the presence of the un-methylated site. We also ruled out the possibility that the oligos affected the DNase I activity by incubating another protein (His_6_-ChpT) [28], which was proteolyzed identically with or without the DNAs (data not shown).

CcrM-dependent methylation of *ctrA*P1 was previously proposed as an essential element of a transcriptional regulatory switch, culminating in the GcrA-dependent activation of *ctrA*P1 upon the conversion from full- to hemi-methylation [19]. Intriguingly, our results reveal that His_6_-GcrA binds hemi-methylated versus fully methylated *ctrA*P1 in strikingly different manner, with the latter covering a much larger area. This raises the possibility that cooperative interactions, induced by the transition from hemi- to full-methylation mediated by CcrM, can lead to a wider and stronger association of GcrA with the target DNA. As His_6_-GcrA wraps the entire region from −35 to +7 of fully methylated *ctrA*P1, it may interfere with RNA polymerase holo-enzyme (RNAP) at *ctrA*P1; a possibility that must be explored in future work. By contrast, on the hemi-methylated plus strand of *ctrA*P1, His_6_-GcrA occupies a 12 nt stretch overlapping the −35 region and adjacent GAnTC site and with lower affinity a 12 nt region from +5 onwards. GcrA could compete with RNAP for binding to *ctrA*P1 or alternatively tether it at the promoter, preventing promoter clearance, *i.e.* the switch from transcription initiation to the elongation phase. Furthermore the methylation strand-specificity opens the possibility of an “asymmetric” mechanism of gene regulation, in which only one of the two replicated loci is preferentially bound and transcriptionally regulated by GcrA before re-methylation by CcrM in the pre-divisional cell. Such, a regulatory bias could have far reaching consequences in all forms of spatiotemporal and/or of gene-dosage regulation for all living cells, as it has been suggested before for PapI-promoted Lrp binding to hemi-methylated sites in uropathogenic *E. coli* [29].

### GcrA–dependent interactions with RNAP

**Figure 5:**
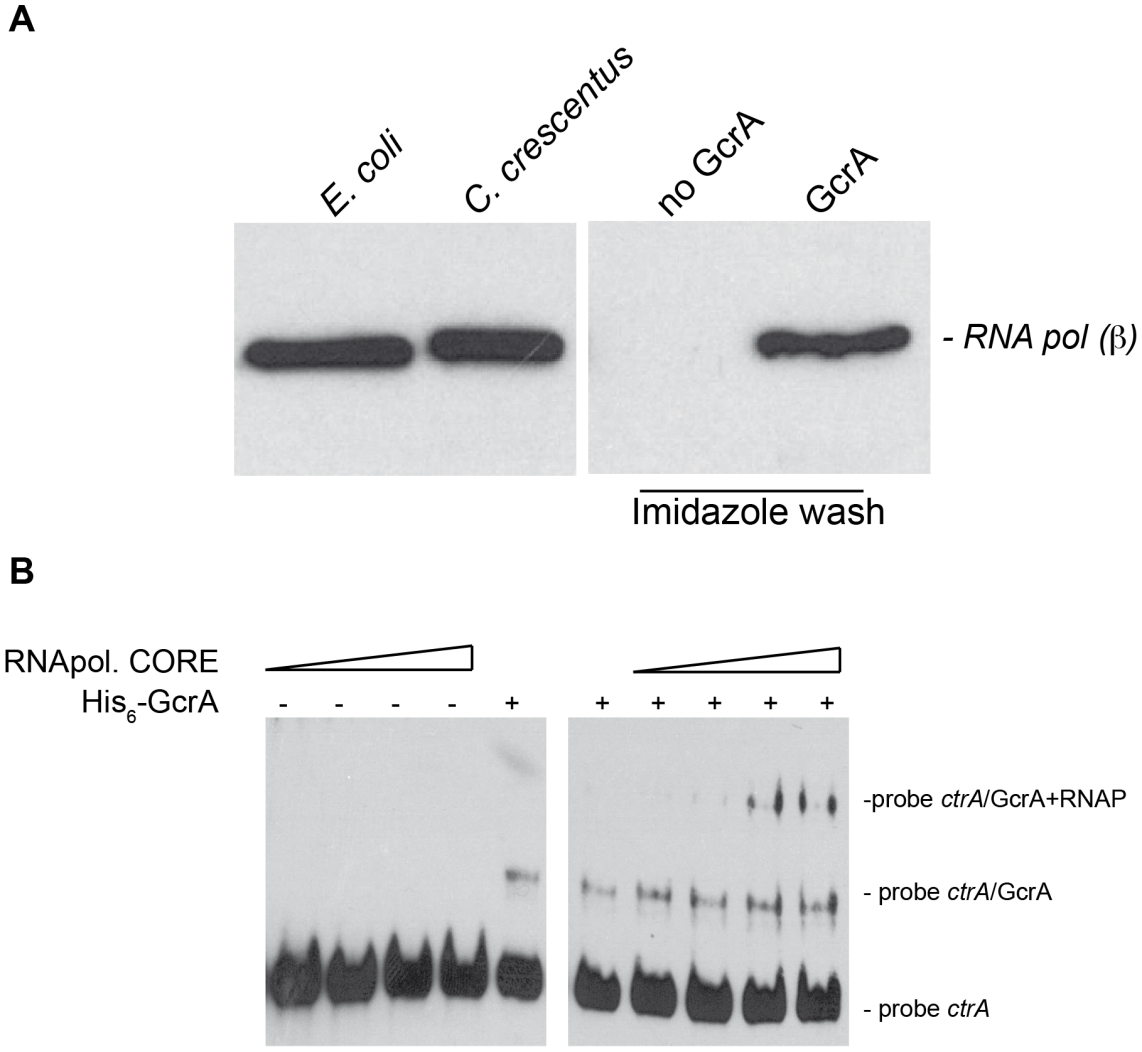
GcrA interacts with RNA polymerase. *(A)* Immunoblots using RNAP β subunit antibodies on several samples. On the left cellular lysates of *E. coli* cells and *C. crescentus* cells, both positives to the antibodies. On the right, samples of *C. crescentus* CB15N cell lysate applied to the column containing His_6_-GcrA and subsequently washed with salt (up to 1 M) and imidazole in order to remove His_6_-GcrA and putative interactors. This procedure was done also with an empty column (“No GcrA” lane). The nickel column loaded with His_6_-GcrA was used to detect the association of GcrA to RNA polymerase. *(B) E. coli* RNA polymerase core enzyme is able to bind the GcrA-DNA complex (promoter *ctrA*P1), as visualized by a slower migration rate as the amount of RNA polymerase increased. On the left the same RNA polymerase conditions were tested without GcrA.

To explore the models described above, we tested whether GcrA can directly or indirectly associate with RNAP. To this end, we passed a soluble C. *crescentus* cell lysate, in which DNA was fragmented by a mild DNase I treatment, over a nickel-NTA column that had been pre-loaded or not with His_6_-GcrA. Following extensive washes with buffer containing up to 1 M NaCl, we eluted His_6_-GcrA and associated proteins with buffer containing imidazole (see Materials and Methods). Blots harbouring these samples were then probed with antibodies to the β subunit of core RNAP, revealing that RNAP β in the eluate from the His_6_-GcrA pre-loaded column only (Figure 5A).

We extended these findings by showing that *E. coli* RNAP core enzyme can associate with the DNA•GcrA complex in an EMSA using *ctrA*P1 promoter. Increasing concentration of RNA polymerase clearly showed the formation of lower mobility complex whose formation was dependent on the presence of GcrA (Figure 5B). This interaction of RNAP with GcrA bound to its target was also observed with the *mipZ* promoter (Figure S5). Taken together, these results indicate that GcrA binds components of the RNA polymerase core complex and they provide a mechanistic explanation for how GcrA might affect gene transcription.

### Transcriptional activation and promoter binding by GcrA *in vivo* requires methylation

**Figure 6:**
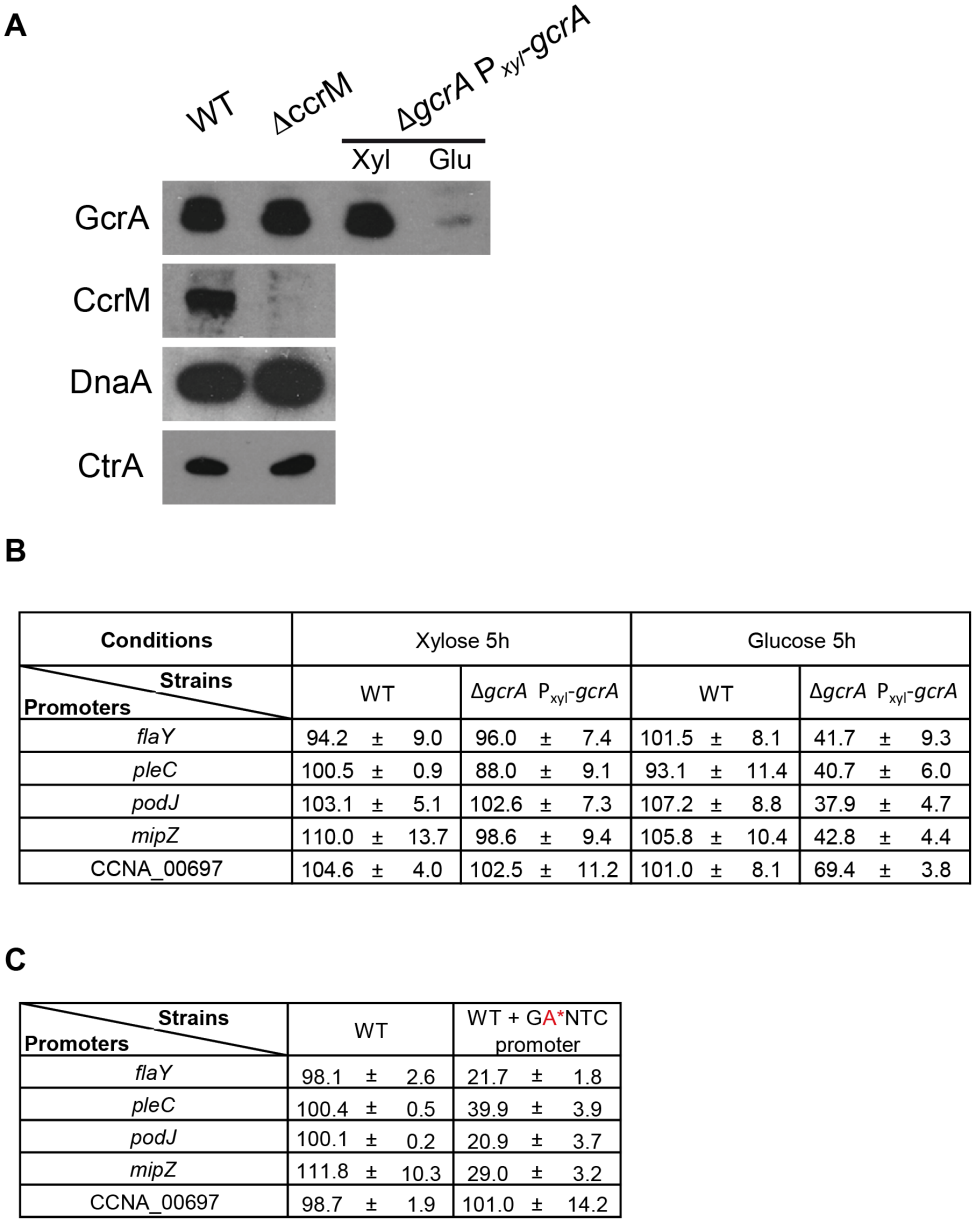
CcrM and GcrA dependence of selected promoters. *(A)* Immunoblots showing that the Δ*ccrM* mutation does not affect the steady-state levels of the master cell cycle regulators, GcrA, CtrA and DnaA using polyclonal antibodies to these proteins. *(B)* GcrA-depletion impairs P*_mipZ_*-, P*_podJ_*-_,_ P*_flaY_* - and P*_pleC_*-*lacZ* activity, while affecting P*_CCNA_00697_*-*lacZ* to a lesser extent. β-Galactosidase activities were measured in the WT or the GcrA depletion strain harbouring the transcriptional reporters after growth in PYE supplemented with xylose (0.3%) or glucose (0.2%) for 5 hours and tetracycline to select for the reporter plasmid. *(C)* Mutation of the CcrM recognition sites (GAnTC→GCnTC) cripples P*_mipZ_*-, P*_podJ_*-_,_ P*_flaY_* - and P*_pleC_*-*lacZ* activity, but not P*_CCNA_00697_*-*lacZ.* WT cells harbouring the reporter plasmids were grown PYE and tetracycline to select for the reporter plasmid.

The connection between methylation by CcrM and DNA-binding of GcrA, seen *in vitro*, together with the association of GcrA to RNAP, prompted us to explore if other GcrA target promoters are regulated in a methylation-dependent manner *in vivo*. To this end, we fused several promoters that have methylation sites and that emerged as *in vivo* targets of GcrA in the ChIP-seq experiments to the promoter-less *lacZ* reporter gene. We first confirmed the GcrA-dependence of these promoters by measuring *lacZ*-encoded β–galactosidase activities under GcrA-replete and deplete conditions using a Δ*gcrA*::Ω; *xylX*::P_xyl_-*gcrA* strain [16] in which GcrA expression is induced in the presence of xylose and repressed in the presence of glucose (Figure 6A). After 5 h of depletion of GcrA in glucose, the β–galactosidase activities of the P*_mipZ_*-, P*_podJ_*-_,_ P*_flaY_* - and P*_pleC_*-*lacZ* reporters dropped by *ca.* 60% compared to the WT grown in glucose or the P*_xyl_*-*gcrA* strain grown in xylose, and immunoblotting revealed a strong reduction in MipZ and PodJ abundance (Figure S6). It was previously also shown that activation of the *ctrA*P1 promoter requires GcrA [19]. By contrast, the P*_CCNA_00697_*-*lacZ* reporter only exhibited a 31% reduction in β–galactosidase activity under the same condition, possibly because as the preferred *in vivo* target of GcrA, residual GcrA that remains in the cell clings to the CCNA_00697 promoter (Figure 6B).

Next, we asked if a mutation of the GAnTC influences the promoter activity. To this end, all GAnTC sites in a promoter fragment of the *lacZ* reporter construct were mutated to GCnTC and the activity of the mutant promoters assayed by β–galactosidase measurements. The mutant promoters were crippled by 60–80% and immunoblotting showed the PodJ and MipZ failed to accumulate in cells lacking CcrM (Figure S6). Interestingly, the mutant P*_CCNA_00697_*-*lacZ* reporter showed a different response, retaining WT (100%) activity. Interestingly, the effect on P*_CCNA_00697_*-*lacZ* is mirrored for *ctrA*P1 whose activity was also unchanged by mutation of the GAnTC site to prevent CcrM-dependent methylation (Figure 6C) [19]. To test if this response was typical of GcrA-dependent promoters that are distal to the replication terminus, we analysed the methylation/GcrA dependency of another promoter, *tipF* (CCNA_00747), at a comparable location with respect to the origin of replication (). Unlike *ctrAP1* and the region at CCNA_00697, *tipF* promoter activity requires GcrA and an intact GAnTC methylation site (see below).

**Figure 7:**
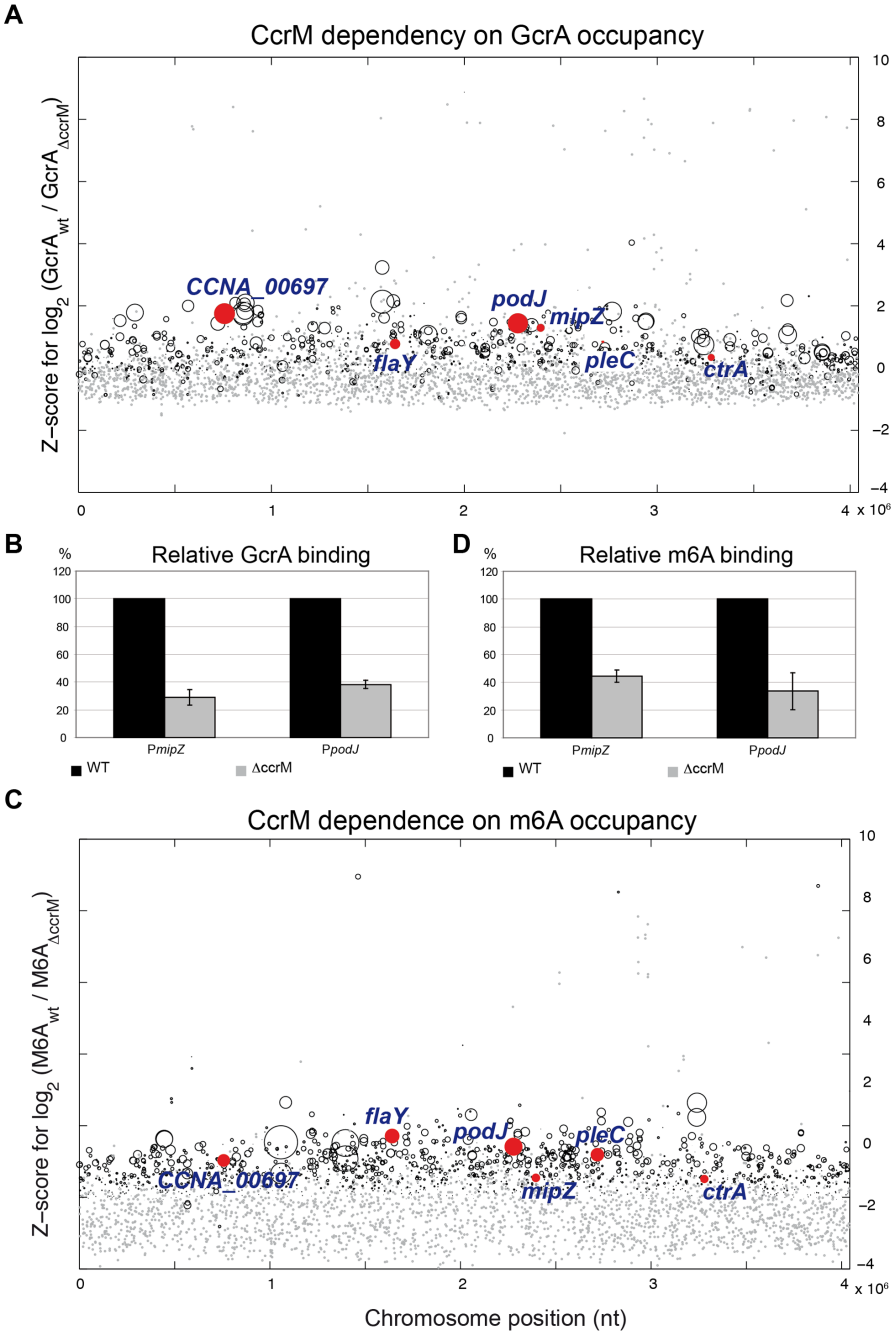
GcrA binding and m6A marks in promoters across the genome in WT and Δ*ccrM* cells. *(A)* Comparative ChIP-seq analysis of promoter regions bound by GcrA in WT and Δ*ccrM* cells. The y-axis shows the Z-score for the log2 ratio of the binding efficiency measured as sequence reads (Table S6). The x-axis indicates the position of the target sites along the chromosome (nt position). Red dots highlight genes analyzed in this work. Grey dots are promoters non-significantly changed. Black circles are significantly changed at a Z-score threshold = 2. The diameter of the circles is proportional to the coverage in the wild type. The promoter regions of genes of Figure 6 are highlighted in red. *(B)* Quantitative ChIP analysis to show the reduction in GcrA binding to P*_mipZ_* and P*_podJ_* in Δ*ccrM* cells compared to WT cells. *(C)* Comparative ChIP-seq analysis of promoter regions carrying m6A marks in WT and Δ*ccrM* cells. The y-axis shows the Z-score of the log2 of the binding efficiency measured as sequence reads (Table S7). The x-axis indicates the relative position of the target sites along the genome (nt position). The diameter of the circles is proportional to the coverage in the wild type. The promoter regions of genes of Figure 6 are highlighted in red. Grey dots are promoters non-significantly changed. Black circles are significantly changed. *(D)* Quantitative ChIP analysis to show the reduction in m6A marks to P*_mipZ_* and P*_podJ_* in Δ*ccrM* cells compared to WT cells.

To explore if methylation at GAnTC is required for GcrA to associate with its target sites *in vivo*, we compared the genome-wide promoter occupancy of GcrA in WT and Δ*ccrM* cells by ChIP-seq (Figure 7A). Analysis of the two data sets (Table S3) unearthed a major role of GAnTC methylation in directing GcrA to target promoters, with 80 loci requiring CcrM to be efficiently bound by GcrA, including *CCNA_00697*, *mipZ*, *podJ*, *flaY* (encoding a putative flagellar regulator), *pleC* (encoding a developmental histidine kinase/phosphatase) and to a lesser extent *ctrA*. However for *ctrA*P1, detailed analysis of the ChIP-Seq traces (Figure S7) revealed that GcrA binding dropped in proximity to the GAnTC methylation sites. Immunoblotting confirmed that no apparent difference in the GcrA steady-state levels were discernible in WT and Δ*ccrM* cells (Figure 7A).

To corroborate the ChIP-seq data, we performed ChIP analysis of WT and Δ*ccrM* cells and measured the abundance of precipitated (GcrA-bound) *mipZ* and *podJ* promoters by quantitative real-time PCR (qChIP). As shown in Figure 7B, in the absence of CcrM, GcrA occupancy is reduced by 70% and 60%, respectively.

If CcrM-dependent GAnTC methylation is required for GcrA binding to its targets, then the corresponding promoters should not be methylated in Δ*ccrM* cells. Because other methyltransferases might also contribute to adenosine methylation at the N6 position (m6A), we first determined the abundance of m6A across the genomes of WT and Δ*ccrM* cells by ChIP-Seq analysis using an m6A-specific polyclonal antibody (Figure 7C). This analysis revealed that chromosomal loci, particularly towards the replication terminus, carry abundant CcrM-dependent m6A marks (Table S3). We validated this conclusion by qChIP experiments for two promoters proximal to the terminus, P*_mipZ_* and P*_podJ_*_,_ (Figure 7D) and a distal one, P*_tipF_* (Figure S10).

### Methylation-dependent DNA binding of GcrA orthologs

**Figure 8:**
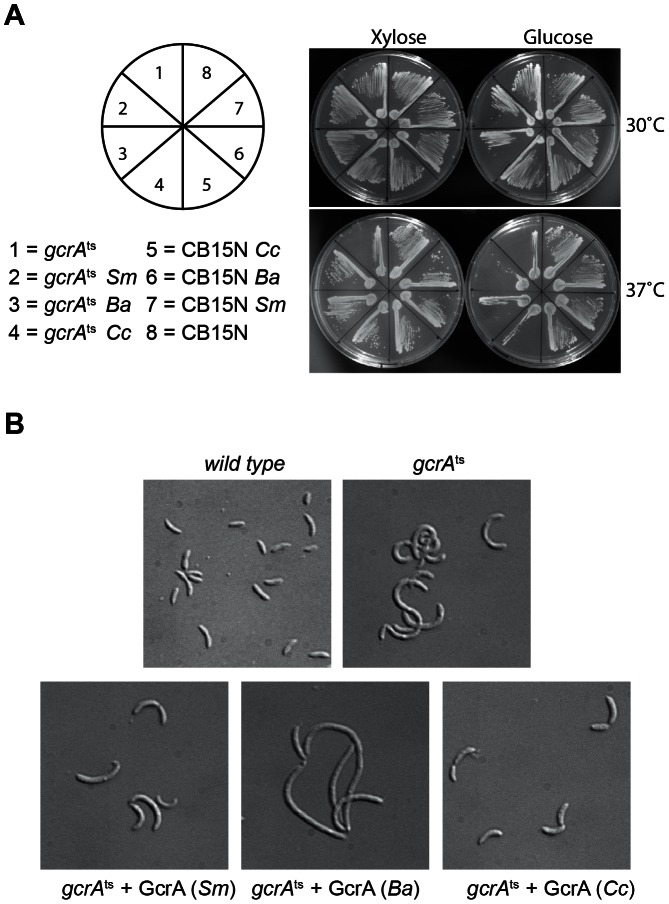
GcrA orthologs in *Alphaproteobacteria*. *(A)* Genetic complementation of *gcrA*ts (T10P) allele by *C. crescentus* (*Cc*), *B. abortus* (*Ba*) and *S. meliloti* (*Sm*) GcrA orthologs. *(B)* Orthologs, although able to support viability, caused diverse morphological phenotypes when expressed as the only functional copy of GcrA in *Caulobacter*. Morphologies at 30°C with and without xylose are not reported since cells appeared normal (data not shown).

**Figure 9:**
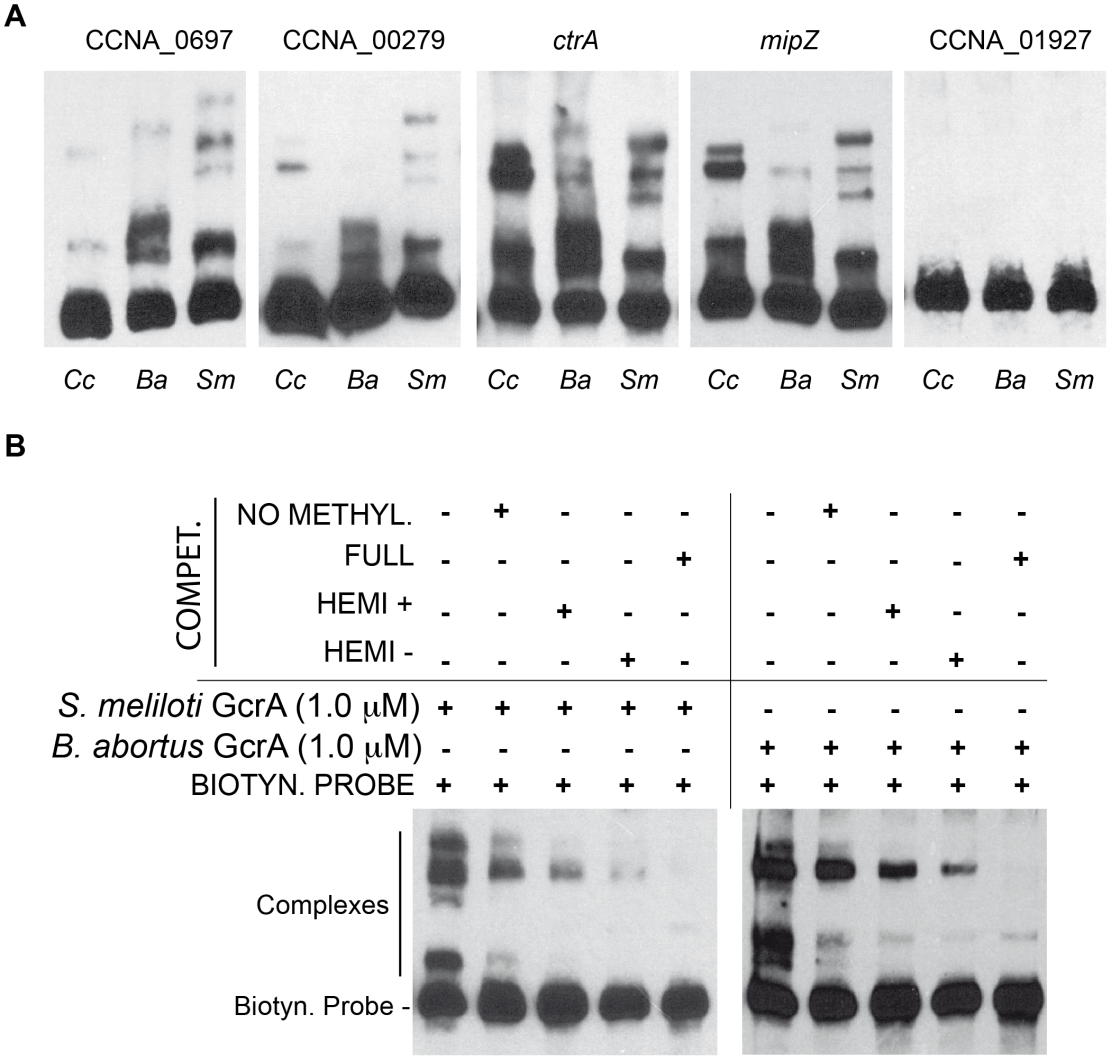
GcrAs in *Alphaproteobacteria* conserve specificity and GAnTC methylation-dependency. *(A)* Both *B. abortus* and *S. meliloti* GcrAs form DNA-protein complexes with similar specificity as observed in *C. crescentus* using probes described in Figure 2. *(B)* GcrAs binds the *ctrA* probe with different methylation states with the same differential efficiency as in *C. crescentus*.

To explore if GcrA-controlled functions are conserved across the *Alphaproteobacteria*, we introduced the GcrA ortholog [20] from *Brucella melitensis* biovar *abortus* 2308 (BAB1_0329) and *Sinorhizobium meliloti* Rm1021 (SMc02139) under the control of an xylose-inducible promoter on a low-copy plasmid [30] in *C. crescentus*, harbouring a temperature sensitive allele of *gcrA* with a Thr→Pro mutation at position 10 and evaluated their ability to support growth at the restrictive temperature [16]. As shown in Figure 8A, both *B. abortus* and *S. meliloti gcrA* orthologs are able to support viability of the strain *gcrA*ts at the restrictive temperature (37°C) following induction with xylose. Orthologs of GcrA from *S. meliloti* and *B. abortus*, although able to complement GcrA functions, revealed morphological diversities in *C. crescentus* (Figure 8B), likely owing to differences in abundance, activity and/or target specificity of these GcrA versions. Regardless, the complementation of the temperature-sensitive phenotype indicates that the function and target site specificity of GcrA orthologs are similar. We confirmed this result by testing directly the ability of GcrA orthologs to bind the *Caulobacter* target promoters. Therefore *B. abortus* and *S. meliloti* GcrA with an N-terminal His_6_ tag were purified from *E. coli* overexpression strains (Figure S2). EMSA experiments using target sites of *C. crescentus* GcrA revealed that these GcrAs are able to bind DNA efficiently and with the same apparent specificity (Figure 9A). Surprisingly the *B. abortus* and *S. meliloti* GcrA orthologs are able to form multiple complexes with different migration properties, likely due to structural and/or charge differences.

Finally we tested if binding of *B. abortus* and *S. meliloti* GcrAs is also stimulated by GAnTC methylation. EMSAs showed that methylation still affects GcrA binding, as fully methylated probes showed a stronger binding affinity in comparison with hemi-methylated and even more with non-methylated DNA. However the asymmetry in binding efficiency found for certain regions of DNA (Figure 9B) appeared different in other GcrAs with respect to the *C. crescentus* one.

### Concluding remarks

Despite the pervasive effects of adenosine methylation on transcription in various bacterial lineages, our understanding of the underlying operating principles in these systems is still limited. With the identification and genetic/biochemical characterizations of the m6A-marked promoters and the transcriptional effector(s) recognizing them, we elucidated a crucial first step towards understanding the physiological underpinnings and the evolution of these epigenetic control systems in *Alphaproteobacteria*. Studies with the methylation-sensitive *ctrA*P1 promoter of *C. crescentus* as model led to the appealing model that replication of a given locus by DNA polymerase converts the promoter from the fully to the hemi-methylated state at a specific time in the cell-cycle that is dictated by the relative distance of the promoter from the origin of replication (*ori*). For *ctrA*P1, the hemi-methylated state was thought to be a prerequisite for GcrA-mediated activation, while full (re)-methylation by CcrM at the end of S-phase was viewed as the event leading to promoter silencing.

**Figure 10:**
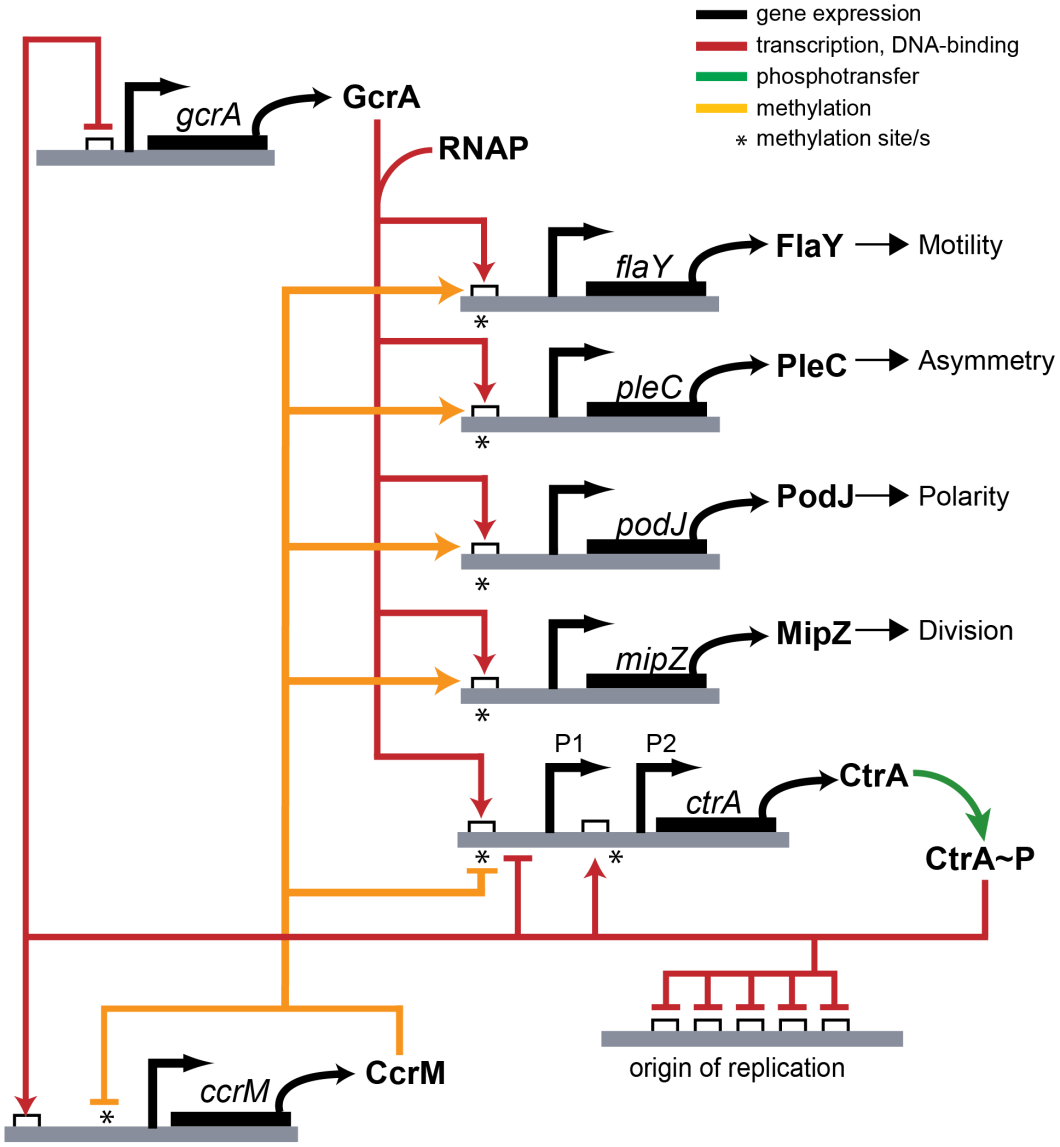
Model of transcriptional regulation by GcrA and CcrM. GAnTC methylation and GcrA/RNAP control the transcription of several promoters of important genes in *C. crescentus*.

Our experiments not only establish GcrA as a methylation-dependent transcription factor binding *ctrA*P1 and other promoters *in vivo* and *in vitro* (Figure 10), but they may suggest an elegant explanation for the methylation-induced regulation of expression. While activation of the hemi-methylated plus strand of *ctrA*P1 correlates with localized binding of GcrA to 13 nt over the −35 region, in the fully methylated state more than 40 nt of *ctrA*P1, are covered. Once the DNA replication fork moves through the fully methylated *ctrA* locus in the ensuing cell cycle the binding state for hemi-methylated DNA is reinstated. Methylation seems to help recruiting GcrA to promoters but GcrA might interact with RNAP even in the absence of target DNA. Perhaps in the hemi-methylated state this binding allows the initiation of transcription and release of the polymerase, while in the fully methylated state GcrA could sequester RNAP, preventing its movement through the coding sequence. It is likely that the partially unstructured dimeric GcrA adopts compacter structure upon interacting with methylated target DNA or possibly RNAP, thus conferring these properties.

Contrary to the view that CcrM- and GcrA-dependent control of *ctrA*P1 in response to DNA replication applies to all GcrA target promoters, we note that many *ori*-distal promoters (such as P*_mipZ_*, P*_podJ_*_,_ and P*_pleC_*), but also *terminus*-distal promoters (such as P*_tipF_*) that fire in early S-phase, also carry CcrM-dependent m6A marks that are required for the recruitment of GcrA. Promoters near the terminus will be replicated late in S-phase and are, thus, thought to reside in the hemi-methylated state only during a short window before the synthesis of CcrM. This begs the question what purpose of m6A marks at these promoters may serve, since methylation change by replication should not be temporally correlated with promoter activation. As many promoters of cell division (e.g., *mipZ*), motility (e.g., *flaY*) and polarity genes (e.g., *podJ*) carry m6A marks that are recognized by GcrA (Figure 10), CcrM-dependent methylation might serve as a quality control function or coupling mechanism to prepare these promoters for activation in the ensuing cell division cycle once GcrA is expressed. In the gammaproteobacterium *Vibrio cholerae*, the origin-binding protein of chromosome II RctB is recruited to sites carrying m6A marks that have been introduced by the GATC-specific Dam methyltransferase [4]. Thus, while full methylation also has been adopted for regulatory purposes, different effectors and processes have been selected during evolution.

## Materials and Methods

### Strains and growth conditions

*C. crescentus* strains were grown in peptone-yeast extract (PYE, rich medium) at 30°C [31] or 37°C as necessary, tetracycline (1 µg/ml), kanamycin (25 µg/ml), spectinomycin/streptomycin (100-5 µg/ml) 0.1% glucose, or 0.1% xylose, as required. *E. coli* strains were grown at 20°C or 37°C in LB broth supplemented with ampicillin (100 µg/ml), as necessary. Plasmids were transformed into *C. crescentus* and *E. coli* BL21 (DE3) by electroporation. Plasmids and strains are listed in Table S5. To construct the Δ*ccrM* mutant UG2212, the Δ*ccrM*::Ω mutation was transduced by ϕCr30-mediated generalized transduction from LS2144 [6] into NA1000. One transductant was selected on spectinomycin/streptomycin-containing medium and subjected to whole genome Illumina sequencing by Fasteris SA (Geneva, Switzerland). The genome sequence of UG2212 failed to reveal point mutations or insertions/deletions compared to the parent.

### Cloning

DNA fragments from *C. crescentus*, *S. meliloti* and *B. abortus* were amplified by PCR using cell lysates or genomic DNA for *Brucella* using Pfu-Turbo (Life Technologies, www.lifetechnologies.com/) following a protocol as recommended by the manufacturer. Primers are listed in the Figure S8. PCR products were then transferred in pENTR by Directional TOPO Cloning (Life technologies, www.lifetechnologies.com/), sequence verified and then transferred in pET derivatives His_6_-tagged destination vectors for *E. coli* BL21 expression, or pMR20 destination vector for xylose inducible expression in *C. crescentus* strains [30].

Transcriptional reporters were made by cloning PCR-amplified promoters fragments (Figure S8) into plac290 using *Eco*RI/*Xb*aI [32].

### Purification of GcrAs from *C. crescentus*, *S. meliloti*, and *B. abortus*

The full-length DNA fragment of GcrA was cloned into the pET15 derivative, pML375 vector [30], obtaining a recombinant protein with a His_6_-Thrombin cleavage site tag in N-terminus of the protein. Overexpression of GcrA was induced in *E. coli* BL21 (DE3) at OD (600 nm) 0.6–0.8 by 500 µM isopropyl-b-D-thiogalactoside (IPTG) O/N at 20°C. The cells were harvested by centrifugation and then resuspended in lysis buffer (PBS 1× pH 7.5, 0.2 M NaCl, 1 mM DTT, 0.1% Triton, supplemented with Complete Protease Inhibitor Cocktail (Roche, http://www.roche.com/) and DNase I (Euromedex, www.euromedex.com/) and lysed by Emulsiflex (Avestin, www.avestin.com/) at 10°C.

From the supernatant, GcrA was purified in two steps of purification, first, using Ni2+-nitrilotriacetate affinity resin (Ni-NTA) (Qiagen, www.qiagen.com/) equilibrated with lysis buffer and eluted by PBS 1× (pH 7.5), 0.5 M Imidazole, followed by Gel filtration step using HiLoad 16/60 Superdex 75 prep grade (GE Healthcare, www.gehealthcare.com/) equilibrated with running buffer (0.1 M Tris pH 8.5, 0.2 M NaCl, 5% Glycerol) optimized after DLS (See below the experimental procedure).

DLS measurements by the Zetasizer nano ZS (Malvern, www.malvern.com/) with an accuracy of 0.1°C were performed immediately after both the size exclusion step and the concentration step in order to find the best buffer composition. DLS was employed to estimate the thermo-stability of protein samples in different buffer solutions from to 15°C to 64°C, one degree steps. DLS was also used for the estimation of monodispersity of GcrA preparation.

### Limited proteolysis

Purified His_6_-GcrA was digested with proteases Thermolysin (Sigma-Aldrich, www.sigmaaldrich.com/) and Endoproteinase GluC V8 (New England Biolabs, www.neb.com/) (25°C with 0.5 mg/ml GcrA in 20 mM TRIS pH 8, 150 mM NaCl for digestion with Thermolysin and 20 mM Tris (pH 7.6), 1 mM CaCl_2_ in case of digestion with V8). The protease/substrate ratio was 1∶100 (w/w) in each case. At different time intervals, aliquots of the proteolysis reactions were stopped with loading buffer. The protein samples were then analyzed by SDS-PAGE and the fragments analyzed by Trypsin digestion and mass spectrometry. Proteolysis control of His_6_-ChpT [28] in presence of differentially methylated DNAs was performed as described above.

### Affinity chromatography for RNAP detection

Nickel columns loaded by His_6_-GcrA were also used for affinity chromatography showed in Figure 5B. A 1 ml HisPur-Ni-NTA Chromatography Cartridge (Qiagen, www.qiagen.com/), equilibrated with running buffer (0.1 M Tris pH 8.5, 0.15 M NaCl, 5% Glycerol) was loaded at 15°C with 23 mg of histidine-tagged *C. crescentus* GcrA (His_6_-GcrA) that was prepared as previously described, and washed with 15 volumes of running buffer. Meanwhile, 2 liters of *C. crescentus* cells (OD 600 nm of 0.6) were harvested by centrifugation (5000 rpm, 20 min, 4°C) and resuspended in 30 ml of lysis buffer (0.1 M Tris pH 8.5, 0.15 M NaCl, 1 mM DTT, 0.1% Triton, supplemented with Complete Protease Inhibitor Cocktail (Roche, www.roche.com/) and DNase I (Euromedex, www.euromedex.com/)) and lysed by Emulsiflex (Avestin, www.avestin.com/) at 10°C. The lysate was then centrifuged at 9500 rpm, 20 min, 4°C and the supernatant obtained was applied to the column. The column was eluted with running buffer NaCl gradient from 0.15 M and 1 M of NaCl. A last wash was done in presence of Imidazole (0,1 M Tris pH 8,5, 0,15 M NaCl, 5% Glycerol, 0.5 M Imidazole) in order to remove the His_6_-GcrA and proteins still bound at the column.

The negative control to this experiment was performed doing the same procedure with a 1 ml HisPur Ni-NTA Chromatography Cartridge without His_6_-GcrA.

The eluted samples were run in SDS-PAGE gel and transferred to nitrocellulose membrane. The membrane was blocked with PBS, 0.1% NP-40 and 3% dry milk for 1 hour at room temp. The membrane was incubated with anti-RNA polymerase B-subunit antibody (Thermo Scientific, www.pierce-antibodies.com/) against the β-subunit (1∶5000) at 4°C overnight. Each membrane was washed 5 times each for 10 min with PBS containing 0.1% NP-40, followed by incubation with the secondary antibody (1∶50,000) for 45 min. The membrane was developed following the procedure described under immunoblot section.

### DNA binding *in vitro* assays

EMSAs were performed using the LightShift Chemiluminescent EMSA Kit (Thermo Scientific). Briefly, different versions of GcrA were incubated at room temperature in 10 mM Tris pH 7.5, 100 mM KCl, 0.5 mM DTT, 50 ng/µl poly(dI-dC), and 0.05% Nonidet P-40 binding buffer with 5 fmol of a biotin-labeled DNA fragment for 25 minutes.

After 25 min incubation at room temperature, samples were resolved by a 10% non-denaturing polyacrylamide gel prepared in TBE buffer (450 mM Tris, 450 mM boric acid and 0.01 mM EDTA). The samples were blotted onto a 0.45-µm Biodyne B nylon membrane (Thermo Scientific, www.piercenet.com/) at constant current of 300 mA for 45 min at 4°C, and then cross-linked to the membrane using a 312 nm UV Transilluminator (Uvitec, www.uvitec.com.) for 10 min. Membranes were processed as recommended in the Chemiluminescent Nucleic Acid Detection Module Kit (Thermo Scientific, www.piercenet.com/).

Competitive EMSAs were performed as described above, adding a preincubation step of 20 min at room temperature of GcrA and competitor DNAs before the usual 25 min GcrA/biotin-labeled DNA fragment incubation.

EMSA in presence of RNA polymerase core enzyme (Epicentre, www.epibio.com/) was performed by pre-incubating GcrA in presence of RNAP for 20 min at room temperature before the usual incubation with biotin-labeled DNA.

For detecting the binding region of GcrA, a 120 bp probe from *ctrA*P1 (Figure S8) was synthesized and labeled with Fam-6 (Eurogentec, www.eurogentec.com/). Single stranded probes containing m6A were also synthesized, which were later assembled into double stranded probes in different combinations. Five fmoles of probes were incubated at room temperature with increasing concentrations of purified GcrA as done with EMSA for 30 min. The samples were digested with approximately 7U of DNaseI (Euromedex, www.euromedex.com/) at room temp for 3 min. DNaseI was inactivated by adding 0.1 M EDTA followed by incubation at 75°C for 10 min. The digested fragments were eluted using the mini-elute columns (Qiagen, www.qiagen.com/). The samples were run in a 3130 Genetic Analyzer (Life Technologies) as described before [33], analyzed by GelQuest (SequentiX, www.sequentix.de/). Sequencing reactions were also performed using Thermo Sequenase Dye Primer Manual Cycle Sequencing Kit (Affymetrix, www.affymetrix.com/) using the probe region as a template and a sequencing primer labeled with FAM at the 5 primes.

### β-Galactosidase assays

β-Galactosidase assays were performed at 30°C as described earlier [34], [35].

### Immunoblots

PVDF (polyvinylidenfluoride) membranes (Merck-Millipore, www.merckmillipore.com) were blocked with PBS, 0.05% tween 20 and 5% dry milk for 1 h and then incubated for 1 h with the primary antibodies diluted in PBS, 0.05% tween 20, 5% dry milk. The membranes were washed 4 times for 5 min in PBS and incubated 1 h with the specific secondary antibody diluted in PBS, 0.05% tween 20 and 5% dry milk. The membranes were finally washed again 4 times for 5 min in PBS and revealed with Immobilon Western Blotting Chemoluminescence HRP substrate (Merck Millipore, www.merckmillipore.com/). The different antisera were used at the following dilutions: anti-CcrM (1∶10;000), anti-DnaA (1∶10;000), anti-GcrA (1∶10,000), anti-CtrA (1∶10,000). Anti-RNA polymerase beta antibodies (Abcam, www.abcam.com/) were used using the protocol described in “*Affinity chromatography for RNAP detection*”.

### Quantitative Chromatin Immunoprecipitation (qChIP) assays

Mid-log phase cells were cross-linked in 10 mM sodium phosphate (pH 7.6) and 1% formaldehyde at room temperature for 10 min and on ice for 30 min thereafter, washed thrice in phosphate buffered saline (PBS) and lysed in a Ready-Lyse lysozyme solution (Epicentre, Madison, WI) according to the manufacturer's instructions. Lysates were sonicated (Sonifier Cell Disruptor *B*-*30*) (Branson Sonic Power. Co., www.bransonic.com/) on ice using 10 bursts of 20 sec at output level 4.5 to shear DNA fragments to an average length of 0.3–0.5 kbp and cleared by centrifugation at 14,000 rpm for 2 min at 4°C. Lysates were normalized by protein content, diluted to 1 mL using ChIP buffer (0.01% SDS, 1.1% Triton X-100, 1.2 mM EDTA, 16.7 mM Tris-HCl [pH 8.1], 167 mM NaCl plus protease inhibitors (Roche, www.roche.com/) and pre-cleared with 80 µL of protein-A agarose (Roche, www.roche.com/) and 100 µg BSA. Ten % of the supernatant was removed and used as total chromatin input DNA.

Polyclonal antibodies to GcrA [16] and m6A (Synaptic Systems GmbH, www.sysy.com/) were added to the remains of the supernatant (1∶1,000 dilution), incubated overnight at 4°C with 80 µL of protein-A agarose beads pre-saturated with BSA, washed once with low salt buffer (0.1% SDS, 1% Triton X-100, 2 mM EDTA, 20 mM Tris-HCl (pH 8.1), 150 mM NaCl), high salt buffer (0.1% SDS, 1% Triton X-100, 2 mM EDTA, 20 mM Tris-HCl (pH 8.1), 500 mM NaCl) and LiCl buffer (0.25 M LiCl, 1% NP-40, 1% sodium deoxycholate, 1 mM EDTA, 10 mM Tris-HCl (pH 8.1) and twice with TE buffer (10 mM Tris-HCl (pH 8.1) and 1 mM EDTA). The protein•DNA complexes were eluted in 500 µL freshly prepared elution buffer (1% SDS, 0.1 M NaHCO_3_), supplemented with NaCl to a final concentration of 300 mM and incubated overnight at 65°C to reverse the crosslinks. The samples were treated with 2 µg of Proteinase K for 2 h at 45°C in 40 mM EDTA and 40 mM Tris-HCl (pH 6.5). DNA was extracted using phenol∶chloroform∶isoamyl alcohol (25∶24∶1), ethanol-precipitated using 20 µg of glycogen as carrier and resuspended in 100 µl of water.

### Real-time PCR

Real-time PCR was performed using a Step-One Real-Time PCR system (Applied Biosystems, www.appliedbiosystems.com/) using 5% of each ChIP sample (5 µL), 12.5 µL of SYBR green PCR master mix (Quanta Biosciences, www.quantabio.com/), 0.5 µL of primers (10 µM) and 6.5 µL of water per reaction. Standard curve generated from the cycle threshold (Ct) value of the serially diluted chromatin input was used to calculate the percentage input value of each sample. Average values are from triplicate measurements done per culture. The final data was generated from three independent cultures. The DNA regions analyzed by real-time PCR were from nucleotide −167 to +43 relative to the start codon of *podJ*, from −208 to +9 relative to the start codon of *mipZ*, from −185 to −16 relative to the start codon of *ctrA*.

## Supporting Information

Figure S1 Alignment of GcrA orthologs from *C. crescentus*, *B. abortus*, *Agrobacterium tumefaciens* and *S. meliloti.* Alignment was performed using ClustalW [36].
(PDF)

Figure S2 Purification of GcrA from *C. crescentus*, *B. abortus* and *S. meliloti*. SDS-PAGE gels of purifications at different steps: NI = Non-induced sample; I = Induced by IPTG; NI-NTA = purification by nickel columns; Gel FI = purification after Gel filtration (procedure is described in Materials and Methods).
(PDF)

Figure S3 Secondary structure prediction of *C. crescentus* GcrA by SOPMA. In the upper part, the amino acid sequence is shown with the corresponding prediction below as explained in the legend. Also the overall percentage of secondary elements is given (See main text for more details).
(PDF)

Figure S4 GcrA is partially unfolded dimer with elongated shape. *(A)* Small angle scattering (SAXS) data from GcrA in solution: (from upper-left corner in clockwise order) i. Experimental scattering curve (values in Table S1). Intensity at q = 0 (I_0_) obtained by extrapolation of the curve at law value of q is directly related to the Molecular Weight (MW) of the particle that can thus be estimated. For GcrA the estimated MW corresponds to a dimeric organization of the molecule (ca. 42 KDa). ii. The Guignier plot, which represents the logarithm of scattering intensity versus q^2^, is linear over a restricted region attesting that there is no aggregation of GcrA in solution. The radius of gyration (R_G_) of GcrA (43.45 Å?), estimated from the slope, provides information about the average size of the particle. iii. The Kratky plot representation of the intensity curve (q^2^I(q) versus q) assess the globular nature of the polypeptide chain. Kratky plot for GcrA shows the typical shape observed for non or partially globular molecules having significant flexibility. iv. The distance distribution function P(*r*) calculated by the program GNOM [33] is a histogram of all interatomic distances *r* within the molecule. The maximal value of *r* (D_max_) of GcrA (152 Å?) corresponds to the maximal diameter of the protein and gives information on the shape of the particle. In the case of GcrA, P(*r*) shows that the molecule has a rather elongated shape. *(B)* Limited proteolysis of GcrA using Thermolysin (left) and V8 (right). Asterisks correspond to resistant bands that were analyzed by MS and the interval between parentheses is the amino acid region of GcrA.
(PDF)

Figure S5 EMSA using *E. coli* RNA polymerase core enzyme. RNAP is able to bind the GcrA-DNA (*mipZ* promoter) complex, as visualized by the formation of a slower migration rate band as the amount of RNA polymerase increased.
(PDF)

Figure S6 Immunoblots anti-MipZ and PodJ in wild type, Δ*ccrM* and *gcrA* depletion strains. Immunoblots showing that the steady-state levels of PodJ and MipZ drop without CcrM and GcrA using polyclonal antibodies to these proteins.
(PDF)

Figure S7 Binding of GcrA to the *ctrA*P1 promoter drops after CcrM depletion. Using data represented of Figure 7A, we zoomed into the *ctrA*P1 promoter. Genetic map of the *ctrA* promoter region is below the plot. Black and red lines denote the traces of the m6A signals in WT and Δ*ccrM* cells, respectively, as determined by ChIP-seq in the *ctrA*P1 promoter.
(PDF)

Figure S8 Primers and probes sequences.
(PDF)

Figure S9 Calculation of dissociation constants of EMSA probes. A. EMSAs using probes reported in Figure 2. B. EMSAs using *ctrA* and *mipZ* promoters methylated probes of Figure 3. C. Table with values of Kds. All probes were at 0.125 nM concentration.
(PDF)

Figure S10 Quantitative ChIp analysis of the *tipF* promoter. Results show the reduction in m6A marks to P*_tipF_* in Δ*ccrM* cells compared to WT cells.
(PDF)

Protocol S1 SAXS and data analysis protocols.
(PDF)

Protocol S2 ChIP–Seq and data analysis protocols.
(PDF)

Table S1 SAXS data.
(PDF)

Table S2 Best peaks (1 kbp long) derived from GcrA in wild type ChIP–Seq.
(PDF)

Table S3 Best promoter regions derived from GcrA in wild type, GcrA in Δ*ccrM* m6A in wild type and m6A in Δ*ccrM* ChIp–Seqs.
(PDF)

Table S4 Transcription of *lacZ* by the *tipF* promoter in different genetic backgrounds.
(PDF)

Table S5 Strains and plasmids.
(PDF)

Table S6 Log2 ratios of Figure 7A.
(PDF)

Table S7 Log2 ratios of Figure 7C.
(PDF)
