# Supplementary material for: DNA Binding of the Cell Cycle Transcriptional Regulator GcrA Depends on N6-Adenosine Methylation in Caulobacter crescentus and Other Alphaproteobacteria
Source: PLoS Genet. 2013 May 30;9(5):e1003541. doi: 10.1371/journal.pgen.1003541 (PMC3667746; doi:10.1371/journal.pgen.1003541)
Supplement: Figure S1 — Alignment of GcrA orthologs from C. crescentus, B. abortus, Agrobacterium tumefaciens and S. meliloti. Alignment was performed using ClustalW [36]. (PDF) [file pgen.1003541.s001.pdf]

Figure S1

CLUSTAL multiple sequence alignment

```
Sinorhizobium      MNWTDERVEKLKKLWSEGLSASQIAAQLGGVSRNAVIGKVHRLSLPGRAKAGG-STAAAA
59
Agrobacterium      MNWTDERVEKLKKLWSEGLSASQIAAQLGGVSRNAVIGKVHRLNLPGRVKAGGPVTSARS
60
Brucella           MNWTDERVELLKKLWSEGLSASQIAAQLGGVSRNAVIGKVHRLKLSGRGKTTTAAPRSKK
60
Caulobacter        MSWTDERVSTLKKLWLDGLSASQIAKQLGGVTRNAVIGKVHRLGLSGRAAPSQPARPAFK
60
                   *.******. ***** :******** *****:*********** *.**  .      :

Sinorhizobium      RPKR-ATSAPRAPN--YAARS---VTRTVTRTAGATALKEELAVDLTIDQELQIDRNIVL
113
Agrobacterium      APKRTAAPAPRATT--FAGRVNAAPARILTRSNAATALHEEIDIETAQVLDYVPSKNVVT
118
Brucella           VNTPAAAPRPAVQNNNSGTHTTTMRTATVTKTVGATALQMEYATEVVAETVIKPASDVVV
120
Caulobacter        APRPARPAAQAMPS----------APRRVTPVEAPTS------VPVAAAPAPLPAFRHEE
104
                         ..     .           .  :*   ..*:         .

Sinorhizobium      PMSRRLELTELTERTCKWPIGDPLKEEFHFCGNDSPEASPYCTFHARLAYQPSAERRRMR
173
Agrobacterium      PISRRLTLTELTERTCKWPVGDPLKDDFHFCGCEALESSPYCKFHAKLAYQPVSERRKA-
177
Brucella           PISRHLTLLQLSERTCKWPIGDPLNEDFHFCGNESGEASPYCSYHSRLAFQPTAERRRAR
180
Caulobacter        PGS--ATVLTLGAHMCKWPIGDPSSEGFTFCGRRSSEG-PYCVEHARVAYQPQQTKKKSG
161
                   * *    :  *  : ****:*** .: * ***  : *. ***  *:::*:**   :::

Sinorhizobium      ------------
Agrobacterium      ------------
Brucella           ------------
Caulobacter        GAELARSLRRYI 173
```

"*" means that the residues or nucleotides in that column are identical in all sequences in the alignment.

":" means that conserved substitutions have been observed.

"." means that semi-conserved substitutions are observed.
