# Supplementary figures and images for: DNA Binding of the Cell Cycle Transcriptional Regulator GcrA Depends on N6-Adenosine Methylation in Caulobacter crescentus and Other Alphaproteobacteria
Source: PLoS Genet. 2013 May 30;9(5):e1003541. doi: 10.1371/journal.pgen.1003541 (PMC3667746; doi:10.1371/journal.pgen.1003541)

FIGURE S2

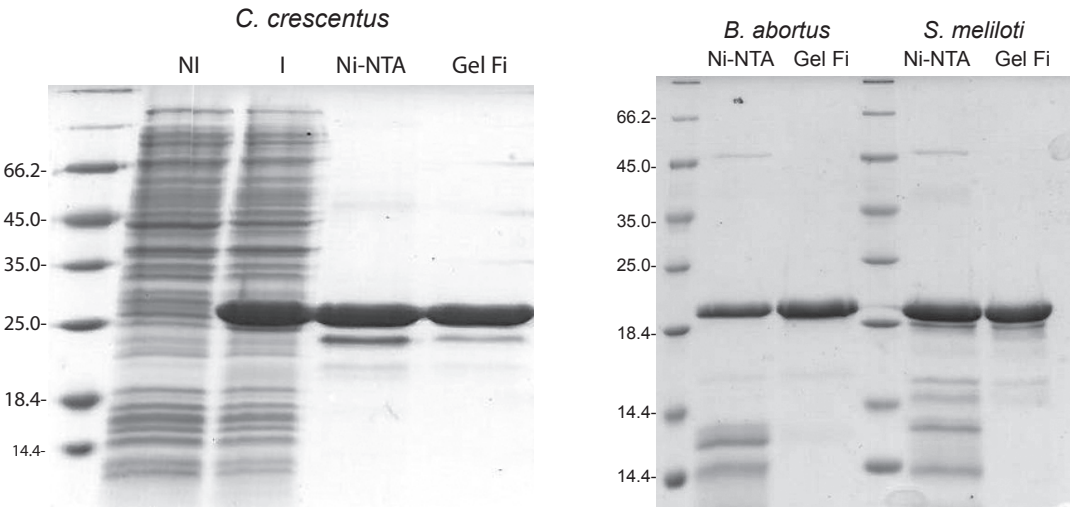

Supplement: Figure S2 — Purification of GcrA from C. crescentus, B. abortus and S. meliloti. SDS-PAGE gels of purifications at different steps: NI = Non-induced sample; I = Induced by IPTG; NI-NTA = purification by nickel columns; Gel FI = purification after Gel filtration (procedure is described in Materials and Methods). (PDF) [file pgen.1003541.s002.pdf]

FIGURE S4

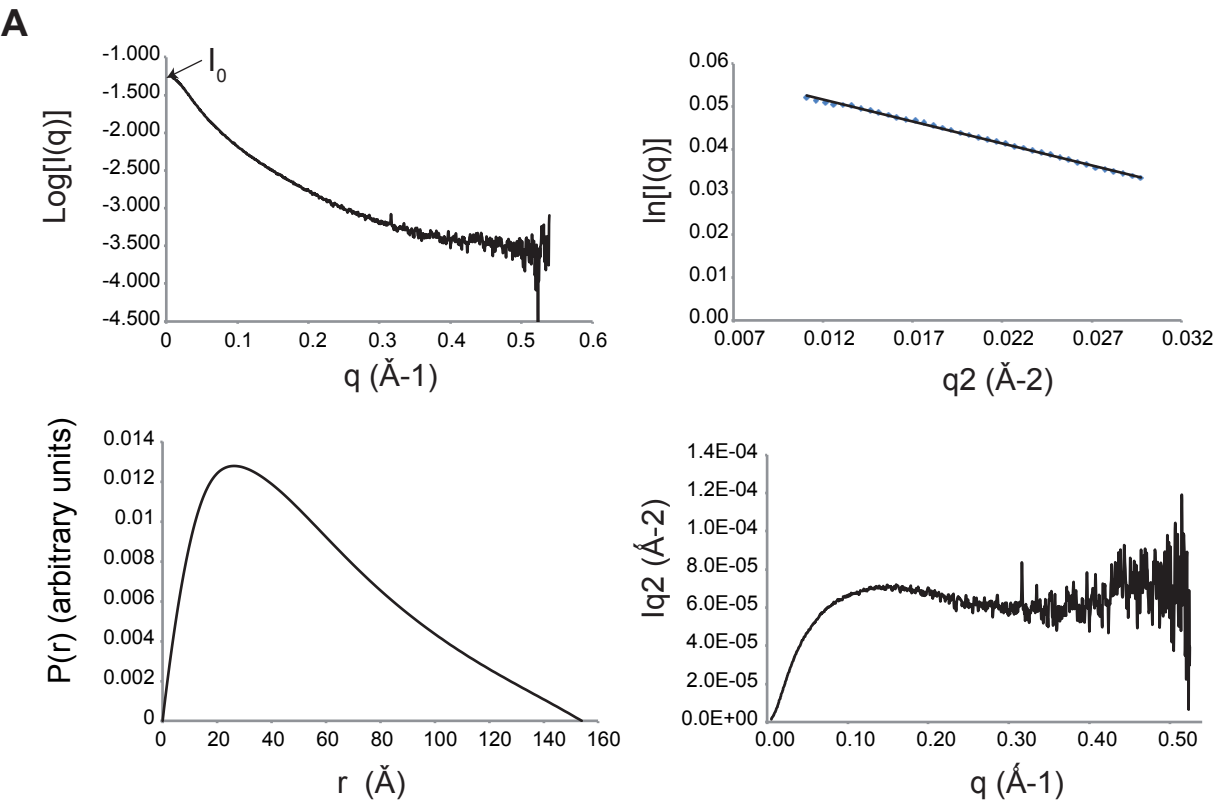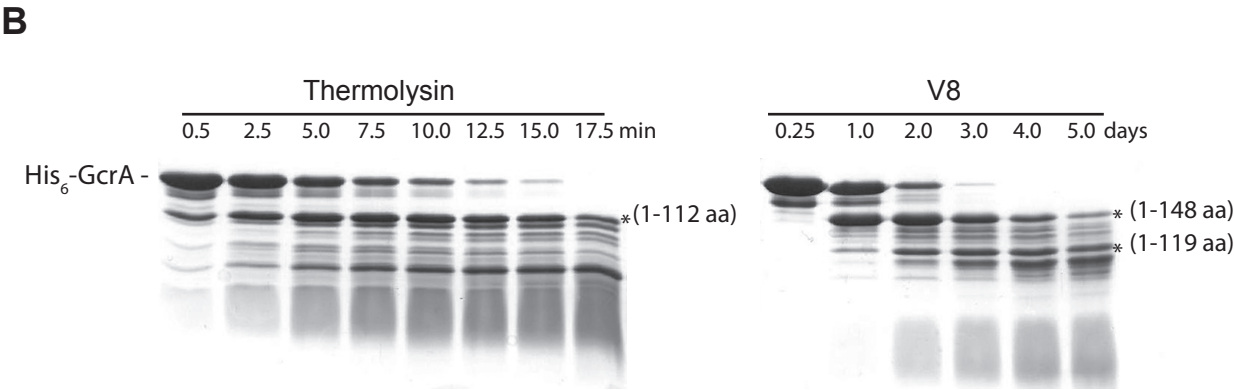

Supplement: Figure S4 — GcrA is partially unfolded dimer with elongated shape. (A) Small angle scattering (SAXS) data from GcrA in solution: (from upper-left corner in clockwise order) i. Experimental scattering curve (values in Table S1). Intensity at q = 0 (I0) obtained by extrapolation of the curve at law value of q is directly related to the Molecular Weight (MW) of the particle that can thus be estimated. For GcrA the estimated MW corresponds to a dimeric organization of the molecule (ca. 42 KDa). ii. The Guignier plot, which represents the logarithm of scattering intensity versus q2, is linear over a restricted region attesting that there is no aggregation of GcrA in solution. The radius of gyration (RG) of GcrA (43.45 Å?), estimated from the slope, provides information about the average size of the particle. iii. The Kratky plot representation of the intensity curve (q2I(q) versus q) assess the globular nature of the polypeptide chain. Kratky plot for GcrA shows the typical shape observed for non or partially globular molecules having significant flexibility. iv. The distance distribution function P(r) calculated by the program GNOM [33] is a histogram of all interatomic distances r within the molecule. The maximal value of r (Dmax) of GcrA (152 Å?) corresponds to the maximal diameter of the protein and gives information on the shape of the particle. In the case of GcrA, P(r) shows that the molecule has a rather elongated shape. (B) Limited proteolysis of GcrA using Thermolysin (left) and V8 (right). Asterisks correspond to resistant bands that were analyzed by MS and the interval between parentheses is the amino acid region of GcrA. (PDF) [file pgen.1003541.s004.pdf]

FIGURE S5

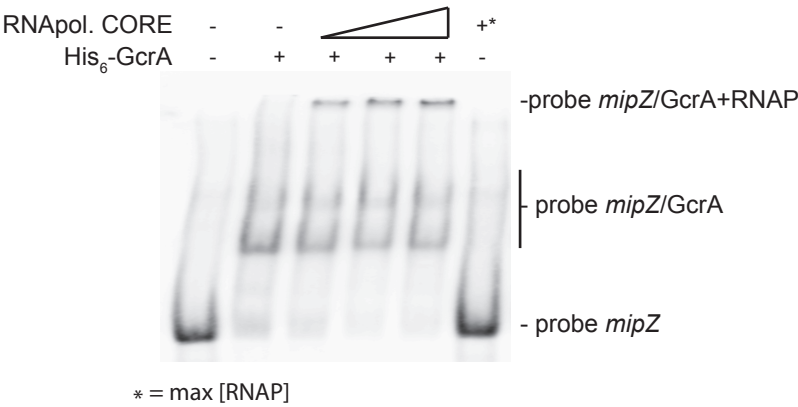

Supplement: Figure S5 — EMSA using E. coli RNA polymerase core enzyme. RNAP is able to bind the GcrA-DNA (mipZ promoter) complex, as visualized by the formation of a slower migration rate band as the amount of RNA polymerase increased. (PDF) [file pgen.1003541.s005.pdf]

FIGURE S6

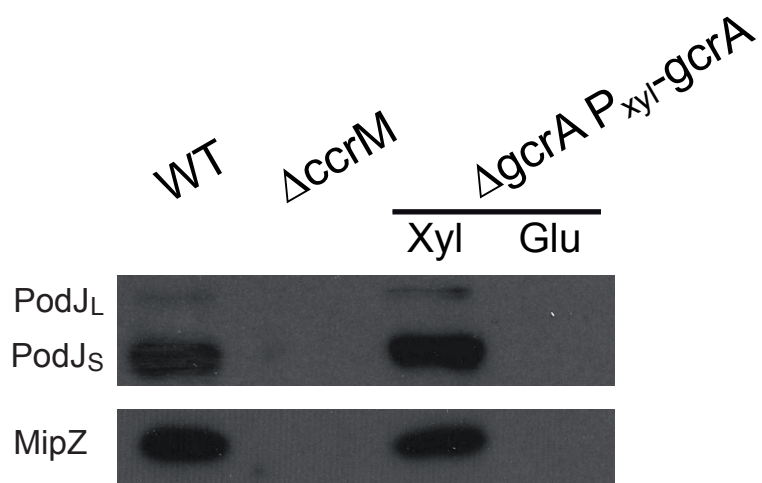

Supplement: Figure S6 — Immunoblots anti-MipZ and PodJ in wild type, ΔccrM and gcrA depletion strains. Immunoblots showing that the steady-state levels of PodJ and MipZ drop without CcrM and GcrA using polyclonal antibodies to these proteins. (PDF) [file pgen.1003541.s006.pdf]

FIGURE S7

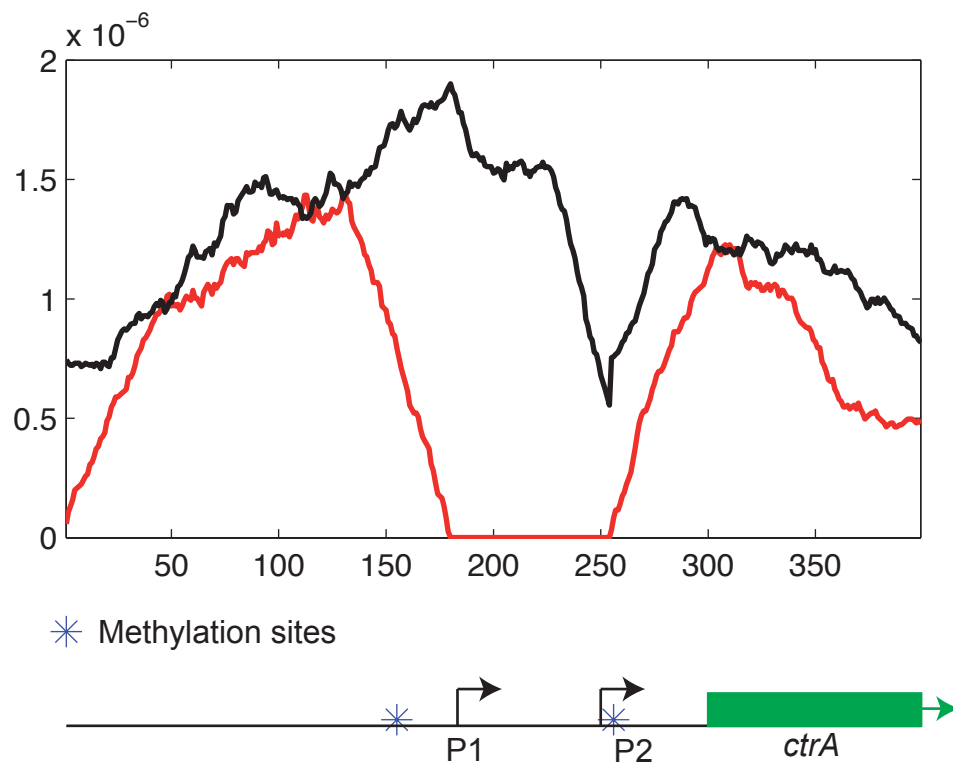

Supplement: Figure S7 — Binding of GcrA to the ctrAP1 promoter drops after CcrM depletion. Using data represented of Figure 7A, we zoomed into the ctrAP1 promoter. Genetic map of the ctrA promoter region is below the plot. Black and red lines denote the traces of the m6A signals in WT and ΔccrM cells, respectively, as determined by ChIP-seq in the ctrAP1 promoter. (PDF) [file pgen.1003541.s007.pdf]

Figure S10

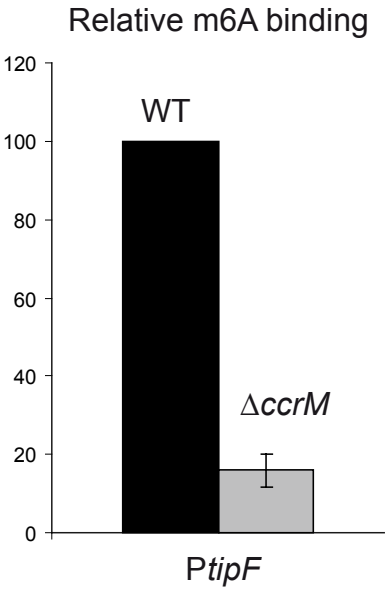

Supplement: Figure S10 — Quantitative ChIp analysis of the tipF promoter. Results show the reduction in m6A marks to PtipF in ΔccrM cells compared to WT cells. (PDF) [file pgen.1003541.s010.pdf]
