# Supplementary material for: DNA Binding of the Cell Cycle Transcriptional Regulator GcrA Depends on N6-Adenosine Methylation in Caulobacter crescentus and Other Alphaproteobacteria
Source: PLoS Genet. 2013 May 30;9(5):e1003541. doi: 10.1371/journal.pgen.1003541 (PMC3667746; doi:10.1371/journal.pgen.1003541)
Supplement: Figure S3 — Secondary structure prediction of C. crescentus GcrA by SOPMA. In the upper part, the amino acid sequence is shown with the corresponding prediction below as explained in the legend. Also the overall percentage of secondary elements is given (See main text for more details). (PDF) [file pgen.1003541.s003.pdf]

FIGURE S3

```
          10        20        30        40        50        60        70
           |         |         |         |         |         |         |
MSWTDERVSTLKKLWLDGLSASQIAKQLGGVTRNAVIGKVHRLGLSGRAAPSQPARPAFKAPRPARPAAQ
hccchhhhhhhhhhhhttcchhhhhhhhhhcchhhhhhhhhetccccccccccccccccccccccccccc

AMPSAPRRVTPVEAPTSVPVAAAPAPLPAFRHEEPGSATVLTLGAHMCKWPIGDPSSEGFTFCGRRSSEG
ccccccccccccccccccccccccccccccccccttceeeeehcccccccccccccccceeetcccccc

PYCVEHARVAYQPQQTKKKSGGAELARSLRRYI
cccchhheeeccccccccccchhhhhhhhhhhh


Legend:
   Alpha helix     (h): 28.90%
   Extended strand (e): 6.94%
   Beta turn       (t): 3.47%
   Random coil     (c): 60.69%
```
