# Supplementary material for: DNA Binding of the Cell Cycle Transcriptional Regulator GcrA Depends on N6-Adenosine Methylation in Caulobacter crescentus and Other Alphaproteobacteria
Source: PLoS Genet. 2013 May 30;9(5):e1003541. doi: 10.1371/journal.pgen.1003541 (PMC3667746; doi:10.1371/journal.pgen.1003541)
Supplement: Figure S8 — Primers and probes sequences. (PDF) [file pgen.1003541.s008.pdf]

Figure S8

Primers for GcrAs cloning:

S. meliloti Rm1021 (SMc02139)
pSMc02139-CACC- fw
CACCATGAACTGGACTGACGAGCG
pSMc02139-rev
AGCGGAATGAGCAAGGCG

B. melitensis biovar abortus (BAB1_0329)
pBAB1_0329- CACC- fw
CACCATGAACTGGACAGACGAGCG
pBAB1_0329- rev
GGGGACGCTTCCTGAATTCA

C. crescentus CB15N (CC2245)
pCC2245-NdeI-fw
CACCATGAGCTGGACCGACGAA
pCC2245-rev2
GTCATCCCGCGCTTTATGC

Oligonucleotide pairs for *lacZ* reporter constructions 5′- 3:

P_CC_2165 (mipZ):
AAA AAA GAA TTC GTG CAG GGC GCC CAT GGT CGG AA
AAA AAA TCT AGA CGA CGT CGT TGT CGC TGA GGT TCA A

P_CC_2045 (podJ):
AAA AAA GAA TTC ATC GGA CGC GCG CCC AGC CAG CCG AA
AAA AAA TCT AGA ATC CAG CGC CAG CGC GAC GCG ACC AA

P_CCNA_00697:
AAA AAA GAA TTC AAG GGG TCG TGG ACC GAC AAG AA
AAA AAA TCT AGA ATC CCT CTC TCC ATG AGA GAG GGA TT

P_CC_2482 (pleC):
AAA AAA GAA TTC CTG GCG GAC GAG ATC CTC AA
AAA AAA TCT AGA GGG CTG GGC CAT GGC CCA A

P_CC_1465 (flaY):
AAA AAA GAA TTC CTG GAT CGC GCC TTC CTT GA
AAA AAA TCT AGA TTG AGC GCC TGA TAG GTG GCG AAC A

EMSA probes (70 bp) 5′- 3:

CCNA_0697:
ACGTTTCCCCGAACAGGGGCGAAAC**GAATC**GGGACC**GAATC**AGCGACGTTCGCCGATTCAGTATTTGTTC

ctrA promoter:
AGACTGGTTAATGGTGAATGTTTCCCGTCGGAGGAATGGTTAATCTGATTTGC**GAATC**GGGTGCAAGCCG

mipZ promoter:
TGGCTCGGATCCTTCTGCGTCGC**GACTC**AGC**GACTC**TTAATCGAAGGTTAACACGATGTTTCCCGCCCCC

CCNA_0278 and CCNA_0279:
CGCGCGCTGGCGTCGGCCGGCGCAGGCTGCGGCTCATGACCACGCCAAGCCGTCGCATCCTGATCTTGGT

CCNA_1926 and CCNA_1927:
TTCTGAAGATTTCGCTAGCCTTCCCAAGGGGCAAAGGCGCCTAGACTCGCCCCCAACGAACACAGGGAGG

DNase I foot printing region (*ctrAP1* promoter), 120 bp, 5′- 3:

cggtgaaacccttcggccactttgccggagagttaatttaagactggttaatggtgaatgtttcccgtcggaggaatggttaatctgatttgcg
Aatcgggtgcaagccgcgtcggagcc
