# Supplementary material for: DNA Binding of the Cell Cycle Transcriptional Regulator GcrA Depends on N6-Adenosine Methylation in Caulobacter crescentus and Other Alphaproteobacteria
Source: PLoS Genet. 2013 May 30;9(5):e1003541. doi: 10.1371/journal.pgen.1003541 (PMC3667746; doi:10.1371/journal.pgen.1003541)
Supplement: Figure S9 — Calculation of dissociation constants of EMSA probes. A. EMSAs using probes reported in Figure 2. B. EMSAs using ctrA and mipZ promoters methylated probes of Figure 3. C. Table with values of Kds. All probes were at 0.125 nM concentration. (PDF) [file pgen.1003541.s009.pdf]

FIGURE S9

A

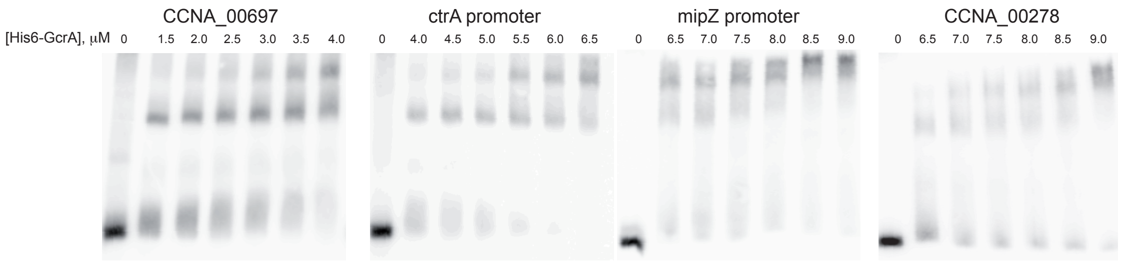

B

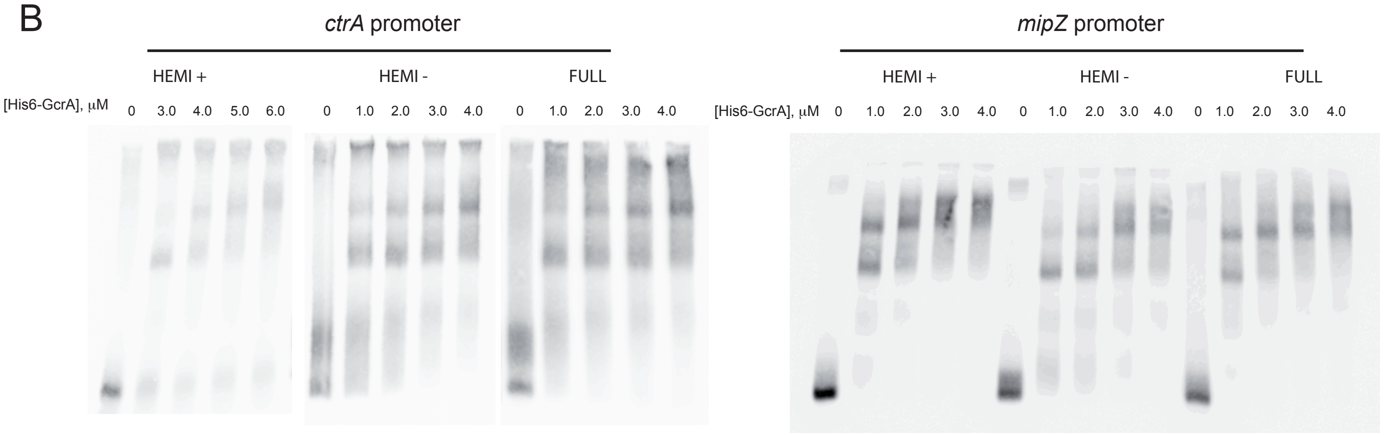

C

	**Kd (μM) (Error +/- 0.5)**
CCNA_00697	4
promoter of *ctrA*	6.5
promoter of *mipZ*	8.5
CCNA_00287	> 9
promoter of *ctrA* - HEMI PLUS	4
promoter of *ctrA* - HEMI MINUS	4
promoter of *ctrA* - FULLY	2
promoter of *mipZ* - HEMI PLUS	2
promoter of *mipZ* - HEMI MINUS	3
promoter of *mipZ* - FULLY	1
