# Supplementary material for: DNA Binding of the Cell Cycle Transcriptional Regulator GcrA Depends on N6-Adenosine Methylation in Caulobacter crescentus and Other Alphaproteobacteria
Source: PLoS Genet. 2013 May 30;9(5):e1003541. doi: 10.1371/journal.pgen.1003541 (PMC3667746; doi:10.1371/journal.pgen.1003541)
Supplement: Protocol S1 — SAXS and data analysis protocols. (PDF) [file pgen.1003541.s011.pdf]

Protocol S1

SAXS analysis

SAXS experiments were conducted on the SWING beamline at the SOLEIL synchrotron. The Aviex charge-coupled device detector was positioned at a distance of 1.8 m. Data were collected in the q-range 0.008–0.3 Å−1 (λ = 1.033 Å) (q = 4πsinθ λ−1, where 2θ is the scattering angle) in a fixed-temperature (15°C) quartz capillary (diameter of 1.5 mm and a wall thickness of 10 µm.

GcrA (9.9 mg/ml) was injected onto a size-exclusion column (SEC-3, 150 Å, Agilent), using an Agilent HPLC system, and eluted (0.1 M TRIS pH 8.5, 150 mM NaCl and 10% glycerol) directly into the SAXS flow-through capillary cell at a flow rate of 0.15 ml min−1. SAXS data were collected continuously, with frame duration of 1.5 s and a dead time between frames of 0.5 s. Selected frames corresponding to the main elution peak were averaged. Data reduction to absolute units, frame averaging and subtraction were done using FOXTROT (David and Pérez, 2009). Data are in Table S1.

All subsequent data processing, analysis and modeling steps were carried out with PRIMUS and other programs of the ATSAS suite (EMBL Hamburg, Germany).

The experimental SAXS data for all samples were linear in a Guinier plot of the low *q* region, indicating that the proteins did not undergo aggregation. The radius of gyration R_G was derived by the Guinier approximation $I(q) = I(0)\ \exp(-q2R_G^2/3)$ for $qR_G < 1.0$ using the software PRIMUS (Konarev et al., 2003). Interference-free SAXS profiles were estimated by extrapolating the measured scattering curves to infinite dilution. The software GNOM (Svergun, 1992) was used to compute the pair-distance distribution functions, P(r). This approach also features the maximum dimension of the macromolecule, D_{max}. Normalized Kratky plot (ie $(qRg)^2$I(q)/I(0) as a function of qRG) was used to assess the conformational behavior of the polypeptide chain. In this representation, the scattering curve of a globular protein yields a characteristic bell-shape profile (Kratky and Porod, 1949), whereas the scattering curve of a disordered/non globular protein exhibits a plateau at large *q* values. For all constructs, the data measured at different protein concentrations were perfectly superimposable at large q (q>0.15Å), indicating that the buffer subtraction was correct, and therefore that the profiles of the Kratky plot is significant.

- David, G., and Pérez, J. (2009). Combined sampler robot and high-performance liquid chromatography: a fully automated system for biological small-angle X-ray scattering

experiments at the synchrotron SOLEIL SWING beamline. Journal of applied crystallography 42, 892-900.

- Ghosh, P. (2004). Process of protein transport by the type III secretion system. Microbiol Mol Biol Rev 68, 771-795.
- Konarev, P.V., Volkov, V.V., Petoukhov, M.V., and Svergun, D.I. (2006). ATSAS 2.1, a program package for small-angle scattering data analysis. Journal of applied crystallography 39, 277-286.
- Konarev, P.V., Volkov, V.V., Sokolova, A.V., Koch, M.H.J., and Svergun, D.I. (2003). PRIMUS: a Windows PC-based system for small-angle scattering data analysis. Journal of applied crystallography 36, 1277-1282.
- Kratky, O., and Porod, G. (1949). Diffuse small-angle scattering of X-rays in colloid systems. Journal of colloid science 4.
- Svergun, D.I. (1992). Determination of the regularization parameter in indirect-transform methods using perceptual criteria. Journal of applied crystallography 25, 495-503.
