# Supplementary material for: DNA Binding of the Cell Cycle Transcriptional Regulator GcrA Depends on N6-Adenosine Methylation in Caulobacter crescentus and Other Alphaproteobacteria
Source: PLoS Genet. 2013 May 30;9(5):e1003541. doi: 10.1371/journal.pgen.1003541 (PMC3667746; doi:10.1371/journal.pgen.1003541)
Supplement: Protocol S2 — ChIP–Seq and data analysis protocols. (PDF) [file pgen.1003541.s012.pdf]

Protocol S2

ChIP-Seq

Illumina Genome Analyzer IIx or HiSeq 2000 runs of barcoded ChIP-Seq libraries yielded several million reads that were mapped to *C. crescentus* NA1000 (NC_011916) using Blast (data available for download at http://www.iri.cnrs.fr/spip.php?rubrique169&lang=en).

Alignments at 100% identity with the genome spanning the entire length of the read without gaps were selected to build a genome wide coverage profile; only one alignment per reads was considered to avoid artefacts concerning repeated genome segments.

The coverage profile obtained was loaded in Matlab(R) and the mspeaks function was used in order to identify peaks i.e. genome regions with high coverage in the experiment. mspeaks is a function that converts raw peak data to a peak list (centroided data) by first smoothing the signal using undecimated wavelet transform with Daubechies coefficients, assigns peak locations, estimates noise and finally eliminates peaks that do not satisfy specified criteria. We used the following parameters: "minimum height" (coverage) of the peak 600 (this was adjusted to obtain a manageable number of peaks, since our analysis does not require to identify all binding locations); "over segmentation" was set empirically to 300 to avoid the identification of multiple peaks actually corresponding to a single bound region. Mspeaks with these parameters returned 50 peaks with height ranging from about 1600 down to a minimum of 600 (as specified by the minimum height parameter); width of the peaks identified by the algorithm ranged from 400 to 1700 nucleotides.

The identification of GcrA controlled promoters was accomplished by extracting from the genome-wide coverage profile the regions from 300 nucleotide upstream to 100 nucleotide downstream the first codon of each gene. The *total coverage* of a gene was defined as the sum of all sequenced nucleotides in that region; after checking for normality, we transformed the total coverage into a Z-score and we retained only genes with Z-score>2 (where $p(Z>2)\approx 0.02$), ending with a list of 161 genes.

Comparison of ChIP-seq data

To compare different ChIP-Seq experiments, we normalized the coverage at each nucleotide by the total coverage of the genome. Then the log_2 ratio among different ChiP-seq dataset was calculated (i.e. wild type vs mutant) and Z-score transformed to highlight differences in binding in different conditions (i.e. presence or absence of methylation).
