# Supplementary material for: DNA Binding of the Cell Cycle Transcriptional Regulator GcrA Depends on N6-Adenosine Methylation in Caulobacter crescentus and Other Alphaproteobacteria
Source: PLoS Genet. 2013 May 30;9(5):e1003541. doi: 10.1371/journal.pgen.1003541 (PMC3667746; doi:10.1371/journal.pgen.1003541)
Supplement: Table S1 — SAXS data. (PDF) [file pgen.1003541.s013.pdf]

TABLE S1

**Q**	**log I**	**I**
0.008	-1.268	0.053970
0.009	-1.268	0.053943
0.009	-1.274	0.053256
0.010	-1.275	0.053110
0.010	-1.279	0.052603
0.011	-1.281	0.052340
0.011	-1.283	0.052143
0.012	-1.289	0.051447
0.012	-1.291	0.051115
0.013	-1.296	0.050559
0.013	-1.298	0.050373
0.014	-1.300	0.050151
0.014	-1.305	0.049600
0.015	-1.309	0.049096
0.015	-1.313	0.048646
0.016	-1.318	0.048070
0.016	-1.323	0.047582
0.017	-1.328	0.047031
0.017	-1.331	0.046718
0.018	-1.335	0.046284
0.018	-1.342	0.045512
0.019	-1.346	0.045085
0.019	-1.351	0.044562
0.020	-1.357	0.043923
0.020	-1.363	0.043344
0.021	-1.368	0.042870
0.021	-1.373	0.042392
0.022	-1.379	0.041764
0.022	-1.384	0.041279
0.023	-1.391	0.040678
0.023	-1.395	0.040296
0.024	-1.401	0.039690
0.024	-1.406	0.039290
0.025	-1.411	0.038798
0.025	-1.418	0.038155
0.026	-1.425	0.037588
0.026	-1.432	0.036977
0.027	-1.439	0.036427
0.027	-1.447	0.035700
0.028	-1.453	0.035238
0.028	-1.459	0.034760
0.029	-1.466	0.034221
0.029	-1.472	0.033741
0.030	-1.477	0.033319
0.030	-1.483	0.032879
0.031	-1.490	0.032339
0.031	-1.496	0.031944
0.032	-1.503	0.031439
0.032	-1.510	0.030902
0.033	-1.516	0.030446

0.033	-1.523	0.030014
0.034	-1.529	0.029608
0.034	-1.532	0.029345
0.035	-1.541	0.028780
0.035	-1.547	0.028411
0.036	-1.554	0.027953
0.036	-1.560	0.027570
0.037	-1.567	0.027115
0.037	-1.572	0.026811
0.038	-1.579	0.026376
0.038	-1.585	0.025994
0.039	-1.591	0.025637
0.039	-1.598	0.025221
0.040	-1.604	0.024905
0.040	-1.613	0.024404
0.041	-1.618	0.024098
0.041	-1.625	0.023695
0.042	-1.630	0.023429
0.042	-1.635	0.023159
0.043	-1.640	0.022921
0.043	-1.645	0.022642
0.044	-1.651	0.022324
0.044	-1.659	0.021934
0.045	-1.666	0.021556
0.045	-1.671	0.021329
0.046	-1.676	0.021108
0.046	-1.683	0.020761
0.047	-1.691	0.020389
0.047	-1.696	0.020147
0.048	-1.700	0.019971
0.048	-1.705	0.019734
0.049	-1.711	0.019438
0.049	-1.719	0.019087
0.050	-1.725	0.018818
0.050	-1.731	0.018569
0.051	-1.737	0.018309
0.051	-1.743	0.018090
0.052	-1.746	0.017927
0.052	-1.755	0.017597
0.053	-1.761	0.017328
0.053	-1.767	0.017117
0.054	-1.772	0.016889
0.054	-1.776	0.016751
0.055	-1.778	0.016656
0.055	-1.785	0.016406
0.056	-1.790	0.016205
0.056	-1.797	0.015962
0.057	-1.801	0.015800
0.057	-1.807	0.015612
0.058	-1.812	0.015405
0.058	-1.818	0.015218
0.059	-1.825	0.014977

0.059	-1.831	0.014766
0.060	-1.838	0.014522
0.060	-1.840	0.014466
0.061	-1.844	0.014336
0.061	-1.847	0.014207
0.062	-1.854	0.013989
0.062	-1.861	0.013766
0.063	-1.865	0.013651
0.064	-1.868	0.013554
0.064	-1.872	0.013442
0.065	-1.876	0.013308
0.065	-1.881	0.013150
0.066	-1.883	0.013081
0.066	-1.886	0.012990
0.067	-1.892	0.012817
0.067	-1.900	0.012581
0.068	-1.902	0.012534
0.068	-1.906	0.012427
0.069	-1.916	0.012139
0.069	-1.923	0.011954
0.070	-1.925	0.011878
0.070	-1.929	0.011769
0.071	-1.935	0.011622
0.071	-1.941	0.011464
0.072	-1.943	0.011400
0.072	-1.946	0.011312
0.073	-1.951	0.011200
0.073	-1.956	0.011070
0.074	-1.960	0.010957
0.074	-1.965	0.010851
0.075	-1.968	0.010763
0.075	-1.970	0.010713
0.076	-1.976	0.010557
0.076	-1.982	0.010423
0.077	-1.989	0.010249
0.077	-1.993	0.010171
0.078	-1.999	0.010030
0.078	-2.003	0.009930
0.079	-2.007	0.009835
0.079	-2.013	0.009696
0.080	-2.017	0.009614
0.080	-2.019	0.009578
0.081	-2.026	0.009428
0.081	-2.031	0.009316
0.082	-2.031	0.009321
0.082	-2.036	0.009213
0.083	-2.036	0.009210
0.083	-2.041	0.009093
0.084	-2.048	0.008948
0.084	-2.057	0.008763
0.085	-2.062	0.008672
0.085	-2.064	0.008636

0.086	-2.064	0.008625
0.086	-2.069	0.008533
0.087	-2.072	0.008474
0.087	-2.076	0.008400
0.088	-2.078	0.008354
0.088	-2.083	0.008255
0.089	-2.094	0.008047
0.089	-2.096	0.008017
0.090	-2.101	0.007930
0.090	-2.106	0.007833
0.091	-2.108	0.007800
0.091	-2.113	0.007716
0.092	-2.120	0.007591
0.092	-2.125	0.007501
0.093	-2.129	0.007430
0.093	-2.133	0.007357
0.094	-2.137	0.007301
0.094	-2.135	0.007323
0.095	-2.136	0.007315
0.095	-2.139	0.007264
0.096	-2.144	0.007179
0.096	-2.151	0.007060
0.097	-2.155	0.006996
0.097	-2.155	0.006997
0.098	-2.164	0.006848
0.098	-2.170	0.006761
0.099	-2.174	0.006691
0.099	-2.173	0.006709
0.100	-2.176	0.006665
0.100	-2.185	0.006536
0.101	-2.189	0.006471
0.101	-2.187	0.006509
0.102	-2.192	0.006432
0.102	-2.197	0.006354
0.103	-2.201	0.006291
0.103	-2.208	0.006195
0.104	-2.207	0.006209
0.104	-2.206	0.006218
0.105	-2.211	0.006154
0.105	-2.219	0.006039
0.106	-2.223	0.005982
0.106	-2.226	0.005947
0.107	-2.231	0.005878
0.107	-2.234	0.005834
0.108	-2.236	0.005803
0.108	-2.241	0.005737
0.109	-2.245	0.005691
0.109	-2.249	0.005642
0.110	-2.252	0.005600
0.110	-2.256	0.005551
0.111	-2.262	0.005470
0.111	-2.262	0.005474

0.112	-2.264	0.005450
0.112	-2.269	0.005381
0.113	-2.275	0.005314
0.113	-2.284	0.005204
0.114	-2.286	0.005172
0.114	-2.293	0.005099
0.115	-2.291	0.005119
0.115	-2.288	0.005152
0.116	-2.290	0.005125
0.116	-2.295	0.005065
0.117	-2.300	0.005016
0.117	-2.303	0.004978
0.118	-2.306	0.004945
0.118	-2.310	0.004892
0.119	-2.314	0.004850
0.119	-2.316	0.004833
0.120	-2.314	0.004853
0.120	-2.320	0.004789
0.121	-2.326	0.004719
0.121	-2.332	0.004658
0.122	-2.333	0.004648
0.122	-2.335	0.004621
0.123	-2.340	0.004573
0.123	-2.342	0.004545
0.124	-2.351	0.004458
0.124	-2.353	0.004438
0.125	-2.351	0.004457
0.125	-2.354	0.004426
0.126	-2.359	0.004372
0.126	-2.362	0.004348
0.127	-2.358	0.004381
0.127	-2.364	0.004320
0.128	-2.370	0.004264
0.128	-2.369	0.004273
0.129	-2.370	0.004264
0.130	-2.377	0.004196
0.130	-2.383	0.004135
0.131	-2.388	0.004096
0.131	-2.391	0.004065
0.132	-2.396	0.004022
0.132	-2.397	0.004009
0.133	-2.396	0.004014
0.133	-2.406	0.003926
0.134	-2.407	0.003913
0.134	-2.408	0.003904
0.135	-2.407	0.003917
0.135	-2.410	0.003894
0.136	-2.417	0.003828
0.136	-2.420	0.003805
0.137	-2.424	0.003767
0.137	-2.424	0.003764
0.138	-2.429	0.003722

0.138	-2.434	0.003680
0.139	-2.437	0.003655
0.139	-2.433	0.003689
0.140	-2.438	0.003651
0.140	-2.446	0.003578
0.141	-2.448	0.003566
0.141	-2.451	0.003537
0.142	-2.458	0.003484
0.142	-2.458	0.003481
0.143	-2.466	0.003423
0.143	-2.466	0.003421
0.144	-2.468	0.003405
0.144	-2.465	0.003431
0.145	-2.475	0.003350
0.145	-2.481	0.003307
0.146	-2.485	0.003272
0.146	-2.485	0.003272
0.147	-2.483	0.003285
0.147	-2.491	0.003226
0.148	-2.496	0.003189
0.148	-2.491	0.003227
0.149	-2.489	0.003246
0.149	-2.491	0.003231
0.150	-2.495	0.003198
0.150	-2.502	0.003146
0.151	-2.508	0.003101
0.151	-2.510	0.003093
0.152	-2.511	0.003080
0.152	-2.514	0.003064
0.153	-2.516	0.003050
0.153	-2.521	0.003012
0.154	-2.522	0.003006
0.154	-2.528	0.002962
0.155	-2.540	0.002883
0.155	-2.539	0.002892
0.156	-2.545	0.002850
0.156	-2.547	0.002840
0.157	-2.545	0.002849
0.157	-2.543	0.002867
0.158	-2.549	0.002827
0.158	-2.546	0.002844
0.159	-2.554	0.002795
0.159	-2.561	0.002747
0.160	-2.554	0.002794
0.160	-2.552	0.002806
0.161	-2.559	0.002764
0.161	-2.560	0.002754
0.162	-2.562	0.002740
0.162	-2.566	0.002719
0.163	-2.571	0.002683
0.163	-2.576	0.002653
0.164	-2.585	0.002603

0.164	-2.590	0.002571
0.165	-2.599	0.002520
0.165	-2.603	0.002493
0.166	-2.601	0.002508
0.166	-2.597	0.002529
0.167	-2.594	0.002550
0.167	-2.604	0.002486
0.168	-2.605	0.002481
0.168	-2.600	0.002512
0.169	-2.613	0.002440
0.169	-2.614	0.002435
0.170	-2.618	0.002407
0.170	-2.624	0.002374
0.171	-2.617	0.002415
0.171	-2.624	0.002376
0.172	-2.630	0.002346
0.172	-2.630	0.002345
0.173	-2.630	0.002342
0.173	-2.636	0.002314
0.174	-2.644	0.002272
0.174	-2.644	0.002272
0.175	-2.644	0.002269
0.175	-2.644	0.002272
0.176	-2.648	0.002251
0.176	-2.647	0.002256
0.177	-2.651	0.002232
0.177	-2.661	0.002183
0.178	-2.655	0.002214
0.178	-2.648	0.002247
0.179	-2.668	0.002150
0.179	-2.678	0.002099
0.180	-2.673	0.002122
0.180	-2.673	0.002125
0.181	-2.674	0.002119
0.181	-2.684	0.002068
0.182	-2.686	0.002059
0.182	-2.683	0.002073
0.183	-2.686	0.002060
0.183	-2.694	0.002023
0.184	-2.700	0.001994
0.184	-2.696	0.002016
0.185	-2.689	0.002046
0.185	-2.690	0.002042
0.186	-2.702	0.001984
0.186	-2.705	0.001972
0.187	-2.703	0.001981
0.187	-2.695	0.002018
0.188	-2.705	0.001974
0.188	-2.714	0.001930
0.189	-2.714	0.001931
0.189	-2.718	0.001916
0.190	-2.720	0.001904

0.190	-2.734	0.001846
0.191	-2.734	0.001846
0.191	-2.725	0.001883
0.192	-2.737	0.001830
0.192	-2.748	0.001788
0.193	-2.735	0.001842
0.193	-2.737	0.001832
0.194	-2.744	0.001803
0.194	-2.756	0.001755
0.195	-2.756	0.001754
0.195	-2.762	0.001731
0.196	-2.756	0.001753
0.196	-2.753	0.001766
0.197	-2.763	0.001727
0.197	-2.768	0.001707
0.198	-2.761	0.001734
0.198	-2.759	0.001741
0.199	-2.754	0.001762
0.200	-2.755	0.001760
0.200	-2.755	0.001759
0.201	-2.760	0.001739
0.201	-2.766	0.001715
0.202	-2.772	0.001689
0.202	-2.787	0.001631
0.203	-2.785	0.001641
0.203	-2.783	0.001650
0.204	-2.789	0.001627
0.204	-2.782	0.001652
0.205	-2.787	0.001634
0.205	-2.809	0.001552
0.206	-2.816	0.001528
0.206	-2.816	0.001529
0.207	-2.813	0.001538
0.207	-2.802	0.001578
0.208	-2.802	0.001577
0.208	-2.797	0.001595
0.209	-2.808	0.001556
0.209	-2.818	0.001520
0.210	-2.811	0.001544
0.210	-2.816	0.001528
0.211	-2.828	0.001488
0.211	-2.831	0.001476
0.212	-2.822	0.001508
0.212	-2.827	0.001490
0.213	-2.830	0.001480
0.213	-2.824	0.001500
0.214	-2.836	0.001460
0.214	-2.839	0.001450
0.215	-2.849	0.001416
0.215	-2.865	0.001365
0.216	-2.855	0.001396
0.216	-2.850	0.001414

0.217	-2.846	0.001426
0.217	-2.846	0.001427
0.218	-2.850	0.001413
0.218	-2.857	0.001390
0.219	-2.856	0.001393
0.219	-2.866	0.001363
0.220	-2.866	0.001361
0.220	-2.866	0.001361
0.221	-2.882	0.001312
0.221	-2.889	0.001291
0.222	-2.883	0.001309
0.222	-2.882	0.001311
0.223	-2.883	0.001308
0.223	-2.886	0.001299
0.224	-2.883	0.001310
0.224	-2.885	0.001304
0.225	-2.879	0.001322
0.225	-2.881	0.001315
0.226	-2.914	0.001220
0.226	-2.916	0.001212
0.227	-2.912	0.001224
0.227	-2.903	0.001251
0.228	-2.910	0.001229
0.228	-2.913	0.001221
0.229	-2.898	0.001264
0.229	-2.901	0.001257
0.230	-2.908	0.001235
0.230	-2.917	0.001210
0.231	-2.901	0.001256
0.231	-2.901	0.001257
0.232	-2.936	0.001159
0.232	-2.957	0.001105
0.233	-2.950	0.001123
0.233	-2.937	0.001157
0.234	-2.943	0.001141
0.234	-2.966	0.001082
0.235	-2.977	0.001055
0.235	-2.954	0.001111
0.236	-2.935	0.001161
0.236	-2.939	0.001150
0.237	-2.932	0.001170
0.237	-2.935	0.001161
0.238	-2.935	0.001160
0.238	-2.944	0.001138
0.239	-2.944	0.001138
0.239	-2.947	0.001129
0.240	-2.950	0.001121
0.240	-2.955	0.001109
0.241	-2.958	0.001100
0.241	-2.964	0.001086
0.242	-2.983	0.001040
0.242	-2.973	0.001065

0.243	-2.981	0.001046
0.243	-2.976	0.001057
0.244	-2.977	0.001053
0.244	-2.988	0.001028
0.245	-2.995	0.001012
0.245	-3.003	0.000994
0.246	-2.990	0.001024
0.246	-2.970	0.001072
0.247	-2.962	0.001091
0.247	-2.970	0.001071
0.248	-2.987	0.001029
0.248	-3.008	0.000981
0.249	-3.001	0.000998
0.249	-2.986	0.001032
0.250	-3.006	0.000986
0.250	-3.020	0.000954
0.251	-3.014	0.000968
0.251	-3.007	0.000984
0.252	-3.019	0.000958
0.252	-3.014	0.000967
0.253	-3.021	0.000952
0.253	-3.017	0.000962
0.254	-3.028	0.000937
0.254	-3.035	0.000924
0.255	-3.046	0.000900
0.255	-3.058	0.000875
0.256	-3.039	0.000915
0.256	-3.018	0.000959
0.257	-3.004	0.000991
0.257	-3.018	0.000959
0.258	-3.016	0.000963
0.258	-3.031	0.000932
0.259	-3.039	0.000914
0.259	-3.037	0.000918
0.260	-3.016	0.000964
0.260	-3.032	0.000930
0.261	-3.035	0.000923
0.261	-3.047	0.000898
0.262	-3.072	0.000848
0.262	-3.070	0.000852
0.263	-3.060	0.000871
0.263	-3.041	0.000909
0.264	-3.051	0.000890
0.264	-3.058	0.000874
0.265	-3.063	0.000866
0.265	-3.066	0.000859
0.266	-3.056	0.000879
0.266	-3.056	0.000879
0.267	-3.074	0.000844
0.267	-3.063	0.000866
0.268	-3.083	0.000827
0.268	-3.086	0.000821

0.269	-3.071	0.000849
0.269	-3.052	0.000886
0.270	-3.048	0.000895
0.270	-3.074	0.000844
0.271	-3.095	0.000804
0.271	-3.103	0.000788
0.272	-3.101	0.000792
0.272	-3.095	0.000804
0.273	-3.076	0.000840
0.273	-3.093	0.000808
0.274	-3.100	0.000794
0.274	-3.089	0.000814
0.275	-3.087	0.000818
0.275	-3.094	0.000805
0.276	-3.081	0.000830
0.276	-3.099	0.000796
0.277	-3.099	0.000797
0.277	-3.084	0.000824
0.278	-3.095	0.000804
0.278	-3.109	0.000779
0.279	-3.089	0.000815
0.279	-3.112	0.000773
0.280	-3.155	0.000700
0.281	-3.135	0.000732
0.281	-3.124	0.000751
0.282	-3.110	0.000777
0.282	-3.112	0.000772
0.283	-3.125	0.000749
0.283	-3.104	0.000786
0.284	-3.118	0.000763
0.284	-3.126	0.000748
0.285	-3.114	0.000769
0.285	-3.099	0.000796
0.286	-3.088	0.000816
0.286	-3.091	0.000812
0.287	-3.111	0.000774
0.287	-3.136	0.000732
0.288	-3.146	0.000715
0.288	-3.135	0.000733
0.289	-3.143	0.000720
0.289	-3.138	0.000728
0.290	-3.143	0.000720
0.290	-3.157	0.000697
0.291	-3.166	0.000683
0.291	-3.165	0.000684
0.292	-3.165	0.000683
0.292	-3.137	0.000729
0.293	-3.160	0.000692
0.293	-3.170	0.000676
0.294	-3.179	0.000662
0.294	-3.177	0.000666
0.295	-3.155	0.000700

0.295	-3.148	0.000711
0.296	-3.146	0.000714
0.296	-3.181	0.000660
0.297	-3.179	0.000662
0.297	-3.151	0.000707
0.298	-3.152	0.000704
0.298	-3.180	0.000660
0.299	-3.199	0.000633
0.299	-3.180	0.000660
0.300	-3.207	0.000621
0.300	-3.191	0.000644
0.301	-3.201	0.000629
0.301	-3.199	0.000633
0.302	-3.206	0.000622
0.302	-3.167	0.000681
0.303	-3.166	0.000682
0.303	-3.197	0.000636
0.304	-3.187	0.000649
0.304	-3.181	0.000659
0.305	-3.179	0.000662
0.305	-3.182	0.000658
0.306	-3.210	0.000617
0.306	-3.209	0.000618
0.307	-3.222	0.000600
0.307	-3.193	0.000641
0.308	-3.180	0.000661
0.308	-3.178	0.000663
0.309	-3.200	0.000631
0.309	-3.226	0.000594
0.310	-3.213	0.000612
0.310	-3.187	0.000650
0.311	-3.202	0.000628
0.311	-3.226	0.000595
0.312	-3.214	0.000610
0.312	-3.192	0.000643
0.313	-3.196	0.000637
0.313	-3.198	0.000633
0.314	-3.210	0.000616
0.314	-3.209	0.000618
0.315	-3.195	0.000639
0.315	-3.204	0.000625
0.316	-3.157	0.000696
0.316	-3.077	0.000837
0.317	-3.112	0.000773
0.317	-3.185	0.000653
0.318	-3.234	0.000584
0.318	-3.224	0.000596
0.319	-3.224	0.000598
0.319	-3.254	0.000557
0.320	-3.248	0.000565
0.320	-3.286	0.000517
0.321	-3.261	0.000548

0.321	-3.223	0.000599
0.322	-3.270	0.000537
0.322	-3.282	0.000523
0.323	-3.240	0.000575
0.323	-3.278	0.000528
0.324	-3.293	0.000510
0.324	-3.289	0.000514
0.325	-3.284	0.000520
0.325	-3.308	0.000492
0.326	-3.286	0.000518
0.326	-3.260	0.000549
0.327	-3.266	0.000542
0.327	-3.273	0.000534
0.328	-3.249	0.000563
0.328	-3.266	0.000542
0.329	-3.241	0.000574
0.329	-3.234	0.000583
0.330	-3.267	0.000541
0.330	-3.245	0.000569
0.331	-3.246	0.000567
0.331	-3.275	0.000531
0.332	-3.251	0.000561
0.332	-3.245	0.000569
0.333	-3.287	0.000516
0.333	-3.263	0.000546
0.334	-3.253	0.000558
0.334	-3.205	0.000623
0.335	-3.194	0.000640
0.335	-3.268	0.000539
0.336	-3.288	0.000515
0.336	-3.291	0.000512
0.337	-3.272	0.000534
0.337	-3.288	0.000515
0.338	-3.290	0.000513
0.338	-3.263	0.000546
0.339	-3.252	0.000560
0.339	-3.268	0.000539
0.340	-3.289	0.000514
0.340	-3.332	0.000465
0.341	-3.330	0.000468
0.341	-3.304	0.000496
0.342	-3.297	0.000505
0.342	-3.330	0.000468
0.343	-3.356	0.000440
0.343	-3.304	0.000497
0.344	-3.311	0.000489
0.344	-3.291	0.000512
0.345	-3.325	0.000473
0.345	-3.268	0.000540
0.346	-3.290	0.000513
0.346	-3.307	0.000493
0.347	-3.320	0.000479

0.347	-3.378	0.000418
0.348	-3.357	0.000440
0.348	-3.348	0.000448
0.349	-3.388	0.000409
0.349	-3.302	0.000499
0.350	-3.279	0.000526
0.350	-3.285	0.000519
0.351	-3.261	0.000549
0.351	-3.270	0.000537
0.352	-3.285	0.000519
0.352	-3.285	0.000519
0.353	-3.259	0.000551
0.353	-3.297	0.000505
0.354	-3.358	0.000438
0.354	-3.362	0.000434
0.355	-3.397	0.000401
0.355	-3.386	0.000411
0.356	-3.370	0.000427
0.356	-3.363	0.000433
0.357	-3.302	0.000498
0.357	-3.280	0.000525
0.358	-3.333	0.000464
0.358	-3.363	0.000433
0.359	-3.408	0.000391
0.359	-3.431	0.000371
0.360	-3.421	0.000379
0.360	-3.371	0.000426
0.361	-3.332	0.000465
0.361	-3.342	0.000455
0.362	-3.335	0.000462
0.362	-3.334	0.000463
0.363	-3.363	0.000433
0.363	-3.398	0.000400
0.364	-3.383	0.000414
0.364	-3.364	0.000432
0.365	-3.343	0.000454
0.365	-3.345	0.000452
0.366	-3.393	0.000404
0.366	-3.349	0.000447
0.367	-3.374	0.000422
0.367	-3.354	0.000443
0.368	-3.360	0.000436
0.368	-3.387	0.000410
0.369	-3.350	0.000447
0.369	-3.363	0.000434
0.370	-3.343	0.000454
0.370	-3.357	0.000439
0.371	-3.393	0.000404
0.371	-3.329	0.000469
0.372	-3.291	0.000512
0.372	-3.339	0.000458
0.373	-3.333	0.000464

0.373	-3.310	0.000489
0.374	-3.314	0.000486
0.374	-3.335	0.000463
0.375	-3.336	0.000462
0.375	-3.388	0.000409
0.376	-3.355	0.000441
0.376	-3.374	0.000423
0.377	-3.350	0.000446
0.377	-3.333	0.000465
0.378	-3.360	0.000437
0.378	-3.367	0.000429
0.379	-3.386	0.000411
0.379	-3.338	0.000459
0.380	-3.308	0.000492
0.380	-3.295	0.000507
0.381	-3.350	0.000447
0.381	-3.390	0.000407
0.382	-3.357	0.000440
0.382	-3.342	0.000455
0.383	-3.338	0.000460
0.383	-3.329	0.000469
0.384	-3.358	0.000439
0.384	-3.394	0.000404
0.385	-3.446	0.000358
0.385	-3.423	0.000378
0.386	-3.406	0.000392
0.386	-3.390	0.000407
0.387	-3.387	0.000410
0.387	-3.421	0.000380
0.388	-3.459	0.000348
0.388	-3.452	0.000353
0.389	-3.438	0.000365
0.389	-3.437	0.000366
0.390	-3.462	0.000345
0.390	-3.410	0.000389
0.391	-3.373	0.000424
0.392	-3.403	0.000395
0.392	-3.379	0.000417
0.393	-3.439	0.000364
0.393	-3.464	0.000344
0.394	-3.452	0.000353
0.394	-3.474	0.000336
0.395	-3.445	0.000359
0.395	-3.410	0.000389
0.396	-3.418	0.000382
0.396	-3.406	0.000392
0.397	-3.332	0.000466
0.397	-3.303	0.000497
0.398	-3.343	0.000454
0.398	-3.425	0.000376
0.399	-3.422	0.000379
0.399	-3.394	0.000404

0.400	-3.452	0.000354
0.400	-3.528	0.000296
0.401	-3.467	0.000342
0.401	-3.456	0.000350
0.402	-3.436	0.000367
0.402	-3.402	0.000397
0.403	-3.391	0.000407
0.403	-3.388	0.000409
0.404	-3.383	0.000414
0.404	-3.367	0.000429
0.405	-3.367	0.000429
0.405	-3.382	0.000415
0.406	-3.429	0.000372
0.406	-3.430	0.000371
0.407	-3.443	0.000360
0.407	-3.381	0.000416
0.408	-3.331	0.000466
0.408	-3.366	0.000431
0.409	-3.437	0.000366
0.409	-3.433	0.000369
0.410	-3.424	0.000377
0.410	-3.425	0.000376
0.411	-3.459	0.000347
0.411	-3.475	0.000335
0.412	-3.478	0.000333
0.412	-3.406	0.000393
0.413	-3.435	0.000368
0.413	-3.490	0.000324
0.414	-3.459	0.000348
0.414	-3.475	0.000335
0.415	-3.456	0.000350
0.415	-3.451	0.000354
0.416	-3.532	0.000293
0.416	-3.542	0.000287
0.417	-3.379	0.000418
0.417	-3.381	0.000416
0.418	-3.415	0.000385
0.418	-3.429	0.000372
0.419	-3.385	0.000412
0.419	-3.395	0.000402
0.420	-3.389	0.000408
0.420	-3.459	0.000348
0.421	-3.460	0.000347
0.421	-3.459	0.000348
0.422	-3.465	0.000343
0.422	-3.444	0.000360
0.423	-3.454	0.000351
0.423	-3.476	0.000334
0.424	-3.586	0.000260
0.424	-3.532	0.000294
0.425	-3.469	0.000340
0.425	-3.461	0.000346

0.426	-3.467	0.000341
0.426	-3.448	0.000356
0.427	-3.401	0.000397
0.427	-3.391	0.000406
0.428	-3.345	0.000452
0.428	-3.369	0.000428
0.429	-3.445	0.000359
0.429	-3.403	0.000396
0.430	-3.417	0.000383
0.430	-3.426	0.000375
0.431	-3.445	0.000359
0.431	-3.408	0.000391
0.432	-3.391	0.000406
0.432	-3.348	0.000448
0.433	-3.369	0.000428
0.433	-3.405	0.000394
0.434	-3.411	0.000388
0.434	-3.447	0.000357
0.435	-3.451	0.000354
0.435	-3.418	0.000382
0.436	-3.429	0.000373
0.436	-3.331	0.000467
0.437	-3.408	0.000391
0.437	-3.430	0.000372
0.438	-3.402	0.000396
0.438	-3.348	0.000449
0.439	-3.328	0.000470
0.439	-3.387	0.000410
0.440	-3.395	0.000403
0.440	-3.398	0.000400
0.441	-3.466	0.000342
0.441	-3.474	0.000336
0.442	-3.469	0.000339
0.442	-3.430	0.000372
0.443	-3.379	0.000418
0.443	-3.326	0.000472
0.444	-3.339	0.000458
0.444	-3.372	0.000425
0.445	-3.391	0.000406
0.445	-3.428	0.000373
0.446	-3.442	0.000362
0.446	-3.524	0.000299
0.447	-3.460	0.000347
0.447	-3.447	0.000358
0.448	-3.484	0.000328
0.448	-3.565	0.000272
0.449	-3.494	0.000320
0.449	-3.457	0.000350
0.450	-3.492	0.000322
0.450	-3.470	0.000339
0.451	-3.373	0.000424
0.451	-3.399	0.000399

0.452	-3.480	0.000331
0.452	-3.416	0.000384
0.453	-3.431	0.000371
0.453	-3.475	0.000335
0.454	-3.426	0.000375
0.454	-3.485	0.000327
0.455	-3.465	0.000343
0.455	-3.417	0.000383
0.456	-3.391	0.000407
0.456	-3.435	0.000367
0.457	-3.474	0.000336
0.457	-3.510	0.000309
0.458	-3.448	0.000357
0.458	-3.407	0.000392
0.459	-3.451	0.000354
0.459	-3.607	0.000247
0.460	-3.633	0.000233
0.460	-3.523	0.000300
0.461	-3.491	0.000322
0.461	-3.463	0.000344
0.462	-3.436	0.000367
0.462	-3.435	0.000367
0.463	-3.574	0.000267
0.463	-3.533	0.000293
0.464	-3.439	0.000364
0.464	-3.378	0.000419
0.465	-3.444	0.000360
0.465	-3.467	0.000341
0.466	-3.475	0.000335
0.466	-3.507	0.000311
0.467	-3.465	0.000343
0.467	-3.415	0.000385
0.468	-3.399	0.000399
0.468	-3.445	0.000359
0.469	-3.430	0.000372
0.469	-3.384	0.000413
0.470	-3.455	0.000351
0.470	-3.500	0.000316
0.471	-3.466	0.000342
0.471	-3.399	0.000399
0.472	-3.441	0.000362
0.472	-3.449	0.000356
0.473	-3.522	0.000301
0.473	-3.542	0.000287
0.474	-3.594	0.000254
0.474	-3.587	0.000259
0.475	-3.581	0.000263
0.475	-3.535	0.000292
0.476	-3.555	0.000279
0.476	-3.491	0.000323
0.477	-3.526	0.000298
0.477	-3.551	0.000281

0.478	-3.579	0.000264
0.478	-3.652	0.000223
0.479	-3.550	0.000282
0.479	-3.499	0.000317
0.480	-3.437	0.000366
0.480	-3.502	0.000315
0.481	-3.475	0.000335
0.481	-3.410	0.000389
0.482	-3.410	0.000389
0.482	-3.432	0.000370
0.483	-3.505	0.000313
0.483	-3.558	0.000277
0.484	-3.504	0.000313
0.484	-3.448	0.000357
0.485	-3.448	0.000356
0.485	-3.555	0.000278
0.486	-3.484	0.000328
0.486	-3.556	0.000278
0.487	-3.574	0.000267
0.487	-3.681	0.000208
0.488	-3.604	0.000249
0.488	-3.465	0.000343
0.489	-3.457	0.000349
0.489	-3.461	0.000346
0.490	-3.505	0.000312
0.490	-3.620	0.000240
0.491	-3.572	0.000268
0.491	-3.632	0.000233
0.492	-3.640	0.000229
0.492	-3.498	0.000318
0.493	-3.470	0.000339
0.493	-3.471	0.000338
0.494	-3.436	0.000366
0.494	-3.558	0.000276
0.495	-3.629	0.000235
0.495	-3.556	0.000278
0.496	-3.617	0.000242
0.496	-3.556	0.000278
0.497	-3.401	0.000397
0.497	-3.402	0.000396
0.498	-3.515	0.000306
0.498	-3.459	0.000348
0.499	-3.421	0.000379
0.499	-3.491	0.000323
0.500	-3.661	0.000218
0.500	-3.550	0.000282
0.501	-3.436	0.000366
0.501	-3.530	0.000295
0.502	-3.673	0.000212
0.502	-3.780	0.000166
0.503	-3.751	0.000177
0.503	-3.550	0.000282

0.504	-3.590	0.000257
0.504	-3.717	0.000192
0.505	-3.835	0.000146
0.505	-3.655	0.000221
0.506	-3.550	0.000282
0.506	-3.405	0.000394
0.507	-3.391	0.000406
0.507	-3.616	0.000242
0.508	-3.514	0.000307
0.508	-3.505	0.000312
0.509	-3.607	0.000247
0.509	-3.463	0.000344
0.510	-3.421	0.000379
0.510	-3.646	0.000226
0.511	-3.710	0.000195
0.511	-3.718	0.000191
0.512	-3.558	0.000277
0.512	-3.481	0.000330
0.513	-3.529	0.000296
0.513	-3.539	0.000289
0.514	-3.742	0.000181
0.514	-3.458	0.000349
0.515	-3.347	0.000450
0.515	-3.404	0.000394
0.516	-3.638	0.000230
0.516	-3.620	0.000240
0.517	-3.432	0.000370
0.517	-3.436	0.000367
0.518	-3.462	0.000345
0.518	-3.508	0.000310
0.519	-3.888	0.000129
0.519	-3.737	0.000183
0.520	-3.687	0.000206
0.520	-4.084	0.000082
0.521	-3.583	0.000261
0.521	-3.484	0.000328
0.522	-3.788	0.000163
0.522	-3.758	0.000175
0.523	-3.893	0.000128
0.523	-4.626	0.000024
0.524	-3.599	0.000252
0.524	-3.966	0.000108
0.525	-3.844	0.000143
0.525	-3.545	0.000285
0.526	-3.524	0.000299
0.526	-3.647	0.000225
0.527	-3.507	0.000311
0.527	-3.477	0.000333
0.528	-3.398	0.000400
0.528	-3.238	0.000578
0.529	-3.314	0.000485
0.529	-3.436	0.000367

0.530	-3.460	0.000347
0.530	-3.362	0.000435
0.531	-3.225	0.000596
0.531	-3.222	0.000599
0.532	-3.431	0.000371
0.532	-3.678	0.000210
0.533	-3.711	0.000195
0.533	-3.376	0.000420
0.534	-3.534	0.000293
0.534	-3.819	0.000152
0.536	-3.370	0.000427
0.537	-3.502	0.000315
0.537	-3.614	0.000243
0.538	-3.452	0.000353
0.538	-3.755	0.000176
0.539	-3.206	0.000622
0.539	-3.094	0.000806
