# Supplementary material for: DNA Binding of the Cell Cycle Transcriptional Regulator GcrA Depends on N6-Adenosine Methylation in Caulobacter crescentus and Other Alphaproteobacteria
Source: PLoS Genet. 2013 May 30;9(5):e1003541. doi: 10.1371/journal.pgen.1003541 (PMC3667746; doi:10.1371/journal.pgen.1003541)
Supplement: Table S2 — Best peaks (1 kbp long) derived from GcrA in wild type ChIP–Seq. (PDF) [file pgen.1003541.s014.pdf]

Peak maximum (nt position in NA1000)	Height (number of reads)	Closest gene start	Strand	GANTC methylation sites
757214	1607.84908	CCNA_00697	0	4
2766545	1340.684383	CCNA_02617	0	1
3240749	1274.696054	CCNA_03092	1	7
853657	1249.510308	CCNA_00790	1	1
1574607	1237.130047	CCNA_01467	1	2
3676529	1229.489792	CCNA_03518	0	2
3673685	1156.479156	CCNA_03516	0	2
2279319	1144.701866	CCNA_02126	1	5
721839	1143.975009	CCNA_00665	1	2
292314	1108.752766	CCNA_00279	1	0
1812369	1073.67743	CCNA_01684	0	2
3854853	1045.903403	CCNA_03689	1	2
3376200	979.247797	CCNA_03214	0	2
2349723	977.380465	CCNA_02200	0	2
864798	975.2115345	CCNA_00800	1	0
1632212	913.122217	CCNA_01523	0	1
3215023	904.9247305	CCNA_03062	0	5
2867923	872.4536166	-	0	1
3976528	839.0294446	CCNA_03813	1	4
1917883	836.4550302	CCNA_01791	1	1
136696	834.6053674	CCNA_00126	0	1
2940437	825.5585172	CCNA_02782	0	3
920769	821.0088263	CCNA_00847	1	0
861176	820.0753908	CCNA_00796	0	0
813315	806.7680845	CCNA_00754	0	0
484581	791.754344	-	0	0
1213723	768.4076388	CCNA_01108	0	4
1491807	758.7170666	-	0	0
3240410	741.4531948	CCNA_03091	1	8
568875	741.2500911	CCNA_00551	0	2
2294257	737.9556949	CCNA_02136	0	1
1583469	727.0637471	CCNA_01476	1	0
1272541	716.5360305	CCNA_01163	1	1
929113	714.3068704	CCNA_00854	1	1
3764128	692.5839505	CCNA_03609	0	2
3904026	688.1558129	CCNA_03734	0	0
2727779	677.1672756	CCNA_02576	1	3
3860010	673.8175102	CCNA_03694	1	0
1057152	653.160436	CCNA_00977	1	5
3573948	644.8775698	CCNA_03406	1	2
215237	643.285953	CCNA_00201	1	1
1081928	638.3206506	CCNA_00999	1	7
2152403	623.979405	CCNA_02003	0	3
1639320	622.4050809	CCNA_01532	1	4
2445754	620.2120944	CCNA_02299	1	3
2108149	616.1845584	CCNA_01966	1	1
48961	611.0390459	CCNA_00044	0	3
2186900	610.0562312	CCNA_02041	0	1
1983866	609.3718079	CCNA_01851	0	0
3547252	605.1256119	CCNA_03371	0	2
1755833	603.1284043	CCNA_01637	1	3
1176239	599.5615142	-	0	0
3994429	593.5048097	CCNA_03830	1	3

2960581	587.8380285	CCNA_02805	1	2
2435683	583.0533325	CCNA_02287	1	2
406003	582.6219374	CCNA_00390	1	1
3975756	580.9789969	CCNA_03811	0	1
2396055	566.5476575	CCNA_02246	0	2
634994	562.7829258	CCNA_00598	0	1
278021	559.106736	CCNA_00265	1	0
519938	552.0430734	CCNA_00504	0	1
2773202	548.4620861	CCNA_02623	0	2
3798041	537.7561563	CCNA_03638	0	1
58628	525.5947236	CCNA_00055	0	2
2543464	524.1480847	CCNA_02401	1	1
2868260	517.6871245	CCNA_02708	1	1
3184764	514.1951846	CCNA_03029	0	3
404079	508.2508497	CCNA_00388	0	1
2577623	508.1107197	CCNA_02436	0	4
3702058	500.985044	CCNA_03545	1	0
3618089	500.7682216	-	0	2
2866441	496.1437569	-	0	0
938858	493.8343185	CCNA_00860	0	1
1159110	493.6191007	CCNA_01057	0	0
856077	491.9221678	CCNA_00793	1	3
1628284	489.5975999	CCNA_01519	1	0
304379	488.0371371	CCNA_00291	1	4
3426577	486.3599835	CCNA_03255	0	0
3818458	484.002705	CCNA_03656	0	3
1985446	483.8455731	CCNA_01853	1	4
2589801	483.5754687	CCNA_02449	1	3
20783	483.5668386	CCNA_00020	0	2
2750336	482.3721182	CCNA_02604	0	0
2290387	477.8848845	CCNA_02133	0	0
1159638	472.6763432	CCNA_01059	1	1
290091	472.0939434	CCNA_00276	0	0
2236239	470.5136949	CCNA_02086	0	2
1815944	470.0474954	CCNA_01689	1	3
3720829	467.9571091	CCNA_03567	1	2
2239171	463.424966	CCNA_02091	1	2
2338501	463.2597591	-	0	2
4022960	461.0859555	CCNA_03859	0	2
2178231	457.5930894	-	0	0
281915	457.5861075	CCNA_00267	0	5
899898	456.8870776	-	0	1
998588	454.6077138	CCNA_00922	1	1
3874323	453.9262748	-	0	1
1653488	451.9707307	CCNA_01542	0	3
370406	449.7225859	CCNA_00354	1	0
2560092	447.9432303	CCNA_02416	1	3
2936623	443.3308244	CCNA_02779	1	2
735527	437.4537652	CCNA_00679	1	2
338448	435.1270375	CCNA_00325	0	4
1541385	433.5805365	CCNA_01425	1	2
1895418	430.127904	CCNA_01766	0	1
335837	428.2773758	CCNA_00321	0	5
2663293	428.1947558	CCNA_02512	0	2
477396	421.8383028	CCNA_00464	1	5
2889903	419.7436702	CCNA_02726	0	4
454829	417.1291908	CCNA_00450	1	2
2145117	415.9123835	CCNA_01998	0	1

1634112	413.1833031	CCNA_01524	0	4
3506609	408.8727669	CCNA_03326	1	1
2044475	408.0094772	CCNA_01901	0	3
3750251	406.8668607	CCNA_03598	1	2
4812	405.2941038	CCNA_00007	1	2
3931447	402.652319	CCNA_03762	1	1
3279769	402.2195688	CCNA_03130	0	2
3294542	402.0392072	CCNA_03143	0	1
314531	401.5611581	CCNA_00300	0	2
3594186	398.9976831	CCNA_03430	0	1
3882816	397.8305991	CCNA_03718	1	4
2177490	397.5428607	CCNA_02033	0	2
1431119	396.9402413	CCNA_01305	1	3
511840	395.8003579	CCNA_00495	1	1
2317078	395.4347381	CCNA_02162	0	3
1423062	393.6724819	CCNA_01297	0	2
1534753	393.4176638	CCNA_01417	1	1
2676563	392.3034402	CCNA_02528	1	1
1812872	389.6968652	CCNA_01686	1	6
83965	388.4843013	CCNA_00079	0	0
567065	387.9091641	CCNA_00550	1	1
945883	386.4580559	CCNA_00866	0	0
1211146	383.5970212	CCNA_01105	1	3
1672239	383.0061478	CCNA_01556	1	2
1638717	382.6787835	CCNA_01531	1	1
2974835	382.263418	CCNA_02818	1	2
76216	380.8698369	-	0	0
3487644	377.4401113	CCNA_03312	1	2
30187	376.1729875	CCNA_00027	0	1
1348022	373.303679	CCNA_01221	0	3
4028743	372.5249152	CCNA_03866	1	2
1845852	372.3835529	CCNA_01715	1	3
3159561	371.2253879	CCNA_03006	1	1
2822621	368.0305798	CCNA_02669	0	1
1199270	365.1888206	CCNA_01094	0	0
3451017	364.9913468	CCNA_03280	0	2
1136909	364.9347964	CCNA_01041	0	1
804893	364.2798798	CCNA_00747	0	3
473497	363.255243	-	0	0
3136383	358.2181559	CCNA_02980	0	1
2465200	357.0792323	CCNA_02323	0	0
433212	356.8610447	CCNA_00422	0	0
1301880	356.4749123	CCNA_01181	0	6
3256857	356.0627561	CCNA_03107	1	3
3101495	355.7189436	CCNA_02941	1	0
2832658	354.6562442	CCNA_02679	0	2
1602083	353.263304	CCNA_01492	0	1
2245237	350.0886007	CCNA_02096	0	2
2692183	349.9094452	CCNA_02543	1	3
506644	349.6343646	CCNA_00488	0	0
482835	348.140722	-	0	0
3571156	348.0348256	CCNA_03401	0	0
2391799	345.9536292	CCNA_02243	1	1
1094491	345.524169	-	0	0
582139	345.4345942	-	0	3
2434066	344.984723	CCNA_02283	0	2
2147264	342.5612811	CCNA_02000	0	1
1781924	342.1028442	CCNA_01660	0	2

38748	337.4711167	CCNA_00034	0	0
3906409	337.3970439	CCNA_03736	1	3
3834205	336.4376683	CCNA_03674	0	2
2864978	336.2670888	-	0	0
1901987	335.9798576	CCNA_01776	1	1
2923184	335.6564907	CCNA_02760	1	1
1564911	334.438289	-	0	2
1395561	334.2408186	CCNA_01265	0	2
2158027	333.2432798	CCNA_02011	0	1
3459093	332.7920107	CCNA_03291	0	3
2185233	332.1433833	CCNA_02039	0	1
74002	331.5297844	-	0	2
3565550	330.3684024	CCNA_03394	1	2
1529188	328.7483831	CCNA_01410	0	1
844333	328.2983073	CCNA_00782	0	0
1771266	327.6455251	CCNA_01651	1	2
2383606	327.3070942	CCNA_02235	1	1
1856369	327.2219559	CCNA_01727	0	3
718869	327.0062849	CCNA_00660	0	1
967794	324.5636945	CCNA_00889	0	2
166601	324.3979886	CCNA_00159	1	4
1582559	322.26797	CCNA_01475	1	1
1956579	321.3128824	CCNA_01830	1	1
67929	320.7691983	-	0	0
52174	320.1099657	CCNA_00048	0	4
2103414	317.3811563	CCNA_01957	0	2
2551882	317.205461	CCNA_02408	0	2
1191123	316.2182902	CCNA_01085	0	2
708255	316.1653282	CCNA_00655	0	0
2227213	314.3368512	CCNA_02076	0	1
2709507	313.6640792	-	0	0
2289183	313.6294944	CCNA_02132	0	3
2570916	312.9829764	CCNA_02428	0	3
170864	312.0624163	CCNA_00161	1	1
1266850	311.2933456	CCNA_01159	1	1
4025307	310.92048	CCNA_03864	1	4
670390	310.7975982	CCNA_00624	0	2
2090245	310.5833948	CCNA_01948	1	1
1571958	309.5078316	CCNA_01463	0	2
842265	307.1841786	-	0	1
2097120	306.6811044	CCNA_01952	0	2
66256	306.4190371	CCNA_00063	0	0
1113601	303.2646094	CCNA_01026	0	0
981708	303.1098257	CCNA_00901	0	2
2710573	302.6483671	CCNA_02562	0	3
2863441	302.2357208	-	0	0
1140073	302.1554094	CCNA_01044	1	0
368532	301.8856903	CCNA_00352	0	0
2408835	301.0811232	CCNA_02256	0	5
