# Supplementary material for: DNA Binding of the Cell Cycle Transcriptional Regulator GcrA Depends on N6-Adenosine Methylation in Caulobacter crescentus and Other Alphaproteobacteria
Source: PLoS Genet. 2013 May 30;9(5):e1003541. doi: 10.1371/journal.pgen.1003541 (PMC3667746; doi:10.1371/journal.pgen.1003541)
Supplement: Table S3 — Best promoter regions derived from GcrA in wild type, GcrA in ΔccrM m6A in wild type and m6A in ΔccrM ChIp–Seqs. (PDF) [file pgen.1003541.s015.pdf]

Start..end	Strand	Product length	gene name	Gene code	Z-score coverage ChIP-seq GcrA in wt
4945..5235	+	96	rpsT	CCNA_00007	2.559813191
20444..20668	-	74	-	CCNA_00020	2.376856495
48271..48846	-	191	-	CCNA_00044	3.400383997
49105..49638	+	177	-	CCNA_00045	3.287447357
58201..58608	-	135	-	CCNA_00055	2.319018145
83414..83893	-	159	-	CCNA_00079	2.40745932
84087..84728	+	213	-	CCNA_00080	2.407360282
136184..1365	-	135	-	CCNA_00126	3.818655622
136811..1370	+	66	-	CCNA_00127	3.817830303
214320..2153	-	346	-	CCNA_00200	3.359778307
215418..2160	+	226	-	CCNA_00201	5.000545318
278017..2794	+	484	-	CCNA_00265	2.763931065
280931..2818	-	313	-	CCNA_00267	2.922458321
282085..2839	+	623	-	CCNA_00268	2.856366783
292311..2929	+	196	-	CCNA_00279	7.146539554
304469..3058	+	471	phoR	CCNA_00291	3.149949226
335240..3357	-	171	rplU	CCNA_00321	3.004891174
335959..3365	+	209	-	CCNA_00322	3.005947583
337932..3383	-	152	-	CCNA_00324	2.542811622
338634..3396	+	343	-	CCNA_00326	2.199611005
405104..4059	-	281	-	CCNA_00389	3.095610229
405999..4072	+	404	-	CCNA_00390	2.973925209
454858..4553	+	156	cheE	CCNA_00450	2.252530454
477514..4776	+	54	-	CCNA_00464	3.532567074
477921..4790	+	370	-	CCNA_00465	2.497848247
519729..5201	-	154	-	CCNA_00504	2.801202467
520398..5209	+	183	-	CCNA_00505	2.735936247
567006..5680	+	348	-	CCNA_00550	2.144809829
568260..5687	-	146	-	CCNA_00551	4.818380927
631886..6348	-	976	-	CCNA_00598	3.027570938
721949..7230	+	381	-	CCNA_00665	5.890239105
735544..7364	+	311	-	CCNA_00679	2.404752274
756955..7572	-	113	-	CCNA_00697	8.416903437
812595..8133	-	241	-	CCNA_00754	4.665564877
852873..8535	-	216	fixK	CCNA_00789	9.071546398
853690..8540	+	128	fixT	CCNA_00790	8.373260573
856299..8576	+	455	-	CCNA_00793	2.52363121
859581..8610	-	504	fixL	CCNA_00796	4.97387101
863065..8647	-	546	cydD	CCNA_00799	7.060937476
864829..8663	+	520	cydA	CCNA_00800	7.336858095
917301..9203	-	1029	-	CCNA_00846	2.273724643
920836..9213	+	155	-	CCNA_00847	5.097800898
928249..9290	-	260	-	CCNA_00853	4.266572702
929146..9307	+	530	-	CCNA_00854	4.228641045
938619..9388	-	78	-	CCNA_00860	2.701338878
944663..9458	-	384	xylX	CCNA_00866	2.37045202
946004..9464	+	162	-	CCNA_00867	2.386232118
998598..1001	+	859	clpB	CCNA_00922	2.419839104
1055002..105	-	594	sppA	CCNA_00976	2.50758701
1057369..105	+	136	-	CCNA_00977	4.587555734

1081948..108	+	177	-	CCNA_00999	3.226472796
1157551..115	-	498	-	CCNA_01057	2.988615885
1159130..115	-	99	-	CCNA_01058	2.224007432
1212651..121	-	286	-	CCNA_01108	3.044308406
1213981..121	+	596	-	CCNA_01109	4.175127367
1272589..128	+	2479	-	CCNA_01163	4.904841337
1301278..130	-	174	-	CCNA_01181	2.366061323
1301993..130	+	197	-	CCNA_01182	2.375635023
1347735..134	-	86	-	CCNA_01221	2.598471129
1422154..142	-	278	-	CCNA_01297	2.715072185
1534788..153	+	408	-	CCNA_01417	2.272899324
1574323..157	-	134	-	CCNA_01466	5.724614106
1574712..157	+	548	ccoN	CCNA_01467	9.7651114
1583425..158	+	255	ftrB	CCNA_01476	4.530707767
1627539..162	-	198	-	CCNA_04008	2.421192627
1631535..163	-	196	flmH	CCNA_01523	5.466256274
1632277..163	-	596	flbA	CCNA_01524	2.428125306
1634266..163	+	141	flbT	CCNA_01525	2.43188876
1639377..164	+	954	flaY	CCNA_01532	3.968566549
1651185..165	-	756	-	CCNA_01542	2.305647979
1670626..167	-	502	-	CCNA_01555	2.339618105
1672295..167	+	357	-	CCNA_01556	2.491740887
1755866..175	+	759	-	CCNA_01637	3.140078412
1811466..181	-	294	phhA	CCNA_01684	7.832544634
1812515..181	-	109	-	CCNA_01685	2.264613123
1812889..181	+	407	-	CCNA_01686	3.287051204
1894443..189	-	275	-	CCNA_01766	2.337670353
1917887..191	+	550	pyrG	CCNA_01791	3.420323702
1982720..198	-	353	qoxA	CCNA_01851	4.445633894
1983992..198	+	446	-	CCNA_01852	4.413512482
1985425..198	+	134	-	CCNA_01853	2.497980298
2043376..204	-	329	-	CCNA_01901	2.619533268
2107547..210	-	131	-	CCNA_01964	3.87107988
2107942..210	-	40	-	CCNA_01965	3.688849464
2108222..211	+	907	-	CCNA_01966	3.961138679
2148907..215	-	1143	dnaE	CCNA_02003	3.32065819
2152629..215	+	319	-	CCNA_02005	2.565293309
2177095..217	-	125	nuoA	CCNA_02033	2.85884274
2186211..218	-	213	clpP	CCNA_02041	2.554002946
2235387..223	-	266	-	CCNA_02086	2.438161184
2244856..224	-	122	-	CCNA_02096	2.434628819
2276272..227	-	974	podJ	CCNA_02125	8.123783172
2279405..227	+	71	-	CCNA_02126	8.041779485
2289247..229	-	331	glk	CCNA_02133	2.246588158
2292773..229	-	487	zwf	CCNA_02136	3.39467279
2316896..231	-	73	-	CCNA_02162	2.516104302
2317218..231	+	286	-	CCNA_02163	2.947019812
2349330..234	-	130	-	CCNA_02200	5.552353543
2395180..239	-	278	-	CCNA_02246	2.982937691
2396214..239	+	285	panC	CCNA_02247	2.996274845
2435476..243	-	36	-	CCNA_02286	3.511339872
2435736..243	+	215	-	CCNA_02287	3.459905997
2445821..244	+	235	-	CCNA_02299	3.33729662

2542088..254	-	418	-	CCNA_02400	2.813978404
2543511..254	+	350	-	CCNA_02401	2.700348496
2560136..256	+	92	-	CCNA_02416	2.215622192
2570674..257	-	76	infA	CCNA_02428	2.217404881
2577082..257	-	156	-	CCNA_02436	3.184777685
2589111..258	-	223	-	CCNA_02448	2.412840399
2589811..259	+	257	-	CCNA_02449	2.995845679
2676573..267	+	218	-	CCNA_02528	2.312613671
2727217..272	-	104	-	CCNA_02575	4.125740283
2727918..272	+	252	-	CCNA_02576	4.216030172
2749903..275	-	138	-	CCNA_02604	2.319513337
2765344..276	-	378	-	CCNA_02617	7.904446418
2771579..277	-	508	ftsZ	CCNA_02623	2.701933108
2867620..286	+	132	-	CCNA_02706	2.288646409
2889287..288	-	176	-	CCNA_02726	2.785158267
2936684..293	+	483	-	CCNA_02779	2.367183757
2939969..294	-	161	-	CCNA_02782	6.529201008
2940573..294	+	572	-	CCNA_02783	6.891846138
2959801..296	-	196	-	CCNA_02803	2.239919581
2960591..296	+	137	-	CCNA_02805	2.239919581
3183958..318	-	245	-	CCNA_03029	2.308520089
3184852..318	+	228	-	CCNA_03030	2.338363621
3213796..321	-	370	-	CCNA_03062	6.013640789
3239258..324	-	320	-	CCNA_03090	4.324807205
3240500..324	+	66	-	CCNA_03091	5.331531214
3240801..324	+	458	-	CCNA_03092	8.444766204
3278952..327	-	231	ctrA	CCNA_03130	2.556544928
3375756..337	-	136	-	CCNA_03214	5.109982606
3376165..337	+	471	-	CCNA_03215	4.008511985
3487695..348	+	75	-	CCNA_03312	2.454436473
3506186..350	-	108	-	CCNA_03325	2.748018916
3506595..350	+	657	-	CCNA_03326	2.153591223
3546725..354	-	159	-	CCNA_03371	3.648144735
3573190..357	-	225	-	CCNA_03405	4.046245566
3574019..357	+	87	rpsU	CCNA_03406	4.246831074
3593983..359	-	41	rpmJ	CCNA_03430	2.197267099
3594318..359	+	433	-	CCNA_03431	2.166466198
3672702..367	-	339	coxE	CCNA_03516	5.104799603
3675514..367	-	331	coxB	CCNA_03518	7.520904213
3676818..367	+	108	-	CCNA_03519	4.966245064
3749725..375	-	136	-	CCNA_03597	2.357378968
3750296..375	+	769	-	CCNA_03598	2.258868903
3762727..376	-	449	-	CCNA_03609	4.788009191
3817495..381	-	307	-	CCNA_03656	2.504681888
3854292..385	-	157	-	CCNA_03688	6.735629774
3854885..385	+	370	-	CCNA_03689	6.535143304
3859081..385	-	251	-	CCNA_03693	3.342710712
3860040..386	+	314	-	CCNA_03694	3.384042683
3881992..388	-	205	-	CCNA_03717	2.937479126
3883021..388	+	319	-	CCNA_03718	2.864983112
3901941..390	-	678	nadE	CCNA_03734	3.07418495
3904122..390	+	671	-	CCNA_03735	3.170285084
3906574..390	+	123	-	CCNA_03736	2.257251278

3974785..397	-	300 -	CCNA_03811	3.315409162
3975911..397	+	183 -	CCNA_03812	3.252222746
3976615..397	+	457 creS	CCNA_03813	3.63011977
3994545..399	+	396 -	CCNA_03830	3.678351407
4023026..402	+	169 -	CCNA_03860	2.74686347

Start..end	Strand	Product length	Gene code	Z-score coverage ChIP-seq GcrA in Δ*ccrM*
51..100	+	301	CCNA_00001	4.969991492
3201..3250	+	237	CCNA_00005	4.130647742
4851..4900	+	96	CCNA_00007	13.18856927
5401..5450	+	490	CCNA_00008	5.284745722
7951..8000	-	206	CCNA_00009	2.74922712
8001..8050	+	631	CCNA_00010	2.801685727
11401..11450	+	903	CCNA_00012	2.224636737
19751..19800	-	349	CCNA_00018	2.067260054
20751..20800	-	74	CCNA_00020	2.242123227
23701..23750	-	176	CCNA_00022	3.256331013
23651..23700	+	156	CCNA_00023	3.06398135
29751..29800	-	900	CCNA_00026	2.46944587
32801..32850	-	851	CCNA_00028	3.256331013
32701..32750	+	210	CCNA_00029	3.168899426
38701..38750	-	89	CCNA_00034	12.31425254
39951..40000	-	149	CCNA_00036	3.710975436
41901..41950	-	172	CCNA_00039	2.959063273
46001..46050	-	1037	CCNA_00041	3.221358033
48951..49000	-	191	CCNA_00044	8.20496365
49001..49050	+	177	CCNA_00045	6.246494038
52101..52150	-	407	CCNA_00048	2.801685727
58601..58650	-	135	CCNA_00055	3.62354385
64451..64500	-	344	CCNA_00062	2.032287074
71901..71950	-	367	CCNA_00069	2.679282023
71951..72000	+	322	CCNA_00070	2.224636737
83951..84000	-	159	CCNA_00079	11.43993581
84051..84100	+	213	CCNA_00080	10.74048225
85201..85250	-	69	CCNA_00081	2.556877457
96351..96400	-	1607	CCNA_00086	2.76671361
111601..111650	-	226	CCNA_00100	2.521904477
116051..116100	+	232	CCNA_00105	2.45195938
120051..120100	-	299	CCNA_00109	2.067260054
127801..127850	-	448	CCNA_00116	2.18966462
127851..127900	+	606	CCNA_00117	2.609336064
136651..136700	-	135	CCNA_00126	4.777641829
136701..136750	+	66	CCNA_00127	4.707696732
138451..138500	-	408	CCNA_00128	2.871631686
140951..141000	-	665	CCNA_00130	18.04977032
141001..141050	+	140	CCNA_00131	15.70660138
142601..142650	+	404	CCNA_00133	3.86835212
163251..163300	-	86	CCNA_00154	2.43447289
163301..163350	+	372	CCNA_00155	2.7842001
165451..165500	+	387	CCNA_00158	2.259609717
166601..166650	+	821	CCNA_00159	2.46944587
170751..170800	+	208	CCNA_00161	2.924090293
200151..200200	-	841	CCNA_00185	4.462887599
203751..203800	-	334	CCNA_00188	2.347041304
203801..203850	+	88	CCNA_00189	2.067260054
212151..212200	-	245	CCNA_00198	5.512068365

212051..212100	+	626	CCNA_00199	5.529553992
221501..221550	+	213	CCNA_00207	2.137205151
225001..225050	+	843	CCNA_00210	2.312068324
232551..232600	+	262	CCNA_00215	3.641030339
247951..248000	-	138	CCNA_00232	2.99403539
248001..248050	+	345	CCNA_00233	3.396221207
252351..252400	-	565	CCNA_00236	4.340483895
252401..252450	+	235	CCNA_00237	5.949226298
256951..257000	+	213	CCNA_00244	2.067260054
260901..260950	-	466	CCNA_00248	4.078188273
260951..261000	+	196	CCNA_00249	3.536112263
261601..261650	+	427	CCNA_00250	3.04649486
265801..265850	-	653	CCNA_00252	2.644309043
277751..277800	-	417	CCNA_00264	3.098953467
278001..278050	+	484	CCNA_00265	7.575455191
281951..282000	-	313	CCNA_00267	3.938298079
282001..282050	+	623	CCNA_00268	3.431194186
304351..304400	+	471	CCNA_00291	7.243215334
314501..314550	-	197	CCNA_00300	10.26835134
314651..314700	+	360	CCNA_00301	4.427915482
322901..322950	-	607	CCNA_00307	3.238844523
322951..323000	+	221	CCNA_00308	2.871631686
324101..324150	-	77	CCNA_00309	2.084746544
335801..335850	-	171	CCNA_00321	20.2530482
335851..335900	+	209	CCNA_00322	19.57108113
336701..336750	+	365	CCNA_00323	2.014801447
338401..338450	-	152	CCNA_00324	4.008243176
338451..338500	-	42	CCNA_00325	3.973270196
338601..338650	+	343	CCNA_00326	3.221358033
352351..352400	+	399	CCNA_00340	5.302232212
360251..360300	-	329	CCNA_00345	2.539390967
360201..360250	+	475	CCNA_00346	2.924090293
364251..364300	-	667	CCNA_00348	3.676002457
366001..366050	-	226	CCNA_00350	3.693488946
366051..366100	+	719	CCNA_00351	3.04649486
369251..369300	+	364	CCNA_00353	3.081467839
370401..370450	+	235	CCNA_00354	4.148134232
382701..382750	-	419	CCNA_00365	8.362340334
382951..383000	+	264	CCNA_00366	3.676002457
390051..390100	-	253	CCNA_00373	5.651958559
390251..390300	-	126	CCNA_00374	14.22026268
390601..390650	+	129	CCNA_00375	6.473816681
390701..390750	+	268	CCNA_00376	2.556877457
396801..396850	-	204	CCNA_00379	4.952505002
400751..400800	-	211	CCNA_00383	2.382014283
407801..407850	+	444	CCNA_00392	2.329554814
416801..416850	+	414	CCNA_00399	2.032287074
424351..424400	-	434	CCNA_00408	2.74922712
430301..430350	-	132	CCNA_00416	2.48693236
430551..430600	-	148	CCNA_00417	15.54922383
430501..430550	+	54	CCNA_00418	14.50004393
430651..430700	+	178	CCNA_00419	5.966712788
433151..433200	-	250	CCNA_00422	7.94266889

433251..433300	+	156	CCNA_00423	2.224636737
471451..471500	+	413	CCNA_00459	4.340483895
472851..472900	+	51	CCNA_00460	4.515347069
477401..477450	+	54	CCNA_00464	3.60605736
477601..477650	+	370	CCNA_00465	2.049773564
500301..500350	-	281	CCNA_00483	2.644309043
501051..501100	-	247	CCNA_00484	2.556877457
507751..507800	-	139	CCNA_00489	2.347041304
511851..511900	+	84	CCNA_00495	10.07600168
520201..520250	-	154	CCNA_00504	5.389663798
520051..520100	+	183	CCNA_00505	3.86835212
527201..527250	-	366	CCNA_00512	2.679282023
533801..533850	-	197	CCNA_00518	5.844308222
535001..535050	-	358	CCNA_00519	3.501139283
536201..536250	-	312	CCNA_00520	2.18966462
539651..539700	-	302	CCNA_00525	3.413707696
539701..539750	+	85	CCNA_00526	4.462887599
542301..542350	+	172	CCNA_00530	21.82681849
547451..547500	+	52	CCNA_00535	2.224636737
547551..547600	+	1356	CCNA_00536	4.288024426
562401..562450	-	216	CCNA_00543	2.46944587
562501..562550	+	391	CCNA_00544	2.73174063
564601..564650	+	260	CCNA_00546	3.501139283
575751..575800	-	382	CCNA_00559	2.45195938
575801..575850	+	170	CCNA_00560	2.18966462
581951..582000	-	580	CCNA_00565	5.249772742
628451..628500	+	1046	CCNA_00597	2.871631686
660001..660050	-	972	CCNA_00615	2.137205151
735101..735150	-	143	CCNA_00678	2.119718661
738051..738100	-	245	CCNA_00681	2.17217813
741751..741800	-	205	CCNA_00685	2.277096207
757201..757250	-	113	CCNA_00697	3.133926446
757951..758000	-	233	CCNA_00698	6.386385094
758051..758100	+	99	CCNA_00699	6.176548941
763351..763400	-	138	CCNA_00705	2.364527793
785001..785050	+	458	CCNA_00729	2.889118176
793101..793150	-	189	CCNA_00735	3.3437626
811301..811350	-	292	CCNA_00752	2.329554814
814151..814200	-	92	CCNA_00756	3.763434906
822951..823000	-	248	CCNA_00764	3.641030339
823051..823100	+	112	CCNA_00765	2.556877457
824551..824600	-	84	CCNA_00767	4.043216156
824651..824700	+	74	CCNA_00768	5.354690819
839751..839800	-	610	CCNA_00778	6.333925625
840101..840150	+	153	CCNA_00779	2.18966462
841701..841750	-	308	CCNA_00780	3.780920533
841751..841800	+	118	CCNA_00781	4.130647742
844151..844200	-	561	CCNA_00782	4.427915482
851401..851450	-	288	CCNA_00787	5.651958559
851451..851500	+	450	CCNA_00788	4.253052309
853551..853600	-	216	CCNA_00789	2.46944587
853601..853650	+	128	CCNA_00790	2.556877457
867651..867700	+	39	CCNA_00802	3.62354385

867801..867850	+	172	CCNA_00803	5.267259232
871701..871750	-	615	CCNA_00806	2.032287074
872251..872300	-	44	CCNA_00808	2.661795533
915201..915250	-	574	CCNA_00842	2.242123227
920551..920600	-	1029	CCNA_00846	2.574363947
920751..920800	+	155	CCNA_00847	8.4847449
927551..927600	+	204	CCNA_00852	3.203871543
938801..938850	-	78	CCNA_00860	4.008243176
955001..955050	-	202	CCNA_00877	4.532833559
955101..955150	+	476	CCNA_00878	2.801685727
973901..973950	+	383	CCNA_00894	2.137205151
996201..996250	+	74	CCNA_00919	4.777641829
1005101..1005150	-	240	CCNA_00928	2.976549763
1014451..1014500	+	167	CCNA_00939	3.081467839
1050851..1050900	-	135	CCNA_00973	2.067260054
1057001..1057050	-	594	CCNA_00976	20.76015209
1057101..1057150	+	136	CCNA_00977	12.75141047
1081901..1081950	+	177	CCNA_00999	2.48693236
1132851..1132900	-	261	CCNA_01037	2.347041304
1139951..1140000	-	71	CCNA_01043	3.536112263
1140051..1140100	+	210	CCNA_01044	3.763434906
1141751..1141800	-	217	CCNA_01046	13.85304984
1142001..1142050	+	903	CCNA_01047	3.58857087
1159051..1159100	-	498	CCNA_01057	3.133926446
1159651..1159700	-	99	CCNA_01058	2.941576783
1159601..1159650	+	1073	CCNA_01059	2.854145197
1162901..1162950	+	578	CCNA_01060	5.267259232
1174251..1174300	-	429	CCNA_01068	2.48693236
1174201..1174250	+	554	CCNA_01069	2.102233033
1193051..1193100	-	602	CCNA_01086	5.162341156
1193201..1193250	+	221	CCNA_01087	4.113161253
1199251..1199300	-	98	CCNA_01093	4.795128319
1204001..1204050	-	66	CCNA_01098	8.344853844
1213601..1213650	-	286	CCNA_01108	6.246494038
1213751..1213800	+	596	CCNA_01109	4.183106349
1291701..1291750	+	112	CCNA_01171	8.100045574
1291851..1291900	+	280	CCNA_01172	2.679282023
1301801..1301850	-	174	CCNA_01181	3.151412936
1369201..1369250	-	546	CCNA_01242	3.518625773
1369251..1369300	+	823	CCNA_01243	3.710975436
1399951..1400000	-	153	CCNA_01273	2.294582697
1422951..1423000	-	278	CCNA_01297	18.50441474
1423251..1423300	+	396	CCNA_01298	2.7842001
1431051..1431100	+	110	CCNA_01304	17.90987926
1431101..1431150	+	102	CCNA_01305	20.8126107
1443251..1443300	+	122	CCNA_01328	8.904417206
1493151..1493200	+	488	CCNA_01379	2.242123227
1502251..1502300	+	266	CCNA_01385	2.644309043
1520951..1521000	-	88	CCNA_01402	3.081467839
1520901..1520950	+	299	CCNA_01403	3.168899426
1532801..1532850	+	265	CCNA_01415	2.014801447
1534701..1534750	+	408	CCNA_01417	2.661795533
1544951..1545000	-	160	CCNA_01427	4.078188273

1544851..1544900	+	39	CCNA_01428	2.17217813
1545001..1545050	+	176	CCNA_01429	4.270537936
1549351..1549400	+	40	CCNA_01436	15.0421208
1552801..1552850	+	157	CCNA_01440	3.01152188
1557651..1557700	-	672	CCNA_01444	2.312068324
1560751..1560800	+	317	CCNA_01448	2.294582697
1564851..1564900	+	196	CCNA_01451	4.602778656
1566401..1566450	+	101	CCNA_01453	11.35250336
1571951..1572000	-	369	CCNA_01463	4.760155339
1631451..1631500	-	289	CCNA_01522	3.168899426
1639351..1639400	+	954	CCNA_01532	3.833380003
1646101..1646150	+	896	CCNA_01538	2.609336064
1654901..1654950	-	446	CCNA_01543	3.06398135
1658701..1658750	-	741	CCNA_01546	6.159062451
1665701..1665750	+	882	CCNA_01552	2.17217813
1694651..1694700	+	120	CCNA_01578	3.88583861
1733001..1733050	+	347	CCNA_01612	2.50441885
1741601..1741650	+	119	CCNA_01621	10.21589187
1765551..1765600	-	298	CCNA_01644	3.676002457
1765601..1765650	+	286	CCNA_01645	3.203871543
1781951..1782000	-	230	CCNA_01660	14.13283109
1782051..1782100	+	104	CCNA_01662	8.50223139
1797601..1797650	+	594	CCNA_01675	2.049773564
1812351..1812400	-	294	CCNA_01684	9.149225477
1815901..1815950	+	487	CCNA_01689	7.557969564
1845851..1845900	+	212	CCNA_01715	4.218079329
1850501..1850550	+	250	CCNA_01724	2.73174063
1866301..1866350	-	507	CCNA_01737	4.532833559
1870601..1870650	-	126	CCNA_01741	13.03119172
1870701..1870750	+	219	CCNA_01742	19.37873147
1877001..1877050	+	78	CCNA_01749	2.347041304
1887151..1887200	-	449	CCNA_01759	2.45195938
1891851..1891900	-	379	CCNA_01762	2.941576783
1898251..1898300	+	139	CCNA_01770	2.609336064
1917201..1917250	-	171	CCNA_01789	12.59403379
1917251..1917300	+	155	CCNA_01790	11.50988091
1917851..1917900	+	550	CCNA_01791	11.0202635
1919501..1919550	+	60	CCNA_01792	5.022450962
1923651..1923700	+	343	CCNA_01799	2.43447289
1934401..1934450	-	358	CCNA_01808	2.574363947
1934451..1934500	+	325	CCNA_01809	2.7842001
1941251..1941300	+	747	CCNA_01816	2.48693236
1945901..1945950	+	82	CCNA_01819	2.18966462
1950951..1951000	-	111	CCNA_01824	2.17217813
1951051..1951100	+	650	CCNA_01826	3.02900837
1985401..1985450	+	134	CCNA_01853	2.889118176
2044451..2044500	-	329	CCNA_01901	4.340483895
2072451..2072500	-	485	CCNA_01925	2.119718661
2072551..2072600	+	353	CCNA_01926	4.078188273
2085451..2085500	-	158	CCNA_01942	2.801685727
2088001..2088050	-	377	CCNA_01945	2.976549763
2090301..2090350	+	383	CCNA_01948	2.416986401
2093201..2093250	-	371	CCNA_01950	2.20715111

2097101..2097150	-	395	CCNA_01952	2.959063273
2107951..2108000	-	131	CCNA_01964	4.357969523
2108101..2108150	-	40	CCNA_01965	6.019171395
2108151..2108200	+	907	CCNA_01966	4.707696732
2147251..2147300	-	268	CCNA_02000	17.7175296
2147301..2147350	+	216	CCNA_02001	14.6749071
2157001..2157050	-	443	CCNA_02008	2.014801447
2177601..2177650	-	125	CCNA_02033	12.92627364
2177701..2177750	+	337	CCNA_02034	4.935018513
2180501..2180550	-	402	CCNA_02035	2.277096207
2188551..2188600	-	454	CCNA_02042	3.571085242
2214551..2214600	-	309	CCNA_02063	2.626822554
2229851..2229900	-	482	CCNA_02078	3.378735579
2232601..2232650	-	200	CCNA_02081	2.18966462
2232751..2232800	-	74	CCNA_02082	4.865073416
2247501..2247550	+	298	CCNA_02098	3.676002457
2279251..2279300	-	974	CCNA_02125	3.29130313
2279301..2279350	+	71	CCNA_02126	3.378735579
2294201..2294250	-	487	CCNA_02136	7.260701824
2308501..2308550	-	84	CCNA_02151	2.539390967
2319201..2319250	-	353	CCNA_02164	2.73174063
2344451..2344500	-	205	CCNA_02193	2.539390967
2344501..2344550	+	463	CCNA_02194	2.609336064
2379601..2379650	-	555	CCNA_02231	2.18966462
2391651..2391700	-	144	CCNA_02242	4.690210242
2391751..2391800	+	451	CCNA_02243	5.651958559
2394601..2394650	-	425	CCNA_02244	2.277096207
2408951..2409000	-	489	CCNA_02256	5.477095385
2409051..2409100	+	241	CCNA_02258	2.836658707
2421901..2421950	+	330	CCNA_02269	6.386385094
2435601..2435650	-	36	CCNA_02286	6.771084421
2435651..2435700	+	215	CCNA_02287	5.774363125
2445851..2445900	-	343	CCNA_02298	2.102233033
2445801..2445850	+	235	CCNA_02299	2.644309043
2479751..2479800	-	298	CCNA_02339	2.591850436
2498001..2498050	-	498	CCNA_02357	2.46944587
2535001..2535050	+	259	CCNA_02391	8.624635957
2565801..2565850	-	302	CCNA_02421	3.431194186
2568901..2568950	-	57	CCNA_02425	3.920811589
2577101..2577150	-	429	CCNA_02435	3.518625773
2577601..2577650	-	156	CCNA_02436	16.14375931
2597451..2597500	-	207	CCNA_02455	4.847586926
2656401..2656450	-	46	CCNA_02506	3.483652793
2656501..2656550	+	501	CCNA_02507	6.491302308
2664851..2664900	-	512	CCNA_02513	3.466167166
2664901..2664950	+	246	CCNA_02514	2.836658707
2673451..2673500	+	332	CCNA_02525	3.133926446
2681151..2681200	-	60	CCNA_02534	3.221358033
2692201..2692250	+	55	CCNA_02543	14.60496201
2702201..2702250	+	120	CCNA_02554	6.893488125
2727651..2727700	-	104	CCNA_02575	8.79949913
2727701..2727750	+	252	CCNA_02576	6.771084421
2732251..2732300	+	48	CCNA_02582	2.312068324

2732401..2732450	+	220	CCNA_02583	4.935018513
2747551..2747600	-	369	CCNA_02601	5.756876635
2747601..2747650	+	292	CCNA_02602	4.690210242
2775851..2775900	-	302	CCNA_02625	2.242123227
2830151..2830200	-	298	CCNA_02676	2.277096207
2859351..2859400	+	758	CCNA_02704	2.556877457
2868401..2868450	-	31	CCNA_02707	10.95031841
2868251..2868300	+	143	CCNA_02708	13.81807686
2870351..2870400	+	147	CCNA_02711	2.014801447
2879851..2879900	+	204	CCNA_02719	2.46944587
2895101..2895150	-	68	CCNA_02732	2.889118176
2897151..2897200	-	290	CCNA_02734	2.661795533
2910251..2910300	+	84	CCNA_02748	2.067260054
2934401..2934450	-	63	CCNA_02775	4.952505002
2940451..2940500	-	161	CCNA_02782	2.20715111
2940501..2940550	+	572	CCNA_02783	2.45195938
2960601..2960650	+	137	CCNA_02805	3.833380003
2974251..2974300	-	123	CCNA_02816	6.019171395
3069401..3069450	-	979	CCNA_02910	4.025729666
3086651..3086700	-	92	CCNA_02924	5.896767691
3086701..3086750	+	388	CCNA_02925	5.634472069
3101451..3101500	+	157	CCNA_02941	3.833380003
3119701..3119750	+	348	CCNA_02964	2.084746544
3150201..3150250	-	1099	CCNA_02994	5.809336105
3159551..3159600	+	377	CCNA_03006	15.95140965
3165101..3165150	-	169	CCNA_03011	3.466167166
3165251..3165300	+	187	CCNA_03012	6.893488125
3184601..3184650	-	245	CCNA_03029	6.019171395
3184751..3184800	+	228	CCNA_03030	6.928461104
3197801..3197850	-	297	CCNA_03041	2.801685727
3199501..3199550	+	183	CCNA_03045	2.259609717
3215001..3215050	-	370	CCNA_03062	4.672723753
3236051..3236100	-	184	CCNA_03086	2.067260054
3236001..3236050	+	186	CCNA_03087	2.329554814
3240251..3240300	-	320	CCNA_03090	3.88583861
3240551..3240600	+	66	CCNA_03091	3.641030339
3240701..3240750	+	458	CCNA_03092	12.26179307
3256451..3256500	-	216	CCNA_03105	4.148134232
3256501..3256550	+	97	CCNA_03106	4.532833559
3260951..3261000	-	1112	CCNA_03108	2.539390967
3261651..3261700	-	122	CCNA_03110	2.50441885
3261801..3261850	-	35	CCNA_03111	2.382014283
3264851..3264900	+	336	CCNA_03115	2.224636737
3268051..3268100	+	158	CCNA_03120	2.137205151
3279651..3279700	-	231	CCNA_03130	4.200592839
3282751..3282800	+	444	CCNA_03135	2.626822554
3286001..3286050	+	738	CCNA_03138	4.637751635
3294551..3294600	-	653	CCNA_03142	5.232286252
3298401..3298450	-	643	CCNA_03144	2.924090293
3298451..3298500	+	119	CCNA_03145	3.151412936
3316601..3316650	-	171	CCNA_03160	8.187477161
3316651..3316700	+	351	CCNA_03161	12.10441638
3320501..3320550	-	323	CCNA_03163	2.609336064

3320551..3320600	+	923	CCNA_03164	3.098953467
3363151..3363200	-	428	CCNA_03201	3.081467839
3363751..3363800	-	208	CCNA_03202	2.609336064
3363801..3363850	+	325	CCNA_03203	2.539390967
3376201..3376250	-	136	CCNA_03214	7.995127497
3376101..3376150	+	471	CCNA_03215	4.392942503
3377951..3378000	-	82	CCNA_03216	3.62354385
3426751..3426800	+	372	CCNA_03256	3.536112263
3428401..3428450	+	180	CCNA_03257	2.854145197
3430101..3430150	-	121	CCNA_03260	2.871631686
3451001..3451050	-	1146	CCNA_03280	4.183106349
3450851..3450900	+	234	CCNA_03281	2.032287074
3466551..3466600	+	411	CCNA_03296	2.312068324
3484151..3484200	-	123	CCNA_03306	10.460701
3484101..3484150	+	88	CCNA_03307	5.547040482
3484301..3484350	+	289	CCNA_03308	19.48364955
3487451..3487500	-	105	CCNA_03311	6.106602982
3487551..3487600	+	75	CCNA_03312	15.09457941
3516501..3516550	-	626	CCNA_03334	2.347041304
3535951..3536000	-	127	CCNA_03357	6.403870722
3536001..3536050	+	335	CCNA_03358	8.065072594
3546051..3546100	-	201	CCNA_03369	2.312068324
3547401..3547450	-	159	CCNA_03371	3.221358033
3547551..3547600	-	61	CCNA_03372	7.365619038
3547601..3547650	+	214	CCNA_03373	7.872722931
3550601..3550650	-	151	CCNA_03377	4.847586926
3550701..3550750	+	305	CCNA_03378	6.736111441
3557101..3557150	-	617	CCNA_03383	11.36998985
3557201..3557250	+	76	CCNA_03384	10.5306461
3565451..3565500	-	192	CCNA_03393	3.501139283
3565301..3565350	+	237	CCNA_03394	3.186385054
3566401..3566450	+	119	CCNA_03395	5.651958559
3566551..3566600	+	574	CCNA_03396	2.679282023
3570001..3570050	-	205	CCNA_03399	3.186385054
3570101..3570150	+	70	CCNA_03400	2.73174063
3571151..3571200	-	237	CCNA_03401	5.966712788
3572401..3572450	-	324	CCNA_03402	2.294582697
3572351..3572400	+	68	CCNA_03403	2.014801447
3572451..3572500	+	169	CCNA_03404	2.18966462
3573901..3573950	-	225	CCNA_03405	8.4847449
3573951..3574000	+	87	CCNA_03406	7.540483074
3592401..3592450	-	190	CCNA_03426	3.973270196
3592451..3592500	+	155	CCNA_03427	6.176548941
3593301..3593350	-	89	CCNA_03428	3.86835212
3594001..3594050	-	205	CCNA_03429	11.49239442
3594151..3594200	-	41	CCNA_03430	19.29129988
3594201..3594250	+	433	CCNA_03431	14.91971624
3624601..3624650	-	139	CCNA_03461	2.74922712
3637601..3637650	+	319	CCNA_03471	4.270537936
3639701..3639750	-	75	CCNA_03474	3.01152188
3643201..3643250	-	148	CCNA_03479	2.014801447
3655051..3655100	-	265	CCNA_03495	2.119718661
3676601..3676650	-	331	CCNA_03518	5.407150288

3676651..3676700	+	108	CCNA_03519	5.477095385
3692501..3692550	-	597	CCNA_03533	2.416986401
3692801..3692850	+	328	CCNA_03534	5.319718702
3702001..3702050	+	181	CCNA_03545	7.767804854
3702151..3702200	+	173	CCNA_03546	4.445401972
3708751..3708800	+	394	CCNA_03552	3.693488946
3713801..3713850	-	251	CCNA_03557	6.316439135
3719401..3719450	-	197	CCNA_03563	5.984199278
3720251..3720300	-	150	CCNA_03565	5.879281202
3720301..3720350	+	178	CCNA_03566	6.368898605
3720801..3720850	+	435	CCNA_03567	26.40823829
3730301..3730350	-	847	CCNA_03574	4.322997406
3730351..3730400	+	395	CCNA_03575	3.850866492
3737851..3737900	-	152	CCNA_03580	2.102233033
3740001..3740050	-	80	CCNA_03583	2.941576783
3739951..3740000	+	253	CCNA_03584	4.235565819
3747801..3747850	-	375	CCNA_03593	2.976549763
3747851..3747900	+	238	CCNA_03594	2.74922712
3750151..3750200	-	136	CCNA_03597	2.46944587
3750251..3750300	+	769	CCNA_03598	4.095674763
3752701..3752750	+	348	CCNA_03599	5.057423079
3754951..3755000	+	232	CCNA_03601	2.941576783
3764101..3764150	-	449	CCNA_03609	11.35250336
3777451..3777500	-	321	CCNA_03621	2.382014283
3777501..3777550	+	120	CCNA_03622	2.014801447
3790701..3790750	-	341	CCNA_03636	3.116439957
3790751..3790800	+	509	CCNA_03637	2.45195938
3798301..3798350	-	76	CCNA_03638	11.30004475
3803651..3803700	-	135	CCNA_03644	3.431194186
3818451..3818500	-	307	CCNA_03656	3.536112263
3820501..3820550	-	410	CCNA_03658	2.45195938
3824801..3824850	-	766	CCNA_03663	2.014801447
3824851..3824900	+	268	CCNA_03664	2.049773564
3834151..3834200	-	114	CCNA_03674	2.294582697
3834201..3834250	+	612	CCNA_03675	3.58857087
3843901..3843950	+	226	CCNA_03682	2.801685727
3854551..3854600	-	293	CCNA_03687	3.571085242
3854751..3854800	-	157	CCNA_03688	9.726274467
3854851..3854900	+	370	CCNA_03689	14.79731167
3859901..3859950	-	251	CCNA_03693	3.30878962
3860001..3860050	+	314	CCNA_03694	6.386385094
3869101..3869150	-	139	CCNA_03700	2.032287074
3871551..3871600	-	569	CCNA_03702	10.5306461
3873751..3873800	-	443	CCNA_03704	3.850866492
3873701..3873750	+	188	CCNA_03705	4.952505002
3874351..3874400	+	142	CCNA_03706	9.516438313
3879851..3879900	-	497	CCNA_03713	2.084746544
3881501..3881550	-	186	CCNA_03715	2.20715111
3882751..3882800	-	205	CCNA_03717	8.659608074
3882851..3882900	+	319	CCNA_03718	15.1120659
3904001..3904050	-	678	CCNA_03734	7.488023604
3904051..3904100	+	671	CCNA_03735	5.214800625
3906501..3906550	+	123	CCNA_03736	9.35906163

3906651..3906700	+	98	CCNA_03737	3.256331013
3910601..3910650	-	328	CCNA_03740	2.329554814
3915601..3915650	-	280	CCNA_03745	3.151412936
3915801..3915850	+	391	CCNA_03746	3.536112263
3924401..3924450	-	232	CCNA_03754	4.602778656
3931401..3931450	+	32	CCNA_03762	5.197314135
3932751..3932800	+	284	CCNA_03764	2.539390967
3937051..3937100	-	508	CCNA_03768	2.889118176
3938851..3938900	-	320	CCNA_03770	3.676002457
3938901..3938950	+	348	CCNA_03771	3.01152188
3944501..3944550	-	350	CCNA_03777	3.30878962
3944601..3944650	+	250	CCNA_03778	2.626822554
3945951..3946000	-	110	CCNA_03779	5.074909569
3947151..3947200	-	247	CCNA_03780	2.48693236
3950001..3950050	-	895	CCNA_03781	2.71425414
3950151..3950200	+	204	CCNA_03782	2.696768513
3955251..3955300	+	91	CCNA_03790	2.609336064
3957801..3957850	-	313	CCNA_03793	4.777641829
3957851..3957900	+	229	CCNA_03794	4.900046395
3975751..3975800	-	300	CCNA_03811	7.575455191
3975801..3975850	+	183	CCNA_03812	5.407150288
3976551..3976600	+	457	CCNA_03813	2.871631686
3987301..3987350	+	268	CCNA_03821	3.378735579
3993701..3993750	+	155	CCNA_03828	2.941576783
3994601..3994650	-	139	CCNA_03829	13.0661647
3994551..3994600	+	396	CCNA_03830	11.94703884
3998751..3998800	-	278	CCNA_03833	2.976549763
4002451..4002500	-	170	CCNA_03836	3.396221207
4009651..4009700	-	256	CCNA_03842	2.416986401
4014851..4014900	-	309	CCNA_03848	3.203871543
4014801..4014850	+	365	CCNA_03849	2.224636737
4021251..4021300	-	395	CCNA_03856	2.084746544
4021651..4021700	-	234	CCNA_03857	3.431194186
4021701..4021750	+	163	CCNA_03858	2.137205151
4022851..4022900	-	232	CCNA_03859	3.116439957
4022801..4022850	+	169	CCNA_03860	4.025729666
4025251..4025300	-	150	CCNA_03863	6.351412115
4025301..4025350	+	234	CCNA_03864	5.092396059
475151..475200	+	413	CCNA_04006	4.008243176
2456951..2457000	+	112	CCNA_04009	3.168899426
3637551..3637600	-	240	CCNA_04011	4.183106349

Start..end	Strand	Product length	Gene code	Z-score coverage ChIP-seq m6A wt
51..100	+	301	CCNA_00001	4.8827815
3101..3150	+	237	CCNA_00005	2.67743515
3951..4000	+	262	CCNA_00006	2.15720025
4801..4850	+	96	CCNA_00007	2.89231536
5401..5450	+	490	CCNA_00008	2.63219791
7901..7950	-	206	CCNA_00009	3.68397778
10051..10100	+	385	CCNA_00011	2.55303146
19051..19100	-	79	CCNA_00017	2.81314891
20701..20750	-	74	CCNA_00020	4.26075999
26851..26900	-	550	CCNA_00025	2.14589017
30151..30200	-	229	CCNA_00027	2.68874523
32901..32950	+	210	CCNA_00029	2.39469856
33551..33600	+	244	CCNA_00030	2.5077932
34751..34800	-	128	CCNA_00031	4.35123652
34901..34950	-	80	CCNA_00032	2.0101764
45751..45800	-	1037	CCNA_00041	2.3494603
48301..48350	-	548	CCNA_00043	2.10065293
49051..49100	+	177	CCNA_00045	3.86492878
52151..52200	-	407	CCNA_00048	3.26552744
52651..52700	-	144	CCNA_00049	2.15720025
55851..55900	-	157	CCNA_00052	2.49648415
58351..58400	-	448	CCNA_00054	2.1685093
58551..58600	-	135	CCNA_00055	4.38516471
60001..60050	-	211	CCNA_00057	2.33815125
73601..73650	+	304	CCNA_00072	2.4173177
74401..74450	+	511	CCNA_00073	2.47386501
78651..78700	-	733	CCNA_00074	2.80183986
81851..81900	-	391	CCNA_00077	4.55480666
85101..85150	-	69	CCNA_00081	4.50956839
87101..87150	-	552	CCNA_00082	2.48517406
97851..97900	+	516	CCNA_00088	2.23636567
104201..1042	-	297	CCNA_00091	3.67266873
105301..1053	-	69	CCNA_00093	2.74529254
106201..1062	-	306	CCNA_00094	2.72267341
115951..1160	-	233	CCNA_00104	2.88100528
118451..1185	-	65	CCNA_00107	3.9214761
131701..1317	+	153	CCNA_00119	5.82146674
135401..1354	-	218	CCNA_00124	3.67266873
141501..1415	+	266	CCNA_00132	2.58695965
148851..1489	-	514	CCNA_00137	2.10065293
148701..1487	+	730	CCNA_00138	3.29945562
155751..1558	-	290	CCNA_00143	4.8827815
156901..1569	-	384	CCNA_00144	2.1685093
157801..1578	-	200	CCNA_00145	3.01671904
160301..1603	-	325	CCNA_00150	4.86016237
161401..1614	-	238	CCNA_00151	2.20243748
164951..1650	+	123	CCNA_00157	2.0101764
166701..1667	+	821	CCNA_00159	3.88754791
171351..1714	+	422	CCNA_00162	2.33815125

172801..1728	+	739	CCNA_00163	2.12327104
175101..1751	+	641	CCNA_00164	2.31553212
192601..1926	+	324	CCNA_00179	3.05064722
195251..1953	+	238	CCNA_00182	2.47386501
197101..1971	-	253	CCNA_00183	3.4012412
199901..1999	-	841	CCNA_00185	3.3107657
204651..2047	+	158	CCNA_00191	2.37207943
208001..2080	-	350	CCNA_00193	2.11196198
215701..2157	-	346	CCNA_00200	2.96017173
215051..2151	+	226	CCNA_00201	2.20243748
215901..2159	+	351	CCNA_00202	3.87623886
217001..2170	+	347	CCNA_00203	3.60481133
225301..2253	-	524	CCNA_00209	8.42264431
228551..2286	+	292	CCNA_00212	2.98279086
233851..2339	-	130	CCNA_00216	3.44647844
233901..2339	+	716	CCNA_00217	2.56434051
247951..2480	-	138	CCNA_00232	2.37207943
248001..2480	+	345	CCNA_00233	2.58695965
253151..2532	+	534	CCNA_00238	2.86969623
256501..2565	-	51	CCNA_00242	2.97148181
256801..2568	-	158	CCNA_00243	2.31553212
257601..2576	+	94	CCNA_00245	3.15243281
259701..2597	-	185	CCNA_00247	2.27029488
260551..2606	+	196	CCNA_00249	2.02148649
266801..2668	+	300	CCNA_00254	3.36731302
273901..2739	-	236	CCNA_00260	2.85838718
273651..2737	+	347	CCNA_00261	2.36077038
281901..2819	-	313	CCNA_00267	8.49050068
282001..2820	+	623	CCNA_00268	2.42862675
286651..2867	+	173	CCNA_00273	2.2589848
289551..2896	-	481	CCNA_00275	2.22505662
294601..2946	+	110	CCNA_00282	2.55303146
296101..2961	-	286	CCNA_00283	3.37862207
296701..2967	-	221	CCNA_00284	3.79707242
299451..2995	-	368	CCNA_00287	3.86492878
304801..3048	-	1145	CCNA_00290	2.51910225
304401..3044	+	471	CCNA_00291	4.8149241
305651..3057	+	477	CCNA_00292	2.3494603
309351..3094	+	230	CCNA_00295	2.96017173
310351..3104	+	230	CCNA_00296	2.11196198
311801..3118	-	285	CCNA_00297	2.08934285
314601..3146	+	360	CCNA_00301	2.5982687
319551..3196	-	425	CCNA_00304	2.53041233
333101..3331	+	345	CCNA_00318	2.58695965
335001..3350	-	177	CCNA_00319	2.19112843
335151..3352	-	89	CCNA_00320	9.92680271
336101..3361	-	171	CCNA_00321	3.05064722
335951..3360	+	209	CCNA_00322	3.08457644
338501..3385	-	42	CCNA_00325	2.75660159
338551..3386	+	343	CCNA_00326	3.78576336
341301..3413	+	182	CCNA_00329	2.17981835
342601..3426	-	256	CCNA_00330	3.61612142
348201..3482	+	824	CCNA_00338	2.97148181

352101..3521	+	399	CCNA_00340	2.98279086
357751..3578	+	402	CCNA_00343	2.08934285
360051..3601	+	475	CCNA_00346	2.46255493
361601..3616	+	59	CCNA_00347	2.36077038
366001..3660	-	226	CCNA_00350	3.59350228
366051..3661	+	719	CCNA_00351	2.1685093
373451..3735	+	475	CCNA_00357	4.50956839
378751..3788	-	315	CCNA_00361	2.72267341
383551..3836	+	341	CCNA_00367	3.94409523
385501..3855	+	437	CCNA_00369	5.65182479
388951..3890	-	74	CCNA_00372	3.69528683
390601..3906	+	129	CCNA_00375	3.20898012
396651..3967	-	204	CCNA_00379	3.84231068
399751..3998	-	358	CCNA_00382	5.74230029
400301..4003	+	301	CCNA_00384	3.11850462
401351..4014	+	444	CCNA_00385	2.60957878
403501..4035	+	164	CCNA_00387	2.4173177
405951..4060	+	404	CCNA_00390	3.3107657
407601..4076	+	444	CCNA_00392	2.77922073
413351..4134	+	512	CCNA_00397	2.08934285
416951..4170	-	449	CCNA_00398	4.14766536
416751..4168	+	414	CCNA_00399	3.25421839
422401..4224	+	163	CCNA_00407	2.68874523
424151..4242	+	47	CCNA_00409	2.04410562
425601..4256	+	216	CCNA_00412	2.66612609
426351..4264	+	417	CCNA_00413	2.90362441
429751..4298	-	336	CCNA_00415	3.41255025
436501..4365	+	114	CCNA_00426	4.35123652
445301..4453	-	124	CCNA_00438	6.95241306
445451..4455	+	673	CCNA_00439	8.33216778
447801..4478	+	121	CCNA_00441	3.03933817
451851..4519	+	344	CCNA_00445	4.09111805
452651..4527	+	129	CCNA_00446	2.02148649
453301..4533	+	187	CCNA_00447	2.60957878
453451..4535	+	127	CCNA_00448	5.13158887
455201..4552	+	1013	CCNA_00451	4.80361505
462101..4621	-	270	CCNA_00454	4.27207008
475351..4754	+	413	CCNA_04006	3.15243281
477401..4774	+	54	CCNA_00464	5.64051574
477601..4776	+	370	CCNA_00465	2.20243748
479251..4793	+	414	CCNA_00466	2.2589848
482001..4820	-	37	CCNA_00468	4.21552276
483951..4840	-	459	CCNA_00469	3.85361973
486101..4861	-	447	CCNA_00470	2.05541467
488351..4884	-	358	CCNA_00472	4.12504726
493301..4933	-	605	CCNA_00475	2.91493449
494451..4945	-	64	CCNA_00477	2.27029488
497001..4970	+	96	CCNA_00481	3.25421839
509851..5099	-	212	CCNA_00491	2.04410562
509651..5097	+	463	CCNA_00492	3.26552744
514051..5141	+	308	CCNA_00497	2.3268422
518401..5184	-	559	CCNA_00501	2.5982687
520501..5205	-	154	CCNA_00504	3.84231068

520051..5201	+	183	CCNA_00505	2.67743515
520551..5206	+	426	CCNA_00506	3.15243281
522151..5222	+	281	CCNA_00507	2.67743515
535201..5352	-	358	CCNA_00519	3.59350228
536451..5365	-	83	CCNA_00521	2.88100528
540201..5402	+	168	CCNA_00527	3.14112376
542601..5426	+	172	CCNA_00530	5.154208
547551..5476	+	1356	CCNA_00536	2.70005428
561551..5616	-	107	CCNA_00542	3.38993112
565651..5657	+	82	CCNA_00547	4.9053996
565851..5659	+	88	CCNA_00548	4.22683181
567051..5671	+	348	CCNA_00550	2.3494603
568701..5687	-	146	CCNA_00551	2.93755259
569701..5697	-	156	CCNA_00552	3.52564489
570201..5702	+	268	CCNA_00554	3.25421839
571051..5711	+	114	CCNA_00555	2.13458112
582051..5821	-	580	CCNA_00565	4.05718986
586001..5860	-	770	CCNA_00567	2.90362441
588451..5885	-	358	CCNA_00569	3.50302678
589201..5892	+	878	CCNA_00571	2.45124588
613101..6131	-	455	CCNA_00583	3.08457644
612701..6127	+	141	CCNA_00584	2.42862675
612901..6129	+	351	CCNA_00585	3.34469389
615801..6158	-	443	CCNA_00586	3.20898012
627901..6279	-	329	CCNA_00595	5.94587042
647201..6472	+	348	CCNA_00609	4.12504726
648301..6483	+	867	CCNA_00610	3.45778852
655751..6558	-	447	CCNA_00613	3.06195731
660551..6606	-	162	CCNA_00616	2.4173177
664101..6641	+	392	CCNA_00620	2.51910225
670501..6705	+	125	CCNA_00625	2.12327104
673001..6730	-	637	CCNA_00626	3.38993112
675901..6759	+	764	CCNA_00630	2.75660159
678651..6787	+	150	CCNA_00631	2.55303146
678701..6787	+	122	CCNA_00632	3.28814657
692401..6924	-	91	CCNA_00640	4.23814189
692651..6927	-	98	CCNA_00641	2.97148181
692851..6929	-	85	CCNA_00642	3.36731302
698501..6985	+	240	CCNA_00648	2.33815125
704601..7046	+	892	CCNA_00653	2.49648415
710401..7104	-	660	CCNA_00655	2.53041233
722001..7220	+	381	CCNA_00665	4.74706774
722701..7227	+	117	CCNA_00666	2.13458112
729001..7290	-	250	CCNA_00670	3.43516939
735151..7352	-	143	CCNA_00678	3.02802912
741751..7418	-	205	CCNA_00685	3.01671904
745051..7451	-	436	CCNA_00688	2.29291298
757201..7572	-	113	CCNA_00697	3.77445328
758201..7582	-	233	CCNA_00698	3.14112376
757751..7578	+	99	CCNA_00699	3.68397778
762551..7626	+	65	CCNA_00704	3.00540999
771001..7710	-	89	CCNA_00714	3.52564489
771601..7716	-	66	CCNA_00716	3.86492878

771301..7713	+	38	CCNA_00717	3.12981367
774051..7741	+	413	CCNA_00719	4.14766536
775251..7753	+	726	CCNA_00720	2.2589848
789551..7896	-	637	CCNA_00731	2.06672372
789401..7894	+	350	CCNA_00732	2.31553212
799951..8000	-	152	CCNA_00741	2.4173177
800901..8009	+	188	CCNA_00743	2.13458112
804901..8049	-	452	CCNA_00747	4.973257
804751..8048	+	107	CCNA_00748	4.03457073
805451..8055	+	631	CCNA_00749	2.47386501
818651..8187	-	824	CCNA_00759	2.67743515
820501..8205	-	566	CCNA_00760	4.74706774
824101..8241	-	192	CCNA_00766	6.95241306
838101..8381	-	842	CCNA_00777	2.24767575
839701..8397	-	610	CCNA_00778	4.30599826
846351..8464	-	658	CCNA_00783	2.23636567
846301..8463	+	407	CCNA_00784	3.35600294
850751..8508	-	108	CCNA_04003	4.73575766
853601..8536	+	128	CCNA_00790	2.31553212
856351..8564	+	455	CCNA_00793	3.06195731
859501..8595	-	202	CCNA_00795	5.57265835
867801..8678	+	172	CCNA_00803	2.3268422
870151..8702	-	229	CCNA_00805	2.77922073
875251..8753	-	380	CCNA_00810	4.14766536
884551..8846	-	130	CCNA_00822	5.41432648
899351..8994	+	273	CCNA_00834	3.29945562
902201..9022	+	273	CCNA_00836	2.54172138
915201..9152	-	574	CCNA_00842	2.03279554
941701..9417	-	591	CCNA_00862	2.06672372
954051..9541	-	57	CCNA_00875	2.63219791
955201..9552	+	476	CCNA_00878	2.31553212
964101..9641	-	128	CCNA_00884	2.11196198
964751..9648	-	182	CCNA_00885	2.27029488
965001..9650	+	415	CCNA_00886	2.0101764
967751..9678	-	158	CCNA_00889	3.93278618
975701..9757	+	54	CCNA_00897	2.63219791
978401..9784	-	356	CCNA_00899	3.69528683
978351..9784	+	735	CCNA_00900	2.70005428
981601..9816	-	278	CCNA_00901	2.72267341
982751..9828	+	515	CCNA_00903	3.25421839
993201..9932	-	58	CCNA_00913	2.5982687
996001..9960	+	74	CCNA_00919	3.00540999
998451..9985	+	859	CCNA_00922	2.0780338
1002601..100	-	180	CCNA_00925	2.5982687
1008601..100	+	474	CCNA_00933	2.06672372
1018301..101	-	410	CCNA_00941	2.20243748
1019351..101	-	307	CCNA_00942	3.02802912
1028601..102	+	340	CCNA_00951	6.13813151
1032501..103	-	44	CCNA_00955	3.27683649
1038851..103	-	148	CCNA_00962	2.83576804
1040901..104	-	232	CCNA_00965	2.76791064
1057301..105	+	136	CCNA_00977	8.35478691
1057501..105	+	122	CCNA_00978	2.67743515

1064051..106	-	605	CCNA_00983	2.4173177
1072051..107	-	156	CCNA_00990	2.33815125
1072901..107	-	148	CCNA_00991	3.11850462
1081951..108	+	177	CCNA_00999	6.27384527
1085601..108	+	139	CCNA_01005	3.71790597
1085951..108	+	103	CCNA_01006	2.20243748
1087101..108	-	89	CCNA_01007	2.33815125
1093301..109	+	235	CCNA_01013	2.75660159
1112801..111	-	207	CCNA_01025	2.0780338
1121751..112	+	331	CCNA_01033	2.03279554
1132901..113	-	261	CCNA_01037	2.30422307
1136901..113	-	44	CCNA_01041	2.65481601
1146151..114	+	542	CCNA_01050	4.01195263
1147951..114	+	1035	CCNA_01051	2.5077932
1153401..115	+	499	CCNA_01054	4.24945094
1157551..115	-	453	CCNA_01056	3.5821922
1159751..115	-	99	CCNA_01058	4.13635631
1164801..116	+	436	CCNA_01061	2.27029488
1166251..116	+	325	CCNA_01062	2.49648415
1177251..117	-	249	CCNA_01070	5.2220654
1179001..117	-	51	CCNA_01072	3.23159925
1191251..119	-	335	CCNA_01085	2.03279554
1204051..120	-	66	CCNA_01098	2.21374757
1205551..120	-	396	CCNA_01099	2.56434051
1211101..121	-	390	CCNA_01104	4.19290363
1211151..121	+	290	CCNA_01105	4.43040297
1212151..121	+	89	CCNA_01106	3.52564489
1212301..121	+	72	CCNA_01107	4.83754324
1213701..121	-	286	CCNA_01108	3.4012412
1213951..121	+	596	CCNA_01109	6.5452728
1220351..122	-	263	CCNA_01115	2.93755259
1220401..122	+	600	CCNA_01116	2.63219791
1224751..122	-	97	CCNA_01120	2.90362441
1224501..122	+	504	CCNA_01121	5.88932311
1233451..123	+	88	CCNA_01129	3.10719454
1233851..123	+	251	CCNA_01130	2.23636567
1234151..123	+	361	CCNA_01131	3.62743047
1245301..124	-	547	CCNA_01140	3.27683649
1245101..124	+	356	CCNA_01141	2.53041233
1251201..125	+	58	CCNA_01148	2.77922073
1253751..125	+	231	CCNA_01150	3.3107657
1258501..125	+	1001	CCNA_01155	3.16374186
1266751..126	+	451	CCNA_01159	2.99409991
1269701..126	+	767	CCNA_01162	4.37385463
1272201..127	+	2479	CCNA_01163	2.46255493
1291601..129	+	112	CCNA_01171	4.05718986
1293701..129	-	121	CCNA_01173	3.26552744
1301551..130	-	725	CCNA_01180	2.38338951
1301851..130	-	174	CCNA_01181	4.31730731
1302001..130	+	197	CCNA_01182	5.30123082
1303551..130	+	148	CCNA_01184	2.23636567
1319101..131	-	341	CCNA_01195	2.62088783
1331801..133	-	315	CCNA_01204	6.02503687

1331651..133	+	328	CCNA_01205	2.13458112
1336851..133	+	253	CCNA_01210	5.87801406
1339701..133	-	384	CCNA_01212	3.52564489
1342251..134	-	406	CCNA_01214	2.03279554
1343701..134	-	305	CCNA_01216	2.27029488
1343501..134	+	375	CCNA_01217	2.67743515
1344201..134	+	330	CCNA_01218	2.24767575
1347851..134	-	404	CCNA_01220	6.08158419
1347901..134	-	86	CCNA_01221	3.9214761
1352201..135	-	443	CCNA_01224	2.92624354
1369601..136	-	546	CCNA_01242	2.62088783
1376701..137	+	268	CCNA_01249	4.05718986
1381101..138	+	315	CCNA_01253	2.1685093
1382251..138	+	152	CCNA_01254	2.3268422
1386151..138	-	220	CCNA_01258	2.3494603
1388001..138	+	371	CCNA_01261	2.64350696
1395551..139	-	40	CCNA_01265	3.76314423
1405951..140	-	260	CCNA_01279	3.57088315
1410351..141	+	315	CCNA_01284	3.18636099
1412701..141	-	329	CCNA_01285	2.3494603
1412651..141	+	105	CCNA_01286	5.81015769
1413701..141	+	405	CCNA_01288	2.11196198
1418401..141	+	164	CCNA_01294	5.08635163
1423201..142	-	278	CCNA_01297	2.36077038
1423401..142	+	396	CCNA_01298	2.66612609
1431651..143	+	267	CCNA_01306	3.51433583
1434551..143	+	126	CCNA_01311	6.17205969
1434701..143	+	250	CCNA_01312	4.13635631
1435501..143	+	143	CCNA_01313	2.40600762
1436701..143	+	104	CCNA_01317	4.33992644
1437101..143	+	185	CCNA_01318	4.03457073
1441051..144	+	451	CCNA_01326	4.70182947
1442551..144	+	191	CCNA_01327	3.07326636
1443151..144	+	122	CCNA_01328	5.94587042
1444301..144	+	338	CCNA_01330	2.51910225
1448651..144	-	327	CCNA_01335	3.26552744
1451951..145	+	109	CCNA_01339	2.77922073
1454401..145	+	472	CCNA_01341	4.23814189
1455951..145	+	433	CCNA_01342	2.30422307
1456851..145	+	270	CCNA_01343	2.20243748
1457651..145	+	178	CCNA_01344	2.05541467
1462251..146	+	136	CCNA_01349	5.68575298
1462401..146	+	122	CCNA_01350	2.10065293
1466001..146	-	595	CCNA_01352	2.54172138
1485801..148	+	51	CCNA_01370	2.11196198
1492351..149	-	73	CCNA_01377	5.35777916
1492501..149	+	190	CCNA_01378	4.52087847
1500001..150	+	120	CCNA_01383	2.66612609
1505151..150	+	355	CCNA_01388	2.99409991
1506051..150	+	337	CCNA_01389	2.06672372
1516901..151	+	290	CCNA_01397	4.20421268
1529551..152	-	136	CCNA_04014	2.46255493
1529801..152	+	381	CCNA_01412	2.75660159

1531151..153	+	199	CCNA_01413	2.72267341
1531401..153	+	350	CCNA_01414	2.65481601
1532451..153	+	265	CCNA_01415	2.55303146
1534601..153	+	408	CCNA_01417	2.03279554
1536651..153	+	428	CCNA_01419	2.94886267
1538701..153	+	149	CCNA_01422	4.45302108
1539401..153	+	352	CCNA_01423	2.20243748
1541451..154	+	712	CCNA_01425	5.00718518
1548401..154	+	100	CCNA_01433	3.61612142
1549801..154	-	56	CCNA_01435	2.90362441
1553901..155	+	317	CCNA_01442	2.5982687
1557901..155	-	672	CCNA_01444	3.51433583
1557301..155	+	198	CCNA_01445	4.82623419
1557951..155	+	406	CCNA_01446	3.14112376
1559401..155	+	429	CCNA_01447	4.27207008
1565051..156	+	196	CCNA_01451	3.67266873
1572701..157	-	130	CCNA_01464	2.81314891
1572551..157	+	531	CCNA_01465	3.42386034
1574701..157	-	134	CCNA_01466	3.51433583
1574651..157	+	548	CCNA_01467	2.10065293
1576751..157	+	55	CCNA_01469	2.5077932
1586301..158	-	248	CCNA_01478	2.49648415
1586051..158	+	687	CCNA_01479	2.79052978
1591201..159	-	101	CCNA_01482	3.96671436
1591151..159	+	139	CCNA_01484	4.56611571
1594051..159	-	336	CCNA_01486	2.11196198
1593801..159	+	248	CCNA_01487	4.05718986
1594401..159	+	600	CCNA_01488	2.21374757
1606001..160	-	483	CCNA_01495	2.22505662
1606101..160	+	287	CCNA_01496	3.45778852
1616351..161	-	698	CCNA_01504	3.28814657
1618401..161	-	219	CCNA_01507	2.11196198
1627551..162	-	342	CCNA_01517	3.02802912
1632051..163	-	196	CCNA_01523	2.67743515
1634101..163	-	596	CCNA_01524	5.39170735
1634151..163	+	141	CCNA_01525	3.66135865
1634651..163	+	120	CCNA_01526	3.29945562
1636301..163	+	273	CCNA_01528	2.64350696
1637851..163	+	278	CCNA_01530	3.76314423
1639351..163	+	954	CCNA_01532	8.32085873
1642751..164	+	132	CCNA_01534	2.58695965
1643601..164	+	167	CCNA_01535	2.54172138
1653451..165	-	756	CCNA_01542	8.00419397
1659401..165	+	569	CCNA_01547	2.33815125
1672001..167	+	357	CCNA_01556	2.90362441
1676901..167	+	909	CCNA_01560	3.02802912
1679601..167	+	127	CCNA_01561	2.36077038
1684701..168	+	481	CCNA_01568	2.53041233
1694451..169	-	272	CCNA_01577	2.33815125
1694301..169	+	120	CCNA_01578	2.64350696
1709251..170	-	378	CCNA_01589	4.13635631
1709351..170	+	783	CCNA_01590	3.26552744
1719101..171	-	811	CCNA_01596	2.39469856

1719451..171	-	81	CCNA_01597	3.07326636
1719301..171	+	503	CCNA_01598	3.57088315
1722101..172	+	78	CCNA_01601	2.66612609
1724451..172	-	400	CCNA_01603	2.0780338
1726551..172	-	220	CCNA_01606	2.54172138
1734301..173	+	346	CCNA_01613	2.05541467
1751001..175	-	51	CCNA_01631	2.65481601
1755751..175	+	759	CCNA_01637	2.80183986
1761601..176	+	311	CCNA_01641	2.36077038
1768351..176	+	420	CCNA_01648	2.12327104
1771251..177	+	919	CCNA_01651	2.53041233
1781901..178	-	230	CCNA_01660	2.5982687
1782051..178	+	104	CCNA_01662	2.49648415
1786451..178	+	197	CCNA_01665	4.50956839
1786701..178	+	892	CCNA_01666	3.11850462
1789651..178	+	232	CCNA_01667	4.64528216
1795051..179	-	325	CCNA_01672	2.93755259
1797551..179	-	506	CCNA_01674	3.5821922
1803601..180	+	414	CCNA_01677	2.72267341
1805801..180	+	485	CCNA_01679	2.45124588
1812501..181	-	294	CCNA_01684	2.57565059
1813101..181	-	109	CCNA_01685	3.12981367
1812901..181	+	407	CCNA_01686	5.25599358
1813801..181	+	236	CCNA_01687	2.65481601
1815951..181	+	487	CCNA_01689	4.13635631
1819101..181	+	520	CCNA_01692	3.03933817
1840951..184	-	109	CCNA_01709	4.75837679
1844651..184	-	127	CCNA_01712	3.22028917
1844701..184	+	192	CCNA_01713	4.39647376
1845401..184	+	131	CCNA_01714	5.96848956
1850201..185	+	250	CCNA_01724	2.4173177
1851201..185	+	476	CCNA_01725	2.72267341
1856401..185	-	514	CCNA_01727	3.29945562
1858601..185	-	337	CCNA_01729	2.28160393
1859951..186	-	500	CCNA_01730	4.80361505
1863251..186	-	364	CCNA_01733	5.05242345
1864351..186	+	199	CCNA_01736	2.22505662
1873451..187	-	370	CCNA_01744	2.14589017
1874001..187	-	177	CCNA_01745	2.12327104
1877401..187	+	426	CCNA_01750	3.24290831
1878601..187	+	366	CCNA_01751	2.08934285
1881601..188	-	101	CCNA_01754	2.06672372
1889501..188	-	809	CCNA_01760	3.37862207
1895401..189	-	275	CCNA_01766	2.73398246
1901951..190	+	389	CCNA_01776	2.53041233
1907151..190	-	117	CCNA_01779	2.0780338
1911151..191	-	738	CCNA_01782	4.21552276
1912901..191	-	130	CCNA_01784	2.19112843
1928851..192	+	466	CCNA_01805	2.84707709
1930201..193	+	485	CCNA_01806	2.74529254
1933301..193	-	517	CCNA_01807	4.14766536
1937351..193	+	335	CCNA_01813	2.58695965
1941051..194	+	747	CCNA_01816	2.08934285

1956701..195	+	953	CCNA_01830	3.35600294
1959501..195	+	507	CCNA_01831	2.29291298
1977901..197	-	177	CCNA_01845	2.49648415
1979601..197	-	245	CCNA_01847	3.14112376
1985351..198	+	134	CCNA_01853	3.41255025
1985701..198	+	221	CCNA_01854	3.25421839
1986801..198	+	238	CCNA_01855	2.57565059
1987151..198	+	732	CCNA_01856	2.12327104
1991601..199	+	677	CCNA_01859	3.25421839
1998651..199	-	478	CCNA_01863	3.54826402
1998701..199	+	207	CCNA_01864	2.89231536
1999301..199	+	362	CCNA_01865	3.17505194
2016001..201	-	556	CCNA_01876	2.28160393
2018201..201	+	367	CCNA_01879	3.22028917
2020551..202	+	212	CCNA_01881	2.39469856
2022101..202	+	390	CCNA_01883	2.65481601
2027901..202	+	42	CCNA_01887	3.08457644
2039801..203	-	68	CCNA_01896	6.7940812
2042751..204	-	336	CCNA_01899	3.42386034
2044551..204	-	329	CCNA_01901	2.24767575
2045401..204	+	279	CCNA_01903	2.53041233
2050551..205	+	594	CCNA_01906	2.47386501
2053401..205	-	253	CCNA_01907	2.5077932
2055801..205	-	39	CCNA_01910	2.4173177
2056101..205	-	123	CCNA_01911	6.51134462
2056251..205	+	126	CCNA_01913	4.13635631
2056901..205	+	302	CCNA_01914	2.51910225
2061201..206	-	375	CCNA_01917	4.10242813
2061251..206	+	441	CCNA_01918	3.45778852
2067401..206	-	97	CCNA_01921	3.17505194
2072351..207	-	485	CCNA_01925	2.70005428
2072251..207	+	353	CCNA_01926	3.52564489
2073601..207	+	267	CCNA_01927	2.88100528
2082401..208	-	129	CCNA_01939	5.21075532
2086751..208	-	172	CCNA_01944	3.43516939
2088001..208	-	377	CCNA_01945	3.4917167
2088201..208	+	441	CCNA_01946	2.97148181
2089551..208	+	153	CCNA_01947	2.27029488
2095801..209	-	789	CCNA_01951	2.80183986
2097101..209	-	395	CCNA_01952	2.10065293
2103401..210	-	269	CCNA_01957	2.02148649
2106101..210	+	217	CCNA_01962	2.79052978
2111401..211	+	289	CCNA_01968	4.63397311
2115601..211	-	253	CCNA_01970	3.7405251
2118951..211	+	198	CCNA_01974	2.46255493
2121751..212	+	196	CCNA_01977	2.20243748
2135651..213	-	159	CCNA_01989	2.05541467
2141201..214	-	425	CCNA_01993	2.4173177
2142251..214	-	399	CCNA_01994	3.07326636
2146201..214	-	312	CCNA_01999	2.84707709
2152351..215	-	1143	CCNA_02003	3.54826402
2152401..215	-	40	CCNA_02004	3.52564489
2157901..215	-	139	CCNA_02011	2.31553212

2159751..215	-	559	CCNA_02012	3.4917167
2167351..216	-	217	CCNA_02019	3.06195731
2172751..217	-	439	CCNA_02024	3.00540999
2174601..217	-	110	CCNA_02028	2.21374757
2175901..217	-	131	CCNA_02030	3.26552744
2176551..217	-	197	CCNA_02031	2.27029488
2177451..217	-	125	CCNA_02033	2.24767575
2180101..218	-	402	CCNA_02035	4.32861739
2183151..218	+	110	CCNA_02038	2.42862675
2186851..218	-	213	CCNA_02041	2.22505662
2200201..220	+	695	CCNA_02052	2.57565059
2213451..221	-	568	CCNA_02062	3.02802912
2214451..221	-	309	CCNA_02063	2.77922073
2223201..222	-	103	CCNA_02071	2.5077932
2228401..222	-	263	CCNA_02077	2.10065293
2231851..223	-	300	CCNA_02080	2.76791064
2232551..223	-	200	CCNA_02081	3.61612142
2232701..223	-	74	CCNA_02082	4.09111805
2237901..223	-	394	CCNA_02087	2.12327104
2239151..223	+	309	CCNA_02091	2.28160393
2240701..224	-	212	CCNA_02092	2.54172138
2240501..224	+	619	CCNA_02093	2.4173177
2243601..224	-	305	CCNA_02094	3.70659692
2243801..224	+	341	CCNA_02095	2.56434051
2245101..224	-	122	CCNA_02096	3.26552744
2247651..224	+	298	CCNA_02098	2.58695965
2250201..225	-	471	CCNA_02099	2.11196198
2250601..225	+	128	CCNA_02101	3.01671904
2251001..225	+	347	CCNA_02102	4.39647376
2256951..225	-	73	CCNA_02107	5.26730263
2261051..226	-	142	CCNA_02110	2.03279554
2261351..226	-	184	CCNA_02111	4.27207008
2261501..226	+	333	CCNA_02112	3.88754791
2271101..227	-	330	CCNA_02120	2.64350696
2270901..227	+	859	CCNA_02121	2.53041233
2274601..227	+	164	CCNA_02123	3.19767107
2279251..227	-	974	CCNA_02125	6.28515535
2279301..227	+	71	CCNA_02126	6.96372314
2288151..228	-	821	CCNA_02131	2.03279554
2289101..228	-	339	CCNA_02132	5.48218285
2300101..230	-	374	CCNA_02140	4.30599826
2300601..230	-	205	CCNA_02141	6.36432077
2300651..230	+	245	CCNA_02142	4.06849994
2301201..230	+	262	CCNA_02143	2.43993683
2307551..230	-	640	CCNA_02149	2.76791064
2309551..230	-	219	CCNA_02152	2.46255493
2310101..231	-	306	CCNA_02153	2.66612609
2316351..231	-	169	CCNA_02160	3.75183415
2316201..231	+	222	CCNA_02161	6.10420332
2317301..231	-	73	CCNA_02162	3.84231068
2317251..231	+	286	CCNA_02163	2.79052978
2321251..232	-	194	CCNA_02166	2.94886267
2324851..232	-	426	CCNA_02171	2.13458112

2332851..233	-	366	CCNA_02180	2.60957878
2334351..233	-	307	CCNA_02182	2.11196198
2336101..233	+	601	CCNA_02185	2.56434051
2338851..233	+	162	CCNA_02187	5.25599358
2339851..233	+	257	CCNA_02189	2.2589848
2343851..234	-	146	CCNA_02192	3.42386034
2344401..234	-	205	CCNA_02193	2.17981835
2346901..234	-	132	CCNA_02196	2.67743515
2349651..234	-	130	CCNA_02200	2.85838718
2350351..235	-	186	CCNA_02201	3.07326636
2350201..235	+	265	CCNA_02202	2.46255493
2354401..235	-	318	CCNA_02205	5.27861271
2354351..235	+	187	CCNA_02206	3.01671904
2354601..235	+	316	CCNA_02207	6.33039259
2359401..235	+	303	CCNA_02212	4.05718986
2364751..236	-	100	CCNA_02217	2.4173177
2396001..239	-	278	CCNA_02246	3.38993112
2397351..239	-	50	CCNA_02248	2.85838718
2400701..240	-	279	CCNA_02251	3.20898012
2409001..240	-	489	CCNA_02256	4.38516471
2409151..240	+	241	CCNA_02258	5.34646908
2423901..242	-	169	CCNA_02270	2.02148649
2424951..242	-	80	CCNA_02272	2.51910225
2425251..242	-	82	CCNA_02273	3.69528683
2425801..242	-	161	CCNA_02274	2.04410562
2430851..243	+	593	CCNA_02281	2.65481601
2434001..243	-	187	CCNA_02283	3.68397778
2435801..243	+	215	CCNA_02287	2.12327104
2444801..244	-	193	CCNA_02297	2.05541467
2446151..244	-	343	CCNA_02298	2.17981835
2447951..244	+	236	CCNA_02302	3.57088315
2449651..244	-	181	CCNA_02303	2.68874523
2450101..245	+	281	CCNA_02305	3.94409523
2454501..245	+	111	CCNA_02309	2.15720025
2461901..246	-	100	CCNA_02318	3.48040765
2463301..246	+	394	CCNA_02321	2.11196198
2466851..246	-	302	CCNA_02324	4.15897544
2467851..246	-	311	CCNA_02325	2.47386501
2470251..247	-	273	CCNA_02327	3.41255025
2475451..247	-	314	CCNA_02333	2.0101764
2478401..247	-	102	CCNA_02337	2.06672372
2482701..248	-	421	CCNA_02342	3.23159925
2484201..248	-	419	CCNA_02343	2.85838718
2485001..248	+	77	CCNA_02345	3.85361973
2485151..248	+	318	CCNA_02346	6.91848488
2488401..248	+	299	CCNA_02349	4.24945094
2496501..249	-	322	CCNA_02356	2.57565059
2501701..250	-	383	CCNA_02361	4.15897544
2502551..250	-	164	CCNA_02362	2.43993683
2502351..250	+	109	CCNA_02363	2.29291298
2519951..252	-	88	CCNA_02374	2.92624354
2523351..252	-	118	CCNA_02380	2.73398246
2530801..253	-	407	CCNA_02386	3.67266873

2534201..253	-	462	CCNA_02389	6.33039259
2534251..253	+	237	CCNA_02390	4.37385463
2536401..253	+	145	CCNA_04015	4.63397311
2538201..253	-	220	CCNA_02394	3.20898012
2541151..254	-	331	CCNA_02398	4.27207008
2543451..254	+	350	CCNA_02401	2.55303146
2546201..254	-	559	CCNA_02402	3.01671904
2546151..254	+	371	CCNA_02403	4.91670969
2547351..254	+	278	CCNA_02404	2.48517406
2550651..255	-	254	CCNA_02407	2.85838718
2551851..255	-	377	CCNA_02408	2.58695965
2556001..255	-	288	CCNA_02411	5.25599358
2555851..255	+	240	CCNA_02412	4.82623419
2558151..255	-	387	CCNA_02413	2.38338951
2559551..255	-	338	CCNA_02414	2.39469856
2560101..256	+	92	CCNA_02416	2.28160393
2565101..256	-	144	CCNA_02420	2.3268422
2569301..256	-	57	CCNA_02425	4.01195263
2570301..257	-	353	CCNA_02426	2.92624354
2571101..257	-	76	CCNA_02428	3.66135865
2571351..257	-	152	CCNA_02429	3.43516939
2577101..257	-	429	CCNA_02435	3.26552744
2577551..257	-	156	CCNA_02436	2.22505662
2582301..258	-	346	CCNA_02440	3.76314423
2584601..258	-	500	CCNA_02442	2.06672372
2586401..258	+	245	CCNA_02445	3.45778852
2588501..258	-	387	CCNA_02446	3.37862207
2589101..258	-	252	CCNA_02447	3.57088315
2589551..258	+	257	CCNA_02449	6.4774154
2590601..259	+	143	CCNA_02450	3.4012412
2603901..260	-	153	CCNA_02460	2.99409991
2604001..260	+	105	CCNA_02462	3.24290831
2607601..260	-	435	CCNA_02464	2.5982687
2610151..261	-	435	CCNA_02465	5.38039726
2612001..261	+	198	CCNA_02469	2.02148649
2613251..261	+	421	CCNA_02471	4.06849994
2620751..262	-	244	CCNA_02475	3.88754791
2648351..264	+	104	CCNA_02499	2.42862675
2656151..265	-	46	CCNA_02506	3.02802912
2663401..266	-	233	CCNA_02512	2.96017173
2664851..266	-	512	CCNA_02513	2.48517406
2664701..266	+	246	CCNA_02514	2.54172138
2666651..266	+	197	CCNA_02517	2.03279554
2672301..267	+	164	CCNA_02523	2.64350696
2676451..267	+	218	CCNA_02528	2.3494603
2681301..268	+	899	CCNA_02535	2.39469856
2688051..268	-	340	CCNA_02538	2.10065293
2692001..269	+	55	CCNA_02543	2.92624354
2696151..269	-	454	CCNA_02546	3.45778852
2696351..269	-	125	CCNA_02547	2.11196198
2699701..269	+	776	CCNA_02553	5.42563553
2710751..271	-	533	CCNA_02562	2.66612609
2716601..271	+	842	CCNA_02567	7.26907886

2721251..272	+	444	CCNA_02570	4.38516471
2724601..272	+	436	CCNA_02572	2.31553212
2727551..272	-	104	CCNA_02575	2.1685093
2727901..272	+	252	CCNA_02576	3.898858
2730801..273	+	59	CCNA_02580	3.44647844
2738151..273	-	637	CCNA_02587	2.74529254
2739151..273	-	309	CCNA_02588	3.03933817
2739401..273	+	78	CCNA_02589	4.13635631
2739451..273	+	296	CCNA_02590	4.40778384
2742451..274	-	125	CCNA_02593	4.03457073
2744451..274	-	205	CCNA_02596	2.11196198
2762851..276	-	335	CCNA_02614	2.06672372
2773201..277	-	508	CCNA_02623	3.34469389
2774951..277	-	441	CCNA_02624	5.68575298
2776051..277	-	302	CCNA_02625	2.1685093
2778301..277	-	467	CCNA_02627	3.63873952
2781801..278	-	57	CCNA_02631	3.41255025
2804551..280	-	242	CCNA_02650	2.58695965
2811501..281	-	265	CCNA_02658	2.02148649
2812451..281	+	935	CCNA_02661	2.48517406
2818601..281	-	144	CCNA_02664	3.85361973
2818651..281	+	370	CCNA_02665	2.47386501
2822601..282	-	426	CCNA_02669	3.46909757
2828401..282	+	137	CCNA_02674	3.32207475
2830051..283	-	298	CCNA_02676	5.66313384
2830001..283	+	204	CCNA_02677	3.06195731
2832601..283	-	250	CCNA_02679	3.9214761
2837301..283	-	454	CCNA_02683	4.973257
2840301..284	+	426	CCNA_02687	2.43993683
2844301..284	+	230	CCNA_02691	2.55303146
2867251..286	+	132	CCNA_02706	5.05242345
2874951..287	+	693	CCNA_02715	3.14112376
2879901..287	+	204	CCNA_02719	7.41610167
2881551..288	+	993	CCNA_02721	5.61789661
2889901..288	-	176	CCNA_02726	4.39647376
2895051..289	-	68	CCNA_02732	2.06672372
2908301..290	-	213	CCNA_02745	2.20243748
2926451..292	-	95	CCNA_02764	6.08158419
2927201..292	-	92	CCNA_02766	2.5982687
2928451..292	-	418	CCNA_02767	2.60957878
2929551..292	-	327	CCNA_02768	5.85539493
2931501..293	-	451	CCNA_02770	3.18636099
2936751..293	+	483	CCNA_02779	6.73753388
2939001..293	+	198	CCNA_02781	6.91848488
2940401..294	-	161	CCNA_02782	3.19767107
2946101..294	+	205	CCNA_02790	2.81314891
2955301..295	-	585	CCNA_02794	3.12981367
2960701..296	-	196	CCNA_02803	4.14766536
2960651..296	+	137	CCNA_02805	2.85838718
2960751..296	+	412	CCNA_02806	2.63219791
2973951..297	-	123	CCNA_02816	2.58695965
2975101..297	+	629	CCNA_02820	2.1685093
2979851..297	-	284	CCNA_02822	2.98279086

2980801..298	+	340	CCNA_02826	2.70005428
2982201..298	+	88	CCNA_02827	2.88100528
2985601..298	+	422	CCNA_02832	3.08457644
2989551..298	-	209	CCNA_02835	4.74706774
2990601..299	-	186	CCNA_02837	3.17505194
3006951..300	-	456	CCNA_02852	3.10719454
3026151..302	-	442	CCNA_02877	2.23636567
3029051..302	-	567	CCNA_02880	2.36077038
3031001..303	-	133	CCNA_02882	2.12327104
3032901..303	-	541	CCNA_02883	2.0780338
3037951..303	-	168	CCNA_02889	2.03279554
3037651..303	+	34	CCNA_02890	3.17505194
3047001..304	+	502	CCNA_02896	2.21374757
3060301..306	+	249	CCNA_02904	2.11196198
3061101..306	+	125	CCNA_02905	2.02148649
3061301..306	+	364	CCNA_02906	3.38993112
3070001..307	-	108	CCNA_02911	2.06672372
3086401..308	-	92	CCNA_02924	3.43516939
3086701..308	+	388	CCNA_02925	2.30422307
3098951..309	+	140	CCNA_02939	2.63219791
3103851..310	-	169	CCNA_02943	3.15243281
3103901..310	+	246	CCNA_02944	3.93278618
3116401..311	-	123	CCNA_02957	2.43993683
3119001..311	+	75	CCNA_02962	2.42862675
3119801..311	+	348	CCNA_02964	3.23159925
3135901..313	-	394	CCNA_02979	2.11196198
3144101..314	+	164	CCNA_02991	2.93755259
3150201..315	-	1099	CCNA_02994	2.04410562
3153101..315	-	69	CCNA_02997	3.19767107
3153351..315	-	106	CCNA_02998	2.43993683
3155101..315	-	289	CCNA_03000	2.86969623
3160801..316	+	511	CCNA_03007	3.91016705
3174501..317	+	375	CCNA_03020	7.26907886
3180251..318	+	168	CCNA_03025	3.00540999
3184751..318	+	228	CCNA_03030	2.80183986
3187651..318	+	295	CCNA_03033	2.72267341
3195301..319	-	225	CCNA_03039	2.70005428
3196801..319	-	560	CCNA_03040	2.3268422
3197801..319	-	297	CCNA_03041	5.2446835
3207301..320	-	391	CCNA_03052	2.19112843
3209051..320	+	312	CCNA_03054	2.11196198
3215101..321	-	370	CCNA_03062	4.33992644
3219551..321	-	713	CCNA_03066	3.36731302
3222501..322	+	234	CCNA_03072	4.57742579
3240301..324	-	320	CCNA_03090	7.8006228
3240351..324	+	66	CCNA_03091	12.652384
3240751..324	+	458	CCNA_03092	3.57088315
3256651..325	-	216	CCNA_03105	2.4173177
3256901..325	+	167	CCNA_03107	3.15243281
3264451..326	-	259	CCNA_03113	2.47386501
3267301..326	-	79	CCNA_03118	2.77922073
3268351..326	-	205	CCNA_03119	5.34646908
3274701..327	+	412	CCNA_03127	2.22505662

3279601..327	-	231	CCNA_03130	2.22505662
3280951..328	-	267	CCNA_03131	3.41255025
3281201..328	-	108	CCNA_03132	4.13635631
3282701..328	+	444	CCNA_03135	2.1685093
3284101..328	+	139	CCNA_03136	2.28160393
3294501..329	-	653	CCNA_03142	2.45124588
3296351..329	-	589	CCNA_03143	3.19767107
3301201..330	+	597	CCNA_03148	2.08934285
3311051..331	+	209	CCNA_03155	2.28160393
3311551..331	+	171	CCNA_03156	3.3333838
3316651..331	+	351	CCNA_03161	2.40600762
3326851..332	-	284	CCNA_03167	3.26552744
3331851..333	-	850	CCNA_03171	3.88754791
3332501..333	+	158	CCNA_03173	2.4173177
3340201..334	-	243	CCNA_03180	2.63219791
3340401..334	+	424	CCNA_03181	2.4173177
3346001..334	-	591	CCNA_03184	2.84707709
3345851..334	+	208	CCNA_03185	2.86969623
3353951..335	-	748	CCNA_03191	2.79052978
3358501..335	-	269	CCNA_03198	4.44171202
3363051..336	-	428	CCNA_03201	3.10719454
3366101..336	+	557	CCNA_03205	2.56434051
3372951..337	+	317	CCNA_03210	3.07326636
3375401..337	+	102	CCNA_03213	2.4173177
3386901..338	-	89	CCNA_03224	2.03279554
3400951..340	-	381	CCNA_03235	2.1685093
3407351..340	-	466	CCNA_03241	3.97802341
3423501..342	+	449	CCNA_03253	2.0101764
3435251..343	-	67	CCNA_03264	2.97148181
3457801..345	-	158	CCNA_03290	2.48517406
3459101..345	-	332	CCNA_03291	3.94409523
3466601..346	+	411	CCNA_03296	2.96017173
3467951..346	+	371	CCNA_03297	2.66612609
3485151..348	+	358	CCNA_03309	4.23814189
3490851..349	+	627	CCNA_03316	2.86969623
3498751..349	-	266	CCNA_03319	4.70182947
3498601..349	+	1078	CCNA_03320	4.96194692
3508401..350	+	121	CCNA_03327	3.52564489
3509951..351	-	275	CCNA_03328	2.13458112
3511351..351	-	267	CCNA_03331	3.14112376
3512551..351	-	274	CCNA_03332	3.22028917
3516501..351	-	626	CCNA_03334	2.54172138
3519301..351	-	188	CCNA_03337	2.02148649
3521501..352	-	270	CCNA_03339	3.80838147
3526301..352	-	173	CCNA_03346	2.77922073
3529051..352	-	62	CCNA_03349	2.85838718
3528951..352	+	65	CCNA_03350	5.34646908
3535501..353	-	108	CCNA_03356	3.34469389
3538451..353	+	363	CCNA_03360	2.04410562
3542851..354	+	254	CCNA_03365	2.70005428
3545051..354	+	142	CCNA_03368	2.94886267
3547201..354	-	159	CCNA_03371	2.84707709
3566351..356	+	119	CCNA_03395	4.11373718

3566551..356	+	574	CCNA_03396	2.04410562
3569451..356	-	183	CCNA_03398	2.85838718
3573851..357	-	225	CCNA_03405	6.7262238
3573701..357	+	87	CCNA_03406	3.05064722
3576701..357	-	305	CCNA_03408	2.08934285
3581201..358	-	111	CCNA_03413	3.48040765
3585201..358	-	157	CCNA_03418	2.28160393
3590301..359	+	488	CCNA_03424	2.06672372
3596251..359	+	409	CCNA_03433	2.63219791
3597851..359	+	236	CCNA_03434	2.14589017
3605101..360	-	122	CCNA_03441	2.13458112
3605301..360	-	136	CCNA_03442	7.94764665
3608701..360	+	816	CCNA_03445	2.88100528
3613851..361	-	167	CCNA_03448	2.90362441
3616001..361	+	118	CCNA_03451	2.5982687
3624051..362	-	166	CCNA_03460	2.04410562
3628101..362	-	121	CCNA_03464	2.64350696
3629301..362	-	375	CCNA_03465	2.79052978
3629451..362	+	267	CCNA_03466	3.11850462
3637401..363	-	240	CCNA_04011	2.71136333
3637601..363	+	319	CCNA_03471	2.31553212
3638651..363	+	89	CCNA_03472	3.06195731
3648101..364	-	324	CCNA_03485	2.81314891
3649501..364	-	179	CCNA_03488	2.06672372
3652501..365	-	286	CCNA_03492	2.85838718
3654101..365	-	353	CCNA_03494	3.3333838
3660751..366	-	427	CCNA_03502	2.97148181
3661801..366	-	213	CCNA_03503	2.51910225
3663951..366	-	160	CCNA_03505	3.32207475
3663751..366	+	195	CCNA_03506	4.01195263
3667001..366	-	192	CCNA_03508	2.38338951
3672501..367	-	216	CCNA_03514	2.49648415
3672701..367	-	53	CCNA_03515	3.69528683
3673851..367	-	339	CCNA_03516	3.07326636
3676401..367	-	331	CCNA_03518	3.06195731
3687251..368	-	126	CCNA_03530	2.27029488
3688551..368	-	504	CCNA_03531	2.08934285
3688701..368	+	582	CCNA_03532	3.63873952
3695701..369	+	117	CCNA_03537	2.38338951
3697401..369	+	218	CCNA_03540	2.60957878
3698651..369	+	187	CCNA_03542	2.85838718
3702151..370	+	173	CCNA_03546	5.55003921
3710301..371	+	438	CCNA_03554	4.83754324
3714401..371	-	86	CCNA_03558	2.5982687
3714901..371	-	83	CCNA_03559	3.87623886
3720351..372	+	178	CCNA_03566	3.36731302
3730201..373	-	847	CCNA_03574	3.00540999
3740951..374	+	276	CCNA_03586	2.19112843
3744951..374	+	266	CCNA_03591	5.2220654
3750501..375	-	136	CCNA_03597	2.20243748
3750251..375	+	769	CCNA_03598	5.28992177
3754751..375	+	232	CCNA_03601	2.04410562
3764051..376	-	449	CCNA_03609	2.36077038

3786251..378	-	237	CCNA_03629	4.24945094
3786801..378	-	188	CCNA_03630	4.13635631
3798601..379	-	76	CCNA_03638	2.33815125
3799151..379	-	106	CCNA_03639	2.08934285
3800451..380	-	425	CCNA_03640	2.54172138
3804401..380	-	195	CCNA_03645	2.70005428
3805801..380	-	372	CCNA_03646	2.46255493
3806801..380	+	363	CCNA_03649	3.14112376
3808401..380	+	996	CCNA_03651	2.37207943
3819301..381	-	275	CCNA_03657	2.0101764
3834201..383	+	612	CCNA_03675	3.01671904
3835801..383	+	147	CCNA_03676	2.1685093
3843601..384	-	294	CCNA_03681	3.26552744
3843801..384	+	226	CCNA_03682	2.49648415
3854451..385	-	293	CCNA_03687	2.06672372
3854651..385	-	157	CCNA_03688	3.4012412
3854801..385	+	370	CCNA_03689	2.70005428
3865751..386	+	948	CCNA_03699	2.86969623
3869151..386	-	139	CCNA_03700	2.54172138
3871301..387	-	569	CCNA_03702	3.08457644
3876101..387	-	122	CCNA_03708	2.4173177
3876251..387	-	136	CCNA_03709	6.8166993
3877551..387	-	208	CCNA_03711	3.10719454
3878501..387	-	204	CCNA_03712	2.14589017
3880651..388	-	252	CCNA_03714	3.42386034
3881501..388	-	186	CCNA_03715	2.83576804
3882401..388	-	220	CCNA_03716	3.53695497
3882501..388	-	205	CCNA_03717	3.59350228
3884701..388	+	84	CCNA_03720	2.04410562
3895851..389	-	386	CCNA_03726	3.43516939
3897351..389	-	97	CCNA_03728	2.27029488
3899301..389	-	202	CCNA_03731	2.0780338
3906401..390	+	123	CCNA_03736	5.94587042
3910501..391	-	328	CCNA_03740	2.99409991
3911751..391	-	381	CCNA_03741	3.84231068
3911801..391	+	144	CCNA_03742	2.58695965
3913851..391	-	479	CCNA_03743	2.13458112
3915951..391	-	280	CCNA_03745	3.10719454
3918251..391	-	341	CCNA_03747	2.98279086
3917901..391	+	185	CCNA_03748	2.30422307
3918451..391	+	300	CCNA_03749	3.12981367
3930851..393	+	139	CCNA_03761	3.27683649
3934401..393	-	187	CCNA_03765	2.98279086
3935101..393	-	193	CCNA_03766	3.29945562
3937051..393	-	508	CCNA_03768	2.91493449
3938851..393	-	320	CCNA_03770	2.3494603
3945851..394	-	110	CCNA_03779	6.5452728
3950801..395	+	221	CCNA_03783	2.29291298
3954351..395	+	170	CCNA_03788	2.83576804
3955151..395	-	127	CCNA_03789	2.13458112
3955301..395	+	91	CCNA_03790	4.20421268
3959251..395	+	336	CCNA_03796	2.1685093
3966501..396	+	718	CCNA_03802	2.36077038

3968801..396	+	361	CCNA_03803	3.15243281
3970201..397	-	110	CCNA_03804	2.08934285
3971801..397	+	220	CCNA_03807	3.11850462
3972501..397	+	170	CCNA_03808	3.19767107
3972951..397	+	269	CCNA_03809	3.03933817
3976551..397	+	457	CCNA_03813	4.28337913
3981701..398	-	708	CCNA_03816	3.88754791
3981951..398	+	289	CCNA_03817	4.7244486
3986451..398	+	219	CCNA_03820	2.13458112
3992351..399	-	184	CCNA_03826	3.4917167
3994401..399	+	396	CCNA_03830	3.14112376
3997351..399	+	202	CCNA_03832	2.31553212
4002201..400	-	403	CCNA_03835	2.19112843
4003301..400	-	181	CCNA_03837	2.19112843
4013101..401	-	158	CCNA_03846	4.973257
4016851..401	+	244	CCNA_03852	3.27683649
4020101..402	-	189	CCNA_03855	2.83576804
4021551..402	-	234	CCNA_03857	3.63873952
4022851..402	-	232	CCNA_03859	2.06672372
4022801..402	+	169	CCNA_03860	3.03933817
4025401..402	-	150	CCNA_03863	3.00540999
4025351..402	+	234	CCNA_03864	4.41909289
4029151..402	+	350	CCNA_03867	3.50302678
4031451..403	-	304	CCNA_03868	2.90362441
4032601..403	-	267	CCNA_03869	2.14589017
4034801..403	-	640	CCNA_03871	3.7405251
4039851..403	-	477	CCNA_03876	3.61612142
4040651..404	-	171	CCNA_03877	2.14589017
4042801..404	-	363	CCNA_03879	2.19112843

Start..end	Strand	Product length	Gene code	Z-score coverage ChIP-seq m6A in Δ*ccrM*
851..900	+	199	CCNA_00002	2.66031876
1601..1650	+	285	CCNA_00003	3.01892589
3151..3200	+	237	CCNA_00005	2.36691293
3951..4000	+	262	CCNA_00006	2.04090645
4751..4800	+	96	CCNA_00007	2.10610775
10151..10200	+	385	CCNA_00011	2.75812071
11351..11400	+	903	CCNA_00012	2.62771811
17501..17550	-	158	CCNA_00014	2.0735071
17951..18000	-	183	CCNA_00015	2.39951358
19101..19150	-	79	CCNA_00017	4.32295182
19401..19450	+	210	CCNA_00019	2.98632524
20751..20800	-	74	CCNA_00020	2.8885233
23151..23200	-	730	CCNA_00021	2.823322
26601..26650	-	550	CCNA_00025	2.43211423
29551..29600	-	900	CCNA_00026	3.34493237
32851..32900	-	851	CCNA_00028	2.10610775
32901..32950	+	210	CCNA_00029	2.43211423
33301..33350	+	244	CCNA_00030	2.72552006
34701..34750	-	128	CCNA_00031	2.823322
35301..35350	-	80	CCNA_00032	2.0083058
38401..38450	-	89	CCNA_00034	2.43211423
39501..39550	-	310	CCNA_00035	2.46471487
40201..40250	-	149	CCNA_00036	2.17130904
41951..42000	-	172	CCNA_00039	2.59511747
42701..42750	-	189	CCNA_00040	2.56251682
46601..46650	-	216	CCNA_00042	2.20390969
48251..48300	-	548	CCNA_00043	2.9537246
50901..50950	-	226	CCNA_00047	2.8885233
53051..53100	-	144	CCNA_00049	2.0083058
54651..54700	-	530	CCNA_00050	3.44273432
55701..55750	-	157	CCNA_00052	2.52991617
58251..58300	-	448	CCNA_00054	2.33431228
59051..59100	-	150	CCNA_00056	2.59511747
60051..60100	-	211	CCNA_00057	3.83394209
61051..61100	-	195	CCNA_00059	2.36691293
68501..68550	-	598	CCNA_00064	2.52991617
72201..72250	-	367	CCNA_00069	2.49731552
73651..73700	+	304	CCNA_00072	2.04090645
78901..78950	-	733	CCNA_00074	2.98632524
79951..80000	-	401	CCNA_00075	2.26911099
80601..80650	-	127	CCNA_00076	4.25775052
81851..81900	-	391	CCNA_00077	3.99694533
85201..85250	-	69	CCNA_00081	2.36691293
88601..88650	-	546	CCNA_00083	2.10610775
90601..90650	+	252	CCNA_00085	2.23651034
97451..97500	+	516	CCNA_00088	3.08412719
102251..1023	-	809	CCNA_00089	2.36691293

104951..1050	-	296	CCNA_00092	2.39951358
107001..1070	+	292	CCNA_00096	2.46471487
111151..1112	-	576	CCNA_00099	2.04090645
116451..1165	+	421	CCNA_00106	3.01892589
118551..1186	-	65	CCNA_00107	3.44273432
119151..1192	-	225	CCNA_00108	2.46471487
120001..1200	-	299	CCNA_00109	2.62771811
122651..1227	+	1115	CCNA_00115	3.37753302
127851..1279	+	606	CCNA_00117	2.72552006
129451..1295	+	747	CCNA_00118	2.04090645
131651..1317	+	153	CCNA_00119	2.62771811
132101..1321	+	172	CCNA_00120	2.0083058
133851..1339	+	260	CCNA_00123	2.30171163
134201..1342	-	136	CCNA_00122	2.13870839
135451..1355	-	218	CCNA_00124	3.21452978
136151..1362	-	222	CCNA_00125	2.33431228
138451..1385	-	408	CCNA_00128	2.823322
139101..1391	-	120	CCNA_00129	2.23651034
142601..1426	+	404	CCNA_00133	3.01892589
143601..1436	+	142	CCNA_00134	2.62771811
144251..1443	+	277	CCNA_00135	2.43211423
154751..1548	-	271	CCNA_00141	2.10610775
158601..1586	+	177	CCNA_00148	2.13870839
166651..1667	+	821	CCNA_00159	2.0083058
169451..1695	+	379	CCNA_00160	2.33431228
174751..1748	+	641	CCNA_00164	2.10610775
179951..1800	+	203	CCNA_00168	3.05152654
182951..1830	+	888	CCNA_00170	2.33431228
185501..1855	+	145	CCNA_00171	2.46471487
190851..1909	+	150	CCNA_00175	2.75812071
193751..1938	+	368	CCNA_00180	3.50793561
194651..1947	+	158	CCNA_00181	3.11672784
197401..1974	-	65	CCNA_00184	2.52991617
200151..2002	-	841	CCNA_00185	2.0083058
202751..2028	-	374	CCNA_00187	2.43211423
204501..2045	+	158	CCNA_00191	2.13870839
207151..2072	-	539	CCNA_00192	2.04090645
208601..2086	-	100	CCNA_00194	2.52991617
209301..2093	-	202	CCNA_00195	2.72552006
212351..2124	+	626	CCNA_00199	4.6489583
215701..2157	-	346	CCNA_00200	2.04090645
217251..2173	+	347	CCNA_00203	2.20390969
221551..2216	+	213	CCNA_00207	2.0735071
225301..2253	-	524	CCNA_00209	2.30171163
228601..2286	+	292	CCNA_00212	2.23651034
236601..2366	-	169	CCNA_00218	3.21452978
236651..2367	+	105	CCNA_00219	3.89914339
236951..2370	+	201	CCNA_00220	2.8885233
239701..2397	+	260	CCNA_00223	3.01892589
244401..2444	+	324	CCNA_00229	2.04090645
247801..2478	+	345	CCNA_00233	2.43211423
248751..2488	+	386	CCNA_00234	3.37753302
250601..2506	-	91	CCNA_00235	2.59511747

252551..2526	+	235	CCNA_00237	2.66031876
255701..2557	+	92	CCNA_00241	2.85592265
257551..2576	+	94	CCNA_00245	2.75812071
257701..2577	+	261	CCNA_00246	2.69291941
261101..2611	-	466	CCNA_00248	2.75812071
261451..2615	+	427	CCNA_00250	2.39951358
270251..2703	-	283	CCNA_00256	2.0083058
273751..2738	+	347	CCNA_00261	2.79072136
273901..2739	-	236	CCNA_00260	3.34493237
274701..2747	+	290	CCNA_00262	2.20390969
277751..2778	-	417	CCNA_00264	2.04090645
284251..2843	+	200	CCNA_00270	2.26911099
286751..2868	+	173	CCNA_00273	2.26911099
287651..2877	+	120	CCNA_00274	2.59511747
289851..2899	+	386	CCNA_00277	2.59511747
294601..2946	+	110	CCNA_00282	2.33431228
296601..2966	-	221	CCNA_00284	3.18192913
297851..2979	-	304	CCNA_00285	2.04090645
298551..2986	-	152	CCNA_00286	2.8885233
307301..3073	+	431	CCNA_00293	2.10610775
309951..3100	+	230	CCNA_00296	2.92112395
314901..3149	-	197	CCNA_00300	2.39951358
323201..3232	-	607	CCNA_00307	2.8885233
324101..3241	-	77	CCNA_00309	2.0083058
325001..3250	-	259	CCNA_00310	2.20390969
326951..3270	-	133	CCNA_00312	2.33431228
328901..3289	-	609	CCNA_00313	2.62771811
329501..3295	-	203	CCNA_00314	2.52991617
330751..3308	-	509	CCNA_00315	2.20390969
333101..3331	+	345	CCNA_00318	2.26911099
340601..3406	+	229	CCNA_00328	2.33431228
340801..3408	-	220	CCNA_00327	2.66031876
340951..3410	+	182	CCNA_00329	2.0083058
343151..3432	+	224	CCNA_00332	2.36691293
345001..3450	+	186	CCNA_00334	2.26911099
347201..3472	+	257	CCNA_00337	2.20390969
353451..3535	+	294	CCNA_00341	2.9537246
357301..3573	+	402	CCNA_00343	3.01892589
360251..3603	-	329	CCNA_00345	2.33431228
361601..3616	+	59	CCNA_00347	4.583757
366001..3660	-	226	CCNA_00350	2.20390969
366051..3661	+	719	CCNA_00351	2.10610775
373401..3734	+	475	CCNA_00357	2.13870839
376851..3769	+	296	CCNA_00360	2.0083058
378751..3788	-	315	CCNA_00361	2.10610775
379651..3797	-	163	CCNA_00362	2.0735071
381601..3816	-	364	CCNA_00364	2.0735071
383051..3831	+	264	CCNA_00366	2.0083058
384501..3845	+	278	CCNA_00368	2.0083058
389251..3893	-	74	CCNA_00372	2.13870839
401551..4016	+	444	CCNA_00385	2.17130904
406001..4060	+	404	CCNA_00390	2.43211423
406151..4062	-	281	CCNA_00389	2.33431228

406951..4070	+	185	CCNA_00391	2.8885233
407801..4078	+	444	CCNA_00392	2.20390969
418451..4185	+	256	CCNA_00401	2.17130904
420301..4203	+	171	CCNA_00404	3.01892589
420951..4210	+	285	CCNA_00405	2.0735071
422701..4227	-	127	CCNA_00406	2.92112395
427551..4276	+	233	CCNA_00414	3.7687408
430251..4303	-	132	CCNA_00416	2.33431228
440101..4401	+	500	CCNA_00435	2.26911099
450551..4506	+	155	CCNA_00443	2.69291941
450951..4510	+	293	CCNA_00444	2.43211423
451851..4519	+	344	CCNA_00445	2.20390969
452901..4529	+	129	CCNA_00446	3.05152654
453001..4530	+	187	CCNA_00447	2.69291941
454051..4541	+	200	CCNA_00449	2.20390969
459901..4599	-	378	CCNA_00452	2.20390969
461001..4610	-	363	CCNA_00453	2.13870839
462151..4622	-	270	CCNA_00454	2.33431228
487201..4872	-	322	CCNA_00471	2.59511747
491751..4918	-	936	CCNA_00474	2.0735071
494201..4942	-	64	CCNA_00477	2.823322
495351..4954	+	107	CCNA_00479	2.46471487
502451..5025	-	342	CCNA_00485	2.43211423
507551..5076	-	208	CCNA_00488	2.79072136
508101..5081	-	139	CCNA_00489	2.0083058
509301..5093	+	463	CCNA_00492	2.23651034
509801..5098	-	212	CCNA_00491	2.0735071
512101..5121	+	84	CCNA_00495	2.20390969
519101..5191	+	154	CCNA_00503	2.79072136
520551..5206	+	426	CCNA_00506	2.26911099
527201..5272	-	366	CCNA_00512	2.26911099
528251..5283	-	266	CCNA_00513	3.08412719
532551..5326	-	249	CCNA_00516	2.59511747
534101..5341	-	197	CCNA_00518	2.49731552
536451..5365	-	83	CCNA_00521	2.0735071
542851..5429	+	127	CCNA_00531	2.26911099
561701..5617	-	107	CCNA_00542	2.46471487
562451..5625	+	391	CCNA_00544	2.04090645
569651..5697	-	156	CCNA_00552	2.33431228
571501..5715	+	505	CCNA_00556	2.98632524
573751..5738	-	259	CCNA_00557	2.39951358
575601..5756	+	170	CCNA_00560	2.04090645
586251..5863	-	770	CCNA_00567	2.30171163
588201..5882	-	358	CCNA_00569	2.36691293
589451..5895	-	301	CCNA_00570	2.17130904
598801..5988	+	372	CCNA_00575	2.0083058
610351..6104	-	383	CCNA_04005	2.13870839
626301..6263	-	606	CCNA_00594	2.98632524
660351..6604	+	347	CCNA_00617	2.20390969
660601..6606	-	162	CCNA_00616	2.66031876
664301..6643	+	392	CCNA_00620	2.04090645
667001..6670	+	735	CCNA_00623	2.39951358
676101..6761	+	764	CCNA_00630	2.04090645

678301..6783	+	150	CCNA_00631	2.98632524
690101..6901	-	902	CCNA_00638	2.17130904
692251..6923	-	91	CCNA_00640	2.13870839
695151..6952	+	430	CCNA_00645	2.49731552
697701..6977	+	261	CCNA_00647	3.34493237
710501..7105	+	630	CCNA_00656	2.23651034
719801..7198	-	124	CCNA_00662	2.36691293
729151..7292	-	250	CCNA_00670	2.39951358
734001..7340	-	79	CCNA_00676	2.13870839
734601..7346	-	229	CCNA_00677	2.20390969
735551..7356	+	311	CCNA_00679	2.79072136
738151..7382	-	245	CCNA_00681	2.39951358
745301..7453	+	776	CCNA_00689	2.52991617
757551..7576	-	113	CCNA_00697	2.10610775
783201..7832	-	435	CCNA_00726	2.9537246
799901..7999	-	152	CCNA_00741	2.17130904
800701..8007	+	188	CCNA_00743	2.26911099
810651..8107	-	438	CCNA_00751	2.66031876
820551..8206	-	566	CCNA_00760	2.26911099
822301..8223	-	313	CCNA_00763	2.39951358
846301..8463	+	407	CCNA_00784	2.56251682
850751..8508	-	108	CCNA_04003	2.49731552
851151..8512	+	450	CCNA_00788	2.0083058
871601..8716	-	615	CCNA_00806	2.39951358
886001..8860	-	242	CCNA_00824	4.29035117
890751..8908	+	504	CCNA_00828	2.79072136
894551..8946	+	983	CCNA_00831	2.39951358
906701..9067	+	826	CCNA_00839	2.0083058
941751..9418	-	591	CCNA_00862	2.13870839
970251..9703	+	754	CCNA_00892	2.04090645
974851..9749	+	131	CCNA_00895	2.23651034
995601..9956	-	88	CCNA_00917	2.33431228
1002651..100	-	180	CCNA_00925	2.23651034
1019301..101	-	307	CCNA_00942	2.75812071
1040951..104	-	232	CCNA_00965	2.17130904
1060601..106	+	234	CCNA_00981	2.13870839
1084051..108	-	303	CCNA_01001	2.39951358
1132851..113	-	261	CCNA_01037	2.49731552
1151151..115	+	240	CCNA_01052	2.62771811
1194701..119	-	175	CCNA_01088	2.62771811
1205351..120	-	396	CCNA_01099	2.13870839
1238301..123	-	176	CCNA_01133	2.0083058
1239601..123	-	127	CCNA_01135	2.10610775
1272201..127	+	2479	CCNA_01163	2.20390969
1302301..130	+	342	CCNA_01183	2.79072136
1305401..130	+	307	CCNA_01188	2.36691293
1315651..131	-	657	CCNA_01193	2.23651034
1406101..140	-	260	CCNA_01279	2.0083058
1407001..140	+	496	CCNA_01281	2.0735071
1416001..141	-	125	CCNA_01289	3.05152654
1438301..143	+	177	CCNA_01321	2.0083058
1463901..146	-	242	CCNA_01351	2.10610775
1532551..153	+	265	CCNA_01415	2.13870839

1576851..157	+	55	CCNA_01469	2.72552006
1586601..158	-	248	CCNA_01478	2.46471487
1752501..175	+	283	CCNA_01634	2.0735071
1773901..177	+	163	CCNA_01652	2.39951358
1805801..180	+	485	CCNA_01679	2.04090645
1850251..185	+	250	CCNA_01724	2.52991617
1915801..191	-	311	CCNA_01787	2.56251682
2337601..233	+	185	CCNA_02186	2.17130904
2396151..239	+	285	CCNA_02247	2.20390969
2519901..251	-	88	CCNA_02374	2.17130904
2534251..253	+	237	CCNA_02390	2.10610775
2538051..253	-	220	CCNA_02394	2.39951358
2589351..258	-	252	CCNA_02447	2.04090645
2607601..260	-	435	CCNA_02464	3.47533497
2663251..266	-	233	CCNA_02512	2.26911099
2677101..267	+	365	CCNA_02529	2.0735071
2688301..268	-	340	CCNA_02538	2.0083058
2696601..269	-	125	CCNA_02547	2.0083058
2721451..272	-	209	CCNA_02569	2.56251682
2880601..288	+	362	CCNA_02720	2.17130904
2890701..289	+	101	CCNA_02728	2.04090645
2921001..292	+	239	CCNA_02757	2.33431228
2926951..292	-	127	CCNA_02765	2.0083058
2960001..296	+	66	CCNA_02804	2.0083058
2980351..298	+	130	CCNA_02825	2.0735071
2997301..299	-	100	CCNA_02843	2.46471487
2997601..299	-	72	CCNA_02844	2.0083058
3010451..301	-	60	CCNA_02857	2.52991617
3064701..306	-	241	CCNA_02908	2.0083058
3085851..308	-	1024	CCNA_02923	2.49731552
3088651..308	+	245	CCNA_02927	2.20390969
3099001..309	+	140	CCNA_02939	2.30171163
3128501..312	+	117	CCNA_02973	2.10610775
3138401..313	+	330	CCNA_02983	2.0735071
3160601..316	+	511	CCNA_03007	2.17130904
3165401..316	-	169	CCNA_03011	2.0083058
3169151..316	-	167	CCNA_03016	2.23651034
3189101..318	-	64	CCNA_03034	2.17130904
3206301..320	-	132	CCNA_03051	3.05152654
3233051..323	-	574	CCNA_03083	2.13870839
3241801..324	+	158	CCNA_03093	2.30171163
3245351..324	-	341	CCNA_03095	2.75812071
3261651..326	-	122	CCNA_03110	2.0083058
3268101..326	-	205	CCNA_03119	2.23651034
3269501..326	-	225	CCNA_03122	2.13870839
3282701..328	+	444	CCNA_03135	2.9537246
3329601..332	-	173	CCNA_03170	3.21452978
3333151..333	+	462	CCNA_03174	2.36691293
3335501..333	-	96	CCNA_03176	2.26911099
3342401..334	-	123	CCNA_03182	3.24713043
3345551..334	+	208	CCNA_03185	2.26911099
3363501..336	+	325	CCNA_03203	2.26911099
3363951..336	-	208	CCNA_03202	2.20390969

3379801..337	+	1022	CCNA_03219	3.31233172
3384351..338	+	404	CCNA_03222	2.13870839
3385701..338	+	202	CCNA_03223	2.13870839
3388551..338	-	431	CCNA_03225	2.52991617
3408751..340	-	485	CCNA_03242	2.52991617
3411251..341	-	223	CCNA_03244	2.36691293
3425001..342	+	451	CCNA_03254	2.36691293
3426751..342	+	372	CCNA_03256	2.39951358
3431951..343	+	937	CCNA_03263	2.13870839
3435401..343	-	67	CCNA_03264	2.0083058
3438101..343	+	325	CCNA_03269	2.72552006
3440851..344	-	401	CCNA_03271	2.10610775
3441101..344	+	473	CCNA_03272	2.59511747
3446451..344	-	372	CCNA_03278	2.75812071
3457851..345	-	158	CCNA_03290	2.0083058
3460951..346	-	524	CCNA_03292	2.52991617
3464151..346	+	642	CCNA_03295	2.0083058
3466551..346	+	411	CCNA_03296	2.26911099
3479801..347	-	327	CCNA_03302	2.30171163
3481201..348	-	396	CCNA_03303	2.26911099
3487001..348	-	185	CCNA_03310	2.36691293
3489451..348	+	408	CCNA_03315	2.0083058
3512551..351	-	274	CCNA_03332	2.30171163
3514701..351	-	797	CCNA_03333	2.36691293
3520701..352	-	443	CCNA_03338	2.43211423
3522651..352	-	232	CCNA_03341	2.39951358
3526351..352	-	173	CCNA_03346	2.0083058
3529451..352	-	62	CCNA_03349	2.0735071
3532101..353	-	277	CCNA_03353	2.30171163
3538301..353	+	363	CCNA_03360	2.0083058
3544601..354	+	166	CCNA_03367	2.13870839
3544901..354	+	142	CCNA_03368	2.23651034
3545801..354	+	173	CCNA_03370	2.66031876
3550951..355	-	151	CCNA_03377	2.36691293
3565751..356	-	192	CCNA_03393	2.20390969
3569301..356	-	139	CCNA_03397	3.60573756
3569451..356	-	183	CCNA_03398	5.04016607
3572651..357	+	169	CCNA_03404	2.20390969
3575801..357	-	476	CCNA_03407	2.04090645
3576651..357	-	305	CCNA_03408	2.46471487
3581451..358	-	111	CCNA_03413	2.30171163
3584101..358	-	333	CCNA_03416	2.04090645
3590151..359	-	129	CCNA_03423	2.30171163
3590201..359	+	488	CCNA_03424	2.39951358
3593551..359	-	89	CCNA_03428	2.23651034
3604451..360	-	330	CCNA_03440	2.26911099
3622001..362	-	161	CCNA_03458	3.37753302
3624901..362	-	139	CCNA_03461	2.10610775
3633151..363	+	597	CCNA_03468	2.85592265
3637801..363	-	240	CCNA_04011	2.46471487
3638551..363	+	89	CCNA_03472	3.24713043
3639701..363	-	75	CCNA_03474	2.0735071
3640451..364	+	149	CCNA_03476	3.05152654

3646851..364	-	314	CCNA_03483	2.04090645
3648001..364	-	324	CCNA_03485	2.9537246
3651601..365	-	260	CCNA_03491	2.26911099
3653401..365	-	143	CCNA_03493	3.11672784
3654351..365	-	353	CCNA_03494	2.98632524
3655701..365	+	150	CCNA_03497	2.52991617
3658501..365	-	469	CCNA_03500	2.85592265
3660851..366	-	427	CCNA_03502	3.14932848
3661251..366	+	603	CCNA_03504	3.31233172
3661401..366	-	213	CCNA_03503	2.23651034
3663701..366	+	195	CCNA_03506	3.01892589
3668351..366	-	423	CCNA_03509	2.23651034
3671151..367	-	120	CCNA_03512	2.17130904
3671851..367	-	290	CCNA_03513	2.04090645
3672501..367	-	216	CCNA_03514	3.86654274
3675751..367	-	552	CCNA_03517	2.17130904
3677651..367	+	278	CCNA_03521	2.62771811
3678601..367	+	490	CCNA_03522	2.98632524
3682901..368	-	626	CCNA_03524	2.0083058
3684701..368	-	453	CCNA_03527	3.57313691
3685751..368	+	253	CCNA_03529	2.23651034
3692851..369	+	328	CCNA_03534	3.31233172
3697001..369	-	289	CCNA_03538	2.23651034
3697051..369	+	120	CCNA_03539	2.17130904
3697201..369	+	218	CCNA_03540	2.23651034
3698151..369	+	279	CCNA_03541	2.62771811
3698751..369	+	187	CCNA_03542	2.85592265
3702201..370	+	173	CCNA_03546	2.20390969
3709601..370	+	172	CCNA_03553	3.24713043
3710351..371	+	438	CCNA_03554	3.44273432
3713101..371	-	110	CCNA_03556	2.17130904
3714501..371	-	83	CCNA_03559	2.0083058
3717101..371	-	291	CCNA_03561	2.39951358
3720651..372	+	435	CCNA_03567	2.23651034
3721901..372	+	254	CCNA_03568	2.17130904
3722851..372	+	306	CCNA_03569	2.13870839
3725151..372	+	143	CCNA_03572	2.39951358
3730301..373	-	847	CCNA_03574	2.43211423
3736851..373	+	102	CCNA_03579	2.26911099
3741051..374	+	276	CCNA_03586	2.75812071
3742501..374	-	47	CCNA_03587	2.43211423
3746901..374	-	203	CCNA_03592	2.85592265
3749801..374	-	135	CCNA_03596	2.20390969
3754101..375	+	256	CCNA_03600	2.49731552
3760651..376	-	41	CCNA_03606	2.46471487
3764101..376	-	449	CCNA_03609	2.10610775
3767751..376	+	150	CCNA_03613	2.20390969
3770551..377	+	470	CCNA_03616	2.10610775
3777801..377	+	401	CCNA_03623	2.72552006
3787901..378	+	123	CCNA_03633	2.62771811
3790501..379	+	509	CCNA_03637	2.30171163
3798601..379	-	76	CCNA_03638	2.33431228
3799201..379	-	106	CCNA_03639	2.62771811

3801101..380	-	260	CCNA_03641	2.20390969
3803701..380	-	135	CCNA_03644	2.04090645
3804451..380	-	195	CCNA_03645	3.44273432
3815451..381	+	127	CCNA_03653	2.72552006
3819251..381	-	275	CCNA_03657	2.26911099
3820501..382	-	410	CCNA_03658	2.04090645
3825751..382	+	1079	CCNA_03665	2.13870839
3829901..382	-	62	CCNA_03668	2.36691293
3830801..383	+	286	CCNA_03670	2.13870839
3833051..383	-	206	CCNA_03672	3.21452978
3838251..383	-	433	CCNA_03677	2.20390969
3839451..383	-	142	CCNA_03679	2.75812071
3843051..384	-	1195	CCNA_03680	2.52991617
3845101..384	+	222	CCNA_03684	2.75812071
3854501..385	-	293	CCNA_03687	2.92112395
3854751..385	-	157	CCNA_03688	2.17130904
3859801..385	-	251	CCNA_03693	2.33431228
3869101..386	-	139	CCNA_03700	2.8885233
3871301..387	-	569	CCNA_03702	3.08412719
3872551..387	-	213	CCNA_03703	2.9537246
3878351..387	-	204	CCNA_03712	2.0083058
3879751..387	-	497	CCNA_03713	2.85592265
3880651..388	-	252	CCNA_03714	2.26911099
3881501..388	-	186	CCNA_03715	2.52991617
3892701..389	+	541	CCNA_03725	2.36691293
3897151..389	-	97	CCNA_03728	2.36691293
3899301..389	-	202	CCNA_03731	2.36691293
3899351..389	+	390	CCNA_03732	2.23651034
3902051..390	-	436	CCNA_03733	2.0083058
3904001..390	-	678	CCNA_03734	2.72552006
3906901..390	+	98	CCNA_03737	3.27973108
3911951..391	-	381	CCNA_03741	2.20390969
3914701..391	-	356	CCNA_03744	2.79072136
3915501..391	-	280	CCNA_03745	2.62771811
3917901..391	+	185	CCNA_03748	2.20390969
3918051..391	-	341	CCNA_03747	2.62771811
3918501..391	+	300	CCNA_03749	3.01892589
3919401..391	+	417	CCNA_03750	2.43211423
3921701..392	+	298	CCNA_03752	2.26911099
3921901..392	-	177	CCNA_03751	2.59511747
3924751..392	-	232	CCNA_03754	3.05152654
3927101..392	-	309	CCNA_03756	2.39951358
3931251..393	+	32	CCNA_03762	2.20390969
3932451..393	+	284	CCNA_03764	2.39951358
3934401..393	-	187	CCNA_03765	6.18118876
3935151..393	-	193	CCNA_03766	2.56251682
3935301..393	-	165	CCNA_03767	2.30171163
3937901..393	-	193	CCNA_03769	2.92112395
3938701..393	+	348	CCNA_03771	2.26911099
3939601..393	+	141	CCNA_03772	3.34493237
3941701..394	-	391	CCNA_03773	2.72552006
3944501..394	-	350	CCNA_03777	2.20390969
3955101..395	-	127	CCNA_03789	2.10610775

3956901..395	-	206	CCNA_03792	2.36691293
3957551..395	+	229	CCNA_03794	2.52991617
3961451..396	-	143	CCNA_03797	2.0083058
3964551..396	-	288	CCNA_03800	2.0735071
3970401..397	-	110	CCNA_03804	2.20390969
3972551..397	+	170	CCNA_03808	3.18192913
3972901..397	+	269	CCNA_03809	2.13870839
3974001..397	+	256	CCNA_03810	2.26911099
3976651..397	+	457	CCNA_03813	2.49731552
3979951..398	-	322	CCNA_03815	2.66031876
3981501..398	+	289	CCNA_03817	2.17130904
3981801..398	-	708	CCNA_03816	2.13870839
3983151..398	-	116	CCNA_03818	3.01892589
3986051..398	+	219	CCNA_03820	2.36691293
3992001..399	-	120	CCNA_03825	2.9537246
3993651..399	+	155	CCNA_03828	3.44273432
3994751..399	-	139	CCNA_03829	2.79072136
3995501..399	+	533	CCNA_03831	2.13870839
3996951..399	+	202	CCNA_03832	2.04090645
3998751..399	-	278	CCNA_03833	3.24713043
4000701..400	-	596	CCNA_03834	2.33431228
4001951..400	-	403	CCNA_03835	2.39951358
4003451..400	-	181	CCNA_03837	2.56251682
4003951..400	+	667	CCNA_03839	2.39951358
4009151..400	-	319	CCNA_03841	2.39951358
4009851..400	-	256	CCNA_03842	2.43211423
4010201..401	+	430	CCNA_03844	3.63833821
4013401..401	-	158	CCNA_03846	3.41013367
4015001..401	-	309	CCNA_03848	3.08412719
4016551..401	+	213	CCNA_03851	2.66031876
4016901..401	+	244	CCNA_03852	2.59511747
4018301..401	+	107	CCNA_03854	3.34493237
4022001..402	-	234	CCNA_03857	2.0083058
4024201..402	-	217	CCNA_03861	2.13870839
4024951..402	+	234	CCNA_03864	2.46471487
4028651..402	+	166	CCNA_03866	2.20390969
4029051..402	+	350	CCNA_03867	2.823322
4031701..403	-	304	CCNA_03868	2.92112395
4032251..403	-	267	CCNA_03869	2.8885233
4036901..403	-	85	CCNA_03873	3.73614015
4037151..403	+	321	CCNA_03875	3.7687408
4039751..403	-	477	CCNA_03876	2.30171163
4040551..404	-	171	CCNA_03877	2.49731552
4041651..404	-	347	CCNA_03878	2.10610775
