# Supplementary material for: DNA Binding of the Cell Cycle Transcriptional Regulator GcrA Depends on N6-Adenosine Methylation in Caulobacter crescentus and Other Alphaproteobacteria
Source: PLoS Genet. 2013 May 30;9(5):e1003541. doi: 10.1371/journal.pgen.1003541 (PMC3667746; doi:10.1371/journal.pgen.1003541)
Supplement: Table S4 — Transcription of lacZ by the tipF promoter in different genetic backgrounds. (PDF) [file pgen.1003541.s016.pdf]

TABLE S4

**Promoter fused with *lacZ***	**Activity**
tipF in WT (Xylose 5h)	101.8 ± 4.2
tipF in Δ*gcrA* Pxyl-*gcrA* (Xylose 5h)	89.1 ± 5.9
tipF in WT(Glucose 5h)	103.5 ± 4.5
tipF in Δ*gcrA* Pxyl-*gcrA* (Glucose 5h)	45.8 ± 5.2
tipF promoter WT	101.8 ± 2.5
tipF promoter GA*NTC	60.8 ± 2.7

* = mutation A to C
