# Supplementary material for: DNA Binding of the Cell Cycle Transcriptional Regulator GcrA Depends on N6-Adenosine Methylation in Caulobacter crescentus and Other Alphaproteobacteria
Source: PLoS Genet. 2013 May 30;9(5):e1003541. doi: 10.1371/journal.pgen.1003541 (PMC3667746; doi:10.1371/journal.pgen.1003541)
Supplement: Table S5 — Strains and plasmids. (PDF) [file pgen.1003541.s017.pdf]

TABLE S5

Strains

Name	Organism or plasmid	Description	Source
NA1000	*Caulobacter crescentus*	Reference strain	/
Rm1021	*Sinorhizobium meliloti*	Reference strain	/
2308	*Brucella melitensis* biovar *abortus*	Reference strain	Xavier de Bolle
CB15N	*Caulobacter crescentus*	Reference strain	/
gcrAts	*Caulobacter crescentus*	GcrA temperature (37) sensitive (mutation T10P), grow at 30C	Lucy Shapiro
EB510	*Caulobacter crescentus*	CB15N + pMR20 (TetR)	This study
EB517	*Caulobacter crescentus*	gcrAts + pMR20 (TetR)	This study
EB514	*Caulobacter crescentus*	CB15N + pMR20-GcrA(S. meliloti) (TetR)	This study
EB519	*Caulobacter crescentus*	gcrAts + pMR20-GcrA(S. meliloti) (TetR)	This study
EB516	*Caulobacter crescentus*	CB15N + pMR20-GcrA(B. abortus) (TetR)	This study
EB521	*Caulobacter crescentus*	gcrAts + pMR20-GcrA(B. abortus) (TetR)	This study
EB516	*Caulobacter crescentus*	CB15N + pMR20-GcrA(C. crescentus) (TetR)	This study
EB523	*Caulobacter crescentus*	gcrAts + pMR20-GcrA(C. crescentus) (TetR)	This study
BL21(D3E)	*Escherichia coli*	Expression strain (induction by IPTG)	/
UG2212	*Caulobacter crescentus*	CB15N Δ*ccrM::*Ω(SpecR), transduced from LS2144	This study
LS3707	*Caulobacter crescentus*	CB15N Δ*gcrA::*Ω(SpecR) P_{xyl}-*gcrA* (KanR)	Holtzendorff *et al*., 2004
LS2144	*Caulobacter crescentus*	CB15N Δ*ccrM::*Ω(SpecR) P_{xyl}-*ccrM* (TetR)	Stephens *et al*., 1996

Plasmids

Name	Organism or plasmid	Description	Source
EB343	pMR20 (TetR)	Low copy vector tetR	R. Roberts
EB494	pMR20-Pxyl-GcrA(S. mel) (TetR)	Low copy vector tetR expressing *S. meliloti gcrA* by a xylose inducible promoter	This study
EB498	pMR20-Pxyl-GcrA(B. abor) (TetR)	Low copy vector tetR expressing *B. abortus gcrA* by a xylose inducible promoter	This study
EB502	pMR20-Pxyl-GcrA(C. cres) (TetR)	Low copy vector tetR expressing *C. crescentus gcrA* by a xylose inducible promoter	This study
EB335	pET-His6-GcrA(Cc)	*E. coli* IPTG-inducible vector expressing Cc *gcrA*	This study
EB488	pET-His6-GcrA(Sm)	*E. coli* IPTG-inducible vector expressing Sm *gcrA*	This study
EB490	pET-His6-GcrA(Ba)	*E. coli* IPTG-inducible vector expressing Ba *gcrA*	This study
LT375	P_{mipZ}-*lacZ*	*lacZ* gene under the control of the wild-type *mipZ* promoter in p*lac290*	This study
LT376	P_{podJ}-*lacZ*	*lacZ* gene under the control of the wild-type *podJ* promoter in p*lac290*	This study
LT377	P_{flaY}-*lacZ*	*lacZ* gene under the control of the wild-type *flaY* promoter in p*lac290*	This study
LT378	P_{pleC}-*lacZ*	*lacZ* gene under the control of the wild-type *pleC* promoter in p*lac290*	This study
LT379	P_{CCNA_0697}-*lacZ*	*lacZ* gene under the control of the wild-type CCNA_0697 promoter in p*lac290*	This study
LT432	P_{tipF}-*lacZ*	*lacZ* gene under the control of the wild-type *tipF* promoter in p*lac290*	This study
LT380	P_{mipZ}-*lacZ* mut	*lacZ* gene under the control of the GANTC-mutant *mipZ* promoter in p*lac290*	This study
LT381	P_{podJ}-*lacZ* mut	*lacZ* gene under the control of the GANTCmutant *podJ* promoter in p*lac290*	This study
LT382	P_{flaY}-*lacZ* mut	*lacZ* gene under the control of the GANTCmutant *flaY* promoter in p*lac290*	This study
LT383	P_{pleC}-*lacZ* mut	*lacZ* gene under the control of the GANTCmutant *pleC* promoter in p*lac290*	This study
LT384	P_{CCNA_0697}-*lacZ* mut	*lacZ* gene under the control of the GANTCmutant CCNA_0697 promoter in p*lac290*	This study
LT433	P_{tipF}-*lacZ* mut	*lacZ* gene under the control of the GANTCmutant *tipF* promoter in p*lac290*	This study
