# Supplementary material for: DNA Binding of the Cell Cycle Transcriptional Regulator GcrA Depends on N6-Adenosine Methylation in Caulobacter crescentus and Other Alphaproteobacteria
Source: PLoS Genet. 2013 May 30;9(5):e1003541. doi: 10.1371/journal.pgen.1003541 (PMC3667746; doi:10.1371/journal.pgen.1003541)
Supplement: Table S6 — Log2 ratios of Figure 7A. (PDF) [file pgen.1003541.s018.pdf]

Gene	Normalized coverage 100-300 GcrA wt	Normalized coverage 100-300 GcrA delccrM	log2(RATIO)	Z-score(log2RATIO)
CCNA_00001	1.9312E-04	2.8189E-04	-5.4568E-01	-5.6341E-01
CCNA_00002	7.3644E-05	9.5168E-05	-3.6996E-01	-4.5132E-01
CCNA_00003	9.3250E-05	1.6158E-04	-7.9308E-01	-7.2121E-01
CCNA_00004	7.9475E-05	9.5384E-05	-2.6329E-01	-3.8329E-01
CCNA_00005	1.6732E-04	1.6344E-04	3.3873E-02	-1.9375E-01
CCNA_00006	9.8059E-05	1.4510E-04	-5.6532E-01	-5.7594E-01
CCNA_00007	5.0057E-04	4.9199E-04	2.4959E-02	-1.9943E-01
CCNA_00008	2.8533E-04	1.7670E-04	6.9132E-01	2.2560E-01
CCNA_00009	1.0134E-04	1.6886E-04	-7.3664E-01	-6.8521E-01
CCNA_00010	1.0346E-04	1.6243E-04	-6.5072E-01	-6.3041E-01
CCNA_00011	9.9511E-05	7.7418E-05	3.6211E-01	1.5616E-02
CCNA_00012	1.7340E-04	1.5751E-04	1.3857E-01	-1.2697E-01
CCNA_00013	1.0126E-04	1.0133E-04	-9.3727E-04	-2.1595E-01
CCNA_00014	7.8231E-05	6.2841E-05	3.1593E-01	-1.3836E-02
CCNA_00015	1.0416E-04	1.5442E-04	-5.6804E-01	-5.7767E-01
CCNA_00016	5.6026E-05	5.3924E-05	5.5067E-02	-1.8023E-01
CCNA_00017	7.0578E-05	1.2598E-04	-8.3590E-01	-7.4853E-01
CCNA_00018	1.4661E-04	1.6155E-04	-1.3998E-01	-3.0464E-01
CCNA_00019	1.7686E-04	1.6086E-04	1.3676E-01	-1.2812E-01
CCNA_00020	4.7495E-04	1.6187E-04	1.5529E+00	7.7513E-01
CCNA_00021	7.4263E-05	9.5724E-05	-3.6626E-01	-4.4897E-01
CCNA_00022	2.4266E-04	2.0144E-04	2.6857E-01	-4.4046E-02
CCNA_00023	1.7982E-04	1.6214E-04	1.4930E-01	-1.2012E-01
CCNA_00024	1.1387E-04	6.1001E-05	9.0039E-01	3.5896E-01
CCNA_00025	1.5101E-04	8.7138E-05	7.9317E-01	2.9057E-01
CCNA_00026	1.2540E-04	1.0970E-04	1.9294E-01	-9.2287E-02
CCNA_00027	3.3472E-04	1.5366E-04	1.1232E+00	5.0106E-01
CCNA_00028	2.1079E-04	1.6247E-04	3.7558E-01	2.4212E-02
CCNA_00029	2.7816E-04	2.6645E-04	6.2007E-02	-1.7580E-01
CCNA_00030	8.0659E-05	1.6092E-04	-9.9642E-01	-8.5091E-01
CCNA_00031	1.4627E-04	1.0525E-04	4.7470E-01	8.7429E-02
CCNA_00032	1.1849E-04	1.0270E-04	2.0623E-01	-8.3809E-02
CCNA_00033	1.1100E-04	1.0265E-04	1.1277E-01	-1.4342E-01
CCNA_00034	3.0348E-04	3.5480E-04	-2.2544E-01	-3.5915E-01
CCNA_00035	8.4413E-05	1.5813E-04	-9.0552E-01	-7.9293E-01
CCNA_00036	9.1493E-05	1.6243E-04	-8.2805E-01	-7.4352E-01
CCNA_00037	1.0512E-04	1.6243E-04	-6.2776E-01	-6.1576E-01
CCNA_00038	6.9237E-05	1.5884E-04	-1.1979E+00	-9.7944E-01
CCNA_00039	1.5468E-04	1.7540E-04	-1.8145E-01	-3.3109E-01
CCNA_00040	9.7948E-05	3.9405E-05	1.3134E+00	6.2239E-01
CCNA_00041	2.2381E-04	1.5291E-04	5.4953E-01	1.3516E-01
CCNA_00042	7.0425E-05	8.1727E-05	-2.1477E-01	-3.5234E-01
CCNA_00043	9.5206E-05	1.4882E-04	-6.4444E-01	-6.2640E-01
CCNA_00044	6.1831E-04	3.4280E-04	8.5094E-01	3.2741E-01
CCNA_00045	6.0250E-04	3.2861E-04	8.7456E-01	3.4248E-01
CCNA_00046	8.7618E-05	1.3841E-04	-6.5967E-01	-6.3611E-01
CCNA_00047	7.0333E-05	8.5920E-05	-2.8884E-01	-3.9959E-01
CCNA_00048	3.5867E-04	1.6248E-04	1.1424E+00	5.1332E-01
CCNA_00049	1.6753E-04	1.4482E-04	2.1013E-01	-8.1322E-02
CCNA_00050	6.5098E-05	3.2568E-05	9.9890E-01	4.2179E-01

CCNA_00051	1.4874E-04	1.1802E-04	3.3369E-01	-2.5078E-03
CCNA_00052	1.1032E-04	1.0364E-04	9.0058E-02	-1.5791E-01
CCNA_00053	7.2552E-05	1.6276E-04	-1.1656E+00	-9.5883E-01
CCNA_00054	9.0998E-05	1.1213E-04	-3.0132E-01	-4.0755E-01
CCNA_00055	4.6685E-04	2.0103E-04	1.2155E+00	5.5995E-01
CCNA_00056	7.0920E-05	6.4225E-05	1.4298E-01	-1.2416E-01
CCNA_00057	9.4290E-05	1.5758E-04	-7.4089E-01	-6.8792E-01
CCNA_00058	9.8748E-05	1.4951E-04	-5.9848E-01	-5.9709E-01
CCNA_00059	8.7928E-05	7.2056E-05	2.8711E-01	-3.2221E-02
CCNA_00060	1.5430E-04	1.3521E-04	1.9045E-01	-9.3873E-02
CCNA_00061	1.6695E-04	1.4041E-04	2.4974E-01	-5.6054E-02
CCNA_00062	1.8447E-04	1.4998E-04	2.9862E-01	-2.4880E-02
CCNA_00063	1.3435E-04	1.8769E-04	-4.8240E-01	-5.2305E-01
CCNA_00064	1.0342E-04	2.5019E-05	2.0471E+00	1.0904E+00
CCNA_00065	8.1093E-05	1.6279E-04	-1.0054E+00	-8.5661E-01
CCNA_00066	6.6019E-05	1.0681E-04	-6.9414E-01	-6.5810E-01
CCNA_00067	6.3767E-05	1.0883E-04	-7.7115E-01	-7.0722E-01
CCNA_00068	6.4571E-05	1.4041E-04	-1.1206E+00	-9.3014E-01
CCNA_00069	2.0178E-04	1.7715E-04	1.8775E-01	-9.5600E-02
CCNA_00070	2.1758E-04	1.6354E-04	4.1191E-01	4.7383E-02
CCNA_00071	8.2490E-05	8.5365E-05	-4.9489E-02	-2.4692E-01
CCNA_00072	1.6020E-04	1.0223E-04	6.4802E-01	1.9798E-01
CCNA_00073	1.0244E-04	9.8011E-05	6.3681E-02	-1.7473E-01
CCNA_00074	9.8859E-05	1.3703E-04	-4.7103E-01	-5.1579E-01
CCNA_00075	8.3956E-05	7.8901E-05	8.9510E-02	-1.5826E-01
CCNA_00076	9.0919E-05	1.1473E-04	-3.3556E-01	-4.2939E-01
CCNA_00077	8.9828E-05	9.4373E-05	-7.1258E-02	-2.6080E-01
CCNA_00078	6.4830E-05	1.2801E-04	-9.8151E-01	-8.4140E-01
CCNA_00079	4.7923E-04	4.8728E-04	-2.4025E-02	-2.3068E-01
CCNA_00080	4.7922E-04	4.8728E-04	-2.4067E-02	-2.3070E-01
CCNA_00081	2.0265E-04	1.6243E-04	3.1918E-01	-1.1765E-02
CCNA_00082	8.9370E-05	1.0453E-04	-2.2613E-01	-3.5959E-01
CCNA_00083	8.9689E-05	1.0162E-04	-1.8016E-01	-3.3026E-01
CCNA_00084	9.3287E-05	1.6712E-04	-8.4111E-01	-7.5185E-01
CCNA_00085	9.3458E-05	1.6243E-04	-7.9739E-01	-7.2396E-01
CCNA_00086	2.6438E-04	1.4134E-04	9.0345E-01	3.6091E-01
CCNA_00087	1.6902E-04	8.9210E-05	9.2179E-01	3.7260E-01
CCNA_00088	1.6389E-04	1.0085E-04	7.0053E-01	2.3148E-01
CCNA_00089	1.1293E-04	4.7775E-05	1.2409E+00	5.7616E-01
CCNA_00090	7.0374E-05	1.0549E-04	-5.8394E-01	-5.8782E-01
CCNA_00091	2.1128E-04	1.1906E-04	8.2740E-01	3.1240E-01
CCNA_00092	1.2266E-04	1.1210E-04	1.2983E-01	-1.3254E-01
CCNA_00093	1.0214E-04	8.0973E-05	3.3492E-01	-1.7238E-03
CCNA_00094	7.8074E-05	8.5688E-05	-1.3431E-01	-3.0102E-01
CCNA_00095	9.3352E-05	1.4926E-04	-6.7707E-01	-6.4721E-01
CCNA_00096	1.0004E-04	9.2649E-05	1.1064E-01	-1.4478E-01
CCNA_00097	5.9027E-05	1.5407E-04	-1.3841E+00	-1.0982E+00
CCNA_00098	6.3105E-05	2.8665E-05	1.1382E+00	5.1062E-01
CCNA_00099	6.9570E-05	1.6031E-04	-1.2043E+00	-9.8353E-01
CCNA_00100	1.5294E-04	1.3701E-04	1.5867E-01	-1.1415E-01
CCNA_00101	1.0183E-04	6.7258E-05	5.9831E-01	1.6628E-01
CCNA_00102	1.1874E-04	1.0137E-04	2.2819E-01	-6.9804E-02
CCNA_00103	6.0095E-05	1.5398E-04	-1.3574E+00	-1.0812E+00

CCNA_00104	1.2602E-04	1.5699E-04	-3.1703E-01	-4.1757E-01
CCNA_00105	1.3138E-04	1.6243E-04	-3.0606E-01	-4.1057E-01
CCNA_00106	1.6782E-04	6.8592E-05	1.2907E+00	6.0788E-01
CCNA_00107	1.1542E-04	1.1613E-04	-8.8727E-03	-2.2101E-01
CCNA_00108	7.1383E-05	8.2000E-05	-2.0011E-01	-3.4299E-01
CCNA_00109	1.4142E-04	1.6109E-04	-1.8788E-01	-3.3519E-01
CCNA_00110	1.2753E-04	1.1351E-04	1.6798E-01	-1.0821E-01
CCNA_00111	1.2041E-04	1.3453E-04	-1.6006E-01	-3.1744E-01
CCNA_00112	6.7614E-05	5.2026E-05	3.7795E-01	2.5723E-02
CCNA_00113	7.3547E-05	8.3823E-05	-1.8875E-01	-3.3574E-01
CCNA_00114	6.8132E-05	1.4648E-04	-1.1043E+00	-9.1972E-01
CCNA_00115	8.1019E-05	1.1810E-04	-5.4368E-01	-5.6213E-01
CCNA_00116	1.4090E-04	1.6243E-04	-2.0517E-01	-3.4622E-01
CCNA_00117	1.3911E-04	1.6243E-04	-2.2356E-01	-3.5795E-01
CCNA_00118	1.1045E-04	8.6028E-05	3.6046E-01	1.4566E-02
CCNA_00119	1.5749E-04	8.5613E-05	8.7928E-01	3.4549E-01
CCNA_00120	7.8185E-05	7.8371E-05	-3.4954E-03	-2.1758E-01
CCNA_00121	8.1024E-05	7.3978E-05	1.3116E-01	-1.3169E-01
CCNA_00122	1.1136E-04	1.5982E-04	-5.2129E-01	-5.4785E-01
CCNA_00123	1.0429E-04	1.5648E-04	-5.8538E-01	-5.8873E-01
CCNA_00124	1.0647E-04	1.3268E-04	-3.1742E-01	-4.1781E-01
CCNA_00125	1.4796E-04	1.1870E-04	3.1787E-01	-1.2602E-02
CCNA_00126	6.7690E-04	2.3684E-04	1.5150E+00	7.5099E-01
CCNA_00127	6.7679E-04	2.3803E-04	1.5075E+00	7.4621E-01
CCNA_00128	1.0743E-04	1.6243E-04	-5.9637E-01	-5.9574E-01
CCNA_00129	9.3407E-05	1.5702E-04	-7.4937E-01	-6.9333E-01
CCNA_00130	3.1797E-04	6.4253E-04	-1.0149E+00	-8.6267E-01
CCNA_00131	3.1763E-04	6.4137E-04	-1.0138E+00	-8.6201E-01
CCNA_00132	9.0124E-05	1.5981E-04	-8.2635E-01	-7.4243E-01
CCNA_00133	1.2570E-04	1.7032E-04	-4.3827E-01	-4.9490E-01
CCNA_00134	1.5647E-04	8.6608E-05	8.5323E-01	3.2887E-01
CCNA_00135	1.3137E-04	9.3420E-05	4.9172E-01	9.8288E-02
CCNA_00136	1.5483E-04	1.6243E-04	-6.9135E-02	-2.5945E-01
CCNA_00137	9.2741E-05	7.0838E-05	3.8860E-01	3.2513E-02
CCNA_00138	9.4022E-05	7.0904E-05	4.0704E-01	4.4274E-02
CCNA_00139	1.0734E-04	1.3864E-04	-3.6921E-01	-4.5085E-01
CCNA_00140	9.8068E-05	5.1893E-05	9.1807E-01	3.7023E-01
CCNA_00141	1.2171E-04	1.6276E-04	-4.1928E-01	-4.8279E-01
CCNA_00142	8.4164E-05	5.3584E-05	6.5125E-01	2.0004E-01
CCNA_00143	2.5251E-04	1.6243E-04	6.3651E-01	1.9064E-01
CCNA_00144	1.3359E-04	1.1660E-04	1.9622E-01	-9.0197E-02
CCNA_00145	1.3374E-04	1.3181E-04	2.0966E-02	-2.0198E-01
CCNA_00146	1.9049E-04	7.6059E-05	1.3244E+00	6.2942E-01
CCNA_00147	1.2787E-04	1.0482E-04	2.8667E-01	-3.2501E-02
CCNA_00148	1.4776E-04	1.6243E-04	-1.3657E-01	-3.0246E-01
CCNA_00149	7.3191E-05	1.6132E-04	-1.1402E+00	-9.4263E-01
CCNA_00150	1.1004E-04	1.2560E-04	-1.9088E-01	-3.3711E-01
CCNA_00151	1.1766E-04	1.0312E-04	1.9025E-01	-9.4001E-02
CCNA_00152	1.1861E-04	9.8318E-05	2.7068E-01	-4.2701E-02
CCNA_00153	1.0126E-04	8.8191E-05	1.9928E-01	-8.8242E-02
CCNA_00154	3.6753E-04	1.6243E-04	1.1780E+00	5.3605E-01
CCNA_00155	3.6198E-04	1.6243E-04	1.1561E+00	5.2203E-01
CCNA_00156	6.5288E-05	7.1012E-05	-1.2131E-01	-2.9273E-01

CCNA_00157	7.5595E-05	1.5968E-04	-1.0788E+00	-9.0348E-01
CCNA_00158	8.5768E-05	1.6881E-04	-9.7686E-01	-8.3843E-01
CCNA_00159	3.2166E-04	1.5178E-04	1.0835E+00	4.7576E-01
CCNA_00160	2.2808E-04	6.3180E-05	1.8518E+00	9.6580E-01
CCNA_00161	4.3139E-04	1.7985E-04	1.2622E+00	5.8973E-01
CCNA_00162	3.1739E-04	1.6199E-04	9.7035E-01	4.0358E-01
CCNA_00163	1.6091E-04	1.0839E-04	5.7000E-01	1.4822E-01
CCNA_00164	2.0240E-04	1.6231E-04	3.1843E-01	-1.2242E-02
CCNA_00165	1.4687E-04	7.7633E-05	9.1970E-01	3.7127E-01
CCNA_00166	1.6789E-04	2.3527E-05	2.8347E+00	1.5927E+00
CCNA_00167	1.7536E-04	4.2438E-05	2.0466E+00	1.0901E+00
CCNA_00168	1.3994E-04	1.6243E-04	-2.1505E-01	-3.5252E-01
CCNA_00169	8.5158E-05	1.6798E-05	2.3412E+00	1.2780E+00
CCNA_00170	1.0958E-04	1.6272E-04	-5.7043E-01	-5.7919E-01
CCNA_00171	1.3139E-04	1.6243E-04	-3.0590E-01	-4.1047E-01
CCNA_00172	6.5306E-05	7.3912E-05	-1.7865E-01	-3.2930E-01
CCNA_00173	7.7731E-05	6.7829E-05	1.9650E-01	-9.0019E-02
CCNA_00174	7.0425E-05	1.5866E-04	-1.1717E+00	-9.6272E-01
CCNA_00175	1.0727E-04	1.6277E-04	-6.0156E-01	-5.9905E-01
CCNA_00176	7.7662E-05	6.1896E-05	3.2726E-01	-6.6127E-03
CCNA_00177	7.1961E-05	6.1506E-05	2.2637E-01	-7.0966E-02
CCNA_00178	7.3436E-05	1.4681E-04	-9.9934E-01	-8.5277E-01
CCNA_00179	8.2050E-05	1.5275E-04	-8.9656E-01	-7.8721E-01
CCNA_00180	7.7574E-05	6.3015E-05	2.9978E-01	-2.4136E-02
CCNA_00181	9.2663E-05	8.9426E-05	5.1241E-02	-1.8267E-01
CCNA_00182	8.1907E-05	1.6243E-04	-9.8772E-01	-8.4536E-01
CCNA_00183	7.5193E-05	1.5866E-04	-1.0773E+00	-9.0249E-01
CCNA_00184	7.1156E-05	7.2603E-05	-2.9112E-02	-2.3392E-01
CCNA_00185	1.3547E-04	1.6164E-04	-2.5480E-01	-3.7787E-01
CCNA_00186	2.1386E-04	1.6242E-04	3.9689E-01	3.7801E-02
CCNA_00187	9.1419E-05	1.0232E-04	-1.6257E-01	-3.1905E-01
CCNA_00188	1.7106E-04	1.3432E-04	3.4878E-01	7.1134E-03
CCNA_00189	1.8860E-04	1.6156E-04	2.2329E-01	-7.2930E-02
CCNA_00190	9.5872E-05	1.4593E-04	-6.0616E-01	-6.0198E-01
CCNA_00191	9.1026E-05	1.6243E-04	-8.3544E-01	-7.4823E-01
CCNA_00192	4.6583E-05	1.2375E-04	-1.4095E+00	-1.1144E+00
CCNA_00193	7.6275E-05	1.5682E-04	-1.0398E+00	-8.7857E-01
CCNA_00194	6.8479E-05	5.6592E-05	2.7493E-01	-3.9987E-02
CCNA_00195	5.7048E-05	5.8109E-05	-2.6675E-02	-2.3237E-01
CCNA_00196	7.3302E-05	1.6243E-04	-1.1479E+00	-9.4750E-01
CCNA_00197	1.0853E-04	8.0882E-05	4.2416E-01	5.5196E-02
CCNA_00198	1.4529E-04	1.9717E-04	-4.4044E-01	-4.9628E-01
CCNA_00199	1.5304E-04	3.1739E-04	-1.0524E+00	-8.8662E-01
CCNA_00200	6.1263E-04	1.6243E-04	1.9152E+00	1.0062E+00
CCNA_00201	8.4245E-04	1.2732E-04	2.7260E+00	1.5234E+00
CCNA_00202	1.2220E-04	8.9185E-05	4.5425E-01	7.4390E-02
CCNA_00203	1.1375E-04	1.3016E-04	-1.9443E-01	-3.3937E-01
CCNA_00204	8.7169E-05	8.5058E-05	3.5304E-02	-1.9283E-01
CCNA_00205	1.4609E-04	9.8442E-05	5.6944E-01	1.4786E-01
CCNA_00206	1.3919E-04	1.0088E-04	4.6433E-01	8.0817E-02
CCNA_00207	7.6108E-05	1.5739E-04	-1.0482E+00	-8.8393E-01
CCNA_00208	7.5147E-05	1.6243E-04	-1.1120E+00	-9.2463E-01
CCNA_00209	2.4275E-04	1.6270E-04	5.7723E-01	1.5283E-01

CCNA_00210	1.8808E-04	1.5524E-04	2.7680E-01	-3.8795E-02
CCNA_00211	6.0872E-05	1.4510E-04	-1.2532E+00	-1.0147E+00
CCNA_00212	9.3490E-05	1.6272E-04	-7.9954E-01	-7.2533E-01
CCNA_00213	6.4863E-05	5.5540E-05	2.2375E-01	-7.2634E-02
CCNA_00214	8.1708E-05	7.0042E-05	2.2217E-01	-7.3643E-02
CCNA_00215	1.0727E-04	1.7853E-04	-7.3497E-01	-6.8414E-01
CCNA_00216	1.1962E-04	1.0230E-04	2.2550E-01	-7.1518E-02
CCNA_00217	1.2513E-04	1.4201E-04	-1.8258E-01	-3.3181E-01
CCNA_00218	1.0968E-04	9.6677E-05	1.8205E-01	-9.9231E-02
CCNA_00219	1.0588E-04	1.2833E-04	-2.7745E-01	-3.9232E-01
CCNA_00220	9.8799E-05	1.5983E-04	-6.9400E-01	-6.5801E-01
CCNA_00221	1.1489E-04	1.2564E-04	-1.2913E-01	-2.9771E-01
CCNA_00222	1.1670E-04	1.4501E-04	-3.1330E-01	-4.1519E-01
CCNA_00223	7.9294E-05	1.6063E-04	-1.0184E+00	-8.6494E-01
CCNA_00224	1.1635E-04	8.1536E-05	5.1289E-01	1.1179E-01
CCNA_00225	1.0590E-04	9.3238E-05	1.8368E-01	-9.8196E-02
CCNA_00226	1.7656E-04	1.6146E-04	1.2892E-01	-1.3312E-01
CCNA_00227	1.1841E-04	9.2649E-05	3.5392E-01	1.0395E-02
CCNA_00228	9.9733E-05	1.6228E-04	-7.0232E-01	-6.6332E-01
CCNA_00229	1.1047E-04	1.0834E-04	2.8147E-02	-1.9740E-01
CCNA_00230	8.7738E-05	9.4265E-05	-1.0357E-01	-2.8141E-01
CCNA_00231	9.5109E-05	8.5928E-05	1.4638E-01	-1.2199E-01
CCNA_00232	2.5074E-04	2.0349E-04	3.0120E-01	-2.3232E-02
CCNA_00233	2.5286E-04	2.1488E-04	2.3476E-01	-6.5611E-02
CCNA_00234	7.5831E-05	9.5251E-05	-3.2899E-01	-4.2520E-01
CCNA_00235	9.6589E-05	9.8450E-05	-2.7593E-02	-2.3295E-01
CCNA_00236	2.3766E-04	2.6296E-04	-1.4596E-01	-3.0845E-01
CCNA_00237	2.3952E-04	2.7782E-04	-2.1402E-01	-3.5186E-01
CCNA_00238	1.0334E-04	1.6243E-04	-6.5233E-01	-6.3144E-01
CCNA_00239	6.3609E-05	9.8309E-05	-6.2810E-01	-6.1598E-01
CCNA_00240	3.3724E-04	1.0791E-04	1.6439E+00	8.3320E-01
CCNA_00241	1.5125E-04	8.8091E-05	7.7973E-01	2.8199E-01
CCNA_00242	1.9865E-04	1.5720E-04	3.3763E-01	1.8998E-06
CCNA_00243	9.8119E-05	1.6273E-04	-7.2990E-01	-6.8091E-01
CCNA_00244	1.1955E-04	1.4421E-04	-2.7057E-01	-3.8793E-01
CCNA_00245	1.0529E-04	1.6246E-04	-6.2570E-01	-6.1445E-01
CCNA_00246	9.2265E-05	1.0765E-04	-2.2251E-01	-3.5728E-01
CCNA_00247	1.4438E-04	9.6793E-05	5.7681E-01	1.5256E-01
CCNA_00248	2.2859E-04	2.3793E-04	-5.7801E-02	-2.5222E-01
CCNA_00249	2.4495E-04	1.7920E-04	4.5090E-01	7.2254E-02
CCNA_00250	1.6983E-04	1.6243E-04	6.4234E-02	-1.7438E-01
CCNA_00251	1.2752E-04	8.9376E-05	5.1268E-01	1.1166E-01
CCNA_00252	1.4581E-04	1.6359E-04	-1.6601E-01	-3.2124E-01
CCNA_00253	1.4960E-04	1.7636E-04	-2.3737E-01	-3.6676E-01
CCNA_00254	6.3170E-05	3.3289E-05	9.2394E-01	3.7398E-01
CCNA_00255	6.3295E-05	1.3810E-04	-1.1256E+00	-9.3329E-01
CCNA_00256	1.6156E-04	1.1666E-04	4.6976E-01	8.4280E-02
CCNA_00257	1.7794E-04	1.4202E-04	3.2518E-01	-7.9358E-03
CCNA_00258	2.0358E-04	1.6274E-04	3.2295E-01	-9.3575E-03
CCNA_00259	1.0694E-04	1.4773E-04	-4.6618E-01	-5.1270E-01
CCNA_00260	1.8332E-04	1.5669E-04	2.2639E-01	-7.0953E-02
CCNA_00261	1.7538E-04	1.0893E-04	6.8700E-01	2.2284E-01
CCNA_00262	5.7473E-05	1.6379E-04	-1.5108E+00	-1.1790E+00

CCNA_00263	7.9142E-05	1.2238E-04	-6.2891E-01	-6.1649E-01
CCNA_00264	2.6326E-04	1.9986E-04	3.9750E-01	3.8191E-02
CCNA_00265	5.2917E-04	2.3923E-04	1.1453E+00	5.1516E-01
CCNA_00266	6.6014E-05	1.6128E-04	-1.2887E+00	-1.0374E+00
CCNA_00267	5.5137E-04	1.6280E-04	1.7599E+00	9.0717E-01
CCNA_00268	5.4211E-04	1.6319E-04	1.7320E+00	8.8939E-01
CCNA_00269	5.8680E-05	1.0554E-04	-8.4679E-01	-7.5547E-01
CCNA_00270	5.8569E-05	3.5303E-05	7.3013E-01	2.5036E-01
CCNA_00271	8.5306E-05	2.9104E-05	1.5511E+00	7.7400E-01
CCNA_00272	8.5116E-05	1.0332E-04	-2.7969E-01	-3.9375E-01
CCNA_00273	1.6860E-04	1.6272E-04	5.1213E-02	-1.8269E-01
CCNA_00274	1.0237E-04	5.8076E-05	8.1763E-01	3.0617E-01
CCNA_00275	2.0765E-04	1.7261E-04	2.6660E-01	-4.5304E-02
CCNA_00276	3.6208E-04	1.1040E-04	1.7135E+00	8.7758E-01
CCNA_00277	4.0967E-04	7.5097E-05	2.4475E+00	1.3458E+00
CCNA_00278	7.0495E-05	9.6528E-05	-4.5346E-01	-5.0458E-01
CCNA_00279	1.1430E-03	1.3025E-04	3.1335E+00	1.7833E+00
CCNA_00280	7.0097E-05	1.1324E-04	-6.9200E-01	-6.5674E-01
CCNA_00281	1.0233E-04	1.0176E-04	8.0132E-03	-2.1024E-01
CCNA_00282	1.0272E-04	1.2207E-04	-2.4900E-01	-3.7417E-01
CCNA_00283	7.3237E-05	1.5392E-04	-1.0715E+00	-8.9879E-01
CCNA_00284	1.1327E-04	1.6243E-04	-5.2006E-01	-5.4707E-01
CCNA_00285	8.3193E-05	1.5256E-04	-8.7489E-01	-7.7339E-01
CCNA_00286	6.9709E-05	1.5985E-04	-1.1973E+00	-9.7903E-01
CCNA_00287	9.1558E-05	1.6274E-04	-8.2983E-01	-7.4465E-01
CCNA_00288	7.4971E-05	1.4134E-04	-9.1472E-01	-7.9880E-01
CCNA_00289	6.3891E-05	7.0191E-06	3.1847E+00	1.8160E+00
CCNA_00290	3.2055E-04	1.6179E-04	9.8641E-01	4.1382E-01
CCNA_00291	5.8324E-04	2.3574E-04	1.3068E+00	6.1820E-01
CCNA_00292	9.3028E-05	1.5982E-04	-7.8075E-01	-7.1335E-01
CCNA_00293	8.7382E-05	5.2167E-05	7.4405E-01	2.5923E-01
CCNA_00294	1.1468E-04	1.0161E-04	1.7458E-01	-1.0400E-01
CCNA_00295	9.6089E-05	9.9851E-05	-5.5448E-02	-2.5072E-01
CCNA_00296	9.1604E-05	5.2929E-05	7.9119E-01	2.8930E-01
CCNA_00297	1.5101E-04	1.0195E-04	5.6679E-01	1.4617E-01
CCNA_00298	7.2035E-05	1.4680E-04	-1.0270E+00	-8.7045E-01
CCNA_00299	8.6503E-05	1.1231E-04	-3.7674E-01	-4.5565E-01
CCNA_00300	3.5545E-04	3.0972E-04	1.9865E-01	-8.8645E-02
CCNA_00301	3.9762E-04	3.2485E-04	2.9158E-01	-2.9368E-02
CCNA_00302	1.2265E-04	8.1851E-05	5.8338E-01	1.5675E-01
CCNA_00303	5.7223E-05	1.9209E-05	1.5743E+00	7.8880E-01
CCNA_00304	1.1669E-04	9.9809E-05	2.2537E-01	-7.1602E-02
CCNA_00305	9.8313E-05	1.6243E-04	-7.2433E-01	-6.7736E-01
CCNA_00306	6.5750E-05	1.2911E-04	-9.7355E-01	-8.3632E-01
CCNA_00307	1.1083E-04	2.3452E-04	-1.0813E+00	-9.0506E-01
CCNA_00308	1.1339E-04	2.3908E-04	-1.0762E+00	-9.0178E-01
CCNA_00309	1.4952E-04	1.5943E-04	-9.2617E-02	-2.7443E-01
CCNA_00310	6.3656E-05	1.0176E-04	-6.7689E-01	-6.4710E-01
CCNA_00311	1.2449E-04	6.4150E-05	9.5638E-01	3.9467E-01
CCNA_00312	1.1449E-04	6.7299E-05	7.6642E-01	2.7351E-01
CCNA_00313	1.0057E-04	1.2131E-04	-2.7065E-01	-3.8798E-01
CCNA_00314	7.7879E-05	1.6243E-04	-1.0605E+00	-8.9176E-01
CCNA_00315	6.8696E-05	4.7617E-05	5.2858E-01	1.2180E-01

CCNA_00316	7.0638E-05	1.2828E-04	-8.6072E-01	-7.6436E-01
CCNA_00317	1.3599E-04	1.6243E-04	-2.5635E-01	-3.7886E-01
CCNA_00318	1.2793E-04	1.3523E-04	-8.0066E-02	-2.6642E-01
CCNA_00319	9.3680E-05	1.4205E-04	-6.0058E-01	-5.9843E-01
CCNA_00320	1.6305E-04	5.7777E-05	1.4965E+00	7.3921E-01
CCNA_00321	5.6292E-04	6.6317E-04	-2.3646E-01	-3.6618E-01
CCNA_00322	5.6307E-04	6.6026E-04	-2.2974E-01	-3.6189E-01
CCNA_00323	1.6297E-04	1.4233E-04	1.9530E-01	-9.0779E-02
CCNA_00324	4.9819E-04	1.9831E-04	1.3289E+00	6.3228E-01
CCNA_00325	3.7965E-04	2.2437E-04	7.5876E-01	2.6862E-01
CCNA_00326	4.5012E-04	1.9391E-04	1.2149E+00	5.5956E-01
CCNA_00327	1.6028E-04	1.5504E-04	4.7866E-02	-1.8482E-01
CCNA_00328	1.3708E-04	1.0583E-04	3.7312E-01	2.2643E-02
CCNA_00329	1.7705E-04	1.4344E-04	3.0368E-01	-2.1650E-02
CCNA_00330	1.1830E-04	1.3273E-04	-1.6613E-01	-3.2132E-01
CCNA_00331	1.0187E-04	1.6386E-04	-6.8575E-01	-6.5275E-01
CCNA_00332	9.1067E-05	9.2467E-05	-2.2058E-02	-2.2942E-01
CCNA_00333	8.7266E-05	7.4277E-05	2.3243E-01	-6.7098E-02
CCNA_00334	7.6233E-05	1.6477E-04	-1.1120E+00	-9.2461E-01
CCNA_00335	1.1078E-04	1.6085E-04	-5.3799E-01	-5.5850E-01
CCNA_00336	6.4516E-05	9.7539E-05	-5.9634E-01	-5.9572E-01
CCNA_00337	7.7704E-05	1.5783E-04	-1.0223E+00	-8.6740E-01
CCNA_00338	1.2747E-04	9.1298E-05	4.8140E-01	9.1706E-02
CCNA_00339	6.7512E-05	1.1652E-04	-7.8741E-01	-7.1760E-01
CCNA_00340	1.6627E-04	2.2279E-04	-4.2213E-01	-4.8460E-01
CCNA_00341	9.7402E-05	1.1444E-04	-2.3255E-01	-3.6368E-01
CCNA_00342	9.1294E-05	4.1270E-05	1.1452E+00	5.1512E-01
CCNA_00343	7.5674E-05	9.8616E-05	-3.8206E-01	-4.5905E-01
CCNA_00344	6.6236E-05	3.7772E-05	8.1007E-01	3.0134E-01
CCNA_00345	1.2481E-04	1.6243E-04	-3.8002E-01	-4.5775E-01
CCNA_00346	1.4569E-04	1.7252E-04	-2.4386E-01	-3.7090E-01
CCNA_00347	7.4587E-05	4.6582E-05	6.7900E-01	2.1774E-01
CCNA_00348	2.2281E-04	1.6243E-04	4.5598E-01	7.5490E-02
CCNA_00349	1.2947E-04	1.6254E-04	-3.2821E-01	-4.2470E-01
CCNA_00350	2.6743E-04	2.1548E-04	3.1159E-01	-1.6610E-02
CCNA_00351	2.7483E-04	2.1462E-04	3.5671E-01	1.2175E-02
CCNA_00352	2.8110E-04	1.6243E-04	7.9127E-01	2.8936E-01
CCNA_00353	2.8996E-04	1.8077E-04	6.8169E-01	2.1946E-01
CCNA_00354	3.7305E-04	2.4174E-04	6.2586E-01	1.8385E-01
CCNA_00355	1.9389E-04	1.1054E-04	8.1058E-01	3.0167E-01
CCNA_00356	9.1743E-05	1.1640E-04	-3.4346E-01	-4.3442E-01
CCNA_00357	1.4656E-04	8.4569E-05	7.9316E-01	2.9056E-01
CCNA_00358	1.4230E-04	7.4418E-05	9.3508E-01	3.8108E-01
CCNA_00359	1.5521E-04	9.9370E-05	6.4329E-01	1.9497E-01
CCNA_00360	1.0546E-04	1.6243E-04	-6.2313E-01	-6.1281E-01
CCNA_00361	1.2622E-04	1.4894E-04	-2.3885E-01	-3.6770E-01
CCNA_00362	6.2060E-05	6.0860E-05	2.8086E-02	-1.9744E-01
CCNA_00363	8.0511E-05	1.3279E-04	-7.2192E-01	-6.7582E-01
CCNA_00364	8.1366E-05	7.1542E-05	1.8555E-01	-9.7001E-02
CCNA_00365	2.4688E-04	3.2455E-04	-3.9465E-01	-4.6707E-01
CCNA_00366	2.5705E-04	3.0170E-04	-2.3110E-01	-3.6275E-01
CCNA_00367	2.3777E-04	6.0346E-05	1.9780E+00	1.0463E+00
CCNA_00368	7.8198E-05	1.3710E-04	-8.1003E-01	-7.3202E-01

CCNA_00369	1.8860E-04	1.6538E-04	1.8957E-01	-9.4436E-02
CCNA_00370	6.5020E-05	1.5044E-04	-1.2102E+00	-9.8730E-01
CCNA_00371	8.0862E-05	6.4274E-05	3.3111E-01	-4.1526E-03
CCNA_00372	1.2055E-04	1.1302E-04	9.2973E-02	-1.5605E-01
CCNA_00373	1.1552E-04	1.2648E-04	-1.3077E-01	-2.9876E-01
CCNA_00374	2.7904E-04	5.5682E-04	-9.9673E-01	-8.5111E-01
CCNA_00375	2.4335E-04	4.2141E-04	-7.9218E-01	-7.2064E-01
CCNA_00376	1.1262E-04	1.3457E-04	-2.5695E-01	-3.7925E-01
CCNA_00377	7.6908E-05	6.9528E-05	1.4545E-01	-1.2257E-01
CCNA_00378	6.7901E-05	7.2719E-05	-9.8972E-02	-2.7848E-01
CCNA_00379	3.1100E-04	2.0360E-04	6.1114E-01	1.7446E-01
CCNA_00380	1.0700E-04	5.8556E-05	8.6960E-01	3.3932E-01
CCNA_00381	1.0602E-04	9.7340E-05	1.2314E-01	-1.3681E-01
CCNA_00382	1.2802E-04	1.6243E-04	-3.4345E-01	-4.3442E-01
CCNA_00383	2.0007E-04	1.8593E-04	1.0574E-01	-1.4791E-01
CCNA_00384	1.7420E-04	1.4448E-04	2.6983E-01	-4.3245E-02
CCNA_00385	7.7112E-05	7.0871E-05	1.2168E-01	-1.3774E-01
CCNA_00386	7.1470E-05	1.5934E-04	-1.1566E+00	-9.5310E-01
CCNA_00387	6.9676E-05	1.2959E-04	-8.9524E-01	-7.8638E-01
CCNA_00388	8.3354E-05	1.4796E-04	-8.2785E-01	-7.4339E-01
CCNA_00389	5.7562E-04	1.4288E-04	2.0103E+00	1.0669E+00
CCNA_00390	5.5858E-04	1.4714E-04	1.9245E+00	1.0122E+00
CCNA_00391	7.4370E-05	4.8587E-05	6.1399E-01	1.7628E-01
CCNA_00392	1.0792E-04	1.2731E-04	-2.3840E-01	-3.6741E-01
CCNA_00393	8.4330E-05	1.3745E-04	-7.0479E-01	-6.6489E-01
CCNA_00394	9.8156E-05	1.6460E-04	-7.4579E-01	-6.9105E-01
CCNA_00395	1.1352E-04	7.9614E-05	5.1174E-01	1.1105E-01
CCNA_00396	1.2904E-04	1.4343E-04	-1.5263E-01	-3.1270E-01
CCNA_00397	1.3046E-04	1.1016E-04	2.4396E-01	-5.9746E-02
CCNA_00398	2.0945E-04	1.6278E-04	3.6362E-01	1.6578E-02
CCNA_00399	1.8470E-04	1.4396E-04	3.5942E-01	1.3899E-02
CCNA_00400	8.2698E-05	1.5425E-04	-8.9932E-01	-7.8897E-01
CCNA_00401	7.7223E-05	5.6261E-05	4.5677E-01	7.5996E-02
CCNA_00402	5.6017E-05	4.5264E-05	3.0735E-01	-1.9311E-02
CCNA_00403	9.4055E-05	9.5243E-05	-1.8170E-02	-2.2694E-01
CCNA_00404	6.0789E-05	7.1932E-05	-2.4288E-01	-3.7027E-01
CCNA_00405	6.2268E-05	3.4590E-05	8.4790E-01	3.2548E-01
CCNA_00406	8.1421E-05	1.1138E-04	-4.5201E-01	-5.0366E-01
CCNA_00407	8.5389E-05	3.4366E-05	1.3128E+00	6.2200E-01
CCNA_00408	1.2454E-04	1.6285E-04	-3.8698E-01	-4.6218E-01
CCNA_00409	1.4926E-04	1.5401E-04	-4.5300E-02	-2.4425E-01
CCNA_00410	1.3413E-04	1.6694E-04	-3.1572E-01	-4.1673E-01
CCNA_00411	7.6058E-05	7.7890E-05	-3.4412E-02	-2.3730E-01
CCNA_00412	8.1264E-05	7.6059E-05	9.5436E-02	-1.5448E-01
CCNA_00413	1.7207E-04	1.6028E-04	1.0235E-01	-1.5007E-01
CCNA_00414	6.7188E-05	1.5614E-04	-1.2166E+00	-9.9133E-01
CCNA_00415	1.0512E-04	1.5027E-04	-5.1559E-01	-5.4422E-01
CCNA_00416	8.3502E-05	1.6334E-04	-9.6796E-01	-8.3276E-01
CCNA_00417	1.0928E-04	4.4497E-04	-2.0256E+00	-1.5074E+00
CCNA_00418	1.1671E-04	4.5193E-04	-1.9532E+00	-1.4612E+00
CCNA_00419	1.1480E-04	4.7114E-04	-2.0370E+00	-1.5146E+00
CCNA_00420	5.7478E-05	1.6197E-04	-1.4946E+00	-1.1687E+00
CCNA_00421	8.3586E-05	5.0808E-05	7.1805E-01	2.4265E-01

CCNA_00422	3.7315E-04	3.1375E-04	2.5012E-01	-5.5813E-02
CCNA_00423	3.0582E-04	2.6714E-04	1.9504E-01	-9.0948E-02
CCNA_00424	8.5260E-05	8.1379E-05	6.7139E-02	-1.7253E-01
CCNA_00425	5.2299E-05	9.1307E-05	-8.0395E-01	-7.2814E-01
CCNA_00426	9.6630E-05	1.0819E-04	-1.6303E-01	-3.1934E-01
CCNA_00427	9.7897E-05	1.2465E-04	-3.4852E-01	-4.3765E-01
CCNA_00428	8.6402E-05	1.2512E-04	-5.3417E-01	-5.5607E-01
CCNA_00429	7.2451E-05	3.7068E-05	9.6660E-01	4.0118E-01
CCNA_00430	6.8303E-05	1.1328E-04	-7.2993E-01	-6.8093E-01
CCNA_00431	6.5496E-05	1.4258E-04	-1.1223E+00	-9.3118E-01
CCNA_00432	9.7869E-05	1.0155E-04	-5.3302E-02	-2.4935E-01
CCNA_00433	9.7046E-05	1.2385E-04	-3.5187E-01	-4.3979E-01
CCNA_00434	7.7611E-05	2.6668E-05	1.5408E+00	7.6744E-01
CCNA_00435	1.1911E-04	1.6259E-04	-4.4895E-01	-5.0171E-01
CCNA_00436	7.7005E-05	5.1653E-05	5.7596E-01	1.5202E-01
CCNA_00437	7.3080E-05	7.0904E-05	4.3524E-02	-1.8759E-01
CCNA_00438	4.1195E-04	9.6105E-05	2.0997E+00	1.1239E+00
CCNA_00439	4.1421E-04	1.1609E-04	1.8350E+00	9.5508E-01
CCNA_00440	6.9690E-05	5.2838E-05	3.9924E-01	3.9301E-02
CCNA_00441	1.0135E-04	1.4703E-04	-5.3675E-01	-5.5771E-01
CCNA_00442	9.1423E-05	5.0551E-05	8.5466E-01	3.2979E-01
CCNA_00443	8.5135E-05	1.5363E-04	-8.5160E-01	-7.5854E-01
CCNA_00444	1.0405E-04	1.5738E-04	-5.9695E-01	-5.9611E-01
CCNA_00445	1.6755E-04	8.2158E-05	1.0281E+00	4.4039E-01
CCNA_00446	1.2364E-04	1.6205E-04	-3.9029E-01	-4.6430E-01
CCNA_00447	1.0126E-04	1.4868E-04	-5.5409E-01	-5.6877E-01
CCNA_00448	8.2462E-05	1.0013E-04	-2.8015E-01	-3.9404E-01
CCNA_00449	8.7086E-05	7.1468E-05	2.8506E-01	-3.3526E-02
CCNA_00450	4.5753E-04	6.5368E-05	2.8070E+00	1.5751E+00
CCNA_00451	1.5388E-04	1.5804E-04	-3.8524E-02	-2.3992E-01
CCNA_00452	7.8943E-05	6.0015E-05	3.9538E-01	3.6837E-02
CCNA_00453	7.9646E-05	1.6300E-04	-1.0332E+00	-8.7435E-01
CCNA_00454	1.9806E-04	8.6260E-05	1.1991E+00	5.4949E-01
CCNA_00455	1.9589E-04	8.4644E-05	1.2105E+00	5.5674E-01
CCNA_00456	8.3021E-05	8.9931E-05	-1.1539E-01	-2.8895E-01
CCNA_00457	8.2245E-05	1.4830E-04	-8.5049E-01	-7.5783E-01
CCNA_00458	1.0059E-04	1.9238E-04	-9.3546E-01	-8.1202E-01
CCNA_00459	2.8722E-04	1.6590E-04	7.9180E-01	2.8969E-01
CCNA_00460	1.4086E-04	1.6243E-04	-2.0555E-01	-3.4646E-01
CCNA_04006	2.0609E-04	1.2760E-04	6.9156E-01	2.2575E-01
CCNA_00464	6.3683E-04	2.4717E-04	1.3654E+00	6.5554E-01
CCNA_00465	4.9190E-04	1.6260E-04	1.5970E+00	8.0326E-01
CCNA_00466	9.4290E-05	6.1018E-05	6.2776E-01	1.8506E-01
CCNA_00467	1.3699E-04	7.3100E-05	9.0601E-01	3.6254E-01
CCNA_00468	2.0041E-04	1.5999E-04	3.2496E-01	-8.0771E-03
CCNA_00469	1.3129E-04	7.3738E-05	8.3214E-01	3.1542E-01
CCNA_04002	1.3199E-04	7.1119E-05	8.9195E-01	3.5357E-01
CCNA_00470	1.2137E-04	1.6010E-04	-3.9951E-01	-4.7018E-01
CCNA_00471	8.8131E-05	1.6116E-04	-8.7075E-01	-7.7075E-01
CCNA_00472	2.9433E-04	1.6274E-04	8.5483E-01	3.2989E-01
CCNA_00473	8.4386E-05	1.6134E-04	-9.3504E-01	-8.1176E-01
CCNA_00474	5.8259E-05	8.7387E-05	-5.8495E-01	-5.8846E-01
CCNA_00475	7.3528E-05	6.3769E-05	2.0535E-01	-8.4372E-02

CCNA_00476	5.6687E-05	1.1900E-04	-1.0699E+00	-8.9776E-01
CCNA_00477	9.4656E-05	1.6121E-04	-7.6816E-01	-7.0532E-01
CCNA_00478	2.4845E-04	6.8260E-05	1.8637E+00	9.7338E-01
CCNA_00479	2.4711E-04	7.1932E-05	1.7803E+00	9.2020E-01
CCNA_00480	1.0647E-04	3.8178E-05	1.4793E+00	7.2821E-01
CCNA_00481	1.4294E-04	9.0047E-05	6.6658E-01	2.0982E-01
CCNA_00482	9.9506E-05	8.0335E-05	3.0869E-01	-1.8459E-02
CCNA_00483	1.6108E-04	1.8708E-04	-2.1593E-01	-3.5308E-01
CCNA_00484	9.5881E-05	1.8963E-04	-9.8388E-01	-8.4291E-01
CCNA_00485	7.5697E-05	1.5351E-04	-1.0200E+00	-8.6596E-01
CCNA_00486	6.6236E-05	1.5113E-04	-1.1901E+00	-9.7444E-01
CCNA_00487	6.6624E-05	1.4245E-04	-1.0963E+00	-9.1460E-01
CCNA_00488	8.8857E-05	1.6243E-04	-8.7022E-01	-7.7042E-01
CCNA_00489	1.7568E-04	1.6243E-04	1.1316E-01	-1.4317E-01
CCNA_00490	1.8800E-04	1.8272E-04	4.1045E-02	-1.8917E-01
CCNA_00491	2.4511E-04	1.7879E-04	4.5508E-01	7.4915E-02
CCNA_00492	2.4319E-04	1.7638E-04	4.6335E-01	8.0195E-02
CCNA_00493	6.6578E-05	1.0491E-04	-6.5599E-01	-6.3377E-01
CCNA_00494	9.1294E-05	8.9964E-05	2.1109E-02	-2.0189E-01
CCNA_00495	4.1501E-04	3.2485E-04	3.5333E-01	1.0014E-02
CCNA_00496	1.3668E-04	1.2029E-04	1.8432E-01	-9.7783E-02
CCNA_00497	2.3365E-04	1.5817E-04	5.6284E-01	1.4365E-01
CCNA_00498	5.8944E-05	1.6200E-04	-1.4586E+00	-1.1457E+00
CCNA_00499	6.5413E-05	1.0376E-04	-6.6565E-01	-6.3993E-01
CCNA_00500	5.8560E-05	9.4928E-05	-6.9694E-01	-6.5989E-01
CCNA_00501	1.9265E-04	1.6599E-04	2.1485E-01	-7.8310E-02
CCNA_00502	2.0444E-04	9.6834E-05	1.0780E+00	4.7225E-01
CCNA_00503	1.4269E-04	1.6243E-04	-1.8696E-01	-3.3460E-01
CCNA_00504	5.3439E-04	2.5716E-04	1.0552E+00	4.5768E-01
CCNA_00505	5.2524E-04	2.5902E-04	1.0199E+00	4.3518E-01
CCNA_00506	1.4314E-04	9.9113E-05	5.3020E-01	1.2283E-01
CCNA_00507	1.3926E-04	1.4169E-04	-2.4966E-02	-2.3128E-01
CCNA_00508	1.2394E-04	1.5939E-04	-3.6297E-01	-4.4687E-01
CCNA_00509	7.9965E-05	1.1895E-04	-5.7296E-01	-5.8081E-01
CCNA_00510	6.7961E-05	1.3634E-04	-1.0044E+00	-8.5601E-01
CCNA_00511	6.2366E-05	1.3041E-04	-1.0643E+00	-8.9418E-01
CCNA_00512	1.3269E-04	1.6243E-04	-2.9171E-01	-4.0141E-01
CCNA_00513	8.3738E-05	1.6243E-04	-9.5582E-01	-8.2501E-01
CCNA_00514	8.9042E-05	1.5649E-04	-8.1354E-01	-7.3426E-01
CCNA_00515	1.1644E-04	1.5947E-04	-4.5370E-01	-5.0474E-01
CCNA_00516	7.3006E-05	1.4604E-04	-1.0003E+00	-8.5339E-01
CCNA_00517	6.3646E-05	1.2322E-04	-9.5308E-01	-8.2327E-01
CCNA_00518	1.3279E-04	3.1532E-04	-1.2477E+00	-1.0112E+00
CCNA_00519	1.7558E-04	1.9030E-04	-1.1622E-01	-2.8948E-01
CCNA_00520	7.5017E-05	1.6511E-04	-1.1381E+00	-9.4130E-01
CCNA_00521	8.6096E-05	5.1902E-05	7.3002E-01	2.5028E-01
CCNA_00522	6.9371E-05	1.6116E-04	-1.2161E+00	-9.9100E-01
CCNA_00523	1.4071E-04	9.8094E-05	5.2039E-01	1.1657E-01
CCNA_00524	8.5278E-05	1.5434E-04	-8.5585E-01	-7.6125E-01
CCNA_00525	3.4729E-04	2.5956E-04	4.2005E-01	5.2576E-02
CCNA_00526	3.3149E-04	2.4451E-04	4.3906E-01	6.4702E-02
CCNA_00527	2.8898E-04	1.4727E-04	9.7248E-01	4.0494E-01
CCNA_00528	6.7933E-05	1.3853E-04	-1.0280E+00	-8.7103E-01

CCNA_00529	5.3728E-05	1.6278E-04	-1.5992E+00	-1.2354E+00
CCNA_00530	3.5950E-04	4.5841E-04	-3.5066E-01	-4.3902E-01
CCNA_00531	1.0965E-04	8.5067E-05	3.6612E-01	1.8178E-02
CCNA_00532	8.9467E-05	8.1329E-05	1.3752E-01	-1.2764E-01
CCNA_00533	1.2922E-04	6.6818E-05	9.5134E-01	3.9145E-01
CCNA_00534	1.3868E-04	8.5721E-05	6.9392E-01	2.2726E-01
CCNA_00535	1.0340E-04	1.1562E-04	-1.6127E-01	-3.1822E-01
CCNA_00536	2.1934E-04	2.0993E-04	6.3254E-02	-1.7501E-01
CCNA_00537	9.5622E-05	1.5963E-04	-7.3928E-01	-6.8689E-01
CCNA_00538	9.5946E-05	6.2236E-05	6.2435E-01	1.8289E-01
CCNA_00539	1.9222E-04	1.2787E-04	5.8800E-01	1.5970E-01
CCNA_00540	8.2490E-05	8.3194E-05	-1.2323E-02	-2.2321E-01
CCNA_00541	8.0580E-05	8.7279E-05	-1.1527E-01	-2.8888E-01
CCNA_00542	1.4443E-04	1.6105E-04	-1.5722E-01	-3.1563E-01
CCNA_00543	2.5950E-04	2.0307E-04	3.5370E-01	1.0253E-02
CCNA_00544	2.5782E-04	2.0228E-04	3.5000E-01	7.8938E-03
CCNA_00545	7.4610E-05	7.4318E-05	5.5843E-03	-2.1179E-01
CCNA_00546	1.4181E-04	1.6386E-04	-2.0850E-01	-3.4834E-01
CCNA_00547	2.3345E-04	7.6158E-05	1.6159E+00	8.1536E-01
CCNA_00548	2.7244E-04	3.0628E-04	-1.6894E-01	-3.2311E-01
CCNA_00549	2.7100E-04	5.3995E-04	-9.9455E-01	-8.4972E-01
CCNA_00550	4.4245E-04	3.6182E-04	2.9020E-01	-3.0247E-02
CCNA_00551	8.1693E-04	7.3514E-05	3.4740E+00	2.0005E+00
CCNA_00552	1.0133E-04	9.4481E-05	1.0095E-01	-1.5096E-01
CCNA_00553	3.0027E-04	1.5907E-04	9.1656E-01	3.6927E-01
CCNA_00554	2.9712E-04	1.6229E-04	8.7238E-01	3.4109E-01
CCNA_00555	9.4374E-05	9.3693E-05	1.0380E-02	-2.0873E-01
CCNA_00556	1.0208E-04	1.6241E-04	-6.6992E-01	-6.4265E-01
CCNA_00557	8.2559E-05	1.5053E-04	-8.6651E-01	-7.6805E-01
CCNA_00558	5.4111E-05	1.3251E-04	-1.2921E+00	-1.0395E+00
CCNA_00559	1.0192E-04	1.6243E-04	-6.7242E-01	-6.4425E-01
CCNA_00560	1.0245E-04	1.6243E-04	-6.6484E-01	-6.3942E-01
CCNA_00561	5.6484E-05	1.1357E-04	-1.0077E+00	-8.5811E-01
CCNA_00562	5.8019E-05	7.4302E-05	-3.5692E-01	-4.4301E-01
CCNA_00563	4.6699E-05	7.1285E-05	-6.1024E-01	-6.0459E-01
CCNA_00564	1.1714E-04	1.6295E-04	-4.7616E-01	-5.1907E-01
CCNA_00565	3.5749E-04	2.3312E-04	6.1678E-01	1.7806E-01
CCNA_00566	5.7626E-05	7.3788E-05	-3.5672E-01	-4.4288E-01
CCNA_00567	1.0762E-04	1.6269E-04	-5.9618E-01	-5.9562E-01
CCNA_00568	6.7480E-05	1.5053E-04	-1.1575E+00	-9.5368E-01
CCNA_00569	7.7098E-05	5.4438E-05	5.0196E-01	1.0482E-01
CCNA_00570	4.8942E-05	0.0000E+00	1.2528E+01	7.7756E+00
CCNA_00571	7.4897E-05	1.2157E-05	2.6222E+00	1.4572E+00
CCNA_00572	0.0000E+00	0.0000E+00	-8.4168E-01	-7.5221E-01
CCNA_00573	4.4170E-05	0.0000E+00	1.2380E+01	7.6812E+00
CCNA_00574	6.0840E-05	2.3519E-05	1.3708E+00	6.5901E-01
CCNA_00575	5.4588E-05	3.2908E-05	7.2990E-01	2.5021E-01
CCNA_00576	7.5641E-05	1.0835E-04	-5.1852E-01	-5.4608E-01
CCNA_00577	6.0266E-05	5.6692E-05	8.8110E-02	-1.5915E-01
CCNA_00578	4.9265E-05	1.4099E-04	-1.5169E+00	-1.1829E+00
CCNA_00579	5.1439E-05	9.1746E-05	-8.3479E-01	-7.4782E-01
CCNA_00580	7.2770E-05	1.4674E-04	-1.0118E+00	-8.6074E-01
CCNA_04005	7.5590E-05	3.7283E-05	1.0194E+00	4.3489E-01

CCNA_00582	1.6265E-04	1.1489E-04	5.0146E-01	1.0450E-01
CCNA_00583	7.7366E-05	3.3248E-05	1.2182E+00	5.6165E-01
CCNA_00584	8.4279E-05	7.3912E-05	1.8928E-01	-9.4620E-02
CCNA_00585	7.4763E-05	4.3905E-05	7.6776E-01	2.7436E-01
CCNA_00586	1.1419E-04	1.0627E-04	1.0358E-01	-1.4928E-01
CCNA_00587	1.1643E-04	1.1331E-04	3.9163E-02	-1.9037E-01
CCNA_00588	8.2638E-05	1.1378E-04	-4.6142E-01	-5.0966E-01
CCNA_00589	7.8832E-05	3.9861E-05	9.8360E-01	4.1203E-01
CCNA_00590	8.0450E-05	8.9185E-05	-1.4876E-01	-3.1023E-01
CCNA_00591	6.7077E-05	7.6879E-05	-1.9682E-01	-3.4089E-01
CCNA_00592	1.3943E-04	1.2394E-04	1.6980E-01	-1.0705E-01
CCNA_00593	6.3078E-05	1.2487E-04	-9.8521E-01	-8.4376E-01
CCNA_00594	1.6502E-04	1.6204E-04	2.6222E-02	-1.9863E-01
CCNA_00595	1.0626E-04	8.4677E-05	3.2751E-01	-6.4533E-03
CCNA_00596	1.7912E-04	1.1120E-04	6.8769E-01	2.2328E-01
CCNA_00597	1.9888E-04	1.5861E-04	3.2644E-01	-7.1375E-03
CCNA_00598	5.6609E-04	1.6276E-04	1.7983E+00	9.3165E-01
CCNA_00600	6.2463E-05	1.3413E-04	-1.1026E+00	-9.1863E-01
CCNA_00601	7.1766E-05	9.7638E-05	-4.4417E-01	-4.9866E-01
CCNA_00602	5.1920E-05	1.3084E-04	-1.3334E+00	-1.0658E+00
CCNA_00603	1.6863E-04	9.6536E-05	8.0462E-01	2.9787E-01
CCNA_00604	6.1408E-05	1.2326E-04	-1.0052E+00	-8.5651E-01
CCNA_00605	1.5842E-04	1.3765E-04	2.0275E-01	-8.6030E-02
CCNA_00606	7.8439E-05	5.7728E-05	4.4219E-01	6.6694E-02
CCNA_00607	8.0025E-05	4.4319E-05	8.5233E-01	3.2830E-01
CCNA_00608	7.3519E-05	2.1812E-05	1.7526E+00	9.0251E-01
CCNA_00609	2.2972E-04	1.1787E-04	9.6266E-01	3.9867E-01
CCNA_00610	1.0925E-04	1.5712E-04	-5.2422E-01	-5.4972E-01
CCNA_00611	1.5656E-04	1.4645E-04	9.6270E-02	-1.5395E-01
CCNA_00612	7.8374E-05	1.2180E-04	-6.3611E-01	-6.2109E-01
CCNA_00613	8.1699E-05	9.1671E-05	-1.6620E-01	-3.2136E-01
CCNA_00614	8.2739E-05	1.4429E-04	-8.0237E-01	-7.2714E-01
CCNA_00615	2.6830E-04	7.5454E-05	1.8300E+00	9.5192E-01
CCNA_00616	6.9621E-05	9.6702E-05	-4.7405E-01	-5.1772E-01
CCNA_00617	8.1065E-05	5.0103E-05	6.9402E-01	2.2732E-01
CCNA_00618	7.3366E-05	1.5590E-04	-1.0874E+00	-9.0893E-01
CCNA_00619	5.7496E-05	1.4211E-04	-1.3054E+00	-1.0480E+00
CCNA_00620	6.5501E-05	1.4041E-04	-1.1000E+00	-9.1699E-01
CCNA_00621	2.4392E-04	1.2587E-04	9.5437E-01	3.9339E-01
CCNA_00622	2.1387E-04	1.7833E-04	2.6213E-01	-4.8156E-02
CCNA_00623	2.0396E-04	1.6065E-04	3.4430E-01	4.2599E-03
CCNA_00624	3.7811E-04	1.6250E-04	1.2183E+00	5.6174E-01
CCNA_00625	3.9946E-04	1.6120E-04	1.3092E+00	6.1968E-01
CCNA_00626	1.0172E-04	8.0824E-05	3.3163E-01	-3.8261E-03
CCNA_00627	9.8780E-05	6.6959E-05	5.6083E-01	1.4237E-01
CCNA_00628	7.9160E-05	8.7793E-05	-1.4938E-01	-3.1063E-01
CCNA_00629	5.3182E-05	2.1911E-05	1.2789E+00	6.0036E-01
CCNA_00630	8.0631E-05	9.3644E-05	-2.1590E-01	-3.5306E-01
CCNA_00631	8.4705E-05	1.3766E-04	-7.0056E-01	-6.6220E-01
CCNA_00632	1.2890E-04	1.6223E-04	-3.3176E-01	-4.2696E-01
CCNA_00633	8.8663E-05	6.6595E-05	4.1282E-01	4.7961E-02
CCNA_00634	8.9555E-05	1.2279E-04	-4.5536E-01	-5.0580E-01
CCNA_00635	1.0355E-04	1.3312E-04	-3.6249E-01	-4.4656E-01

CCNA_00636	1.1939E-04	8.7578E-05	4.4703E-01	6.9781E-02
CCNA_00637	7.4629E-05	5.9286E-05	3.3194E-01	-3.6281E-03
CCNA_00638	1.0567E-04	1.6243E-04	-6.2022E-01	-6.1096E-01
CCNA_00639	2.8456E-04	1.0873E-04	1.3879E+00	6.6994E-01
CCNA_00640	1.6874E-04	5.6377E-05	1.5815E+00	7.9339E-01
CCNA_00641	2.4155E-04	1.0609E-04	1.1869E+00	5.4171E-01
CCNA_00642	2.2995E-04	7.5180E-05	1.6128E+00	8.1333E-01
CCNA_00643	1.3447E-04	1.6269E-04	-2.7488E-01	-3.9068E-01
CCNA_00644	6.9732E-05	1.1202E-04	-6.8394E-01	-6.5159E-01
CCNA_00645	7.0879E-05	1.5348E-04	-1.1147E+00	-9.2633E-01
CCNA_00646	5.0722E-05	1.1778E-04	-1.2153E+00	-9.9054E-01
CCNA_00647	6.8141E-05	4.3689E-05	6.4107E-01	1.9355E-01
CCNA_00648	6.6472E-05	9.2218E-05	-4.7234E-01	-5.1663E-01
CCNA_00649	5.7704E-05	1.1841E-04	-1.0371E+00	-8.7684E-01
CCNA_00650	6.6476E-05	5.9294E-05	1.6486E-01	-1.1020E-01
CCNA_00651	6.5561E-05	8.3741E-05	-3.5314E-01	-4.4060E-01
CCNA_00652	6.6282E-05	1.4931E-04	-1.1716E+00	-9.6263E-01
CCNA_00653	6.8673E-05	4.1095E-05	7.4057E-01	2.5701E-01
CCNA_00654	5.8597E-05	7.6523E-05	-3.8511E-01	-4.6099E-01
CCNA_00655	1.6368E-04	1.6383E-04	-1.3569E-03	-2.1622E-01
CCNA_00656	1.8564E-04	1.8323E-04	1.8848E-02	-2.0333E-01
CCNA_00657	7.7422E-05	1.2761E-04	-7.2097E-01	-6.7521E-01
CCNA_00658	7.7981E-05	3.5452E-05	1.1370E+00	5.0988E-01
CCNA_00659	4.1437E-05	1.0821E-04	-1.3848E+00	-1.0987E+00
CCNA_00660	4.0508E-04	2.6728E-04	5.9981E-01	1.6723E-01
CCNA_00661	1.3089E-04	1.4931E-04	-1.9001E-01	-3.3655E-01
CCNA_00662	8.1264E-05	8.7263E-05	-1.0280E-01	-2.8092E-01
CCNA_00663	7.6099E-05	1.6013E-04	-1.0733E+00	-8.9994E-01
CCNA_00664	9.9155E-05	6.4390E-05	6.2272E-01	1.8185E-01
CCNA_00665	9.6707E-04	1.6010E-04	2.5946E+00	1.4396E+00
CCNA_00666	6.7424E-05	9.1298E-05	-4.3735E-01	-4.9431E-01
CCNA_00667	5.9961E-05	3.3712E-05	8.3053E-01	3.1440E-01
CCNA_00668	5.4287E-05	6.5476E-05	-2.7041E-01	-3.8783E-01
CCNA_00669	7.6034E-05	1.3631E-04	-8.4221E-01	-7.5255E-01
CCNA_00670	8.8251E-05	4.3739E-05	1.0125E+00	4.3046E-01
CCNA_00671	7.3306E-05	1.4288E-04	-9.6276E-01	-8.2944E-01
CCNA_00672	9.9150E-05	1.1388E-04	-1.9987E-01	-3.4284E-01
CCNA_00673	9.9169E-05	1.5241E-04	-6.1998E-01	-6.1080E-01
CCNA_00674	9.6912E-05	1.3453E-04	-4.7322E-01	-5.1719E-01
CCNA_00675	1.4387E-04	7.0829E-05	1.0223E+00	4.3670E-01
CCNA_00676	1.4608E-04	1.6022E-04	-1.3334E-01	-3.0040E-01
CCNA_00677	8.5088E-05	8.7039E-05	-3.2756E-02	-2.3624E-01
CCNA_00678	8.0071E-05	1.6756E-04	-1.0653E+00	-8.9483E-01
CCNA_00679	4.7886E-04	7.8352E-04	-7.1037E-01	-6.6846E-01
CCNA_00680	5.2581E-05	4.8827E-05	1.0673E-01	-1.4727E-01
CCNA_00681	7.2594E-05	1.4603E-04	-1.0084E+00	-8.5854E-01
CCNA_00682	6.3711E-05	8.7602E-05	-4.5946E-01	-5.0841E-01
CCNA_00683	7.2178E-05	4.5529E-05	6.6460E-01	2.0856E-01
CCNA_00684	8.4885E-05	6.3935E-05	4.0880E-01	4.5401E-02
CCNA_00685	2.5453E-04	1.5295E-04	7.3466E-01	2.5325E-01
CCNA_00686	2.7039E-04	1.5439E-04	8.0844E-01	3.0030E-01
CCNA_00687	1.2495E-04	1.2419E-04	8.7965E-03	-2.0974E-01
CCNA_00688	8.1893E-05	8.9682E-05	-1.3113E-01	-2.9899E-01

CCNA_00689	8.2342E-05	9.4215E-05	-1.9439E-01	-3.3934E-01
CCNA_00690	2.9600E-04	1.3230E-04	1.1617E+00	5.2562E-01
CCNA_00691	1.3613E-04	1.6243E-04	-2.5478E-01	-3.7786E-01
CCNA_00692	1.3595E-04	1.5928E-04	-2.2850E-01	-3.6110E-01
CCNA_00693	7.8004E-05	8.8431E-05	-1.8105E-01	-3.3083E-01
CCNA_00694	6.4701E-05	2.6552E-05	1.2846E+00	6.0404E-01
CCNA_00695	1.0031E-04	8.2663E-05	2.7908E-01	-3.7341E-02
CCNA_00696	9.9506E-05	4.5256E-05	1.1365E+00	5.0955E-01
CCNA_00697	1.3210E-03	1.5595E-04	3.0824E+00	1.7507E+00
CCNA_00698	2.3278E-04	2.4605E-04	-7.9991E-02	-2.6637E-01
CCNA_00699	2.9284E-04	3.2485E-04	-1.4968E-01	-3.1082E-01
CCNA_00700	7.5688E-05	9.2077E-05	-2.8283E-01	-3.9575E-01
CCNA_00701	8.1754E-05	9.5160E-05	-2.1911E-01	-3.5511E-01
CCNA_00702	9.3842E-05	7.2744E-05	3.6732E-01	1.8938E-02
CCNA_00703	7.9244E-05	1.2873E-04	-7.0000E-01	-6.6184E-01
CCNA_00704	1.0094E-04	8.0807E-05	3.2086E-01	-1.0692E-02
CCNA_00705	8.8922E-05	1.5319E-04	-7.8476E-01	-7.1590E-01
CCNA_00706	7.7736E-05	1.6219E-04	-1.0610E+00	-8.9209E-01
CCNA_00707	6.0923E-05	8.6111E-05	-4.9924E-01	-5.3379E-01
CCNA_00708	6.9218E-05	5.0021E-05	4.6849E-01	8.3472E-02
CCNA_00709	8.0645E-05	3.3355E-05	1.2734E+00	5.9687E-01
CCNA_00710	7.1970E-05	1.2466E-04	-7.9256E-01	-7.2088E-01
CCNA_00711	7.8934E-05	1.0293E-04	-3.8303E-01	-4.5966E-01
CCNA_00712	9.2260E-05	1.6243E-04	-8.1600E-01	-7.3583E-01
CCNA_00713	7.5424E-05	6.6810E-05	1.7487E-01	-1.0382E-01
CCNA_00714	1.1531E-04	1.6665E-05	2.7900E+00	1.5642E+00
CCNA_00715	8.6402E-05	6.1863E-05	4.8187E-01	9.2006E-02
CCNA_00716	1.6835E-04	8.6053E-05	9.6811E-01	4.0215E-01
CCNA_00717	1.9098E-04	6.4515E-05	1.5655E+00	7.8322E-01
CCNA_00718	1.9346E-04	1.3877E-04	4.7932E-01	9.0377E-02
CCNA_00719	1.8747E-04	3.4797E-05	2.4293E+00	1.3342E+00
CCNA_00720	5.1268E-05	8.8116E-05	-7.8136E-01	-7.1374E-01
CCNA_00721	9.1729E-05	8.0857E-05	1.8193E-01	-9.9309E-02
CCNA_00722	1.2574E-04	1.5040E-04	-2.5841E-01	-3.8018E-01
CCNA_00723	1.2595E-04	1.3819E-04	-1.3378E-01	-3.0068E-01
CCNA_00724	1.1170E-04	1.3075E-04	-2.2730E-01	-3.6034E-01
CCNA_00725	4.2916E-05	1.0771E-04	-1.3275E+00	-1.0621E+00
CCNA_00726	1.0443E-04	1.6273E-04	-6.3996E-01	-6.2355E-01
CCNA_00727	9.6755E-05	1.4065E-04	-5.3969E-01	-5.5959E-01
CCNA_00728	1.2696E-04	1.5347E-04	-2.7359E-01	-3.8986E-01
CCNA_00729	1.4553E-04	1.6054E-04	-1.4164E-01	-3.0570E-01
CCNA_00730	7.8083E-05	4.1095E-05	9.2582E-01	3.7518E-01
CCNA_00731	1.7186E-04	4.6151E-05	1.8966E+00	9.9435E-01
CCNA_00732	1.4270E-04	7.5371E-05	9.2085E-01	3.7200E-01
CCNA_00733	8.0196E-05	1.3138E-04	-7.1218E-01	-6.6961E-01
CCNA_00734	8.1662E-05	1.2174E-05	2.7450E+00	1.5355E+00
CCNA_00735	3.1439E-04	1.6277E-04	9.4972E-01	3.9042E-01
CCNA_00736	7.1327E-05	1.0943E-04	-6.1751E-01	-6.0922E-01
CCNA_00737	1.4044E-04	1.6276E-04	-2.1276E-01	-3.5106E-01
CCNA_00738	6.1501E-05	9.9014E-05	-6.8703E-01	-6.5357E-01
CCNA_00739	4.0091E-05	7.5222E-05	-9.0786E-01	-7.9442E-01
CCNA_00740	7.9507E-05	2.2963E-05	1.7913E+00	9.2722E-01
CCNA_00741	2.6222E-04	1.4223E-04	8.8249E-01	3.4754E-01

CCNA_00742	7.9410E-05	8.0882E-05	-2.6554E-02	-2.3229E-01
CCNA_00743	9.7694E-05	5.9277E-05	7.2065E-01	2.4431E-01
CCNA_00744	9.6219E-05	0.0000E+00	1.3503E+01	8.3976E+00
CCNA_00745	5.3820E-05	1.1169E-04	-1.0533E+00	-8.8719E-01
CCNA_00746	8.3983E-05	1.4006E-04	-7.3787E-01	-6.8600E-01
CCNA_00747	3.4224E-04	1.4311E-04	1.2578E+00	5.8695E-01
CCNA_00748	3.3216E-04	1.3816E-04	1.2654E+00	5.9179E-01
CCNA_00749	1.0648E-04	9.9436E-05	9.8675E-02	-1.5241E-01
CCNA_00750	6.8307E-05	1.1242E-04	-7.1882E-01	-6.7384E-01
CCNA_00751	7.7625E-05	1.5849E-04	-1.0298E+00	-8.7219E-01
CCNA_00752	2.1274E-04	8.3020E-05	1.3574E+00	6.5048E-01
CCNA_00753	7.8411E-05	1.1204E-05	2.8061E+00	1.5745E+00
CCNA_00754	7.9553E-04	6.4432E-05	3.6259E+00	2.0974E+00
CCNA_00755	7.2802E-05	1.1169E-04	-6.1750E-01	-6.0922E-01
CCNA_00756	8.2980E-05	1.6243E-04	-9.6894E-01	-8.3339E-01
CCNA_00757	1.3704E-04	1.4564E-04	-8.7887E-02	-2.7141E-01
CCNA_00758	1.2530E-04	1.6243E-04	-3.7447E-01	-4.5421E-01
CCNA_00759	1.2279E-04	8.3683E-05	5.5315E-01	1.3747E-01
CCNA_00760	1.6309E-04	7.8313E-05	1.0583E+00	4.5965E-01
CCNA_00761	1.3195E-04	1.6275E-04	-3.0264E-01	-4.0839E-01
CCNA_00762	5.9989E-05	5.9700E-05	6.8733E-03	-2.1097E-01
CCNA_00763	7.0957E-05	1.4810E-04	-1.0615E+00	-8.9243E-01
CCNA_00764	2.2104E-04	2.3931E-04	-1.1460E-01	-2.8845E-01
CCNA_00765	2.1997E-04	2.3559E-04	-9.8981E-02	-2.7849E-01
CCNA_00766	2.9586E-04	1.2760E-04	1.2131E+00	5.5845E-01
CCNA_00767	2.4149E-04	2.6413E-04	-1.2929E-01	-2.9782E-01
CCNA_00768	1.8588E-04	2.0143E-04	-1.1589E-01	-2.8927E-01
CCNA_00769	8.2901E-05	9.8806E-05	-2.5325E-01	-3.7689E-01
CCNA_00770	6.9866E-05	4.7162E-05	5.6682E-01	1.4619E-01
CCNA_00771	5.8953E-05	3.2452E-05	8.6100E-01	3.3383E-01
CCNA_00772	6.3244E-05	8.9989E-05	-5.0885E-01	-5.3992E-01
CCNA_00773	6.0969E-05	4.3333E-05	4.9244E-01	9.8750E-02
CCNA_00774	6.5367E-05	1.3283E-04	-1.0230E+00	-8.6785E-01
CCNA_00775	4.3712E-05	1.1486E-05	1.9273E+00	1.0139E+00
CCNA_00776	1.0133E-04	1.0522E-04	-5.4428E-02	-2.5007E-01
CCNA_00777	1.5213E-04	1.0249E-04	5.6968E-01	1.4801E-01
CCNA_00778	3.5207E-04	3.2475E-04	1.1649E-01	-1.4105E-01
CCNA_00779	2.8860E-04	1.5256E-04	9.1965E-01	3.7124E-01
CCNA_00780	2.9405E-04	2.7920E-04	7.4737E-02	-1.6768E-01
CCNA_00781	2.7507E-04	2.5032E-04	1.3601E-01	-1.2860E-01
CCNA_00782	3.8310E-04	1.7333E-04	1.1441E+00	5.1443E-01
CCNA_00783	1.3482E-04	1.2695E-04	8.6697E-02	-1.6005E-01
CCNA_00784	1.1730E-04	9.7373E-05	2.6860E-01	-4.4025E-02
CCNA_00785	4.0179E-05	1.9226E-05	1.0629E+00	4.6263E-01
CCNA_04003	1.5541E-04	1.2298E-04	3.3758E-01	-2.9089E-05
CCNA_00787	2.3044E-04	2.2100E-04	6.0345E-02	-1.7686E-01
CCNA_00788	2.5251E-04	1.7472E-04	5.3122E-01	1.2348E-01
CCNA_00789	1.4127E-03	1.6284E-04	3.1168E+00	1.7727E+00
CCNA_00790	1.3149E-03	1.6479E-04	2.9962E+00	1.6957E+00
CCNA_00791	6.5667E-05	9.3122E-05	-5.0397E-01	-5.3681E-01
CCNA_00792	2.8683E-04	1.4261E-04	1.0081E+00	4.2763E-01
CCNA_00793	4.9551E-04	9.3287E-05	2.4090E+00	1.3212E+00
CCNA_00794	6.8936E-05	3.0969E-05	1.1542E+00	5.2082E-01

CCNA_00795	2.0202E-04	5.3277E-05	1.9227E+00	1.0110E+00
CCNA_00796	8.3871E-04	6.9860E-05	3.5855E+00	2.0716E+00
CCNA_00797	4.2846E-04	1.3795E-04	1.6350E+00	8.2750E-01
CCNA_00798	7.2215E-05	4.0358E-05	8.3924E-01	3.1995E-01
CCNA_00799	1.1311E-03	1.2152E-04	3.2183E+00	1.8374E+00
CCNA_00800	1.1697E-03	9.4406E-05	3.6310E+00	2.1006E+00
CCNA_00801	1.0028E-04	1.4008E-04	-4.8223E-01	-5.2294E-01
CCNA_00802	1.2665E-04	9.4000E-05	4.3009E-01	5.8980E-02
CCNA_00803	2.9007E-04	2.5327E-04	1.9571E-01	-9.0518E-02
CCNA_00804	7.0985E-05	1.4487E-04	-1.0292E+00	-8.7182E-01
CCNA_00805	5.8430E-05	1.0220E-04	-8.0655E-01	-7.2980E-01
CCNA_00806	1.0397E-04	1.6258E-04	-6.4497E-01	-6.2674E-01
CCNA_00807	2.2233E-04	1.5902E-04	4.8343E-01	9.3002E-02
CCNA_00808	2.8131E-04	2.0409E-04	4.6289E-01	7.9898E-02
CCNA_00809	4.6089E-05	3.8071E-05	2.7556E-01	-3.9588E-02
CCNA_00810	6.5084E-05	1.0881E-04	-7.4142E-01	-6.8826E-01
CCNA_00811	9.8725E-05	1.2122E-04	-2.9621E-01	-4.0428E-01
CCNA_00812	5.4278E-05	4.3010E-06	3.6550E+00	2.1159E+00
CCNA_00813	6.0881E-05	5.6485E-05	1.0804E-01	-1.4644E-01
CCNA_00814	1.2710E-04	1.4322E-04	-1.7228E-01	-3.2524E-01
CCNA_00815	5.4754E-05	2.7074E-05	1.0158E+00	4.3254E-01
CCNA_00816	5.3996E-05	4.4253E-05	2.8693E-01	-3.2334E-02
CCNA_00817	1.0473E-04	5.2184E-05	1.0048E+00	4.2555E-01
CCNA_00818	5.2428E-05	7.0481E-05	-4.2694E-01	-4.8767E-01
CCNA_00819	5.3321E-05	7.5768E-05	-5.0693E-01	-5.3870E-01
CCNA_00820	5.7774E-05	3.1267E-05	8.8550E-01	3.4946E-01
CCNA_00821	8.8117E-05	1.2489E-04	-5.0313E-01	-5.3627E-01
CCNA_00822	8.1232E-05	1.4917E-04	-8.7681E-01	-7.7462E-01
CCNA_00823	9.8434E-05	1.0699E-04	-1.2024E-01	-2.9205E-01
CCNA_00824	1.0789E-04	1.3757E-04	-3.5073E-01	-4.3906E-01
CCNA_00825	6.1112E-05	1.0864E-04	-8.3006E-01	-7.4480E-01
CCNA_00826	1.6000E-04	1.4065E-04	1.8598E-01	-9.6728E-02
CCNA_00827	2.1242E-04	8.6741E-05	1.2921E+00	6.0877E-01
CCNA_00828	7.2895E-05	9.4307E-05	-3.7158E-01	-4.5236E-01
CCNA_00829	7.4379E-05	5.2788E-05	4.9453E-01	1.0008E-01
CCNA_00830	7.5979E-05	9.1895E-05	-2.7443E-01	-3.9040E-01
CCNA_00831	5.6946E-05	7.2031E-05	-3.3907E-01	-4.3163E-01
CCNA_00832	9.7157E-05	3.6786E-05	1.4009E+00	6.7820E-01
CCNA_00833	1.0155E-04	6.7705E-05	5.8468E-01	1.5758E-01
CCNA_00834	1.3883E-04	3.4043E-05	2.0276E+00	1.0780E+00
CCNA_00835	8.0242E-05	5.1380E-07	7.2640E+00	4.4179E+00
CCNA_00836	1.0213E-04	5.0642E-05	1.0119E+00	4.3006E-01
CCNA_00837	4.1127E-05	0.0000E+00	1.2277E+01	7.6155E+00
CCNA_00838	1.5682E-04	1.8596E-05	3.0755E+00	1.7463E+00
CCNA_00839	1.5697E-04	2.3154E-05	2.7607E+00	1.5455E+00
CCNA_00840	4.3457E-05	9.2160E-05	-1.0845E+00	-9.0710E-01
CCNA_00841	5.9591E-05	1.1400E-04	-9.3592E-01	-8.1232E-01
CCNA_00842	1.5341E-04	1.5143E-04	1.8668E-02	-2.0344E-01
CCNA_00843	6.6837E-05	5.3112E-05	3.3149E-01	-3.9116E-03
CCNA_00844	1.0378E-04	1.4095E-04	-4.4167E-01	-4.9707E-01
CCNA_00845	1.0184E-04	9.8848E-05	4.2924E-02	-1.8797E-01
CCNA_00846	4.6050E-04	1.7076E-04	1.4312E+00	6.9750E-01
CCNA_00847	8.5607E-04	2.8073E-04	1.6085E+00	8.1062E-01

CCNA_00848	5.5272E-05	7.4525E-05	-4.3122E-01	-4.9040E-01
CCNA_00849	6.1390E-05	1.4447E-04	-1.2347E+00	-1.0029E+00
CCNA_00850	6.4035E-05	7.4658E-05	-2.2150E-01	-3.5663E-01
CCNA_00851	1.3863E-04	1.7189E-04	-3.1026E-01	-4.1325E-01
CCNA_00852	1.3243E-04	1.7025E-04	-3.6244E-01	-4.4653E-01
CCNA_00853	7.3964E-04	1.1607E-04	2.6718E+00	1.4888E+00
CCNA_00854	7.3433E-04	7.6995E-05	3.2534E+00	1.8598E+00
CCNA_00855	4.5192E-05	1.2143E-04	-1.4260E+00	-1.1249E+00
CCNA_00856	4.9936E-05	1.2789E-04	-1.3568E+00	-1.0808E+00
CCNA_00857	1.9638E-04	1.0639E-04	8.8420E-01	3.4863E-01
CCNA_00858	1.7759E-04	1.1831E-04	5.8586E-01	1.5833E-01
CCNA_00859	3.6297E-04	1.5060E-04	1.2690E+00	5.9410E-01
CCNA_00860	5.2040E-04	2.1358E-04	1.2848E+00	6.0414E-01
CCNA_00861	1.0649E-04	3.9098E-05	1.4453E+00	7.0651E-01
CCNA_00862	6.9200E-05	1.0307E-04	-5.7475E-01	-5.8195E-01
CCNA_00863	1.0445E-04	5.2109E-05	1.0030E+00	4.2443E-01
CCNA_00864	1.4303E-04	1.2738E-04	1.6712E-01	-1.0876E-01
CCNA_00865	6.2342E-05	1.2043E-04	-9.4987E-01	-8.2122E-01
CCNA_00866	4.7405E-04	5.9683E-05	2.9895E+00	1.6914E+00
CCNA_00867	4.7626E-04	5.7728E-05	3.0442E+00	1.7264E+00
CCNA_04017	5.2937E-05	3.7573E-05	4.9437E-01	9.9977E-02
CCNA_00869	2.8544E-04	4.5363E-05	2.6534E+00	1.4771E+00
CCNA_00870	5.8865E-05	9.4290E-05	-6.7971E-01	-6.4890E-01
CCNA_00871	9.7250E-05	6.7208E-05	5.3295E-01	1.2459E-01
CCNA_00872	1.0002E-04	9.7323E-05	3.9374E-02	-1.9024E-01
CCNA_00873	6.4049E-05	5.2921E-05	2.7520E-01	-3.9815E-02
CCNA_00874	6.0955E-05	3.9728E-05	6.1740E-01	1.7845E-01
CCNA_00875	9.3689E-05	2.4140E-05	1.9560E+00	1.0323E+00
CCNA_00876	5.9910E-05	1.4689E-04	-1.2938E+00	-1.0406E+00
CCNA_00877	1.6074E-04	2.9326E-04	-8.6747E-01	-7.6866E-01
CCNA_00878	1.5483E-04	2.6848E-04	-7.9407E-01	-7.2184E-01
CCNA_00879	3.4491E-05	7.0108E-05	-1.0233E+00	-8.6808E-01
CCNA_00880	5.3085E-05	1.5803E-05	1.7474E+00	8.9924E-01
CCNA_00881	5.8481E-05	4.5396E-05	3.6525E-01	1.7617E-02
CCNA_00882	4.9196E-05	1.1187E-04	-1.1851E+00	-9.7128E-01
CCNA_00883	8.0848E-05	1.2686E-04	-6.4994E-01	-6.2991E-01
CCNA_00884	7.9461E-05	9.4307E-05	-2.4716E-01	-3.7300E-01
CCNA_00885	1.7104E-04	1.2685E-04	4.3113E-01	5.9642E-02
CCNA_00886	1.7026E-04	1.2796E-04	4.1203E-01	4.7458E-02
CCNA_00887	6.3938E-05	1.2326E-04	-9.4698E-01	-8.1937E-01
CCNA_00888	9.4679E-05	5.2084E-05	8.6204E-01	3.3449E-01
CCNA_00889	2.9064E-04	1.1873E-04	1.2915E+00	6.0840E-01
CCNA_00890	6.4576E-05	5.8208E-05	1.4967E-01	-1.1989E-01
CCNA_00891	6.4266E-05	3.5510E-05	8.5560E-01	3.3038E-01
CCNA_00892	2.1788E-04	1.1693E-04	8.9781E-01	3.5731E-01
CCNA_00893	1.8637E-04	1.6272E-04	1.9574E-01	-9.0499E-02
CCNA_00894	1.6778E-04	1.3989E-04	2.6225E-01	-4.8075E-02
CCNA_00895	8.2295E-05	9.4157E-05	-1.9431E-01	-3.3929E-01
CCNA_00896	8.4117E-05	1.1632E-04	-4.6761E-01	-5.1361E-01
CCNA_00897	9.9895E-05	1.6115E-04	-6.8993E-01	-6.5542E-01
CCNA_00898	8.8506E-05	6.5302E-05	4.3854E-01	6.4365E-02
CCNA_00899	9.5123E-05	1.3151E-04	-4.6730E-01	-5.1341E-01
CCNA_00900	9.5141E-05	1.3190E-04	-4.7138E-01	-5.1602E-01

CCNA_00901	1.9182E-04	5.3833E-05	1.8330E+00	9.5381E-01
CCNA_00902	1.6082E-04	9.6710E-06	4.0544E+00	2.3707E+00
CCNA_00903	1.0291E-04	1.0282E-04	1.3089E-03	-2.1452E-01
CCNA_00904	6.0983E-05	1.6040E-04	-1.3952E+00	-1.1053E+00
CCNA_00905	6.6809E-05	1.4278E-04	-1.0956E+00	-9.1419E-01
CCNA_00906	8.6411E-05	9.7994E-05	-1.8153E-01	-3.3114E-01
CCNA_00907	1.3347E-04	5.6799E-05	1.2324E+00	5.7070E-01
CCNA_00908	5.2757E-05	1.2453E-04	-1.2390E+00	-1.0057E+00
CCNA_00909	6.8548E-05	1.5470E-04	-1.1743E+00	-9.6436E-01
CCNA_00910	8.0506E-05	5.8225E-05	4.6734E-01	8.2737E-02
CCNA_00911	7.8161E-05	4.7941E-05	7.0505E-01	2.3436E-01
CCNA_00912	5.8652E-05	1.0058E-04	-7.7809E-01	-7.1165E-01
CCNA_00913	6.6194E-05	1.7809E-05	1.8935E+00	9.9243E-01
CCNA_00914	1.4502E-04	5.1935E-05	1.4813E+00	7.2946E-01
CCNA_00915	1.3231E-04	1.0640E-04	3.1445E-01	-1.4784E-02
CCNA_00916	6.7706E-05	1.3171E-04	-9.6004E-01	-8.2771E-01
CCNA_00917	6.9547E-05	6.0868E-05	1.9219E-01	-9.2765E-02
CCNA_00918	7.2594E-05	7.4153E-05	-3.0714E-02	-2.3494E-01
CCNA_00919	2.1440E-04	1.6835E-04	3.4877E-01	7.1094E-03
CCNA_00920	2.1888E-04	2.3375E-04	-9.4877E-02	-2.7587E-01
CCNA_00921	4.8461E-05	8.4909E-05	-8.0910E-01	-7.3143E-01
CCNA_00922	4.8097E-04	1.3180E-04	1.8675E+00	9.7585E-01
CCNA_00923	5.1346E-05	6.9968E-05	-4.4647E-01	-5.0013E-01
CCNA_00924	4.8391E-05	5.0311E-05	-5.6213E-02	-2.5121E-01
CCNA_00925	1.3787E-04	5.9534E-05	1.2113E+00	5.5730E-01
CCNA_00926	4.2436E-05	8.7934E-05	-1.0511E+00	-8.8580E-01
CCNA_00927	4.5145E-05	3.5145E-05	3.6105E-01	1.4940E-02
CCNA_00928	1.7721E-04	1.6093E-04	1.3906E-01	-1.2665E-01
CCNA_00929	2.0818E-04	1.7303E-04	2.6673E-01	-4.5221E-02
CCNA_00930	4.2491E-05	9.9884E-05	-1.2331E+00	-1.0018E+00
CCNA_00931	8.7659E-05	1.6243E-04	-8.8980E-01	-7.8290E-01
CCNA_00932	5.4685E-05	6.7854E-05	-3.1135E-01	-4.1395E-01
CCNA_00933	7.1961E-05	5.9708E-05	2.6917E-01	-4.3664E-02
CCNA_00934	4.3273E-05	9.0246E-06	2.2603E+00	1.2264E+00
CCNA_00935	5.6345E-05	8.6724E-05	-6.2217E-01	-6.1219E-01
CCNA_00936	5.4662E-05	5.4695E-05	-9.6206E-04	-2.1597E-01
CCNA_00937	6.0853E-05	3.8651E-05	6.5464E-01	2.0221E-01
CCNA_00938	1.3672E-04	1.6521E-04	-2.7309E-01	-3.8954E-01
CCNA_00939	1.4831E-04	1.8330E-04	-3.0558E-01	-4.1026E-01
CCNA_00940	8.7678E-05	5.7040E-05	6.2011E-01	1.8018E-01
CCNA_00941	1.2419E-04	1.0646E-04	2.2218E-01	-7.3634E-02
CCNA_00942	5.9822E-05	8.4627E-05	-5.0047E-01	-5.3457E-01
CCNA_00943	6.3975E-05	4.0076E-05	6.7456E-01	2.1491E-01
CCNA_00944	4.2842E-05	9.6561E-05	-1.1724E+00	-9.6313E-01
CCNA_00945	2.9974E-05	8.3127E-05	-1.4716E+00	-1.1540E+00
CCNA_00946	6.1270E-05	7.5909E-05	-3.0916E-01	-4.1254E-01
CCNA_00947	6.9866E-05	1.5206E-04	-1.1220E+00	-9.3098E-01
CCNA_00948	7.9974E-05	1.0425E-05	2.9384E+00	1.6589E+00
CCNA_00949	7.1785E-05	1.0754E-04	-5.8315E-01	-5.8731E-01
CCNA_00950	8.1482E-05	1.0461E-04	-3.6047E-01	-4.4528E-01
CCNA_00951	1.0982E-04	6.7647E-05	6.9890E-01	2.3044E-01
CCNA_00952	7.5799E-05	1.0080E-04	-4.1122E-01	-4.7764E-01
CCNA_00953	5.0694E-05	2.7662E-05	8.7361E-01	3.4187E-01

CCNA_00954	5.5901E-05	1.4189E-04	-1.3438E+00	-1.0725E+00
CCNA_00955	1.0179E-04	6.9528E-05	5.4983E-01	1.3535E-01
CCNA_00956	7.4189E-05	1.3858E-04	-9.0139E-01	-7.9030E-01
CCNA_00957	6.1630E-05	1.5009E-04	-1.2841E+00	-1.0344E+00
CCNA_00958	2.0714E-04	1.4584E-04	5.0623E-01	1.0754E-01
CCNA_00959	1.9550E-04	1.2087E-04	6.9358E-01	2.2704E-01
CCNA_00960	1.6956E-04	1.2605E-04	4.2781E-01	5.7521E-02
CCNA_00961	1.7390E-04	1.4350E-04	2.7717E-01	-3.8560E-02
CCNA_00962	6.3716E-05	1.0935E-04	-7.7920E-01	-7.1236E-01
CCNA_00963	7.9336E-05	1.6243E-04	-1.0337E+00	-8.7471E-01
CCNA_00964	4.2241E-05	1.6848E-05	1.3256E+00	6.3015E-01
CCNA_00965	1.5868E-04	1.3281E-04	2.5670E-01	-5.1619E-02
CCNA_00966	1.4099E-04	8.8489E-05	6.7193E-01	2.1323E-01
CCNA_00967	1.1417E-04	1.3322E-04	-2.2263E-01	-3.5736E-01
CCNA_00968	1.0517E-04	9.7108E-05	1.1496E-01	-1.4203E-01
CCNA_00969	1.3106E-04	1.6243E-04	-3.0956E-01	-4.1280E-01
CCNA_00970	1.3303E-04	1.6142E-04	-2.7908E-01	-3.9336E-01
CCNA_00971	5.8652E-05	4.8106E-05	2.8583E-01	-3.3039E-02
CCNA_00972	4.9788E-05	2.1679E-05	1.1991E+00	5.4948E-01
CCNA_00973	2.3664E-04	1.6270E-04	5.4043E-01	1.2936E-01
CCNA_00974	1.6185E-04	4.1112E-05	1.9768E+00	1.0455E+00
CCNA_00975	4.3726E-05	5.0800E-05	-2.1642E-01	-3.5339E-01
CCNA_00976	4.9326E-04	5.9270E-04	-2.6496E-01	-3.8435E-01
CCNA_00977	7.8460E-04	5.7719E-04	4.4290E-01	6.7146E-02
CCNA_00978	2.5304E-04	4.7964E-04	-9.2257E-01	-8.0381E-01
CCNA_00979	1.9653E-04	2.1908E-04	-1.5680E-01	-3.1536E-01
CCNA_00980	7.1780E-05	1.5503E-04	-1.1108E+00	-9.2389E-01
CCNA_00981	5.5198E-05	6.5227E-05	-2.4092E-01	-3.6902E-01
CCNA_00982	1.0872E-04	1.3641E-04	-3.2736E-01	-4.2416E-01
CCNA_00983	1.1047E-04	4.0457E-05	1.4490E+00	7.0889E-01
CCNA_00984	6.0867E-05	6.2087E-05	-2.8697E-02	-2.3366E-01
CCNA_00985	1.7496E-04	1.0172E-04	7.8232E-01	2.8364E-01
CCNA_00986	1.4126E-04	1.4820E-04	-6.9224E-02	-2.5951E-01
CCNA_00987	9.6589E-05	9.5152E-05	2.1564E-02	-2.0160E-01
CCNA_00988	8.3243E-05	8.5920E-05	-4.5719E-02	-2.4451E-01
CCNA_00989	9.5261E-05	1.1662E-04	-2.9183E-01	-4.0149E-01
CCNA_00990	9.0397E-05	5.1769E-05	8.0402E-01	2.9749E-01
CCNA_00991	7.7597E-05	5.9857E-05	3.7437E-01	2.3434E-02
CCNA_00992	6.4118E-05	1.5747E-04	-1.2963E+00	-1.0422E+00
CCNA_00993	4.4946E-05	1.3695E-04	-1.6073E+00	-1.2406E+00
CCNA_00994	1.0316E-04	7.1567E-05	5.2741E-01	1.2105E-01
CCNA_00995	1.0359E-04	8.3509E-05	3.1080E-01	-1.7108E-02
CCNA_00996	6.0937E-05	1.4933E-05	2.0281E+00	1.0782E+00
CCNA_00997	6.4784E-05	1.4767E-04	-1.1886E+00	-9.7350E-01
CCNA_00998	1.3294E-04	1.1734E-04	1.8011E-01	-1.0047E-01
CCNA_00999	5.9395E-04	1.5871E-04	1.9039E+00	9.9901E-01
CCNA_01000	8.9435E-05	1.5977E-04	-8.3705E-01	-7.4926E-01
CCNA_01001	9.5631E-05	1.0867E-04	-1.8441E-01	-3.3298E-01
CCNA_01002	9.5164E-05	1.1657E-04	-2.9268E-01	-4.0204E-01
CCNA_01003	2.4094E-04	1.1293E-04	1.0932E+00	4.8195E-01
CCNA_01004	2.2007E-04	1.0711E-04	1.0388E+00	4.4723E-01
CCNA_01005	1.5790E-04	1.3815E-04	1.9265E-01	-9.2470E-02
CCNA_01006	1.6221E-04	1.2058E-04	4.2775E-01	5.7486E-02

CCNA_01007	7.1531E-05	1.4045E-04	-9.7341E-01	-8.3623E-01
CCNA_01008	5.6701E-05	7.6630E-05	-4.3458E-01	-4.9255E-01
CCNA_01009	7.3015E-05	1.6176E-05	2.1737E+00	1.1711E+00
CCNA_01010	5.5947E-05	5.2565E-05	8.9863E-02	-1.5803E-01
CCNA_01011	1.8529E-04	1.2061E-04	6.1940E-01	1.7973E-01
CCNA_01012	6.3133E-05	5.2382E-05	2.6919E-01	-4.3650E-02
CCNA_01013	4.9672E-05	8.4810E-05	-7.7179E-01	-7.0763E-01
CCNA_01014	1.6621E-04	1.1052E-04	5.8868E-01	1.6013E-01
CCNA_01015	1.9115E-04	1.4699E-04	3.7895E-01	2.6356E-02
CCNA_01016	5.4689E-05	6.1076E-05	-1.5941E-01	-3.1703E-01
CCNA_01017	6.7540E-05	8.9409E-05	-4.0471E-01	-4.7350E-01
CCNA_01018	5.3242E-05	4.9515E-05	1.0458E-01	-1.4864E-01
CCNA_01019	6.8381E-05	4.1071E-05	7.3531E-01	2.5366E-01
CCNA_01020	1.1404E-04	1.0156E-04	1.6718E-01	-1.0872E-01
CCNA_01021	6.3637E-05	3.1110E-05	1.0322E+00	4.4305E-01
CCNA_01022	7.0717E-05	1.0955E-05	2.6894E+00	1.5001E+00
CCNA_01023	6.4654E-05	8.1362E-05	-3.3165E-01	-4.2689E-01
CCNA_01024	1.0162E-04	1.4081E-04	-4.7054E-01	-5.1548E-01
CCNA_01025	4.2978E-04	1.6243E-04	1.4038E+00	6.8002E-01
CCNA_01026	3.6740E-04	1.0868E-04	1.7571E+00	9.0541E-01
CCNA_01027	6.3448E-05	4.9747E-05	3.5082E-01	8.4131E-03
CCNA_01028	7.5914E-05	1.0110E-04	-4.1340E-01	-4.7904E-01
CCNA_01029	4.7550E-05	6.7664E-05	-5.0898E-01	-5.4000E-01
CCNA_01030	9.9469E-05	1.2016E-04	-2.7269E-01	-3.8928E-01
CCNA_01031	9.3994E-05	1.4588E-04	-6.3411E-01	-6.1982E-01
CCNA_01032	7.2663E-05	8.8307E-05	-2.8134E-01	-3.9480E-01
CCNA_01033	2.5532E-04	1.6270E-04	6.5005E-01	1.9928E-01
CCNA_01034	8.1976E-05	2.4190E-05	1.7604E+00	9.0750E-01
CCNA_01035	6.5931E-05	8.8174E-05	-4.1944E-01	-4.8289E-01
CCNA_01036	6.2611E-05	6.3454E-05	-1.9385E-02	-2.2772E-01
CCNA_01037	1.3683E-04	1.5968E-04	-2.2288E-01	-3.5751E-01
CCNA_01038	7.1725E-05	1.4033E-04	-9.6830E-01	-8.3298E-01
CCNA_01039	7.5355E-05	1.5550E-04	-1.0451E+00	-8.8197E-01
CCNA_01040	5.5711E-05	9.7530E-05	-8.0788E-01	-7.3065E-01
CCNA_01041	3.6985E-04	7.7650E-05	2.2517E+00	1.2209E+00
CCNA_01042	2.9377E-04	1.6261E-04	8.5326E-01	3.2889E-01
CCNA_01043	3.6701E-04	1.9975E-04	8.7758E-01	3.4441E-01
CCNA_01044	3.8180E-04	2.2979E-04	7.3247E-01	2.5185E-01
CCNA_01045	9.8656E-05	7.8669E-05	3.2652E-01	-7.0821E-03
CCNA_01046	2.4675E-04	5.0715E-04	-1.0394E+00	-8.7831E-01
CCNA_01047	1.9494E-04	1.9421E-04	5.4004E-03	-2.1191E-01
CCNA_01048	7.1452E-05	8.1876E-06	3.1241E+00	1.7773E+00
CCNA_01049	1.2770E-04	8.7710E-05	5.4191E-01	1.3030E-01
CCNA_01050	7.1822E-05	8.8124E-05	-2.9516E-01	-4.0362E-01
CCNA_01051	7.4226E-05	6.1705E-05	2.6643E-01	-4.5410E-02
CCNA_01052	6.6731E-05	9.3511E-05	-4.8681E-01	-5.2586E-01
CCNA_01053	6.8664E-05	4.3192E-05	6.6860E-01	2.1111E-01
CCNA_01054	7.2839E-05	1.4260E-04	-9.6913E-01	-8.3350E-01
CCNA_01055	5.7714E-05	2.3660E-05	1.2861E+00	6.0498E-01
CCNA_01056	1.4757E-04	6.2832E-05	1.2317E+00	5.7029E-01
CCNA_01057	5.6064E-04	1.6243E-04	1.7872E+00	9.2461E-01
CCNA_01058	4.5354E-04	1.6243E-04	1.4814E+00	7.2954E-01
CCNA_01059	4.1124E-04	1.4285E-04	1.5254E+00	7.5761E-01

CCNA_01060	2.3673E-04	2.4211E-04	-3.2435E-02	-2.3604E-01
CCNA_01061	6.8862E-05	1.2230E-04	-8.2864E-01	-7.4389E-01
CCNA_01062	7.5174E-05	1.6298E-04	-1.1164E+00	-9.2743E-01
CCNA_01063	6.5676E-05	3.9562E-05	7.3104E-01	2.5094E-01
CCNA_01064	6.9237E-05	5.3319E-05	3.7677E-01	2.4967E-02
CCNA_01065	6.1025E-05	1.6156E-04	-1.4045E+00	-1.1112E+00
CCNA_01066	7.2677E-05	1.7129E-05	2.0844E+00	1.1142E+00
CCNA_01067	2.2279E-04	1.3510E-04	7.2153E-01	2.4487E-01
CCNA_01068	1.3913E-04	1.6243E-04	-2.2337E-01	-3.5783E-01
CCNA_01069	1.0892E-04	8.1404E-05	4.1997E-01	5.2524E-02
CCNA_01070	1.5230E-04	3.1897E-05	2.2551E+00	1.2231E+00
CCNA_01071	7.4680E-05	4.5579E-07	7.3303E+00	4.4602E+00
CCNA_01072	8.7396E-05	1.2329E-04	-4.9649E-01	-5.3204E-01
CCNA_01073	9.4841E-05	7.0200E-05	4.3394E-01	6.1436E-02
CCNA_01074	8.4857E-05	1.6269E-04	-9.3902E-01	-8.1430E-01
CCNA_01075	5.6618E-05	1.0277E-04	-8.6006E-01	-7.6393E-01
CCNA_01076	6.3591E-05	5.3203E-05	2.5720E-01	-5.1301E-02
CCNA_01077	5.7667E-05	1.5721E-05	1.8745E+00	9.8026E-01
CCNA_01078	6.6971E-05	5.1247E-05	3.8593E-01	3.0814E-02
CCNA_01079	1.1056E-04	1.6267E-04	-5.5707E-01	-5.7067E-01
CCNA_01080	1.1079E-04	1.2543E-04	-1.7907E-01	-3.2957E-01
CCNA_01081	9.5363E-05	4.0325E-05	1.2415E+00	5.7655E-01
CCNA_01082	2.6154E-04	1.4925E-04	8.0928E-01	3.0084E-01
CCNA_01083	2.5524E-04	1.4221E-04	8.4374E-01	3.2282E-01
CCNA_01084	8.6235E-05	5.4918E-05	6.5085E-01	1.9979E-01
CCNA_01085	3.4713E-04	1.0945E-04	1.6651E+00	8.4675E-01
CCNA_01086	1.3053E-04	1.7526E-04	-4.2521E-01	-4.8657E-01
CCNA_01087	1.2885E-04	1.7453E-04	-4.3775E-01	-4.9457E-01
CCNA_01088	4.7818E-05	2.3129E-05	1.0475E+00	4.5277E-01
CCNA_01089	6.1778E-05	4.8264E-05	3.5602E-01	1.1732E-02
CCNA_01090	1.0074E-04	4.4443E-05	1.1804E+00	5.3756E-01
CCNA_01091	5.2271E-05	2.9278E-05	8.3591E-01	3.1783E-01
CCNA_01092	5.9961E-05	1.5799E-04	-1.3977E+00	-1.1069E+00
CCNA_01093	2.7153E-04	1.1961E-04	1.1828E+00	5.3906E-01
CCNA_01094	9.3084E-05	6.3802E-05	5.4481E-01	1.3215E-01
CCNA_01095	4.6371E-05	1.2107E-04	-1.3846E+00	-1.0985E+00
CCNA_01096	4.0517E-05	5.2805E-05	-3.8223E-01	-4.5915E-01
CCNA_01097	1.2472E-04	1.9501E-04	-6.4485E-01	-6.2666E-01
CCNA_01098	1.2969E-04	3.2485E-04	-1.3247E+00	-1.0603E+00
CCNA_01099	8.7794E-05	1.6169E-04	-8.8103E-01	-7.7731E-01
CCNA_01100	1.2390E-04	9.1274E-05	4.4081E-01	6.5819E-02
CCNA_01101	3.8605E-04	1.1139E-04	1.7930E+00	9.2831E-01
CCNA_01102	3.8560E-04	1.0226E-04	1.9147E+00	1.0059E+00
CCNA_01103	1.0639E-04	1.1325E-04	-9.0241E-02	-2.7291E-01
CCNA_01104	4.1711E-04	1.6072E-04	1.3758E+00	6.6221E-01
CCNA_01105	4.0388E-04	1.2987E-04	1.6368E+00	8.2868E-01
CCNA_01106	1.4404E-04	1.4160E-04	2.4569E-02	-1.9968E-01
CCNA_01107	2.6707E-04	4.4883E-05	2.5727E+00	1.4256E+00
CCNA_01108	5.6844E-04	3.2841E-04	7.9149E-01	2.8950E-01
CCNA_01109	7.2683E-04	1.3455E-04	2.4334E+00	1.3368E+00
CCNA_01110	7.1591E-05	4.3225E-05	7.2771E-01	2.4881E-01
CCNA_01111	1.8168E-04	1.1697E-04	6.3515E-01	1.8977E-01
CCNA_01112	6.0363E-05	5.3526E-05	1.7332E-01	-1.0480E-01

CCNA_01113	5.7085E-05	8.0948E-05	-5.0392E-01	-5.3677E-01
CCNA_01114	6.4432E-05	7.4567E-05	-2.1080E-01	-3.4981E-01
CCNA_01115	2.0182E-04	9.1456E-05	1.1419E+00	5.1297E-01
CCNA_01116	2.0037E-04	7.3291E-05	1.4508E+00	7.1004E-01
CCNA_01117	7.4485E-05	1.1039E-04	-5.6762E-01	-5.7741E-01
CCNA_01118	7.2742E-05	1.3788E-04	-9.2255E-01	-8.0379E-01
CCNA_01119	1.1648E-04	1.0222E-04	1.8830E-01	-9.5248E-02
CCNA_01120	1.9774E-04	1.2856E-04	6.2115E-01	1.8084E-01
CCNA_01121	2.7356E-04	1.2905E-04	1.0838E+00	4.7594E-01
CCNA_01122	1.2307E-04	1.0244E-04	2.6453E-01	-4.6623E-02
CCNA_01123	6.2948E-05	6.1838E-05	2.5584E-02	-1.9903E-01
CCNA_01124	4.5261E-05	1.6988E-06	4.7288E+00	2.8009E+00
CCNA_01125	9.8041E-05	4.0573E-05	1.2726E+00	5.9638E-01
CCNA_01126	8.4543E-05	3.6422E-05	1.2146E+00	5.5940E-01
CCNA_01127	7.2751E-05	1.0136E-04	-4.7845E-01	-5.2053E-01
CCNA_01128	4.9682E-05	1.3222E-04	-1.4121E+00	-1.1161E+00
CCNA_01129	1.6165E-04	2.5814E-05	2.6463E+00	1.4725E+00
CCNA_01130	1.3524E-04	1.4681E-04	-1.1844E-01	-2.9090E-01
CCNA_01131	9.5876E-05	1.6133E-04	-7.5079E-01	-6.9424E-01
CCNA_01132	2.4438E-04	2.4182E-05	3.3367E+00	1.9129E+00
CCNA_01133	7.5387E-05	1.0317E-04	-4.5260E-01	-5.0404E-01
CCNA_01134	5.9674E-05	1.4335E-04	-1.2643E+00	-1.0218E+00
CCNA_01135	6.8839E-05	4.6731E-05	5.5870E-01	1.4101E-01
CCNA_01136	5.2687E-05	1.1123E-04	-1.0780E+00	-9.0294E-01
CCNA_01137	7.0647E-05	6.1324E-06	3.5243E+00	2.0326E+00
CCNA_01138	6.2624E-05	3.7035E-05	7.5763E-01	2.6790E-01
CCNA_01139	6.7087E-05	1.5901E-04	-1.2450E+00	-1.0095E+00
CCNA_01140	2.6143E-04	7.8851E-05	1.7291E+00	8.8755E-01
CCNA_01141	2.6373E-04	5.9990E-05	2.1361E+00	1.1471E+00
CCNA_01142	1.7124E-04	5.3675E-05	1.6735E+00	8.5208E-01
CCNA_01143	9.2307E-05	7.1948E-05	3.5938E-01	1.3878E-02
CCNA_01144	1.8027E-04	1.0143E-04	8.2964E-01	3.1383E-01
CCNA_01145	6.1353E-05	1.6045E-04	-1.3868E+00	-1.0999E+00
CCNA_01146	6.1071E-05	5.8067E-05	7.2658E-02	-1.6901E-01
CCNA_01147	6.9931E-05	1.4150E-04	-1.0168E+00	-8.6392E-01
CCNA_01148	6.4257E-05	1.1410E-04	-8.2843E-01	-7.4376E-01
CCNA_01149	7.1808E-05	1.9060E-07	8.4961E+00	5.2038E+00
CCNA_01150	7.3769E-05	1.2049E-05	2.6131E+00	1.4514E+00
CCNA_01151	1.3547E-04	1.2309E-04	1.3827E-01	-1.2716E-01
CCNA_01152	6.9357E-05	4.2165E-05	7.1783E-01	2.4251E-01
CCNA_01153	4.9816E-05	1.5663E-05	1.6687E+00	8.4899E-01
CCNA_01154	1.1795E-04	1.6243E-04	-4.6160E-01	-5.0978E-01
CCNA_01155	1.5611E-04	7.2197E-05	1.1125E+00	4.9423E-01
CCNA_01156	8.9856E-05	4.4394E-05	1.0171E+00	4.3337E-01
CCNA_01157	9.1923E-05	1.6195E-04	-8.1709E-01	-7.3652E-01
CCNA_01158	8.1593E-05	1.0925E-04	-4.2113E-01	-4.8396E-01
CCNA_01159	3.6561E-04	1.3016E-04	1.4900E+00	7.3503E-01
CCNA_01160	6.8728E-05	9.6080E-05	-4.8336E-01	-5.2366E-01
CCNA_01161	6.1713E-05	2.8259E-06	4.4447E+00	2.6197E+00
CCNA_01162	1.1831E-04	5.8540E-05	1.0149E+00	4.3200E-01
CCNA_01163	8.2904E-04	1.6267E-04	2.3494E+00	1.2832E+00
CCNA_01164	1.5384E-04	1.1613E-04	4.0561E-01	4.3366E-02
CCNA_01165	7.3713E-05	4.4253E-05	7.3597E-01	2.5408E-01

CCNA_01166	5.3982E-05	1.0853E-04	-1.0075E+00	-8.5797E-01
CCNA_01167	6.1172E-05	1.1007E-04	-8.4745E-01	-7.5589E-01
CCNA_01168	6.8687E-05	1.0013E-04	-5.4383E-01	-5.6223E-01
CCNA_01169	7.6820E-05	2.4861E-05	1.6272E+00	8.2255E-01
CCNA_01170	2.1375E-04	1.5910E-04	4.2592E-01	5.6317E-02
CCNA_01171	3.5564E-04	3.2518E-04	1.2919E-01	-1.3295E-01
CCNA_01172	8.5801E-05	1.5537E-04	-8.5661E-01	-7.6173E-01
CCNA_01173	1.2601E-04	1.1440E-04	1.3939E-01	-1.2644E-01
CCNA_01174	1.8816E-04	1.0602E-04	8.2763E-01	3.1255E-01
CCNA_01175	1.9170E-04	1.0335E-04	8.9124E-01	3.5312E-01
CCNA_01176	3.9051E-05	1.0895E-04	-1.4802E+00	-1.1595E+00
CCNA_01177	6.0174E-05	6.8211E-05	-1.8093E-01	-3.3076E-01
CCNA_01178	5.5397E-05	5.7073E-05	-4.3088E-02	-2.4284E-01
CCNA_01179	9.8119E-05	6.4125E-05	6.1353E-01	1.7598E-01
CCNA_01180	1.0276E-04	1.6243E-04	-6.6055E-01	-6.3668E-01
CCNA_01181	4.7344E-04	1.6243E-04	1.5433E+00	7.6905E-01
CCNA_01182	4.7478E-04	1.6243E-04	1.5474E+00	7.7165E-01
CCNA_01183	1.3577E-04	1.1591E-04	2.2813E-01	-6.9841E-02
CCNA_01184	6.9376E-05	1.1791E-04	-7.6517E-01	-7.0341E-01
CCNA_01185	1.2473E-04	2.0187E-05	2.6268E+00	1.4601E+00
CCNA_01186	1.3051E-04	8.5854E-06	3.9248E+00	2.2881E+00
CCNA_01187	9.2515E-05	6.8617E-05	4.3102E-01	5.9571E-02
CCNA_01188	6.0160E-05	1.6238E-04	-1.4325E+00	-1.1291E+00
CCNA_01189	1.1243E-04	4.4402E-05	1.3401E+00	6.3944E-01
CCNA_01190	1.2930E-04	1.2116E-05	3.4149E+00	1.9628E+00
CCNA_01191	1.7708E-04	4.1344E-05	2.0984E+00	1.1231E+00
CCNA_01192	5.3589E-05	5.9667E-05	-1.5507E-01	-3.1426E-01
CCNA_01193	1.0831E-04	9.2550E-05	2.2681E-01	-7.0684E-02
CCNA_01194	1.1938E-04	1.4087E-04	-2.3884E-01	-3.6769E-01
CCNA_01195	3.3734E-04	3.2999E-05	3.3534E+00	1.9236E+00
CCNA_01196	3.6161E-04	4.0764E-05	3.1488E+00	1.7931E+00
CCNA_01197	1.2177E-04	3.7176E-05	1.7115E+00	8.7630E-01
CCNA_01198	1.2185E-04	3.7441E-05	1.7022E+00	8.7036E-01
CCNA_01199	9.8068E-05	1.6267E-04	-7.3006E-01	-6.8102E-01
CCNA_01200	5.3866E-05	2.0022E-05	1.4274E+00	6.9508E-01
CCNA_01201	5.5772E-05	1.0823E-04	-9.5648E-01	-8.2543E-01
CCNA_01202	1.0269E-04	6.0636E-05	7.5988E-01	2.6933E-01
CCNA_01203	6.3864E-05	9.6138E-05	-5.9013E-01	-5.9176E-01
CCNA_01204	1.3385E-04	8.9542E-05	5.7990E-01	1.5453E-01
CCNA_01205	1.4911E-04	6.9222E-05	1.1069E+00	4.9071E-01
CCNA_01206	6.3831E-05	8.4288E-05	-4.0109E-01	-4.7118E-01
CCNA_01207	8.1334E-05	7.2909E-05	1.5766E-01	-1.1479E-01
CCNA_01208	5.8084E-05	8.4196E-05	-5.3566E-01	-5.5702E-01
CCNA_01209	5.3543E-05	1.4986E-04	-1.4848E+00	-1.1624E+00
CCNA_01210	1.3935E-04	6.0031E-05	1.2148E+00	5.5950E-01
CCNA_01211	9.3042E-05	1.0866E-04	-2.2390E-01	-3.5817E-01
CCNA_01212	8.4529E-05	6.3222E-05	4.1891E-01	5.1847E-02
CCNA_01213	5.7408E-05	1.3797E-04	-1.2650E+00	-1.0222E+00
CCNA_01214	1.1306E-04	1.3989E-05	3.0140E+00	1.7071E+00
CCNA_01215	1.2530E-04	1.3437E-04	-1.0086E-01	-2.7968E-01
CCNA_01216	1.9252E-04	4.9938E-05	1.9466E+00	1.0263E+00
CCNA_01217	1.6889E-04	3.4449E-05	2.2932E+00	1.2474E+00
CCNA_01218	6.2088E-05	1.4143E-04	-1.1876E+00	-9.7288E-01

CCNA_01219	5.9489E-05	7.0357E-05	-2.4212E-01	-3.6979E-01
CCNA_01220	3.3162E-04	3.1375E-05	3.4015E+00	1.9543E+00
CCNA_01221	5.0599E-04	1.0310E-04	2.2950E+00	1.2485E+00
CCNA_01222	5.6951E-05	1.0399E-04	-8.6860E-01	-7.6938E-01
CCNA_01223	1.1899E-04	6.8575E-05	7.9499E-01	2.9173E-01
CCNA_01224	1.3923E-04	1.5932E-04	-1.9445E-01	-3.3938E-01
CCNA_01225	6.6421E-05	2.5731E-05	1.3677E+00	6.5706E-01
CCNA_01226	1.9787E-04	8.4395E-05	1.2292E+00	5.6867E-01
CCNA_01227	1.9899E-04	1.0113E-04	9.7641E-01	4.0744E-01
CCNA_01228	6.7040E-05	7.9017E-05	-2.3718E-01	-3.6664E-01
CCNA_01229	1.7149E-04	1.7403E-05	3.3000E+00	1.8896E+00
CCNA_01230	1.5959E-04	5.4612E-05	1.5469E+00	7.7135E-01
CCNA_01231	5.2729E-05	2.5035E-05	1.0743E+00	4.6987E-01
CCNA_01232	6.6624E-05	4.7609E-05	4.8466E-01	9.3785E-02
CCNA_01233	1.0481E-04	9.6967E-05	1.1222E-01	-1.4377E-01
CCNA_01234	1.1144E-04	5.8117E-05	9.3904E-01	3.8361E-01
CCNA_01235	1.2164E-04	9.3362E-05	3.8166E-01	2.8090E-02
CCNA_01236	4.5802E-05	6.9462E-05	-6.0084E-01	-5.9859E-01
CCNA_01237	4.6472E-05	1.5704E-04	-1.7566E+00	-1.3358E+00
CCNA_01238	5.2091E-05	1.2553E-04	-1.2689E+00	-1.0247E+00
CCNA_01239	5.5762E-05	1.3020E-04	-1.2233E+00	-9.9563E-01
CCNA_01240	6.6925E-05	9.3387E-05	-4.8070E-01	-5.2196E-01
CCNA_01241	6.1908E-05	1.3920E-04	-1.1689E+00	-9.6094E-01
CCNA_01242	2.0933E-04	1.8577E-04	1.7222E-01	-1.0550E-01
CCNA_01243	2.0141E-04	1.7398E-04	2.1118E-01	-8.0653E-02
CCNA_01244	9.5100E-05	2.7198E-05	1.8056E+00	9.3631E-01
CCNA_01245	5.2396E-05	1.4060E-04	-1.4240E+00	-1.1236E+00
CCNA_01246	1.1678E-04	8.4395E-05	4.6850E-01	8.3478E-02
CCNA_01247	1.8238E-04	8.9144E-05	1.0327E+00	4.4333E-01
CCNA_01248	1.6971E-04	1.0383E-04	7.0883E-01	2.3677E-01
CCNA_01249	9.1812E-05	1.5409E-04	-7.4702E-01	-6.9183E-01
CCNA_01250	5.4851E-05	1.1914E-04	-1.1191E+00	-9.2914E-01
CCNA_01251	4.3337E-05	4.5919E-05	-8.3576E-02	-2.6866E-01
CCNA_01252	1.9536E-04	1.5995E-04	2.8852E-01	-3.1321E-02
CCNA_01253	1.9745E-04	1.6108E-04	2.9364E-01	-2.8058E-02
CCNA_01254	8.3724E-05	1.2848E-04	-6.1787E-01	-6.0945E-01
CCNA_01255	1.3765E-04	9.3602E-05	5.5627E-01	1.3946E-01
CCNA_01256	6.5552E-05	1.1702E-04	-8.3607E-01	-7.4863E-01
CCNA_01257	5.6428E-05	7.9688E-05	-4.9798E-01	-5.3299E-01
CCNA_01258	2.1991E-04	1.2643E-04	7.9857E-01	2.9401E-01
CCNA_01259	2.0694E-04	1.4245E-04	5.3877E-01	1.2830E-01
CCNA_01260	9.0873E-05	4.0316E-05	1.1723E+00	5.3237E-01
CCNA_01261	3.4961E-04	1.2116E-04	1.5287E+00	7.5970E-01
CCNA_01262	6.9010E-05	1.1570E-04	-7.4556E-01	-6.9090E-01
CCNA_01263	7.9382E-05	7.0805E-05	1.6489E-01	-1.1018E-01
CCNA_01264	3.4181E-04	2.8765E-04	2.4887E-01	-5.6612E-02
CCNA_01265	2.5135E-04	1.8392E-04	4.5055E-01	7.2029E-02
CCNA_01266	7.4065E-05	3.8626E-05	9.3899E-01	3.8358E-01
CCNA_01267	6.2245E-05	2.2781E-05	1.4497E+00	7.0933E-01
CCNA_01268	2.1297E-04	5.8995E-05	1.8518E+00	9.6580E-01
CCNA_01269	2.0737E-04	4.8388E-05	2.0993E+00	1.1236E+00
CCNA_01270	4.2338E-05	1.3326E-04	-1.6541E+00	-1.2704E+00
CCNA_01271	7.1567E-05	5.8382E-05	2.9366E-01	-2.8041E-02

CCNA_01272	5.7580E-05	1.4098E-04	-1.2918E+00	-1.0393E+00
CCNA_01273	8.7706E-05	1.6234E-04	-8.8830E-01	-7.8195E-01
CCNA_01274	1.0629E-04	1.5735E-04	-5.6589E-01	-5.7630E-01
CCNA_01275	1.0157E-04	1.4615E-04	-5.2501E-01	-5.5023E-01
CCNA_01276	6.3716E-05	1.4308E-04	-1.1670E+00	-9.5974E-01
CCNA_01277	8.9523E-05	4.3756E-06	4.3521E+00	2.5606E+00
CCNA_01278	1.4947E-04	9.9958E-05	5.8034E-01	1.5481E-01
CCNA_01279	1.0590E-04	3.0687E-05	1.7866E+00	9.2424E-01
CCNA_01280	1.3104E-04	1.4811E-04	-1.7675E-01	-3.2809E-01
CCNA_01281	1.3198E-04	1.3349E-04	-1.6460E-02	-2.2585E-01
CCNA_01282	4.2958E-05	9.6818E-05	-1.1723E+00	-9.6310E-01
CCNA_01283	4.1751E-05	1.5060E-04	-1.8508E+00	-1.3958E+00
CCNA_01284	6.0844E-05	5.8109E-05	6.6267E-02	-1.7308E-01
CCNA_01285	2.2642E-04	1.0096E-04	1.1651E+00	5.2781E-01
CCNA_01286	1.9176E-04	1.6243E-04	2.3950E-01	-6.2591E-02
CCNA_01287	1.1545E-04	9.9362E-05	2.1645E-01	-7.7292E-02
CCNA_01288	1.1258E-04	1.2986E-04	-2.0597E-01	-3.4673E-01
CCNA_01289	7.4582E-05	5.2449E-05	5.0779E-01	1.0854E-01
CCNA_01290	7.2899E-05	8.1304E-05	-1.5748E-01	-3.1580E-01
CCNA_01291	8.1047E-05	1.5286E-04	-9.1540E-01	-7.9923E-01
CCNA_01292	5.8449E-05	7.6158E-05	-3.8186E-01	-4.5892E-01
CCNA_01293	0.0000E+00	0.0000E+00	-8.4168E-01	-7.5221E-01
CCNA_01294	0.0000E+00	0.0000E+00	-8.4168E-01	-7.5221E-01
CCNA_01295	6.1820E-05	1.0104E-04	-7.0873E-01	-6.6741E-01
CCNA_01296	1.4408E-04	1.0064E-04	5.1765E-01	1.1483E-01
CCNA_01297	5.2232E-04	6.7056E-04	-3.6044E-01	-4.4525E-01
CCNA_01298	2.8698E-04	1.9422E-04	5.6318E-01	1.4387E-01
CCNA_01299	1.1739E-04	7.6771E-05	6.1255E-01	1.7536E-01
CCNA_01300	7.0814E-05	6.1142E-05	2.1177E-01	-8.0277E-02
CCNA_01301	3.8302E-05	7.8114E-05	-1.0282E+00	-8.7115E-01
CCNA_01302	5.5776E-05	1.1898E-04	-1.0929E+00	-9.1248E-01
CCNA_01303	9.0753E-05	1.1884E-04	-3.8909E-01	-4.6353E-01
CCNA_01304	2.6809E-04	3.6950E-04	-4.6289E-01	-5.1060E-01
CCNA_01305	3.7677E-04	4.8889E-04	-3.7581E-01	-4.5506E-01
CCNA_01306	1.4837E-04	1.3469E-04	1.3951E-01	-1.2636E-01
CCNA_01307	7.0527E-05	1.3819E-04	-9.7045E-01	-8.3434E-01
CCNA_01308	7.4055E-05	9.4506E-05	-3.5183E-01	-4.3976E-01
CCNA_01309	6.4419E-05	1.1801E-04	-8.7333E-01	-7.7240E-01
CCNA_01310	6.3915E-05	1.0518E-04	-7.1864E-01	-6.7373E-01
CCNA_01311	7.8268E-05	1.8016E-05	2.1186E+00	1.1360E+00
CCNA_01312	8.4251E-05	1.3636E-04	-6.9469E-01	-6.5845E-01
CCNA_01313	1.1693E-04	1.4960E-04	-3.5552E-01	-4.4212E-01
CCNA_01314	6.6994E-05	2.7016E-06	4.6278E+00	2.7365E+00
CCNA_01315	8.4330E-05	5.8904E-05	5.1755E-01	1.1476E-01
CCNA_01316	1.2083E-04	1.1822E-04	3.1401E-02	-1.9532E-01
CCNA_01317	1.4240E-04	4.1974E-05	1.7621E+00	9.0862E-01
CCNA_01318	8.3586E-05	8.9492E-05	-9.8556E-02	-2.7821E-01
CCNA_01319	7.0092E-05	1.9176E-05	1.8694E+00	9.7704E-01
CCNA_01320	7.1956E-05	6.8136E-05	7.8611E-02	-1.6521E-01
CCNA_01321	7.6992E-05	5.1148E-05	5.8989E-01	1.6090E-01
CCNA_01322	8.0723E-05	1.3966E-04	-7.9088E-01	-7.1981E-01
CCNA_01323	6.9255E-05	3.3397E-05	1.0520E+00	4.5563E-01
CCNA_01324	8.6513E-05	3.9024E-05	1.1483E+00	5.1710E-01

CCNA_01325	1.4057E-04	1.6243E-04	-2.0854E-01	-3.4836E-01
CCNA_01326	2.4054E-04	1.3876E-04	7.9366E-01	2.9088E-01
CCNA_01327	1.0240E-04	1.4486E-04	-5.0049E-01	-5.3458E-01
CCNA_01328	3.6959E-04	3.9245E-04	-8.6587E-02	-2.7058E-01
CCNA_01329	1.2457E-04	1.2357E-04	1.1650E-02	-2.0792E-01
CCNA_01330	1.3958E-04	8.9417E-06	3.9631E+00	2.3125E+00
CCNA_01331	9.6468E-05	1.1250E-04	-2.2190E-01	-3.5689E-01
CCNA_01332	7.4744E-05	1.1921E-04	-6.7347E-01	-6.4492E-01
CCNA_01333	4.5973E-05	1.7991E-05	1.3530E+00	6.4763E-01
CCNA_01334	4.0498E-05	9.1762E-05	-1.1800E+00	-9.6802E-01
CCNA_01335	2.0389E-04	1.4327E-04	5.0902E-01	1.0932E-01
CCNA_01336	2.5505E-04	1.4316E-04	8.3310E-01	3.1603E-01
CCNA_01337	6.9445E-05	2.5599E-05	1.4394E+00	7.0278E-01
CCNA_01338	6.0548E-05	1.4337E-05	2.0777E+00	1.1099E+00
CCNA_01339	4.5108E-05	1.2556E-04	-1.4768E+00	-1.1573E+00
CCNA_01340	4.2907E-05	4.2156E-05	2.5346E-02	-1.9918E-01
CCNA_01341	9.2959E-05	1.5554E-04	-7.4263E-01	-6.8903E-01
CCNA_01342	8.2443E-05	1.4351E-04	-7.9965E-01	-7.2540E-01
CCNA_01343	6.1774E-05	1.1038E-05	2.4835E+00	1.3687E+00
CCNA_01344	6.5158E-05	3.6488E-05	8.3631E-01	3.1808E-01
CCNA_01345	4.3323E-05	9.6047E-06	2.1722E+00	1.1702E+00
CCNA_01346	4.6357E-05	3.1913E-05	5.3839E-01	1.2805E-01
CCNA_01347	6.1607E-05	3.6007E-05	7.7459E-01	2.7871E-01
CCNA_01348	5.6146E-05	5.2830E-05	8.7722E-02	-1.5940E-01
CCNA_01349	4.2311E-05	3.0662E-07	7.0701E+00	4.2943E+00
CCNA_01350	0.0000E+00	0.0000E+00	-8.4168E-01	-7.5221E-01
CCNA_01351	7.0587E-05	4.3689E-05	6.9195E-01	2.2600E-01
CCNA_01352	1.2941E-04	1.4505E-04	-1.6466E-01	-3.2038E-01
CCNA_01353	5.0662E-05	7.1923E-05	-5.0559E-01	-5.3784E-01
CCNA_01354	2.6434E-04	7.4998E-05	1.8174E+00	9.4383E-01
CCNA_01355	2.4789E-04	6.3081E-05	1.9743E+00	1.0439E+00
CCNA_01356	1.0261E-04	1.1496E-04	-1.6398E-01	-3.1995E-01
CCNA_01357	5.7325E-05	1.2886E-05	2.1525E+00	1.1576E+00
CCNA_01358	5.6123E-05	6.0636E-05	-1.1167E-01	-2.8658E-01
CCNA_01359	5.4389E-05	8.8158E-05	-6.9679E-01	-6.5979E-01
CCNA_01360	5.9101E-05	9.0403E-05	-6.1322E-01	-6.0649E-01
CCNA_01361	8.3516E-05	1.2658E-04	-5.9999E-01	-5.9805E-01
CCNA_01362	6.5515E-05	3.4051E-05	9.4385E-01	3.8667E-01
CCNA_01363	4.9117E-05	4.1966E-05	2.2688E-01	-7.0640E-02
CCNA_01364	5.8125E-05	1.0399E-04	-8.3915E-01	-7.5060E-01
CCNA_01365	8.2379E-05	4.0275E-06	4.3514E+00	2.5602E+00
CCNA_01366	1.0844E-04	1.1370E-04	-6.8354E-02	-2.5895E-01
CCNA_01367	1.1279E-04	1.5442E-04	-4.5323E-01	-5.0444E-01
CCNA_01368	1.2216E-04	1.2866E-04	-7.4828E-02	-2.6308E-01
CCNA_01369	6.3896E-05	4.4419E-05	5.2440E-01	1.1913E-01
CCNA_01370	1.1087E-04	1.2350E-04	-1.5567E-01	-3.1464E-01
CCNA_01371	2.0857E-04	1.4779E-04	4.9690E-01	1.0159E-01
CCNA_01372	9.9192E-05	1.2298E-04	-3.1016E-01	-4.1318E-01
CCNA_01373	1.0415E-04	1.5658E-04	-5.8823E-01	-5.9055E-01
CCNA_01374	6.2782E-05	1.0722E-04	-7.7213E-01	-7.0785E-01
CCNA_01375	7.9706E-05	8.5000E-05	-9.2837E-02	-2.7457E-01
CCNA_01376	5.7020E-05	1.3418E-04	-1.2346E+00	-1.0029E+00
CCNA_01377	3.9565E-04	1.6262E-04	1.2827E+00	6.0281E-01

CCNA_01378	3.8541E-04	1.6276E-04	1.2436E+00	5.7788E-01
CCNA_01379	1.4434E-04	1.6242E-04	-1.7025E-01	-3.2394E-01
CCNA_01380	2.2271E-04	3.8410E-05	2.5353E+00	1.4018E+00
CCNA_01381	6.8215E-05	3.9438E-05	7.9030E-01	2.8873E-01
CCNA_01382	9.2866E-05	5.6824E-05	7.0851E-01	2.3656E-01
CCNA_01383	7.3061E-05	1.5924E-04	-1.1240E+00	-9.3227E-01
CCNA_01384	1.2735E-04	1.6243E-04	-3.5103E-01	-4.3925E-01
CCNA_01385	1.6559E-04	1.2724E-04	3.8001E-01	2.7038E-02
CCNA_01386	8.5125E-05	8.8017E-05	-4.8245E-02	-2.4612E-01
CCNA_01387	8.7604E-05	1.5914E-04	-8.6127E-01	-7.6470E-01
CCNA_01388	1.0546E-04	6.4109E-05	7.1801E-01	2.4263E-01
CCNA_01389	1.3965E-04	1.5389E-04	-1.4009E-01	-3.0470E-01
CCNA_01390	6.0451E-05	5.2681E-05	1.9838E-01	-8.8819E-02
CCNA_01391	6.8187E-05	4.8239E-05	4.9916E-01	1.0303E-01
CCNA_01392	8.3720E-05	1.0738E-04	-3.5917E-01	-4.4444E-01
CCNA_01393	1.7245E-04	1.6243E-04	8.6375E-02	-1.6026E-01
CCNA_01394	2.2091E-04	1.1515E-04	9.3988E-01	3.8414E-01
CCNA_01395	2.0846E-04	1.2798E-04	7.0386E-01	2.3360E-01
CCNA_01396	9.5470E-05	3.5270E-05	1.4363E+00	7.0081E-01
CCNA_01397	1.0748E-04	7.1666E-05	5.8457E-01	1.5751E-01
CCNA_01398	5.1675E-05	5.1537E-05	3.7378E-03	-2.1297E-01
CCNA_01399	4.5696E-05	6.6073E-05	-5.3203E-01	-5.5471E-01
CCNA_01400	5.2331E-05	1.1309E-04	-1.1116E+00	-9.2440E-01
CCNA_01401	6.5926E-05	1.6276E-04	-1.3038E+00	-1.0470E+00
CCNA_01402	6.8594E-05	1.5667E-04	-1.1916E+00	-9.7540E-01
CCNA_01403	6.3646E-05	1.6276E-04	-1.3545E+00	-1.0793E+00
CCNA_01404	6.1912E-05	7.0332E-05	-1.8402E-01	-3.3273E-01
CCNA_01405	1.5364E-04	2.3635E-05	2.7001E+00	1.5069E+00
CCNA_01406	8.5213E-05	8.1014E-05	7.2834E-02	-1.6889E-01
CCNA_01407	9.7911E-05	1.6275E-04	-7.3311E-01	-6.8296E-01
CCNA_01408	6.3637E-05	1.0536E-04	-7.2742E-01	-6.7933E-01
CCNA_01409	1.3461E-04	1.1875E-04	1.8075E-01	-1.0006E-01
CCNA_01410	3.5035E-04	4.3366E-05	3.0139E+00	1.7070E+00
CCNA_04014	1.9774E-04	1.4511E-04	4.4646E-01	6.9417E-02
CCNA_01412	1.9144E-04	1.6014E-04	2.5755E-01	-5.1074E-02
CCNA_01413	7.3283E-05	8.2183E-05	-1.6541E-01	-3.2086E-01
CCNA_01414	6.5672E-05	2.3378E-05	1.4897E+00	7.3486E-01
CCNA_01415	1.8332E-04	1.6130E-04	1.8458E-01	-9.7621E-02
CCNA_01416	4.1145E-05	8.7760E-06	2.2279E+00	1.2057E+00
CCNA_01417	4.6039E-04	1.6243E-04	1.5030E+00	7.4333E-01
CCNA_01418	1.8786E-04	1.5144E-04	3.1089E-01	-1.7054E-02
CCNA_01419	2.0696E-04	1.6243E-04	3.4954E-01	7.5989E-03
CCNA_01420	1.2602E-04	1.6238E-04	-3.6577E-01	-4.4866E-01
CCNA_01421	1.2972E-04	1.6243E-04	-3.2440E-01	-4.2227E-01
CCNA_01422	1.6986E-04	9.8922E-05	7.7991E-01	2.8211E-01
CCNA_01423	1.4196E-04	5.9228E-05	1.2610E+00	5.8896E-01
CCNA_01424	9.5363E-05	1.4537E-04	-6.0825E-01	-6.0332E-01
CCNA_01425	4.3318E-04	1.5412E-04	1.4908E+00	7.3557E-01
CCNA_01426	7.8120E-05	1.2182E-05	2.6800E+00	1.4941E+00
CCNA_01427	1.5879E-04	1.6243E-04	-3.2675E-02	-2.3619E-01
CCNA_01428	1.1324E-04	1.3526E-04	-2.5645E-01	-3.7893E-01
CCNA_01429	1.5416E-04	1.9337E-04	-3.2696E-01	-4.2390E-01
CCNA_01430	8.3202E-05	1.6258E-04	-9.6642E-01	-8.3177E-01

CCNA_01431	2.2915E-04	1.4927E-04	6.1829E-01	1.7902E-01
CCNA_01432	6.4506E-05	1.0454E-04	-6.9657E-01	-6.5965E-01
CCNA_01433	2.4978E-04	1.0337E-04	1.2727E+00	5.9644E-01
CCNA_01434	1.0532E-04	1.1311E-04	-1.0294E-01	-2.8101E-01
CCNA_01435	1.7962E-04	1.0347E-04	7.9562E-01	2.9213E-01
CCNA_01436	2.0598E-04	3.1574E-04	-6.1628E-01	-6.0844E-01
CCNA_01437	3.7266E-04	1.6236E-04	1.1986E+00	5.4918E-01
CCNA_01438	3.4906E-04	1.5126E-04	1.2063E+00	5.5410E-01
CCNA_01439	1.4438E-04	1.5972E-04	-1.4563E-01	-3.0824E-01
CCNA_01440	1.4097E-04	1.5568E-04	-1.4327E-01	-3.0673E-01
CCNA_01441	7.9498E-05	6.2244E-05	3.5287E-01	9.7254E-03
CCNA_01442	6.4719E-05	1.9243E-05	1.7494E+00	9.0047E-01
CCNA_01443	8.0705E-05	1.4344E-04	-8.2973E-01	-7.4459E-01
CCNA_01444	2.8003E-04	1.5271E-04	8.7468E-01	3.4256E-01
CCNA_01445	2.4643E-04	8.3848E-05	1.5552E+00	7.7664E-01
CCNA_01446	7.7436E-05	1.2611E-04	-7.0365E-01	-6.6417E-01
CCNA_01447	1.1778E-04	5.6833E-05	1.0511E+00	4.5509E-01
CCNA_01448	2.0749E-04	1.2180E-04	7.6845E-01	2.7480E-01
CCNA_01449	5.7700E-05	1.4410E-04	-1.3204E+00	-1.0575E+00
CCNA_01450	1.6385E-04	5.3261E-05	1.6211E+00	8.1862E-01
CCNA_01451	2.0987E-04	8.6011E-05	1.2868E+00	6.0542E-01
CCNA_01452	8.1625E-05	8.5199E-05	-6.1887E-02	-2.5483E-01
CCNA_01453	1.3971E-04	3.6526E-04	-1.3864E+00	-1.0997E+00
CCNA_01454	6.5200E-05	0.0000E+00	1.2942E+01	8.0395E+00
CCNA_01455	7.0083E-05	3.2402E-06	4.4313E+00	2.6111E+00
CCNA_01456	9.5599E-05	1.3973E-04	-5.4757E-01	-5.6461E-01
CCNA_01457	6.9672E-05	8.8754E-06	2.9714E+00	1.6800E+00
CCNA_01458	8.9222E-05	1.1304E-04	-3.4133E-01	-4.3306E-01
CCNA_01459	1.1043E-04	8.7909E-05	3.2902E-01	-5.4915E-03
CCNA_01460	6.7535E-05	6.5567E-05	4.2585E-02	-1.8819E-01
CCNA_01461	8.2124E-05	1.1428E-04	-4.7669E-01	-5.1941E-01
CCNA_01462	4.9182E-05	3.7872E-06	3.6959E+00	2.1421E+00
CCNA_01463	2.6932E-04	2.5888E-04	5.7011E-02	-1.7899E-01
CCNA_01464	1.8503E-04	1.4312E-04	3.7055E-01	2.0998E-02
CCNA_01465	1.7762E-04	1.5719E-04	1.7623E-01	-1.0294E-01
CCNA_01466	9.4387E-04	2.2135E-05	5.4137E+00	3.2377E+00
CCNA_01467	1.5098E-03	1.1777E-04	3.6803E+00	2.1321E+00
CCNA_01468	7.3292E-05	6.3346E-05	2.1031E-01	-8.1210E-02
CCNA_01469	7.8906E-05	3.7176E-05	1.0855E+00	4.7705E-01
CCNA_01470	6.6185E-05	6.4730E-05	3.1983E-02	-1.9495E-01
CCNA_01471	4.3753E-05	8.1528E-05	-8.9789E-01	-7.8807E-01
CCNA_01472	6.0132E-05	2.0983E-05	1.5185E+00	7.5319E-01
CCNA_01473	4.7943E-05	9.9577E-05	-1.0545E+00	-8.8794E-01
CCNA_01474	1.3451E-04	9.7456E-06	3.7856E+00	2.1993E+00
CCNA_01475	3.0372E-04	1.0051E-04	1.5954E+00	8.0223E-01
CCNA_01476	7.7664E-04	1.0587E-04	2.8749E+00	1.6184E+00
CCNA_01477	8.0917E-05	1.0372E-04	-3.5821E-01	-4.4384E-01
CCNA_01478	1.5422E-04	1.1667E-04	4.0245E-01	4.1346E-02
CCNA_01479	1.7959E-04	5.3733E-05	1.7406E+00	8.9491E-01
CCNA_01480	1.2141E-04	1.3251E-05	3.1948E+00	1.8224E+00
CCNA_01481	1.2873E-04	1.3083E-04	-2.3349E-02	-2.3024E-01
CCNA_01482	1.1654E-04	6.5161E-05	8.3864E-01	3.1957E-01
CCNA_01483	5.2627E-05	8.6301E-05	-7.1359E-01	-6.7051E-01

CCNA_01484	1.0211E-04	4.7186E-05	1.1134E+00	4.9484E-01
CCNA_01485	5.6706E-05	1.4347E-04	-1.3391E+00	-1.0695E+00
CCNA_01486	1.4692E-04	7.9183E-05	8.9164E-01	3.5338E-01
CCNA_01487	1.4844E-04	9.1340E-05	7.0051E-01	2.3146E-01
CCNA_01488	1.1022E-04	3.5079E-05	1.6514E+00	8.3800E-01
CCNA_01489	6.0211E-05	8.0542E-05	-4.1976E-01	-4.8309E-01
CCNA_01490	6.4553E-05	1.5032E-04	-1.2195E+00	-9.9317E-01
CCNA_01491	7.1697E-05	8.5348E-05	-2.5150E-01	-3.7577E-01
CCNA_01492	3.3583E-04	1.4246E-04	1.2371E+00	5.7372E-01
CCNA_01493	5.1291E-05	5.1015E-05	7.6704E-03	-2.1046E-01
CCNA_01494	6.4187E-05	1.2901E-04	-1.0071E+00	-8.5775E-01
CCNA_01495	1.4325E-04	1.5039E-04	-7.0200E-02	-2.6013E-01
CCNA_01496	1.5893E-04	1.5695E-04	1.8034E-02	-2.0385E-01
CCNA_01497	5.5300E-05	1.4966E-04	-1.4363E+00	-1.1315E+00
CCNA_01498	7.9734E-05	2.7331E-05	1.5443E+00	7.6968E-01
CCNA_01499	4.7198E-05	1.1644E-04	-1.3028E+00	-1.0463E+00
CCNA_01500	6.9084E-05	8.5009E-05	-2.9929E-01	-4.0625E-01
CCNA_01501	9.5252E-05	1.3072E-04	-4.5668E-01	-5.0664E-01
CCNA_01503	7.6668E-05	8.4263E-05	-1.3633E-01	-3.0231E-01
CCNA_01502	7.8161E-05	1.6243E-04	-1.0552E+00	-8.8843E-01
CCNA_01504	1.4328E-04	9.5856E-05	5.7984E-01	1.5450E-01
CCNA_01505	8.9185E-05	1.6330E-04	-8.7268E-01	-7.7199E-01
CCNA_01506	4.8095E-05	1.2714E-04	-1.4024E+00	-1.1099E+00
CCNA_01507	1.7356E-04	1.4664E-04	2.4310E-01	-6.0295E-02
CCNA_01508	1.6132E-04	1.5985E-04	1.3157E-02	-2.0696E-01
CCNA_01509	1.1178E-04	1.4735E-04	-3.9864E-01	-4.6962E-01
CCNA_01510	1.1082E-04	1.5023E-04	-4.3899E-01	-4.9536E-01
CCNA_01511	5.8953E-05	2.7596E-05	1.0948E+00	4.8295E-01
CCNA_01512	6.0511E-05	9.3171E-05	-6.2270E-01	-6.1254E-01
CCNA_01513	8.1065E-05	1.5846E-04	-9.6692E-01	-8.3210E-01
CCNA_01514	7.8564E-05	6.1448E-05	3.5438E-01	1.0686E-02
CCNA_01515	5.6123E-05	9.4025E-05	-7.4446E-01	-6.9020E-01
CCNA_01516	4.8632E-05	4.5272E-05	1.0315E-01	-1.4956E-01
CCNA_01517	8.1172E-05	1.3448E-04	-7.2837E-01	-6.7994E-01
CCNA_04008	4.8116E-04	1.2590E-04	1.9342E+00	1.0184E+00
CCNA_01519	4.1692E-04	9.4298E-05	2.1443E+00	1.1524E+00
CCNA_01520	6.0835E-05	6.6744E-05	-1.3381E-01	-3.0070E-01
CCNA_01521	7.6649E-05	9.4564E-05	-3.0305E-01	-4.0865E-01
CCNA_01522	1.1825E-04	1.9709E-04	-7.3699E-01	-6.8544E-01
CCNA_01523	9.0768E-04	6.9015E-05	3.7170E+00	2.1555E+00
CCNA_01524	4.8213E-04	1.1843E-04	2.0253E+00	1.0765E+00
CCNA_01525	4.8266E-04	1.1842E-04	2.0270E+00	1.0775E+00
CCNA_01526	2.3094E-04	1.2410E-04	8.9595E-01	3.5612E-01
CCNA_01527	1.0170E-04	6.4059E-05	6.6677E-01	2.0994E-01
CCNA_01528	6.7027E-05	2.4944E-06	4.7433E+00	2.8101E+00
CCNA_01529	1.7021E-04	1.8977E-05	3.1644E+00	1.8030E+00
CCNA_01530	2.8170E-04	1.4371E-04	9.7097E-01	4.0397E-01
CCNA_01531	4.0481E-04	7.1443E-05	2.5022E+00	1.3807E+00
CCNA_01532	6.9790E-04	2.3795E-04	1.5523E+00	7.7477E-01
CCNA_01533	1.4988E-04	1.1628E-04	3.6607E-01	1.8143E-02
CCNA_01534	1.2944E-04	7.5561E-05	7.7644E-01	2.7989E-01
CCNA_01535	2.7441E-04	7.6780E-05	1.8374E+00	9.5662E-01
CCNA_01536	1.0092E-04	4.1435E-06	4.6033E+00	2.7209E+00

CCNA_01537	3.0095E-04	1.5788E-04	9.3068E-01	3.7828E-01
CCNA_01538	2.8639E-04	1.6243E-04	8.1817E-01	3.0651E-01
CCNA_01539	8.2379E-05	4.5173E-05	8.6664E-01	3.3743E-01
CCNA_01540	7.7759E-05	3.0115E-05	1.3682E+00	6.5735E-01
CCNA_01541	8.4945E-05	1.6176E-05	2.3920E+00	1.3104E+00
CCNA_01542	4.6497E-04	3.8236E-05	3.6038E+00	2.0833E+00
CCNA_01543	7.2959E-05	1.4238E-04	-9.6457E-01	-8.3060E-01
CCNA_01544	6.7480E-05	1.4326E-04	-1.0861E+00	-9.0810E-01
CCNA_01545	7.6645E-05	1.5625E-04	-1.0276E+00	-8.7080E-01
CCNA_01546	2.2319E-04	2.0239E-04	1.4109E-01	-1.2536E-01
CCNA_01547	1.0696E-04	7.8163E-05	4.5242E-01	7.3222E-02
CCNA_01548	5.7311E-05	2.9626E-05	9.5166E-01	3.9166E-01
CCNA_01549	6.4654E-05	1.0150E-04	-6.5067E-01	-6.3038E-01
CCNA_01550	9.6718E-05	1.3051E-04	-4.3236E-01	-4.9113E-01
CCNA_01551	9.9812E-05	1.2208E-04	-2.9063E-01	-4.0073E-01
CCNA_01552	2.2161E-04	1.6243E-04	4.4817E-01	7.0511E-02
CCNA_01553	5.4181E-05	1.2646E-05	2.0983E+00	1.1230E+00
CCNA_01554	5.1503E-05	7.5760E-05	-5.5680E-01	-5.7050E-01
CCNA_01555	4.6973E-04	8.0434E-05	2.5458E+00	1.4085E+00
CCNA_01556	4.9104E-04	1.0196E-04	2.2678E+00	1.2311E+00
CCNA_01557	7.6095E-05	1.3741E-04	-8.5260E-01	-7.5917E-01
CCNA_01558	6.4127E-05	1.3407E-04	-1.0639E+00	-8.9398E-01
CCNA_01559	4.5862E-05	9.8458E-05	-1.1022E+00	-9.1838E-01
CCNA_01560	4.6422E-05	4.6548E-05	-4.0489E-03	-2.1793E-01
CCNA_01561	1.0044E-04	1.1161E-04	-1.5217E-01	-3.1241E-01
CCNA_01562	6.2907E-05	5.9112E-05	8.9674E-02	-1.5815E-01
CCNA_01563	5.9156E-05	8.7055E-05	-5.5743E-01	-5.7090E-01
CCNA_01564	3.5407E-05	1.0156E-04	-1.5201E+00	-1.1850E+00
CCNA_01565	4.8433E-05	6.2236E-06	2.9584E+00	1.6716E+00
CCNA_01566	6.8442E-05	1.2300E-04	-8.4567E-01	-7.5476E-01
CCNA_01567	7.9160E-05	1.1285E-04	-5.1162E-01	-5.4168E-01
CCNA_01568	1.0026E-04	2.5682E-05	1.9645E+00	1.0377E+00
CCNA_01569	4.1446E-05	6.4092E-05	-6.2894E-01	-6.1651E-01
CCNA_01570	4.4410E-05	1.0206E-04	-1.2004E+00	-9.8099E-01
CCNA_01571	5.2807E-05	1.2558E-04	-1.2498E+00	-1.0125E+00
CCNA_01572	5.8971E-05	5.4164E-05	1.2257E-01	-1.3717E-01
CCNA_01573	7.2072E-05	1.2874E-04	-8.3695E-01	-7.4919E-01
CCNA_01574	7.5082E-05	1.3862E-04	-8.8457E-01	-7.7957E-01
CCNA_01575	7.2668E-05	4.3391E-05	7.4374E-01	2.5904E-01
CCNA_01576	8.6952E-05	8.1619E-05	9.1241E-02	-1.5715E-01
CCNA_01577	2.1115E-04	1.8763E-04	1.7034E-01	-1.0670E-01
CCNA_01578	2.1018E-04	1.9230E-04	1.2820E-01	-1.3358E-01
CCNA_01579	7.0564E-05	1.3009E-04	-8.8250E-01	-7.7825E-01
CCNA_01580	1.1546E-04	1.2325E-04	-9.4334E-02	-2.7552E-01
CCNA_01581	1.0269E-04	1.6243E-04	-6.6146E-01	-6.3726E-01
CCNA_01582	4.4863E-05	2.6403E-05	7.6455E-01	2.7231E-01
CCNA_01583	1.0456E-04	8.1420E-05	3.6080E-01	1.4780E-02
CCNA_01584	6.6708E-05	1.7651E-06	5.2333E+00	3.1227E+00
CCNA_01585	6.4881E-05	1.0302E-04	-6.6713E-01	-6.4088E-01
CCNA_01586	6.3272E-05	8.2663E-05	-3.8572E-01	-4.6138E-01
CCNA_01587	5.1642E-05	2.5193E-05	1.0352E+00	4.4494E-01
CCNA_01588	5.5822E-05	6.3827E-05	-1.9339E-01	-3.3870E-01
CCNA_01589	1.2631E-04	7.9713E-05	6.6396E-01	2.0815E-01

CCNA_01590	1.1743E-04	1.0640E-04	1.4228E-01	-1.2460E-01
CCNA_01591	5.4389E-05	1.5816E-04	-1.5399E+00	-1.1976E+00
CCNA_01592	5.5929E-05	1.4734E-04	-1.3975E+00	-1.1067E+00
CCNA_01593	1.3902E-04	1.3810E-04	9.5454E-03	-2.0926E-01
CCNA_01594	8.7997E-05	6.7407E-05	3.8446E-01	2.9872E-02
CCNA_01595	8.6332E-05	4.8637E-05	8.2769E-01	3.1258E-01
CCNA_01596	2.3695E-04	7.4882E-05	1.6618E+00	8.4461E-01
CCNA_01597	2.2660E-04	6.1109E-05	1.8905E+00	9.9049E-01
CCNA_01598	2.2662E-04	2.9162E-05	2.9577E+00	1.6712E+00
CCNA_01599	2.3675E-04	1.6243E-04	5.4357E-01	1.3136E-01
CCNA_01600	9.6455E-05	9.7862E-05	-2.0949E-02	-2.2871E-01
CCNA_01601	9.2552E-05	1.2968E-04	-4.8669E-01	-5.2578E-01
CCNA_01602	7.4619E-05	1.3640E-05	2.4509E+00	1.3479E+00
CCNA_01603	1.8929E-04	7.1012E-05	1.4143E+00	6.8676E-01
CCNA_01604	1.9166E-04	4.6830E-05	2.0328E+00	1.0812E+00
CCNA_01605	7.9068E-05	1.3471E-04	-7.6866E-01	-7.0563E-01
CCNA_01606	2.9012E-04	1.6267E-04	8.3469E-01	3.1705E-01
CCNA_01607	2.9205E-04	1.6267E-04	8.4423E-01	3.2313E-01
CCNA_01608	3.9434E-05	8.5796E-05	-1.1214E+00	-9.3064E-01
CCNA_01609	5.5540E-05	1.2218E-04	-1.1374E+00	-9.4085E-01
CCNA_01610	1.0134E-04	1.1359E-04	-1.6465E-01	-3.2037E-01
CCNA_01611	5.6447E-05	1.3158E-04	-1.2210E+00	-9.9414E-01
CCNA_01612	2.6616E-04	1.2047E-04	1.1436E+00	5.1407E-01
CCNA_01613	6.1552E-05	9.3901E-05	-6.0935E-01	-6.0402E-01
CCNA_01614	4.2903E-05	4.4195E-05	-4.2928E-02	-2.4273E-01
CCNA_01615	5.7575E-05	1.6029E-04	-1.4771E+00	-1.1575E+00
CCNA_01616	4.8391E-05	4.0109E-06	3.5899E+00	2.0744E+00
CCNA_01617	6.6042E-05	1.0056E-04	-6.0655E-01	-6.0224E-01
CCNA_01618	5.9221E-05	5.7305E-05	4.7356E-02	-1.8515E-01
CCNA_01619	1.9523E-04	2.4389E-05	3.0005E+00	1.6985E+00
CCNA_01620	1.7701E-04	8.6268E-05	1.0368E+00	4.4596E-01
CCNA_01621	2.8438E-04	4.2449E-04	-5.7791E-01	-5.8397E-01
CCNA_01622	2.3896E-04	5.0510E-05	2.2419E+00	1.2146E+00
CCNA_01623	3.2073E-04	7.3166E-05	2.1320E+00	1.1445E+00
CCNA_01624	2.9031E-04	7.3456E-05	1.9825E+00	1.0492E+00
CCNA_01625	8.5181E-05	8.7321E-05	-3.5851E-02	-2.3822E-01
CCNA_01626	8.3456E-05	8.0600E-05	5.0172E-02	-1.8335E-01
CCNA_01627	4.6297E-05	5.9335E-05	-3.5804E-01	-4.4372E-01
CCNA_01628	4.8132E-05	9.8077E-05	-1.0269E+00	-8.7035E-01
CCNA_01629	1.1157E-04	1.5782E-04	-5.0032E-01	-5.3448E-01
CCNA_01630	9.1530E-05	1.1255E-04	-2.9824E-01	-4.0558E-01
CCNA_01631	8.5491E-05	9.7646E-05	-1.9184E-01	-3.3772E-01
CCNA_01632	1.7226E-04	1.6183E-04	9.0098E-02	-1.5788E-01
CCNA_01633	1.7294E-04	1.4194E-04	2.8492E-01	-3.3619E-02
CCNA_01634	6.6536E-05	1.2180E-04	-8.7233E-01	-7.7176E-01
CCNA_01635	6.5829E-05	1.9549E-05	1.7511E+00	9.0158E-01
CCNA_01636	6.4752E-05	3.0074E-05	1.1061E+00	4.9018E-01
CCNA_01637	5.8185E-04	1.4240E-04	2.0307E+00	1.0799E+00
CCNA_01638	5.6410E-05	6.3205E-05	-1.6418E-01	-3.2007E-01
CCNA_01639	4.6699E-05	1.0549E-05	2.1452E+00	1.1530E+00
CCNA_01640	5.5901E-05	1.3814E-04	-1.3051E+00	-1.0478E+00
CCNA_01641	5.4532E-05	1.0611E-04	-9.6033E-01	-8.2789E-01
CCNA_01642	4.3097E-05	8.3359E-05	-9.5175E-01	-8.2242E-01

CCNA_01643	8.0238E-05	2.9651E-05	1.4359E+00	7.0051E-01
CCNA_01644	1.4624E-04	1.7124E-04	-2.2776E-01	-3.6063E-01
CCNA_01645	1.5399E-04	1.6243E-04	-7.6955E-02	-2.6444E-01
CCNA_01646	4.1427E-05	1.3069E-05	1.6637E+00	8.4584E-01
CCNA_01647	7.6483E-05	1.3275E-04	-7.9551E-01	-7.2276E-01
CCNA_01648	7.1979E-05	7.3092E-05	-2.2203E-02	-2.2951E-01
CCNA_01649	6.8118E-05	5.5962E-05	2.8346E-01	-3.4549E-02
CCNA_01650	2.8863E-04	6.4813E-05	2.1547E+00	1.1590E+00
CCNA_01651	2.6991E-04	8.8630E-05	1.6065E+00	8.0934E-01
CCNA_01652	1.0559E-04	1.6142E-04	-6.1235E-01	-6.0594E-01
CCNA_01653	8.5583E-05	1.1322E-04	-4.0373E-01	-4.7287E-01
CCNA_01654	8.9671E-05	1.3409E-04	-5.8053E-01	-5.8564E-01
CCNA_01655	1.3925E-04	1.0849E-04	3.6007E-01	1.4316E-02
CCNA_01656	9.2547E-05	2.7985E-05	1.7252E+00	8.8503E-01
CCNA_01657	2.1579E-04	7.4318E-05	1.5377E+00	7.6546E-01
CCNA_01658	1.6977E-04	9.8392E-05	7.8692E-01	2.8658E-01
CCNA_01659	7.7135E-05	1.1310E-04	-5.5218E-01	-5.6756E-01
CCNA_01660	3.9015E-04	4.8933E-04	-3.2679E-01	-4.2379E-01
CCNA_01661	1.5134E-04	1.0776E-04	4.8982E-01	9.7078E-02
CCNA_01662	3.3343E-04	3.4115E-04	-3.3044E-02	-2.3643E-01
CCNA_01663	1.1210E-04	1.6150E-04	-5.2677E-01	-5.5135E-01
CCNA_01664	4.8151E-05	1.3199E-04	-1.4547E+00	-1.1432E+00
CCNA_01665	1.3597E-04	3.1557E-05	2.1069E+00	1.1285E+00
CCNA_01666	4.9460E-05	5.7902E-05	-2.2742E-01	-3.6041E-01
CCNA_01667	8.2406E-05	4.2165E-05	9.6653E-01	4.0114E-01
CCNA_01668	1.8650E-04	1.5314E-05	3.6054E+00	2.0843E+00
CCNA_01669	4.8687E-05	4.8993E-05	-9.1364E-03	-2.2118E-01
CCNA_01670	6.7887E-05	6.2136E-05	1.2760E-01	-1.3396E-01
CCNA_01671	1.4438E-04	1.1833E-04	2.8698E-01	-3.2304E-02
CCNA_01672	8.7840E-05	5.7106E-05	6.2110E-01	1.8081E-01
CCNA_01673	6.5778E-05	1.3418E-04	-1.0284E+00	-8.7133E-01
CCNA_01674	1.7268E-04	1.6281E-04	8.4888E-02	-1.6121E-01
CCNA_01675	1.7398E-04	1.5538E-04	1.6308E-01	-1.1133E-01
CCNA_01676	6.1320E-05	4.2488E-05	5.2915E-01	1.2216E-01
CCNA_01677	3.1903E-04	1.4463E-04	1.1413E+00	5.1263E-01
CCNA_01678	5.1097E-05	2.1604E-05	1.2415E+00	5.7652E-01
CCNA_01679	6.8358E-05	1.2960E-04	-9.2288E-01	-8.0401E-01
CCNA_01680	6.1713E-05	5.4139E-05	1.8880E-01	-9.4929E-02
CCNA_01681	7.1188E-05	6.1473E-05	2.1158E-01	-8.0397E-02
CCNA_01682	1.0447E-04	4.6814E-05	1.1579E+00	5.2323E-01
CCNA_01683	8.9911E-05	8.9243E-05	1.0701E-02	-2.0853E-01
CCNA_01684	1.2391E-03	3.0370E-04	2.0286E+00	1.0786E+00
CCNA_01685	4.5923E-04	1.4942E-04	1.6197E+00	8.1778E-01
CCNA_01686	6.0244E-04	1.2973E-04	2.2152E+00	1.1976E+00
CCNA_01687	9.0956E-05	3.5154E-05	1.3712E+00	6.5928E-01
CCNA_01688	6.5219E-05	9.3113E-05	-5.1373E-01	-5.4303E-01
CCNA_01689	3.7802E-04	3.2485E-04	2.1867E-01	-7.5877E-02
CCNA_01690	5.0010E-05	4.8952E-05	3.0746E-02	-1.9574E-01
CCNA_01691	4.7541E-05	7.6564E-05	-6.8752E-01	-6.5388E-01
CCNA_01692	6.7077E-05	1.1734E-04	-8.0685E-01	-7.3000E-01
CCNA_01693	5.1850E-05	4.9673E-05	6.1790E-02	-1.7594E-01
CCNA_01694	6.8862E-05	1.0725E-04	-6.3922E-01	-6.2307E-01
CCNA_01695	6.3808E-05	2.4074E-05	1.4059E+00	6.8138E-01

CCNA_01696	6.4123E-05	1.0593E-04	-7.2415E-01	-6.7724E-01
CCNA_01697	7.3274E-05	6.5625E-05	1.5896E-01	-1.1396E-01
CCNA_01698	1.2551E-04	1.5580E-04	-3.1186E-01	-4.1427E-01
CCNA_01699	2.3944E-04	1.2828E-04	9.0034E-01	3.5892E-01
CCNA_01700	2.3352E-04	1.1986E-04	9.6217E-01	3.9836E-01
CCNA_01701	5.4722E-05	4.7767E-05	1.9599E-01	-9.0342E-02
CCNA_01702	5.8990E-05	4.1502E-05	5.0713E-01	1.0812E-01
CCNA_01703	5.6220E-05	5.3195E-05	7.9700E-02	-1.6452E-01
CCNA_01704	9.0577E-05	1.4752E-04	-7.0368E-01	-6.6419E-01
CCNA_01705	3.5754E-04	1.4593E-04	1.2927E+00	6.0920E-01
CCNA_01706	3.6734E-04	1.6166E-04	1.1841E+00	5.3991E-01
CCNA_01707	5.3200E-05	5.5283E-05	-5.5484E-02	-2.5074E-01
CCNA_01708	5.2761E-05	1.1099E-04	-1.0728E+00	-8.9965E-01
CCNA_01709	6.1811E-05	1.0193E-06	5.9106E+00	3.5547E+00
CCNA_01710	4.4549E-05	5.7976E-05	-3.8013E-01	-4.5782E-01
CCNA_01711	7.7348E-05	5.5656E-05	4.7470E-01	8.7430E-02
CCNA_01712	3.0716E-04	1.1452E-04	1.4233E+00	6.9250E-01
CCNA_01713	2.8463E-04	8.3351E-05	1.7717E+00	9.1472E-01
CCNA_01714	2.0773E-04	6.3421E-05	1.7115E+00	8.7633E-01
CCNA_01715	3.3900E-04	1.8678E-04	8.5990E-01	3.3313E-01
CCNA_01716	5.4874E-05	1.2921E-04	-1.2355E+00	-1.0034E+00
CCNA_01717	5.5531E-05	4.3946E-05	3.3740E-01	-1.4125E-04
CCNA_01718	5.7445E-05	7.9332E-05	-4.6574E-01	-5.1242E-01
CCNA_01719	9.2159E-05	3.8659E-05	1.2531E+00	5.8391E-01
CCNA_04016	6.6407E-05	9.6204E-05	-5.3479E-01	-5.5646E-01
CCNA_01721	7.4527E-05	8.1263E-05	-1.2489E-01	-2.9501E-01
CCNA_01722	5.4361E-05	7.3647E-05	-4.3809E-01	-4.9478E-01
CCNA_01723	6.2190E-05	5.6625E-05	1.3512E-01	-1.2916E-01
CCNA_01724	2.1447E-04	1.6272E-04	3.9838E-01	3.8755E-02
CCNA_01725	1.1335E-04	3.1565E-05	1.8441E+00	9.6087E-01
CCNA_01726	1.0005E-04	6.5277E-05	6.1592E-01	1.7751E-01
CCNA_01727	3.9488E-04	1.1592E-04	1.7682E+00	9.1249E-01
CCNA_01728	8.7498E-05	1.3203E-04	-5.9356E-01	-5.9395E-01
CCNA_01729	1.3065E-04	7.1385E-05	8.7195E-01	3.4082E-01
CCNA_01730	2.1696E-04	5.2283E-05	2.0528E+00	1.0940E+00
CCNA_01731	7.6612E-05	1.4650E-04	-9.3523E-01	-8.1188E-01
CCNA_01732	1.8044E-04	1.4687E-04	2.9694E-01	-2.5950E-02
CCNA_01733	9.5081E-05	6.3031E-05	5.9297E-01	1.6287E-01
CCNA_01734	8.2360E-05	8.9011E-05	-1.1209E-01	-2.8685E-01
CCNA_01735	7.0472E-05	1.4433E-04	-1.0342E+00	-8.7502E-01
CCNA_01736	6.8224E-05	1.3329E-04	-9.6619E-01	-8.3163E-01
CCNA_01737	1.5963E-04	1.7820E-04	-1.5884E-01	-3.1666E-01
CCNA_01738	5.8736E-05	1.3331E-04	-1.1824E+00	-9.6954E-01
CCNA_01739	8.1967E-05	1.5509E-04	-9.2000E-01	-8.0217E-01
CCNA_01740	5.8638E-05	4.0192E-05	5.4475E-01	1.3211E-01
CCNA_01741	2.9468E-04	6.5614E-04	-1.1549E+00	-9.5198E-01
CCNA_01742	3.0681E-04	6.7704E-04	-1.1419E+00	-9.4369E-01
CCNA_01743	6.0238E-05	1.4332E-04	-1.2505E+00	-1.0130E+00
CCNA_01744	5.9258E-05	3.3720E-05	8.1317E-01	3.0332E-01
CCNA_01745	1.4925E-04	5.6700E-05	1.3962E+00	6.7518E-01
CCNA_01746	1.5130E-04	6.7399E-05	1.1665E+00	5.2866E-01
CCNA_01747	7.2677E-05	1.2455E-05	2.5439E+00	1.4072E+00
CCNA_01748	1.0470E-04	4.0789E-05	1.3598E+00	6.5201E-01

CCNA_01749	3.0953E-04	1.7082E-04	8.5756E-01	3.3164E-01
CCNA_01750	1.2658E-04	1.2099E-04	6.5059E-02	-1.7385E-01
CCNA_01751	7.0227E-05	3.3173E-05	1.0817E+00	4.7463E-01
CCNA_01752	6.2976E-05	1.5984E-04	-1.3437E+00	-1.0724E+00
CCNA_01753	5.5984E-05	1.1875E-04	-1.0848E+00	-9.0726E-01
CCNA_01754	4.2861E-05	3.2096E-05	4.1707E-01	5.0670E-02
CCNA_01755	5.7862E-05	1.4772E-04	-1.3521E+00	-1.0778E+00
CCNA_01756	4.6542E-05	1.0904E-04	-1.2282E+00	-9.9877E-01
CCNA_01757	5.0990E-05	6.0935E-05	-2.5711E-01	-3.7935E-01
CCNA_01758	4.3647E-05	1.2538E-05	1.7987E+00	9.3196E-01
CCNA_01759	1.7456E-04	1.4242E-04	2.9348E-01	-2.8158E-02
CCNA_01760	1.4257E-04	6.0844E-05	1.2283E+00	5.6811E-01
CCNA_01761	6.4789E-05	1.4823E-04	-1.1940E+00	-9.7694E-01
CCNA_01762	3.4897E-04	1.6062E-04	1.1194E+00	4.9866E-01
CCNA_01763	3.3447E-04	1.6243E-04	1.0420E+00	4.4931E-01
CCNA_01764	4.1256E-05	1.1382E-04	-1.4640E+00	-1.1492E+00
CCNA_01765	4.2223E-05	3.4209E-05	3.0346E-01	-2.1793E-02
CCNA_01766	4.6946E-04	1.0182E-04	2.2048E+00	1.1910E+00
CCNA_01767	2.6767E-04	1.4776E-04	8.5715E-01	3.3137E-01
CCNA_01768	6.7161E-05	4.7468E-05	5.0050E-01	1.0389E-01
CCNA_01769	1.2564E-04	1.6637E-04	-4.0510E-01	-4.7374E-01
CCNA_01770	1.3017E-04	1.6243E-04	-3.1937E-01	-4.1906E-01
CCNA_01771	4.0891E-05	8.0550E-06	2.3425E+00	1.2788E+00
CCNA_01772	3.7552E-05	2.9900E-05	3.2856E-01	-5.7846E-03
CCNA_01773	6.6675E-05	5.6460E-05	2.3982E-01	-6.2386E-02
CCNA_01774	3.5546E-05	1.1486E-05	1.6290E+00	8.2367E-01
CCNA_01775	3.9068E-04	1.6276E-04	1.2632E+00	5.9038E-01
CCNA_01776	3.4639E-04	1.6243E-04	1.0926E+00	4.8154E-01
CCNA_01777	1.0135E-04	1.1572E-04	-1.9131E-01	-3.3738E-01
CCNA_01778	2.2333E-04	6.8965E-05	1.6951E+00	8.6584E-01
CCNA_01779	1.1213E-04	7.7840E-05	5.2649E-01	1.2047E-01
CCNA_01780	7.3389E-05	1.3309E-04	-8.5876E-01	-7.6310E-01
CCNA_01781	4.7397E-05	6.2899E-06	2.9119E+00	1.6420E+00
CCNA_01782	5.9425E-05	7.4103E-05	-3.1852E-01	-4.1852E-01
CCNA_01783	4.7249E-05	1.0475E-04	-1.1485E+00	-9.4794E-01
CCNA_01784	1.0499E-04	0.0000E+00	1.3629E+01	8.4779E+00
CCNA_01785	9.3694E-05	9.4108E-05	-6.4137E-03	-2.1944E-01
CCNA_01786	3.2840E-05	1.6574E-06	4.3015E+00	2.5283E+00
CCNA_01787	3.0857E-05	2.8756E-05	1.0152E-01	-1.5060E-01
CCNA_01788	3.9767E-05	5.5391E-05	-4.7810E-01	-5.2031E-01
CCNA_01789	2.1564E-04	4.2428E-04	-9.7641E-01	-8.3815E-01
CCNA_01790	2.2357E-04	4.2238E-04	-9.1781E-01	-8.0077E-01
CCNA_01791	6.2111E-04	2.6831E-04	1.2109E+00	5.5702E-01
CCNA_01792	2.1853E-04	2.6320E-04	-2.6832E-01	-3.8649E-01
CCNA_01793	1.6215E-04	1.3547E-04	2.5932E-01	-4.9946E-02
CCNA_01794	1.0933E-04	1.5913E-04	-5.4148E-01	-5.6073E-01
CCNA_01795	1.0490E-04	1.6243E-04	-6.3074E-01	-6.1766E-01
CCNA_01796	1.2060E-04	1.0755E-04	1.6514E-01	-1.1002E-01
CCNA_01797	2.4461E-04	6.7324E-05	1.8611E+00	9.7175E-01
CCNA_01798	9.5063E-05	1.0181E-04	-9.9042E-02	-2.7852E-01
CCNA_01799	2.6195E-04	1.2129E-04	1.1108E+00	4.9315E-01
CCNA_01800	6.0419E-05	8.9641E-05	-5.6919E-01	-5.7840E-01
CCNA_01801	7.6478E-05	6.4324E-05	2.4959E-01	-5.6150E-02

CCNA_01802	1.3437E-04	8.1536E-05	7.2062E-01	2.4429E-01
CCNA_01803	1.1385E-04	5.9708E-05	9.3094E-01	3.7844E-01
CCNA_01804	4.6038E-05	1.4090E-04	-1.6137E+00	-1.2446E+00
CCNA_01805	7.0772E-05	1.1696E-04	-7.2471E-01	-6.7760E-01
CCNA_01806	7.9179E-05	1.3145E-04	-7.3133E-01	-6.8182E-01
CCNA_01807	2.2376E-04	6.7490E-05	1.7290E+00	8.8750E-01
CCNA_01808	2.1937E-04	1.4730E-04	5.7455E-01	1.5112E-01
CCNA_01809	2.1313E-04	1.4809E-04	5.2525E-01	1.1967E-01
CCNA_01810	7.3107E-05	1.2082E-04	-7.2474E-01	-6.7762E-01
CCNA_01811	6.7133E-05	7.6780E-05	-1.9376E-01	-3.3894E-01
CCNA_01812	9.0374E-05	4.8048E-05	9.1124E-01	3.6588E-01
CCNA_01813	1.0155E-04	2.0792E-05	2.2876E+00	1.2438E+00
CCNA_01814	4.8507E-05	8.6608E-05	-8.3631E-01	-7.4878E-01
CCNA_01815	4.6842E-05	1.1849E-04	-1.3388E+00	-1.0693E+00
CCNA_01816	1.3515E-04	1.6046E-04	-2.4766E-01	-3.7332E-01
CCNA_01817	8.4316E-05	9.8044E-05	-2.1766E-01	-3.5419E-01
CCNA_01818	5.4176E-05	1.0500E-04	-9.5461E-01	-8.2424E-01
CCNA_01819	2.1943E-04	1.3781E-04	6.7109E-01	2.1270E-01
CCNA_01820	1.5792E-04	1.6243E-04	-4.0595E-02	-2.4124E-01
CCNA_01821	1.0261E-04	1.1232E-04	-1.3053E-01	-2.9861E-01
CCNA_01822	4.7291E-05	1.0636E-04	-1.1694E+00	-9.6121E-01
CCNA_01823	5.3658E-05	2.0883E-06	4.6778E+00	2.7683E+00
CCNA_01824	7.9331E-05	1.5325E-04	-9.4994E-01	-8.2126E-01
CCNA_01825	6.9870E-05	1.2547E-04	-8.4454E-01	-7.5404E-01
CCNA_01826	1.1599E-04	1.0623E-04	1.2675E-01	-1.3451E-01
CCNA_01827	4.6713E-05	1.0293E-04	-1.1398E+00	-9.4236E-01
CCNA_01828	8.5801E-05	6.1432E-05	4.8188E-01	9.2013E-02
CCNA_01829	8.2864E-05	4.3035E-05	9.4505E-01	3.8744E-01
CCNA_01830	3.8681E-04	1.3087E-04	1.5634E+00	7.8186E-01
CCNA_01831	5.0801E-05	1.2109E-04	-1.2531E+00	-1.0147E+00
CCNA_01832	4.6218E-05	1.5459E-04	-1.7419E+00	-1.3264E+00
CCNA_01833	6.0729E-05	3.1623E-05	9.4112E-01	3.8493E-01
CCNA_01834	4.1686E-05	1.2449E-04	-1.5783E+00	-1.2221E+00
CCNA_01835	6.8525E-05	1.3405E-04	-9.6808E-01	-8.3283E-01
CCNA_01836	6.5427E-05	8.9997E-05	-4.6003E-01	-5.0878E-01
CCNA_01837	1.2861E-04	8.0268E-05	6.8001E-01	2.1839E-01
CCNA_01838	5.1938E-05	8.4080E-05	-6.9499E-01	-6.5865E-01
CCNA_01839	1.0593E-04	1.3922E-05	2.9269E+00	1.6516E+00
CCNA_01840	6.7619E-05	1.7643E-05	1.9377E+00	1.0206E+00
CCNA_01841	6.6675E-05	5.1960E-06	3.6795E+00	2.1316E+00
CCNA_01842	6.0294E-05	6.1954E-05	-3.9267E-02	-2.4040E-01
CCNA_01843	4.4188E-05	1.4045E-04	-1.6683E+00	-1.2794E+00
CCNA_01844	2.3126E-04	1.5706E-04	5.5809E-01	1.4062E-01
CCNA_01845	6.3397E-05	1.0368E-04	-7.0966E-01	-6.6800E-01
CCNA_01846	4.3074E-05	8.3301E-05	-9.5152E-01	-8.2227E-01
CCNA_01847	8.8788E-05	7.1318E-05	3.1599E-01	-1.3797E-02
CCNA_01848	8.7414E-05	9.5956E-05	-1.3455E-01	-3.0117E-01
CCNA_01849	5.6562E-05	1.0054E-04	-8.2984E-01	-7.4466E-01
CCNA_01850	8.5024E-05	8.3020E-05	3.4346E-02	-1.9344E-01
CCNA_01851	7.6472E-04	1.0013E-04	2.9329E+00	1.6554E+00
CCNA_01852	7.6022E-04	1.0675E-04	2.8321E+00	1.5911E+00
CCNA_01853	4.9191E-04	1.6243E-04	1.5986E+00	8.0428E-01
CCNA_01854	8.1741E-05	1.0797E-04	-4.0156E-01	-4.7148E-01

CCNA_01855	5.5993E-05	1.1441E-04	-1.0309E+00	-8.7289E-01
CCNA_01856	5.0976E-05	2.9063E-05	8.1038E-01	3.0154E-01
CCNA_01857	4.0308E-05	2.8259E-06	3.8302E+00	2.2277E+00
CCNA_01858	4.5603E-05	9.4555E-05	-1.0520E+00	-8.8637E-01
CCNA_01859	5.0611E-05	1.0261E-04	-1.0196E+00	-8.6572E-01
CCNA_01860	6.2255E-05	4.8372E-05	3.6388E-01	1.6747E-02
CCNA_01861	5.6511E-05	1.3783E-04	-1.2862E+00	-1.0358E+00
CCNA_01862	6.9436E-05	1.4958E-05	2.2140E+00	1.1969E+00
CCNA_01863	1.0531E-04	1.2368E-04	-2.3206E-01	-3.6337E-01
CCNA_01864	9.2603E-05	9.1771E-05	1.2960E-02	-2.0709E-01
CCNA_01865	1.6153E-04	0.0000E+00	1.4251E+01	8.8743E+00
CCNA_01866	4.2570E-05	1.5000E-05	1.5043E+00	7.4413E-01
CCNA_01867	5.4292E-05	9.4182E-05	-7.9473E-01	-7.2226E-01
CCNA_01868	5.3617E-05	1.3346E-04	-1.3157E+00	-1.0545E+00
CCNA_01869	8.9865E-05	2.4115E-05	1.8974E+00	9.9488E-01
CCNA_01870	4.6852E-05	8.7743E-05	-9.0518E-01	-7.9272E-01
CCNA_01871	5.1337E-05	6.4995E-05	-3.4039E-01	-4.3247E-01
CCNA_01872	4.6828E-05	9.1232E-05	-9.6214E-01	-8.2905E-01
CCNA_01873	4.6838E-05	1.0839E-04	-1.2104E+00	-9.8740E-01
CCNA_01874	5.2257E-05	5.1976E-05	7.6709E-03	-2.1046E-01
CCNA_01875	5.5758E-05	0.0000E+00	1.2716E+01	7.8955E+00
CCNA_01876	9.4309E-05	9.1050E-05	5.0678E-02	-1.8303E-01
CCNA_01877	9.4346E-05	9.1464E-05	4.4694E-02	-1.8684E-01
CCNA_01878	5.7117E-05	5.5424E-05	4.3319E-02	-1.8772E-01
CCNA_01879	5.9836E-05	4.6432E-05	3.6574E-01	1.7933E-02
CCNA_01880	4.9834E-05	4.6573E-06	3.4171E+00	1.9642E+00
CCNA_01881	6.5491E-05	4.8098E-05	4.4518E-01	6.8604E-02
CCNA_01882	1.1123E-04	1.2135E-04	-1.2559E-01	-2.9546E-01
CCNA_01883	1.0293E-04	1.0578E-04	-3.9355E-02	-2.4045E-01
CCNA_01884	5.7949E-05	3.3148E-06	4.1243E+00	2.4153E+00
CCNA_01885	4.3259E-05	8.8240E-05	-1.0284E+00	-8.7133E-01
CCNA_01886	5.8657E-05	7.3796E-05	-3.3129E-01	-4.2666E-01
CCNA_01887	6.5015E-05	2.1132E-06	4.9377E+00	2.9341E+00
CCNA_01888	5.8389E-05	6.3255E-05	-1.1556E-01	-2.8906E-01
CCNA_01889	7.1295E-05	7.6721E-05	-1.0590E-01	-2.8290E-01
CCNA_01890	6.9454E-05	8.5257E-05	-2.9580E-01	-4.0403E-01
CCNA_01891	5.1457E-05	6.5410E-05	-3.4618E-01	-4.3616E-01
CCNA_01892	5.6100E-05	3.7557E-05	5.7872E-01	1.5378E-01
CCNA_01893	5.1217E-05	5.7305E-05	-1.6212E-01	-3.1876E-01
CCNA_01894	6.0386E-05	9.9950E-05	-7.2699E-01	-6.7906E-01
CCNA_01895	5.5924E-05	1.3644E-04	-1.2867E+00	-1.0360E+00
CCNA_01896	1.4024E-04	1.5372E-04	-1.3245E-01	-2.9983E-01
CCNA_01897	4.8641E-05	7.4666E-05	-6.1830E-01	-6.0973E-01
CCNA_01898	7.4323E-05	7.1376E-05	5.8294E-02	-1.7817E-01
CCNA_01899	7.0208E-05	1.2174E-05	2.5270E+00	1.3965E+00
CCNA_01900	8.0538E-05	7.9348E-05	2.1404E-02	-2.0170E-01
CCNA_01901	5.0894E-04	1.8285E-04	1.4768E+00	7.2663E-01
CCNA_01902	3.0680E-04	7.4608E-05	2.0397E+00	1.0857E+00
CCNA_01903	1.8393E-04	5.9733E-05	1.6224E+00	8.1947E-01
CCNA_01904	1.5037E-04	9.2475E-05	7.0127E-01	2.3195E-01
CCNA_01905	7.4795E-05	1.6140E-04	-1.1096E+00	-9.2310E-01
CCNA_01906	7.9368E-05	1.2268E-04	-6.2829E-01	-6.1610E-01
CCNA_01907	1.3306E-04	1.6213E-04	-2.8504E-01	-3.9716E-01

CCNA_01908	5.7695E-05	1.3380E-04	-1.2136E+00	-9.8941E-01
CCNA_01909	7.4878E-05	6.2700E-05	2.5599E-01	-5.2073E-02
CCNA_01910	1.3937E-04	4.9971E-06	4.7993E+00	2.8458E+00
CCNA_01911	2.7264E-04	1.0193E-04	1.4193E+00	6.8996E-01
CCNA_01912	2.2668E-04	8.1893E-05	1.4688E+00	7.2148E-01
CCNA_01913	2.2273E-04	5.1686E-05	2.1072E+00	1.1287E+00
CCNA_01914	1.2506E-04	9.7348E-05	3.6132E-01	1.5114E-02
CCNA_01915	6.6527E-05	8.5274E-05	-3.5820E-01	-4.4382E-01
CCNA_04000	6.4201E-05	1.1060E-04	-7.8467E-01	-7.1585E-01
CCNA_01916	1.3925E-04	9.0453E-05	6.2236E-01	1.8161E-01
CCNA_01917	3.2135E-04	1.2671E-04	1.3426E+00	6.4099E-01
CCNA_01918	3.1955E-04	1.2145E-04	1.3957E+00	6.7486E-01
CCNA_01919	5.7251E-05	6.0272E-05	-7.4256E-02	-2.6272E-01
CCNA_01920	8.6476E-05	4.1352E-05	1.0641E+00	4.6338E-01
CCNA_01921	1.1593E-04	1.6161E-04	-4.7930E-01	-5.2107E-01
CCNA_01922	3.4870E-04	1.5537E-04	1.1662E+00	5.2850E-01
CCNA_01923	3.4921E-04	1.6271E-04	1.1018E+00	4.8740E-01
CCNA_01924	6.4340E-05	1.9226E-05	1.7421E+00	8.9586E-01
CCNA_01925	2.0480E-04	2.4023E-04	-2.3022E-01	-3.6220E-01
CCNA_01926	2.0485E-04	2.4240E-04	-2.4283E-01	-3.7024E-01
CCNA_01927	1.3454E-04	2.8590E-06	5.5523E+00	3.3261E+00
CCNA_01928	6.0830E-05	1.3650E-04	-1.1661E+00	-9.5911E-01
CCNA_01929	7.5193E-05	6.2468E-05	2.6738E-01	-4.4805E-02
CCNA_01930	5.0976E-05	9.5384E-05	-9.0391E-01	-7.9191E-01
CCNA_01931	5.4643E-05	5.8241E-05	-9.2085E-02	-2.7409E-01
CCNA_01932	4.7943E-05	5.1976E-05	-1.1663E-01	-2.8974E-01
CCNA_01933	6.2222E-05	6.8816E-05	-1.4537E-01	-3.0808E-01
CCNA_01934	7.8328E-05	9.3561E-05	-2.5642E-01	-3.7891E-01
CCNA_01935	8.0460E-05	1.7303E-05	2.2166E+00	1.1985E+00
CCNA_01936	5.7533E-05	9.4738E-05	-7.1955E-01	-6.7431E-01
CCNA_01937	4.7656E-05	3.9165E-05	2.8295E-01	-3.4875E-02
CCNA_01938	5.5096E-05	0.0000E+00	1.2699E+01	7.8846E+00
CCNA_01939	1.1597E-04	1.6077E-04	-4.7122E-01	-5.1591E-01
CCNA_01940	5.7066E-05	1.0903E-04	-9.3404E-01	-8.1112E-01
CCNA_01941	3.9633E-05	8.8340E-05	-1.1563E+00	-9.5290E-01
CCNA_01942	1.2468E-04	1.6243E-04	-3.8163E-01	-4.5877E-01
CCNA_01943	1.2700E-04	1.6243E-04	-3.5496E-01	-4.4176E-01
CCNA_01944	7.8231E-05	1.2853E-05	2.6048E+00	1.4461E+00
CCNA_01945	3.1333E-04	1.6363E-04	9.3724E-01	3.8246E-01
CCNA_01946	3.2121E-04	1.6363E-04	9.7305E-01	4.0530E-01
CCNA_01947	1.5882E-04	4.3350E-05	1.8731E+00	9.7937E-01
CCNA_01948	3.3734E-04	1.7898E-04	9.1439E-01	3.6788E-01
CCNA_01949	5.7986E-05	4.3557E-05	4.1266E-01	4.7861E-02
CCNA_01950	6.3905E-05	1.6116E-04	-1.3344E+00	-1.0665E+00
CCNA_01951	1.7476E-04	1.4798E-04	2.3991E-01	-6.2329E-02
CCNA_01952	3.8623E-04	1.6243E-04	1.2496E+00	5.8172E-01
CCNA_01953	9.8979E-05	1.1681E-04	-2.3905E-01	-3.6783E-01
CCNA_01954	1.8062E-04	5.2863E-05	1.7725E+00	9.1520E-01
CCNA_01955	1.5866E-04	1.0621E-04	5.7902E-01	1.5397E-01
CCNA_01956	6.1209E-05	6.4647E-05	-7.8910E-02	-2.6568E-01
CCNA_01957	2.8564E-04	1.5556E-04	8.7669E-01	3.4384E-01
CCNA_01958	3.4198E-04	1.2319E-04	1.4730E+00	7.2417E-01
CCNA_01959	2.6095E-04	1.0004E-04	1.3831E+00	6.6685E-01

CCNA_01960	1.5446E-04	5.0302E-05	1.6183E+00	8.1689E-01
CCNA_01961	4.5409E-05	1.3019E-04	-1.5195E+00	-1.1846E+00
CCNA_01962	7.7278E-05	4.2148E-05	8.7440E-01	3.4238E-01
CCNA_01963	9.7070E-05	1.0026E-04	-4.6659E-02	-2.4511E-01
CCNA_01964	6.8424E-04	3.2275E-04	1.0841E+00	4.7612E-01
CCNA_01965	6.5872E-04	2.5484E-04	1.3700E+00	6.5850E-01
CCNA_01966	6.9686E-04	3.1152E-04	1.1615E+00	5.2552E-01
CCNA_01967	7.6571E-05	7.9506E-05	-5.4332E-02	-2.5001E-01
CCNA_01968	1.1578E-04	1.6030E-04	-4.6941E-01	-5.1476E-01
CCNA_01969	9.5183E-05	1.5469E-04	-7.0058E-01	-6.6221E-01
CCNA_01970	1.9546E-04	1.3525E-04	5.3117E-01	1.2345E-01
CCNA_01971	1.8169E-04	1.3422E-04	4.3687E-01	6.3300E-02
CCNA_01972	4.3541E-05	1.2591E-04	-1.5318E+00	-1.1924E+00
CCNA_01973	1.8112E-04	5.6302E-05	1.6855E+00	8.5975E-01
CCNA_01974	2.0136E-04	8.4346E-05	1.2553E+00	5.8531E-01
CCNA_01975	1.8377E-04	1.6277E-04	1.7497E-01	-1.0375E-01
CCNA_01976	3.9259E-05	7.3954E-05	-9.1360E-01	-7.9808E-01
CCNA_01977	1.1902E-04	8.9310E-05	4.1430E-01	4.8906E-02
CCNA_01978	2.1867E-04	1.2881E-04	7.6353E-01	2.7166E-01
CCNA_01979	1.1335E-04	2.8358E-05	1.9985E+00	1.0594E+00
CCNA_01980	5.0949E-05	4.4874E-05	1.8301E-01	-9.8618E-02
CCNA_01981	5.8158E-05	7.1965E-05	-3.0737E-01	-4.1141E-01
CCNA_01982	5.6793E-05	9.0072E-05	-6.6537E-01	-6.3975E-01
CCNA_01983	1.5259E-04	1.2133E-04	3.3063E-01	-4.4619E-03
CCNA_01984	4.4452E-05	4.1261E-05	1.0731E-01	-1.4690E-01
CCNA_01985	1.0678E-04	2.8433E-05	1.9087E+00	1.0021E+00
CCNA_01986	4.1099E-05	1.4018E-04	-1.7701E+00	-1.3444E+00
CCNA_01987	6.6647E-05	1.3471E-04	-1.0152E+00	-8.6288E-01
CCNA_01988	6.5413E-05	9.6610E-05	-5.6263E-01	-5.7422E-01
CCNA_01989	9.8961E-05	1.1098E-04	-1.6541E-01	-3.2086E-01
CCNA_01990	1.4155E-04	1.1911E-04	2.4896E-01	-5.6557E-02
CCNA_01991	6.3230E-05	1.3387E-04	-1.0821E+00	-9.0557E-01
CCNA_01992	9.7620E-05	1.3781E-04	-4.9749E-01	-5.3267E-01
CCNA_01993	1.6353E-04	1.3191E-04	3.0993E-01	-1.7663E-02
CCNA_01994	8.9477E-05	6.8700E-05	3.8111E-01	2.7737E-02
CCNA_01995	5.6030E-05	1.8173E-05	1.6238E+00	8.2040E-01
CCNA_01996	1.6999E-04	7.8072E-05	1.1225E+00	5.0061E-01
CCNA_01997	6.6698E-05	1.3351E-04	-1.0012E+00	-8.5399E-01
CCNA_01998	3.5029E-04	9.7911E-05	1.8389E+00	9.5758E-01
CCNA_01999	5.6941E-05	4.8214E-05	2.3989E-01	-6.2337E-02
CCNA_02000	3.8621E-04	6.4995E-04	-7.5095E-01	-6.9434E-01
CCNA_02001	4.0594E-04	6.3935E-04	-6.5534E-01	-6.3336E-01
CCNA_02002	6.6231E-05	9.5599E-05	-5.2951E-01	-5.5310E-01
CCNA_02003	6.0715E-04	9.6478E-05	2.6537E+00	1.4773E+00
CCNA_02004	3.9447E-04	1.4589E-04	1.4349E+00	6.9990E-01
CCNA_02005	5.0134E-04	1.1941E-04	2.0698E+00	1.1049E+00
CCNA_02006	6.9325E-05	1.0784E-04	-6.3746E-01	-6.2195E-01
CCNA_02007	7.0411E-05	1.3754E-04	-9.6596E-01	-8.3148E-01
CCNA_02008	9.1447E-05	1.5550E-04	-7.6590E-01	-7.0387E-01
CCNA_02009	7.0883E-05	4.8894E-06	3.8554E+00	2.2438E+00
CCNA_02010	1.4812E-04	1.0791E-04	4.5697E-01	7.6125E-02
CCNA_02011	3.2524E-04	6.8658E-05	2.2439E+00	1.2159E+00
CCNA_02012	1.0822E-04	1.0308E-04	7.0151E-02	-1.7061E-01

CCNA_02013	9.0240E-05	1.2739E-04	-4.9742E-01	-5.3263E-01
CCNA_02014	1.2263E-04	3.1383E-05	1.9659E+00	1.0386E+00
CCNA_02015	5.5642E-05	6.1390E-05	-1.4191E-01	-3.0587E-01
CCNA_02016	5.9452E-05	1.1892E-04	-1.0002E+00	-8.5330E-01
CCNA_02017	7.1443E-05	4.7899E-05	5.7663E-01	1.5245E-01
CCNA_02018	5.6655E-05	1.2697E-04	-1.1642E+00	-9.5795E-01
CCNA_02019	1.0583E-04	9.6354E-05	1.3531E-01	-1.2905E-01
CCNA_02020	5.5832E-05	1.2464E-05	2.1625E+00	1.1640E+00
CCNA_02021	5.2109E-05	1.1606E-04	-1.1552E+00	-9.5221E-01
CCNA_02022	5.1772E-05	1.2034E-04	-1.2168E+00	-9.9148E-01
CCNA_02023	7.4286E-05	7.2114E-05	4.2746E-02	-1.8809E-01
CCNA_02024	8.9329E-05	4.4410E-05	1.0080E+00	4.2762E-01
CCNA_02025	6.6814E-05	6.5700E-05	2.4179E-02	-1.9993E-01
CCNA_02026	5.9794E-05	1.3147E-04	-1.1366E+00	-9.4031E-01
CCNA_02027	6.7850E-05	1.1038E-04	-7.0201E-01	-6.6312E-01
CCNA_02028	7.6478E-05	8.6492E-05	-1.7757E-01	-3.2861E-01
CCNA_02029	1.0801E-04	4.1593E-05	1.3765E+00	6.6266E-01
CCNA_02030	2.3360E-04	1.2163E-04	9.4146E-01	3.8515E-01
CCNA_02031	1.3211E-04	1.5972E-04	-2.7383E-01	-3.9001E-01
CCNA_02032	1.7583E-04	1.4198E-04	3.0844E-01	-1.8613E-02
CCNA_02033	5.4246E-04	4.6108E-04	2.3448E-01	-6.5793E-02
CCNA_02034	3.5969E-04	2.0195E-04	8.3274E-01	3.1581E-01
CCNA_02035	1.4968E-04	8.3003E-05	8.5057E-01	3.2718E-01
CCNA_02036	7.6409E-05	1.3506E-04	-8.2181E-01	-7.3954E-01
CCNA_02037	3.0612E-04	6.7448E-05	2.1821E+00	1.1765E+00
CCNA_02038	2.6458E-04	1.3788E-04	9.4025E-01	3.8438E-01
CCNA_02039	4.3715E-04	9.7406E-05	2.1659E+00	1.1662E+00
CCNA_02040	4.0252E-04	1.2789E-04	1.6540E+00	8.3965E-01
CCNA_02041	4.9976E-04	1.6242E-04	1.6215E+00	8.1889E-01
CCNA_02042	1.7002E-04	1.6649E-04	3.0222E-02	-1.9607E-01
CCNA_02043	6.8340E-05	1.1188E-04	-7.1121E-01	-6.6899E-01
CCNA_02044	8.1223E-05	1.8488E-05	2.1347E+00	1.1462E+00
CCNA_02045	1.1861E-04	3.0786E-05	1.9455E+00	1.0256E+00
CCNA_02046	1.1742E-04	3.8891E-05	1.5939E+00	8.0128E-01
CCNA_02047	1.2238E-04	1.2606E-04	-4.2790E-02	-2.4264E-01
CCNA_02048	1.0952E-04	1.3265E-04	-2.7650E-01	-3.9171E-01
CCNA_02049	1.2098E-04	8.6799E-05	4.7895E-01	9.0145E-02
CCNA_02050	1.1609E-04	7.0805E-05	7.1326E-01	2.3959E-01
CCNA_02051	5.2488E-05	4.6789E-05	1.6571E-01	-1.0965E-01
CCNA_02052	2.0796E-04	7.0390E-05	1.5627E+00	7.8142E-01
CCNA_02053	2.8947E-05	9.4174E-05	-1.7018E+00	-1.3008E+00
CCNA_02054	1.0863E-04	1.6243E-04	-5.8038E-01	-5.8554E-01
CCNA_02055	4.6371E-05	4.3565E-05	8.9912E-02	-1.5800E-01
CCNA_02056	5.0981E-05	6.7954E-06	2.9057E+00	1.6380E+00
CCNA_02057	4.8724E-05	6.1490E-05	-3.3576E-01	-4.2952E-01
CCNA_02058	3.7465E-05	1.2831E-04	-1.7759E+00	-1.3481E+00
CCNA_02059	5.9055E-05	1.5963E-04	-1.4346E+00	-1.1304E+00
CCNA_02060	7.3861E-05	8.7594E-06	3.0746E+00	1.7458E+00
CCNA_02061	7.6367E-05	2.7646E-05	1.4656E+00	7.1944E-01
CCNA_02062	1.6894E-04	1.0985E-04	6.2088E-01	1.8067E-01
CCNA_02063	1.2816E-04	1.6275E-04	-3.4470E-01	-4.3522E-01
CCNA_02064	1.7648E-04	6.5940E-05	1.4202E+00	6.9049E-01
CCNA_02065	1.5574E-04	8.5249E-05	8.6925E-01	3.3909E-01

CCNA_02066	1.1971E-04	3.4731E-05	1.7849E+00	9.2316E-01
CCNA_02067	8.4066E-05	2.8002E-05	1.5857E+00	7.9605E-01
CCNA_02068	7.0846E-05	1.2208E-04	-7.8513E-01	-7.1614E-01
CCNA_02069	7.9757E-05	9.6610E-05	-2.7661E-01	-3.9179E-01
CCNA_02070	8.7974E-05	5.9658E-05	5.6022E-01	1.4198E-01
CCNA_02071	1.0777E-04	1.6151E-04	-5.8371E-01	-5.8767E-01
CCNA_02072	1.1586E-04	1.3806E-04	-2.5294E-01	-3.7669E-01
CCNA_02073	3.5569E-05	1.0962E-04	-1.6238E+00	-1.2511E+00
CCNA_02074	5.3131E-05	6.1216E-05	-2.0443E-01	-3.4575E-01
CCNA_02075	2.0266E-04	1.5517E-04	3.8521E-01	3.0350E-02
CCNA_02076	6.9598E-05	1.0989E-04	-6.5891E-01	-6.3563E-01
CCNA_02077	9.5331E-05	1.4076E-04	-5.6219E-01	-5.7394E-01
CCNA_02078	2.2962E-04	1.6271E-04	4.9692E-01	1.0160E-01
CCNA_02079	5.7404E-05	2.7223E-05	1.0760E+00	4.7097E-01
CCNA_02080	6.9954E-05	1.3923E-04	-9.9300E-01	-8.4873E-01
CCNA_02081	1.5048E-04	1.6267E-04	-1.1239E-01	-2.8704E-01
CCNA_02082	2.6628E-04	2.1755E-04	2.9153E-01	-2.9401E-02
CCNA_02083	5.2419E-05	1.2728E-04	-1.2798E+00	-1.0317E+00
CCNA_02084	6.7249E-05	9.9851E-05	-5.7029E-01	-5.7911E-01
CCNA_02085	7.0925E-05	1.3516E-05	2.3908E+00	1.3096E+00
CCNA_02086	4.8353E-04	7.6249E-05	2.6647E+00	1.4843E+00
CCNA_02087	2.2084E-04	1.4015E-04	6.5599E-01	2.0307E-01
CCNA_02088	2.1030E-04	1.6129E-04	3.8271E-01	2.8759E-02
CCNA_02089	3.9916E-04	1.1354E-04	1.8137E+00	9.4148E-01
CCNA_02090	3.2154E-04	1.6243E-04	9.8517E-01	4.1303E-01
CCNA_02091	3.4232E-04	6.8120E-05	2.3290E+00	1.2702E+00
CCNA_02092	1.2141E-04	6.9885E-05	7.9673E-01	2.9284E-01
CCNA_02093	1.0963E-04	1.0386E-04	7.7969E-02	-1.6562E-01
CCNA_02094	2.6011E-04	1.6243E-04	6.7927E-01	2.1792E-01
CCNA_02095	2.9980E-04	1.5902E-04	9.1472E-01	3.6809E-01
CCNA_02096	4.8304E-04	1.4531E-04	1.7330E+00	8.9002E-01
CCNA_02097	4.1026E-04	1.0500E-04	1.9661E+00	1.0387E+00
CCNA_02098	1.0167E-04	1.7506E-04	-7.8398E-01	-7.1541E-01
CCNA_02099	2.2864E-04	8.9011E-05	1.3609E+00	6.5269E-01
CCNA_02100	1.3235E-04	2.8565E-05	2.2117E+00	1.1953E+00
CCNA_02101	1.6610E-04	3.1971E-05	2.3769E+00	1.3007E+00
CCNA_02102	9.0938E-05	1.5448E-04	-7.6446E-01	-7.0296E-01
CCNA_02103	6.9316E-05	1.0360E-04	-5.7986E-01	-5.8521E-01
CCNA_02104	6.2416E-05	3.6098E-05	7.8977E-01	2.8839E-01
CCNA_02105	1.3288E-04	9.6925E-05	4.5514E-01	7.4957E-02
CCNA_02106	1.0155E-04	6.8401E-05	5.6992E-01	1.4817E-01
CCNA_02107	1.6182E-04	5.3451E-05	1.5979E+00	8.0384E-01
CCNA_02108	6.8576E-05	8.7014E-07	6.2867E+00	3.7946E+00
CCNA_02109	2.4834E-04	1.5695E-04	6.6197E-01	2.0688E-01
CCNA_02110	1.4416E-04	1.0039E-04	5.2201E-01	1.1761E-01
CCNA_02111	2.5052E-04	1.5978E-04	6.4877E-01	1.9846E-01
CCNA_02112	2.4749E-04	1.6194E-04	6.1187E-01	1.7493E-01
CCNA_02113	6.0923E-05	6.0620E-05	7.1058E-03	-2.1082E-01
CCNA_02114	9.8457E-05	2.8425E-05	1.7920E+00	9.2766E-01
CCNA_02115	7.0781E-05	1.3331E-04	-9.1338E-01	-7.9794E-01
CCNA_02116	6.2805E-05	9.3486E-05	-5.7390E-01	-5.8141E-01
CCNA_02117	5.4255E-05	1.0514E-04	-9.5445E-01	-8.2414E-01
CCNA_02118	4.2070E-05	1.0988E-04	-1.3850E+00	-1.0988E+00

CCNA_02119	9.1618E-05	1.6103E-04	-8.1367E-01	-7.3434E-01
CCNA_02120	8.9111E-05	6.9901E-05	3.5020E-01	8.0179E-03
CCNA_02121	9.3546E-05	1.0120E-04	-1.1353E-01	-2.8777E-01
CCNA_02122	4.9173E-05	6.3810E-06	2.9443E+00	1.6626E+00
CCNA_02123	0.0000E+00	0.0000E+00	-8.4168E-01	-7.5221E-01
CCNA_02124	9.1188E-05	9.4456E-05	-5.0854E-02	-2.4779E-01
CCNA_02125	1.2799E-03	2.1170E-04	2.5959E+00	1.4404E+00
CCNA_02126	1.2684E-03	2.1003E-04	2.5944E+00	1.4394E+00
CCNA_02127	2.7697E-04	6.0786E-05	2.1877E+00	1.1801E+00
CCNA_02128	2.8275E-04	6.1241E-05	2.2068E+00	1.1922E+00
CCNA_02129	4.8285E-05	1.1362E-04	-1.2345E+00	-1.0028E+00
CCNA_02130	2.0963E-04	1.0441E-04	1.0055E+00	4.2599E-01
CCNA_02131	6.3743E-05	4.5803E-05	4.7669E-01	8.8704E-02
CCNA_02132	2.6138E-04	2.3983E-05	3.4456E+00	1.9824E+00
CCNA_02133	4.5670E-04	9.0809E-05	2.3302E+00	1.2710E+00
CCNA_02134	6.1237E-05	1.3031E-04	-1.0894E+00	-9.1022E-01
CCNA_02135	5.4019E-05	7.3556E-05	-4.4541E-01	-4.9945E-01
CCNA_02136	6.1751E-04	2.4288E-04	1.3462E+00	6.4332E-01
CCNA_02137	3.4237E-05	3.4806E-07	6.5863E+00	3.9857E+00
CCNA_02138	5.7649E-05	4.6457E-05	3.1125E-01	-1.6824E-02
CCNA_02139	5.8796E-05	1.5820E-04	-1.4279E+00	-1.1261E+00
CCNA_02140	1.2172E-04	2.8300E-05	2.1043E+00	1.1268E+00
CCNA_02141	1.2250E-04	1.2000E-04	2.9616E-02	-1.9646E-01
CCNA_02142	1.2271E-04	1.0976E-04	1.6088E-01	-1.1274E-01
CCNA_02143	1.0401E-04	8.6218E-05	2.7058E-01	-4.2762E-02
CCNA_02144	5.2308E-05	3.3471E-05	6.4387E-01	1.9534E-01
CCNA_02145	4.7906E-05	9.4406E-05	-9.7866E-01	-8.3958E-01
CCNA_02146	4.6024E-05	1.2441E-04	-1.4346E+00	-1.1304E+00
CCNA_02147	8.2952E-05	9.4837E-05	-1.9322E-01	-3.3859E-01
CCNA_02148	8.7142E-05	6.1250E-05	5.0854E-01	1.0902E-01
CCNA_02149	7.0213E-05	7.3937E-05	-7.4634E-02	-2.6296E-01
CCNA_02150	1.0440E-04	5.1057E-05	1.0318E+00	4.4279E-01
CCNA_02151	1.4554E-04	1.6243E-04	-1.5840E-01	-3.1639E-01
CCNA_02152	1.5668E-04	1.1742E-04	4.1613E-01	5.0072E-02
CCNA_02153	1.1258E-04	3.1400E-05	1.8418E+00	9.5942E-01
CCNA_02154	1.2526E-04	1.0752E-04	2.2018E-01	-7.4910E-02
CCNA_02155	8.2624E-05	1.1894E-04	-5.2557E-01	-5.5058E-01
CCNA_02156	6.2689E-05	1.4363E-04	-1.1961E+00	-9.7825E-01
CCNA_02157	5.4241E-05	1.7237E-05	1.6533E+00	8.3919E-01
CCNA_02158	5.6040E-05	3.6156E-05	6.3199E-01	1.8776E-01
CCNA_02159	7.4938E-05	1.7138E-05	2.1279E+00	1.1419E+00
CCNA_02160	2.2841E-04	1.6299E-04	4.8679E-01	9.5144E-02
CCNA_02161	2.0713E-04	1.5237E-04	4.4294E-01	6.7174E-02
CCNA_02162	4.9445E-04	9.9221E-05	2.3170E+00	1.2625E+00
CCNA_02163	5.5481E-04	1.0339E-04	2.4238E+00	1.3307E+00
CCNA_02164	1.4238E-04	1.6165E-04	-1.8316E-01	-3.3218E-01
CCNA_02165	7.0855E-05	6.8385E-05	5.1123E-02	-1.8274E-01
CCNA_02166	6.3864E-05	1.3708E-04	-1.1020E+00	-9.1824E-01
CCNA_02167	9.3514E-05	1.2116E-04	-3.7365E-01	-4.5368E-01
CCNA_04007	1.1743E-04	9.6569E-05	2.8210E-01	-3.5419E-02
CCNA_02169	1.2936E-04	1.1695E-04	1.4549E-01	-1.2255E-01
CCNA_02170	8.0622E-05	1.3950E-04	-7.9107E-01	-7.1993E-01
CCNA_02171	2.1870E-04	8.7304E-05	1.3247E+00	6.2962E-01

CCNA_02172	2.1597E-04	4.8960E-05	2.1409E+00	1.1502E+00
CCNA_02173	6.0641E-05	1.5107E-05	2.0044E+00	1.0631E+00
CCNA_02174	5.9628E-05	2.6411E-05	1.1745E+00	5.3381E-01
CCNA_02175	3.6396E-05	6.5070E-05	-8.3819E-01	-7.4999E-01
CCNA_02176	5.6141E-05	1.4895E-04	-1.4077E+00	-1.1132E+00
CCNA_02177	3.6110E-05	3.3571E-05	1.0501E-01	-1.4837E-01
CCNA_02178	4.9871E-05	7.2039E-05	-5.3061E-01	-5.5380E-01
CCNA_02179	4.4179E-05	9.0395E-05	-1.0329E+00	-8.7416E-01
CCNA_02180	8.1380E-05	3.9173E-05	1.0546E+00	4.5731E-01
CCNA_02181	6.3702E-05	5.7015E-06	3.4799E+00	2.0043E+00
CCNA_02182	1.1000E-04	1.0846E-04	2.0321E-02	-2.0239E-01
CCNA_02183	1.1903E-04	7.4327E-05	6.7932E-01	2.1795E-01
CCNA_02184	5.4666E-05	2.5441E-05	1.1031E+00	4.8827E-01
CCNA_02185	7.9142E-05	6.8028E-05	2.1821E-01	-7.6167E-02
CCNA_02186	1.2218E-04	9.6784E-05	3.3607E-01	-9.8924E-04
CCNA_02187	3.1797E-04	7.3746E-05	2.1081E+00	1.1293E+00
CCNA_02188	6.2925E-05	8.3442E-05	-4.0718E-01	-4.7507E-01
CCNA_02189	8.2106E-05	9.7157E-05	-2.4288E-01	-3.7027E-01
CCNA_02190	9.2672E-05	1.1695E-04	-3.3568E-01	-4.2946E-01
CCNA_02191	5.4213E-05	1.0806E-05	2.3258E+00	1.2681E+00
CCNA_02192	8.8728E-05	7.1401E-05	3.1334E-01	-1.5489E-02
CCNA_02193	1.4223E-04	1.6097E-04	-1.7859E-01	-3.2926E-01
CCNA_02194	1.7845E-04	1.6625E-04	1.0219E-01	-1.5017E-01
CCNA_02195	6.2717E-05	2.8483E-05	1.1385E+00	5.1081E-01
CCNA_02196	8.5172E-05	1.1393E-04	-4.1973E-01	-4.8308E-01
CCNA_02197	4.7314E-05	4.1344E-05	1.9444E-01	-9.1331E-02
CCNA_02198	1.5074E-04	1.6243E-04	-1.0778E-01	-2.8410E-01
CCNA_02199	1.4073E-04	1.6243E-04	-2.0692E-01	-3.4734E-01
CCNA_02200	9.1974E-04	1.6243E-04	2.5014E+00	1.3801E+00
CCNA_02201	5.7728E-05	1.4559E-04	-1.3345E+00	-1.0666E+00
CCNA_02202	7.6862E-05	1.0034E-04	-3.8458E-01	-4.6065E-01
CCNA_02203	5.4962E-05	4.7684E-05	2.0482E-01	-8.4711E-02
CCNA_02204	1.0164E-04	1.4498E-04	-5.1238E-01	-5.4217E-01
CCNA_02205	1.7467E-04	7.0829E-05	1.3021E+00	6.1518E-01
CCNA_02206	1.3026E-04	9.7953E-06	3.7320E+00	2.1651E+00
CCNA_02207	2.8752E-04	4.6184E-05	2.6380E+00	1.4673E+00
CCNA_02208	1.9487E-04	1.4414E-04	4.3497E-01	6.2094E-02
CCNA_02209	1.9108E-04	1.2033E-04	6.6716E-01	2.1019E-01
CCNA_02210	1.5806E-04	7.2487E-05	1.1246E+00	5.0195E-01
CCNA_02211	6.4821E-05	2.2102E-05	1.5519E+00	7.7450E-01
CCNA_02212	9.7268E-05	1.2716E-04	-3.8659E-01	-4.6193E-01
CCNA_02213	5.7510E-05	1.2551E-04	-1.1259E+00	-9.3347E-01
CCNA_02214	5.7529E-05	1.0225E-04	-8.2969E-01	-7.4456E-01
CCNA_02215	5.6067E-05	1.0806E-04	-9.4663E-01	-8.1915E-01
CCNA_02216	7.1151E-05	3.6140E-05	9.7706E-01	4.0786E-01
CCNA_02217	1.4353E-04	1.1806E-04	2.8179E-01	-3.5614E-02
CCNA_02218	1.4146E-04	7.1285E-05	9.8856E-01	4.1520E-01
CCNA_02219	1.5803E-04	8.4470E-05	9.0363E-01	3.6102E-01
CCNA_02220	4.1478E-05	5.3103E-05	-3.5651E-01	-4.4275E-01
CCNA_02221	3.4981E-05	5.4703E-05	-6.4505E-01	-6.2679E-01
CCNA_02222	6.2703E-05	1.2943E-04	-1.0455E+00	-8.8223E-01
CCNA_02223	4.7203E-05	1.2020E-04	-1.3485E+00	-1.0755E+00
CCNA_02224	4.8710E-05	1.3517E-04	-1.4724E+00	-1.1545E+00

CCNA_02225	2.4516E-04	6.4614E-05	1.9237E+00	1.0116E+00
CCNA_02226	1.4420E-04	1.4434E-04	-1.4064E-03	-2.1625E-01
CCNA_02227	7.4837E-05	7.6034E-05	-2.2960E-02	-2.3000E-01
CCNA_02228	7.3986E-05	1.0568E-04	-5.1435E-01	-5.4343E-01
CCNA_02229	4.8401E-05	5.5888E-05	-2.0758E-01	-3.4776E-01
CCNA_02230	6.2601E-05	1.3326E-05	2.2312E+00	1.2078E+00
CCNA_02231	8.8704E-05	1.6243E-04	-8.7270E-01	-7.7200E-01
CCNA_02232	8.2910E-05	1.6003E-04	-9.4872E-01	-8.2049E-01
CCNA_02233	5.0777E-05	4.9109E-05	4.8087E-02	-1.8468E-01
CCNA_02234	3.6331E-04	1.6275E-04	1.1585E+00	5.2358E-01
CCNA_02235	3.5026E-04	1.6244E-04	1.1085E+00	4.9167E-01
CCNA_02236	7.7773E-05	8.8398E-05	-1.8479E-01	-3.3322E-01
CCNA_02237	6.1126E-05	1.5726E-04	-1.3633E+00	-1.0849E+00
CCNA_02238	6.5718E-05	1.4040E-04	-1.0952E+00	-9.1389E-01
CCNA_02239	5.9457E-05	1.3191E-04	-1.1497E+00	-9.4865E-01
CCNA_02240	1.6707E-04	1.3055E-04	3.5579E-01	1.1585E-02
CCNA_02241	9.3000E-05	1.0725E-04	-2.0572E-01	-3.4657E-01
CCNA_02242	3.7839E-04	2.3587E-04	6.8183E-01	2.1955E-01
CCNA_02243	4.0876E-04	2.7840E-04	5.5410E-01	1.3808E-01
CCNA_02244	2.1127E-04	1.6274E-04	3.7645E-01	2.4761E-02
CCNA_02245	8.7373E-05	1.4870E-04	-7.6718E-01	-7.0469E-01
CCNA_02246	5.5984E-04	1.0866E-04	2.3651E+00	1.2932E+00
CCNA_02247	5.6171E-04	1.0869E-04	2.3695E+00	1.2960E+00
CCNA_02248	6.3956E-05	1.3329E-04	-1.0594E+00	-8.9107E-01
CCNA_02249	9.1322E-05	7.2429E-05	3.3430E-01	-2.1192E-03
CCNA_02250	5.7829E-05	4.1593E-05	4.7530E-01	8.7813E-02
CCNA_02251	2.5842E-04	8.2614E-05	1.6452E+00	8.3401E-01
CCNA_02252	2.8488E-04	1.6272E-04	8.0796E-01	3.0000E-01
CCNA_02253	2.5835E-04	1.6274E-04	6.6672E-01	2.0991E-01
CCNA_02254	9.8748E-05	4.8670E-05	1.0205E+00	4.3560E-01
CCNA_02255	7.5868E-05	2.8789E-05	1.3976E+00	6.7612E-01
CCNA_02256	3.3904E-04	2.1273E-04	6.7239E-01	2.1353E-01
CCNA_02257	8.8039E-05	7.3340E-05	2.6344E-01	-4.7318E-02
CCNA_02258	1.4200E-04	1.6243E-04	-1.9395E-01	-3.3906E-01
CCNA_02259	4.8808E-05	1.1062E-04	-1.1805E+00	-9.6830E-01
CCNA_02260	3.3341E-04	4.3432E-05	2.9402E+00	1.6600E+00
CCNA_02261	9.8697E-05	1.0444E-04	-8.1663E-02	-2.6744E-01
CCNA_02262	9.8864E-05	1.6238E-04	-7.1591E-01	-6.7199E-01
CCNA_02263	7.2497E-05	1.1526E-04	-6.6897E-01	-6.4205E-01
CCNA_02264	1.0398E-04	1.5857E-04	-6.0888E-01	-6.0372E-01
CCNA_02265	9.7652E-05	1.1408E-04	-2.2435E-01	-3.5845E-01
CCNA_02266	9.7943E-05	1.0193E-06	6.5747E+00	3.9783E+00
CCNA_02267	4.3060E-05	9.1000E-05	-1.0795E+00	-9.0390E-01
CCNA_02268	1.4655E-04	1.1116E-04	3.9868E-01	3.8945E-02
CCNA_02269	2.8446E-04	2.3228E-04	2.9236E-01	-2.8874E-02
CCNA_02270	2.6371E-04	1.2710E-04	1.0530E+00	4.5627E-01
CCNA_02271	3.0387E-04	4.8753E-05	2.6397E+00	1.4683E+00
CCNA_02272	2.5125E-04	1.0215E-04	1.2983E+00	6.1275E-01
CCNA_02273	1.2263E-04	1.1058E-04	1.4910E-01	-1.2025E-01
CCNA_02274	7.2423E-05	1.3113E-04	-8.5652E-01	-7.6168E-01
CCNA_02275	7.6548E-05	1.6289E-04	-1.0895E+00	-9.1025E-01
CCNA_02276	6.9366E-05	3.4184E-05	1.0207E+00	4.3567E-01
CCNA_02277	7.5886E-05	1.6302E-04	-1.1031E+00	-9.1898E-01

CCNA_02278	6.5232E-05	1.3757E-04	-1.0764E+00	-9.0195E-01
CCNA_02279	1.2671E-04	5.9136E-05	1.0992E+00	4.8577E-01
CCNA_02280	5.7455E-05	1.4212E-05	2.0146E+00	1.0696E+00
CCNA_02281	1.1788E-04	1.0011E-04	2.3568E-01	-6.5024E-02
CCNA_02282	6.5533E-05	8.4395E-05	-3.6498E-01	-4.4815E-01
CCNA_02283	4.2943E-04	4.8662E-05	3.1413E+00	1.7883E+00
CCNA_02284	6.4456E-05	8.7462E-05	-4.4038E-01	-4.9624E-01
CCNA_02285	2.5575E-04	1.6243E-04	6.5488E-01	2.0236E-01
CCNA_02286	6.3386E-04	2.3253E-04	1.4467E+00	7.0739E-01
CCNA_02287	6.2665E-04	2.4165E-04	1.3747E+00	6.6149E-01
CCNA_02288	6.9144E-05	4.6275E-05	5.7922E-01	1.5410E-01
CCNA_02289	4.7434E-05	8.8688E-05	-9.0280E-01	-7.9120E-01
CCNA_02290	1.1677E-04	1.5673E-04	-4.2462E-01	-4.8619E-01
CCNA_02291	6.2772E-05	1.1900E-05	2.3982E+00	1.3144E+00
CCNA_02292	6.6037E-05	5.7661E-05	1.9557E-01	-9.0611E-02
CCNA_02293	7.5997E-05	1.1135E-04	-5.5114E-01	-5.6689E-01
CCNA_02294	7.1415E-05	1.2591E-04	-8.1814E-01	-7.3719E-01
CCNA_02295	5.8319E-05	3.1698E-05	8.7932E-01	3.4552E-01
CCNA_02296	5.3450E-05	9.4630E-05	-8.2410E-01	-7.4100E-01
CCNA_02297	6.5024E-05	3.3985E-05	9.3583E-01	3.8156E-01
CCNA_02298	4.1847E-04	1.6237E-04	1.3658E+00	6.5582E-01
CCNA_02299	6.0948E-04	1.8305E-04	1.7353E+00	8.9147E-01
CCNA_02300	6.9200E-05	1.5209E-04	-1.1361E+00	-9.3999E-01
CCNA_02301	1.2764E-04	1.5008E-04	-2.3367E-01	-3.6440E-01
CCNA_02302	1.2972E-04	8.1296E-05	6.7400E-01	2.1456E-01
CCNA_02303	9.0226E-05	6.5650E-05	4.5864E-01	7.7187E-02
CCNA_02304	1.0295E-04	1.3110E-05	2.9723E+00	1.6805E+00
CCNA_02305	1.7456E-04	1.0757E-04	6.9832E-01	2.3007E-01
CCNA_02306	6.0400E-05	9.6876E-05	-6.8159E-01	-6.5010E-01
CCNA_02307	5.7140E-05	3.9049E-05	5.4905E-01	1.3485E-01
CCNA_02308	1.1305E-04	1.5788E-04	-4.8191E-01	-5.2273E-01
CCNA_02309	1.1088E-04	1.6082E-04	-5.3643E-01	-5.5751E-01
CCNA_02310	5.5545E-05	2.1745E-05	1.3525E+00	6.4735E-01
CCNA_04009	2.6596E-04	1.8833E-04	4.9788E-01	1.0221E-01
CCNA_02312	6.5186E-05	1.3839E-04	-1.0861E+00	-9.0813E-01
CCNA_02313	7.7158E-05	1.0555E-04	-4.5209E-01	-5.0372E-01
CCNA_02314	5.8796E-05	1.1744E-04	-9.9808E-01	-8.5197E-01
CCNA_02315	6.4733E-05	4.0209E-05	6.8680E-01	2.2272E-01
CCNA_02316	9.7070E-05	1.5735E-04	-6.9694E-01	-6.5989E-01
CCNA_02317	5.0144E-05	0.0000E+00	1.2563E+01	7.7979E+00
CCNA_02318	1.4695E-04	8.6517E-05	7.6413E-01	2.7205E-01
CCNA_02319	1.3481E-04	2.9046E-05	2.2142E+00	1.1969E+00
CCNA_02320	1.4047E-04	1.2204E-04	2.0288E-01	-8.5944E-02
CCNA_02321	1.6577E-04	1.5580E-04	8.9448E-02	-1.5830E-01
CCNA_02322	4.7758E-05	1.1833E-04	-1.3090E+00	-1.0503E+00
CCNA_02323	6.3332E-05	5.9501E-06	3.4100E+00	1.9597E+00
CCNA_02324	1.4968E-04	7.8462E-05	9.3174E-01	3.7895E-01
CCNA_02325	5.2465E-05	7.8967E-05	-5.8992E-01	-5.9163E-01
CCNA_02326	1.0644E-04	1.5835E-04	-5.7304E-01	-5.8086E-01
CCNA_02327	2.5419E-04	1.3992E-04	8.6125E-01	3.3399E-01
CCNA_02328	2.5040E-04	1.3600E-04	8.8056E-01	3.4630E-01
CCNA_02329	9.5460E-05	9.0743E-06	3.3938E+00	1.9494E+00
CCNA_02330	1.2468E-04	8.4528E-05	5.6070E-01	1.4229E-01

CCNA_02331	1.2139E-04	6.0031E-05	1.0157E+00	4.3250E-01
CCNA_02332	1.0923E-04	1.6243E-04	-5.7241E-01	-5.8046E-01
CCNA_02333	1.2263E-04	8.0376E-05	6.0934E-01	1.7331E-01
CCNA_02334	1.1503E-04	1.0720E-04	1.0169E-01	-1.5049E-01
CCNA_02335	1.0696E-04	1.1990E-04	-1.6482E-01	-3.2048E-01
CCNA_02336	8.1038E-05	5.9915E-05	4.3555E-01	6.2461E-02
CCNA_02337	8.5264E-05	1.4734E-04	-7.8910E-01	-7.1867E-01
CCNA_02338	4.0678E-05	1.3021E-04	-1.6784E+00	-1.2859E+00
CCNA_02339	1.0291E-04	1.2225E-04	-2.4849E-01	-3.7385E-01
CCNA_02340	1.0241E-04	5.1098E-05	1.0029E+00	4.2431E-01
CCNA_02341	1.0420E-04	6.1291E-05	7.6548E-01	2.7290E-01
CCNA_02342	1.8368E-04	4.5314E-05	2.0189E+00	1.0724E+00
CCNA_02343	1.1599E-04	1.6286E-04	-4.8966E-01	-5.2768E-01
CCNA_02344	1.4197E-04	9.8624E-05	5.2545E-01	1.1980E-01
CCNA_02345	1.2633E-04	1.2464E-04	1.9377E-02	-2.0299E-01
CCNA_02346	1.6644E-04	7.8304E-05	1.0877E+00	4.7845E-01
CCNA_02347	2.7808E-04	1.1546E-04	1.2681E+00	5.9347E-01
CCNA_02348	6.1908E-05	7.3291E-05	-2.4357E-01	-3.7071E-01
CCNA_02349	1.2591E-04	1.4404E-04	-1.9413E-01	-3.3918E-01
CCNA_02350	7.6090E-05	6.8501E-05	1.5150E-01	-1.1872E-01
CCNA_02351	1.0032E-04	1.5836E-04	-6.5851E-01	-6.3538E-01
CCNA_02352	6.3952E-05	1.5613E-04	-1.2876E+00	-1.0367E+00
CCNA_02353	1.9025E-04	1.0774E-04	8.2027E-01	3.0785E-01
CCNA_02354	2.5585E-04	1.6243E-04	6.5545E-01	2.0273E-01
CCNA_02355	5.3963E-05	1.1293E-04	-1.0653E+00	-8.9486E-01
CCNA_02356	7.6797E-05	8.9019E-05	-2.1311E-01	-3.5128E-01
CCNA_02357	1.7272E-04	1.6035E-04	1.0717E-01	-1.4699E-01
CCNA_02358	5.5494E-05	1.3531E-04	-1.2858E+00	-1.0355E+00
CCNA_02359	4.6204E-05	9.9030E-06	2.2210E+00	1.2013E+00
CCNA_02360	6.3397E-05	2.3461E-05	1.4338E+00	6.9916E-01
CCNA_02361	9.6838E-05	8.1644E-05	2.4615E-01	-5.8345E-02
CCNA_02362	2.2491E-04	1.4140E-04	6.6951E-01	2.1169E-01
CCNA_02363	2.2310E-04	1.4378E-04	6.3380E-01	1.8891E-01
CCNA_02364	2.8108E-04	1.2020E-04	1.2254E+00	5.6628E-01
CCNA_02365	5.1221E-05	7.8710E-05	-6.1983E-01	-6.1070E-01
CCNA_02366	5.3409E-05	7.7476E-05	-5.3670E-01	-5.5768E-01
CCNA_02367	5.7963E-05	1.0373E-04	-8.3961E-01	-7.5089E-01
CCNA_02368	1.3175E-04	1.3046E-04	1.4126E-02	-2.0634E-01
CCNA_02369	7.8860E-05	5.9750E-05	4.0024E-01	3.9941E-02
CCNA_02370	8.1949E-05	7.8412E-05	6.3574E-02	-1.7480E-01
CCNA_02371	9.8660E-05	6.6951E-05	5.5925E-01	1.4136E-01
CCNA_02372	2.1867E-05	0.0000E+00	1.1366E+01	7.0343E+00
CCNA_02373	5.1328E-07	3.9778E-06	-2.9442E+00	-2.0933E+00
CCNA_02374	3.8177E-05	7.6465E-05	-1.0021E+00	-8.5452E-01
CCNA_02375	7.5123E-05	8.2241E-05	-1.3065E-01	-2.9868E-01
CCNA_02376	5.6285E-05	1.1176E-04	-9.8956E-01	-8.4654E-01
CCNA_02377	1.0264E-04	2.6485E-05	1.9539E+00	1.0309E+00
CCNA_02378	1.2186E-04	1.2879E-04	-7.9775E-02	-2.6624E-01
CCNA_02379	7.4707E-05	1.2397E-04	-7.3073E-01	-6.8144E-01
CCNA_02380	9.7130E-05	1.2302E-04	-3.4095E-01	-4.3283E-01
CCNA_02381	1.0457E-04	1.3307E-04	-3.4763E-01	-4.3708E-01
CCNA_02382	2.1799E-04	4.7377E-05	2.2018E+00	1.1891E+00
CCNA_02383	7.2714E-05	1.2095E-04	-7.3410E-01	-6.8359E-01

CCNA_02384	1.3889E-04	2.3353E-05	2.5718E+00	1.4251E+00
CCNA_02385	6.8779E-05	1.9524E-05	1.8162E+00	9.4309E-01
CCNA_02386	1.3984E-04	9.6412E-05	5.3645E-01	1.2682E-01
CCNA_02387	8.2721E-05	1.3727E-04	-7.3075E-01	-6.8146E-01
CCNA_02388	5.0856E-05	1.4482E-04	-1.5097E+00	-1.1783E+00
CCNA_02389	2.1129E-04	1.2593E-04	7.4657E-01	2.6084E-01
CCNA_02390	1.9950E-04	7.2885E-05	1.4526E+00	7.1115E-01
CCNA_02391	1.6060E-04	2.6576E-04	-7.2664E-01	-6.7883E-01
CCNA_02392	7.7958E-05	1.0993E-04	-4.9581E-01	-5.3160E-01
CCNA_04015	2.9407E-04	4.6499E-05	2.6607E+00	1.4817E+00
CCNA_02394	1.3138E-04	9.9967E-05	3.9411E-01	3.6030E-02
CCNA_02395	2.9925E-04	4.5769E-05	2.7087E+00	1.5124E+00
CCNA_02396	1.2065E-04	3.8941E-05	1.6313E+00	8.2513E-01
CCNA_02397	1.5012E-04	7.9241E-05	9.2167E-01	3.7253E-01
CCNA_02398	1.9518E-04	8.5912E-05	1.1838E+00	5.3973E-01
CCNA_02399	7.0194E-05	1.4948E-04	-1.0905E+00	-9.1094E-01
CCNA_02400	5.3618E-04	1.1458E-04	2.2263E+00	1.2047E+00
CCNA_02401	5.2026E-04	1.0976E-04	2.2448E+00	1.2164E+00
CCNA_02402	1.7075E-04	1.5287E-04	1.5953E-01	-1.1360E-01
CCNA_02403	2.4006E-04	1.1411E-04	1.0728E+00	4.6895E-01
CCNA_02404	9.5909E-05	8.9160E-05	1.0520E-01	-1.4825E-01
CCNA_02405	6.1667E-05	9.6320E-05	-6.4335E-01	-6.2571E-01
CCNA_02406	1.4695E-04	3.9985E-05	1.8775E+00	9.8222E-01
CCNA_02407	1.5123E-04	4.0573E-05	1.8979E+00	9.9521E-01
CCNA_02408	3.7573E-04	5.6700E-05	2.7281E+00	1.5247E+00
CCNA_02409	4.0175E-04	6.6313E-05	2.5988E+00	1.4423E+00
CCNA_02410	1.0401E-04	1.1466E-04	-1.4067E-01	-3.0508E-01
CCNA_02411	2.5857E-04	1.0948E-04	1.2398E+00	5.7543E-01
CCNA_02412	2.6368E-04	1.1357E-04	1.2152E+00	5.5975E-01
CCNA_02413	1.4367E-04	1.6255E-04	-1.7814E-01	-3.2898E-01
CCNA_02414	1.9993E-04	1.3558E-04	5.6036E-01	1.4207E-01
CCNA_02415	2.0253E-04	1.4147E-04	5.1762E-01	1.1481E-01
CCNA_02416	4.5236E-04	1.6033E-04	1.4964E+00	7.3910E-01
CCNA_02417	1.3991E-04	4.8562E-05	1.5264E+00	7.5826E-01
CCNA_02418	1.8699E-04	9.8110E-05	9.3040E-01	3.7810E-01
CCNA_02419	7.8920E-05	3.7797E-05	1.0619E+00	4.6196E-01
CCNA_02420	1.4938E-04	1.4021E-04	9.1348E-02	-1.5709E-01
CCNA_02421	1.9083E-04	1.5774E-04	2.7472E-01	-4.0121E-02
CCNA_02422	5.5910E-05	1.0091E-04	-8.5190E-01	-7.5873E-01
CCNA_02423	1.2394E-04	1.3693E-04	-1.4374E-01	-3.0704E-01
CCNA_02424	1.1484E-04	9.5914E-05	2.5980E-01	-4.9641E-02
CCNA_02425	2.4835E-04	1.6115E-04	6.2394E-01	1.8262E-01
CCNA_02426	7.8874E-05	5.5208E-05	5.1453E-01	1.1283E-01
CCNA_02427	3.1404E-04	4.8852E-05	2.6842E+00	1.4968E+00
CCNA_02428	4.5261E-04	1.6049E-04	1.4958E+00	7.3871E-01
CCNA_02429	1.5126E-04	1.4187E-04	9.2373E-02	-1.5643E-01
CCNA_02430	1.3642E-04	6.4175E-05	1.0878E+00	4.7849E-01
CCNA_02431	1.1432E-04	9.4439E-05	2.7552E-01	-3.9613E-02
CCNA_02432	8.3012E-05	6.0893E-05	4.4693E-01	6.9716E-02
CCNA_02433	1.0454E-04	1.5901E-04	-6.0506E-01	-6.0129E-01
CCNA_02434	7.0199E-05	1.5453E-04	-1.1383E+00	-9.4143E-01
CCNA_02435	2.7759E-04	1.9009E-04	5.4625E-01	1.3307E-01
CCNA_02436	5.8811E-04	5.0595E-04	2.1709E-01	-7.6882E-02

CCNA_02437	7.3995E-05	9.9271E-05	-4.2397E-01	-4.8577E-01
CCNA_02438	8.1889E-05	1.2300E-04	-5.8690E-01	-5.8970E-01
CCNA_02439	1.1995E-04	1.2995E-04	-1.1550E-01	-2.8902E-01
CCNA_02440	1.8608E-04	9.0718E-05	1.0364E+00	4.4570E-01
CCNA_02441	1.7431E-04	3.4880E-05	2.3209E+00	1.2650E+00
CCNA_02442	7.9845E-05	1.1954E-04	-5.8225E-01	-5.8674E-01
CCNA_02443	8.3377E-05	2.4513E-05	1.7657E+00	9.1088E-01
CCNA_02444	9.9100E-05	7.0904E-05	4.8291E-01	9.2669E-02
CCNA_02445	9.7139E-05	4.8206E-05	1.0107E+00	4.2929E-01
CCNA_02446	1.0239E-04	1.8886E-05	2.4381E+00	1.3397E+00
CCNA_02447	1.5742E-04	8.1876E-06	4.2636E+00	2.5042E+00
CCNA_02448	4.7999E-04	9.4000E-05	2.3522E+00	1.2850E+00
CCNA_02449	5.6165E-04	9.9005E-05	2.5040E+00	1.3818E+00
CCNA_02450	1.5812E-04	5.4786E-05	1.5289E+00	7.5988E-01
CCNA_02451	6.7457E-05	9.3221E-05	-4.6672E-01	-5.1305E-01
CCNA_02452	6.4289E-05	9.2541E-05	-5.2555E-01	-5.5057E-01
CCNA_02453	6.6564E-05	1.4860E-04	-1.1586E+00	-9.5437E-01
CCNA_02454	6.4331E-05	4.7609E-05	4.3412E-01	6.1551E-02
CCNA_02455	9.5766E-05	1.6757E-04	-8.0721E-01	-7.3022E-01
CCNA_02456	4.6149E-05	5.2929E-05	-1.9786E-01	-3.4155E-01
CCNA_02457	1.3380E-04	8.8647E-05	5.9384E-01	1.6343E-01
CCNA_02458	1.3335E-04	1.0202E-04	3.8623E-01	3.1000E-02
CCNA_02459	9.8586E-05	1.3936E-04	-4.9941E-01	-5.3390E-01
CCNA_02460	1.0299E-04	1.2987E-04	-3.3460E-01	-4.2877E-01
CCNA_02461	1.3976E-04	9.3901E-05	5.7366E-01	1.5055E-01
CCNA_02462	2.6604E-04	1.0538E-04	1.3360E+00	6.3679E-01
CCNA_02463	6.0997E-05	1.9531E-04	-1.6789E+00	-1.2862E+00
CCNA_02464	1.8246E-04	1.8716E-04	-3.6754E-02	-2.3879E-01
CCNA_02465	9.4480E-05	1.1629E-04	-2.9970E-01	-4.0652E-01
CCNA_02466	9.9331E-05	2.8019E-05	1.8255E+00	9.4903E-01
CCNA_02467	1.8724E-04	9.2715E-05	1.0139E+00	4.3136E-01
CCNA_02468	7.9872E-05	1.2513E-04	-6.4763E-01	-6.2843E-01
CCNA_02469	1.3349E-04	1.3401E-04	-5.6067E-03	-2.1893E-01
CCNA_02470	5.5013E-05	9.6967E-05	-8.1772E-01	-7.3692E-01
CCNA_02471	7.3713E-05	2.6461E-05	1.4777E+00	7.2720E-01
CCNA_02472	5.8070E-05	1.2179E-04	-1.0686E+00	-8.9694E-01
CCNA_02473	5.2659E-05	6.3810E-05	-2.7716E-01	-3.9213E-01
CCNA_02474	7.8901E-05	6.4274E-05	2.9571E-01	-2.6738E-02
CCNA_02475	7.3866E-05	3.1308E-05	1.2381E+00	5.7433E-01
CCNA_02476	7.2233E-05	5.1786E-05	4.7997E-01	9.0794E-02
CCNA_02477	7.3551E-05	1.8588E-05	1.9838E+00	1.0500E+00
CCNA_02478	6.9533E-05	1.0273E-04	-5.6306E-01	-5.7449E-01
CCNA_02479	4.8132E-05	1.3060E-05	1.8810E+00	9.8445E-01
CCNA_02480	5.6447E-05	1.0421E-04	-8.8453E-01	-7.7954E-01
CCNA_02481	9.8693E-05	1.2034E-04	-2.8609E-01	-3.9783E-01
CCNA_02482	5.8070E-05	9.3851E-05	-6.9260E-01	-6.5712E-01
CCNA_02483	5.0629E-05	1.1318E-04	-1.1605E+00	-9.5556E-01
CCNA_02484	5.8870E-05	9.7431E-05	-7.2686E-01	-6.7898E-01
CCNA_02485	6.1852E-05	1.1051E-04	-8.3725E-01	-7.4939E-01
CCNA_02486	5.6160E-05	4.6863E-05	2.6095E-01	-4.8908E-02
CCNA_02487	5.6373E-05	7.3100E-05	-3.7492E-01	-4.5449E-01
CCNA_02488	5.2322E-05	1.1914E-04	-1.1872E+00	-9.7258E-01
CCNA_02489	6.6842E-05	1.2452E-04	-8.9757E-01	-7.8786E-01

CCNA_02490	5.6844E-05	1.1776E-05	2.2703E+00	1.2327E+00
CCNA_02491	2.6718E-05	3.3148E-07	6.2974E+00	3.8014E+00
CCNA_02492	4.8336E-05	8.4710E-05	-8.0944E-01	-7.3165E-01
CCNA_02493	6.0900E-05	1.3483E-04	-1.1466E+00	-9.4671E-01
CCNA_02494	6.2231E-05	2.9751E-05	1.0644E+00	4.6359E-01
CCNA_02495	5.0648E-05	1.0284E-04	-1.0218E+00	-8.6712E-01
CCNA_02496	5.9050E-05	9.0370E-05	-6.1393E-01	-6.0694E-01
CCNA_02497	1.2643E-04	3.3372E-05	1.9214E+00	1.0102E+00
CCNA_02498	1.3695E-04	3.8634E-05	1.8255E+00	9.4901E-01
CCNA_02499	7.0236E-05	3.3314E-06	4.3945E+00	2.5877E+00
CCNA_02500	5.1749E-05	1.1750E-04	-1.1831E+00	-9.6996E-01
CCNA_02501	5.6382E-05	9.3387E-05	-7.2800E-01	-6.7970E-01
CCNA_02502	6.7896E-05	7.6597E-05	-1.7402E-01	-3.2635E-01
CCNA_02503	4.7702E-05	5.8813E-05	-3.0214E-01	-4.0807E-01
CCNA_02504	9.3218E-05	5.5540E-05	7.4694E-01	2.6108E-01
CCNA_02505	7.3676E-05	1.6306E-04	-1.1461E+00	-9.4637E-01
CCNA_02506	3.7014E-04	1.9028E-04	9.5990E-01	3.9691E-01
CCNA_02507	2.9706E-04	3.0111E-04	-1.9562E-02	-2.2783E-01
CCNA_02508	8.2897E-05	1.6120E-04	-9.5946E-01	-8.2733E-01
CCNA_02509	9.5021E-05	4.7468E-05	1.0011E+00	4.2319E-01
CCNA_02510	4.8701E-05	8.7843E-05	-8.5096E-01	-7.5813E-01
CCNA_02511	6.3577E-05	4.6921E-05	4.3811E-01	6.4096E-02
CCNA_02512	3.4353E-04	5.0170E-05	2.7753E+00	1.5549E+00
CCNA_02513	2.1272E-04	1.6274E-04	3.8633E-01	3.1064E-02
CCNA_02514	2.3855E-04	1.7830E-04	4.2000E-01	5.2543E-02
CCNA_02515	7.4499E-05	1.7676E-05	2.0748E+00	1.1081E+00
CCNA_02516	5.4185E-05	7.3150E-05	-4.3299E-01	-4.9153E-01
CCNA_02517	5.6678E-05	5.1512E-05	1.3775E-01	-1.2749E-01
CCNA_02518	4.7883E-05	1.2051E-04	-1.3315E+00	-1.0647E+00
CCNA_02519	1.0456E-04	1.0147E-04	4.3098E-02	-1.8786E-01
CCNA_02520	1.6946E-04	1.1953E-04	5.0344E-01	1.0577E-01
CCNA_02521	1.7158E-04	1.3447E-04	3.5162E-01	8.9235E-03
CCNA_02522	8.8843E-05	1.3726E-04	-6.2757E-01	-6.1564E-01
CCNA_02523	1.1669E-04	8.6450E-05	4.3271E-01	6.0647E-02
CCNA_04001	2.1283E-04	1.2392E-04	7.8014E-01	2.8226E-01
CCNA_02525	2.5998E-04	1.6393E-04	6.6529E-01	2.0900E-01
CCNA_02526	5.8671E-05	3.2104E-05	8.6963E-01	3.3934E-01
CCNA_02527	5.7339E-05	9.3867E-05	-7.1112E-01	-6.6893E-01
CCNA_02528	4.6595E-04	1.6104E-04	1.5327E+00	7.6225E-01
CCNA_02529	6.6106E-05	1.2281E-04	-8.9351E-01	-7.8527E-01
CCNA_02530	1.7655E-04	2.6999E-05	2.7087E+00	1.5124E+00
CCNA_02531	5.6604E-05	1.2218E-04	-1.1100E+00	-9.2334E-01
CCNA_02532	1.1432E-04	1.1293E-04	1.7600E-02	-2.0413E-01
CCNA_02533	7.1350E-05	1.5765E-04	-1.1437E+00	-9.4488E-01
CCNA_02534	2.1413E-04	1.8191E-04	2.3526E-01	-6.5292E-02
CCNA_02535	2.2588E-04	1.6952E-04	4.1405E-01	4.8750E-02
CCNA_04018	1.1089E-04	1.5975E-04	-5.2674E-01	-5.5133E-01
CCNA_02537	4.5201E-05	4.1062E-05	1.3839E-01	-1.2708E-01
CCNA_02538	1.8792E-04	1.1964E-04	6.5139E-01	2.0013E-01
CCNA_02539	1.8829E-04	1.6174E-04	2.1929E-01	-7.5480E-02
CCNA_02540	1.0915E-04	1.3958E-04	-3.5476E-01	-4.4163E-01
CCNA_02541	1.0953E-04	1.4090E-04	-3.6340E-01	-4.4714E-01
CCNA_02542	1.7740E-04	1.6267E-04	1.2509E-01	-1.3556E-01

CCNA_02543	3.8875E-04	5.0892E-04	-3.8859E-01	-4.6321E-01
CCNA_02544	1.0759E-04	6.5194E-05	7.2266E-01	2.4559E-01
CCNA_02545	9.8244E-05	1.5264E-04	-6.3569E-01	-6.2082E-01
CCNA_02546	2.5711E-04	1.0939E-04	1.2329E+00	5.7102E-01
CCNA_02547	2.7093E-04	1.6243E-04	7.3807E-01	2.5542E-01
CCNA_02548	2.9293E-04	1.5870E-04	8.8425E-01	3.4866E-01
CCNA_02549	1.5621E-04	1.2842E-04	2.8249E-01	-3.5166E-02
CCNA_02550	5.6558E-05	1.4745E-04	-1.3824E+00	-1.0971E+00
CCNA_02551	7.2710E-05	3.7068E-05	9.7174E-01	4.0447E-01
CCNA_02552	1.7374E-04	1.5367E-04	1.7706E-01	-1.0241E-01
CCNA_02553	3.2422E-04	3.1491E-05	3.3636E+00	1.9301E+00
CCNA_02554	1.9056E-04	2.2593E-04	-2.4561E-01	-3.7201E-01
CCNA_02555	8.2504E-05	8.6923E-05	-7.5336E-02	-2.6340E-01
CCNA_02556	8.9010E-05	1.3734E-04	-6.2574E-01	-6.1448E-01
CCNA_02557	4.2019E-05	1.2972E-04	-1.6262E+00	-1.2526E+00
CCNA_02558	6.3295E-05	1.4026E-04	-1.1479E+00	-9.4753E-01
CCNA_02559	4.8299E-05	6.5393E-05	-4.3719E-01	-4.9421E-01
CCNA_02560	5.6192E-05	6.8799E-05	-2.9207E-01	-4.0165E-01
CCNA_02561	6.1186E-05	6.2236E-05	-2.4616E-02	-2.3105E-01
CCNA_02562	3.9689E-04	1.6057E-04	1.3055E+00	6.1735E-01
CCNA_02563	7.6941E-05	6.8783E-05	1.6162E-01	-1.1226E-01
CCNA_02564	9.5433E-05	1.1991E-04	-3.2937E-01	-4.2544E-01
CCNA_02565	9.3708E-05	1.4025E-04	-5.8177E-01	-5.8643E-01
CCNA_02566	1.4809E-04	1.5594E-04	-7.4504E-02	-2.6287E-01
CCNA_02567	2.6191E-04	8.2456E-05	1.6673E+00	8.4811E-01
CCNA_02568	1.0991E-04	1.6238E-04	-5.6310E-01	-5.7452E-01
CCNA_02569	1.1036E-04	6.7042E-05	7.1895E-01	2.4322E-01
CCNA_02570	1.2130E-04	2.5392E-05	2.2557E+00	1.2235E+00
CCNA_02571	5.0930E-05	4.4037E-05	2.0965E-01	-8.1629E-02
CCNA_02572	5.7339E-05	5.6625E-05	1.7975E-02	-2.0389E-01
CCNA_02573	5.9684E-05	7.0324E-05	-2.3674E-01	-3.6635E-01
CCNA_02574	5.3228E-05	1.1339E-04	-1.0910E+00	-9.1126E-01
CCNA_02575	7.1992E-04	3.2485E-04	1.1480E+00	5.1690E-01
CCNA_02576	7.3256E-04	2.8261E-04	1.3741E+00	6.6110E-01
CCNA_02577	7.0282E-05	1.6243E-04	-1.2085E+00	-9.8621E-01
CCNA_02578	5.1480E-05	5.6783E-05	-1.4152E-01	-3.0562E-01
CCNA_02579	4.1700E-05	0.0000E+00	1.2297E+01	7.6282E+00
CCNA_02580	9.0383E-05	9.3950E-05	-5.5899E-02	-2.5101E-01
CCNA_02581	4.9386E-05	1.2756E-04	-1.3690E+00	-1.0886E+00
CCNA_02582	4.8715E-05	6.0562E-05	-3.1410E-01	-4.1570E-01
CCNA_02583	6.6032E-05	1.7278E-04	-1.3877E+00	-1.1005E+00
CCNA_04012	5.9536E-05	1.2726E-04	-1.0959E+00	-9.1436E-01
CCNA_02585	1.0029E-04	1.3340E-04	-4.1169E-01	-4.7794E-01
CCNA_02586	9.9331E-05	1.5965E-04	-6.8461E-01	-6.5202E-01
CCNA_02587	1.1737E-04	9.7920E-05	2.6132E-01	-4.8671E-02
CCNA_02588	2.7089E-04	2.4331E-05	3.4764E+00	2.0020E+00
CCNA_02589	2.5641E-04	2.5715E-05	3.3173E+00	1.9006E+00
CCNA_02590	2.0791E-04	1.2287E-04	7.5870E-01	2.6858E-01
CCNA_02591	9.4804E-05	1.0384E-04	-1.3135E-01	-2.9913E-01
CCNA_02592	8.4372E-05	1.6183E-04	-9.3964E-01	-8.1469E-01
CCNA_02593	9.0059E-05	8.2854E-05	1.2024E-01	-1.3866E-01
CCNA_02594	7.9674E-05	1.1153E-04	-4.8524E-01	-5.2486E-01
CCNA_02595	7.2483E-05	3.6819E-05	9.7695E-01	4.0779E-01

CCNA_02596	7.8388E-05	1.0302E-04	-3.9431E-01	-4.6686E-01
CCNA_02597	1.4211E-04	1.4313E-04	-1.0363E-02	-2.2196E-01
CCNA_02598	1.7222E-04	9.7323E-05	8.2332E-01	3.0980E-01
CCNA_02599	1.2100E-04	1.3040E-04	-1.0804E-01	-2.8426E-01
CCNA_02600	4.8826E-05	1.4674E-04	-1.5875E+00	-1.2279E+00
CCNA_02601	1.6661E-04	3.0868E-04	-8.8964E-01	-7.8280E-01
CCNA_02602	1.8163E-04	2.9725E-04	-7.1066E-01	-6.6864E-01
CCNA_02603	8.4358E-05	6.5857E-05	3.5708E-01	1.2408E-02
CCNA_02604	4.6692E-04	7.4501E-05	2.6477E+00	1.4735E+00
CCNA_02605	5.4412E-05	7.4882E-05	-4.6073E-01	-5.0922E-01
CCNA_02606	2.9220E-04	1.2008E-04	1.2829E+00	6.0294E-01
CCNA_02607	2.8556E-04	1.1794E-04	1.2757E+00	5.9832E-01
CCNA_02608	8.7655E-05	5.4255E-05	6.9192E-01	2.2599E-01
CCNA_02609	4.8683E-05	1.4961E-04	-1.6197E+00	-1.2485E+00
CCNA_02610	6.7762E-05	1.4551E-04	-1.1026E+00	-9.1862E-01
CCNA_02611	1.2347E-04	1.5634E-04	-3.4052E-01	-4.3255E-01
CCNA_02612	1.4963E-04	1.5952E-04	-9.2371E-02	-2.7427E-01
CCNA_02613	6.3785E-05	8.9881E-05	-4.9483E-01	-5.3098E-01
CCNA_02614	5.6451E-05	1.3856E-04	-1.2954E+00	-1.0416E+00
CCNA_02615	5.8037E-05	7.4658E-05	-3.6336E-01	-4.4712E-01
CCNA_02616	5.2146E-05	5.8200E-05	-1.5853E-01	-3.1647E-01
CCNA_02617	1.2492E-03	1.3695E-04	3.1892E+00	1.8188E+00
CCNA_02618	1.5147E-04	1.4710E-04	4.2267E-02	-1.8839E-01
CCNA_02619	1.5256E-04	1.0660E-04	5.1703E-01	1.1443E-01
CCNA_02620	8.6383E-05	7.8942E-05	1.2988E-01	-1.3251E-01
CCNA_02621	4.9959E-05	5.9949E-05	-2.6305E-01	-3.8313E-01
CCNA_02622	8.4635E-05	1.2585E-04	-5.7236E-01	-5.8043E-01
CCNA_02623	5.2048E-04	1.0969E-04	2.2464E+00	1.2175E+00
CCNA_02624	9.6135E-05	1.0347E-04	-1.0615E-01	-2.8306E-01
CCNA_02625	2.2007E-04	1.6243E-04	4.3814E-01	6.4114E-02
CCNA_02626	8.1098E-05	3.5783E-05	1.1801E+00	5.3738E-01
CCNA_02627	6.6476E-05	5.8275E-05	1.8987E-01	-9.4244E-02
CCNA_02628	3.5412E-05	1.1667E-04	-1.7201E+00	-1.3125E+00
CCNA_02629	1.0213E-04	1.0588E-04	-5.2079E-02	-2.4857E-01
CCNA_02630	1.3650E-04	1.6277E-04	-2.5393E-01	-3.7732E-01
CCNA_02631	9.3527E-05	1.0244E-04	-1.3143E-01	-2.9918E-01
CCNA_02632	7.1789E-05	5.3004E-05	4.3754E-01	6.3729E-02
CCNA_02633	5.7875E-05	1.3616E-04	-1.2342E+00	-1.0026E+00
CCNA_02634	5.9448E-05	4.2397E-05	4.8750E-01	9.5599E-02
CCNA_02635	1.2240E-04	1.6243E-04	-4.0819E-01	-4.7571E-01
CCNA_02636	1.3283E-04	1.5634E-04	-2.3506E-01	-3.6528E-01
CCNA_02637	1.2715E-04	1.2847E-04	-1.4993E-02	-2.2491E-01
CCNA_02638	5.5600E-05	1.4515E-04	-1.3843E+00	-1.0983E+00
CCNA_02639	5.9286E-05	9.4033E-05	-6.6550E-01	-6.3983E-01
CCNA_02640	2.6040E-04	1.0641E-04	1.2911E+00	6.0815E-01
CCNA_02641	4.4979E-05	1.4227E-04	-1.6613E+00	-1.2750E+00
CCNA_02642	4.9131E-05	5.5871E-05	-1.8554E-01	-3.3370E-01
CCNA_02643	4.3925E-05	1.1818E-04	-1.4279E+00	-1.1261E+00
CCNA_02644	4.7753E-05	3.2361E-05	5.6112E-01	1.4255E-01
CCNA_02645	1.2452E-04	1.4908E-05	3.0615E+00	1.7374E+00
CCNA_02646	9.1983E-05	1.5928E-04	-7.9210E-01	-7.2059E-01
CCNA_02647	4.9820E-05	9.0809E-05	-8.6611E-01	-7.6779E-01
CCNA_02648	6.4941E-05	1.0414E-04	-6.8126E-01	-6.4989E-01

CCNA_02649	4.9954E-05	3.7375E-05	4.1837E-01	5.1500E-02
CCNA_02650	1.2778E-04	5.8291E-05	1.1322E+00	5.0681E-01
CCNA_02651	7.8120E-05	1.5627E-04	-1.0003E+00	-8.5336E-01
CCNA_02652	7.8037E-05	1.5589E-04	-9.9827E-01	-8.5209E-01
CCNA_02653	6.4298E-05	6.6901E-05	-5.7328E-02	-2.5192E-01
CCNA_02654	5.8509E-05	2.8027E-05	1.0615E+00	4.6174E-01
CCNA_02655	5.1850E-05	8.0600E-05	-6.3645E-01	-6.2130E-01
CCNA_02656	5.2965E-05	4.6175E-05	1.9777E-01	-8.9204E-02
CCNA_02657	5.5711E-05	8.0931E-05	-5.3875E-01	-5.5899E-01
CCNA_02658	2.3735E-04	4.4982E-05	2.3994E+00	1.3151E+00
CCNA_02659	2.4749E-04	5.3750E-05	2.2028E+00	1.1897E+00
CCNA_02660	9.2020E-05	1.6038E-04	-8.0147E-01	-7.2656E-01
CCNA_02661	7.2358E-05	1.5555E-04	-1.1041E+00	-9.1960E-01
CCNA_02662	1.7447E-04	4.5280E-05	1.9458E+00	1.0257E+00
CCNA_02663	2.6744E-04	1.6274E-04	7.1659E-01	2.4172E-01
CCNA_02664	1.4526E-04	1.2507E-04	2.1589E-01	-7.7645E-02
CCNA_02665	1.3958E-04	1.3973E-04	-1.5757E-03	-2.1636E-01
CCNA_02666	5.8467E-05	5.2424E-05	1.5729E-01	-1.1502E-01
CCNA_02667	8.3789E-05	9.8442E-05	-2.3255E-01	-3.6368E-01
CCNA_02668	4.8720E-05	7.9274E-05	-7.0235E-01	-6.6334E-01
CCNA_02669	3.2835E-04	1.2972E-04	1.3398E+00	6.3924E-01
CCNA_02670	1.4182E-04	1.6243E-04	-1.9573E-01	-3.4020E-01
CCNA_02671	9.0808E-05	5.5573E-05	7.0830E-01	2.3643E-01
CCNA_02672	2.0539E-04	1.4585E-04	4.9381E-01	9.9620E-02
CCNA_02673	1.8709E-04	1.3885E-04	4.3017E-01	5.9032E-02
CCNA_02674	8.3327E-05	0.0000E+00	1.3296E+01	8.2652E+00
CCNA_02675	0.0000E+00	0.0000E+00	-8.4168E-01	-7.5221E-01
CCNA_02676	1.4029E-04	1.4972E-04	-9.3943E-02	-2.7527E-01
CCNA_02677	1.4634E-04	1.5784E-04	-1.0920E-01	-2.8501E-01
CCNA_02678	5.4172E-05	1.5075E-04	-1.4765E+00	-1.1571E+00
CCNA_02679	4.2707E-04	1.6276E-04	1.3917E+00	6.7233E-01
CCNA_02680	1.1506E-04	5.2142E-05	1.1417E+00	5.1288E-01
CCNA_02681	1.1071E-04	5.8067E-05	9.3085E-01	3.7838E-01
CCNA_02682	4.9954E-05	4.0242E-05	3.1175E-01	-1.6505E-02
CCNA_02683	7.2696E-05	5.1968E-05	4.8411E-01	9.3433E-02
CCNA_02684	7.7371E-05	9.6892E-05	-3.2463E-01	-4.2241E-01
CCNA_02685	6.1455E-05	4.2728E-05	5.2417E-01	1.1898E-01
CCNA_02686	9.6700E-05	4.7767E-05	1.0173E+00	4.3354E-01
CCNA_02687	5.1628E-05	1.4044E-04	-1.4437E+00	-1.1362E+00
CCNA_02688	2.9386E-05	6.1714E-05	-1.0704E+00	-8.9811E-01
CCNA_02689	5.1429E-05	1.3137E-04	-1.3529E+00	-1.0783E+00
CCNA_02690	1.0838E-04	1.6267E-04	-5.8584E-01	-5.8903E-01
CCNA_02691	1.2350E-04	6.7879E-05	8.6330E-01	3.3530E-01
CCNA_02692	6.6250E-05	5.5532E-05	2.5449E-01	-5.3025E-02
CCNA_02693	8.6490E-05	5.4943E-05	6.5445E-01	2.0208E-01
CCNA_02694	1.0454E-04	9.9701E-05	6.8341E-02	-1.7176E-01
CCNA_02695	1.0894E-04	1.3227E-04	-2.7998E-01	-3.9394E-01
CCNA_02696	8.3433E-05	1.8538E-05	2.1696E+00	1.1685E+00
CCNA_02697	5.1198E-05	5.3932E-05	-7.5141E-02	-2.6328E-01
CCNA_02698	9.2108E-05	1.6166E-04	-8.1160E-01	-7.3303E-01
CCNA_02699	4.0470E-05	1.8679E-05	1.1150E+00	4.9582E-01
CCNA_02700	4.5478E-05	5.0675E-05	-1.5620E-01	-3.1498E-01
CCNA_02701	5.3714E-05	1.3359E-04	-1.3144E+00	-1.0537E+00

CCNA_02702	6.0978E-05	1.3661E-04	-1.1637E+00	-9.5761E-01
CCNA_02703	1.1725E-04	8.7445E-05	4.2305E-01	5.4488E-02
CCNA_02704	1.3226E-04	1.3176E-04	5.3693E-03	-2.1193E-01
CCNA_02705	1.6021E-04	1.1591E-04	4.6690E-01	8.2459E-02
CCNA_02706	4.6259E-04	4.5579E-06	6.6626E+00	4.0344E+00
CCNA_02707	1.9629E-04	3.4085E-04	-7.9618E-01	-7.2319E-01
CCNA_02708	4.1134E-04	5.5260E-04	-4.2591E-01	-4.8701E-01
CCNA_02709	7.3181E-05	2.4861E-08	1.1108E+01	6.8701E+00
CCNA_02710	2.6871E-04	1.2281E-04	1.1296E+00	5.0515E-01
CCNA_02711	2.6295E-04	1.1519E-04	1.1907E+00	5.4413E-01
CCNA_02712	1.0304E-04	9.5732E-05	1.0606E-01	-1.4770E-01
CCNA_02713	6.4169E-05	4.4220E-05	5.3702E-01	1.2718E-01
CCNA_02714	5.7487E-05	3.3347E-05	7.8543E-01	2.8563E-01
CCNA_02715	1.4703E-04	4.7261E-05	1.6372E+00	8.2893E-01
CCNA_02716	1.0561E-04	7.0117E-05	5.9088E-01	1.6153E-01
CCNA_02717	6.2467E-05	1.4626E-04	-1.2273E+00	-9.9819E-01
CCNA_02718	5.2636E-05	6.3487E-05	-2.7047E-01	-3.8787E-01
CCNA_02719	1.6050E-04	1.6243E-04	-1.7256E-02	-2.2636E-01
CCNA_02720	7.8041E-05	1.5741E-04	-1.0122E+00	-8.6100E-01
CCNA_02721	1.7006E-04	1.1266E-04	5.9398E-01	1.6351E-01
CCNA_02722	1.1644E-04	9.5599E-05	2.8450E-01	-3.3884E-02
CCNA_02723	7.1577E-05	7.2031E-05	-9.2003E-03	-2.2122E-01
CCNA_02724	6.6740E-05	1.5755E-04	-1.2391E+00	-1.0057E+00
CCNA_02725	3.3261E-04	3.0389E-05	3.4518E+00	1.9864E+00
CCNA_02726	5.3214E-04	7.3937E-05	2.8473E+00	1.6008E+00
CCNA_02727	3.9610E-04	1.3663E-04	1.5355E+00	7.6407E-01
CCNA_02728	1.9362E-04	1.0399E-04	8.9673E-01	3.5662E-01
CCNA_02729	1.0622E-04	1.3289E-04	-3.2327E-01	-4.2155E-01
CCNA_02730	6.5778E-05	6.0205E-05	1.2762E-01	-1.3395E-01
CCNA_02731	1.7096E-04	1.6243E-04	7.3826E-02	-1.6826E-01
CCNA_02732	1.6540E-04	1.4710E-04	1.6909E-01	-1.0750E-01
CCNA_02733	7.5831E-05	5.9203E-05	3.5701E-01	1.2365E-02
CCNA_02734	1.4622E-04	1.6243E-04	-1.5168E-01	-3.1210E-01
CCNA_02735	6.9024E-05	8.6509E-05	-3.2578E-01	-4.2315E-01
CCNA_02736	7.4000E-05	1.6180E-04	-1.1286E+00	-9.3525E-01
CCNA_02737	1.0848E-04	1.2402E-04	-1.9310E-01	-3.3852E-01
CCNA_02738	1.1739E-04	6.1076E-05	9.4253E-01	3.8584E-01
CCNA_02739	0.0000E+00	0.0000E+00	-8.4168E-01	-7.5221E-01
CCNA_02740	8.1010E-05	7.3664E-05	1.3707E-01	-1.2792E-01
CCNA_02741	8.3609E-05	7.9340E-05	7.5530E-02	-1.6718E-01
CCNA_02742	7.1008E-05	2.9949E-05	1.2451E+00	5.7886E-01
CCNA_02743	7.1845E-05	1.2066E-04	-7.4799E-01	-6.9245E-01
CCNA_02744	7.7010E-05	1.0163E-04	-4.0027E-01	-4.7066E-01
CCNA_02745	6.6985E-05	3.9223E-05	7.7195E-01	2.7703E-01
CCNA_02746	7.5535E-05	1.3950E-04	-8.8500E-01	-7.7984E-01
CCNA_02747	1.4833E-04	1.3956E-04	8.7886E-02	-1.5929E-01
CCNA_02748	1.3710E-04	1.6276E-04	-2.4756E-01	-3.7326E-01
CCNA_02749	9.0096E-05	4.2256E-05	1.0921E+00	4.8125E-01
CCNA_02750	6.1603E-05	4.3142E-05	5.1371E-01	1.1232E-01
CCNA_02751	5.7501E-05	1.0054E-04	-8.0609E-01	-7.2951E-01
CCNA_02752	1.3942E-04	1.6267E-04	-2.2256E-01	-3.5731E-01
CCNA_02753	5.2590E-05	2.0552E-05	1.3551E+00	6.4897E-01
CCNA_02754	2.4083E-04	1.6243E-04	5.6818E-01	1.4706E-01

CCNA_02755	2.8061E-04	1.0091E-04	1.4754E+00	7.2572E-01
CCNA_02756	1.1195E-04	1.4439E-04	-3.6709E-01	-4.4950E-01
CCNA_02757	1.2158E-04	1.6098E-04	-4.0501E-01	-4.7368E-01
CCNA_02758	7.1105E-05	9.5467E-05	-4.2508E-01	-4.8648E-01
CCNA_02759	1.8885E-04	1.5099E-04	3.2274E-01	-9.4967E-03
CCNA_02760	3.2010E-04	8.3774E-05	1.9339E+00	1.0181E+00
CCNA_02761	1.3035E-04	3.9463E-05	1.7236E+00	8.8404E-01
CCNA_02762	1.8277E-04	9.7994E-05	8.9916E-01	3.5817E-01
CCNA_02763	1.3219E-04	6.8592E-05	9.4633E-01	3.8825E-01
CCNA_02764	2.0888E-04	1.2894E-04	6.9594E-01	2.2855E-01
CCNA_02765	1.3933E-04	1.3449E-04	5.0954E-02	-1.8285E-01
CCNA_02766	1.9109E-04	9.7505E-05	9.7059E-01	4.0373E-01
CCNA_02767	1.5221E-04	2.1488E-05	2.8239E+00	1.5859E+00
CCNA_02768	1.3315E-04	1.6243E-04	-2.8679E-01	-3.9828E-01
CCNA_02769	5.2183E-05	1.1525E-04	-1.1431E+00	-9.4444E-01
CCNA_02770	3.2887E-05	0.0000E+00	1.1955E+01	7.4098E+00
CCNA_02771	1.2846E-04	0.0000E+00	1.3920E+01	8.6635E+00
CCNA_02772	9.6644E-07	0.0000E+00	6.8726E+00	4.1683E+00
CCNA_02773	5.8296E-05	5.2424E-05	1.5307E-01	-1.1772E-01
CCNA_02774	1.3353E-04	7.2304E-05	8.8490E-01	3.4907E-01
CCNA_02775	1.5563E-04	1.6516E-04	-8.5744E-02	-2.7004E-01
CCNA_02776	9.4845E-05	7.8395E-06	3.5953E+00	2.0779E+00
CCNA_02777	2.9968E-04	1.6119E-04	8.9460E-01	3.5526E-01
CCNA_02778	1.4485E-04	9.0528E-05	6.7805E-01	2.1714E-01
CCNA_02779	4.7359E-04	1.2332E-04	1.9412E+00	1.0228E+00
CCNA_02780	1.0815E-04	8.0451E-05	4.2676E-01	5.6851E-02
CCNA_02781	1.6866E-04	6.5708E-05	1.3599E+00	6.5203E-01
CCNA_02782	1.0566E-03	1.6659E-04	2.6650E+00	1.4845E+00
CCNA_02783	1.1074E-03	1.7079E-04	2.6968E+00	1.5048E+00
CCNA_02784	8.0367E-05	1.6222E-04	-1.0133E+00	-8.6165E-01
CCNA_02785	8.7576E-05	8.1752E-05	9.9219E-02	-1.5207E-01
CCNA_02786	1.1783E-04	5.1512E-05	1.1935E+00	5.4592E-01
CCNA_02787	9.3847E-05	4.1949E-05	1.1615E+00	5.2547E-01
CCNA_02788	1.6714E-04	1.4301E-04	2.2489E-01	-7.1907E-02
CCNA_02789	2.4224E-04	9.8666E-05	1.2957E+00	6.1113E-01
CCNA_02790	2.2779E-04	1.0473E-04	1.1209E+00	4.9963E-01
CCNA_02791	1.3889E-04	9.0710E-05	6.1453E-01	1.7662E-01
CCNA_02792	5.0449E-05	5.5623E-05	-1.4093E-01	-3.0524E-01
CCNA_02793	6.1787E-05	0.0000E+00	1.2864E+01	7.9900E+00
CCNA_02794	2.1817E-04	1.2653E-04	7.8586E-01	2.8590E-01
CCNA_02795	6.5399E-05	9.6005E-05	-5.5387E-01	-5.6863E-01
CCNA_02796	6.9968E-05	9.7116E-05	-4.7305E-01	-5.1708E-01
CCNA_02797	6.6458E-05	5.5747E-05	2.5343E-01	-5.3702E-02
CCNA_02798	7.0772E-05	6.8153E-05	5.4330E-02	-1.8070E-01
CCNA_02799	6.8941E-05	8.3103E-05	-2.6958E-01	-3.8730E-01
CCNA_02800	7.7796E-05	1.4884E-05	2.3853E+00	1.3061E+00
CCNA_02801	8.0871E-05	7.7774E-05	5.6268E-02	-1.7946E-01
CCNA_02802	7.6612E-05	1.4213E-04	-8.9157E-01	-7.8403E-01
CCNA_02803	4.5577E-04	1.6258E-04	1.4871E+00	7.3320E-01
CCNA_02804	1.2526E-04	5.6128E-05	1.1579E+00	5.2324E-01
CCNA_02805	4.5577E-04	1.6258E-04	1.4871E+00	7.3320E-01
CCNA_02806	1.4455E-04	1.1738E-04	3.0031E-01	-2.3803E-02
CCNA_02807	5.2206E-05	9.7613E-05	-9.0285E-01	-7.9122E-01

CCNA_02808	6.6444E-05	6.2377E-05	9.1042E-02	-1.5728E-01
CCNA_02809	5.1610E-05	1.3651E-04	-1.4033E+00	-1.1104E+00
CCNA_02810	5.5513E-05	3.8543E-05	5.2615E-01	1.2025E-01
CCNA_02811	5.4283E-05	1.2304E-04	-1.1805E+00	-9.6833E-01
CCNA_02812	6.0460E-05	3.1806E-05	9.2644E-01	3.7557E-01
CCNA_02813	5.3099E-05	0.0000E+00	1.2646E+01	7.8506E+00
CCNA_02814	0.0000E+00	0.0000E+00	-8.4168E-01	-7.5221E-01
CCNA_02815	8.1426E-05	5.0418E-05	6.9139E-01	2.2564E-01
CCNA_02816	2.8575E-04	1.1640E-04	1.2956E+00	6.1103E-01
CCNA_02817	3.1596E-04	1.3419E-04	1.2354E+00	5.7261E-01
CCNA_02818	3.3813E-04	1.1540E-04	1.5509E+00	7.7388E-01
CCNA_02819	7.0851E-05	1.4061E-04	-9.8880E-01	-8.4605E-01
CCNA_02820	8.0954E-05	1.2039E-04	-5.7250E-01	-5.8052E-01
CCNA_02821	6.1223E-05	6.7332E-05	-1.3728E-01	-3.0292E-01
CCNA_02822	1.2195E-04	2.7878E-05	2.1287E+00	1.1424E+00
CCNA_02823	8.2101E-05	1.4669E-04	-8.3729E-01	-7.4941E-01
CCNA_02824	8.5542E-05	1.5131E-04	-8.2284E-01	-7.4019E-01
CCNA_02825	6.7378E-05	5.5813E-05	2.7156E-01	-4.2142E-02
CCNA_02826	1.9284E-04	7.4376E-05	1.3743E+00	6.6125E-01
CCNA_02827	3.7677E-05	2.7347E-07	7.0633E+00	4.2899E+00
CCNA_02828	3.2831E-07	0.0000E+00	5.3282E+00	3.1832E+00
CCNA_02829	2.1622E-05	0.0000E+00	1.1350E+01	7.0240E+00
CCNA_02830	1.0202E-04	5.4446E-05	9.0576E-01	3.6238E-01
CCNA_02831	8.6013E-05	8.3219E-05	4.7588E-02	-1.8500E-01
CCNA_02832	1.9360E-04	1.5856E-04	2.8795E-01	-3.1682E-02
CCNA_02833	5.4689E-05	9.5616E-05	-8.0599E-01	-7.2945E-01
CCNA_02834	6.3115E-05	1.0864E-04	-7.8356E-01	-7.1514E-01
CCNA_02835	6.5450E-05	6.0744E-06	3.4277E+00	1.9710E+00
CCNA_02836	5.3469E-05	1.4277E-04	-1.4169E+00	-1.1191E+00
CCNA_02837	7.6247E-05	1.4309E-04	-9.0819E-01	-7.9463E-01
CCNA_02838	6.6897E-05	5.8258E-06	3.5195E+00	2.0295E+00
CCNA_02839	6.7914E-05	5.9982E-05	1.7910E-01	-1.0112E-01
CCNA_02840	7.5873E-05	1.3435E-04	-8.2434E-01	-7.4115E-01
CCNA_02841	4.3763E-05	5.9667E-07	6.1769E+00	3.7245E+00
CCNA_02842	7.3843E-05	1.4995E-04	-1.0220E+00	-8.6722E-01
CCNA_02843	1.0887E-04	9.5947E-05	1.8217E-01	-9.9156E-02
CCNA_02844	1.1251E-04	1.6243E-04	-5.2975E-01	-5.5325E-01
CCNA_02845	1.6602E-04	1.2303E-04	4.3225E-01	6.0358E-02
CCNA_02846	1.0532E-04	1.6243E-04	-6.2503E-01	-6.1402E-01
CCNA_02847	8.5380E-05	1.4746E-04	-7.8836E-01	-7.1820E-01
CCNA_02848	5.6854E-05	4.3051E-05	4.0104E-01	4.0446E-02
CCNA_02849	1.0220E-04	1.0824E-04	-8.2879E-02	-2.6821E-01
CCNA_02850	2.6353E-04	1.2963E-04	1.0234E+00	4.3744E-01
CCNA_02851	2.6330E-04	1.1594E-04	1.1833E+00	5.3940E-01
CCNA_02852	1.3884E-04	1.4355E-04	-4.8108E-02	-2.4604E-01
CCNA_02853	5.7122E-05	1.2642E-04	-1.1461E+00	-9.4637E-01
CCNA_02854	7.4083E-05	9.1572E-05	-3.0580E-01	-4.1041E-01
CCNA_02855	1.0002E-04	4.2314E-05	1.2409E+00	5.7613E-01
CCNA_02856	5.4560E-05	2.9436E-05	8.9000E-01	3.5233E-01
CCNA_02857	6.1501E-05	9.2832E-05	-5.9403E-01	-5.9425E-01
CCNA_02858	4.5474E-05	4.6739E-06	3.2799E+00	1.8767E+00
CCNA_02859	5.3834E-05	9.4497E-05	-8.1176E-01	-7.3312E-01
CCNA_02860	1.0499E-04	9.0536E-05	2.1363E-01	-7.9090E-02

CCNA_02861	5.3127E-05	4.1518E-05	3.5553E-01	1.1420E-02
CCNA_02862	4.1599E-05	9.6204E-05	-1.2095E+00	-9.8684E-01
CCNA_02863	4.3564E-05	3.5974E-05	2.7599E-01	-3.9311E-02
CCNA_02864	5.2794E-05	5.4769E-05	-5.3092E-02	-2.4922E-01
CCNA_02865	2.7220E-04	1.4158E-04	9.4305E-01	3.8616E-01
CCNA_02866	2.3608E-04	1.1101E-04	1.0885E+00	4.7893E-01
CCNA_02867	4.9880E-05	4.8123E-05	5.1634E-02	-1.8242E-01
CCNA_02868	4.4419E-05	5.2208E-07	6.3882E+00	3.8593E+00
CCNA_02869	4.1534E-05	9.1108E-05	-1.1333E+00	-9.3819E-01
CCNA_02870	4.4600E-05	9.1820E-06	2.2790E+00	1.2383E+00
CCNA_02871	5.2530E-05	4.9407E-05	8.8300E-02	-1.5903E-01
CCNA_02872	4.5501E-05	1.3970E-04	-1.6183E+00	-1.2476E+00
CCNA_02873	4.5344E-05	1.3950E-04	-1.6212E+00	-1.2494E+00
CCNA_02874	3.6512E-05	9.6486E-05	-1.4019E+00	-1.1095E+00
CCNA_02875	3.2854E-05	5.8838E-07	5.7832E+00	3.4734E+00
CCNA_02877	6.0285E-05	1.4516E-04	-1.2677E+00	-1.0240E+00
CCNA_02876	5.0394E-05	6.6015E-05	-3.8959E-01	-4.6385E-01
CCNA_02878	3.3724E-05	3.7441E-05	-1.5097E-01	-3.1165E-01
CCNA_02879	3.0311E-05	7.8686E-05	-1.3762E+00	-1.0931E+00
CCNA_02880	7.6275E-05	1.1541E-04	-5.9745E-01	-5.9643E-01
CCNA_02881	7.2640E-05	9.9329E-05	-4.5147E-01	-5.0332E-01
CCNA_02882	5.8463E-05	7.3680E-05	-3.3381E-01	-4.2827E-01
CCNA_02883	7.2724E-05	2.6618E-05	1.4497E+00	7.0930E-01
CCNA_02884	6.5908E-05	4.5562E-05	5.3245E-01	1.2426E-01
CCNA_02885	5.4528E-05	6.1498E-05	-1.7363E-01	-3.2610E-01
CCNA_02886	7.7075E-05	5.3219E-05	5.3417E-01	1.2536E-01
CCNA_02887	4.5025E-05	1.1756E-04	-1.3846E+00	-1.0985E+00
CCNA_02888	4.0253E-05	6.1357E-05	-6.0817E-01	-6.0327E-01
CCNA_02889	1.1599E-04	3.5767E-05	1.6970E+00	8.6706E-01
CCNA_02890	1.5621E-04	1.4322E-04	1.2526E-01	-1.3545E-01
CCNA_02891	1.3851E-04	6.4838E-05	1.0949E+00	4.8303E-01
CCNA_02892	5.5397E-05	7.5851E-05	-4.5341E-01	-5.0455E-01
CCNA_02893	4.9746E-05	5.9178E-05	-2.5054E-01	-3.7515E-01
CCNA_02894	4.6176E-05	1.5538E-05	1.5707E+00	7.8651E-01
CCNA_02895	2.3192E-04	1.3300E-04	8.0217E-01	2.9631E-01
CCNA_02896	1.1067E-04	9.9685E-05	1.5075E-01	-1.1920E-01
CCNA_02897	5.8578E-05	7.9042E-05	-4.3229E-01	-4.9108E-01
CCNA_02898	4.8669E-05	1.2107E-05	2.0063E+00	1.0643E+00
CCNA_02899	8.8150E-05	5.1280E-05	7.8139E-01	2.8305E-01
CCNA_02900	5.3145E-05	1.3473E-04	-1.3420E+00	-1.0714E+00
CCNA_02901	8.7618E-05	5.7504E-05	6.0743E-01	1.7210E-01
CCNA_02902	1.0570E-04	7.9771E-05	4.0599E-01	4.3604E-02
CCNA_02903	1.0671E-04	7.6067E-05	4.8821E-01	9.6049E-02
CCNA_02904	1.1873E-04	1.2604E-04	-8.6167E-02	-2.7031E-01
CCNA_02905	5.7547E-05	4.1560E-05	4.6939E-01	8.4047E-02
CCNA_02906	1.1625E-04	1.6333E-04	-4.9057E-01	-5.2825E-01
CCNA_02907	1.9125E-05	0.0000E+00	1.1173E+01	6.9111E+00
CCNA_02908	7.6353E-05	9.3702E-05	-2.9542E-01	-4.0378E-01
CCNA_02909	6.8252E-05	6.4987E-05	7.0633E-02	-1.7030E-01
CCNA_02910	1.3080E-04	2.3140E-04	-8.2305E-01	-7.4032E-01
CCNA_02911	1.1356E-04	5.9973E-05	9.2097E-01	3.7208E-01
CCNA_02912	1.1859E-04	9.7928E-05	2.7618E-01	-3.9192E-02
CCNA_02913	5.6752E-05	6.7142E-05	-2.4260E-01	-3.7009E-01

CCNA_02914	6.5861E-05	3.9728E-05	7.2907E-01	2.4968E-01
CCNA_02915	8.6277E-05	1.5022E-04	-8.0003E-01	-7.2564E-01
CCNA_02916	1.0859E-04	1.4771E-04	-4.4390E-01	-4.9849E-01
CCNA_02917	5.6789E-05	1.3782E-04	-1.2791E+00	-1.0312E+00
CCNA_02918	4.5663E-05	4.4327E-05	4.2708E-02	-1.8811E-01
CCNA_02919	4.8290E-05	4.3930E-05	1.3639E-01	-1.2836E-01
CCNA_02920	4.3837E-05	7.2346E-06	2.5977E+00	1.4415E+00
CCNA_02921	4.6588E-05	9.0337E-05	-9.5535E-01	-8.2471E-01
CCNA_02922	1.1738E-04	5.2449E-05	1.1621E+00	5.2587E-01
CCNA_02923	8.7437E-05	1.3489E-04	-6.2545E-01	-6.1429E-01
CCNA_02924	2.3971E-04	3.1101E-04	-3.7567E-01	-4.5497E-01
CCNA_02925	1.9371E-04	3.0690E-04	-6.6386E-01	-6.3879E-01
CCNA_02926	1.0731E-04	1.3885E-04	-3.7174E-01	-4.5246E-01
CCNA_02927	7.1623E-05	1.8198E-05	1.9761E+00	1.0451E+00
CCNA_02928	2.9793E-05	2.5135E-05	2.4506E-01	-5.9045E-02
CCNA_02929	5.0149E-05	2.1389E-05	1.2289E+00	5.6851E-01
CCNA_02930	6.0789E-05	5.7272E-05	8.5879E-02	-1.6057E-01
CCNA_02931	1.2022E-04	1.3244E-04	-1.3971E-01	-3.0446E-01
CCNA_02932	9.7759E-05	1.4602E-05	2.7423E+00	1.5338E+00
CCNA_02933	9.5322E-05	1.5212E-04	-6.7432E-01	-6.4546E-01
CCNA_02934	6.6694E-05	1.0725E-04	-6.8538E-01	-6.5252E-01
CCNA_02935	9.7412E-05	5.8507E-05	7.3536E-01	2.5369E-01
CCNA_02936	1.0665E-04	1.1415E-04	-9.8194E-02	-2.7798E-01
CCNA_02937	7.6908E-05	9.2301E-05	-2.6325E-01	-3.8326E-01
CCNA_02938	8.8982E-05	1.0304E-04	-2.1168E-01	-3.5037E-01
CCNA_02939	1.0872E-04	8.8887E-05	2.9053E-01	-3.0038E-02
CCNA_02940	7.0199E-05	1.3489E-04	-9.4224E-01	-8.1635E-01
CCNA_02941	3.5450E-04	1.6704E-04	1.0855E+00	4.7704E-01
CCNA_02942	7.7912E-05	1.2092E-04	-6.3411E-01	-6.1981E-01
CCNA_02943	1.8664E-04	6.3098E-05	1.5644E+00	7.8251E-01
CCNA_02944	2.0087E-04	1.4567E-04	4.6352E-01	8.0304E-02
CCNA_02945	1.0419E-04	9.8898E-05	7.5097E-02	-1.6745E-01
CCNA_02946	1.0436E-04	9.9080E-05	7.4807E-02	-1.6764E-01
CCNA_02947	5.0075E-05	4.0598E-05	3.0250E-01	-2.2404E-02
CCNA_02948	4.2149E-05	6.5352E-05	-6.3276E-01	-6.1895E-01
CCNA_02949	1.5231E-04	9.8052E-05	6.3534E-01	1.8990E-01
CCNA_02950	6.6990E-05	7.4327E-05	-1.5000E-01	-3.1103E-01
CCNA_02951	5.6673E-05	7.5536E-05	-4.1455E-01	-4.7977E-01
CCNA_02952	1.3732E-04	1.1962E-04	1.9897E-01	-8.8442E-02
CCNA_02953	7.4744E-05	1.3490E-04	-8.5182E-01	-7.5868E-01
CCNA_02954	5.4958E-05	1.0637E-04	-9.5272E-01	-8.2304E-01
CCNA_02955	4.8475E-05	1.1467E-04	-1.2421E+00	-1.0076E+00
CCNA_02956	5.3409E-05	6.6620E-05	-3.1893E-01	-4.1878E-01
CCNA_02957	8.0117E-05	1.3421E-04	-7.4430E-01	-6.9009E-01
CCNA_02958	7.1609E-05	1.6243E-04	-1.1815E+00	-9.6899E-01
CCNA_02959	7.3690E-05	1.6243E-04	-1.1402E+00	-9.4264E-01
CCNA_02960	1.0198E-04	2.9436E-05	1.7923E+00	9.2786E-01
CCNA_02961	2.8415E-04	1.1543E-04	1.2995E+00	6.1355E-01
CCNA_02962	3.5967E-04	1.6214E-04	1.1493E+00	5.1774E-01
CCNA_02963	2.1952E-04	1.3258E-04	7.2739E-01	2.4861E-01
CCNA_02964	2.3341E-04	1.3822E-04	7.5586E-01	2.6676E-01
CCNA_02965	8.2591E-05	1.3070E-04	-6.6224E-01	-6.3776E-01
CCNA_02966	1.4112E-04	1.5271E-04	-1.1390E-01	-2.8800E-01

CCNA_02967	1.6849E-04	1.2053E-04	4.8328E-01	9.2905E-02
CCNA_02968	5.9545E-05	6.1482E-05	-4.6262E-02	-2.4486E-01
CCNA_02969	8.5916E-05	2.9344E-05	1.5495E+00	7.7299E-01
CCNA_02970	4.8072E-05	9.4820E-05	-9.7998E-01	-8.4042E-01
CCNA_02971	7.9244E-05	7.6904E-05	4.3167E-02	-1.8782E-01
CCNA_02972	1.6052E-04	1.5340E-04	6.5419E-02	-1.7362E-01
CCNA_02973	1.3186E-04	1.1951E-04	1.4187E-01	-1.2486E-01
CCNA_02974	1.3040E-04	1.6243E-04	-3.1681E-01	-4.1743E-01
CCNA_02975	9.4813E-05	5.7164E-05	7.2984E-01	2.5017E-01
CCNA_02976	7.8485E-05	2.2201E-05	1.8213E+00	9.4638E-01
CCNA_02977	7.8587E-05	3.0811E-05	1.3505E+00	6.4607E-01
CCNA_02978	1.2333E-04	6.2683E-05	9.7629E-01	4.0737E-01
CCNA_02979	7.2238E-05	6.9429E-05	5.7142E-02	-1.7890E-01
CCNA_02980	3.7404E-04	1.0744E-04	1.7995E+00	9.3246E-01
CCNA_02981	3.8541E-04	4.9001E-05	2.9753E+00	1.6824E+00
CCNA_02982	6.7614E-05	1.2737E-05	2.4074E+00	1.3202E+00
CCNA_02983	7.8069E-05	1.6226E-04	-1.0555E+00	-8.8858E-01
CCNA_02984	5.8042E-05	4.5852E-05	3.3996E-01	1.4879E-03
CCNA_02985	6.0340E-05	1.0486E-04	-7.9734E-01	-7.2393E-01
CCNA_02986	1.6331E-04	2.2491E-05	2.8597E+00	1.6087E+00
CCNA_02987	2.5542E-04	1.4861E-04	7.8128E-01	2.8298E-01
CCNA_02988	1.2979E-04	6.6296E-08	1.0765E+01	6.6511E+00
CCNA_02989	5.4592E-05	1.1521E-04	-1.0774E+00	-9.0258E-01
CCNA_02990	0.0000E+00	0.0000E+00	-8.4168E-01	-7.5221E-01
CCNA_02991	0.0000E+00	0.0000E+00	-8.4168E-01	-7.5221E-01
CCNA_02992	6.3443E-05	9.5931E-05	-5.9656E-01	-5.9586E-01
CCNA_02993	4.3860E-05	2.8665E-05	6.1335E-01	1.7587E-01
CCNA_02994	2.6332E-04	1.7553E-04	5.8508E-01	1.5783E-01
CCNA_02995	1.1055E-04	1.2630E-04	-1.9218E-01	-3.3793E-01
CCNA_02996	7.9521E-05	3.2651E-06	4.6026E+00	2.7204E+00
CCNA_02997	2.7656E-04	1.3895E-04	9.9297E-01	4.1801E-01
CCNA_02998	6.1603E-05	3.4822E-05	8.2275E-01	3.0943E-01
CCNA_02999	6.3716E-05	1.4935E-04	-1.2289E+00	-9.9922E-01
CCNA_03000	8.8959E-05	9.5583E-05	-1.0366E-01	-2.8147E-01
CCNA_03001	7.5766E-05	1.2016E-04	-6.6537E-01	-6.3975E-01
CCNA_03002	2.5758E-04	1.6084E-04	6.7930E-01	2.1794E-01
CCNA_03003	2.5192E-04	1.6243E-04	6.3312E-01	1.8848E-01
CCNA_03004	5.6863E-05	8.0069E-05	-4.9380E-01	-5.3032E-01
CCNA_03005	7.2072E-05	7.8337E-05	-1.2033E-01	-2.9211E-01
CCNA_03006	3.8101E-04	4.5546E-04	-2.5750E-01	-3.7959E-01
CCNA_03007	7.1762E-05	3.2245E-05	1.1539E+00	5.2063E-01
CCNA_03008	8.7192E-05	2.9295E-05	1.5732E+00	7.8812E-01
CCNA_03009	7.2770E-05	8.3848E-05	-2.0449E-01	-3.4579E-01
CCNA_03010	5.5189E-05	7.8495E-05	-5.0825E-01	-5.3954E-01
CCNA_03011	1.9173E-04	3.1930E-04	-7.3584E-01	-6.8470E-01
CCNA_03012	1.7941E-04	3.2455E-04	-8.5523E-01	-7.6085E-01
CCNA_03013	6.6125E-05	7.9663E-05	-2.6877E-01	-3.8679E-01
CCNA_03014	7.0675E-05	7.9274E-05	-1.6570E-01	-3.2104E-01
CCNA_03015	5.6132E-05	3.4980E-05	6.8209E-01	2.1971E-01
CCNA_03016	9.6505E-05	8.3848E-05	2.0275E-01	-8.6027E-02
CCNA_03017	3.0260E-05	8.8671E-07	5.0796E+00	3.0246E+00
CCNA_03018	1.5468E-05	5.4695E-07	4.8005E+00	2.8466E+00
CCNA_03019	8.2143E-05	1.0278E-04	-3.2333E-01	-4.2159E-01

CCNA_03020	9.0869E-05	5.1952E-05	8.0646E-01	2.9904E-01
CCNA_03021	7.3588E-05	1.4094E-04	-9.3751E-01	-8.1333E-01
CCNA_03022	6.3785E-05	4.0366E-05	6.5988E-01	2.0555E-01
CCNA_03023	4.3078E-05	8.5920E-05	-9.9602E-01	-8.5065E-01
CCNA_03024	1.0153E-04	8.9102E-05	1.8833E-01	-9.5228E-02
CCNA_03025	1.3041E-04	1.6227E-04	-3.1536E-01	-4.1650E-01
CCNA_03026	6.0405E-05	1.4403E-04	-1.2536E+00	-1.0149E+00
CCNA_03027	4.1580E-05	1.1850E-04	-1.5109E+00	-1.1791E+00
CCNA_03028	4.8988E-05	4.4684E-05	1.3254E-01	-1.3081E-01
CCNA_03029	4.6538E-04	2.8884E-04	6.8812E-01	2.2356E-01
CCNA_03030	4.6956E-04	2.9753E-04	6.5824E-01	2.0450E-01
CCNA_03031	5.8773E-05	1.5631E-04	-1.4112E+00	-1.1154E+00
CCNA_03032	1.2182E-04	1.6279E-04	-4.1831E-01	-4.8217E-01
CCNA_03033	1.0787E-04	1.0969E-04	-2.4131E-02	-2.3074E-01
CCNA_03034	7.4999E-05	5.3866E-05	4.7736E-01	8.9129E-02
CCNA_03035	8.4191E-05	9.7456E-05	-2.1112E-01	-3.5001E-01
CCNA_03036	7.2067E-05	7.9489E-05	-1.4148E-01	-3.0560E-01
CCNA_03037	8.3401E-05	1.1957E-04	-5.1979E-01	-5.4690E-01
CCNA_03038	7.3089E-05	1.6257E-04	-1.1533E+00	-9.5097E-01
CCNA_03039	1.1873E-04	1.5870E-04	-4.1857E-01	-4.8233E-01
CCNA_03040	8.5565E-05	4.9921E-05	7.7720E-01	2.8038E-01
CCNA_03041	2.2664E-04	1.6243E-04	4.8059E-01	9.1187E-02
CCNA_03042	8.3008E-05	1.1159E-04	-4.2696E-01	-4.8769E-01
CCNA_03043	1.4423E-04	7.9821E-05	8.5339E-01	3.2898E-01
CCNA_03044	1.4970E-04	1.4705E-04	2.5743E-02	-1.9893E-01
CCNA_03045	1.0218E-04	1.4895E-04	-5.4376E-01	-5.6218E-01
CCNA_03046	6.5126E-05	1.3342E-04	-1.0347E+00	-8.7531E-01
CCNA_03047	4.9945E-05	9.8682E-05	-9.8243E-01	-8.4199E-01
CCNA_03048	4.0637E-05	5.1380E-07	6.2825E+00	3.7919E+00
CCNA_03049	3.8713E-05	1.6640E-05	1.2176E+00	5.6128E-01
CCNA_03050	6.3420E-05	2.7853E-05	1.1868E+00	5.4163E-01
CCNA_03051	4.0544E-05	1.0267E-04	-1.3404E+00	-1.0703E+00
CCNA_03052	1.2923E-04	1.6181E-04	-3.2435E-01	-4.2224E-01
CCNA_03053	1.4868E-04	1.6243E-04	-1.2761E-01	-2.9675E-01
CCNA_03055	6.6241E-05	6.5302E-05	2.0506E-02	-2.0227E-01
CCNA_03054	7.7158E-05	1.3516E-04	-8.0880E-01	-7.3124E-01
CCNA_03056	6.3073E-05	1.0549E-04	-7.4208E-01	-6.8868E-01
CCNA_03057	7.3325E-05	1.0230E-04	-4.8051E-01	-5.2184E-01
CCNA_03058	7.0615E-05	1.6167E-04	-1.1950E+00	-9.7758E-01
CCNA_03059	1.7195E-04	1.5704E-04	1.3081E-01	-1.3192E-01
CCNA_03060	1.6268E-04	1.1844E-04	4.5785E-01	7.6684E-02
CCNA_03061	1.5992E-04	8.7735E-05	8.6599E-01	3.3702E-01
CCNA_03062	9.8435E-04	2.5735E-04	1.9354E+00	1.0191E+00
CCNA_03063	8.7336E-05	6.5285E-05	4.1971E-01	5.2354E-02
CCNA_03064	2.8951E-04	1.5538E-04	8.9775E-01	3.5727E-01
CCNA_03065	2.7448E-04	1.4087E-04	9.6228E-01	3.9843E-01
CCNA_03066	7.6113E-05	1.5368E-04	-1.0137E+00	-8.6191E-01
CCNA_03067	5.5069E-05	1.0217E-04	-8.9168E-01	-7.8410E-01
CCNA_03068	6.5251E-05	1.8323E-05	1.8318E+00	9.5306E-01
CCNA_03069	5.2710E-05	5.1860E-05	2.3350E-02	-2.0046E-01
CCNA_03070	6.2814E-05	1.2116E-04	-9.4781E-01	-8.1990E-01
CCNA_03071	1.0362E-04	2.5275E-05	2.0350E+00	1.0827E+00
CCNA_03072	1.1110E-04	8.4031E-05	4.0284E-01	4.1595E-02

CCNA_03073	5.9244E-05	6.9711E-05	-2.3476E-01	-3.6509E-01
CCNA_03074	4.8068E-05	5.5043E-05	-1.9556E-01	-3.4009E-01
CCNA_03075	5.0121E-05	1.2228E-04	-1.2867E+00	-1.0361E+00
CCNA_03076	7.7149E-05	1.5759E-04	-1.0305E+00	-8.7264E-01
CCNA_03077	4.6708E-05	8.7768E-05	-9.1001E-01	-7.9580E-01
CCNA_03078	7.4139E-05	3.8178E-05	9.5724E-01	3.9522E-01
CCNA_03079	1.1184E-04	9.3014E-05	2.6584E-01	-4.5790E-02
CCNA_03080	7.0194E-05	1.6071E-04	-1.1950E+00	-9.7759E-01
CCNA_03081	7.6654E-05	9.9030E-05	-3.6954E-01	-4.5106E-01
CCNA_03082	1.8178E-04	1.0303E-04	8.1904E-01	3.0706E-01
CCNA_03083	7.7079E-05	1.2532E-04	-7.0117E-01	-6.6259E-01
CCNA_03084	5.8879E-05	1.3069E-04	-1.1503E+00	-9.4904E-01
CCNA_03085	8.3211E-05	6.2377E-05	4.1566E-01	4.9773E-02
CCNA_03086	2.3343E-04	1.3473E-04	7.9282E-01	2.9034E-01
CCNA_03087	2.1740E-04	1.4549E-04	5.7943E-01	1.5423E-01
CCNA_03088	8.2873E-05	6.5161E-05	3.4680E-01	5.8503E-03
CCNA_03089	8.2485E-05	1.0888E-04	-4.0061E-01	-4.7088E-01
CCNA_03090	7.4780E-04	1.7683E-04	2.0802E+00	1.1115E+00
CCNA_03091	8.8881E-04	2.6641E-04	1.7382E+00	8.9333E-01
CCNA_03092	1.3249E-03	4.6271E-04	1.5177E+00	7.5268E-01
CCNA_03093	6.7928E-05	6.9255E-05	-2.7977E-02	-2.3320E-01
CCNA_03094	8.4487E-05	8.2133E-05	4.0707E-02	-1.8939E-01
CCNA_03095	7.2178E-05	1.4048E-04	-9.6075E-01	-8.2816E-01
CCNA_03096	8.7548E-05	8.4089E-05	5.8104E-02	-1.7829E-01
CCNA_03097	7.6760E-05	1.4443E-04	-9.1198E-01	-7.9705E-01
CCNA_03098	7.6589E-05	1.4910E-04	-9.6106E-01	-8.2836E-01
CCNA_03099	1.1267E-04	1.4947E-04	-4.0785E-01	-4.7550E-01
CCNA_03100	1.2199E-04	4.5098E-05	1.4355E+00	7.0024E-01
CCNA_03101	3.9758E-05	1.0553E-04	-1.4082E+00	-1.1136E+00
CCNA_03102	7.5637E-05	9.1274E-05	-2.7115E-01	-3.8830E-01
CCNA_03103	8.1352E-05	6.6230E-05	2.9659E-01	-2.6171E-02
CCNA_03104	6.3286E-05	6.9669E-05	-1.3871E-01	-3.0383E-01
CCNA_03105	3.3500E-04	2.3023E-04	5.4106E-01	1.2976E-01
CCNA_03106	2.2466E-04	2.2982E-04	-3.2772E-02	-2.3626E-01
CCNA_03107	3.7866E-04	8.8522E-05	2.0967E+00	1.1220E+00
CCNA_03108	2.2570E-04	1.5171E-04	5.7302E-01	1.5015E-01
CCNA_03109	2.2404E-04	1.4788E-04	5.9925E-01	1.6687E-01
CCNA_03110	1.9961E-04	1.6243E-04	2.9739E-01	-2.5665E-02
CCNA_03111	1.5561E-04	1.5774E-04	-1.9642E-02	-2.2788E-01
CCNA_03112	1.2692E-04	1.4127E-04	-1.5458E-01	-3.1395E-01
CCNA_03113	1.3942E-04	1.6243E-04	-2.2035E-01	-3.5590E-01
CCNA_03114	9.4235E-05	7.8238E-05	2.6831E-01	-4.4212E-02
CCNA_03115	1.4603E-04	1.6243E-04	-1.5355E-01	-3.1329E-01
CCNA_03116	5.4578E-05	2.5002E-05	1.1259E+00	5.0281E-01
CCNA_03117	3.5564E-05	6.3587E-05	-8.3830E-01	-7.5005E-01
CCNA_03118	8.0210E-05	2.4936E-05	1.6852E+00	8.5952E-01
CCNA_03119	1.3943E-04	1.4417E-04	-4.8252E-02	-2.4613E-01
CCNA_03120	1.2857E-04	1.3579E-04	-7.8835E-02	-2.6564E-01
CCNA_03121	5.2179E-05	1.6005E-04	-1.6169E+00	-1.2467E+00
CCNA_03122	8.1357E-05	1.6171E-04	-9.9110E-01	-8.4752E-01
CCNA_03123	7.3764E-05	1.6243E-04	-1.1388E+00	-9.4171E-01
CCNA_03124	1.0186E-04	1.0966E-04	-1.0659E-01	-2.8334E-01
CCNA_03125	6.7276E-05	7.8603E-05	-2.2453E-01	-3.5857E-01

CCNA_03126	6.7309E-05	7.1128E-05	-7.9689E-02	-2.6618E-01
CCNA_03127	9.8938E-05	4.8454E-05	1.0297E+00	4.4144E-01
CCNA_03128	4.6144E-05	1.0220E-04	-1.1472E+00	-9.4709E-01
CCNA_03129	2.4958E-04	1.2508E-04	9.9654E-01	4.2028E-01
CCNA_03130	5.0012E-04	2.7219E-04	8.7763E-01	3.4444E-01
CCNA_03131	7.1031E-05	1.5258E-04	-1.1030E+00	-9.1892E-01
CCNA_03132	1.0085E-04	5.3559E-05	9.1281E-01	3.6688E-01
CCNA_03133	8.4427E-05	1.6243E-04	-9.4400E-01	-8.1747E-01
CCNA_03134	8.2147E-05	1.6243E-04	-9.8349E-01	-8.4266E-01
CCNA_03135	1.0802E-04	1.6280E-04	-5.9181E-01	-5.9283E-01
CCNA_03136	7.7685E-05	6.5899E-05	2.3730E-01	-6.3994E-02
CCNA_03137	6.0086E-05	8.7279E-05	-5.3864E-01	-5.5892E-01
CCNA_03138	1.7594E-04	2.4098E-04	-4.5381E-01	-5.0481E-01
CCNA_03139	5.5036E-05	0.0000E+00	1.2697E+01	7.8836E+00
CCNA_03140	6.2093E-05	3.6579E-05	7.6319E-01	2.7144E-01
CCNA_03141	6.9907E-05	3.0215E-05	1.2099E+00	5.5637E-01
CCNA_03142	2.3556E-04	1.4022E-04	7.4839E-01	2.6200E-01
CCNA_03143	1.3604E-04	8.8680E-05	6.1729E-01	1.7838E-01
CCNA_03144	1.9616E-04	1.6417E-04	2.5683E-01	-5.1534E-02
CCNA_03145	2.0496E-04	2.0215E-04	1.9915E-02	-2.0265E-01
CCNA_03146	1.2832E-04	1.5633E-04	-2.8485E-01	-3.9704E-01
CCNA_03147	1.3212E-04	1.3203E-04	9.5491E-04	-2.1474E-01
CCNA_03148	1.0626E-04	1.0176E-04	6.2271E-02	-1.7563E-01
CCNA_03149	1.0203E-04	1.2687E-04	-3.1449E-01	-4.1595E-01
CCNA_03150	4.5876E-05	1.0505E-04	-1.1953E+00	-9.7777E-01
CCNA_03151	4.7004E-05	5.1073E-05	-1.1987E-01	-2.9181E-01
CCNA_03152	7.3547E-05	4.6399E-05	6.6439E-01	2.0843E-01
CCNA_03153	5.0898E-05	1.2211E-04	-1.2625E+00	-1.0206E+00
CCNA_03154	8.8598E-05	1.4638E-04	-7.2440E-01	-6.7740E-01
CCNA_03155	9.1262E-05	9.8972E-05	-1.1706E-01	-2.9002E-01
CCNA_03156	5.7233E-05	1.7162E-05	1.7370E+00	8.9258E-01
CCNA_03157	5.9864E-05	9.4796E-05	-6.6315E-01	-6.3834E-01
CCNA_03158	3.6433E-05	7.9058E-06	2.2029E+00	1.1898E+00
CCNA_03159	6.0756E-05	7.2727E-05	-2.5951E-01	-3.8088E-01
CCNA_03160	2.1549E-04	3.3273E-04	-6.2673E-01	-6.1511E-01
CCNA_03161	1.9896E-04	3.1482E-04	-6.6206E-01	-6.3764E-01
CCNA_03162	5.4810E-05	1.4634E-04	-1.4168E+00	-1.1190E+00
CCNA_03163	1.4149E-04	1.9031E-04	-4.2765E-01	-4.8812E-01
CCNA_03164	1.2927E-04	1.6243E-04	-3.2945E-01	-4.2549E-01
CCNA_03165	5.1957E-05	1.4389E-04	-1.4695E+00	-1.1527E+00
CCNA_03166	5.2918E-05	1.0316E-04	-9.6299E-01	-8.2959E-01
CCNA_03167	9.3051E-05	2.9842E-05	1.6404E+00	8.3094E-01
CCNA_03168	5.6497E-05	1.5616E-04	-1.4667E+00	-1.1509E+00
CCNA_03169	5.4861E-05	9.9088E-05	-8.5294E-01	-7.5939E-01
CCNA_03170	7.8014E-05	1.2454E-04	-6.7480E-01	-6.4577E-01
CCNA_03171	1.7833E-04	1.1031E-04	6.9292E-01	2.2662E-01
CCNA_03172	6.8280E-05	1.6116E-04	-1.2389E+00	-1.0056E+00
CCNA_03173	6.7128E-05	1.6293E-04	-1.2792E+00	-1.0313E+00
CCNA_03174	6.5288E-05	1.2494E-04	-9.3638E-01	-8.1262E-01
CCNA_03175	1.1679E-04	9.7157E-05	2.6547E-01	-4.6022E-02
CCNA_03176	1.2017E-04	1.6243E-04	-4.3476E-01	-4.9266E-01
CCNA_03177	9.3745E-05	1.2733E-04	-4.4179E-01	-4.9714E-01
CCNA_03178	9.0600E-05	1.4676E-04	-6.9591E-01	-6.5923E-01

CCNA_03179	8.8570E-05	1.6243E-04	-8.7489E-01	-7.7339E-01
CCNA_03180	1.1536E-04	1.4807E-04	-3.6021E-01	-4.4511E-01
CCNA_03181	1.0065E-04	1.2343E-04	-2.9430E-01	-4.0307E-01
CCNA_03182	8.2055E-05	2.4082E-05	1.7682E+00	9.1249E-01
CCNA_03183	5.2086E-05	2.1463E-06	4.5955E+00	2.7159E+00
CCNA_03184	1.4789E-04	8.3948E-05	8.1685E-01	3.0567E-01
CCNA_03185	1.5333E-04	1.6243E-04	-8.3206E-02	-2.6842E-01
CCNA_03186	9.4466E-05	1.6273E-04	-7.8464E-01	-7.1583E-01
CCNA_03187	9.1396E-05	1.0373E-04	-1.8266E-01	-3.3186E-01
CCNA_03188	5.7691E-05	3.3521E-05	7.8302E-01	2.8409E-01
CCNA_03189	5.5166E-05	1.3123E-04	-1.2502E+00	-1.0128E+00
CCNA_03190	7.0596E-05	6.2716E-05	1.7066E-01	-1.0650E-01
CCNA_03191	1.2587E-04	1.1367E-04	1.4713E-01	-1.2151E-01
CCNA_03192	7.5655E-05	1.5179E-04	-1.0046E+00	-8.5612E-01
CCNA_03193	8.0108E-05	1.0192E-04	-3.4748E-01	-4.3699E-01
CCNA_03194	1.0845E-04	7.4144E-05	5.4852E-01	1.3452E-01
CCNA_03195	1.0560E-04	7.6755E-05	4.6014E-01	7.8146E-02
CCNA_03196	6.5658E-05	6.8691E-05	-6.5233E-02	-2.5696E-01
CCNA_03197	4.9510E-05	1.3127E-05	1.9145E+00	1.0058E+00
CCNA_03198	2.1562E-04	8.4437E-05	1.3524E+00	6.4728E-01
CCNA_03199	9.4549E-05	1.7867E-05	2.4032E+00	1.3175E+00
CCNA_03200	8.3285E-05	9.8318E-05	-2.3943E-01	-3.6807E-01
CCNA_03201	2.1575E-04	2.2578E-04	-6.5600E-02	-2.5719E-01
CCNA_03202	2.1494E-04	1.5764E-04	4.4731E-01	6.9961E-02
CCNA_03203	1.9870E-04	1.6243E-04	2.9079E-01	-2.9873E-02
CCNA_03204	6.3295E-05	1.4839E-04	-1.2292E+00	-9.9938E-01
CCNA_03205	9.0554E-05	1.6243E-04	-8.4293E-01	-7.5301E-01
CCNA_03206	3.8371E-05	1.0525E-04	-1.4557E+00	-1.1439E+00
CCNA_03207	5.9808E-05	8.7171E-05	-5.4353E-01	-5.6204E-01
CCNA_03208	5.2290E-05	9.3329E-05	-8.3580E-01	-7.4846E-01
CCNA_03209	7.0185E-05	1.4829E-04	-1.0792E+00	-9.0368E-01
CCNA_03210	5.9915E-05	9.4365E-05	-6.5535E-01	-6.3336E-01
CCNA_03211	1.5295E-04	1.3285E-04	2.0319E-01	-8.5746E-02
CCNA_03212	1.2254E-04	1.2066E-04	2.2258E-02	-2.0115E-01
CCNA_03213	1.3433E-04	1.5869E-04	-2.4049E-01	-3.6874E-01
CCNA_03214	8.5778E-04	2.4979E-04	1.7799E+00	9.1992E-01
CCNA_03215	7.0350E-04	2.6067E-04	1.4323E+00	6.9822E-01
CCNA_03216	1.7166E-04	1.6080E-04	9.4244E-02	-1.5524E-01
CCNA_03217	9.4744E-05	2.6726E-05	1.8254E+00	9.4898E-01
CCNA_03218	1.0149E-04	8.6310E-05	2.3367E-01	-6.6304E-02
CCNA_03219	7.6793E-05	9.9047E-05	-3.6717E-01	-4.4955E-01
CCNA_03220	4.8932E-05	6.5385E-05	-4.1821E-01	-4.8210E-01
CCNA_03221	5.2757E-05	2.4248E-05	1.1211E+00	4.9975E-01
CCNA_03222	7.4370E-05	1.4183E-04	-9.3140E-01	-8.0944E-01
CCNA_03223	4.8803E-05	9.4365E-05	-9.5127E-01	-8.2211E-01
CCNA_03224	1.8531E-04	1.0361E-04	8.3867E-01	3.1959E-01
CCNA_03225	8.1338E-05	7.2081E-05	1.7424E-01	-1.0422E-01
CCNA_03226	6.7651E-05	1.0374E-04	-6.1677E-01	-6.0875E-01
CCNA_03227	2.1794E-04	7.9813E-05	1.4491E+00	7.0896E-01
CCNA_03228	8.5912E-05	1.6269E-04	-9.2121E-01	-8.0294E-01
CCNA_03229	5.6817E-05	1.0521E-04	-8.8892E-01	-7.8234E-01
CCNA_03230	1.1112E-04	1.2781E-04	-2.0196E-01	-3.4417E-01
CCNA_03231	9.7287E-05	1.6243E-04	-7.3947E-01	-6.8702E-01

CCNA_03232	1.1643E-04	1.1057E-04	7.4458E-02	-1.6786E-01
CCNA_03233	6.0433E-05	2.6162E-05	1.2075E+00	5.5484E-01
CCNA_03234	6.3924E-05	6.3429E-05	1.1126E-02	-2.0826E-01
CCNA_03235	6.5672E-05	6.6421E-05	-1.6438E-02	-2.2584E-01
CCNA_03236	4.9769E-05	8.9533E-05	-8.4716E-01	-7.5571E-01
CCNA_03237	5.7436E-05	1.5698E-04	-1.4505E+00	-1.1406E+00
CCNA_03238	3.7478E-05	4.6739E-06	3.0010E+00	1.6988E+00
CCNA_03239	7.3477E-05	1.1714E-04	-6.7284E-01	-6.4452E-01
CCNA_03240	8.4524E-05	5.4603E-05	6.3024E-01	1.8664E-01
CCNA_03241	5.3492E-05	1.0479E-04	-9.7010E-01	-8.3412E-01
CCNA_03242	7.5480E-05	7.6804E-05	-2.5171E-02	-2.3141E-01
CCNA_03243	7.5678E-05	1.3166E-04	-7.9892E-01	-7.2493E-01
CCNA_03244	9.9058E-05	1.2104E-04	-2.8918E-01	-3.9980E-01
CCNA_03245	9.2052E-05	1.6233E-04	-8.1838E-01	-7.3735E-01
CCNA_03246	1.9625E-04	7.3175E-05	1.4232E+00	6.9240E-01
CCNA_03247	1.9880E-04	4.3764E-05	2.1833E+00	1.1772E+00
CCNA_03248	4.8424E-05	5.8341E-06	3.0512E+00	1.7308E+00
CCNA_03249	7.1202E-05	4.9656E-05	5.1981E-01	1.1620E-01
CCNA_03250	6.4724E-05	2.4447E-05	1.4043E+00	6.8035E-01
CCNA_04004	6.2523E-05	7.2603E-05	-2.1570E-01	-3.5294E-01
CCNA_03252	6.1653E-05	3.8709E-05	6.7132E-01	2.1284E-01
CCNA_03253	6.7193E-05	1.3293E-04	-9.8430E-01	-8.4318E-01
CCNA_03254	7.1554E-05	5.3833E-05	4.1041E-01	4.6428E-02
CCNA_03255	1.1710E-04	6.2965E-05	8.9494E-01	3.5548E-01
CCNA_03256	1.2408E-04	1.1047E-04	1.6760E-01	-1.0845E-01
CCNA_03257	1.5465E-04	1.7949E-04	-2.1492E-01	-3.5244E-01
CCNA_03258	8.3609E-05	1.6235E-04	-9.5739E-01	-8.2602E-01
CCNA_03259	1.1448E-04	9.1812E-05	3.1826E-01	-1.2354E-02
CCNA_03260	2.8606E-04	1.6243E-04	8.1649E-01	3.0544E-01
CCNA_03261	7.9595E-05	5.1769E-05	6.2044E-01	1.8039E-01
CCNA_03262	1.7608E-04	5.1297E-05	1.7791E+00	9.1941E-01
CCNA_03263	1.3910E-04	1.4105E-04	-2.0095E-02	-2.2817E-01
CCNA_03264	1.2621E-04	8.8240E-05	5.1619E-01	1.1389E-01
CCNA_03265	1.0975E-04	1.6165E-04	-5.5866E-01	-5.7169E-01
CCNA_03266	1.9516E-04	1.0277E-04	9.2516E-01	3.7476E-01
CCNA_03267	2.0510E-04	1.5173E-04	4.3482E-01	6.1993E-02
CCNA_03268	8.6499E-05	1.6241E-04	-9.0888E-01	-7.9507E-01
CCNA_03269	9.3176E-05	9.3113E-05	9.1483E-04	-2.1477E-01
CCNA_03271	9.2515E-05	8.6500E-05	9.6914E-02	-1.5354E-01
CCNA_03270	7.6580E-05	1.6047E-04	-1.0673E+00	-8.9609E-01
CCNA_03272	1.2433E-04	1.5356E-04	-3.0460E-01	-4.0964E-01
CCNA_03274	8.1722E-05	1.0376E-04	-3.4452E-01	-4.3510E-01
CCNA_03275	6.7928E-05	5.5391E-05	2.9425E-01	-2.7664E-02
CCNA_03276	8.9824E-05	1.6243E-04	-8.5462E-01	-7.6046E-01
CCNA_03277	6.8381E-05	1.2930E-04	-9.1907E-01	-8.0157E-01
CCNA_03278	1.1759E-04	1.0051E-04	2.2645E-01	-7.0914E-02
CCNA_03279	1.4432E-04	1.3011E-04	1.4956E-01	-1.1995E-01
CCNA_03280	4.3665E-04	1.6338E-04	1.4182E+00	6.8924E-01
CCNA_03281	2.4058E-04	1.2543E-04	9.3953E-01	3.8392E-01
CCNA_03282	4.8641E-05	2.0842E-05	1.2223E+00	5.6425E-01
CCNA_03283	6.6610E-05	9.6884E-05	-5.4053E-01	-5.6013E-01
CCNA_03284	7.0416E-05	5.8714E-05	2.6210E-01	-4.8174E-02
CCNA_03285	6.8442E-05	1.1090E-04	-6.9629E-01	-6.5947E-01

CCNA_03286	8.7715E-05	1.0676E-04	-2.8354E-01	-3.9621E-01
CCNA_03287	1.1487E-04	5.4769E-05	1.0684E+00	4.6614E-01
CCNA_03288	9.5719E-05	9.2707E-05	4.6069E-02	-1.8597E-01
CCNA_03289	1.1492E-04	6.9595E-05	7.2351E-01	2.4613E-01
CCNA_03290	7.1965E-05	1.0026E-04	-4.7835E-01	-5.2047E-01
CCNA_03291	3.7140E-04	1.6243E-04	1.1931E+00	5.4567E-01
CCNA_03292	4.7106E-05	7.1451E-05	-6.0107E-01	-5.9874E-01
CCNA_03293	7.0328E-05	1.5179E-04	-1.1099E+00	-9.2331E-01
CCNA_03294	8.3627E-05	8.1014E-05	4.5730E-02	-1.8618E-01
CCNA_03295	1.3106E-04	8.7039E-05	5.9042E-01	1.6125E-01
CCNA_03296	1.8403E-04	1.3940E-04	4.0062E-01	4.0179E-02
CCNA_03297	2.3635E-04	1.0250E-04	1.2052E+00	5.5337E-01
CCNA_03298	7.4407E-05	9.9503E-05	-4.1933E-01	-4.8282E-01
CCNA_03299	6.5219E-05	1.4553E-04	-1.1579E+00	-9.5393E-01
CCNA_03300	5.8588E-05	8.6459E-05	-5.6144E-01	-5.7346E-01
CCNA_03301	6.4016E-05	9.4282E-05	-5.5856E-01	-5.7163E-01
CCNA_03302	8.4899E-05	0.0000E+00	1.3323E+01	8.2824E+00
CCNA_03303	8.9130E-05	0.0000E+00	1.3393E+01	8.3272E+00
CCNA_03304	9.4263E-05	1.3626E-04	-5.3157E-01	-5.5441E-01
CCNA_03305	1.2417E-04	1.1822E-04	7.0722E-02	-1.7024E-01
CCNA_03306	3.0187E-04	5.0500E-04	-7.4236E-01	-6.8886E-01
CCNA_03307	1.7651E-04	1.7507E-04	1.1785E-02	-2.0783E-01
CCNA_03308	3.5244E-04	6.5264E-04	-8.8890E-01	-7.8233E-01
CCNA_03309	1.0018E-04	1.6258E-04	-6.9850E-01	-6.6088E-01
CCNA_03310	9.3042E-05	6.1805E-05	5.9004E-01	1.6100E-01
CCNA_03311	3.1049E-04	3.5521E-04	-1.9414E-01	-3.3919E-01
CCNA_03312	4.8581E-04	6.0506E-04	-3.1668E-01	-4.1735E-01
CCNA_03313	1.6714E-04	2.5267E-05	2.7253E+00	1.5230E+00
CCNA_03314	1.4873E-04	7.2512E-05	1.0362E+00	4.4561E-01
CCNA_03315	1.3827E-04	8.4586E-05	7.0886E-01	2.3679E-01
CCNA_03316	1.6775E-04	1.2721E-04	3.9908E-01	3.9200E-02
CCNA_03317	4.9760E-05	2.9079E-05	7.7472E-01	2.7880E-01
CCNA_03318	4.5400E-05	3.0745E-05	5.6209E-01	1.4317E-01
CCNA_03319	8.8413E-05	3.5137E-05	1.3310E+00	6.3362E-01
CCNA_03320	9.3347E-05	1.8936E-05	2.3009E+00	1.2523E+00
CCNA_03321	6.7286E-05	1.6265E-04	-1.2734E+00	-1.0276E+00
CCNA_03322	4.5233E-05	1.3166E-04	-1.5413E+00	-1.1984E+00
CCNA_03323	8.5158E-05	1.4003E-04	-7.1749E-01	-6.7300E-01
CCNA_03324	1.6984E-04	1.1854E-04	5.1880E-01	1.1556E-01
CCNA_03325	5.2694E-04	1.2407E-04	2.0864E+00	1.1155E+00
CCNA_03326	4.4368E-04	1.4822E-04	1.5817E+00	7.9351E-01
CCNA_03327	8.8557E-05	1.5547E-04	-8.1200E-01	-7.3328E-01
CCNA_03328	9.4226E-05	1.6256E-04	-7.8677E-01	-7.1719E-01
CCNA_03329	9.0739E-05	1.0275E-04	-1.7940E-01	-3.2978E-01
CCNA_03330	6.2791E-05	1.2404E-04	-9.8217E-01	-8.4182E-01
CCNA_03331	1.6159E-04	7.8271E-05	1.0456E+00	4.5160E-01
CCNA_03332	1.0777E-04	1.6243E-04	-5.9184E-01	-5.9285E-01
CCNA_03333	8.6129E-05	1.6219E-04	-9.1315E-01	-7.9780E-01
CCNA_03334	2.7309E-04	1.6243E-04	7.4955E-01	2.6274E-01
CCNA_03335	6.0067E-05	1.0604E-04	-8.1997E-01	-7.3836E-01
CCNA_03336	8.5278E-05	1.5250E-04	-8.3855E-01	-7.5021E-01
CCNA_03337	1.1657E-04	1.1405E-04	3.1417E-02	-1.9531E-01
CCNA_03338	8.6698E-05	5.1338E-05	7.5580E-01	2.6673E-01

CCNA_03339	9.4240E-05	8.3417E-05	1.7591E-01	-1.0315E-01
CCNA_03340	8.7567E-05	6.1506E-05	5.0953E-01	1.0965E-01
CCNA_03341	1.1055E-04	1.2138E-04	-1.3483E-01	-3.0135E-01
CCNA_03342	1.0715E-04	8.5058E-05	3.3309E-01	-2.8904E-03
CCNA_03343	6.8608E-05	1.6243E-04	-1.2433E+00	-1.0084E+00
CCNA_03344	5.9512E-05	5.0327E-05	2.4173E-01	-6.1169E-02
CCNA_03345	8.1343E-05	9.1323E-05	-1.6702E-01	-3.2188E-01
CCNA_03346	2.4410E-04	1.2217E-04	9.9853E-01	4.2155E-01
CCNA_03347	2.7444E-04	1.6193E-04	7.6106E-01	2.7009E-01
CCNA_03348	2.3400E-04	1.6195E-04	5.3087E-01	1.2326E-01
CCNA_03349	1.1796E-04	1.1960E-04	-1.9931E-02	-2.2806E-01
CCNA_03350	1.0690E-04	4.5521E-05	1.2315E+00	5.7013E-01
CCNA_03351	1.2433E-04	1.2557E-04	-1.4267E-02	-2.2445E-01
CCNA_03352	6.0544E-05	1.0459E-04	-7.8871E-01	-7.1843E-01
CCNA_03353	1.1449E-04	1.5984E-04	-4.8139E-01	-5.2240E-01
CCNA_03354	1.8513E-04	1.6263E-04	1.8685E-01	-9.6169E-02
CCNA_03355	9.6057E-05	4.9416E-05	9.5875E-01	3.9618E-01
CCNA_03356	9.4572E-05	1.6243E-04	-7.8029E-01	-7.1306E-01
CCNA_03357	2.4695E-04	3.2477E-04	-3.9523E-01	-4.6744E-01
CCNA_03358	2.4870E-04	3.2485E-04	-3.8539E-01	-4.6117E-01
CCNA_03359	6.6111E-05	1.1385E-04	-7.8414E-01	-7.1551E-01
CCNA_03360	1.6209E-04	4.8562E-05	1.7387E+00	8.9368E-01
CCNA_03361	8.6910E-05	9.3552E-05	-1.0630E-01	-2.8315E-01
CCNA_03273	6.6079E-05	6.0471E-05	1.2785E-01	-1.3380E-01
CCNA_03362	7.5345E-05	1.0699E-04	-5.0596E-01	-5.3808E-01
CCNA_03363	8.6933E-05	5.2167E-05	7.3662E-01	2.5450E-01
CCNA_03364	6.4035E-05	1.6276E-04	-1.3458E+00	-1.0737E+00
CCNA_03365	7.8203E-05	8.0152E-05	-3.5582E-02	-2.3805E-01
CCNA_03366	6.0354E-05	7.7757E-05	-3.6557E-01	-4.4853E-01
CCNA_03367	6.5658E-05	6.2567E-06	3.3897E+00	1.9467E+00
CCNA_03368	7.3496E-05	1.1898E-04	-6.9496E-01	-6.5863E-01
CCNA_03369	1.4292E-04	1.3094E-04	1.2619E-01	-1.3486E-01
CCNA_03370	1.5080E-04	1.5544E-04	-4.3739E-02	-2.4325E-01
CCNA_03371	6.5302E-04	1.8154E-04	1.8468E+00	9.6262E-01
CCNA_03372	2.0838E-04	2.9668E-04	-5.0971E-01	-5.4047E-01
CCNA_03373	2.5912E-04	3.2537E-04	-3.2851E-01	-4.2489E-01
CCNA_03374	9.1289E-05	7.8984E-05	2.0881E-01	-8.2163E-02
CCNA_03375	9.4549E-05	8.3417E-05	1.8065E-01	-1.0013E-01
CCNA_03376	7.9211E-05	6.1904E-05	3.5556E-01	1.1437E-02
CCNA_03377	1.4850E-04	2.6726E-04	-8.4778E-01	-7.5610E-01
CCNA_03378	1.3055E-04	3.2286E-04	-1.3063E+00	-1.0486E+00
CCNA_03379	1.2425E-04	6.0645E-05	1.0346E+00	4.4456E-01
CCNA_03380	7.7297E-05	4.0764E-05	9.2291E-01	3.7332E-01
CCNA_03381	9.7213E-05	9.0147E-05	1.0881E-01	-1.4595E-01
CCNA_03382	7.5614E-05	1.2216E-04	-6.9206E-01	-6.5677E-01
CCNA_03383	2.4250E-04	4.9030E-04	-1.0157E+00	-8.6321E-01
CCNA_03384	2.3823E-04	4.8797E-04	-1.0345E+00	-8.7518E-01
CCNA_03385	8.8815E-05	1.1380E-04	-3.5762E-01	-4.4346E-01
CCNA_03386	1.1270E-04	6.7614E-05	7.3696E-01	2.5471E-01
CCNA_03387	8.2383E-05	9.9304E-05	-2.6953E-01	-3.8727E-01
CCNA_03388	5.8777E-05	3.1681E-05	8.9136E-01	3.5319E-01
CCNA_03389	6.6139E-05	1.5652E-04	-1.2427E+00	-1.0080E+00
CCNA_03390	6.0770E-05	9.4887E-05	-6.4286E-01	-6.2539E-01

CCNA_03391	8.5768E-05	8.0111E-05	9.8372E-02	-1.5261E-01
CCNA_03392	7.1050E-05	1.4016E-04	-9.8016E-01	-8.4054E-01
CCNA_03393	3.2008E-04	1.6939E-04	9.1806E-01	3.7023E-01
CCNA_03394	3.2384E-04	1.6243E-04	9.9545E-01	4.1959E-01
CCNA_03395	2.8779E-04	2.6330E-04	1.2827E-01	-1.3354E-01
CCNA_03396	1.5427E-04	1.5634E-04	-1.9252E-02	-2.2763E-01
CCNA_03397	9.3158E-05	1.6243E-04	-8.0204E-01	-7.2693E-01
CCNA_03398	1.2133E-04	1.6277E-04	-4.2398E-01	-4.8579E-01
CCNA_03399	2.0587E-04	1.6458E-04	3.2290E-01	-9.3921E-03
CCNA_03400	1.9160E-04	1.6243E-04	2.3824E-01	-6.3390E-02
CCNA_03401	3.0002E-04	2.7312E-04	1.3547E-01	-1.2895E-01
CCNA_03402	1.7070E-04	1.6412E-04	5.6702E-02	-1.7918E-01
CCNA_03403	1.3979E-04	1.6242E-04	-2.1646E-01	-3.5342E-01
CCNA_03404	1.5577E-04	1.6571E-04	-8.9271E-02	-2.7229E-01
CCNA_03405	7.0878E-04	3.2485E-04	1.1255E+00	5.0256E-01
CCNA_03406	7.3688E-04	3.4887E-04	1.0787E+00	4.7270E-01
CCNA_03407	8.8251E-05	1.3160E-04	-5.7647E-01	-5.8305E-01
CCNA_03408	2.1928E-04	7.5992E-05	1.5287E+00	7.5971E-01
CCNA_03409	6.7175E-05	1.4768E-04	-1.1364E+00	-9.4021E-01
CCNA_03410	9.4975E-05	3.2162E-05	1.5619E+00	7.8088E-01
CCNA_04013	8.3461E-05	1.6284E-05	2.3570E+00	1.2880E+00
CCNA_03412	8.3285E-05	8.0592E-05	4.7360E-02	-1.8514E-01
CCNA_03413	8.9481E-05	9.9378E-05	-1.5139E-01	-3.1191E-01
CCNA_03414	1.3872E-04	1.0292E-04	4.3062E-01	5.9314E-02
CCNA_03415	1.3634E-04	1.6175E-04	-2.4658E-01	-3.7263E-01
CCNA_03416	1.0537E-04	1.6243E-04	-6.2427E-01	-6.1354E-01
CCNA_03417	6.2916E-05	4.0656E-05	6.2976E-01	1.8633E-01
CCNA_03418	8.3045E-05	1.0405E-04	-3.2538E-01	-4.2290E-01
CCNA_03419	8.9375E-05	1.5681E-04	-8.1105E-01	-7.3268E-01
CCNA_03420	6.4155E-05	1.1741E-04	-8.7193E-01	-7.7150E-01
CCNA_03421	5.6558E-05	1.1902E-04	-1.0734E+00	-9.0000E-01
CCNA_03422	7.7505E-05	1.2231E-04	-6.5818E-01	-6.3517E-01
CCNA_03423	1.1604E-04	9.1961E-05	3.3548E-01	-1.3696E-03
CCNA_03424	1.1256E-04	1.0165E-04	1.4704E-01	-1.2156E-01
CCNA_03425	7.9627E-05	7.9730E-05	-1.9195E-03	-2.1658E-01
CCNA_03426	2.1698E-04	2.2566E-04	-5.6657E-02	-2.5149E-01
CCNA_03427	2.1722E-04	1.8687E-04	2.1708E-01	-7.6887E-02
CCNA_03428	1.2815E-04	2.2983E-04	-8.4272E-01	-7.5287E-01
CCNA_03429	4.1362E-04	6.5446E-04	-6.6201E-01	-6.3761E-01
CCNA_03430	4.4979E-04	7.1285E-04	-6.6434E-01	-6.3910E-01
CCNA_03431	4.4548E-04	6.9141E-04	-6.3420E-01	-6.1987E-01
CCNA_03432	1.6094E-04	1.6243E-04	-1.3312E-02	-2.2384E-01
CCNA_03433	1.4462E-04	1.2339E-04	2.2898E-01	-6.9297E-02
CCNA_03434	1.4499E-04	1.5272E-04	-7.4994E-02	-2.6319E-01
CCNA_03435	7.6381E-05	1.3936E-04	-8.6747E-01	-7.6866E-01
CCNA_03436	6.4617E-05	1.4568E-04	-1.1728E+00	-9.6339E-01
CCNA_03437	7.0661E-05	7.1824E-05	-2.3617E-02	-2.3042E-01
CCNA_03438	7.6168E-05	9.7099E-05	-3.5030E-01	-4.3879E-01
CCNA_03439	7.6663E-05	9.1315E-05	-2.5236E-01	-3.7632E-01
CCNA_03440	2.1067E-05	0.0000E+00	1.1312E+01	7.0000E+00
CCNA_03441	0.0000E+00	0.0000E+00	-8.4168E-01	-7.5221E-01
CCNA_03442	6.4493E-05	2.6643E-05	1.2750E+00	5.9792E-01
CCNA_03443	1.6423E-04	8.7404E-05	9.0986E-01	3.6500E-01

CCNA_03444	1.6236E-04	6.7233E-05	1.2718E+00	5.9588E-01
CCNA_03445	1.0082E-04	4.0880E-05	1.3021E+00	6.1521E-01
CCNA_03446	1.3521E-04	7.8329E-05	7.8747E-01	2.8693E-01
CCNA_03447	1.7751E-04	1.1054E-04	6.8322E-01	2.2043E-01
CCNA_03448	8.2064E-05	1.0717E-04	-3.8508E-01	-4.6097E-01
CCNA_03449	9.4845E-05	5.8001E-05	7.0936E-01	2.3711E-01
CCNA_03450	1.0834E-04	1.3435E-04	-3.1047E-01	-4.1338E-01
CCNA_03451	8.9879E-05	9.0553E-05	-1.0829E-02	-2.2226E-01
CCNA_03452	8.2171E-05	5.8067E-05	5.0077E-01	1.0406E-01
CCNA_03453	8.8131E-05	1.6276E-04	-8.8500E-01	-7.7984E-01
CCNA_03454	8.5500E-05	1.2150E-04	-5.0694E-01	-5.3870E-01
CCNA_03455	1.7474E-04	1.2094E-04	5.3081E-01	1.2322E-01
CCNA_03456	6.4567E-05	7.7558E-05	-2.6455E-01	-3.8409E-01
CCNA_03457	7.9096E-05	1.0565E-04	-4.1767E-01	-4.8176E-01
CCNA_03458	1.1512E-04	1.0423E-04	1.4333E-01	-1.2393E-01
CCNA_03459	1.1824E-04	9.1530E-05	3.6936E-01	2.0243E-02
CCNA_03460	9.0596E-05	1.1971E-04	-4.0211E-01	-4.7183E-01
CCNA_03461	8.6610E-05	1.6273E-04	-9.0990E-01	-7.9572E-01
CCNA_03462	7.3958E-05	1.4370E-04	-9.5825E-01	-8.2656E-01
CCNA_03463	5.7857E-05	1.6258E-04	-1.4906E+00	-1.1661E+00
CCNA_03464	8.7937E-05	8.3616E-05	7.2617E-02	-1.6903E-01
CCNA_03465	1.7865E-04	9.0669E-05	9.7839E-01	4.0871E-01
CCNA_03466	2.2668E-04	1.6243E-04	4.8085E-01	9.1356E-02
CCNA_03467	1.1410E-04	1.6243E-04	-5.0950E-01	-5.4033E-01
CCNA_03468	1.1411E-04	1.6243E-04	-5.0932E-01	-5.4022E-01
CCNA_03469	7.0786E-05	4.8985E-05	5.3098E-01	1.2333E-01
CCNA_04011	2.6733E-04	1.6863E-04	6.6471E-01	2.0863E-01
CCNA_03471	2.7295E-04	2.0546E-04	4.0975E-01	4.6006E-02
CCNA_03472	8.9310E-05	1.6243E-04	-8.6289E-01	-7.6574E-01
CCNA_03473	1.8372E-04	1.6243E-04	1.7766E-01	-1.0203E-01
CCNA_03474	1.9923E-04	2.0018E-04	-6.8683E-03	-2.1973E-01
CCNA_03475	2.0977E-04	1.7132E-04	2.9208E-01	-2.9049E-02
CCNA_03476	7.5100E-05	1.5694E-04	-1.0633E+00	-8.9358E-01
CCNA_03477	6.1861E-05	2.0022E-05	1.6270E+00	8.2242E-01
CCNA_03478	6.9685E-05	6.9064E-05	1.2841E-02	-2.0716E-01
CCNA_03479	1.0532E-04	1.5620E-04	-5.6867E-01	-5.7807E-01
CCNA_03480	1.1602E-04	1.6250E-04	-4.8610E-01	-5.2541E-01
CCNA_03481	8.3886E-05	7.6133E-05	1.3983E-01	-1.2616E-01
CCNA_03482	7.2858E-05	1.5692E-04	-1.1069E+00	-9.2138E-01
CCNA_03483	7.4393E-05	8.5497E-05	-2.0077E-01	-3.4341E-01
CCNA_03484	5.6188E-05	4.2430E-05	4.0502E-01	4.2985E-02
CCNA_03485	1.9825E-04	1.3449E-04	5.5979E-01	1.4171E-01
CCNA_03486	1.2573E-04	1.6139E-04	-3.6019E-01	-4.4510E-01
CCNA_03487	1.2148E-04	1.2629E-04	-5.6017E-02	-2.5108E-01
CCNA_03488	1.0140E-04	1.0044E-04	1.3652E-02	-2.0664E-01
CCNA_03489	6.0946E-05	6.5932E-05	-1.1352E-01	-2.8776E-01
CCNA_03490	5.2387E-05	3.4582E-05	5.9897E-01	1.6669E-01
CCNA_03491	6.3725E-05	1.0205E-04	-6.7931E-01	-6.4864E-01
CCNA_03492	6.0687E-05	1.4947E-04	-1.3003E+00	-1.0448E+00
CCNA_03493	8.8399E-05	1.6320E-04	-8.8450E-01	-7.7953E-01
CCNA_03494	1.1560E-04	6.8832E-05	7.4785E-01	2.6166E-01
CCNA_03495	2.2844E-04	1.5407E-04	5.6813E-01	1.4703E-01
CCNA_03496	6.7290E-05	8.9401E-05	-4.0992E-01	-4.7682E-01

CCNA_03497	6.6301E-05	5.4869E-05	2.7293E-01	-4.1268E-02
CCNA_03498	7.6686E-05	8.1569E-05	-8.9117E-02	-2.7219E-01
CCNA_03499	7.4726E-05	1.6031E-04	-1.1012E+00	-9.1774E-01
CCNA_03500	8.5398E-05	1.2345E-04	-5.3169E-01	-5.5449E-01
CCNA_03501	7.1752E-05	1.2416E-04	-7.9106E-01	-7.1993E-01
CCNA_03502	1.2271E-04	1.5918E-04	-3.7546E-01	-4.5483E-01
CCNA_03503	1.1212E-04	8.1917E-05	4.5273E-01	7.3417E-02
CCNA_03504	1.1334E-04	1.1420E-04	-1.1036E-02	-2.2239E-01
CCNA_03505	1.6828E-04	1.4399E-04	2.2489E-01	-7.1910E-02
CCNA_03506	1.5774E-04	1.6243E-04	-4.2285E-02	-2.4232E-01
CCNA_03507	1.8312E-04	1.6752E-04	1.2836E-01	-1.3348E-01
CCNA_03508	1.3423E-04	1.0585E-04	3.4265E-01	3.2079E-03
CCNA_03509	6.3868E-05	9.9768E-05	-6.4349E-01	-6.2580E-01
CCNA_03510	6.5084E-05	1.4387E-04	-1.1444E+00	-9.4528E-01
CCNA_03511	6.9205E-05	1.6110E-04	-1.2190E+00	-9.9288E-01
CCNA_03512	5.4694E-05	1.9657E-05	1.4759E+00	7.2602E-01
CCNA_03513	2.6760E-04	1.0773E-04	1.3126E+00	6.2186E-01
CCNA_03514	1.4990E-04	6.8194E-05	1.1361E+00	5.0930E-01
CCNA_03515	1.1239E-04	1.2649E-04	-1.7044E-01	-3.2407E-01
CCNA_03516	8.5705E-04	6.3835E-05	3.7468E+00	2.1745E+00
CCNA_03517	8.5916E-05	8.6260E-05	-5.8216E-03	-2.1906E-01
CCNA_03518	1.1955E-03	2.9674E-04	2.0103E+00	1.0669E+00
CCNA_03519	8.3765E-04	1.7290E-04	2.2763E+00	1.2366E+00
CCNA_03520	8.7197E-05	1.6013E-04	-8.7690E-01	-7.7467E-01
CCNA_03521	7.3551E-05	1.1445E-04	-6.3794E-01	-6.2226E-01
CCNA_03522	1.1736E-04	7.6431E-05	6.1855E-01	1.7918E-01
CCNA_03523	6.4169E-05	1.4661E-04	-1.1921E+00	-9.7570E-01
CCNA_03524	1.6835E-04	1.0718E-04	6.5137E-01	2.0012E-01
CCNA_03525	1.3668E-04	1.6194E-04	-2.4471E-01	-3.7144E-01
CCNA_03526	1.7024E-04	8.1279E-05	1.0665E+00	4.6489E-01
CCNA_03527	8.3516E-05	1.0233E-04	-2.9312E-01	-4.0231E-01
CCNA_03528	1.5639E-04	1.6243E-04	-5.4688E-02	-2.5023E-01
CCNA_03529	1.6380E-04	1.5438E-04	8.5420E-02	-1.6087E-01
CCNA_03530	1.0705E-04	8.6840E-05	3.0182E-01	-2.2839E-02
CCNA_03531	1.3930E-04	1.6244E-04	-2.2179E-01	-3.5682E-01
CCNA_03532	1.2046E-04	1.5252E-04	-3.4046E-01	-4.3251E-01
CCNA_03533	6.6842E-05	1.4243E-04	-1.0914E+00	-9.1150E-01
CCNA_03534	1.8625E-04	2.0337E-04	-1.2690E-01	-2.9630E-01
CCNA_03535	8.7525E-05	5.2971E-05	7.2435E-01	2.4667E-01
CCNA_03536	8.5958E-05	1.6272E-04	-9.2065E-01	-8.0258E-01
CCNA_03537	8.7622E-05	1.5503E-04	-8.2321E-01	-7.4043E-01
CCNA_03538	1.3292E-04	9.7597E-05	4.4564E-01	6.8895E-02
CCNA_03539	1.0556E-04	1.5963E-04	-5.9658E-01	-5.9588E-01
CCNA_03540	1.1389E-04	5.7214E-05	9.9308E-01	4.1808E-01
CCNA_03541	8.0081E-05	7.8851E-05	2.2249E-02	-2.0116E-01
CCNA_03542	8.7955E-05	1.6221E-04	-8.8302E-01	-7.7858E-01
CCNA_03543	9.6191E-05	1.1299E-04	-2.3231E-01	-3.6353E-01
CCNA_03544	7.3417E-05	6.9363E-05	8.1878E-02	-1.6313E-01
CCNA_03545	3.7122E-04	1.7617E-04	1.0752E+00	4.7047E-01
CCNA_03546	1.0453E-04	1.6189E-04	-6.3105E-01	-6.1786E-01
CCNA_03547	1.5226E-04	1.4298E-04	9.0748E-02	-1.5747E-01
CCNA_03548	1.3290E-04	1.2990E-04	3.2873E-02	-1.9438E-01
CCNA_03549	5.9757E-05	0.0000E+00	1.2816E+01	7.9593E+00

CCNA_03550	2.5926E-04	1.8785E-04	4.6475E-01	8.1086E-02
CCNA_03551	1.1116E-04	1.1142E-04	-3.3598E-03	-2.1749E-01
CCNA_03552	2.4004E-04	1.6946E-04	5.0226E-01	1.0501E-01
CCNA_03553	7.6723E-05	6.1390E-05	3.2154E-01	-1.0257E-02
CCNA_03554	1.5806E-04	1.1008E-04	5.2187E-01	1.1752E-01
CCNA_03555	1.5369E-04	7.3862E-05	1.0570E+00	4.5885E-01
CCNA_03556	6.6643E-05	4.4833E-05	5.7172E-01	1.4932E-01
CCNA_03557	1.4605E-04	3.0558E-04	-1.0650E+00	-8.9468E-01
CCNA_03558	8.5366E-05	3.7830E-05	1.1739E+00	5.3340E-01
CCNA_03559	1.0523E-04	1.6126E-04	-6.1582E-01	-6.0815E-01
CCNA_03560	8.2582E-05	5.4628E-05	5.9605E-01	1.6483E-01
CCNA_03561	8.8903E-05	1.4390E-04	-6.9473E-01	-6.5848E-01
CCNA_03562	8.8131E-05	3.8535E-05	1.1933E+00	5.4576E-01
CCNA_03563	1.8068E-04	2.7994E-04	-6.3168E-01	-6.1827E-01
CCNA_03564	9.6117E-05	4.9681E-05	9.5193E-01	3.9183E-01
CCNA_03565	2.5697E-04	2.0453E-04	3.2922E-01	-5.3617E-03
CCNA_03566	2.6213E-04	3.1405E-04	-2.6073E-01	-3.8166E-01
CCNA_03567	4.0974E-04	8.0597E-04	-9.7603E-01	-8.3790E-01
CCNA_03568	1.7853E-04	1.6276E-04	1.3343E-01	-1.3025E-01
CCNA_03569	5.5605E-05	1.2383E-04	-1.1550E+00	-9.5205E-01
CCNA_03570	4.2422E-05	3.0944E-05	4.5492E-01	7.4816E-02
CCNA_03571	5.5230E-05	1.9698E-05	1.4869E+00	7.3306E-01
CCNA_03572	8.3553E-05	1.1302E-04	-4.3582E-01	-4.9334E-01
CCNA_03573	1.0918E-04	9.1158E-05	2.6021E-01	-4.9381E-02
CCNA_03574	2.0412E-04	2.3244E-04	-1.8741E-01	-3.3489E-01
CCNA_03575	2.0998E-04	2.0360E-04	4.4464E-02	-1.8699E-01
CCNA_03576	6.1644E-05	9.4406E-05	-6.1493E-01	-6.0758E-01
CCNA_03577	1.3323E-04	1.6122E-04	-2.7512E-01	-3.9084E-01
CCNA_03578	1.3511E-04	1.0921E-04	3.0704E-01	-1.9511E-02
CCNA_03579	7.5452E-05	1.6243E-04	-1.1061E+00	-9.2090E-01
CCNA_03580	1.2551E-04	1.6243E-04	-3.7197E-01	-4.5261E-01
CCNA_03581	1.3549E-04	1.6243E-04	-2.6166E-01	-3.8225E-01
CCNA_03582	1.1650E-04	1.6243E-04	-4.7941E-01	-5.2114E-01
CCNA_03583	1.6912E-04	2.5269E-04	-5.7934E-01	-5.8488E-01
CCNA_03584	1.3627E-04	1.6258E-04	-2.5468E-01	-3.7780E-01
CCNA_03585	9.3176E-05	1.6272E-04	-8.0433E-01	-7.2839E-01
CCNA_03586	1.4039E-04	1.2605E-04	1.5533E-01	-1.1628E-01
CCNA_03587	6.6832E-05	9.3785E-05	-4.8883E-01	-5.2715E-01
CCNA_03588	7.1415E-05	4.7477E-05	5.8885E-01	1.6024E-01
CCNA_03589	1.4051E-04	9.0387E-05	6.3644E-01	1.9059E-01
CCNA_03590	2.1500E-04	1.2032E-04	8.3739E-01	3.1877E-01
CCNA_03591	2.1477E-04	1.2233E-04	8.1192E-01	3.0252E-01
CCNA_03592	6.7739E-05	2.5706E-05	1.3975E+00	6.7602E-01
CCNA_03593	1.2752E-04	1.8909E-04	-5.6833E-01	-5.7786E-01
CCNA_03594	1.3240E-04	2.2740E-04	-7.8028E-01	-7.1305E-01
CCNA_03595	7.2280E-05	7.7733E-05	-1.0499E-01	-2.8232E-01
CCNA_03596	1.0071E-04	1.5457E-04	-6.1808E-01	-6.0959E-01
CCNA_03597	4.7222E-04	1.9400E-04	1.2834E+00	6.0323E-01
CCNA_03598	4.5842E-04	1.6961E-04	1.4344E+00	6.9956E-01
CCNA_03599	7.0190E-05	1.6425E-04	-1.2265E+00	-9.9769E-01
CCNA_03600	8.5149E-05	9.6983E-05	-1.8780E-01	-3.3514E-01
CCNA_03601	1.4271E-04	1.6243E-04	-1.8668E-01	-3.3443E-01
CCNA_03602	6.1297E-05	1.2412E-04	-1.0178E+00	-8.6453E-01

CCNA_03603	1.0601E-04	1.3050E-04	-2.9996E-01	-4.0668E-01
CCNA_03604	9.6246E-05	1.4870E-04	-6.2764E-01	-6.1569E-01
CCNA_03605	9.6792E-05	9.5541E-05	1.8707E-02	-2.0342E-01
CCNA_03606	8.5740E-05	1.6243E-04	-9.2173E-01	-8.0327E-01
CCNA_03607	1.6829E-04	7.9995E-05	1.0729E+00	4.6899E-01
CCNA_03608	9.9571E-05	1.2571E-04	-3.3628E-01	-4.2985E-01
CCNA_03609	8.1268E-04	4.5455E-04	8.3822E-01	3.1930E-01
CCNA_03610	5.8851E-05	1.2501E-04	-1.0869E+00	-9.0861E-01
CCNA_03611	8.4723E-05	1.0099E-04	-2.5349E-01	-3.7704E-01
CCNA_03612	6.0419E-05	7.0291E-05	-2.1840E-01	-3.5466E-01
CCNA_03613	5.7223E-05	8.2862E-05	-5.3413E-01	-5.5604E-01
CCNA_03614	1.8898E-04	1.2680E-04	5.7561E-01	1.5179E-01
CCNA_03615	8.7738E-05	1.2052E-04	-4.5800E-01	-5.0749E-01
CCNA_03616	5.1295E-05	1.4917E-07	8.3479E+00	5.1093E+00
CCNA_03617	4.2015E-05	4.2968E-05	-3.2498E-02	-2.3608E-01
CCNA_03618	6.8247E-05	1.6243E-04	-1.2509E+00	-1.0132E+00
CCNA_03619	7.7260E-05	1.1221E-04	-5.3839E-01	-5.5876E-01
CCNA_03620	5.3108E-05	1.5667E-04	-1.5607E+00	-1.2108E+00
CCNA_03621	8.5995E-05	1.7399E-04	-1.0166E+00	-8.6381E-01
CCNA_03622	9.1479E-05	1.7316E-04	-9.2057E-01	-8.0253E-01
CCNA_03623	6.6134E-05	1.4741E-04	-1.1563E+00	-9.5292E-01
CCNA_03624	7.7325E-05	1.1650E-04	-5.9134E-01	-5.9253E-01
CCNA_03625	7.2585E-05	1.6204E-04	-1.1586E+00	-9.5438E-01
CCNA_03626	6.2759E-05	1.1545E-04	-8.7934E-01	-7.7623E-01
CCNA_03627	5.6442E-05	7.6307E-05	-4.3509E-01	-4.9287E-01
CCNA_03628	1.3981E-04	1.5515E-04	-1.5027E-01	-3.1120E-01
CCNA_03629	2.1911E-04	1.4022E-05	3.9651E+00	2.3138E+00
CCNA_03630	2.2174E-04	9.8765E-05	1.1667E+00	5.2882E-01
CCNA_03631	2.2282E-04	1.0074E-04	1.1452E+00	5.1509E-01
CCNA_03632	1.5728E-04	4.8272E-05	1.7039E+00	8.7145E-01
CCNA_03633	9.0707E-05	8.7296E-05	5.5234E-02	-1.8012E-01
CCNA_03634	2.8008E-05	3.4449E-05	-2.9873E-01	-4.0589E-01
CCNA_03635	3.7215E-05	1.4668E-05	1.3426E+00	6.4099E-01
CCNA_03636	1.0408E-04	1.4329E-04	-4.6122E-01	-5.0954E-01
CCNA_03637	1.1401E-04	1.7608E-04	-6.2708E-01	-6.1533E-01
CCNA_03638	3.5209E-04	3.8064E-04	-1.1250E-01	-2.8711E-01
CCNA_03639	1.0767E-04	1.5063E-04	-4.8441E-01	-5.2433E-01
CCNA_03640	1.0685E-04	8.9624E-05	2.5355E-01	-5.3629E-02
CCNA_03641	1.2247E-04	1.4258E-04	-2.1936E-01	-3.5527E-01
CCNA_03642	7.9035E-05	6.0089E-05	3.9528E-01	3.6772E-02
CCNA_03643	8.6004E-05	1.6163E-04	-9.1022E-01	-7.9593E-01
CCNA_03644	1.3214E-04	1.6243E-04	-2.9775E-01	-4.0527E-01
CCNA_03645	1.1976E-04	1.6243E-04	-4.3960E-01	-4.9575E-01
CCNA_03646	1.1349E-04	1.2654E-04	-1.5705E-01	-3.1552E-01
CCNA_03647	9.8387E-05	6.4084E-05	6.1840E-01	1.7909E-01
CCNA_03648	7.8208E-05	1.4936E-04	-9.3338E-01	-8.1070E-01
CCNA_03649	9.7740E-05	1.3525E-04	-4.6866E-01	-5.1428E-01
CCNA_03650	8.4848E-05	6.0835E-05	4.7986E-01	9.0720E-02
CCNA_03651	9.6001E-05	1.2400E-04	-3.6923E-01	-4.5086E-01
CCNA_03652	6.3984E-05	1.4855E-04	-1.2152E+00	-9.9044E-01
CCNA_03653	1.0780E-04	1.5565E-04	-5.2991E-01	-5.5335E-01
CCNA_03654	6.4835E-05	4.8736E-05	4.1163E-01	4.7206E-02
CCNA_03655	8.2060E-05	5.0601E-05	6.9736E-01	2.2945E-01

CCNA_03656	4.9285E-04	1.6243E-04	1.6013E+00	8.0603E-01
CCNA_03657	1.3209E-04	8.8481E-05	5.7803E-01	1.5334E-01
CCNA_03658	1.1790E-04	1.6243E-04	-4.6222E-01	-5.1018E-01
CCNA_03659	1.0249E-04	1.5769E-04	-6.2167E-01	-6.1188E-01
CCNA_03660	1.3621E-04	3.9985E-05	1.7680E+00	9.1237E-01
CCNA_03661	7.0342E-05	1.3631E-04	-9.5437E-01	-8.2409E-01
CCNA_03662	6.3448E-05	1.0992E-04	-7.9281E-01	-7.2104E-01
CCNA_03663	1.8296E-04	1.6243E-04	1.7169E-01	-1.0584E-01
CCNA_03664	1.8094E-04	1.6243E-04	1.5571E-01	-1.1604E-01
CCNA_03665	8.6212E-05	6.5940E-05	3.8663E-01	3.1258E-02
CCNA_03666	8.9893E-05	1.4295E-04	-6.6926E-01	-6.4223E-01
CCNA_03667	9.0499E-05	6.1995E-05	5.4562E-01	1.3266E-01
CCNA_03668	7.4985E-05	1.2091E-04	-6.8925E-01	-6.5499E-01
CCNA_03669	1.0709E-04	1.4036E-04	-3.9025E-01	-4.6427E-01
CCNA_03670	1.0559E-04	1.6275E-04	-6.2423E-01	-6.1351E-01
CCNA_03671	6.6689E-05	2.4182E-05	1.4631E+00	7.1790E-01
CCNA_03672	8.4677E-05	1.4864E-04	-8.1175E-01	-7.3312E-01
CCNA_03673	1.0333E-04	1.1460E-04	-1.4940E-01	-3.1064E-01
CCNA_03674	3.9947E-04	2.0193E-04	9.8420E-01	4.1241E-01
CCNA_03675	3.9201E-04	2.1322E-04	8.7852E-01	3.4501E-01
CCNA_03676	1.0785E-04	7.5553E-05	5.1335E-01	1.1208E-01
CCNA_03677	6.7901E-05	1.2870E-04	-9.2249E-01	-8.0375E-01
CCNA_03678	6.5510E-05	3.6786E-05	8.3232E-01	3.1554E-01
CCNA_03679	8.4113E-05	1.6243E-04	-9.4938E-01	-8.2091E-01
CCNA_03680	7.2996E-05	9.4762E-05	-3.7653E-01	-4.5552E-01
CCNA_03681	2.0262E-04	1.2758E-04	6.6735E-01	2.1031E-01
CCNA_03682	2.4690E-04	1.2639E-04	9.6605E-01	4.0084E-01
CCNA_03683	7.8046E-05	7.9373E-05	-2.4397E-02	-2.3091E-01
CCNA_03684	9.1442E-05	1.1425E-04	-3.2124E-01	-4.2025E-01
CCNA_03685	6.0761E-05	1.5982E-04	-1.3952E+00	-1.1053E+00
CCNA_03686	9.9895E-05	1.3796E-04	-4.6582E-01	-5.1247E-01
CCNA_03687	1.6127E-04	1.8813E-04	-2.2229E-01	-3.5714E-01
CCNA_03688	1.0855E-03	4.8761E-04	1.1545E+00	5.2105E-01
CCNA_03689	1.0574E-03	4.9120E-04	1.1061E+00	4.9018E-01
CCNA_03690	2.0521E-04	5.5490E-05	1.8866E+00	9.8801E-01
CCNA_03691	1.6202E-04	1.1481E-04	4.9688E-01	1.0158E-01
CCNA_03692	1.8033E-04	1.6243E-04	1.5083E-01	-1.1915E-01
CCNA_03693	6.1024E-04	2.8578E-04	1.0944E+00	4.8273E-01
CCNA_03694	6.1603E-04	2.8362E-04	1.1190E+00	4.9838E-01
CCNA_03695	1.0483E-04	1.6506E-04	-6.5491E-01	-6.3308E-01
CCNA_03696	7.3755E-05	8.7238E-05	-2.4226E-01	-3.6988E-01
CCNA_03697	8.6957E-05	1.6074E-04	-8.8632E-01	-7.8068E-01
CCNA_03698	6.7073E-05	1.5955E-04	-1.2502E+00	-1.0128E+00
CCNA_03699	7.9988E-05	1.6243E-04	-1.0219E+00	-8.6717E-01
CCNA_03700	1.3266E-04	1.3179E-04	9.4786E-03	-2.0931E-01
CCNA_03701	1.2667E-04	1.2394E-04	3.1359E-02	-1.9535E-01
CCNA_03702	2.1154E-04	3.2575E-04	-6.2280E-01	-6.1260E-01
CCNA_03703	5.4454E-05	6.1904E-05	-1.8508E-01	-3.3340E-01
CCNA_03704	1.4902E-04	2.4684E-04	-7.2805E-01	-6.7973E-01
CCNA_03705	1.1657E-04	1.9665E-04	-7.5440E-01	-6.9654E-01
CCNA_03706	3.0397E-04	2.0956E-04	5.3649E-01	1.2685E-01
CCNA_03707	8.4853E-05	1.6243E-04	-9.3675E-01	-8.1285E-01
CCNA_03708	0.0000E+00	0.0000E+00	-8.4168E-01	-7.5221E-01

CCNA_03709	6.7729E-05	0.0000E+00	1.2997E+01	8.0745E+00
CCNA_03710	1.2016E-04	1.3013E-04	-1.1508E-01	-2.8875E-01
CCNA_03711	1.6976E-04	1.1654E-04	5.4260E-01	1.3074E-01
CCNA_03712	8.6836E-05	1.5309E-04	-8.1806E-01	-7.3714E-01
CCNA_03713	1.2261E-04	1.6243E-04	-4.0574E-01	-4.7415E-01
CCNA_03714	1.7600E-04	1.3803E-04	3.5054E-01	8.2396E-03
CCNA_03715	2.1810E-04	1.6243E-04	4.2514E-01	5.5821E-02
CCNA_03716	1.5168E-04	1.4082E-04	1.0708E-01	-1.4705E-01
CCNA_03717	5.5347E-04	3.5907E-04	6.2423E-01	1.8281E-01
CCNA_03718	5.4332E-04	5.7276E-04	-7.6135E-02	-2.6391E-01
CCNA_03719	9.1900E-05	1.6243E-04	-8.2165E-01	-7.3944E-01
CCNA_03720	7.7542E-05	7.3158E-05	8.3881E-02	-1.6185E-01
CCNA_03721	9.2792E-05	1.5744E-04	-7.6271E-01	-7.0184E-01
CCNA_03722	7.3228E-05	1.4685E-04	-1.0038E+00	-8.5564E-01
CCNA_03723	7.9026E-05	7.6473E-05	4.7311E-02	-1.8517E-01
CCNA_03724	8.9148E-05	7.0954E-05	3.2924E-01	-5.3503E-03
CCNA_03725	1.2862E-04	1.6243E-04	-3.3664E-01	-4.3007E-01
CCNA_03726	9.4730E-05	1.0622E-04	-1.6525E-01	-3.2076E-01
CCNA_03727	9.9617E-05	1.1227E-04	-1.7258E-01	-3.2543E-01
CCNA_03728	1.2656E-04	1.3606E-04	-1.0440E-01	-2.8194E-01
CCNA_03729	1.1702E-04	1.3662E-04	-2.2347E-01	-3.5789E-01
CCNA_03730	6.3679E-05	4.6250E-05	4.6120E-01	7.8824E-02
CCNA_03731	1.0272E-04	8.6707E-05	2.4436E-01	-5.9492E-02
CCNA_03732	1.2734E-04	1.3278E-04	-6.0295E-02	-2.5381E-01
CCNA_03733	1.2387E-04	1.6159E-04	-3.8357E-01	-4.6001E-01
CCNA_03734	5.7262E-04	3.2485E-04	8.1778E-01	3.0626E-01
CCNA_03735	5.8608E-04	3.0985E-04	9.1950E-01	3.7114E-01
CCNA_03736	4.5819E-04	3.6282E-04	3.3671E-01	-5.8545E-04
CCNA_03737	1.5666E-04	1.7245E-04	-1.3852E-01	-3.0371E-01
CCNA_03738	8.9005E-05	8.4263E-05	7.8926E-02	-1.6501E-01
CCNA_03739	7.2802E-05	6.2791E-05	2.1333E-01	-7.9284E-02
CCNA_03740	2.7947E-04	1.6118E-04	7.9396E-01	2.9107E-01
CCNA_03741	1.6749E-04	1.0785E-04	6.3497E-01	1.8966E-01
CCNA_03742	1.8732E-04	9.3072E-05	1.0090E+00	4.2822E-01
CCNA_03743	1.1033E-04	1.6243E-04	-5.5795E-01	-5.7124E-01
CCNA_03744	8.5135E-05	1.4870E-04	-8.0462E-01	-7.2857E-01
CCNA_03745	2.6000E-04	1.8440E-04	4.9570E-01	1.0083E-01
CCNA_03746	2.4909E-04	2.0675E-04	2.6879E-01	-4.3904E-02
CCNA_03747	1.4074E-04	1.1096E-04	3.4294E-01	3.3920E-03
CCNA_03748	1.0720E-04	1.1491E-04	-1.0027E-01	-2.7931E-01
CCNA_03749	8.3410E-05	4.9341E-05	7.5727E-01	2.6766E-01
CCNA_03750	8.0635E-05	1.5663E-04	-9.5790E-01	-8.2634E-01
CCNA_03751	2.3791E-04	1.3686E-04	7.9766E-01	2.9343E-01
CCNA_03752	2.2713E-04	1.1130E-04	1.0290E+00	4.4096E-01
CCNA_03753	9.6718E-05	1.4624E-04	-5.9651E-01	-5.9583E-01
CCNA_03754	2.1784E-04	2.7890E-04	-3.5651E-01	-4.4275E-01
CCNA_03755	7.0171E-05	1.2812E-04	-8.6852E-01	-7.6933E-01
CCNA_03756	2.1295E-04	1.0809E-04	9.7820E-01	4.0858E-01
CCNA_03757	5.9004E-05	6.1573E-05	-6.1567E-02	-2.5462E-01
CCNA_03758	6.7591E-05	1.1916E-04	-8.1800E-01	-7.3710E-01
CCNA_03759	1.1217E-04	1.6238E-04	-5.3378E-01	-5.5582E-01
CCNA_03760	9.7213E-05	1.1186E-04	-2.0250E-01	-3.4451E-01
CCNA_03761	9.2066E-05	1.0775E-04	-2.2696E-01	-3.6012E-01

CCNA_03762	4.0072E-04	2.3689E-04	7.5838E-01	2.6837E-01
CCNA_03763	1.1221E-04	1.2643E-04	-1.7210E-01	-3.2512E-01
CCNA_03764	1.0574E-04	1.3280E-04	-3.2883E-01	-4.2509E-01
CCNA_03765	1.0083E-04	9.5525E-05	7.7903E-02	-1.6566E-01
CCNA_03766	6.7290E-05	2.3113E-05	1.5413E+00	7.6775E-01
CCNA_03767	9.1220E-05	7.9174E-05	2.0424E-01	-8.5080E-02
CCNA_03768	1.3514E-04	2.0504E-04	-6.0150E-01	-5.9901E-01
CCNA_03769	1.1152E-04	1.2111E-04	-1.1902E-01	-2.9127E-01
CCNA_03770	2.5116E-04	2.0907E-04	2.6456E-01	-4.6606E-02
CCNA_03771	2.6448E-04	2.3826E-04	1.5060E-01	-1.1929E-01
CCNA_03772	7.3172E-05	7.2984E-05	3.6394E-03	-2.1303E-01
CCNA_03773	1.1046E-04	1.3070E-04	-2.4285E-01	-3.7025E-01
CCNA_03774	1.0090E-04	1.6243E-04	-6.8682E-01	-6.5344E-01
CCNA_03775	9.5280E-05	1.6069E-04	-7.5400E-01	-6.9629E-01
CCNA_03776	7.7306E-05	1.6243E-04	-1.0711E+00	-8.9856E-01
CCNA_03777	1.6197E-04	2.0128E-04	-3.1349E-01	-4.1531E-01
CCNA_03778	1.6198E-04	2.0343E-04	-3.2871E-01	-4.2502E-01
CCNA_03779	1.2664E-04	2.6250E-04	-1.0516E+00	-8.8609E-01
CCNA_03780	1.2800E-04	1.6242E-04	-3.4353E-01	-4.3447E-01
CCNA_03781	3.7518E-04	2.2796E-04	7.1878E-01	2.4311E-01
CCNA_03782	3.7151E-04	2.3161E-04	6.8168E-01	2.1945E-01
CCNA_03783	1.1521E-04	1.1523E-04	-3.7260E-04	-2.1559E-01
CCNA_03784	7.3607E-05	9.1489E-05	-3.1380E-01	-4.1551E-01
CCNA_04010	6.6768E-05	7.4915E-05	-1.6616E-01	-3.2134E-01
CCNA_03786	1.3510E-04	6.9769E-05	9.5324E-01	3.9266E-01
CCNA_03787	8.4127E-05	7.7733E-05	1.1397E-01	-1.4266E-01
CCNA_03788	9.0600E-05	1.6221E-04	-8.4028E-01	-7.5132E-01
CCNA_03789	3.1497E-04	1.6255E-04	9.5426E-01	3.9332E-01
CCNA_03790	2.8662E-04	1.6279E-04	8.1605E-01	3.0516E-01
CCNA_03791	1.0271E-04	1.1821E-04	-2.0286E-01	-3.4474E-01
CCNA_03792	8.6110E-05	1.6088E-04	-9.0177E-01	-7.9054E-01
CCNA_03793	1.9738E-04	1.6650E-04	2.4543E-01	-5.8807E-02
CCNA_03794	2.0953E-04	2.4781E-04	-2.4209E-01	-3.6977E-01
CCNA_03795	1.0184E-04	1.2289E-04	-2.7112E-01	-3.8828E-01
CCNA_03796	8.2069E-05	1.2378E-04	-5.9283E-01	-5.9348E-01
CCNA_03797	5.8800E-05	1.3906E-04	-1.2418E+00	-1.0074E+00
CCNA_03798	8.5454E-05	1.6018E-04	-9.0648E-01	-7.9354E-01
CCNA_03799	8.7696E-05	8.4801E-05	4.8366E-02	-1.8450E-01
CCNA_03800	1.1340E-04	1.6235E-04	-5.1775E-01	-5.4559E-01
CCNA_03801	1.1351E-04	1.1843E-04	-6.1282E-02	-2.5444E-01
CCNA_03802	6.8687E-05	2.1008E-05	1.7086E+00	8.7449E-01
CCNA_03803	1.5894E-04	9.5898E-05	7.2879E-01	2.4950E-01
CCNA_03804	8.9551E-05	1.1865E-04	-4.0591E-01	-4.7426E-01
CCNA_03805	2.5444E-04	8.8870E-05	1.5175E+00	7.5255E-01
CCNA_03806	2.7653E-04	1.3807E-04	1.0020E+00	4.2374E-01
CCNA_03807	8.8094E-05	7.1277E-05	3.0552E-01	-2.0479E-02
CCNA_03808	9.6459E-05	1.0357E-04	-1.0268E-01	-2.8085E-01
CCNA_03809	8.0492E-05	7.9837E-05	1.1714E-02	-2.0788E-01
CCNA_03810	8.1662E-05	1.4453E-04	-8.2368E-01	-7.4073E-01
CCNA_03811	6.0641E-04	3.1137E-04	9.6164E-01	3.9803E-01
CCNA_03812	5.9756E-04	3.0224E-04	9.8338E-01	4.1189E-01
CCNA_03813	6.5049E-04	1.6693E-04	1.9623E+00	1.0363E+00
CCNA_03814	7.1304E-05	1.5652E-04	-1.1343E+00	-9.3883E-01

CCNA_03815	4.9372E-05	7.9183E-05	-6.8152E-01	-6.5005E-01
CCNA_03816	2.0434E-04	1.6243E-04	3.3111E-01	-4.1538E-03
CCNA_03817	1.8055E-04	1.6243E-04	1.5257E-01	-1.1804E-01
CCNA_03818	4.6653E-05	0.0000E+00	1.2459E+01	7.7315E+00
CCNA_03819	1.0086E-04	1.8066E-06	5.7964E+00	3.4819E+00
CCNA_03820	7.9586E-05	1.0053E-04	-3.3708E-01	-4.3036E-01
CCNA_03821	2.7952E-04	1.9300E-04	5.3435E-01	1.2548E-01
CCNA_03822	1.1084E-04	1.5766E-04	-5.0830E-01	-5.3957E-01
CCNA_03823	1.0484E-04	9.8086E-05	9.5992E-02	-1.5412E-01
CCNA_03824	7.1507E-05	1.6190E-04	-1.1790E+00	-9.6734E-01
CCNA_03825	1.0152E-04	1.0027E-04	1.7863E-02	-2.0396E-01
CCNA_03826	1.5574E-04	6.5302E-05	1.2538E+00	5.8435E-01
CCNA_03827	1.5212E-04	1.0031E-04	6.0072E-01	1.6781E-01
CCNA_03828	1.3028E-04	1.6093E-04	-3.0476E-01	-4.0974E-01
CCNA_03829	3.2321E-04	3.8108E-04	-2.3764E-01	-3.6693E-01
CCNA_03830	6.5725E-04	3.9903E-04	7.1992E-01	2.4385E-01
CCNA_03831	5.4454E-05	5.7661E-05	-8.2660E-02	-2.6808E-01
CCNA_03832	7.8823E-05	7.7434E-05	2.5572E-02	-1.9904E-01
CCNA_03833	7.3718E-05	1.6243E-04	-1.1397E+00	-9.4229E-01
CCNA_03834	8.0353E-05	1.2750E-04	-6.6613E-01	-6.4024E-01
CCNA_03835	1.3362E-04	1.4749E-04	-1.4251E-01	-3.0625E-01
CCNA_03836	1.7223E-04	1.9639E-04	-1.8934E-01	-3.3612E-01
CCNA_03837	2.5516E-04	1.6273E-04	6.4884E-01	1.9851E-01
CCNA_03838	2.4588E-04	9.4240E-05	1.3834E+00	6.6706E-01
CCNA_03839	1.8559E-04	1.6211E-04	1.9509E-01	-9.0915E-02
CCNA_03840	1.1549E-04	9.8981E-05	2.2246E-01	-7.3460E-02
CCNA_03841	6.0641E-05	1.0739E-04	-8.2453E-01	-7.4127E-01
CCNA_03842	1.1139E-04	1.5410E-04	-4.6824E-01	-5.1401E-01
CCNA_03843	7.8194E-05	1.5876E-04	-1.0217E+00	-8.6706E-01
CCNA_03844	8.4899E-05	1.5079E-04	-8.2874E-01	-7.4395E-01
CCNA_03845	6.7253E-05	1.1417E-04	-7.6352E-01	-7.0236E-01
CCNA_03846	1.1460E-04	1.5943E-04	-4.7625E-01	-5.1912E-01
CCNA_03847	9.2700E-05	9.2517E-05	2.7954E-03	-2.1357E-01
CCNA_03848	1.4502E-04	1.8759E-04	-3.7136E-01	-4.5222E-01
CCNA_03849	1.3265E-04	1.6304E-04	-2.9765E-01	-4.0520E-01
CCNA_03850	8.2282E-05	8.0981E-05	2.2919E-02	-2.0073E-01
CCNA_03851	8.5657E-05	1.0358E-04	-2.7413E-01	-3.9021E-01
CCNA_03852	1.1828E-04	1.6243E-04	-4.5753E-01	-5.0719E-01
CCNA_03853	8.5287E-05	8.0451E-05	8.4155E-02	-1.6167E-01
CCNA_03854	8.1833E-05	1.1828E-04	-5.3148E-01	-5.5435E-01
CCNA_03855	5.4722E-05	1.4884E-04	-1.4436E+00	-1.1361E+00
CCNA_03856	8.1995E-05	1.4926E-04	-8.6420E-01	-7.6658E-01
CCNA_03857	1.4435E-04	1.6547E-04	-1.9704E-01	-3.4103E-01
CCNA_03858	1.5439E-04	2.1062E-04	-4.4804E-01	-5.0113E-01
CCNA_03859	4.2877E-04	2.0297E-04	1.0789E+00	4.7278E-01
CCNA_03860	5.2678E-04	2.3309E-04	1.1763E+00	5.3492E-01
CCNA_03861	9.6635E-05	1.5869E-04	-7.1559E-01	-6.7178E-01
CCNA_03862	1.0479E-04	1.6243E-04	-6.3233E-01	-6.1868E-01
CCNA_03863	3.8530E-04	2.5832E-04	5.7682E-01	1.5257E-01
CCNA_03864	3.7846E-04	3.0472E-04	3.1261E-01	-1.5958E-02
CCNA_03865	7.2354E-05	6.7672E-05	9.6420E-02	-1.5385E-01
CCNA_03866	3.5730E-04	1.0114E-04	1.8206E+00	9.4593E-01
CCNA_03867	8.8843E-05	7.3705E-05	2.6941E-01	-4.3509E-02

CCNA_03868	7.9711E-05	1.1776E-04	-5.6301E-01	-5.7446E-01
CCNA_03869	9.5359E-05	1.0119E-04	-8.5725E-02	-2.7003E-01
CCNA_03870	8.3553E-05	8.9028E-05	-9.1614E-02	-2.7379E-01
CCNA_03871	2.3267E-04	1.3395E-04	7.9652E-01	2.9270E-01
CCNA_03872	1.1578E-04	6.3686E-05	8.6225E-01	3.3463E-01
CCNA_03873	1.4888E-04	1.2328E-04	2.7216E-01	-4.1755E-02
CCNA_03874	1.4924E-04	8.8961E-05	7.4628E-01	2.6066E-01
CCNA_03875	9.2862E-05	1.3739E-04	-5.6515E-01	-5.7583E-01
CCNA_03876	1.6812E-04	1.1273E-04	5.7660E-01	1.5243E-01
CCNA_03877	1.1501E-04	1.6144E-04	-4.8924E-01	-5.2741E-01
CCNA_03878	8.6568E-05	1.6243E-04	-9.0787E-01	-7.9443E-01
CCNA_03879	9.7717E-05	9.6528E-05	1.7612E-02	-2.0412E-01
