# Supplementary material for: DNA Binding of the Cell Cycle Transcriptional Regulator GcrA Depends on N6-Adenosine Methylation in Caulobacter crescentus and Other Alphaproteobacteria
Source: PLoS Genet. 2013 May 30;9(5):e1003541. doi: 10.1371/journal.pgen.1003541 (PMC3667746; doi:10.1371/journal.pgen.1003541)
Supplement: Table S7 — Log2 ratios of Figure 7C. (PDF) [file pgen.1003541.s019.pdf]

Gene	Normalized coverage 100-300 m6A wt	Normalized coverage 100-300 m6A delccrM	log2(RATIO)	Z-score(log2RATIO)
CCNA_00001	4.46077E-04	1.51962E-04	1.5535E+00	9.1186E-01
CCNA_00002	7.18130E-05	1.30370E-04	-8.6032E-01	-4.6470E-01
CCNA_00003	1.01419E-04	1.47399E-04	-5.3943E-01	-2.8170E-01
CCNA_00004	3.71841E-05	1.43436E-04	-1.9476E+00	-1.0848E+00
CCNA_00005	1.47671E-04	1.42828E-04	4.8057E-02	5.3328E-02
CCNA_00006	1.80533E-04	1.47099E-04	2.9543E-01	1.9440E-01
CCNA_00007	1.95732E-04	1.18805E-04	7.2022E-01	4.3665E-01
CCNA_00008	2.72748E-04	1.46221E-04	8.9937E-01	5.3881E-01
CCNA_00009	2.86162E-04	9.72127E-05	1.5575E+00	9.1414E-01
CCNA_00010	2.68478E-04	1.47099E-04	8.6796E-01	5.2090E-01
CCNA_00011	1.47782E-04	1.47099E-04	6.6447E-03	2.9712E-02
CCNA_00012	1.26504E-04	9.44659E-05	4.2124E-01	2.6615E-01
CCNA_00013	9.10295E-05	8.87395E-05	3.6683E-02	4.6842E-02
CCNA_00014	6.52550E-05	1.44127E-04	-1.1432E+00	-6.2601E-01
CCNA_00015	5.71500E-05	1.47346E-04	-1.3664E+00	-7.5329E-01
CCNA_00016	1.40156E-04	1.47324E-04	-7.2001E-02	-1.5138E-02
CCNA_00017	1.55361E-04	1.11540E-04	4.7799E-01	2.9851E-01
CCNA_00018	2.30237E-05	1.26685E-04	-2.4599E+00	-1.3769E+00
CCNA_00019	2.55910E-05	9.00829E-05	-1.8156E+00	-1.0095E+00
CCNA_00020	2.29563E-04	1.33470E-04	7.8232E-01	4.7206E-01
CCNA_00021	4.66133E-05	1.39894E-04	-1.5855E+00	-8.7825E-01
CCNA_00022	3.04184E-05	1.47099E-04	-2.2737E+00	-1.2707E+00
CCNA_00023	2.65540E-05	9.04657E-05	-1.7684E+00	-9.8255E-01
CCNA_00024	9.51858E-05	5.07415E-05	9.0741E-01	5.4340E-01
CCNA_00025	1.94347E-04	1.47099E-04	4.0180E-01	2.5506E-01
CCNA_00026	3.95918E-05	1.37800E-04	-1.7993E+00	-1.0002E+00
CCNA_00027	2.03981E-04	9.00904E-05	1.1789E+00	6.9822E-01
CCNA_00028	1.07257E-04	9.67699E-05	1.4838E-01	1.1054E-01
CCNA_00029	1.29393E-04	1.45538E-04	-1.6967E-01	-7.0836E-02
CCNA_00030	1.24626E-04	8.70434E-05	5.1771E-01	3.2116E-01
CCNA_00031	3.60807E-04	1.38198E-04	1.3844E+00	8.1543E-01
CCNA_00032	1.32752E-04	1.32974E-04	-2.4600E-03	2.4520E-02
CCNA_00033	8.92237E-05	8.28781E-05	1.0635E-01	8.6574E-02
CCNA_00034	3.27509E-05	1.22760E-04	-1.9062E+00	-1.0611E+00
CCNA_00035	3.93600E-05	1.05070E-04	-1.4165E+00	-7.8190E-01
CCNA_00036	1.45206E-04	7.41722E-05	9.6903E-01	5.7854E-01
CCNA_00037	4.86297E-05	1.16868E-04	-1.2650E+00	-6.9547E-01
CCNA_00038	2.44503E-05	6.77329E-05	-1.4700E+00	-8.1238E-01
CCNA_00039	7.15331E-05	1.19112E-04	-7.3567E-01	-3.9361E-01
CCNA_00040	6.27239E-05	1.47677E-04	-1.2354E+00	-6.7857E-01
CCNA_00041	1.22194E-04	7.67465E-05	6.7090E-01	4.0852E-01
CCNA_00042	1.40592E-04	1.49876E-04	-9.2289E-02	-2.6708E-02
CCNA_00043	1.58951E-04	1.47099E-04	1.1175E-01	8.9652E-02
CCNA_00044	3.02535E-04	1.42963E-04	1.0814E+00	6.4262E-01
CCNA_00045	3.02803E-04	1.09258E-04	1.4706E+00	8.6454E-01
CCNA_00046	1.01365E-04	8.79890E-05	2.0408E-01	1.4230E-01
CCNA_00047	2.54405E-05	1.46198E-04	-2.5226E+00	-1.4127E+00
CCNA_00048	4.26830E-04	8.92348E-05	2.2579E+00	1.3135E+00
CCNA_00049	2.01844E-04	9.32200E-05	1.1144E+00	6.6146E-01
CCNA_00050	5.70417E-05	1.15690E-04	-1.0202E+00	-5.5587E-01

CCNA_00051	2.24730E-05	9.45784E-05	-2.0732E+00	-1.1564E+00
CCNA_00052	1.30089E-04	1.21882E-04	9.3957E-02	7.9504E-02
CCNA_00053	2.18108E-05	8.82667E-05	-2.0167E+00	-1.1242E+00
CCNA_00054	3.77017E-05	1.22812E-04	-1.7037E+00	-9.4567E-01
CCNA_00055	2.82256E-04	7.11627E-05	1.9877E+00	1.1594E+00
CCNA_00056	2.37009E-05	9.68149E-05	-2.0302E+00	-1.1319E+00
CCNA_00057	1.67742E-04	1.36817E-04	2.9395E-01	1.9355E-01
CCNA_00058	5.29425E-05	1.46783E-04	-1.4712E+00	-8.1306E-01
CCNA_00059	3.00121E-05	1.09919E-04	-1.8728E+00	-1.0421E+00
CCNA_00060	5.70356E-05	1.46408E-04	-1.3601E+00	-7.4969E-01
CCNA_00061	4.06993E-05	8.90697E-05	-1.1299E+00	-6.1846E-01
CCNA_00062	2.92808E-05	1.18805E-04	-2.0205E+00	-1.1263E+00
CCNA_00063	6.37411E-05	1.59324E-04	-1.3217E+00	-7.2780E-01
CCNA_00064	1.22492E-04	8.43040E-05	5.3892E-01	3.3326E-01
CCNA_00065	3.78251E-05	9.74153E-05	-1.3648E+00	-7.5239E-01
CCNA_00066	2.85916E-05	1.46858E-04	-2.3607E+00	-1.3203E+00
CCNA_00067	2.26385E-05	4.12477E-05	-8.6560E-01	-4.6771E-01
CCNA_00068	2.11006E-05	1.27683E-04	-2.5971E+00	-1.4551E+00
CCNA_00069	6.16585E-05	1.47099E-04	-1.2544E+00	-6.8944E-01
CCNA_00070	1.01112E-04	9.01655E-05	1.6523E-01	1.2015E-01
CCNA_00071	1.94904E-05	3.51536E-05	-8.5099E-01	-4.5938E-01
CCNA_00072	1.30405E-04	8.42365E-05	6.3038E-01	3.8542E-01
CCNA_00073	1.42868E-04	1.34430E-04	8.7771E-02	7.5976E-02
CCNA_00074	1.18246E-04	1.47099E-04	-3.1503E-01	-1.5373E-01
CCNA_00075	3.25944E-05	9.05557E-05	-1.4742E+00	-8.1476E-01
CCNA_00076	5.01436E-05	1.35668E-04	-1.4359E+00	-7.9296E-01
CCNA_00077	9.78855E-05	1.47662E-04	-5.9316E-01	-3.1234E-01
CCNA_00078	2.05618E-05	1.47684E-04	-2.8443E+00	-1.5961E+00
CCNA_00079	2.76796E-05	1.29162E-04	-2.2222E+00	-1.2414E+00
CCNA_00080	2.76826E-05	1.32757E-04	-2.2617E+00	-1.2638E+00
CCNA_00081	4.57802E-04	1.47099E-04	1.6379E+00	9.5997E-01
CCNA_00082	1.47084E-04	1.47354E-04	-2.6880E-03	2.4390E-02
CCNA_00083	1.84632E-04	1.00725E-04	8.7415E-01	5.2443E-01
CCNA_00084	1.94260E-04	5.63478E-05	1.7854E+00	1.0441E+00
CCNA_00085	4.51054E-05	9.62896E-05	-1.0941E+00	-5.9801E-01
CCNA_00086	4.99630E-05	2.51269E-05	9.9129E-01	5.9123E-01
CCNA_00087	2.09092E-04	6.44832E-05	1.6970E+00	9.9368E-01
CCNA_00088	1.88361E-04	7.02846E-05	1.4221E+00	8.3691E-01
CCNA_00089	6.31151E-05	1.43684E-04	-1.1868E+00	-6.5091E-01
CCNA_00090	3.67718E-05	1.47099E-04	-2.0001E+00	-1.1147E+00
CCNA_00091	2.94018E-04	1.12771E-04	1.3824E+00	8.1429E-01
CCNA_00092	1.61214E-04	8.47844E-05	9.2701E-01	5.5457E-01
CCNA_00093	2.19920E-04	1.24268E-04	8.2345E-01	4.9552E-01
CCNA_00094	1.65870E-04	9.33476E-05	8.2928E-01	4.9884E-01
CCNA_00095	2.54676E-05	1.25019E-04	-2.2953E+00	-1.2831E+00
CCNA_00096	2.82123E-05	7.83225E-05	-1.4731E+00	-8.1414E-01
CCNA_00097	1.41363E-05	1.51219E-04	-3.4189E+00	-1.9238E+00
CCNA_00098	2.73125E-05	1.38228E-04	-2.3393E+00	-1.3081E+00
CCNA_00099	1.49459E-05	8.69308E-05	-2.5400E+00	-1.4226E+00
CCNA_00100	2.28281E-05	1.47099E-04	-2.6878E+00	-1.5069E+00
CCNA_00101	6.87672E-05	1.39639E-04	-1.0219E+00	-5.5686E-01
CCNA_00102	8.33399E-05	9.54115E-05	-1.9522E-01	-8.5406E-02
CCNA_00103	2.71018E-05	6.53313E-05	-1.2694E+00	-6.9798E-01

CCNA_00104	1.38871E-04	8.43941E-05	7.1843E-01	4.3563E-01
CCNA_00105	1.71378E-04	1.05941E-04	6.9384E-01	4.2160E-01
CCNA_00106	8.32165E-05	1.09994E-04	-4.0253E-01	-2.0363E-01
CCNA_00107	1.67465E-04	1.35924E-04	3.0101E-01	1.9758E-01
CCNA_00108	3.04756E-05	8.07392E-05	-1.4056E+00	-7.7566E-01
CCNA_00109	3.66574E-05	1.36442E-04	-1.8961E+00	-1.0554E+00
CCNA_00110	4.01516E-05	8.06191E-05	-1.0057E+00	-5.4760E-01
CCNA_00111	3.09000E-05	1.19930E-04	-1.9565E+00	-1.0898E+00
CCNA_00112	7.93130E-05	1.17319E-04	-5.6484E-01	-2.9619E-01
CCNA_00113	4.11177E-05	5.64303E-05	-4.5680E-01	-2.3458E-01
CCNA_00114	1.44469E-04	7.75720E-05	8.9704E-01	5.3748E-01
CCNA_00115	1.27675E-04	1.27541E-04	1.4677E-03	2.6759E-02
CCNA_00116	1.09448E-04	1.46941E-04	-4.2502E-01	-2.1646E-01
CCNA_00117	9.44545E-05	1.40262E-04	-5.7046E-01	-2.9940E-01
CCNA_00118	8.43421E-05	9.72878E-05	-2.0607E-01	-9.1592E-02
CCNA_00119	3.32887E-04	1.16065E-04	1.5200E+00	8.9275E-01
CCNA_00120	2.35926E-05	7.93432E-05	-1.7497E+00	-9.7191E-01
CCNA_00121	2.14527E-05	6.98719E-05	-1.7035E+00	-9.4555E-01
CCNA_00122	2.41734E-05	8.63679E-05	-1.8370E+00	-1.0217E+00
CCNA_00123	2.25031E-05	1.44157E-04	-2.6793E+00	-1.5020E+00
CCNA_00124	1.69680E-04	1.13326E-04	5.8227E-01	3.5798E-01
CCNA_00125	4.41303E-05	1.47099E-04	-1.7369E+00	-9.6460E-01
CCNA_00126	1.22185E-04	1.00770E-04	2.7793E-01	1.8442E-01
CCNA_00127	1.21761E-04	1.12268E-04	1.1705E-01	9.2671E-02
CCNA_00128	2.96690E-05	1.19713E-04	-2.0125E+00	-1.1218E+00
CCNA_00129	2.98887E-05	9.72277E-05	-1.7017E+00	-9.4454E-01
CCNA_00130	3.70306E-05	1.20381E-04	-1.7008E+00	-9.4400E-01
CCNA_00131	3.71781E-05	1.20861E-04	-1.7008E+00	-9.4401E-01
CCNA_00132	3.32776E-04	1.35451E-04	1.2967E+00	7.6541E-01
CCNA_00133	5.85344E-05	1.18024E-04	-1.0117E+00	-5.5105E-01
CCNA_00134	2.54314E-05	7.41047E-05	-1.5429E+00	-8.5397E-01
CCNA_00135	5.94614E-05	9.17565E-05	-6.2590E-01	-3.3102E-01
CCNA_00136	1.95145E-05	6.96392E-05	-1.8353E+00	-1.0207E+00
CCNA_00137	1.43611E-04	1.47099E-04	-3.4662E-02	6.1553E-03
CCNA_00138	1.49579E-04	1.47099E-04	2.4079E-02	3.9654E-02
CCNA_00139	1.13897E-04	9.85036E-05	2.0941E-01	1.4534E-01
CCNA_00140	1.49149E-04	6.59467E-05	1.1772E+00	6.9728E-01
CCNA_00141	4.00131E-05	1.46746E-04	-1.8747E+00	-1.0432E+00
CCNA_00142	4.56261E-05	9.74379E-05	-1.0946E+00	-5.9833E-01
CCNA_00143	3.56967E-04	1.11930E-04	1.6731E+00	9.8006E-01
CCNA_00144	1.55345E-04	1.27946E-04	2.7989E-01	1.8554E-01
CCNA_00145	2.19153E-04	1.07652E-04	1.0255E+00	6.1073E-01
CCNA_00146	1.48423E-04	1.47346E-04	1.0462E-02	3.1889E-02
CCNA_00147	4.25683E-05	7.71367E-05	-8.5768E-01	-4.6319E-01
CCNA_00148	2.23135E-05	1.39976E-04	-2.6491E+00	-1.4848E+00
CCNA_00149	2.50372E-05	1.47369E-04	-2.5572E+00	-1.4324E+00
CCNA_00150	4.55148E-04	1.29484E-04	1.8135E+00	1.0601E+00
CCNA_00151	1.44002E-04	6.12711E-05	1.2327E+00	7.2888E-01
CCNA_00152	1.32337E-04	7.72943E-05	7.7567E-01	4.6827E-01
CCNA_00153	4.69203E-05	1.33447E-04	-1.5080E+00	-8.3404E-01
CCNA_00154	2.38818E-04	1.47662E-04	6.9356E-01	4.2144E-01
CCNA_00155	2.42625E-04	1.47624E-04	7.1675E-01	4.3467E-01
CCNA_00156	2.60364E-05	1.07427E-04	-2.0447E+00	-1.1401E+00

CCNA_00157	1.48655E-04	1.47414E-04	1.2052E-02	3.2795E-02
CCNA_00158	1.00221E-04	1.10264E-04	-1.3783E-01	-5.2680E-02
CCNA_00159	4.23026E-04	1.14512E-04	1.8852E+00	1.1010E+00
CCNA_00160	1.34633E-04	1.47099E-04	-1.2779E-01	-4.6954E-02
CCNA_00161	1.55538E-04	1.34941E-04	2.0489E-01	1.4277E-01
CCNA_00162	1.41050E-04	1.46183E-04	-5.1616E-02	-3.5129E-03
CCNA_00163	1.51234E-04	1.39346E-04	1.1807E-01	9.3252E-02
CCNA_00164	1.24774E-04	8.17073E-05	6.1068E-01	3.7418E-01
CCNA_00165	1.30790E-04	6.07833E-05	1.1054E+00	6.5628E-01
CCNA_00166	5.64999E-05	1.41320E-05	1.9986E+00	1.1657E+00
CCNA_00167	4.72513E-05	2.89094E-05	7.0853E-01	4.2998E-01
CCNA_00168	1.47876E-04	1.47039E-04	8.1442E-03	3.0567E-02
CCNA_00169	8.93381E-05	1.60458E-05	2.4765E+00	1.4382E+00
CCNA_00170	8.35716E-05	1.46641E-04	-8.1122E-01	-4.3670E-01
CCNA_00171	6.91645E-05	9.01505E-05	-3.8236E-01	-1.9213E-01
CCNA_00172	2.43570E-05	1.47076E-04	-2.5941E+00	-1.4534E+00
CCNA_00173	5.74510E-05	1.47429E-04	-1.3596E+00	-7.4943E-01
CCNA_00174	3.24168E-05	1.28208E-04	-1.9836E+00	-1.1053E+00
CCNA_00175	2.27468E-05	1.42806E-04	-2.6502E+00	-1.4854E+00
CCNA_00176	6.20949E-05	1.39361E-04	-1.1663E+00	-6.3919E-01
CCNA_00177	1.28313E-04	1.46040E-04	-1.8674E-01	-8.0572E-02
CCNA_00178	6.46711E-05	6.93315E-05	-1.0048E-01	-3.1378E-02
CCNA_00179	1.82306E-04	1.01401E-04	8.4621E-01	5.0850E-01
CCNA_00180	3.41233E-05	8.28331E-05	-1.2795E+00	-7.0372E-01
CCNA_00181	1.66776E-04	9.76705E-05	7.7183E-01	4.6608E-01
CCNA_00182	2.18018E-04	9.94792E-05	1.1319E+00	6.7142E-01
CCNA_00183	2.24964E-04	1.47099E-04	6.1286E-01	3.7542E-01
CCNA_00184	7.73778E-05	1.01633E-04	-3.9343E-01	-1.9844E-01
CCNA_00185	2.10705E-04	9.63646E-05	1.1286E+00	6.6951E-01
CCNA_00186	1.35545E-04	1.44277E-04	-9.0109E-02	-2.5465E-02
CCNA_00187	1.70947E-05	1.48750E-04	-3.1211E+00	-1.7540E+00
CCNA_00188	5.13294E-05	1.22595E-04	-1.2560E+00	-6.9037E-01
CCNA_00189	1.36893E-04	1.42881E-04	-6.1804E-02	-9.3228E-03
CCNA_00190	2.18560E-05	4.21258E-05	-9.4673E-01	-5.1398E-01
CCNA_00191	1.34913E-04	1.04725E-04	3.6535E-01	2.3427E-01
CCNA_00192	2.90611E-05	1.26205E-04	-2.1185E+00	-1.1822E+00
CCNA_00193	1.13283E-04	1.11720E-04	1.9983E-02	3.7318E-02
CCNA_00194	3.21189E-05	1.14302E-04	-1.8313E+00	-1.0184E+00
CCNA_00195	9.46290E-05	9.65523E-05	-2.9093E-02	9.3312E-03
CCNA_00196	2.64908E-05	1.47099E-04	-2.4731E+00	-1.3844E+00
CCNA_00197	1.39518E-04	1.02361E-04	4.4671E-01	2.8067E-01
CCNA_00198	1.32978E-04	1.47099E-04	-1.4564E-01	-5.7132E-02
CCNA_00199	1.46205E-04	1.47099E-04	-8.8333E-03	2.0885E-02
CCNA_00200	1.01596E-04	7.97710E-05	3.4882E-01	2.2485E-01
CCNA_00201	1.16353E-04	1.04102E-04	1.6043E-01	1.1741E-01
CCNA_00202	1.58319E-04	1.40764E-04	1.6950E-01	1.2259E-01
CCNA_00203	2.37057E-04	1.47399E-04	6.8545E-01	4.1682E-01
CCNA_00204	4.68330E-05	1.46866E-04	-1.6489E+00	-9.1440E-01
CCNA_00205	9.28443E-05	1.46070E-04	-6.5381E-01	-3.4693E-01
CCNA_00206	5.44504E-05	1.39001E-04	-1.3521E+00	-7.4514E-01
CCNA_00207	1.06135E-04	1.35181E-04	-3.4903E-01	-1.7312E-01
CCNA_00208	7.40190E-05	9.20567E-05	-3.1469E-01	-1.5354E-01
CCNA_00209	1.77180E-04	1.22445E-04	5.3303E-01	3.2989E-01

CCNA_00210	7.92799E-05	9.92991E-05	-3.2488E-01	-1.5935E-01
CCNA_00211	2.73275E-05	1.37815E-04	-2.3342E+00	-1.3052E+00
CCNA_00212	1.71835E-04	1.48329E-04	2.1217E-01	1.4692E-01
CCNA_00213	1.60925E-05	9.99520E-05	-2.6347E+00	-1.4766E+00
CCNA_00214	2.29094E-05	1.47099E-04	-2.6827E+00	-1.5039E+00
CCNA_00215	1.38344E-04	8.84919E-05	6.4455E-01	3.9350E-01
CCNA_00216	2.69278E-04	1.47692E-04	8.6645E-01	5.2004E-01
CCNA_00217	2.84558E-04	1.33379E-04	1.0931E+00	6.4931E-01
CCNA_00218	2.24152E-04	1.18017E-04	9.2541E-01	5.5366E-01
CCNA_00219	2.23622E-04	1.43121E-04	6.4377E-01	3.9305E-01
CCNA_00220	1.60465E-04	1.46701E-04	1.2933E-01	9.9678E-02
CCNA_00221	1.95807E-05	9.33551E-05	-2.2532E+00	-1.2590E+00
CCNA_00222	1.86026E-05	7.96359E-05	-2.0978E+00	-1.1704E+00
CCNA_00223	6.93601E-05	1.13619E-04	-7.1205E-01	-3.8015E-01
CCNA_00224	2.60063E-05	5.81790E-05	-1.1617E+00	-6.3655E-01
CCNA_00225	2.53562E-05	4.07748E-05	-6.8544E-01	-3.6497E-01
CCNA_00226	2.83147E-05	1.45913E-04	-2.3654E+00	-1.3230E+00
CCNA_00227	2.88143E-05	1.47376E-04	-2.3546E+00	-1.3168E+00
CCNA_00228	1.58677E-04	8.32608E-05	9.3028E-01	5.5644E-01
CCNA_00229	1.62593E-04	1.44817E-04	1.6698E-01	1.2115E-01
CCNA_00230	3.10926E-05	7.25211E-05	-1.2218E+00	-6.7087E-01
CCNA_00231	2.27167E-05	8.66231E-05	-1.9309E+00	-1.0752E+00
CCNA_00232	1.47930E-04	9.89163E-05	5.8055E-01	3.5700E-01
CCNA_00233	1.80584E-04	1.29192E-04	4.8310E-01	3.0142E-01
CCNA_00234	2.40139E-05	1.32817E-04	-2.4674E+00	-1.3812E+00
CCNA_00235	5.01075E-05	1.40262E-04	-1.4850E+00	-8.2095E-01
CCNA_00236	3.89026E-05	1.26385E-04	-1.6999E+00	-9.4347E-01
CCNA_00237	3.87762E-05	1.25874E-04	-1.6987E+00	-9.4282E-01
CCNA_00238	1.34296E-04	1.47099E-04	-1.3141E-01	-4.9017E-02
CCNA_00239	2.47121E-05	1.26910E-04	-2.3604E+00	-1.3202E+00
CCNA_00240	1.11179E-04	1.47016E-04	-4.0312E-01	-2.0397E-01
CCNA_00241	5.99369E-05	1.42708E-04	-1.2516E+00	-6.8781E-01
CCNA_00242	1.69175E-04	8.41464E-05	1.0074E+00	6.0044E-01
CCNA_00243	1.27636E-04	1.09146E-04	2.2571E-01	1.5464E-01
CCNA_00244	1.56880E-04	8.88371E-05	8.2034E-01	4.9374E-01
CCNA_00245	1.62460E-04	1.31150E-04	3.0881E-01	2.0203E-01
CCNA_00246	8.62141E-05	9.56216E-05	-1.4948E-01	-5.9321E-02
CCNA_00247	1.12422E-04	7.25211E-05	6.3234E-01	3.8653E-01
CCNA_00248	6.85686E-05	1.21889E-04	-8.2998E-01	-4.4739E-01
CCNA_00249	7.18641E-05	1.26040E-04	-8.1056E-01	-4.3632E-01
CCNA_00250	7.96440E-05	1.19870E-04	-5.8987E-01	-3.1047E-01
CCNA_00251	9.75695E-05	1.25034E-04	-3.5786E-01	-1.7816E-01
CCNA_00252	2.64969E-05	8.77113E-05	-1.7269E+00	-9.5889E-01
CCNA_00253	2.37912E-05	1.23510E-04	-2.3760E+00	-1.3291E+00
CCNA_00254	1.13990E-04	1.47099E-04	-3.6791E-01	-1.8389E-01
CCNA_00255	3.41413E-05	9.83835E-05	-1.5269E+00	-8.4482E-01
CCNA_00256	1.03303E-04	1.46003E-04	-4.9915E-01	-2.5873E-01
CCNA_00257	9.36629E-05	1.47504E-04	-6.5523E-01	-3.4774E-01
CCNA_00258	1.37655E-04	1.35368E-04	2.4117E-02	3.9676E-02
CCNA_00259	4.84221E-05	1.45620E-04	-1.5885E+00	-8.7994E-01
CCNA_00260	3.01352E-04	1.47099E-04	1.0346E+00	6.1594E-01
CCNA_00261	2.65715E-04	1.37657E-04	9.4874E-01	5.6696E-01
CCNA_00262	2.02097E-05	9.47210E-05	-2.2285E+00	-1.2450E+00

CCNA_00263	2.41915E-05	8.94375E-05	-1.8863E+00	-1.0498E+00
CCNA_00264	7.46902E-05	1.38168E-04	-8.8745E-01	-4.8017E-01
CCNA_00265	5.76707E-05	7.41197E-05	-3.6209E-01	-1.8057E-01
CCNA_00266	5.03091E-05	1.22242E-04	-1.2809E+00	-7.0452E-01
CCNA_00267	4.97638E-04	1.46971E-04	1.7595E+00	1.0293E+00
CCNA_00268	4.91594E-04	1.45680E-04	1.7546E+00	1.0265E+00
CCNA_00269	1.22152E-04	1.32734E-04	-1.1990E-01	-4.2457E-02
CCNA_00270	3.25372E-05	9.81208E-05	-1.5924E+00	-8.8221E-01
CCNA_00271	3.27358E-05	1.47099E-04	-2.1678E+00	-1.2103E+00
CCNA_00272	2.00803E-05	8.28030E-05	-2.0438E+00	-1.1396E+00
CCNA_00273	1.53609E-04	1.05641E-04	5.4002E-01	3.3388E-01
CCNA_00274	2.70446E-05	1.04140E-04	-1.9451E+00	-1.0833E+00
CCNA_00275	2.78244E-04	1.37425E-04	1.0176E+00	6.0626E-01
CCNA_00276	3.32715E-05	7.95083E-05	-1.2568E+00	-6.9082E-01
CCNA_00277	5.11909E-05	1.47024E-04	-1.5221E+00	-8.4208E-01
CCNA_00278	4.86327E-05	7.98986E-05	-7.1629E-01	-3.8256E-01
CCNA_00279	6.33168E-05	1.48187E-04	-1.2268E+00	-6.7367E-01
CCNA_00280	2.11277E-05	1.02751E-04	-2.2819E+00	-1.2754E+00
CCNA_00281	1.07673E-04	1.08485E-04	-1.0905E-02	1.9704E-02
CCNA_00282	1.07221E-04	1.08725E-04	-2.0156E-02	1.4428E-02
CCNA_00283	1.55574E-04	1.08305E-04	5.2243E-01	3.2385E-01
CCNA_00284	1.62845E-04	1.21394E-04	4.2375E-01	2.6757E-01
CCNA_00285	3.17998E-05	4.65687E-05	-5.5044E-01	-2.8798E-01
CCNA_00286	7.24269E-05	1.22272E-04	-7.5552E-01	-4.0494E-01
CCNA_00287	3.35918E-04	1.15570E-04	1.5393E+00	9.0373E-01
CCNA_00288	5.51968E-05	5.40588E-05	2.9934E-02	4.2993E-02
CCNA_00289	1.28406E-04	1.21544E-04	7.9181E-02	7.1077E-02
CCNA_00290	3.42018E-04	1.02121E-04	1.7437E+00	1.0203E+00
CCNA_00291	5.60587E-04	1.35699E-04	2.0465E+00	1.1930E+00
CCNA_00292	6.66003E-05	7.38420E-05	-1.4900E-01	-5.9046E-02
CCNA_00293	1.87786E-04	1.01498E-04	8.8756E-01	5.3208E-01
CCNA_00294	1.18854E-04	1.12831E-04	7.4967E-02	6.8674E-02
CCNA_00295	1.90968E-04	1.47099E-04	3.7649E-01	2.4063E-01
CCNA_00296	1.47984E-04	1.00935E-04	5.5193E-01	3.4068E-01
CCNA_00297	2.38661E-04	1.48352E-04	6.8589E-01	4.1707E-01
CCNA_00298	9.49812E-05	2.31680E-05	2.0351E+00	1.1865E+00
CCNA_00299	6.79185E-05	1.21184E-04	-8.3535E-01	-4.5046E-01
CCNA_00300	2.42772E-04	8.87320E-05	1.4520E+00	8.5395E-01
CCNA_00301	1.60351E-04	6.11810E-05	1.3899E+00	8.1856E-01
CCNA_00302	5.98226E-05	1.60983E-05	1.8932E+00	1.1056E+00
CCNA_00303	3.03040E-05	1.00170E-04	-1.7248E+00	-9.5771E-01
CCNA_00304	9.82376E-05	1.27158E-04	-3.7231E-01	-1.8640E-01
CCNA_00305	2.82063E-05	1.41372E-04	-2.3253E+00	-1.3002E+00
CCNA_00306	3.95828E-05	1.18197E-04	-1.5782E+00	-8.7411E-01
CCNA_00307	1.33824E-04	1.02992E-04	3.7773E-01	2.4134E-01
CCNA_00308	1.39072E-04	1.09191E-04	3.4892E-01	2.2490E-01
CCNA_00309	5.74630E-05	1.47099E-04	-1.3561E+00	-7.4742E-01
CCNA_00310	4.84251E-05	8.94825E-05	-8.8588E-01	-4.7928E-01
CCNA_00311	1.50822E-04	1.02924E-04	5.5119E-01	3.4025E-01
CCNA_00312	7.01396E-05	5.68506E-05	3.0292E-01	1.9867E-01
CCNA_00313	6.52309E-05	1.22219E-04	-9.0587E-01	-4.9067E-01
CCNA_00314	4.68330E-05	1.47399E-04	-1.6541E+00	-9.1738E-01
CCNA_00315	2.48807E-05	1.40382E-04	-2.4962E+00	-1.3976E+00

CCNA_00316	6.64287E-05	1.10159E-04	-7.2974E-01	-3.9023E-01
CCNA_00317	1.30910E-04	1.22985E-04	9.0041E-02	7.7271E-02
CCNA_00318	1.24301E-04	8.63604E-05	5.2531E-01	3.2549E-01
CCNA_00319	1.87073E-04	1.24876E-04	5.8304E-01	3.5842E-01
CCNA_00320	3.18188E-04	1.47624E-04	1.1079E+00	6.5773E-01
CCNA_00321	3.34720E-04	9.83760E-05	1.7665E+00	1.0333E+00
CCNA_00322	3.37892E-04	1.00823E-04	1.7446E+00	1.0209E+00
CCNA_00323	4.15661E-05	1.46956E-04	-1.8219E+00	-1.0131E+00
CCNA_00324	2.56216E-04	9.86612E-05	1.3767E+00	8.1103E-01
CCNA_00325	3.41477E-04	1.45072E-04	1.2350E+00	7.3019E-01
CCNA_00326	3.02496E-04	1.20531E-04	1.3274E+00	7.8293E-01
CCNA_00327	1.03197E-04	9.95993E-05	5.1134E-02	5.5083E-02
CCNA_00328	8.27741E-05	6.60143E-05	3.2629E-01	2.1200E-01
CCNA_00329	1.29553E-04	1.46828E-04	-1.8063E-01	-7.7087E-02
CCNA_00330	2.32073E-04	7.34743E-05	1.6591E+00	9.7209E-01
CCNA_00331	2.15899E-04	5.37210E-05	2.0066E+00	1.1703E+00
CCNA_00332	2.05167E-05	1.49193E-04	-2.8622E+00	-1.6063E+00
CCNA_00333	2.43480E-05	8.55724E-05	-1.8133E+00	-1.0082E+00
CCNA_00334	2.14647E-05	1.48690E-04	-2.7921E+00	-1.5664E+00
CCNA_00335	3.26877E-05	1.19930E-04	-1.8753E+00	-1.0435E+00
CCNA_00336	4.50212E-05	1.47429E-04	-1.7113E+00	-9.5001E-01
CCNA_00337	2.25723E-05	8.55199E-05	-1.9216E+00	-1.0699E+00
CCNA_00338	2.88961E-04	1.46288E-04	9.8201E-01	5.8594E-01
CCNA_00339	4.84823E-05	1.08620E-04	-1.1638E+00	-6.3775E-01
CCNA_00340	1.18869E-04	9.33776E-05	3.4814E-01	2.2446E-01
CCNA_00341	7.32636E-05	1.33732E-04	-8.6820E-01	-4.6919E-01
CCNA_00342	3.54897E-05	1.37635E-04	-1.9553E+00	-1.0892E+00
CCNA_00343	2.17549E-04	1.31353E-04	7.2783E-01	4.4098E-01
CCNA_00344	2.29816E-05	1.03817E-04	-2.1754E+00	-1.2147E+00
CCNA_00345	5.49560E-05	1.01866E-04	-8.9035E-01	-4.8182E-01
CCNA_00346	8.70989E-05	1.45470E-04	-7.4002E-01	-3.9609E-01
CCNA_00347	1.11098E-04	1.21552E-04	-1.2979E-01	-4.8093E-02
CCNA_00348	7.93010E-05	1.18805E-04	-5.8322E-01	-3.0667E-01
CCNA_00349	9.10145E-05	1.47309E-04	-6.9470E-01	-3.7025E-01
CCNA_00350	2.16444E-04	1.11367E-04	9.5859E-01	5.7259E-01
CCNA_00351	2.18978E-04	1.37687E-04	6.6933E-01	4.0763E-01
CCNA_00352	5.49229E-05	1.37169E-04	-1.3205E+00	-7.2712E-01
CCNA_00353	3.91524E-05	1.47099E-04	-1.9096E+00	-1.0631E+00
CCNA_00354	4.41424E-05	1.47894E-04	-1.7443E+00	-9.6882E-01
CCNA_00355	1.44755E-04	9.30849E-05	6.3690E-01	3.8913E-01
CCNA_00356	1.21030E-04	1.46633E-04	-2.7689E-01	-1.3198E-01
CCNA_00357	3.28156E-04	8.61728E-05	1.9290E+00	1.1260E+00
CCNA_00358	3.32475E-05	9.01955E-05	-1.4398E+00	-7.9516E-01
CCNA_00359	3.00723E-05	1.47099E-04	-2.2902E+00	-1.2801E+00
CCNA_00360	4.81301E-05	1.30340E-04	-1.4373E+00	-7.9371E-01
CCNA_00361	1.52176E-04	1.17506E-04	3.7294E-01	2.3860E-01
CCNA_00362	2.65239E-05	4.38669E-05	-7.2592E-01	-3.8805E-01
CCNA_00363	4.84341E-05	1.42851E-04	-1.5604E+00	-8.6394E-01
CCNA_00364	5.28312E-05	1.16936E-04	-1.1463E+00	-6.2777E-01
CCNA_00365	5.10314E-05	7.83300E-05	-6.1823E-01	-3.2664E-01
CCNA_00366	1.34152E-04	1.47076E-04	-1.3274E-01	-4.9776E-02
CCNA_00367	3.83362E-04	7.98160E-05	2.2638E+00	1.3169E+00
CCNA_00368	1.24238E-04	9.18241E-05	4.3608E-01	2.7461E-01

CCNA_00369	3.37365E-04	6.65171E-05	2.3424E+00	1.3617E+00
CCNA_00370	4.42658E-05	1.42828E-04	-1.6900E+00	-9.3784E-01
CCNA_00371	7.34833E-05	1.45410E-04	-9.8465E-01	-5.3560E-01
CCNA_00372	2.11111E-04	9.98995E-05	1.0794E+00	6.4146E-01
CCNA_00373	7.95357E-05	1.14729E-04	-5.2860E-01	-2.7553E-01
CCNA_00374	1.35058E-04	1.47174E-04	-1.2399E-01	-4.4785E-02
CCNA_00375	4.36458E-04	8.31107E-05	2.3926E+00	1.3904E+00
CCNA_00376	1.09099E-04	1.31676E-04	-2.7139E-01	-1.2885E-01
CCNA_00377	2.59882E-05	1.39526E-04	-2.4245E+00	-1.3567E+00
CCNA_00378	4.97132E-05	8.97302E-05	-8.5200E-01	-4.5995E-01
CCNA_00379	3.82468E-04	7.08325E-05	2.4327E+00	1.4132E+00
CCNA_00380	1.98666E-05	1.10211E-04	-2.4717E+00	-1.3837E+00
CCNA_00381	1.77719E-05	3.94765E-05	-1.1514E+00	-6.3071E-01
CCNA_00382	5.31210E-04	1.25342E-04	2.0833E+00	1.2140E+00
CCNA_00383	1.38516E-04	5.48768E-05	1.3356E+00	7.8759E-01
CCNA_00384	2.25338E-04	6.83033E-05	1.7219E+00	1.0079E+00
CCNA_00385	1.38329E-04	1.47632E-04	-9.3939E-02	-2.7649E-02
CCNA_00386	2.07716E-04	1.47099E-04	4.9778E-01	3.0979E-01
CCNA_00387	1.72771E-04	1.24141E-04	4.7682E-01	2.9784E-01
CCNA_00388	1.33667E-04	2.58398E-05	2.3706E+00	1.3778E+00
CCNA_00389	3.79338E-04	1.28381E-04	1.5630E+00	9.1725E-01
CCNA_00390	3.52155E-04	1.47099E-04	1.2594E+00	7.4411E-01
CCNA_00391	7.55088E-05	1.13258E-04	-5.8494E-01	-3.0766E-01
CCNA_00392	1.38558E-04	1.09754E-04	3.3615E-01	2.1762E-01
CCNA_00393	4.83920E-05	5.52446E-05	-1.9117E-01	-8.3098E-02
CCNA_00394	9.86559E-05	8.71935E-05	1.7811E-01	1.2749E-01
CCNA_00395	1.24226E-04	1.47099E-04	-2.4386E-01	-1.1314E-01
CCNA_00396	4.96109E-05	7.84351E-05	-6.6089E-01	-3.5097E-01
CCNA_00397	5.97082E-05	1.07344E-04	-8.4628E-01	-4.5669E-01
CCNA_00398	4.16555E-04	1.01633E-04	2.0350E+00	1.1865E+00
CCNA_00399	2.68342E-04	8.88371E-05	1.5947E+00	9.3536E-01
CCNA_00400	5.38484E-05	1.21184E-04	-1.1702E+00	-6.4143E-01
CCNA_00401	6.63174E-05	7.87053E-05	-2.4715E-01	-1.1502E-01
CCNA_00402	6.03613E-05	1.10407E-04	-8.7116E-01	-4.7088E-01
CCNA_00403	2.61628E-05	4.83174E-05	-8.8509E-01	-4.7882E-01
CCNA_00404	1.83142E-04	8.56700E-05	1.0960E+00	6.5095E-01
CCNA_00405	1.38001E-04	9.51263E-05	5.3668E-01	3.3198E-01
CCNA_00406	1.26441E-04	9.07434E-05	4.7851E-01	2.9881E-01
CCNA_00407	1.71140E-04	1.46911E-04	2.2019E-01	1.5149E-01
CCNA_00408	7.33629E-05	7.92682E-05	-1.1177E-01	-3.7816E-02
CCNA_00409	1.75122E-04	1.43459E-04	2.8767E-01	1.8998E-01
CCNA_00410	8.86519E-05	7.63037E-05	2.1631E-01	1.4928E-01
CCNA_00411	3.41112E-05	6.19465E-05	-8.6082E-01	-4.6499E-01
CCNA_00412	1.50431E-04	6.95341E-05	1.1132E+00	6.6074E-01
CCNA_00413	2.03211E-04	1.11427E-04	8.6680E-01	5.2024E-01
CCNA_00414	1.66839E-04	1.44494E-04	2.0740E-01	1.4420E-01
CCNA_00415	1.91392E-04	8.99628E-05	1.0890E+00	6.4697E-01
CCNA_00416	8.47484E-05	1.08800E-04	-3.6048E-01	-1.7965E-01
CCNA_00417	4.63063E-05	5.51395E-05	-2.5198E-01	-1.1778E-01
CCNA_00418	5.10104E-05	1.41770E-04	-1.4747E+00	-8.1506E-01
CCNA_00419	4.85816E-05	6.23368E-05	-3.5976E-01	-1.7924E-01
CCNA_00420	5.78483E-05	6.88212E-05	-2.5066E-01	-1.1702E-01
CCNA_00421	3.89808E-05	7.78947E-05	-9.9879E-01	-5.4366E-01

CCNA_00422	2.58137E-05	6.63670E-05	-1.3623E+00	-7.5098E-01
CCNA_00423	2.91002E-05	1.15585E-04	-1.9898E+00	-1.1088E+00
CCNA_00424	5.56211E-05	1.45905E-04	-1.3913E+00	-7.6752E-01
CCNA_00425	1.52044E-04	1.24741E-04	2.8549E-01	1.8873E-01
CCNA_00426	3.63655E-04	7.06599E-05	2.3635E+00	1.3738E+00
CCNA_00427	1.68452E-04	1.44810E-04	2.1813E-01	1.5032E-01
CCNA_00428	1.66304E-04	5.11243E-05	1.7016E+00	9.9628E-01
CCNA_00429	2.51407E-04	1.10377E-04	1.1875E+00	7.0313E-01
CCNA_00430	2.58919E-05	8.72760E-05	-1.7530E+00	-9.7380E-01
CCNA_00431	7.02841E-05	6.82283E-05	4.2732E-02	5.0291E-02
CCNA_00432	1.30651E-04	1.30723E-04	-8.3649E-04	2.5445E-02
CCNA_00433	1.32876E-04	1.47099E-04	-1.4675E-01	-5.7766E-02
CCNA_00434	4.90872E-05	3.03879E-05	6.9158E-01	4.2032E-01
CCNA_00435	3.63655E-05	1.47099E-04	-2.0161E+00	-1.1238E+00
CCNA_00436	2.30990E-05	1.47054E-04	-2.6703E+00	-1.4969E+00
CCNA_00437	2.82274E-05	8.10844E-05	-1.5223E+00	-8.4222E-01
CCNA_00438	1.03668E-03	1.47099E-04	2.8170E+00	1.6324E+00
CCNA_00439	9.97581E-04	1.43316E-04	2.7992E+00	1.6222E+00
CCNA_00440	4.04586E-05	1.47414E-04	-1.8653E+00	-1.0378E+00
CCNA_00441	2.17588E-04	8.62629E-05	1.3347E+00	7.8706E-01
CCNA_00442	8.42789E-05	8.16247E-05	4.6084E-02	5.2203E-02
CCNA_00443	8.47333E-05	1.47099E-04	-7.9580E-01	-4.2791E-01
CCNA_00444	5.98045E-05	1.47624E-04	-1.3036E+00	-7.1749E-01
CCNA_00445	3.17685E-04	1.45515E-04	1.1264E+00	6.6827E-01
CCNA_00446	1.83651E-04	1.47384E-04	3.1734E-01	2.0689E-01
CCNA_00447	1.43518E-04	1.30865E-04	1.3309E-01	1.0182E-01
CCNA_00448	1.50852E-04	1.07960E-04	4.8257E-01	3.0112E-01
CCNA_00449	4.97523E-05	1.27083E-04	-1.3529E+00	-7.4562E-01
CCNA_00450	2.21413E-04	1.45260E-04	6.0805E-01	3.7268E-01
CCNA_00451	2.42784E-04	1.17229E-04	1.0503E+00	6.2487E-01
CCNA_00452	2.80679E-05	1.47099E-04	-2.3897E+00	-1.3369E+00
CCNA_00453	1.70234E-04	1.47887E-04	2.0298E-01	1.4168E-01
CCNA_00454	1.84521E-04	5.25578E-05	1.8116E+00	1.0591E+00
CCNA_00455	1.83118E-04	5.27003E-05	1.7967E+00	1.0505E+00
CCNA_00456	9.73076E-05	7.94708E-05	2.9204E-01	1.9246E-01
CCNA_00457	1.42205E-04	1.15382E-04	3.0149E-01	1.9786E-01
CCNA_00458	6.59502E-05	1.12223E-04	-7.6695E-01	-4.1145E-01
CCNA_00459	2.37852E-05	4.62160E-05	-9.5838E-01	-5.2062E-01
CCNA_00460	3.21369E-05	9.61020E-05	-1.5803E+00	-8.7529E-01
CCNA_04006	3.26543E-04	1.47504E-04	1.1465E+00	6.7972E-01
CCNA_00464	6.39000E-04	1.32696E-04	2.2676E+00	1.3191E+00
CCNA_00465	1.10980E-04	7.65964E-05	5.3485E-01	3.3094E-01
CCNA_00466	2.35176E-04	6.02879E-05	1.9636E+00	1.1457E+00
CCNA_00467	1.40255E-04	1.47099E-04	-6.8773E-02	-1.3297E-02
CCNA_00468	4.60047E-04	1.47729E-04	1.6388E+00	9.6047E-01
CCNA_00469	2.35763E-04	8.53322E-06	4.7869E+00	2.7558E+00
CCNA_04002	2.33545E-04	9.62145E-06	4.6002E+00	2.6493E+00
CCNA_00470	2.10648E-04	1.47099E-04	5.1799E-01	3.2132E-01
CCNA_00471	1.00070E-04	1.47099E-04	-5.5580E-01	-2.9104E-01
CCNA_00472	3.02210E-04	1.18805E-04	1.3469E+00	7.9402E-01
CCNA_00473	3.21911E-05	7.22585E-05	-1.1665E+00	-6.3932E-01
CCNA_00474	1.94423E-05	8.27280E-05	-2.0891E+00	-1.1654E+00
CCNA_00475	1.47066E-04	1.18474E-04	3.1183E-01	2.0375E-01

CCNA_00476	4.97975E-05	8.77113E-05	-8.1673E-01	-4.3984E-01
CCNA_00477	1.45982E-04	1.28088E-04	1.8860E-01	1.3348E-01
CCNA_00478	4.00342E-05	1.00170E-04	-1.3231E+00	-7.2863E-01
CCNA_00479	4.03382E-05	1.02699E-04	-1.3482E+00	-7.4293E-01
CCNA_00480	1.03369E-04	9.35803E-05	1.4345E-01	1.0773E-01
CCNA_00481	1.84572E-04	1.47406E-04	3.2434E-01	2.1088E-01
CCNA_00482	5.57566E-05	7.74144E-05	-4.7352E-01	-2.4412E-01
CCNA_00483	1.00257E-04	7.70917E-05	3.7896E-01	2.4203E-01
CCNA_00484	3.87792E-05	1.44960E-04	-1.9023E+00	-1.0589E+00
CCNA_00485	7.29326E-05	8.37637E-05	-1.9983E-01	-8.8037E-02
CCNA_00486	7.63635E-05	5.58825E-05	4.5035E-01	2.8275E-01
CCNA_00487	2.08207E-05	4.24410E-05	-1.0275E+00	-5.6003E-01
CCNA_00488	1.41859E-04	1.47099E-04	-5.2367E-02	-3.9410E-03
CCNA_00489	7.31733E-05	1.45875E-04	-9.9536E-01	-5.4171E-01
CCNA_00490	8.55159E-05	1.09138E-04	-3.5194E-01	-1.7478E-01
CCNA_00491	1.86974E-04	9.92165E-05	9.1410E-01	5.4721E-01
CCNA_00492	1.86570E-04	9.84510E-05	9.2216E-01	5.5181E-01
CCNA_00493	1.14730E-04	4.86026E-05	1.2390E+00	7.3247E-01
CCNA_00494	7.25232E-05	9.75279E-05	-4.2742E-01	-2.1783E-01
CCNA_00495	1.00459E-04	1.15532E-04	-2.0175E-01	-8.9129E-02
CCNA_00496	3.69313E-05	1.39451E-04	-1.9168E+00	-1.0672E+00
CCNA_00497	1.92295E-04	8.57300E-05	1.1653E+00	6.9049E-01
CCNA_00498	7.35465E-05	1.47354E-04	-1.0026E+00	-5.4582E-01
CCNA_00499	2.04806E-05	9.39480E-05	-2.1975E+00	-1.2273E+00
CCNA_00500	4.68330E-05	8.12270E-05	-7.9447E-01	-4.2715E-01
CCNA_00501	1.91723E-04	9.50963E-05	1.0115E+00	6.0274E-01
CCNA_00502	2.20203E-04	1.47819E-04	5.7495E-01	3.5380E-01
CCNA_00503	3.96279E-05	1.10226E-04	-1.4759E+00	-8.1573E-01
CCNA_00504	2.72399E-04	8.06491E-05	1.7559E+00	1.0273E+00
CCNA_00505	2.69796E-04	8.25404E-05	1.7086E+00	1.0003E+00
CCNA_00506	1.51243E-04	1.29057E-04	2.2881E-01	1.5641E-01
CCNA_00507	1.68970E-04	8.39738E-05	1.0087E+00	6.0113E-01
CCNA_00508	2.41283E-05	1.47099E-04	-2.6079E+00	-1.4613E+00
CCNA_00509	2.41584E-05	1.47099E-04	-2.6061E+00	-1.4603E+00
CCNA_00510	2.05910E-04	8.40789E-05	1.2921E+00	7.6277E-01
CCNA_00511	3.46018E-05	7.25361E-05	-1.0679E+00	-5.8306E-01
CCNA_00512	3.62421E-05	1.28951E-04	-1.8311E+00	-1.0183E+00
CCNA_00513	6.23296E-05	1.12928E-04	-8.5744E-01	-4.6306E-01
CCNA_00514	5.52058E-05	8.25253E-05	-5.8007E-01	-3.0488E-01
CCNA_00515	1.19558E-04	1.32044E-04	-1.4335E-01	-5.5827E-02
CCNA_00516	3.05629E-05	1.00740E-04	-1.7208E+00	-9.5538E-01
CCNA_00517	2.22924E-05	9.45034E-05	-2.0837E+00	-1.1624E+00
CCNA_00518	8.73788E-05	1.43256E-04	-7.1327E-01	-3.8084E-01
CCNA_00519	2.00285E-04	1.47099E-04	4.4522E-01	2.7982E-01
CCNA_00520	8.51908E-05	1.16523E-04	-4.5189E-01	-2.3178E-01
CCNA_00521	2.26213E-04	1.47099E-04	6.2085E-01	3.7998E-01
CCNA_00522	6.48487E-05	9.27172E-05	-5.1581E-01	-2.6823E-01
CCNA_00523	1.61897E-04	1.45763E-04	1.5141E-01	1.1227E-01
CCNA_00524	2.81281E-05	1.46108E-04	-2.3769E+00	-1.3296E+00
CCNA_00525	3.78582E-05	6.14212E-05	-6.9819E-01	-3.7224E-01
CCNA_00526	2.92868E-05	7.30915E-05	-1.3195E+00	-7.2653E-01
CCNA_00527	2.04998E-04	1.47099E-04	4.7878E-01	2.9896E-01
CCNA_00528	4.67728E-05	1.09221E-04	-1.2235E+00	-6.7182E-01

CCNA_00529	1.56953E-05	3.73375E-05	-1.2503E+00	-6.8710E-01
CCNA_00530	5.21796E-04	1.47024E-04	1.8274E+00	1.0680E+00
CCNA_00531	8.09773E-05	1.46641E-04	-8.5672E-01	-4.6264E-01
CCNA_00532	4.17888E-05	5.26553E-05	-3.3356E-01	-1.6430E-01
CCNA_00533	1.83498E-04	1.39218E-04	3.9836E-01	2.5310E-01
CCNA_00534	1.80151E-04	7.26712E-05	1.3096E+00	7.7277E-01
CCNA_00535	1.39744E-04	9.94267E-05	4.9100E-01	3.0593E-01
CCNA_00536	2.51383E-04	5.29705E-05	2.2464E+00	1.3070E+00
CCNA_00537	1.49928E-04	9.61545E-05	6.4076E-01	3.9133E-01
CCNA_00538	1.10333E-04	7.67540E-05	5.2345E-01	3.2444E-01
CCNA_00539	2.66910E-04	6.07983E-05	2.1341E+00	1.2429E+00
CCNA_00540	5.49108E-05	9.10436E-05	-7.2951E-01	-3.9010E-01
CCNA_00541	5.37160E-05	8.31708E-05	-6.3077E-01	-3.3379E-01
CCNA_00542	1.64573E-04	1.39601E-04	2.3737E-01	1.6129E-01
CCNA_00543	3.15771E-05	1.07029E-04	-1.7610E+00	-9.7835E-01
CCNA_00544	3.13394E-05	1.06669E-04	-1.7671E+00	-9.8179E-01
CCNA_00545	1.06367E-04	1.05258E-04	1.5052E-02	3.4506E-02
CCNA_00546	1.35687E-04	6.27871E-05	1.1116E+00	6.5984E-01
CCNA_00547	5.30009E-04	1.73749E-04	1.6090E+00	9.4348E-01
CCNA_00548	6.92006E-04	3.18191E-04	1.1209E+00	6.6513E-01
CCNA_00549	4.95934E-04	6.69246E-04	-4.3240E-01	-2.2066E-01
CCNA_00550	3.79594E-04	4.71699E-04	-3.1342E-01	-1.5282E-01
CCNA_00551	3.38205E-04	1.44967E-04	1.2221E+00	7.2286E-01
CCNA_00552	2.01414E-04	1.25964E-04	6.7708E-01	4.1205E-01
CCNA_00553	1.87627E-04	9.30699E-05	1.0114E+00	6.0269E-01
CCNA_00554	1.69882E-04	8.28406E-05	1.0360E+00	6.1674E-01
CCNA_00555	1.26784E-04	1.31931E-04	-5.7458E-02	-6.8448E-03
CCNA_00556	6.68019E-05	1.11930E-04	-7.4467E-01	-3.9874E-01
CCNA_00557	2.04577E-04	1.29387E-04	6.6089E-01	4.0281E-01
CCNA_00558	1.75913E-05	5.36760E-05	-1.6094E+00	-8.9186E-01
CCNA_00559	1.32382E-04	1.35676E-04	-3.5506E-02	5.6740E-03
CCNA_00560	1.70505E-04	1.00808E-04	7.5813E-01	4.5827E-01
CCNA_00561	1.20747E-04	5.69932E-05	1.0830E+00	6.4351E-01
CCNA_00562	2.37280E-05	1.34115E-04	-2.4987E+00	-1.3990E+00
CCNA_00563	1.10273E-05	9.22519E-05	-3.0642E+00	-1.7215E+00
CCNA_00564	1.55306E-04	1.45868E-04	9.0409E-02	7.7481E-02
CCNA_00565	2.81934E-04	1.41800E-04	9.9144E-01	5.9132E-01
CCNA_00566	2.52328E-05	1.34986E-04	-2.4193E+00	-1.3538E+00
CCNA_00567	1.33496E-04	8.75087E-05	6.0920E-01	3.7334E-01
CCNA_00568	1.00799E-04	7.94633E-05	3.4302E-01	2.2154E-01
CCNA_00569	2.19421E-04	6.28697E-05	1.8031E+00	1.0542E+00
CCNA_00570	1.53365E-04	1.33590E-06	6.8350E+00	3.9237E+00
CCNA_00571	2.27017E-04	2.94197E-06	6.2662E+00	3.5994E+00
CCNA_00572	0.00000E+00	0.00000E+00	-1.3183E+00	-7.2586E-01
CCNA_00573	2.29428E-04	1.20013E-04	9.3478E-01	5.5901E-01
CCNA_00574	2.25663E-05	1.35398E-04	-2.5849E+00	-1.4482E+00
CCNA_00575	6.69735E-05	1.47099E-04	-1.1351E+00	-6.2142E-01
CCNA_00576	6.29165E-05	1.44742E-04	-1.2020E+00	-6.5954E-01
CCNA_00577	1.17562E-04	9.87062E-05	2.5214E-01	1.6971E-01
CCNA_00578	9.07225E-05	7.19207E-05	3.3495E-01	2.1694E-01
CCNA_00579	1.74950E-05	5.36385E-05	-1.6163E+00	-8.9580E-01
CCNA_00580	3.72834E-05	1.47099E-04	-1.9801E+00	-1.1033E+00
CCNA_04005	3.52820E-05	1.50311E-04	-2.0909E+00	-1.1665E+00

CCNA_00582	1.02743E-04	1.19638E-04	-2.1968E-01	-9.9356E-02
CCNA_00583	1.23296E-04	1.37725E-04	-1.5971E-01	-6.5156E-02
CCNA_00584	2.26349E-04	1.11277E-04	1.0243E+00	6.1006E-01
CCNA_00585	1.60408E-04	9.23569E-05	7.9636E-01	4.8007E-01
CCNA_00586	2.25982E-04	1.10091E-04	1.0374E+00	6.1754E-01
CCNA_00587	2.28934E-04	1.16658E-04	9.7257E-01	5.8056E-01
CCNA_00588	1.37164E-04	1.37965E-04	-8.4450E-03	2.1106E-02
CCNA_00589	1.41965E-04	8.46868E-05	7.4523E-01	4.5091E-01
CCNA_00590	1.32767E-04	9.29649E-05	5.1406E-01	3.1908E-01
CCNA_00591	9.30610E-05	1.38130E-04	-5.6981E-01	-2.9903E-01
CCNA_00592	8.14528E-05	1.23413E-04	-5.9949E-01	-3.1595E-01
CCNA_00593	1.94061E-05	1.47099E-04	-2.9220E+00	-1.6405E+00
CCNA_00594	1.57199E-04	1.09611E-04	5.2013E-01	3.2254E-01
CCNA_00595	5.37846E-04	9.97794E-05	2.4303E+00	1.4119E+00
CCNA_00596	7.46360E-05	9.54565E-05	-3.5503E-01	-1.7654E-01
CCNA_00597	1.25944E-04	1.41582E-04	-1.6890E-01	-7.0397E-02
CCNA_00598	1.75958E-04	6.75528E-05	1.3810E+00	8.1348E-01
CCNA_00600	9.55981E-05	6.42356E-05	5.7349E-01	3.5297E-01
CCNA_00601	2.15972E-05	7.79998E-05	-1.8526E+00	-1.0306E+00
CCNA_00602	9.22574E-05	9.18616E-05	6.1324E-03	2.9420E-02
CCNA_00603	3.23506E-05	1.16688E-04	-1.8508E+00	-1.0295E+00
CCNA_00604	5.13775E-05	1.10069E-04	-1.0992E+00	-6.0093E-01
CCNA_00605	8.48297E-05	1.38055E-04	-7.0263E-01	-3.7477E-01
CCNA_00606	1.23443E-04	1.30648E-04	-8.1880E-02	-2.0772E-02
CCNA_00607	1.41959E-04	1.47069E-04	-5.1062E-02	-3.1973E-03
CCNA_00608	4.89518E-05	9.81808E-05	-1.0041E+00	-5.4669E-01
CCNA_00609	4.32882E-04	7.38720E-05	2.5507E+00	1.4806E+00
CCNA_00610	3.10784E-04	6.80106E-05	2.1919E+00	1.2759E+00
CCNA_00611	3.08307E-05	9.09385E-05	-1.5605E+00	-8.6400E-01
CCNA_00612	2.33638E-05	1.47099E-04	-2.6543E+00	-1.4878E+00
CCNA_00613	1.50533E-04	1.44284E-04	6.1120E-02	6.0778E-02
CCNA_00614	1.34925E-04	1.07254E-04	3.3105E-01	2.1471E-01
CCNA_00615	2.45165E-05	1.38070E-04	-2.4935E+00	-1.3961E+00
CCNA_00616	1.40430E-04	7.58684E-05	8.8817E-01	5.3242E-01
CCNA_00617	1.46028E-04	1.11292E-04	3.9182E-01	2.4937E-01
CCNA_00618	2.14918E-05	1.46611E-04	-2.7700E+00	-1.5537E+00
CCNA_00619	2.65992E-05	1.11998E-04	-2.0739E+00	-1.1568E+00
CCNA_00620	1.77451E-04	9.83835E-05	8.5085E-01	5.1114E-01
CCNA_00621	2.83719E-05	1.31848E-04	-2.2163E+00	-1.2380E+00
CCNA_00622	3.83699E-05	1.26595E-04	-1.7221E+00	-9.5618E-01
CCNA_00623	3.18059E-05	1.47654E-04	-2.2148E+00	-1.2371E+00
CCNA_00624	2.30162E-04	8.88146E-05	1.3737E+00	8.0930E-01
CCNA_00625	2.26897E-04	5.51395E-05	2.0407E+00	1.1897E+00
CCNA_00626	2.50182E-04	8.17298E-05	1.6139E+00	9.4631E-01
CCNA_00627	1.50015E-04	8.44616E-05	8.2864E-01	4.9848E-01
CCNA_00628	3.34852E-05	1.44397E-04	-2.1084E+00	-1.1764E+00
CCNA_00629	1.32478E-04	3.93114E-05	1.7525E+00	1.0253E+00
CCNA_00630	9.01025E-05	1.43264E-04	-6.6906E-01	-3.5563E-01
CCNA_00631	1.63475E-04	1.47099E-04	1.5223E-01	1.1274E-01
CCNA_00632	2.71966E-04	1.29259E-04	1.0731E+00	6.3788E-01
CCNA_00633	1.47560E-04	7.23260E-05	1.0286E+00	6.1250E-01
CCNA_00634	2.34066E-04	1.05168E-04	1.1541E+00	6.8410E-01
CCNA_00635	4.93551E-05	1.14512E-04	-1.2142E+00	-6.6653E-01

CCNA_00636	1.84244E-04	1.13551E-04	6.9820E-01	4.2409E-01
CCNA_00637	3.84632E-05	1.46964E-04	-1.9339E+00	-1.0769E+00
CCNA_00638	6.37231E-05	1.47436E-04	-1.2102E+00	-6.6423E-01
CCNA_00639	1.14628E-04	1.08072E-04	8.4899E-02	7.4338E-02
CCNA_00640	2.72905E-04	1.47099E-04	8.9156E-01	5.3436E-01
CCNA_00641	4.12019E-04	1.47421E-04	1.4827E+00	8.7148E-01
CCNA_00642	2.50907E-04	9.23269E-05	1.4422E+00	8.4840E-01
CCNA_00643	9.87252E-05	1.26550E-04	-3.5826E-01	-1.7838E-01
CCNA_00644	6.86529E-05	1.18655E-04	-7.8940E-01	-4.2426E-01
CCNA_00645	8.27049E-05	9.93516E-05	-2.6463E-01	-1.2499E-01
CCNA_00646	1.04401E-04	1.38048E-04	-4.0306E-01	-2.0394E-01
CCNA_00647	1.46037E-04	1.41552E-04	4.4947E-02	5.1555E-02
CCNA_00648	7.99179E-05	5.98301E-05	4.1752E-01	2.6402E-01
CCNA_00649	4.71219E-05	6.75078E-05	-5.1872E-01	-2.6989E-01
CCNA_00650	1.09190E-04	9.97194E-05	1.3082E-01	1.0053E-01
CCNA_00651	4.94754E-05	9.53064E-05	-9.4589E-01	-5.1350E-01
CCNA_00652	1.93589E-04	1.47099E-04	3.9616E-01	2.5184E-01
CCNA_00653	1.50058E-04	1.03847E-04	5.3098E-01	3.2873E-01
CCNA_00654	3.33980E-05	8.74637E-05	-1.3889E+00	-7.6615E-01
CCNA_00655	1.57633E-04	1.18640E-04	4.0992E-01	2.5969E-01
CCNA_00656	1.27494E-04	1.23143E-04	5.0049E-02	5.4464E-02
CCNA_00657	4.94905E-05	9.90815E-05	-1.0015E+00	-5.4520E-01
CCNA_00658	3.18901E-05	7.17781E-05	-1.1704E+00	-6.4156E-01
CCNA_00659	3.00061E-05	5.62352E-05	-9.0627E-01	-4.9090E-01
CCNA_00660	3.80846E-04	3.22251E-04	2.4100E-01	1.6336E-01
CCNA_00661	7.42207E-05	1.54686E-04	-1.0595E+00	-5.7827E-01
CCNA_00662	6.50112E-05	1.30820E-04	-1.0088E+00	-5.4940E-01
CCNA_00663	6.11378E-05	1.14332E-04	-9.0311E-01	-4.8910E-01
CCNA_00664	1.44417E-04	1.30355E-04	1.4775E-01	1.1018E-01
CCNA_00665	2.70253E-04	7.58459E-05	1.8330E+00	1.0713E+00
CCNA_00666	1.37089E-04	4.96308E-05	1.4656E+00	8.6173E-01
CCNA_00667	2.54314E-05	3.31948E-05	-3.8450E-01	-1.9335E-01
CCNA_00668	2.62079E-05	1.45297E-04	-2.4708E+00	-1.3831E+00
CCNA_00669	4.44192E-05	1.47369E-04	-1.7302E+00	-9.6074E-01
CCNA_00670	3.11570E-04	8.93774E-05	1.8015E+00	1.0533E+00
CCNA_00671	1.97134E-04	1.47099E-04	4.2234E-01	2.6678E-01
CCNA_00672	1.16145E-04	5.04038E-05	1.2041E+00	7.1262E-01
CCNA_00673	9.83881E-05	4.92180E-05	9.9912E-01	5.9570E-01
CCNA_00674	2.28040E-05	8.90547E-05	-1.9653E+00	-1.0949E+00
CCNA_00675	2.61056E-05	1.43234E-04	-2.4558E+00	-1.3746E+00
CCNA_00676	3.05839E-05	8.77413E-05	-1.5205E+00	-8.4116E-01
CCNA_00677	3.34491E-05	1.20403E-04	-1.8478E+00	-1.0278E+00
CCNA_00678	2.03003E-04	1.48367E-04	4.5228E-01	2.8385E-01
CCNA_00679	2.60878E-04	7.22592E-04	-1.4698E+00	-8.1227E-01
CCNA_00680	2.31020E-05	1.47309E-04	-2.6726E+00	-1.4982E+00
CCNA_00681	1.21978E-04	1.15780E-04	7.5171E-02	6.8791E-02
CCNA_00682	7.73597E-05	1.25724E-04	-7.0064E-01	-3.7364E-01
CCNA_00683	1.17331E-04	7.08100E-05	7.2844E-01	4.4134E-01
CCNA_00684	1.33158E-04	5.77362E-05	1.2054E+00	7.1336E-01
CCNA_00685	2.61679E-04	7.53806E-05	1.7954E+00	1.0498E+00
CCNA_00686	2.64833E-04	7.56207E-05	1.8081E+00	1.0570E+00
CCNA_00687	1.07570E-04	1.45958E-04	-4.4030E-01	-2.2517E-01
CCNA_00688	1.31825E-04	7.37295E-05	8.3820E-01	5.0393E-01

CCNA_00689	1.31298E-04	7.44950E-05	8.1752E-01	4.9214E-01
CCNA_00690	2.28612E-05	1.46416E-04	-2.6790E+00	-1.5018E+00
CCNA_00691	2.35245E-04	1.07194E-04	1.1339E+00	6.7253E-01
CCNA_00692	2.53637E-04	6.56841E-05	1.9490E+00	1.1374E+00
CCNA_00693	6.47885E-05	1.10894E-04	-7.7541E-01	-4.1628E-01
CCNA_00694	1.46145E-04	2.49843E-05	2.5479E+00	1.4789E+00
CCNA_00695	1.79615E-05	1.12305E-04	-2.6443E+00	-1.4821E+00
CCNA_00696	2.60635E-05	1.47331E-04	-2.4989E+00	-1.3991E+00
CCNA_00697	5.01183E-04	1.47639E-04	1.7632E+00	1.0314E+00
CCNA_00698	3.02261E-04	8.83117E-05	1.7750E+00	1.0382E+00
CCNA_00699	4.22358E-04	1.47099E-04	1.5216E+00	8.9367E-01
CCNA_00700	9.48788E-05	1.09326E-04	-2.0453E-01	-9.0716E-02
CCNA_00701	2.88263E-05	4.10600E-05	-5.1046E-01	-2.6518E-01
CCNA_00702	2.57944E-04	1.47016E-04	8.1103E-01	4.8843E-01
CCNA_00703	1.00164E-04	1.06571E-04	-8.9519E-02	-2.5128E-02
CCNA_00704	3.05210E-04	1.25139E-04	1.2862E+00	7.5941E-01
CCNA_00705	2.00622E-05	9.69951E-05	-2.2733E+00	-1.2705E+00
CCNA_00706	2.15219E-05	6.76354E-05	-1.6519E+00	-9.1614E-01
CCNA_00707	5.75021E-05	5.59650E-05	3.8972E-02	4.8147E-02
CCNA_00708	9.85476E-05	4.53229E-05	1.1204E+00	6.6485E-01
CCNA_00709	8.18351E-05	5.62277E-05	5.4130E-01	3.3461E-01
CCNA_00710	6.64287E-05	1.40892E-04	-1.0847E+00	-5.9267E-01
CCNA_00711	7.91926E-05	1.40036E-04	-8.2239E-01	-4.4307E-01
CCNA_00712	3.28051E-05	6.17514E-05	-9.1260E-01	-4.9451E-01
CCNA_00713	1.60384E-05	1.19390E-04	-2.8959E+00	-1.6255E+00
CCNA_00714	2.97443E-04	8.87545E-05	1.7446E+00	1.0208E+00
CCNA_00715	1.44499E-04	1.44022E-04	4.7266E-03	2.8618E-02
CCNA_00716	2.00776E-04	9.98320E-05	1.0079E+00	6.0072E-01
CCNA_00717	2.26204E-04	7.49678E-05	1.5932E+00	9.3447E-01
CCNA_00718	7.12441E-05	1.15930E-04	-7.0245E-01	-3.7467E-01
CCNA_00719	1.50154E-04	3.89061E-05	1.9481E+00	1.1369E+00
CCNA_00720	9.12071E-05	7.45550E-05	2.9074E-01	1.9173E-01
CCNA_00721	3.88905E-05	1.16163E-04	-1.5786E+00	-8.7434E-01
CCNA_00722	1.47993E-04	1.02474E-04	5.3020E-01	3.2828E-01
CCNA_00723	1.43867E-04	9.12462E-05	6.5681E-01	4.0049E-01
CCNA_00724	1.51577E-04	1.32291E-04	1.9628E-01	1.3786E-01
CCNA_00725	1.62159E-05	1.36457E-04	-3.0728E+00	-1.7264E+00
CCNA_00726	2.09230E-05	1.00102E-04	-2.2582E+00	-1.2619E+00
CCNA_00727	2.15370E-05	8.71635E-05	-2.0168E+00	-1.1242E+00
CCNA_00728	2.39688E-05	9.24920E-05	-1.9481E+00	-1.0850E+00
CCNA_00729	6.38043E-05	8.59702E-05	-4.3024E-01	-2.1943E-01
CCNA_00730	3.34913E-05	1.38671E-04	-2.0498E+00	-1.1430E+00
CCNA_00731	2.04902E-04	1.22265E-04	7.4486E-01	4.5070E-01
CCNA_00732	1.86775E-04	7.42473E-05	1.3308E+00	7.8483E-01
CCNA_00733	9.18090E-05	2.00459E-05	2.1948E+00	1.2776E+00
CCNA_00734	3.48035E-05	1.28426E-04	-1.8836E+00	-1.0483E+00
CCNA_00735	1.15697E-04	7.12528E-05	6.9921E-01	4.2467E-01
CCNA_00736	2.56090E-05	4.98785E-05	-9.6181E-01	-5.2258E-01
CCNA_00737	1.16335E-04	1.35361E-04	-2.1857E-01	-9.8725E-02
CCNA_00738	1.68871E-05	1.25019E-04	-2.8880E+00	-1.6210E+00
CCNA_00739	2.54013E-05	4.47825E-05	-8.1810E-01	-4.4062E-01
CCNA_00740	8.73186E-05	1.47099E-04	-7.5245E-01	-4.0318E-01
CCNA_00741	2.01926E-04	1.28509E-04	6.5190E-01	3.9768E-01

CCNA_00742	2.75972E-04	4.05947E-05	2.7649E+00	1.6027E+00
CCNA_00743	2.57508E-04	1.33470E-04	9.4804E-01	5.6657E-01
CCNA_00744	1.14062E-04	5.63628E-05	1.0169E+00	6.0581E-01
CCNA_00745	1.20867E-05	1.01108E-04	-3.0641E+00	-1.7215E+00
CCNA_00746	2.10946E-05	1.19645E-04	-2.5037E+00	-1.4019E+00
CCNA_00747	4.65465E-04	1.40419E-04	1.7289E+00	1.0119E+00
CCNA_00748	4.52222E-04	1.43001E-04	1.6609E+00	9.7312E-01
CCNA_00749	1.89610E-04	6.98493E-05	1.4406E+00	8.4746E-01
CCNA_00750	1.27160E-04	1.12050E-04	1.8244E-01	1.2996E-01
CCNA_00751	2.15881E-05	1.47414E-04	-2.7714E+00	-1.5546E+00
CCNA_00752	2.69754E-05	7.75495E-05	-1.5235E+00	-8.4287E-01
CCNA_00753	9.37653E-05	7.46976E-05	3.2789E-01	2.1291E-01
CCNA_00754	2.29696E-05	1.30175E-04	-2.5025E+00	-1.4012E+00
CCNA_00755	3.72112E-05	9.47586E-05	-1.3485E+00	-7.4311E-01
CCNA_00756	2.03271E-05	1.21627E-04	-2.5809E+00	-1.4459E+00
CCNA_00757	3.70125E-05	1.40479E-04	-1.9242E+00	-1.0714E+00
CCNA_00758	3.02439E-05	1.47099E-04	-2.2820E+00	-1.2755E+00
CCNA_00759	1.73102E-04	5.95975E-05	1.5381E+00	9.0309E-01
CCNA_00760	3.76114E-04	7.68891E-05	2.2902E+00	1.3320E+00
CCNA_00761	8.10887E-05	1.40952E-04	-7.9766E-01	-4.2896E-01
CCNA_00762	1.29177E-04	1.12921E-04	1.9397E-01	1.3654E-01
CCNA_00763	4.59150E-05	1.06309E-04	-1.2112E+00	-6.6481E-01
CCNA_00764	2.85765E-05	1.20508E-04	-2.0762E+00	-1.1581E+00
CCNA_00765	2.83568E-05	1.23743E-04	-2.1255E+00	-1.1862E+00
CCNA_00766	5.66561E-04	1.48472E-04	1.9320E+00	1.1277E+00
CCNA_00767	6.96942E-05	7.66564E-05	-1.3745E-01	-5.2461E-02
CCNA_00768	3.78101E-05	1.06339E-04	-1.4918E+00	-8.2482E-01
CCNA_00769	2.05709E-05	1.14594E-04	-2.4777E+00	-1.3871E+00
CCNA_00770	4.03984E-05	8.22251E-05	-1.0253E+00	-5.5879E-01
CCNA_00771	1.89806E-04	9.00829E-05	1.0751E+00	6.3903E-01
CCNA_00772	7.20447E-05	1.45080E-04	-1.0099E+00	-5.5000E-01
CCNA_00773	8.75323E-05	9.79857E-05	-1.6282E-01	-6.6928E-02
CCNA_00774	1.65997E-04	6.96467E-05	1.2529E+00	7.4042E-01
CCNA_00775	3.23626E-05	4.68089E-05	-5.3255E-01	-2.7778E-01
CCNA_00776	1.56143E-04	8.67657E-05	8.4757E-01	5.0928E-01
CCNA_00777	1.63162E-04	1.37357E-04	2.4832E-01	1.6753E-01
CCNA_00778	2.44906E-04	1.00808E-04	1.2805E+00	7.5618E-01
CCNA_00779	1.76651E-04	7.75045E-05	1.1884E+00	7.0366E-01
CCNA_00780	1.14830E-04	6.35751E-05	8.5283E-01	5.1227E-01
CCNA_00781	1.13767E-04	7.68515E-05	5.6584E-01	3.4861E-01
CCNA_00782	3.58448E-05	4.45874E-05	-3.1499E-01	-1.5371E-01
CCNA_00783	2.70380E-04	9.29649E-05	1.5401E+00	9.0422E-01
CCNA_00784	1.77427E-04	8.93249E-05	9.9000E-01	5.9049E-01
CCNA_00785	2.38935E-05	1.27105E-04	-2.4112E+00	-1.3492E+00
CCNA_04003	3.29504E-04	1.32029E-04	1.3194E+00	7.7833E-01
CCNA_00787	3.16764E-05	1.26460E-04	-1.9971E+00	-1.1130E+00
CCNA_00788	2.68490E-05	7.92907E-05	-1.5623E+00	-8.6500E-01
CCNA_00789	1.50416E-04	1.21769E-04	3.0475E-01	1.9971E-01
CCNA_00790	1.43298E-04	1.38468E-04	4.9418E-02	5.4105E-02
CCNA_00791	8.12000E-05	9.66798E-05	-2.5179E-01	-1.1767E-01
CCNA_00792	2.24937E-04	6.76879E-05	1.7324E+00	1.0139E+00
CCNA_00793	3.19169E-04	5.05164E-05	2.6593E+00	1.5425E+00
CCNA_00794	4.40340E-05	3.10333E-05	5.0455E-01	3.1366E-01

CCNA_00795	4.40912E-04	1.45913E-04	1.5953E+00	9.3570E-01
CCNA_00796	4.07806E-05	5.02837E-05	-3.0232E-01	-1.4648E-01
CCNA_00797	2.80438E-05	5.58074E-05	-9.9281E-01	-5.4026E-01
CCNA_00798	6.08308E-05	6.91964E-05	-1.8598E-01	-8.0138E-02
CCNA_00799	3.01174E-05	1.43226E-04	-2.2496E+00	-1.2569E+00
CCNA_00800	2.51877E-05	1.47099E-04	-2.5459E+00	-1.4259E+00
CCNA_00801	1.21791E-04	1.25372E-04	-4.1853E-02	2.0544E-03
CCNA_00802	4.90661E-05	1.47099E-04	-1.5840E+00	-8.7738E-01
CCNA_00803	1.54467E-04	9.87512E-05	6.4534E-01	3.9395E-01
CCNA_00804	1.41606E-04	7.05698E-05	1.0046E+00	5.9885E-01
CCNA_00805	9.46050E-05	1.34925E-04	-5.1221E-01	-2.6618E-01
CCNA_00806	3.29375E-05	1.05521E-04	-1.6797E+00	-9.3197E-01
CCNA_00807	9.02500E-05	9.10586E-05	-1.2938E-02	1.8544E-02
CCNA_00808	1.81580E-04	4.50152E-05	2.0119E+00	1.1733E+00
CCNA_00809	2.85675E-05	5.30606E-05	-8.9332E-01	-4.8352E-01
CCNA_00810	5.60936E-05	4.06548E-05	4.6422E-01	2.9066E-01
CCNA_00811	7.84793E-05	1.47969E-04	-9.1493E-01	-4.9584E-01
CCNA_00812	8.81463E-05	4.76269E-05	8.8794E-01	5.3230E-01
CCNA_00813	3.44272E-05	1.45755E-04	-2.0819E+00	-1.1613E+00
CCNA_00814	8.07456E-05	5.64979E-05	5.1505E-01	3.1964E-01
CCNA_00815	2.34391E-05	3.17238E-05	-4.3681E-01	-2.2318E-01
CCNA_00816	3.90350E-05	1.05326E-04	-1.4320E+00	-7.9072E-01
CCNA_00817	2.69272E-05	8.92799E-05	-1.7292E+00	-9.6022E-01
CCNA_00818	3.25161E-05	4.76795E-05	-5.5231E-01	-2.8905E-01
CCNA_00819	3.09752E-05	5.41413E-05	-8.0568E-01	-4.3354E-01
CCNA_00820	3.33528E-05	5.79088E-05	-7.9603E-01	-4.2804E-01
CCNA_00821	7.95959E-05	7.18532E-05	1.4755E-01	1.1006E-01
CCNA_00822	4.69558E-04	1.09964E-04	2.0942E+00	1.2202E+00
CCNA_00823	1.89126E-05	1.47609E-04	-2.9642E+00	-1.6645E+00
CCNA_00824	1.65976E-04	1.03269E-04	6.8448E-01	4.1627E-01
CCNA_00825	3.60555E-05	3.15136E-05	1.9402E-01	1.3657E-01
CCNA_00826	2.15908E-04	1.34213E-04	6.8584E-01	4.1704E-01
CCNA_00827	2.68920E-04	1.26512E-04	1.0878E+00	6.4629E-01
CCNA_00828	9.05359E-05	8.97377E-05	1.2704E-02	3.3167E-02
CCNA_00829	2.29876E-05	1.47654E-04	-2.6832E+00	-1.5042E+00
CCNA_00830	2.80288E-05	1.13709E-04	-2.0203E+00	-1.1262E+00
CCNA_00831	4.35796E-05	7.66264E-05	-8.1423E-01	-4.3842E-01
CCNA_00832	3.37170E-05	1.47099E-04	-2.1252E+00	-1.1860E+00
CCNA_00833	2.86758E-05	1.40104E-04	-2.2885E+00	-1.2792E+00
CCNA_00834	3.46819E-04	7.16956E-05	2.2741E+00	1.3228E+00
CCNA_00835	6.19865E-05	9.89914E-05	-6.7539E-01	-3.5924E-01
CCNA_00836	2.21858E-04	1.41530E-04	6.4848E-01	3.9573E-01
CCNA_00837	6.50894E-05	1.06729E-04	-7.1349E-01	-3.8097E-01
CCNA_00838	8.61388E-05	9.89389E-05	-1.9993E-01	-8.8094E-02
CCNA_00839	9.04667E-05	9.40681E-05	-5.6386E-02	-6.2330E-03
CCNA_00840	1.36024E-04	2.31080E-05	2.5570E+00	1.4841E+00
CCNA_00841	1.14676E-04	4.94582E-05	1.2131E+00	7.1773E-01
CCNA_00842	1.31518E-04	6.84609E-05	9.4178E-01	5.6300E-01
CCNA_00843	2.11758E-05	1.33845E-04	-2.6599E+00	-1.4910E+00
CCNA_00844	1.01482E-04	8.73136E-05	2.1686E-01	1.4960E-01
CCNA_00845	1.08633E-04	1.17416E-04	-1.1222E-01	-3.8076E-02
CCNA_00846	5.07305E-05	1.05251E-04	-1.0529E+00	-5.7454E-01
CCNA_00847	6.21099E-05	4.23884E-05	5.5097E-01	3.4013E-01

CCNA_00848	1.85454E-05	8.22777E-05	-2.1493E+00	-1.1998E+00
CCNA_00849	4.86237E-05	1.43399E-04	-1.5603E+00	-8.6387E-01
CCNA_00850	3.80840E-05	1.26798E-04	-1.7352E+00	-9.6365E-01
CCNA_00851	1.19440E-04	1.44772E-05	3.0437E+00	1.7617E+00
CCNA_00852	1.15525E-04	1.53703E-05	2.9093E+00	1.6850E+00
CCNA_00853	7.94544E-05	1.38723E-04	-8.0403E-01	-4.3260E-01
CCNA_00854	1.04976E-04	1.45207E-04	-4.6809E-01	-2.4102E-01
CCNA_00855	5.11368E-05	9.54715E-05	-9.0074E-01	-4.8775E-01
CCNA_00856	2.54164E-05	6.30723E-05	-1.3112E+00	-7.2185E-01
CCNA_00857	2.39044E-04	8.95426E-05	1.4165E+00	8.3373E-01
CCNA_00858	2.29729E-04	1.00185E-04	1.1972E+00	7.0865E-01
CCNA_00859	8.00052E-05	6.59017E-05	2.7967E-01	1.8541E-01
CCNA_00860	9.38104E-05	7.89980E-05	2.4784E-01	1.6726E-01
CCNA_00861	1.19982E-04	1.49223E-04	-3.1468E-01	-1.5353E-01
CCNA_00862	1.42681E-04	1.33132E-04	9.9886E-02	8.2885E-02
CCNA_00863	4.92286E-05	8.63679E-05	-8.1103E-01	-4.3659E-01
CCNA_00864	6.15501E-05	1.08448E-04	-8.1720E-01	-4.4011E-01
CCNA_00865	2.25091E-05	7.12228E-05	-1.6618E+00	-9.2176E-01
CCNA_00866	1.87621E-05	3.94690E-05	-1.0729E+00	-5.8595E-01
CCNA_00867	1.87169E-05	3.41704E-05	-8.6849E-01	-4.6936E-01
CCNA_04017	1.78772E-05	1.13078E-04	-2.6610E+00	-1.4916E+00
CCNA_00869	1.47987E-04	1.14107E-04	3.7502E-01	2.3979E-01
CCNA_00870	1.10776E-04	1.43023E-04	-3.6865E-01	-1.8431E-01
CCNA_00871	1.50431E-04	7.38796E-05	1.0257E+00	6.1088E-01
CCNA_00872	1.61253E-04	7.98010E-05	1.0147E+00	6.0461E-01
CCNA_00873	1.61828E-05	6.15038E-05	-1.9261E+00	-1.0725E+00
CCNA_00874	1.49308E-05	4.83774E-05	-1.6960E+00	-9.4125E-01
CCNA_00875	1.50906E-04	1.06279E-04	5.0573E-01	3.1433E-01
CCNA_00876	4.74349E-05	4.85726E-05	-3.4324E-02	6.3480E-03
CCNA_00877	1.06321E-04	6.28246E-05	7.5890E-01	4.5871E-01
CCNA_00878	1.20455E-04	7.47952E-05	6.8737E-01	4.1791E-01
CCNA_00879	1.33489E-04	1.94005E-05	2.7820E+00	1.6125E+00
CCNA_00880	1.42807E-05	1.25417E-04	-3.1344E+00	-1.7615E+00
CCNA_00881	4.56141E-05	1.02271E-04	-1.1649E+00	-6.3837E-01
CCNA_00882	1.28803E-04	1.32959E-04	-4.5860E-02	-2.3023E-04
CCNA_00883	7.25624E-05	8.26304E-05	-1.8752E-01	-8.1017E-02
CCNA_00884	1.86667E-04	1.14377E-04	7.0660E-01	4.2888E-01
CCNA_00885	1.94188E-04	1.36539E-04	5.0808E-01	3.1567E-01
CCNA_00886	1.95416E-04	1.39158E-04	4.8976E-01	3.0522E-01
CCNA_00887	2.14286E-05	8.46568E-05	-1.9820E+00	-1.1044E+00
CCNA_00888	3.87280E-05	6.49035E-05	-7.4497E-01	-3.9892E-01
CCNA_00889	3.12921E-04	1.47391E-04	1.0861E+00	6.4529E-01
CCNA_00890	3.10926E-05	4.63211E-05	-5.7519E-01	-3.0210E-01
CCNA_00891	2.16724E-05	7.84201E-05	-1.8553E+00	-1.0321E+00
CCNA_00892	1.09596E-04	8.55574E-05	3.5714E-01	2.2959E-01
CCNA_00893	1.52396E-04	1.71265E-05	3.1529E+00	1.8240E+00
CCNA_00894	1.88253E-05	1.41695E-04	-2.9119E+00	-1.6347E+00
CCNA_00895	1.36752E-04	1.47099E-04	-1.0526E-01	-3.4108E-02
CCNA_00896	7.33569E-05	5.59350E-05	3.9105E-01	2.4893E-01
CCNA_00897	1.44014E-04	6.19315E-05	1.2173E+00	7.2013E-01
CCNA_00898	5.19494E-05	5.50719E-05	-8.4324E-02	-2.2166E-02
CCNA_00899	1.13828E-04	8.87395E-05	3.5912E-01	2.3072E-01
CCNA_00900	1.13505E-04	8.91673E-05	3.4809E-01	2.2443E-01

CCNA_00901	2.98180E-04	6.02354E-05	2.3073E+00	1.3417E+00
CCNA_00902	2.30647E-04	2.23950E-05	3.3640E+00	1.9443E+00
CCNA_00903	2.17835E-04	1.47099E-04	5.6640E-01	3.4892E-01
CCNA_00904	3.85264E-05	7.13128E-05	-8.8835E-01	-4.8069E-01
CCNA_00905	2.54826E-05	6.59392E-05	-1.3716E+00	-7.5628E-01
CCNA_00906	3.02890E-05	1.31969E-04	-2.1233E+00	-1.1849E+00
CCNA_00907	9.74942E-05	8.55199E-05	1.8898E-01	1.3369E-01
CCNA_00908	3.15229E-05	7.09301E-05	-1.1700E+00	-6.4131E-01
CCNA_00909	8.63525E-05	4.64937E-05	8.9302E-01	5.3519E-01
CCNA_00910	1.97312E-05	7.06524E-05	-1.8402E+00	-1.0235E+00
CCNA_00911	1.42753E-04	6.59242E-05	1.1145E+00	6.6150E-01
CCNA_00912	1.89968E-04	4.35442E-05	2.1250E+00	1.2378E+00
CCNA_00913	1.59992E-04	1.41920E-04	1.7287E-01	1.2451E-01
CCNA_00914	1.12780E-04	1.43316E-04	-3.4572E-01	-1.7124E-01
CCNA_00915	1.07026E-04	1.12343E-04	-7.0006E-02	-1.4001E-02
CCNA_00916	3.46560E-05	1.03870E-04	-1.5836E+00	-8.7716E-01
CCNA_00917	5.35595E-05	1.21079E-04	-1.1767E+00	-6.4515E-01
CCNA_00918	5.36107E-05	1.47099E-04	-1.4562E+00	-8.0451E-01
CCNA_00919	2.84317E-04	4.74393E-05	2.5831E+00	1.4990E+00
CCNA_00920	2.69790E-04	1.46686E-04	8.7905E-01	5.2722E-01
CCNA_00921	3.31632E-05	1.39196E-04	-2.0694E+00	-1.1542E+00
CCNA_00922	1.72708E-04	6.81832E-05	1.3407E+00	7.9050E-01
CCNA_00923	3.66574E-05	1.16373E-04	-1.6666E+00	-9.2448E-01
CCNA_00924	1.35121E-04	5.61377E-05	1.2670E+00	7.4849E-01
CCNA_00925	1.59303E-04	1.16396E-04	4.5267E-01	2.8407E-01
CCNA_00926	2.08929E-05	1.30152E-04	-2.6390E+00	-1.4790E+00
CCNA_00927	1.74980E-05	1.15825E-04	-2.7265E+00	-1.5290E+00
CCNA_00928	1.70556E-05	1.28591E-04	-2.9143E+00	-1.6360E+00
CCNA_00929	1.94513E-05	8.92574E-05	-2.1980E+00	-1.2275E+00
CCNA_00930	3.35755E-05	1.16553E-04	-1.7955E+00	-9.9799E-01
CCNA_00931	2.96479E-05	1.47099E-04	-2.3107E+00	-1.2918E+00
CCNA_00932	3.17396E-05	1.32494E-04	-2.0615E+00	-1.1497E+00
CCNA_00933	1.18926E-04	8.28105E-05	5.2208E-01	3.2365E-01
CCNA_00934	1.49910E-05	9.39855E-05	-2.6482E+00	-1.4843E+00
CCNA_00935	1.87591E-05	1.47031E-04	-2.9703E+00	-1.6680E+00
CCNA_00936	1.86116E-05	1.27173E-04	-2.7724E+00	-1.5551E+00
CCNA_00937	1.36951E-04	1.12545E-04	2.8308E-01	1.8736E-01
CCNA_00938	5.26115E-05	1.38490E-04	-1.3963E+00	-7.7037E-01
CCNA_00939	5.25513E-05	1.46881E-04	-1.4828E+00	-8.1971E-01
CCNA_00940	1.30046E-04	1.47099E-04	-1.7780E-01	-7.5471E-02
CCNA_00941	1.67059E-04	8.90322E-05	9.0786E-01	5.4366E-01
CCNA_00942	1.69846E-04	7.70542E-05	1.1402E+00	6.7613E-01
CCNA_00943	3.12431E-05	1.46994E-04	-2.2341E+00	-1.2481E+00
CCNA_00944	2.03692E-05	4.93906E-05	-1.2779E+00	-7.0281E-01
CCNA_00945	1.06481E-05	3.39753E-05	-1.6738E+00	-9.2861E-01
CCNA_00946	5.28733E-05	7.05698E-05	-4.1658E-01	-2.1165E-01
CCNA_00947	2.45015E-05	1.23120E-04	-2.3290E+00	-1.3023E+00
CCNA_00948	2.10073E-05	1.03344E-04	-2.2984E+00	-1.2848E+00
CCNA_00949	2.28672E-05	8.46643E-05	-1.8884E+00	-1.0510E+00
CCNA_00950	2.33999E-05	5.43515E-05	-1.2158E+00	-6.6744E-01
CCNA_00951	2.92690E-04	9.02705E-05	1.6969E+00	9.9365E-01
CCNA_00952	2.14377E-05	9.03081E-05	-2.0746E+00	-1.1572E+00
CCNA_00953	3.77529E-05	7.48777E-05	-9.8798E-01	-5.3750E-01

CCNA_00954	1.41026E-04	1.47099E-04	-6.0870E-02	-8.7902E-03
CCNA_00955	2.13838E-04	1.39984E-04	6.1120E-01	3.7448E-01
CCNA_00956	1.18228E-04	5.89971E-05	1.0027E+00	5.9774E-01
CCNA_00957	2.46459E-05	5.31506E-05	-1.1088E+00	-6.0638E-01
CCNA_00958	2.52662E-04	4.99385E-05	2.3388E+00	1.3597E+00
CCNA_00959	2.76769E-04	5.52896E-05	2.3234E+00	1.3509E+00
CCNA_00960	7.61860E-05	4.40771E-05	7.8931E-01	4.7605E-01
CCNA_00961	8.38305E-05	3.99643E-05	1.0685E+00	6.3529E-01
CCNA_00962	1.55087E-04	1.40599E-04	1.4144E-01	1.0658E-01
CCNA_00963	9.32446E-05	1.02144E-04	-1.3156E-01	-4.9106E-02
CCNA_00964	9.79126E-05	4.85351E-05	1.0123E+00	6.0321E-01
CCNA_00965	1.85788E-04	3.57765E-05	2.3763E+00	1.3811E+00
CCNA_00966	1.52273E-04	3.69398E-05	2.0431E+00	1.1911E+00
CCNA_00967	1.21355E-04	9.92165E-05	2.9050E-01	1.9159E-01
CCNA_00968	7.28874E-05	6.66372E-05	1.2924E-01	9.9625E-02
CCNA_00969	3.44874E-05	1.26242E-04	-1.8720E+00	-1.0416E+00
CCNA_00970	3.34973E-05	1.42258E-04	-2.0863E+00	-1.1639E+00
CCNA_00971	8.30299E-05	5.05164E-05	7.1672E-01	4.3465E-01
CCNA_00972	6.25794E-05	4.75669E-05	3.9557E-01	2.5151E-01
CCNA_00973	1.60013E-04	6.03630E-05	1.4063E+00	8.2791E-01
CCNA_00974	4.25563E-05	8.45892E-05	-9.9113E-01	-5.3930E-01
CCNA_00975	2.48235E-05	8.71560E-05	-1.8118E+00	-1.0073E+00
CCNA_00976	7.79887E-05	9.25296E-05	-2.4671E-01	-1.1477E-01
CCNA_00977	1.88352E-03	3.00472E-04	2.6481E+00	1.5361E+00
CCNA_00978	1.21531E-03	5.56934E-04	1.1257E+00	6.6790E-01
CCNA_00979	1.12476E-04	1.22640E-04	-1.2486E-01	-4.5281E-02
CCNA_00980	6.68380E-05	1.22692E-04	-8.7633E-01	-4.7383E-01
CCNA_00981	6.44725E-05	1.33147E-04	-1.0463E+00	-5.7075E-01
CCNA_00982	4.20627E-05	1.08876E-04	-1.3721E+00	-7.5653E-01
CCNA_00983	1.80109E-04	1.47099E-04	2.9203E-01	1.9246E-01
CCNA_00984	4.68841E-05	1.46191E-04	-1.6407E+00	-9.0971E-01
CCNA_00985	2.93289E-05	1.03329E-04	-1.8168E+00	-1.0102E+00
CCNA_00986	2.18620E-05	5.50794E-05	-1.3331E+00	-7.3431E-01
CCNA_00987	1.92533E-04	7.93807E-05	1.2781E+00	7.5481E-01
CCNA_00988	6.43581E-05	7.82850E-05	-2.8269E-01	-1.3529E-01
CCNA_00989	1.12356E-04	1.16208E-04	-4.8689E-02	-1.8435E-03
CCNA_00990	1.54476E-04	1.01663E-04	6.0350E-01	3.7009E-01
CCNA_00991	8.06764E-05	7.45925E-05	1.1302E-01	9.0377E-02
CCNA_00992	4.46450E-05	7.11402E-05	-6.7222E-01	-3.5743E-01
CCNA_00993	1.92075E-05	9.96068E-05	-2.3745E+00	-1.3282E+00
CCNA_00994	2.70145E-05	1.46506E-04	-2.4391E+00	-1.3650E+00
CCNA_00995	2.72643E-05	1.34865E-04	-2.3064E+00	-1.2893E+00
CCNA_00996	2.66684E-05	3.47408E-05	-3.8165E-01	-1.9172E-01
CCNA_00997	1.31720E-04	9.76780E-05	4.3129E-01	2.7188E-01
CCNA_00998	1.46034E-04	3.24142E-05	2.1713E+00	1.2642E+00
CCNA_00999	7.50408E-04	3.09057E-05	4.6014E+00	2.6500E+00
CCNA_01000	1.59592E-04	1.13243E-04	4.9489E-01	3.0815E-01
CCNA_01001	1.36388E-04	1.28989E-04	8.0414E-02	7.1781E-02
CCNA_01002	1.76100E-04	8.88221E-05	9.8731E-01	5.8896E-01
CCNA_01003	2.26969E-04	7.99136E-05	1.5059E+00	8.8468E-01
CCNA_01004	2.54913E-04	4.72817E-05	2.4304E+00	1.4119E+00
CCNA_01005	2.61056E-04	7.95158E-05	1.7149E+00	1.0039E+00
CCNA_01006	2.05570E-04	1.47099E-04	4.8280E-01	3.0125E-01

CCNA_01007	1.39440E-04	5.30981E-05	1.3927E+00	8.2017E-01
CCNA_01008	1.89487E-05	8.26379E-05	-2.1246E+00	-1.1857E+00
CCNA_01009	1.31256E-04	6.65396E-05	9.7997E-01	5.8478E-01
CCNA_01010	2.11939E-05	7.16881E-05	-1.7580E+00	-9.7664E-01
CCNA_01011	1.38476E-04	1.37597E-04	9.1413E-03	3.1136E-02
CCNA_01012	3.33709E-05	1.47099E-04	-2.1401E+00	-1.1945E+00
CCNA_01013	7.98638E-05	1.13431E-04	-5.0624E-01	-2.6278E-01
CCNA_01014	1.70794E-04	7.08700E-05	1.2689E+00	7.4954E-01
CCNA_01015	1.90652E-04	1.25462E-04	6.0363E-01	3.7016E-01
CCNA_01016	4.25202E-05	1.08688E-04	-1.3540E+00	-7.4622E-01
CCNA_01017	1.12281E-04	1.47384E-04	-3.9251E-01	-1.9791E-01
CCNA_01018	7.62793E-05	6.60443E-05	2.0775E-01	1.4440E-01
CCNA_01019	1.19082E-04	3.89661E-05	1.6114E+00	9.4488E-01
CCNA_01020	3.33137E-05	1.14144E-04	-1.7766E+00	-9.8725E-01
CCNA_01021	1.40351E-04	9.03006E-05	6.3615E-01	3.8870E-01
CCNA_01022	9.00785E-05	1.03239E-04	-1.9679E-01	-8.6305E-02
CCNA_01023	9.79908E-05	1.33237E-04	-4.4331E-01	-2.2689E-01
CCNA_01024	1.29478E-04	1.47099E-04	-1.8412E-01	-7.9078E-02
CCNA_01025	1.75402E-04	1.20058E-04	5.4687E-01	3.3779E-01
CCNA_01026	3.90982E-05	8.84093E-05	-1.1771E+00	-6.4536E-01
CCNA_01027	3.68049E-05	1.40367E-04	-1.9312E+00	-1.0754E+00
CCNA_01028	1.86206E-05	1.13153E-04	-2.6032E+00	-1.4586E+00
CCNA_01029	1.74132E-04	6.66447E-05	1.3855E+00	8.1603E-01
CCNA_01030	2.09350E-05	3.84783E-05	-8.7820E-01	-4.7490E-01
CCNA_01031	2.00291E-05	4.29588E-05	-1.1009E+00	-6.0189E-01
CCNA_01032	5.51546E-05	8.61278E-05	-6.4304E-01	-3.4079E-01
CCNA_01033	1.07917E-04	1.32231E-04	-2.9319E-01	-1.4128E-01
CCNA_01034	9.78012E-05	8.24728E-05	2.4585E-01	1.6612E-01
CCNA_01035	2.53261E-05	6.07007E-05	-1.2611E+00	-6.9325E-01
CCNA_01036	2.33247E-05	1.37973E-04	-2.5643E+00	-1.4365E+00
CCNA_01037	1.37586E-04	8.00562E-05	7.8114E-01	4.7139E-01
CCNA_01038	2.28251E-05	5.51020E-05	-1.2715E+00	-6.9918E-01
CCNA_01039	2.21509E-05	6.73277E-05	-1.6038E+00	-8.8869E-01
CCNA_01040	2.08387E-05	5.72484E-05	-1.4579E+00	-8.0551E-01
CCNA_01041	1.62331E-04	8.22251E-05	9.8118E-01	5.8547E-01
CCNA_01042	2.47001E-05	1.43534E-04	-2.5387E+00	-1.4218E+00
CCNA_01043	2.27318E-05	6.86110E-05	-1.5937E+00	-8.8293E-01
CCNA_01044	2.35835E-05	9.04131E-05	-1.9387E+00	-1.0797E+00
CCNA_01045	1.85466E-04	1.25687E-04	5.6126E-01	3.4600E-01
CCNA_01046	1.46169E-04	1.12553E-04	3.7697E-01	2.4090E-01
CCNA_01047	2.11156E-04	4.61935E-05	2.1923E+00	1.2762E+00
CCNA_01048	1.34660E-04	3.23542E-05	2.0570E+00	1.1990E+00
CCNA_01049	2.22864E-05	6.55940E-05	-1.5574E+00	-8.6221E-01
CCNA_01050	2.98535E-04	6.89562E-05	2.1140E+00	1.2315E+00
CCNA_01051	1.18213E-04	1.45500E-04	-2.9967E-01	-1.4498E-01
CCNA_01052	2.16754E-05	6.14662E-05	-1.5037E+00	-8.3161E-01
CCNA_01053	5.53623E-05	7.94258E-05	-5.2076E-01	-2.7106E-01
CCNA_01054	1.30817E-04	1.13499E-04	2.0481E-01	1.4272E-01
CCNA_01055	3.91975E-05	5.59725E-05	-5.1404E-01	-2.6722E-01
CCNA_01056	2.47206E-04	1.18865E-04	1.0563E+00	6.2832E-01
CCNA_01057	2.39868E-05	1.13251E-04	-2.2391E+00	-1.2510E+00
CCNA_01058	6.87973E-05	2.76185E-05	1.3164E+00	7.7663E-01
CCNA_01059	4.57706E-05	4.65312E-07	6.5971E+00	3.7881E+00

CCNA_01060	7.24962E-05	9.89013E-05	-4.4813E-01	-2.2964E-01
CCNA_01061	1.74135E-04	8.24428E-05	1.0786E+00	6.4104E-01
CCNA_01062	1.56519E-04	1.33612E-04	2.2824E-01	1.5608E-01
CCNA_01063	2.10855E-05	1.47399E-04	-2.8053E+00	-1.5739E+00
CCNA_01064	1.13147E-04	1.43226E-04	-3.4013E-01	-1.6805E-01
CCNA_01065	2.29124E-05	1.47234E-04	-2.6838E+00	-1.5046E+00
CCNA_01066	1.51288E-04	1.47099E-04	4.0473E-02	4.9003E-02
CCNA_01067	1.09587E-04	1.47099E-04	-4.2474E-01	-2.1630E-01
CCNA_01068	2.23014E-05	5.58825E-05	-1.3253E+00	-7.2984E-01
CCNA_01069	1.51114E-05	1.22572E-04	-3.0197E+00	-1.6962E+00
CCNA_01070	3.74059E-04	7.39621E-05	2.3383E+00	1.3594E+00
CCNA_01071	2.64315E-04	1.00597E-04	1.3936E+00	8.2065E-01
CCNA_01072	2.54426E-04	4.00469E-05	2.6672E+00	1.5470E+00
CCNA_01073	4.53462E-05	9.43458E-05	-1.0570E+00	-5.7686E-01
CCNA_01074	3.02348E-05	9.03681E-05	-1.5796E+00	-8.7488E-01
CCNA_01075	2.36407E-05	5.50869E-05	-1.2205E+00	-6.7007E-01
CCNA_01076	4.84070E-05	6.27496E-05	-3.7447E-01	-1.8763E-01
CCNA_01077	4.56382E-05	1.47084E-04	-1.6883E+00	-9.3688E-01
CCNA_01078	4.13404E-05	1.31646E-04	-1.6710E+00	-9.2702E-01
CCNA_01079	4.28121E-05	1.07517E-04	-1.3285E+00	-7.3168E-01
CCNA_01080	2.59581E-05	6.12186E-05	-1.2378E+00	-6.7996E-01
CCNA_01081	1.65572E-04	3.38327E-05	2.2907E+00	1.3322E+00
CCNA_01082	8.42277E-05	6.29222E-05	4.2061E-01	2.6578E-01
CCNA_01083	8.71952E-05	6.88287E-05	3.4113E-01	2.2046E-01
CCNA_01084	3.83669E-05	8.66831E-05	-1.1759E+00	-6.4467E-01
CCNA_01085	2.20447E-04	1.18392E-04	8.9679E-01	5.3734E-01
CCNA_01086	2.36798E-05	1.02811E-04	-2.1182E+00	-1.1820E+00
CCNA_01087	2.31772E-05	1.06684E-04	-2.2025E+00	-1.2301E+00
CCNA_01088	5.43179E-05	7.74444E-05	-5.1179E-01	-2.6594E-01
CCNA_01089	1.07396E-04	1.36351E-04	-3.4443E-01	-1.7050E-01
CCNA_01090	1.41838E-04	6.03705E-05	1.2322E+00	7.2861E-01
CCNA_01091	3.75302E-05	4.29963E-05	-1.9630E-01	-8.6022E-02
CCNA_01092	2.44142E-05	1.47076E-04	-2.5907E+00	-1.4515E+00
CCNA_01093	2.21058E-05	5.63478E-05	-1.3499E+00	-7.4391E-01
CCNA_01094	2.72463E-05	7.08175E-05	-1.3780E+00	-7.5994E-01
CCNA_01095	2.40952E-05	1.32471E-04	-2.4588E+00	-1.3763E+00
CCNA_01096	1.69834E-05	1.23488E-04	-2.8620E+00	-1.6062E+00
CCNA_01097	7.37933E-05	1.47331E-04	-9.9752E-01	-5.4294E-01
CCNA_01098	1.16545E-04	7.10952E-05	7.1295E-01	4.3250E-01
CCNA_01099	1.71920E-04	9.29799E-05	8.8665E-01	5.3156E-01
CCNA_01100	2.35474E-05	1.20471E-04	-2.3549E+00	-1.3171E+00
CCNA_01101	1.27479E-04	7.49453E-05	7.6624E-01	4.6289E-01
CCNA_01102	1.28174E-04	4.43322E-05	1.5315E+00	8.9929E-01
CCNA_01103	3.89989E-05	9.92090E-05	-1.3470E+00	-7.4226E-01
CCNA_01104	4.32533E-04	1.30760E-04	1.7258E+00	1.0101E+00
CCNA_01105	3.61774E-04	1.35203E-04	1.4199E+00	8.3565E-01
CCNA_01106	1.56239E-04	6.67948E-05	1.2258E+00	7.2498E-01
CCNA_01107	3.65966E-04	8.07692E-05	2.1797E+00	1.2690E+00
CCNA_01108	4.03749E-04	1.46025E-04	1.4672E+00	8.6262E-01
CCNA_01109	6.08693E-04	7.28439E-05	3.0627E+00	1.7725E+00
CCNA_01110	3.81833E-05	1.28103E-04	-1.7463E+00	-9.6993E-01
CCNA_01111	1.32051E-04	1.30835E-04	1.3293E-02	3.3503E-02
CCNA_01112	2.22593E-05	3.77803E-05	-7.6332E-01	-4.0938E-01

CCNA_01113	1.48496E-05	2.42413E-05	-7.0720E-01	-3.7738E-01
CCNA_01114	4.88103E-05	1.26790E-04	-1.3772E+00	-7.5945E-01
CCNA_01115	3.80596E-04	2.33932E-05	4.0236E+00	2.3205E+00
CCNA_01116	4.05248E-04	4.35592E-05	3.2175E+00	1.8608E+00
CCNA_01117	1.29255E-04	3.41479E-05	1.9201E+00	1.1209E+00
CCNA_01118	1.10484E-04	4.12927E-05	1.4197E+00	8.3552E-01
CCNA_01119	1.27425E-04	1.43819E-04	-1.7465E-01	-7.3674E-02
CCNA_01120	4.31420E-04	1.29852E-04	1.7321E+00	1.0137E+00
CCNA_01121	4.83330E-04	5.48768E-05	3.1386E+00	1.8158E+00
CCNA_01122	5.40561E-05	6.31549E-05	-2.2453E-01	-1.0212E-01
CCNA_01123	5.32315E-05	1.14955E-04	-1.1107E+00	-6.0750E-01
CCNA_01124	1.77569E-05	9.21618E-05	-2.3757E+00	-1.3289E+00
CCNA_01125	1.15359E-04	1.46146E-04	-3.4131E-01	-1.6872E-01
CCNA_01126	3.84963E-05	5.43364E-05	-4.9729E-01	-2.5767E-01
CCNA_01127	1.21015E-04	6.93090E-05	8.0395E-01	4.8439E-01
CCNA_01128	4.64929E-05	8.62478E-05	-8.9151E-01	-4.8249E-01
CCNA_01129	3.50424E-04	1.47099E-04	1.2523E+00	7.4006E-01
CCNA_01130	3.43978E-04	5.26103E-05	2.7087E+00	1.5706E+00
CCNA_01131	3.07483E-04	1.47099E-04	1.0637E+00	6.3250E-01
CCNA_01132	1.10369E-04	1.00597E-04	1.3368E-01	1.0216E-01
CCNA_01133	9.27691E-05	1.11450E-04	-2.6473E-01	-1.2505E-01
CCNA_01134	6.49329E-05	7.13879E-05	-1.3681E-01	-5.2099E-02
CCNA_01135	8.91515E-05	8.19775E-05	1.2095E-01	9.4896E-02
CCNA_01136	1.10418E-04	1.47039E-04	-4.1326E-01	-2.0975E-01
CCNA_01137	1.21580E-04	8.05515E-05	5.9383E-01	3.6457E-01
CCNA_01138	1.84452E-04	2.20948E-05	3.0610E+00	1.7715E+00
CCNA_01139	2.42276E-05	9.23795E-05	-1.9309E+00	-1.0752E+00
CCNA_01140	2.99615E-04	9.66498E-05	1.6322E+00	9.5672E-01
CCNA_01141	2.88979E-04	1.06144E-04	1.4449E+00	8.4989E-01
CCNA_01142	1.77578E-04	1.00117E-04	8.2668E-01	4.9736E-01
CCNA_01143	3.56522E-05	7.96209E-05	-1.1592E+00	-6.3513E-01
CCNA_01144	9.29858E-05	1.40277E-04	-5.9322E-01	-3.1238E-01
CCNA_01145	1.80638E-04	4.44898E-05	2.0213E+00	1.1786E+00
CCNA_01146	5.80379E-05	8.15947E-05	-4.9154E-01	-2.5439E-01
CCNA_01147	1.93616E-04	6.78905E-05	1.5118E+00	8.8806E-01
CCNA_01148	1.68525E-04	8.87620E-05	9.2485E-01	5.5334E-01
CCNA_01149	1.37832E-04	4.92555E-05	1.4844E+00	8.7242E-01
CCNA_01150	2.94439E-04	4.98935E-05	2.5608E+00	1.4863E+00
CCNA_01151	7.62552E-05	4.79422E-05	6.6937E-01	4.0765E-01
CCNA_01152	4.60715E-05	1.47099E-04	-1.6748E+00	-9.2919E-01
CCNA_01153	1.82715E-05	1.21004E-04	-2.7272E+00	-1.5294E+00
CCNA_01154	5.70447E-05	1.47099E-04	-1.3666E+00	-7.5343E-01
CCNA_01155	2.82051E-04	6.06182E-05	2.2180E+00	1.2908E+00
CCNA_01156	2.46971E-05	1.13979E-04	-2.2063E+00	-1.2323E+00
CCNA_01157	7.71701E-05	3.89436E-05	9.8643E-01	5.8846E-01
CCNA_01158	2.41343E-05	1.38445E-04	-2.5201E+00	-1.4112E+00
CCNA_01159	1.94949E-04	1.47099E-04	4.0626E-01	2.5761E-01
CCNA_01160	2.00923E-05	1.21116E-04	-2.5915E+00	-1.4520E+00
CCNA_01161	8.41856E-05	1.02969E-04	-2.9062E-01	-1.3981E-01
CCNA_01162	4.02461E-04	1.14182E-04	1.8174E+00	1.0624E+00
CCNA_01163	1.28069E-04	1.12088E-04	1.9223E-01	1.3555E-01
CCNA_01164	1.23299E-04	1.09003E-04	1.7773E-01	1.2728E-01
CCNA_01165	3.89899E-05	5.50569E-05	-4.9791E-01	-2.5802E-01

CCNA_01166	1.98636E-05	1.29589E-04	-2.7056E+00	-1.5170E+00
CCNA_01167	2.12932E-05	1.69794E-04	-2.9952E+00	-1.6822E+00
CCNA_01168	8.89529E-05	1.45575E-04	-7.1068E-01	-3.7936E-01
CCNA_01169	1.04549E-04	7.70167E-05	4.4084E-01	2.7732E-01
CCNA_01170	3.07919E-04	7.00820E-05	2.1353E+00	1.2436E+00
CCNA_01171	3.49961E-04	4.33191E-05	3.0139E+00	1.7447E+00
CCNA_01172	6.71781E-05	8.92649E-05	-4.1016E-01	-2.0798E-01
CCNA_01173	3.73644E-04	1.12013E-04	1.7379E+00	1.0170E+00
CCNA_01174	5.08418E-05	3.08082E-05	7.2244E-01	4.3791E-01
CCNA_01175	5.08839E-05	3.26769E-05	6.3869E-01	3.9015E-01
CCNA_01176	1.52589E-05	7.74595E-05	-2.3436E+00	-1.3106E+00
CCNA_01177	1.90811E-05	8.43641E-05	-2.1444E+00	-1.1970E+00
CCNA_01178	1.25379E-04	1.29912E-04	-5.1295E-02	-3.3298E-03
CCNA_01179	7.48467E-05	1.47384E-04	-9.7758E-01	-5.3157E-01
CCNA_01180	5.74329E-05	1.44742E-04	-1.3335E+00	-7.3456E-01
CCNA_01181	6.21659E-04	1.46003E-04	2.0901E+00	1.2178E+00
CCNA_01182	6.22014E-04	1.44569E-04	2.1051E+00	1.2264E+00
CCNA_01183	2.49619E-04	1.46498E-04	7.6879E-01	4.6435E-01
CCNA_01184	2.00529E-04	1.32464E-04	5.9815E-01	3.6704E-01
CCNA_01185	2.26355E-05	3.85609E-05	-7.6864E-01	-4.1242E-01
CCNA_01186	2.42848E-05	2.55171E-05	-7.1659E-02	-1.4943E-02
CCNA_01187	2.05558E-05	2.57873E-05	-3.2732E-01	-1.6074E-01
CCNA_01188	1.57615E-05	1.39414E-04	-3.1447E+00	-1.7674E+00
CCNA_01189	1.76539E-04	6.95191E-05	1.3444E+00	7.9259E-01
CCNA_01190	7.21049E-05	8.63379E-05	-2.5996E-01	-1.2233E-01
CCNA_01191	1.63149E-04	7.70392E-05	1.0824E+00	6.4320E-01
CCNA_01192	6.06653E-05	1.18234E-04	-9.6273E-01	-5.2310E-01
CCNA_01193	1.49275E-04	1.42385E-04	6.8124E-02	6.4772E-02
CCNA_01194	1.29369E-04	8.48819E-05	6.0787E-01	3.7258E-01
CCNA_01195	2.17473E-04	4.81673E-05	2.1745E+00	1.2660E+00
CCNA_01196	2.14301E-04	4.24635E-05	2.3351E+00	1.3576E+00
CCNA_01197	9.90983E-05	1.47346E-04	-5.7231E-01	-3.0045E-01
CCNA_01198	9.86981E-05	1.47346E-04	-5.7815E-01	-3.0378E-01
CCNA_01199	6.90200E-05	5.85543E-05	2.3712E-01	1.6115E-01
CCNA_01200	9.16044E-05	7.92081E-05	2.0968E-01	1.4550E-01
CCNA_01201	2.98255E-05	3.94915E-05	-4.0512E-01	-2.0511E-01
CCNA_01202	3.68651E-05	1.44705E-04	-1.9727E+00	-1.0991E+00
CCNA_01203	3.58960E-05	9.77981E-05	-1.4460E+00	-7.9868E-01
CCNA_01204	5.31598E-04	1.23210E-04	2.1091E+00	1.2287E+00
CCNA_01205	5.65017E-04	1.46078E-04	1.9515E+00	1.1388E+00
CCNA_01206	1.52679E-05	6.16388E-05	-2.0132E+00	-1.1222E+00
CCNA_01207	2.32795E-05	6.60893E-05	-1.5053E+00	-8.3253E-01
CCNA_01208	2.09742E-05	3.16412E-05	-5.9333E-01	-3.1244E-01
CCNA_01209	1.54725E-05	5.86443E-05	-1.9222E+00	-1.0703E+00
CCNA_01210	4.77807E-04	8.81841E-05	2.4377E+00	1.4161E+00
CCNA_01211	5.79656E-05	6.65546E-05	-1.9943E-01	-8.7808E-02
CCNA_01212	3.44847E-04	9.68750E-05	1.8317E+00	1.0705E+00
CCNA_01213	1.71038E-05	1.07802E-04	-2.6558E+00	-1.4886E+00
CCNA_01214	8.15431E-05	9.58318E-05	-2.3300E-01	-1.0695E-01
CCNA_01215	4.22884E-05	3.73450E-05	1.7916E-01	1.2809E-01
CCNA_01216	3.53921E-04	1.38701E-04	1.3514E+00	7.9659E-01
CCNA_01217	2.82421E-04	8.90697E-05	1.6647E+00	9.7528E-01
CCNA_01218	7.48557E-05	7.92832E-05	-8.2981E-02	-2.1400E-02

CCNA_01219	3.69794E-05	1.43496E-04	-1.9562E+00	-1.0896E+00
CCNA_01220	4.98748E-04	8.92348E-05	2.4825E+00	1.4416E+00
CCNA_01221	3.03483E-04	1.09844E-04	1.4661E+00	8.6199E-01
CCNA_01222	1.34128E-04	1.22752E-04	1.2780E-01	9.8803E-02
CCNA_01223	1.36234E-04	4.84825E-05	1.4904E+00	8.7584E-01
CCNA_01224	3.65879E-04	1.47099E-04	1.3145E+00	7.7556E-01
CCNA_01225	6.30670E-05	9.09985E-05	-5.2901E-01	-2.7576E-01
CCNA_01226	5.98316E-05	5.21074E-05	1.9928E-01	1.3957E-01
CCNA_01227	5.28854E-05	3.85459E-05	4.5609E-01	2.8602E-01
CCNA_01228	9.67358E-05	4.60359E-05	1.0711E+00	6.3675E-01
CCNA_01229	7.42869E-05	6.64421E-05	1.6091E-01	1.1768E-01
CCNA_01230	1.13424E-04	1.32239E-05	3.0997E+00	1.7936E+00
CCNA_01231	3.72954E-05	1.04763E-04	-1.4900E+00	-8.2382E-01
CCNA_01232	2.21660E-05	1.47099E-04	-2.7302E+00	-1.5311E+00
CCNA_01233	1.85111E-04	3.53787E-05	2.3872E+00	1.3873E+00
CCNA_01234	1.91500E-04	6.30798E-05	1.6019E+00	9.3948E-01
CCNA_01235	2.59762E-05	1.11592E-04	-2.1029E+00	-1.1733E+00
CCNA_01236	2.80649E-05	1.30850E-04	-2.2210E+00	-1.2407E+00
CCNA_01237	2.60996E-05	1.00868E-04	-1.9503E+00	-1.0863E+00
CCNA_01238	2.25482E-05	4.23434E-05	-9.0919E-01	-4.9257E-01
CCNA_01239	4.48165E-05	1.22752E-04	-1.4536E+00	-8.0306E-01
CCNA_01240	1.53762E-04	1.25942E-04	2.8789E-01	1.9010E-01
CCNA_01241	1.52829E-05	7.30690E-05	-2.2572E+00	-1.2613E+00
CCNA_01242	1.55379E-04	9.72352E-05	6.7615E-01	4.1152E-01
CCNA_01243	8.54617E-05	1.47099E-04	-7.8346E-01	-4.2087E-01
CCNA_01244	1.09641E-04	1.23563E-04	-1.7250E-01	-7.2453E-02
CCNA_01245	6.38585E-05	1.13191E-04	-8.2584E-01	-4.4503E-01
CCNA_01246	1.66313E-05	8.09343E-05	-2.2827E+00	-1.2759E+00
CCNA_01247	1.19690E-04	2.90220E-05	2.0438E+00	1.1914E+00
CCNA_01248	1.12624E-04	3.99943E-05	1.4934E+00	8.7758E-01
CCNA_01249	1.38720E-04	7.72418E-05	8.4462E-01	5.0759E-01
CCNA_01250	1.90570E-05	3.12960E-05	-7.1577E-01	-3.8227E-01
CCNA_01251	1.64808E-05	9.90589E-05	-2.5873E+00	-1.4496E+00
CCNA_01252	2.15319E-04	7.71893E-05	1.4799E+00	8.6986E-01
CCNA_01253	2.14404E-04	7.81349E-05	1.4562E+00	8.5634E-01
CCNA_01254	2.04896E-04	1.45965E-04	4.8921E-01	3.0491E-01
CCNA_01255	1.73897E-04	7.49678E-05	1.2138E+00	7.1811E-01
CCNA_01256	2.00442E-05	1.20583E-04	-2.5886E+00	-1.4503E+00
CCNA_01257	2.91514E-05	1.02144E-04	-1.8089E+00	-1.0057E+00
CCNA_01258	2.43642E-04	1.08635E-04	1.1652E+00	6.9040E-01
CCNA_01259	2.36470E-04	1.24546E-04	9.2491E-01	5.5338E-01
CCNA_01260	1.77830E-04	1.11074E-05	4.0000E+00	2.3070E+00
CCNA_01261	3.11696E-04	1.21109E-04	1.3638E+00	8.0365E-01
CCNA_01262	2.13353E-05	3.52962E-05	-7.2637E-01	-3.8831E-01
CCNA_01263	1.05404E-04	1.45860E-04	-4.6870E-01	-2.4137E-01
CCNA_01264	1.47707E-03	4.10623E-04	1.8468E+00	1.0791E+00
CCNA_01265	1.54491E-03	2.52957E-04	2.6105E+00	1.5146E+00
CCNA_01266	4.92678E-05	1.33447E-04	-1.4375E+00	-7.9388E-01
CCNA_01267	2.35874E-04	8.04014E-05	1.5526E+00	9.1134E-01
CCNA_01268	1.71874E-04	4.27111E-05	2.0084E+00	1.1713E+00
CCNA_01269	1.72621E-04	4.31539E-05	1.9998E+00	1.1664E+00
CCNA_01270	1.92015E-05	6.69074E-05	-1.8009E+00	-1.0011E+00
CCNA_01271	1.84100E-05	1.47391E-04	-3.0009E+00	-1.6854E+00

CCNA_01272	1.87440E-05	1.11750E-04	-2.5756E+00	-1.4429E+00
CCNA_01273	4.28572E-05	3.48834E-05	2.9679E-01	1.9517E-01
CCNA_01274	2.28850E-04	1.33117E-04	7.8165E-01	4.7168E-01
CCNA_01275	2.19487E-04	9.10961E-05	1.2686E+00	7.4936E-01
CCNA_01276	2.04776E-05	1.05761E-04	-2.3686E+00	-1.3248E+00
CCNA_01277	1.28906E-04	5.11393E-05	1.3336E+00	7.8646E-01
CCNA_01278	1.47370E-04	1.46138E-04	1.2066E-02	3.2803E-02
CCNA_01279	2.33115E-04	6.99169E-05	1.7372E+00	1.0166E+00
CCNA_01280	1.55809E-04	8.17974E-05	9.2955E-01	5.5602E-01
CCNA_01281	1.65494E-04	9.44133E-05	8.0963E-01	4.8764E-01
CCNA_01282	1.44974E-05	3.97166E-05	-1.4539E+00	-8.0322E-01
CCNA_01283	2.96690E-05	1.16718E-04	-1.9759E+00	-1.1009E+00
CCNA_01284	1.53233E-04	1.16381E-04	3.9681E-01	2.5221E-01
CCNA_01285	4.00718E-04	7.87878E-05	2.3464E+00	1.3640E+00
CCNA_01286	3.67347E-04	1.39646E-04	1.3953E+00	8.2163E-01
CCNA_01287	7.46541E-05	3.04704E-05	1.2925E+00	7.6301E-01
CCNA_01288	8.38305E-05	6.14437E-05	4.4808E-01	2.8146E-01
CCNA_01289	3.28562E-05	1.47384E-04	-2.1653E+00	-1.2089E+00
CCNA_01290	5.07967E-05	1.24546E-04	-1.2939E+00	-7.1195E-01
CCNA_01291	1.23347E-04	1.47099E-04	-2.5410E-01	-1.1899E-01
CCNA_01292	2.05540E-04	5.13119E-05	2.0019E+00	1.1675E+00
CCNA_01293	0.00000E+00	0.00000E+00	-1.3183E+00	-7.2586E-01
CCNA_01294	5.92598E-06	6.37928E-07	3.1994E+00	1.8505E+00
CCNA_01295	1.60865E-05	6.33125E-05	-1.9765E+00	-1.1013E+00
CCNA_01296	1.45046E-04	1.04282E-04	4.7595E-01	2.9734E-01
CCNA_01297	1.96499E-04	3.80430E-05	2.3686E+00	1.3767E+00
CCNA_01298	2.50489E-04	1.81532E-04	4.6448E-01	2.9081E-01
CCNA_01299	6.13123E-05	1.66492E-04	-1.4412E+00	-7.9596E-01
CCNA_01300	4.67427E-05	4.28838E-05	1.2415E-01	9.6722E-02
CCNA_01301	2.84411E-05	7.76171E-05	-1.4484E+00	-8.0006E-01
CCNA_01302	2.90099E-05	2.93147E-05	-1.5297E-02	1.7199E-02
CCNA_01303	2.34661E-05	4.59833E-05	-9.7058E-01	-5.2758E-01
CCNA_01304	1.66785E-04	1.42108E-04	2.3095E-01	1.5763E-01
CCNA_01305	2.63325E-04	3.46207E-05	2.9268E+00	1.6950E+00
CCNA_01306	3.37146E-04	1.37710E-04	1.2917E+00	7.6254E-01
CCNA_01307	5.65270E-05	8.17073E-05	-5.3158E-01	-2.7723E-01
CCNA_01308	7.67879E-05	8.44842E-05	-1.3787E-01	-5.2704E-02
CCNA_01309	5.55639E-05	1.47324E-04	-1.4068E+00	-7.7632E-01
CCNA_01310	5.17327E-05	3.34124E-05	6.3045E-01	3.8545E-01
CCNA_01311	2.66314E-04	1.17236E-04	1.1836E+00	7.0092E-01
CCNA_01312	3.81727E-04	6.38228E-05	2.5802E+00	1.4974E+00
CCNA_01313	1.78369E-04	1.12808E-04	6.6092E-01	4.0283E-01
CCNA_01314	6.66213E-05	5.16421E-05	3.6729E-01	2.3538E-01
CCNA_01315	6.38013E-05	1.19713E-04	-9.0794E-01	-4.9186E-01
CCNA_01316	1.13415E-04	9.15914E-05	3.0825E-01	2.0171E-01
CCNA_01317	2.25314E-04	1.36749E-04	7.2034E-01	4.3672E-01
CCNA_01318	2.06952E-04	6.60743E-05	1.6470E+00	9.6516E-01
CCNA_01319	7.68270E-05	8.67282E-05	-1.7496E-01	-7.3850E-02
CCNA_01320	3.46710E-05	1.45845E-04	-2.0726E+00	-1.1560E+00
CCNA_01321	4.81813E-05	1.46401E-04	-1.6034E+00	-8.8844E-01
CCNA_01322	1.83835E-04	1.41898E-04	3.7351E-01	2.3892E-01
CCNA_01323	5.34000E-05	3.58816E-05	5.7338E-01	3.5291E-01
CCNA_01324	7.81302E-05	4.45799E-05	8.0930E-01	4.8745E-01

CCNA_01325	1.24009E-04	1.46926E-04	-2.4468E-01	-1.1361E-01
CCNA_01326	1.48739E-04	1.28779E-04	2.0784E-01	1.4445E-01
CCNA_01327	2.30830E-04	1.97307E-05	3.5478E+00	2.0491E+00
CCNA_01328	3.79302E-04	8.43791E-05	2.1683E+00	1.2624E+00
CCNA_01329	6.25463E-05	7.12978E-05	-1.8902E-01	-8.1869E-02
CCNA_01330	1.70947E-04	1.31075E-04	3.8310E-01	2.4439E-01
CCNA_01331	7.56141E-05	7.10652E-05	8.9419E-02	7.6916E-02
CCNA_01332	4.14427E-05	1.43826E-04	-1.7951E+00	-9.9779E-01
CCNA_01333	2.59641E-05	7.71292E-05	-1.5707E+00	-8.6983E-01
CCNA_01334	4.23426E-05	1.34573E-04	-1.6682E+00	-9.2540E-01
CCNA_01335	2.29100E-04	4.38819E-05	2.3840E+00	1.3855E+00
CCNA_01336	2.38288E-04	7.66189E-05	1.6368E+00	9.5936E-01
CCNA_01337	1.16599E-04	7.58609E-05	6.2003E-01	3.7951E-01
CCNA_01338	2.13570E-04	8.58801E-05	1.3142E+00	7.7539E-01
CCNA_01339	9.41836E-05	4.50227E-05	1.0646E+00	6.3306E-01
CCNA_01340	1.84521E-05	1.31653E-04	-2.8347E+00	-1.5907E+00
CCNA_01341	2.87815E-04	1.47399E-04	9.6536E-01	5.7644E-01
CCNA_01342	1.96641E-04	4.66588E-05	2.0751E+00	1.2093E+00
CCNA_01343	6.11889E-05	4.07148E-05	5.8752E-01	3.6097E-01
CCNA_01344	1.50524E-04	5.03288E-05	1.5804E+00	9.2716E-01
CCNA_01345	2.62651E-05	4.77020E-05	-8.6096E-01	-4.6507E-01
CCNA_01346	1.90029E-05	1.46641E-04	-2.9478E+00	-1.6552E+00
CCNA_01347	1.68811E-05	1.14850E-04	-2.7661E+00	-1.5515E+00
CCNA_01348	3.70456E-05	1.18354E-05	1.6454E+00	9.6425E-01
CCNA_01349	3.86615E-04	0.00000E+00	1.5653E+01	8.9523E+00
CCNA_01350	0.00000E+00	0.00000E+00	-1.3183E+00	-7.2586E-01
CCNA_01351	2.30358E-05	1.08448E-04	-2.2350E+00	-1.2486E+00
CCNA_01352	2.08652E-04	7.45100E-05	1.4855E+00	8.7305E-01
CCNA_01353	1.60775E-05	1.38671E-04	-3.1084E+00	-1.7467E+00
CCNA_01354	2.44353E-04	1.28854E-04	9.2316E-01	5.5238E-01
CCNA_01355	2.24146E-04	1.13003E-04	9.8800E-01	5.8935E-01
CCNA_01356	1.52838E-04	5.53496E-05	1.4652E+00	8.6149E-01
CCNA_01357	3.24379E-05	9.49537E-05	-1.5495E+00	-8.5774E-01
CCNA_01358	2.45105E-05	3.24443E-05	-4.0472E-01	-2.0488E-01
CCNA_01359	1.48345E-05	1.23946E-04	-3.0625E+00	-1.7205E+00
CCNA_01360	2.04415E-05	1.27751E-04	-2.6436E+00	-1.4817E+00
CCNA_01361	1.58527E-04	1.47099E-04	1.0790E-01	8.7453E-02
CCNA_01362	4.73025E-05	6.72076E-05	-5.0678E-01	-2.6308E-01
CCNA_01363	2.09381E-05	7.46901E-05	-1.8347E+00	-1.0204E+00
CCNA_01364	2.05287E-05	1.43166E-04	-2.8018E+00	-1.5719E+00
CCNA_01365	2.00246E-04	1.06534E-04	9.1038E-01	5.4509E-01
CCNA_01366	1.56802E-05	2.72433E-05	-7.9708E-01	-4.2863E-01
CCNA_01367	2.30930E-05	3.29621E-05	-5.1350E-01	-2.6691E-01
CCNA_01368	1.29764E-04	1.20531E-04	1.0643E-01	8.6615E-02
CCNA_01369	1.20951E-04	1.43774E-04	-2.4941E-01	-1.1631E-01
CCNA_01370	2.11259E-04	1.46979E-04	5.2335E-01	3.2438E-01
CCNA_01371	6.49059E-05	6.51437E-05	-5.3762E-03	2.2856E-02
CCNA_01372	2.15984E-04	1.08996E-04	9.8657E-01	5.8854E-01
CCNA_01373	2.13305E-04	6.12261E-05	1.8005E+00	1.0527E+00
CCNA_01374	1.03501E-04	7.80448E-05	4.0718E-01	2.5813E-01
CCNA_01375	5.45136E-05	1.35128E-04	-1.3096E+00	-7.2094E-01
CCNA_01376	2.66564E-05	9.97494E-05	-1.9038E+00	-1.0598E+00
CCNA_01377	5.50932E-04	7.72118E-05	2.8348E+00	1.6426E+00

CCNA_01378	5.42219E-04	8.38537E-05	2.6928E+00	1.5616E+00
CCNA_01379	7.88946E-05	1.09716E-04	-4.7582E-01	-2.4543E-01
CCNA_01380	2.51856E-04	8.35610E-05	1.5916E+00	9.3357E-01
CCNA_01381	6.23025E-05	8.11819E-05	-3.8193E-01	-1.9188E-01
CCNA_01382	1.16747E-04	5.21525E-05	1.1624E+00	6.8882E-01
CCNA_01383	1.79627E-04	6.27196E-05	1.5179E+00	8.9153E-01
CCNA_01384	7.06091E-05	1.47099E-04	-1.0589E+00	-5.7793E-01
CCNA_01385	1.05714E-04	9.64097E-05	1.3284E-01	1.0168E-01
CCNA_01386	2.07183E-04	3.68722E-05	2.4900E+00	1.4459E+00
CCNA_01387	1.74610E-04	5.26478E-05	1.7295E+00	1.0122E+00
CCNA_01388	2.82755E-04	1.22805E-04	1.2031E+00	7.1203E-01
CCNA_01389	1.60453E-04	9.98169E-05	6.8471E-01	4.1640E-01
CCNA_01390	5.63675E-05	6.38979E-05	-1.8100E-01	-7.7296E-02
CCNA_01391	1.61506E-04	8.14596E-05	9.8733E-01	5.8897E-01
CCNA_01392	3.55619E-05	1.47099E-04	-2.0483E+00	-1.1422E+00
CCNA_01393	3.84391E-05	2.03236E-05	9.1900E-01	5.5001E-01
CCNA_01394	2.10952E-04	7.18457E-05	1.5538E+00	9.1202E-01
CCNA_01395	1.97568E-04	4.91279E-05	2.0075E+00	1.1708E+00
CCNA_01396	1.84039E-05	7.42248E-05	-2.0118E+00	-1.1214E+00
CCNA_01397	3.93507E-04	1.45373E-04	1.4366E+00	8.4517E-01
CCNA_01398	1.55779E-05	7.16055E-05	-2.2004E+00	-1.2289E+00
CCNA_01399	1.69653E-05	1.41297E-04	-3.0579E+00	-1.7179E+00
CCNA_01400	2.39838E-05	4.09475E-05	-7.7180E-01	-4.1422E-01
CCNA_01401	1.12943E-04	1.45898E-04	-3.6940E-01	-1.8474E-01
CCNA_01402	1.43620E-04	7.76396E-05	8.8728E-01	5.3192E-01
CCNA_01403	1.02701E-04	1.47346E-04	-5.2079E-01	-2.7107E-01
CCNA_01404	6.11679E-05	5.70082E-05	1.0149E-01	8.3797E-02
CCNA_01405	2.76556E-05	1.22069E-04	-2.1420E+00	-1.1956E+00
CCNA_01406	1.90570E-05	1.00500E-04	-2.3987E+00	-1.3420E+00
CCNA_01407	2.15370E-05	5.11618E-05	-1.2483E+00	-6.8593E-01
CCNA_01408	4.42387E-05	6.22543E-05	-4.9294E-01	-2.5519E-01
CCNA_01409	3.75723E-05	1.46333E-04	-1.9615E+00	-1.0927E+00
CCNA_01410	4.96741E-05	1.32074E-04	-1.4108E+00	-7.7861E-01
CCNA_04014	3.44077E-04	9.99821E-05	1.7829E+00	1.0427E+00
CCNA_01412	2.58582E-04	1.46656E-04	8.1813E-01	4.9248E-01
CCNA_01413	9.74009E-05	1.21919E-04	-3.2396E-01	-1.5883E-01
CCNA_01414	1.14056E-04	1.17124E-04	-3.8340E-02	4.0579E-03
CCNA_01415	9.40572E-05	1.18444E-04	-3.3265E-01	-1.6378E-01
CCNA_01416	1.53672E-05	6.96542E-05	-2.1802E+00	-1.2174E+00
CCNA_01417	1.93998E-04	1.47099E-04	3.9921E-01	2.5358E-01
CCNA_01418	2.04785E-04	8.19024E-05	1.3220E+00	7.7984E-01
CCNA_01419	2.60424E-04	1.08883E-04	1.2580E+00	7.4333E-01
CCNA_01420	2.76826E-05	7.47501E-05	-1.4331E+00	-7.9133E-01
CCNA_01421	8.25333E-05	5.29480E-05	6.4025E-01	3.9104E-01
CCNA_01422	2.68014E-04	9.60269E-05	1.4807E+00	8.7033E-01
CCNA_01423	2.74753E-04	1.46889E-04	9.0335E-01	5.4109E-01
CCNA_01424	1.67465E-04	1.37702E-04	2.8226E-01	1.8689E-01
CCNA_01425	3.03967E-04	1.12853E-04	1.4294E+00	8.4107E-01
CCNA_01426	3.47252E-04	5.81640E-05	2.5776E+00	1.4959E+00
CCNA_01427	2.37855E-04	9.52689E-05	1.3199E+00	7.7864E-01
CCNA_01428	7.84101E-05	9.67849E-05	-3.0380E-01	-1.4733E-01
CCNA_01429	1.72570E-04	4.99235E-05	1.7892E+00	1.0463E+00
CCNA_01430	9.14087E-05	6.46484E-05	4.9960E-01	3.1083E-01

CCNA_01431	1.26928E-04	3.97016E-05	1.6765E+00	9.8200E-01
CCNA_01432	2.04866E-05	5.88995E-05	-1.5235E+00	-8.4292E-01
CCNA_01433	1.97697E-04	5.88320E-05	1.7485E+00	1.0230E+00
CCNA_01434	8.37432E-05	1.35443E-04	-6.9367E-01	-3.6966E-01
CCNA_01435	2.18418E-04	6.81007E-05	1.6812E+00	9.8468E-01
CCNA_01436	1.68666E-04	1.31278E-04	3.6149E-01	2.3207E-01
CCNA_01437	1.97914E-04	1.37177E-04	5.2878E-01	3.2747E-01
CCNA_01438	1.94025E-04	1.20193E-04	6.9082E-01	4.1988E-01
CCNA_01439	2.17807E-05	6.72076E-05	-1.6255E+00	-9.0108E-01
CCNA_01440	2.17567E-05	6.06782E-05	-1.4797E+00	-8.1792E-01
CCNA_01441	1.69142E-05	6.63295E-05	-1.9713E+00	-1.0983E+00
CCNA_01442	2.27402E-04	4.33115E-05	2.3922E+00	1.3901E+00
CCNA_01443	3.74790E-05	9.45484E-05	-1.3350E+00	-7.3538E-01
CCNA_01444	3.10032E-04	1.46100E-04	1.0854E+00	6.4490E-01
CCNA_01445	3.44874E-04	5.73310E-05	2.5885E+00	1.5021E+00
CCNA_01446	2.27167E-04	5.20249E-05	2.1263E+00	1.2385E+00
CCNA_01447	3.52973E-04	1.36074E-04	1.3751E+00	8.1011E-01
CCNA_01448	1.44129E-04	1.47099E-04	-2.9471E-02	9.1156E-03
CCNA_01449	1.81060E-05	5.65429E-05	-1.6428E+00	-9.1095E-01
CCNA_01450	1.22011E-04	7.00670E-05	8.0008E-01	4.8219E-01
CCNA_01451	2.77600E-04	7.43299E-05	1.9009E+00	1.1099E+00
CCNA_01452	5.79506E-05	6.74402E-05	-2.1887E-01	-9.8895E-02
CCNA_01453	3.95466E-05	8.71184E-05	-1.1394E+00	-6.2387E-01
CCNA_01454	4.62040E-05	4.84975E-05	-7.0024E-02	-1.4011E-02
CCNA_01455	5.20186E-05	2.80463E-05	8.9091E-01	5.3399E-01
CCNA_01456	1.18402E-04	1.35263E-04	-1.9212E-01	-8.3638E-02
CCNA_01457	5.29456E-05	2.27327E-05	1.2193E+00	7.2129E-01
CCNA_01458	1.17620E-04	9.77756E-05	2.6651E-01	1.7791E-01
CCNA_01459	1.27019E-04	9.57942E-05	4.0695E-01	2.5800E-01
CCNA_01460	6.42708E-05	1.03007E-04	-6.8054E-01	-3.6217E-01
CCNA_01461	1.22745E-04	6.81982E-05	8.4774E-01	5.0937E-01
CCNA_01462	7.53613E-05	9.71977E-05	-3.6715E-01	-1.8346E-01
CCNA_01463	1.88930E-04	1.41680E-04	4.1516E-01	2.6268E-01
CCNA_01464	3.03961E-04	1.42603E-04	1.0918E+00	6.4856E-01
CCNA_01465	2.43239E-04	1.47384E-04	7.2274E-01	4.3808E-01
CCNA_01466	2.98192E-04	8.31483E-05	1.8424E+00	1.0766E+00
CCNA_01467	2.74970E-04	4.40846E-05	2.6407E+00	1.5319E+00
CCNA_01468	4.90180E-05	1.21852E-04	-1.3137E+00	-7.2328E-01
CCNA_01469	1.28888E-04	9.11036E-05	5.0045E-01	3.1132E-01
CCNA_01470	5.25964E-05	1.47099E-04	-1.4837E+00	-8.2022E-01
CCNA_01471	1.87681E-05	4.92705E-05	-1.3924E+00	-7.6815E-01
CCNA_01472	4.64447E-05	7.03972E-05	-6.0006E-01	-3.1628E-01
CCNA_01473	1.40099E-05	2.02411E-05	-5.3107E-01	-2.7693E-01
CCNA_01474	1.01506E-04	3.00276E-05	1.7569E+00	1.0278E+00
CCNA_01475	2.97864E-05	1.36974E-04	-2.2011E+00	-1.2293E+00
CCNA_01476	4.71279E-05	1.10504E-04	-1.2295E+00	-6.7521E-01
CCNA_01477	1.35936E-04	3.93564E-05	1.7880E+00	1.0456E+00
CCNA_01478	1.92806E-04	5.74285E-05	1.7471E+00	1.0223E+00
CCNA_01479	2.69862E-04	1.21484E-04	1.1514E+00	6.8253E-01
CCNA_01480	1.62361E-04	1.07465E-04	5.9527E-01	3.6539E-01
CCNA_01481	1.93384E-04	4.19081E-05	2.2059E+00	1.2839E+00
CCNA_01482	3.71236E-04	1.18257E-04	1.6503E+00	9.6707E-01
CCNA_01483	4.12080E-05	8.96852E-05	-1.1220E+00	-6.1391E-01

CCNA_01484	3.08223E-04	9.86687E-05	1.6432E+00	9.6301E-01
CCNA_01485	2.22262E-05	5.44415E-05	-1.2925E+00	-7.1113E-01
CCNA_01486	4.28100E-04	6.55715E-05	2.7067E+00	1.5695E+00
CCNA_01487	4.18505E-04	7.62662E-05	2.4560E+00	1.4265E+00
CCNA_01488	1.34681E-04	1.28088E-04	7.2357E-02	6.7186E-02
CCNA_01489	1.05003E-04	1.33770E-04	-3.4936E-01	-1.7331E-01
CCNA_01490	4.20206E-05	1.21154E-04	-1.5277E+00	-8.4527E-01
CCNA_01491	8.92929E-05	6.32074E-05	4.9833E-01	3.1011E-01
CCNA_01492	1.75787E-04	1.45755E-04	2.7023E-01	1.8003E-01
CCNA_01493	2.30779E-05	7.81949E-05	-1.7605E+00	-9.7806E-01
CCNA_01494	1.50843E-05	1.39684E-04	-3.2108E+00	-1.8051E+00
CCNA_01495	3.16349E-04	9.67324E-05	1.7093E+00	1.0007E+00
CCNA_01496	2.90027E-04	1.46979E-04	9.8052E-01	5.8509E-01
CCNA_01497	6.25222E-05	4.83699E-05	3.7010E-01	2.3698E-01
CCNA_01498	1.99750E-05	1.16441E-04	-2.5432E+00	-1.4244E+00
CCNA_01499	2.37400E-05	6.77629E-05	-1.5132E+00	-8.3699E-01
CCNA_01500	2.44172E-05	8.49945E-05	-1.7994E+00	-1.0002E+00
CCNA_01501	9.36419E-05	1.01671E-04	-1.1874E-01	-4.1791E-02
CCNA_01503	1.81421E-05	5.49218E-05	-1.5980E+00	-8.8538E-01
CCNA_01502	1.59180E-05	1.45838E-04	-3.1954E+00	-1.7964E+00
CCNA_01504	1.98916E-04	9.92015E-05	1.0036E+00	5.9827E-01
CCNA_01505	3.79064E-05	6.78680E-05	-8.4034E-01	-4.5330E-01
CCNA_01506	2.22232E-05	4.48051E-05	-1.0116E+00	-5.5099E-01
CCNA_01507	1.69479E-04	9.48936E-05	8.3663E-01	5.0304E-01
CCNA_01508	1.35175E-04	1.47099E-04	-1.2200E-01	-4.3651E-02
CCNA_01509	1.09054E-04	9.77681E-05	1.5754E-01	1.1576E-01
CCNA_01510	1.09009E-04	9.50137E-05	1.9817E-01	1.3893E-01
CCNA_01511	6.18541E-05	1.06549E-04	-7.8461E-01	-4.2152E-01
CCNA_01512	2.00622E-05	6.62019E-05	-1.7223E+00	-9.5629E-01
CCNA_01513	4.97915E-05	1.43736E-04	-1.5294E+00	-8.4628E-01
CCNA_01514	1.38221E-04	7.40672E-05	8.9995E-01	5.3914E-01
CCNA_01515	6.83639E-05	4.41371E-05	6.3106E-01	3.8580E-01
CCNA_01516	3.93029E-05	1.46626E-04	-1.8994E+00	-1.0573E+00
CCNA_01517	2.52307E-04	3.62568E-05	2.7986E+00	1.6219E+00
CCNA_04008	5.72554E-05	6.24344E-05	-1.2503E-01	-4.5378E-02
CCNA_01519	3.36387E-05	7.60035E-05	-1.1760E+00	-6.4470E-01
CCNA_01520	1.00796E-04	6.25920E-05	6.8726E-01	4.1785E-01
CCNA_01521	4.96831E-05	1.21356E-04	-1.2884E+00	-7.0884E-01
CCNA_01522	1.55264E-04	1.25724E-04	3.0440E-01	1.9952E-01
CCNA_01523	1.76136E-04	1.47099E-04	2.5986E-01	1.7411E-01
CCNA_01524	5.56100E-04	1.27638E-04	2.1232E+00	1.2367E+00
CCNA_01525	5.57463E-04	1.27998E-04	2.1227E+00	1.2364E+00
CCNA_01526	1.85863E-04	1.04185E-04	8.3501E-01	5.0211E-01
CCNA_01527	1.58114E-04	4.23734E-05	1.8995E+00	1.1092E+00
CCNA_01528	1.85950E-04	2.05938E-05	3.1741E+00	1.8361E+00
CCNA_01529	1.10117E-04	6.52262E-05	7.5538E-01	4.5670E-01
CCNA_01530	3.42319E-04	1.37522E-04	1.3156E+00	7.7619E-01
CCNA_01531	1.31581E-04	6.05731E-05	1.1191E+00	6.6410E-01
CCNA_01532	5.99378E-04	7.71442E-05	2.9577E+00	1.7126E+00
CCNA_01533	6.93722E-05	9.16815E-05	-4.0233E-01	-2.0352E-01
CCNA_01534	1.90462E-04	7.01195E-05	1.4415E+00	8.4797E-01
CCNA_01535	1.66231E-04	6.04005E-05	1.4604E+00	8.5876E-01
CCNA_01536	1.86357E-05	3.09207E-05	-7.3062E-01	-3.9074E-01

CCNA_01537	1.39948E-04	9.75804E-05	5.2015E-01	3.2255E-01
CCNA_01538	6.69795E-05	5.79539E-05	2.0869E-01	1.4494E-01
CCNA_01539	2.28823E-05	1.10752E-04	-2.2749E+00	-1.2714E+00
CCNA_01540	2.29515E-05	9.89539E-05	-2.1081E+00	-1.1763E+00
CCNA_01541	2.43058E-05	1.41290E-04	-2.5392E+00	-1.4221E+00
CCNA_01542	4.79869E-04	7.72718E-05	2.6345E+00	1.5283E+00
CCNA_01543	1.54425E-05	1.05040E-04	-2.7658E+00	-1.5513E+00
CCNA_01544	3.10595E-05	1.11232E-04	-1.8404E+00	-1.0236E+00
CCNA_01545	1.50061E-05	1.24291E-04	-3.0499E+00	-1.7134E+00
CCNA_01546	1.86146E-05	1.69914E-05	1.3123E-01	1.0076E-01
CCNA_01547	1.77936E-04	3.79680E-05	2.2282E+00	1.2966E+00
CCNA_01548	1.93580E-05	6.72301E-05	-1.7961E+00	-9.9836E-01
CCNA_01549	6.24831E-05	7.91181E-05	-3.4061E-01	-1.6832E-01
CCNA_01550	1.19016E-04	7.20183E-05	7.2461E-01	4.3915E-01
CCNA_01551	1.01970E-04	7.38720E-05	4.6493E-01	2.9106E-01
CCNA_01552	8.98016E-05	1.30227E-04	-5.3625E-01	-2.7989E-01
CCNA_01553	3.39156E-05	1.40562E-04	-2.0511E+00	-1.1438E+00
CCNA_01554	2.30478E-05	5.24677E-05	-1.1868E+00	-6.5089E-01
CCNA_01555	2.07027E-04	1.33762E-04	6.3009E-01	3.8525E-01
CCNA_01556	2.39642E-04	1.46168E-04	7.1320E-01	4.3264E-01
CCNA_01557	5.03994E-05	1.45800E-04	-1.5325E+00	-8.4803E-01
CCNA_01558	4.55148E-05	5.87119E-05	-3.6741E-01	-1.8360E-01
CCNA_01559	4.83950E-05	1.12636E-04	-1.2187E+00	-6.6910E-01
CCNA_01560	1.41944E-04	7.21609E-05	9.7591E-01	5.8246E-01
CCNA_01561	4.48535E-04	1.06519E-04	2.0740E+00	1.2087E+00
CCNA_01562	1.32788E-04	7.43374E-05	8.3686E-01	5.0316E-01
CCNA_01563	3.02228E-05	1.31173E-04	-2.1177E+00	-1.1818E+00
CCNA_01564	1.49940E-05	3.10333E-05	-1.0495E+00	-5.7258E-01
CCNA_01565	5.66083E-05	1.12035E-04	-9.8489E-01	-5.3574E-01
CCNA_01566	1.55716E-04	7.22510E-05	1.1077E+00	6.5762E-01
CCNA_01567	1.45140E-04	9.14864E-05	6.6573E-01	4.0557E-01
CCNA_01568	1.89168E-04	5.08766E-05	1.8944E+00	1.1063E+00
CCNA_01569	2.58227E-05	1.44457E-04	-2.4838E+00	-1.3906E+00
CCNA_01570	1.98636E-05	1.30895E-04	-2.7201E+00	-1.5253E+00
CCNA_01571	2.89798E-05	4.82649E-05	-7.3600E-01	-3.9380E-01
CCNA_01572	9.03373E-05	4.47675E-05	1.0127E+00	6.0343E-01
CCNA_01573	1.47821E-04	1.18850E-04	3.1465E-01	2.0536E-01
CCNA_01574	1.56426E-04	1.42115E-04	1.3837E-01	1.0483E-01
CCNA_01575	1.19642E-04	3.13260E-05	1.9330E+00	1.1283E+00
CCNA_01576	2.08809E-05	1.02098E-04	-2.2896E+00	-1.2798E+00
CCNA_01577	2.19327E-04	1.18812E-04	8.8433E-01	5.3024E-01
CCNA_01578	1.98061E-04	1.08966E-04	8.6200E-01	5.1750E-01
CCNA_01579	1.67667E-05	7.83300E-05	-2.2238E+00	-1.2423E+00
CCNA_01580	2.60033E-05	1.28306E-04	-2.3027E+00	-1.2873E+00
CCNA_01581	6.05058E-05	1.28764E-04	-1.0896E+00	-5.9545E-01
CCNA_01582	2.29665E-05	2.34833E-05	-3.2370E-02	7.4625E-03
CCNA_01583	3.08849E-05	8.45967E-05	-1.4537E+00	-8.0308E-01
CCNA_01584	4.38564E-05	6.89112E-05	-6.5201E-01	-3.4590E-01
CCNA_01585	2.63554E-05	1.34505E-04	-2.3514E+00	-1.3150E+00
CCNA_01586	2.42968E-05	1.20688E-04	-2.3124E+00	-1.2928E+00
CCNA_01587	2.43600E-05	1.12688E-04	-2.2097E+00	-1.2342E+00
CCNA_01588	3.32535E-05	1.45785E-04	-2.1322E+00	-1.1900E+00
CCNA_01589	4.26481E-04	9.44809E-05	2.1743E+00	1.2659E+00

CCNA_01590	4.01432E-04	5.33233E-05	2.9121E+00	1.6866E+00
CCNA_01591	1.80518E-05	1.39353E-04	-2.9484E+00	-1.6555E+00
CCNA_01592	1.65620E-05	7.48852E-05	-2.1767E+00	-1.2154E+00
CCNA_01593	1.35942E-04	1.47601E-04	-1.1875E-01	-4.1800E-02
CCNA_01594	1.60928E-04	5.71058E-05	1.4945E+00	8.7823E-01
CCNA_01595	5.43240E-05	4.09325E-05	4.0816E-01	2.5869E-01
CCNA_01596	2.77191E-04	7.57783E-05	1.8709E+00	1.0929E+00
CCNA_01597	4.62548E-04	7.49753E-05	2.6250E+00	1.5229E+00
CCNA_01598	4.43970E-04	4.11726E-05	3.4305E+00	1.9822E+00
CCNA_01599	1.08181E-04	4.21107E-05	1.3610E+00	8.0206E-01
CCNA_01600	1.87010E-04	1.37027E-04	4.4860E-01	2.8175E-01
CCNA_01601	1.69087E-04	1.02999E-04	7.1506E-01	4.3370E-01
CCNA_01602	1.19335E-04	5.98526E-05	9.9539E-01	5.9357E-01
CCNA_01603	1.15110E-04	4.41671E-05	1.3818E+00	8.1391E-01
CCNA_01604	9.75695E-05	6.07983E-05	6.8227E-01	4.1500E-01
CCNA_01605	3.12431E-05	2.02636E-06	3.9414E+00	2.2736E+00
CCNA_01606	2.45869E-04	3.51311E-05	2.8068E+00	1.6266E+00
CCNA_01607	2.42631E-04	2.84366E-05	3.0926E+00	1.7896E+00
CCNA_01608	4.68390E-05	4.88803E-05	-6.1671E-02	-9.2473E-03
CCNA_01609	1.01599E-04	2.49467E-05	2.0256E+00	1.1811E+00
CCNA_01610	1.59944E-04	1.47099E-04	1.2074E-01	9.4777E-02
CCNA_01611	1.33432E-04	1.10474E-04	2.7233E-01	1.8123E-01
CCNA_01612	2.09134E-04	6.63445E-05	1.6562E+00	9.7044E-01
CCNA_01613	1.50828E-04	1.12335E-04	4.2502E-01	2.6830E-01
CCNA_01614	5.93200E-05	9.07659E-05	-6.1368E-01	-3.2404E-01
CCNA_01615	1.66322E-04	1.46964E-04	1.7847E-01	1.2770E-01
CCNA_01616	1.54665E-05	7.82625E-05	-2.3390E+00	-1.3080E+00
CCNA_01617	2.42487E-05	1.47099E-04	-2.6007E+00	-1.4572E+00
CCNA_01618	5.17477E-05	3.37952E-05	6.1444E-01	3.7632E-01
CCNA_01619	1.37453E-04	6.52938E-05	1.0738E+00	6.3828E-01
CCNA_01620	1.27813E-04	1.38851E-04	-1.1954E-01	-4.2248E-02
CCNA_01621	1.47743E-04	1.39856E-04	7.9098E-02	7.1030E-02
CCNA_01622	1.72889E-04	7.21759E-05	1.2601E+00	7.4455E-01
CCNA_01623	1.34028E-04	9.10361E-05	5.5794E-01	3.4410E-01
CCNA_01624	1.26664E-04	1.25874E-04	8.9653E-03	3.1035E-02
CCNA_01625	1.09199E-04	1.43279E-04	-3.9190E-01	-1.9757E-01
CCNA_01626	1.07727E-04	1.38535E-04	-3.6291E-01	-1.8104E-01
CCNA_01627	1.58728E-05	1.43714E-04	-3.1784E+00	-1.7866E+00
CCNA_01628	1.51565E-05	6.23368E-05	-2.0400E+00	-1.1375E+00
CCNA_01629	4.59632E-05	8.09718E-05	-8.1698E-01	-4.3998E-01
CCNA_01630	1.91741E-04	6.56540E-05	1.5461E+00	9.0761E-01
CCNA_01631	3.17980E-04	8.19400E-05	1.9562E+00	1.1415E+00
CCNA_01632	3.39638E-05	1.22144E-04	-1.8465E+00	-1.0271E+00
CCNA_01633	3.94172E-05	1.45875E-04	-1.8878E+00	-1.0506E+00
CCNA_01634	6.22845E-05	1.03787E-04	-7.3672E-01	-3.9421E-01
CCNA_01635	2.73787E-05	1.26933E-04	-2.2129E+00	-1.2360E+00
CCNA_01636	1.65500E-05	5.11468E-05	-1.6278E+00	-9.0235E-01
CCNA_01637	4.06984E-04	1.36809E-04	1.5727E+00	9.2282E-01
CCNA_01638	7.66615E-05	7.91856E-05	-4.6816E-02	-7.7578E-04
CCNA_01639	3.06201E-05	1.33515E-04	-2.1244E+00	-1.1856E+00
CCNA_01640	1.89336E-05	1.00718E-04	-2.4112E+00	-1.3491E+00
CCNA_01641	8.89137E-05	9.69725E-05	-1.2523E-01	-4.5495E-02
CCNA_01642	2.02187E-05	7.14629E-05	-1.8214E+00	-1.0128E+00

CCNA_01643	2.06220E-05	1.20516E-04	-2.5468E+00	-1.4265E+00
CCNA_01644	3.99620E-05	8.08217E-05	-1.0161E+00	-5.5356E-01
CCNA_01645	6.85325E-05	5.93723E-05	2.0688E-01	1.4390E-01
CCNA_01646	1.25020E-05	8.79965E-05	-2.8151E+00	-1.5794E+00
CCNA_01647	1.68522E-04	1.39121E-04	2.7654E-01	1.8363E-01
CCNA_01648	1.43864E-04	7.33692E-05	9.7134E-01	5.7986E-01
CCNA_01649	2.81642E-05	4.87302E-05	-7.9102E-01	-4.2518E-01
CCNA_01650	2.69769E-04	1.46296E-04	8.8278E-01	5.2935E-01
CCNA_01651	2.48283E-04	1.27601E-04	9.6028E-01	5.7355E-01
CCNA_01652	1.98389E-04	7.17556E-05	1.4670E+00	8.6254E-01
CCNA_01653	2.05889E-05	1.24734E-05	7.2236E-01	4.3787E-01
CCNA_01654	1.44670E-04	9.28673E-05	6.3944E-01	3.9058E-01
CCNA_01655	6.09030E-05	1.07127E-04	-8.1476E-01	-4.3872E-01
CCNA_01656	1.71730E-05	9.17415E-05	-2.4173E+00	-1.3526E+00
CCNA_01657	1.61049E-04	1.41838E-04	1.8321E-01	1.3040E-01
CCNA_01658	1.29785E-04	8.92874E-05	5.3950E-01	3.3359E-01
CCNA_01659	4.86538E-05	5.49744E-05	-1.7631E-01	-7.4626E-02
CCNA_01660	2.64192E-04	2.43613E-05	3.4385E+00	1.9868E+00
CCNA_01661	6.87100E-05	1.21236E-04	-8.1926E-01	-4.4128E-01
CCNA_01662	1.87103E-04	8.32158E-05	1.1688E+00	6.9246E-01
CCNA_01663	5.50764E-05	5.60176E-05	-2.4560E-02	1.1916E-02
CCNA_01664	1.07540E-04	1.36839E-04	-3.4764E-01	-1.7233E-01
CCNA_01665	2.69664E-04	1.47099E-04	8.7432E-01	5.2453E-01
CCNA_01666	1.47135E-04	1.15578E-04	3.4822E-01	2.2450E-01
CCNA_01667	3.04151E-04	4.82724E-05	2.6553E+00	1.5402E+00
CCNA_01668	1.81596E-04	1.28208E-04	5.0218E-01	3.1230E-01
CCNA_01669	2.51214E-05	4.82123E-05	-9.4053E-01	-5.1044E-01
CCNA_01670	1.26411E-04	1.45297E-04	-2.0093E-01	-8.8663E-02
CCNA_01671	1.39668E-04	1.38018E-04	1.7105E-02	3.5677E-02
CCNA_01672	2.22581E-04	8.57525E-05	1.3760E+00	8.1061E-01
CCNA_01673	4.17046E-05	7.14404E-05	-7.7658E-01	-4.1695E-01
CCNA_01674	3.03769E-04	6.50987E-05	2.2221E+00	1.2931E+00
CCNA_01675	2.95342E-04	7.71217E-05	1.9371E+00	1.1306E+00
CCNA_01676	7.73296E-05	7.54781E-05	3.4876E-02	4.5811E-02
CCNA_01677	3.08157E-04	7.17781E-05	2.1019E+00	1.2246E+00
CCNA_01678	1.34621E-05	4.98559E-05	-1.8888E+00	-1.0512E+00
CCNA_01679	1.57308E-04	1.25282E-04	3.2836E-01	2.1318E-01
CCNA_01680	1.69397E-04	3.79980E-05	2.1562E+00	1.2555E+00
CCNA_01681	3.60193E-05	4.82874E-05	-4.2298E-01	-2.1529E-01
CCNA_01682	1.97709E-04	1.47076E-04	4.2676E-01	2.6930E-01
CCNA_01683	2.14193E-04	1.20583E-04	8.2881E-01	4.9857E-01
CCNA_01684	2.59331E-04	7.43974E-05	1.8013E+00	1.0532E+00
CCNA_01685	4.77533E-04	5.47417E-05	3.1247E+00	1.8079E+00
CCNA_01686	4.67264E-04	1.02068E-04	2.1946E+00	1.2775E+00
CCNA_01687	1.46148E-04	8.13095E-05	8.4583E-01	5.0828E-01
CCNA_01688	1.39352E-04	1.11833E-04	3.1733E-01	2.0689E-01
CCNA_01689	3.70378E-04	1.37545E-04	1.4290E+00	8.4087E-01
CCNA_01690	2.48115E-05	5.88395E-05	-1.2458E+00	-6.8452E-01
CCNA_01691	2.15701E-05	1.38250E-04	-2.6801E+00	-1.5025E+00
CCNA_01692	1.66608E-04	7.38871E-05	1.1729E+00	6.9482E-01
CCNA_01693	2.16092E-05	7.30390E-05	-1.7570E+00	-9.7604E-01
CCNA_01694	1.81331E-05	6.15413E-05	-1.7629E+00	-9.7940E-01
CCNA_01695	3.80870E-05	9.12762E-05	-1.2609E+00	-6.9317E-01

CCNA_01696	1.65199E-05	7.11477E-06	1.2141E+00	7.1827E-01
CCNA_01697	2.48536E-05	4.72667E-05	-9.2742E-01	-5.0297E-01
CCNA_01698	1.99629E-05	9.61620E-05	-2.2680E+00	-1.2675E+00
CCNA_01699	6.95016E-05	1.15855E-04	-7.3724E-01	-3.9451E-01
CCNA_01700	6.65100E-05	1.07855E-04	-6.9748E-01	-3.7184E-01
CCNA_01701	9.78794E-05	9.06083E-05	1.1129E-01	8.9388E-02
CCNA_01702	1.02180E-04	1.06249E-04	-5.6389E-02	-6.2352E-03
CCNA_01703	6.18481E-05	1.05003E-04	-7.6366E-01	-4.0958E-01
CCNA_01704	4.72032E-05	1.74192E-05	1.4377E+00	8.4580E-01
CCNA_01705	2.53261E-05	1.42220E-04	-2.4893E+00	-1.3937E+00
CCNA_01706	2.05829E-05	1.47099E-04	-2.8371E+00	-1.5920E+00
CCNA_01707	3.84451E-05	8.82967E-05	-1.1996E+00	-6.5816E-01
CCNA_01708	7.59091E-05	9.97119E-05	-3.9354E-01	-1.9851E-01
CCNA_01709	2.87553E-04	7.77597E-05	1.8866E+00	1.1018E+00
CCNA_01710	4.06692E-05	1.25094E-04	-1.6210E+00	-8.9849E-01
CCNA_01711	1.60856E-04	4.06848E-05	1.9830E+00	1.1568E+00
CCNA_01712	3.02749E-04	1.28861E-04	1.2322E+00	7.2864E-01
CCNA_01713	3.11245E-04	1.47099E-04	1.0812E+00	6.4251E-01
CCNA_01714	5.02844E-04	1.47009E-04	1.7741E+00	1.0377E+00
CCNA_01715	7.72845E-05	1.47099E-04	-9.2855E-01	-5.0361E-01
CCNA_01716	2.63855E-05	1.45905E-04	-2.4671E+00	-1.3810E+00
CCNA_01717	5.45828E-05	7.45400E-05	-4.4963E-01	-2.3049E-01
CCNA_01718	8.86609E-05	1.39646E-04	-6.5543E-01	-3.4786E-01
CCNA_01719	1.10336E-04	5.68206E-05	9.5727E-01	5.7183E-01
CCNA_04016	1.08371E-04	7.02621E-05	6.2505E-01	3.8237E-01
CCNA_01721	9.12944E-05	9.40005E-05	-4.2211E-02	1.8504E-03
CCNA_01722	1.55839E-05	6.99769E-05	-2.1667E+00	-1.2097E+00
CCNA_01723	8.52901E-05	4.37844E-05	9.6177E-01	5.7440E-01
CCNA_01724	9.32807E-05	1.47917E-04	-6.6516E-01	-3.5340E-01
CCNA_01725	2.07313E-04	1.23503E-04	7.4720E-01	4.5203E-01
CCNA_01726	7.86870E-05	3.73375E-05	1.0753E+00	6.3912E-01
CCNA_01727	3.90479E-04	1.43128E-04	1.4479E+00	8.5161E-01
CCNA_01728	1.44896E-04	4.56831E-05	1.6651E+00	9.7548E-01
CCNA_01729	1.35235E-04	1.47099E-04	-1.2136E-01	-4.3284E-02
CCNA_01730	3.34320E-04	5.14920E-05	2.6986E+00	1.5649E+00
CCNA_01731	9.21250E-05	5.38636E-05	7.7413E-01	4.6739E-01
CCNA_01732	1.57729E-04	7.23860E-05	1.1235E+00	6.6665E-01
CCNA_01733	1.47234E-04	7.38120E-05	9.9607E-01	5.9396E-01
CCNA_01734	1.91097E-04	1.35601E-04	4.9488E-01	3.0814E-01
CCNA_01735	2.31679E-04	6.16238E-05	1.9104E+00	1.1154E+00
CCNA_01736	1.88533E-04	3.12585E-05	2.5922E+00	1.5042E+00
CCNA_01737	1.64808E-05	1.13874E-04	-2.7884E+00	-1.5642E+00
CCNA_01738	2.24218E-05	1.22077E-04	-2.4447E+00	-1.3682E+00
CCNA_01739	7.62913E-05	1.21296E-04	-6.6898E-01	-3.5558E-01
CCNA_01740	1.69593E-05	2.05713E-05	-2.7883E-01	-1.3309E-01
CCNA_01741	2.39537E-05	1.29147E-04	-2.4306E+00	-1.3602E+00
CCNA_01742	2.50071E-05	9.34977E-05	-1.9025E+00	-1.0591E+00
CCNA_01743	3.59411E-05	1.47346E-04	-2.0355E+00	-1.1349E+00
CCNA_01744	2.23631E-04	8.82892E-05	1.3407E+00	7.9050E-01
CCNA_01745	2.85383E-04	4.38894E-05	2.7007E+00	1.5661E+00
CCNA_01746	2.85169E-04	2.19372E-05	3.6999E+00	2.1359E+00
CCNA_01747	1.01217E-04	1.24336E-04	-2.9683E-01	-1.4336E-01
CCNA_01748	7.04857E-05	1.47076E-04	-1.0612E+00	-5.7924E-01

CCNA_01749	1.43894E-04	7.53956E-05	9.3234E-01	5.5761E-01
CCNA_01750	2.28841E-04	7.20558E-05	1.6670E+00	9.7659E-01
CCNA_01751	1.35927E-04	1.07997E-04	3.3177E-01	2.1512E-01
CCNA_01752	3.52549E-05	1.07149E-04	-1.6037E+00	-8.8863E-01
CCNA_01753	1.73918E-04	1.14024E-04	6.0900E-01	3.7322E-01
CCNA_01754	1.59830E-04	1.15052E-04	4.7418E-01	2.9634E-01
CCNA_01755	1.73205E-05	8.96026E-05	-2.3709E+00	-1.3262E+00
CCNA_01756	1.25382E-05	1.41155E-04	-3.4926E+00	-1.9658E+00
CCNA_01757	3.28502E-05	6.18565E-05	-9.1307E-01	-4.9478E-01
CCNA_01758	5.15310E-05	4.83474E-05	9.1862E-02	7.8309E-02
CCNA_01759	4.75222E-05	4.82724E-05	-2.2730E-02	1.2960E-02
CCNA_01760	2.24456E-04	2.12092E-05	3.4032E+00	1.9667E+00
CCNA_01761	3.49660E-05	1.26212E-04	-1.8518E+00	-1.0301E+00
CCNA_01762	2.22626E-04	8.75012E-05	1.3471E+00	7.9417E-01
CCNA_01763	2.08291E-04	9.27097E-05	1.1677E+00	6.9184E-01
CCNA_01764	1.37781E-05	1.38025E-04	-3.3242E+00	-1.8698E+00
CCNA_01765	1.68600E-05	6.49711E-05	-1.9461E+00	-1.0839E+00
CCNA_01766	1.47054E-04	1.41095E-04	5.9635E-02	5.9931E-02
CCNA_01767	6.10475E-05	5.26853E-05	2.1240E-01	1.4705E-01
CCNA_01768	6.77048E-05	7.38720E-05	-1.2585E-01	-4.5848E-02
CCNA_01769	4.67337E-05	5.65204E-05	-2.7441E-01	-1.3057E-01
CCNA_01770	9.01507E-05	3.22191E-05	1.4841E+00	8.7229E-01
CCNA_01771	1.41456E-04	9.23644E-05	6.1486E-01	3.7656E-01
CCNA_01772	1.64293E-04	4.56006E-05	1.8489E+00	1.0803E+00
CCNA_01773	1.73767E-04	1.08928E-04	6.7371E-01	4.1012E-01
CCNA_01774	7.33238E-05	1.28591E-04	-8.1046E-01	-4.3627E-01
CCNA_01775	2.11427E-04	1.21672E-04	7.9710E-01	4.8049E-01
CCNA_01776	1.96743E-04	8.19400E-05	1.2636E+00	7.4650E-01
CCNA_01777	2.43841E-05	1.38543E-04	-2.5062E+00	-1.4033E+00
CCNA_01778	1.20217E-04	4.72592E-05	1.3468E+00	7.9396E-01
CCNA_01779	2.01673E-04	1.80946E-05	3.4778E+00	2.0092E+00
CCNA_01780	1.72603E-05	2.62526E-05	-6.0517E-01	-3.1919E-01
CCNA_01781	1.41002E-05	1.34303E-04	-3.2515E+00	-1.8283E+00
CCNA_01782	2.32871E-04	4.09024E-05	2.5090E+00	1.4568E+00
CCNA_01783	2.03993E-05	3.46132E-05	-7.6290E-01	-4.0914E-01
CCNA_01784	2.66600E-04	1.45673E-04	8.7189E-01	5.2314E-01
CCNA_01785	2.67764E-04	4.06998E-05	2.7176E+00	1.5757E+00
CCNA_01786	1.45597E-04	1.62934E-05	3.1590E+00	1.8274E+00
CCNA_01787	1.19573E-05	7.83000E-05	-2.7109E+00	-1.5200E+00
CCNA_01788	1.64657E-05	2.78437E-05	-7.5801E-01	-4.0635E-01
CCNA_01789	6.13003E-05	3.49660E-05	8.0970E-01	4.8768E-01
CCNA_01790	8.77671E-05	5.94774E-05	5.6121E-01	3.4597E-01
CCNA_01791	1.74700E-04	1.11750E-04	6.4454E-01	3.9349E-01
CCNA_01792	2.72180E-04	1.26115E-04	1.1098E+00	6.5879E-01
CCNA_01793	2.33316E-04	2.48717E-05	3.2293E+00	1.8675E+00
CCNA_01794	4.82535E-05	1.25206E-04	-1.3756E+00	-7.5855E-01
CCNA_01795	5.31382E-05	1.02932E-04	-9.5389E-01	-5.1806E-01
CCNA_01796	1.36773E-04	8.74411E-05	6.4531E-01	3.9393E-01
CCNA_01797	1.96689E-04	4.40846E-05	2.1573E+00	1.2562E+00
CCNA_01798	3.90440E-05	5.69632E-05	-5.4501E-01	-2.8488E-01
CCNA_01799	3.76205E-05	1.41275E-04	-1.9089E+00	-1.0627E+00
CCNA_01800	1.93098E-05	1.23143E-04	-2.6728E+00	-1.4983E+00
CCNA_01801	4.98727E-05	1.37410E-04	-1.4622E+00	-8.0791E-01

CCNA_01802	2.14440E-04	1.21567E-04	8.1876E-01	4.9284E-01
CCNA_01803	1.45149E-04	1.41763E-04	3.4010E-02	4.5317E-02
CCNA_01804	1.92587E-05	1.47099E-04	-2.9331E+00	-1.6467E+00
CCNA_01805	1.27837E-04	1.47099E-04	-2.0251E-01	-8.9567E-02
CCNA_01806	1.56122E-04	7.99211E-05	9.6592E-01	5.7676E-01
CCNA_01807	4.09600E-04	5.93048E-05	2.7878E+00	1.6158E+00
CCNA_01808	1.06060E-04	4.82724E-05	1.1354E+00	6.7343E-01
CCNA_01809	1.39415E-04	9.27922E-05	5.8723E-01	3.6081E-01
CCNA_01810	9.84483E-05	8.54223E-05	2.0467E-01	1.4264E-01
CCNA_01811	1.24307E-04	3.25343E-05	1.9336E+00	1.1286E+00
CCNA_01812	8.45588E-05	1.09371E-04	-3.7125E-01	-1.8579E-01
CCNA_01813	1.29556E-04	7.28364E-05	8.3073E-01	4.9967E-01
CCNA_01814	1.53191E-05	1.12403E-04	-2.8751E+00	-1.6137E+00
CCNA_01815	1.22781E-04	1.29890E-04	-8.1245E-02	-2.0410E-02
CCNA_01816	2.11538E-04	8.74712E-05	1.2739E+00	7.5242E-01
CCNA_01817	6.53513E-05	5.66555E-05	2.0587E-01	1.4333E-01
CCNA_01818	1.72422E-05	5.40362E-05	-1.6479E+00	-9.1386E-01
CCNA_01819	1.84190E-05	1.19773E-04	-2.7009E+00	-1.5143E+00
CCNA_01820	3.61578E-05	4.75819E-05	-3.9621E-01	-2.0003E-01
CCNA_01821	1.45961E-04	6.16388E-05	1.2435E+00	7.3508E-01
CCNA_01822	1.45095E-05	9.27622E-06	6.4452E-01	3.9348E-01
CCNA_01823	1.30140E-04	5.95975E-05	1.1266E+00	6.6839E-01
CCNA_01824	8.87602E-05	4.65988E-05	9.2944E-01	5.5596E-01
CCNA_01825	3.68319E-05	8.87095E-06	2.0527E+00	1.1965E+00
CCNA_01826	1.93851E-05	8.89872E-05	-2.1985E+00	-1.2279E+00
CCNA_01827	4.31672E-05	1.32246E-04	-1.6152E+00	-8.9519E-01
CCNA_01828	1.24024E-04	1.08230E-04	1.9645E-01	1.3796E-01
CCNA_01829	1.17511E-04	1.25372E-04	-9.3460E-02	-2.7376E-02
CCNA_01830	1.50298E-04	1.46611E-04	3.5792E-02	4.6334E-02
CCNA_01831	2.37981E-04	8.41389E-05	1.4999E+00	8.8128E-01
CCNA_01832	3.35304E-05	4.05722E-05	-2.7516E-01	-1.3100E-01
CCNA_01833	2.75773E-05	4.34316E-05	-6.5536E-01	-3.4781E-01
CCNA_01834	1.45456E-05	8.63079E-05	-2.5687E+00	-1.4390E+00
CCNA_01835	6.51737E-05	1.07052E-04	-7.1598E-01	-3.8239E-01
CCNA_01836	4.39678E-05	1.42543E-04	-1.6969E+00	-9.4176E-01
CCNA_01837	2.40461E-04	1.00342E-04	1.2608E+00	7.4492E-01
CCNA_01838	1.60354E-05	1.42280E-04	-3.1492E+00	-1.7700E+00
CCNA_01839	3.10354E-05	6.72676E-05	-1.1160E+00	-6.1052E-01
CCNA_01840	2.91002E-05	7.90730E-05	-1.4421E+00	-7.9650E-01
CCNA_01841	2.92146E-05	9.47811E-05	-1.6979E+00	-9.4234E-01
CCNA_01842	7.41274E-05	1.32802E-04	-8.4122E-01	-4.5380E-01
CCNA_01843	1.73439E-04	5.25878E-05	1.7215E+00	1.0076E+00
CCNA_01844	1.15967E-04	3.97617E-05	1.5440E+00	9.0645E-01
CCNA_01845	1.09846E-04	6.77029E-05	6.9807E-01	4.2402E-01
CCNA_01846	1.98155E-05	2.77911E-05	-4.8817E-01	-2.5247E-01
CCNA_01847	2.28916E-04	7.62737E-05	1.5854E+00	9.3006E-01
CCNA_01848	1.28644E-04	8.03639E-05	6.7866E-01	4.1295E-01
CCNA_01849	9.01086E-05	8.53097E-05	7.8875E-02	7.0903E-02
CCNA_01850	1.48059E-04	8.90247E-05	7.3380E-01	4.4439E-01
CCNA_01851	2.54405E-05	3.94239E-05	-6.3205E-01	-3.3452E-01
CCNA_01852	2.57625E-05	4.14803E-05	-6.8725E-01	-3.6600E-01
CCNA_01853	3.49013E-04	8.02213E-05	2.1211E+00	1.2355E+00
CCNA_01854	1.82571E-04	9.36628E-06	4.2837E+00	2.4688E+00

CCNA_01855	1.95304E-04	7.78197E-05	1.3274E+00	7.8291E-01
CCNA_01856	1.32201E-04	6.73652E-05	9.7253E-01	5.8054E-01
CCNA_01857	1.67065E-05	6.92790E-05	-2.0519E+00	-1.1442E+00
CCNA_01858	1.25887E-04	1.47099E-04	-2.2469E-01	-1.0221E-01
CCNA_01859	2.80769E-04	5.63028E-05	2.3179E+00	1.3478E+00
CCNA_01860	1.35882E-04	7.94933E-05	7.7335E-01	4.6694E-01
CCNA_01861	1.98817E-05	5.79914E-05	-1.5444E+00	-8.5480E-01
CCNA_01862	3.36658E-05	1.11352E-04	-1.7257E+00	-9.5823E-01
CCNA_01863	2.98932E-04	1.91679E-05	3.9625E+00	2.2857E+00
CCNA_01864	2.60048E-04	5.01486E-05	2.3743E+00	1.3799E+00
CCNA_01865	3.18940E-04	1.00372E-04	1.6678E+00	9.7705E-01
CCNA_01866	1.21138E-05	2.73183E-05	-1.1733E+00	-6.4316E-01
CCNA_01867	2.08568E-05	7.32641E-05	-1.8125E+00	-1.0077E+00
CCNA_01868	5.00804E-05	3.09508E-05	6.9401E-01	4.2170E-01
CCNA_01869	1.21009E-04	7.79848E-05	6.3374E-01	3.8733E-01
CCNA_01870	1.64116E-05	4.44898E-05	-1.4387E+00	-7.9456E-01
CCNA_01871	1.01783E-04	1.29559E-04	-3.4816E-01	-1.7262E-01
CCNA_01872	1.65891E-05	4.69815E-05	-1.5018E+00	-8.3053E-01
CCNA_01873	2.23436E-05	9.00004E-05	-2.0100E+00	-1.1203E+00
CCNA_01874	1.69593E-04	9.98169E-07	7.3978E+00	4.2447E+00
CCNA_01875	1.81896E-04	1.39549E-04	3.8230E-01	2.4394E-01
CCNA_01876	1.84545E-04	6.44532E-05	1.5175E+00	8.9132E-01
CCNA_01877	1.83916E-04	6.40555E-05	1.5215E+00	8.9360E-01
CCNA_01878	2.42459E-04	5.01336E-05	2.2737E+00	1.3226E+00
CCNA_01879	2.53234E-04	5.97776E-05	2.0826E+00	1.2136E+00
CCNA_01880	2.31923E-05	8.45817E-05	-1.8666E+00	-1.0386E+00
CCNA_01881	1.32385E-04	4.92180E-05	1.4273E+00	8.3988E-01
CCNA_01882	1.06529E-04	7.86903E-05	4.3689E-01	2.7507E-01
CCNA_01883	2.30854E-04	2.42788E-05	3.2488E+00	1.8786E+00
CCNA_01884	1.10776E-04	6.23218E-05	8.2970E-01	4.9908E-01
CCNA_01885	7.88977E-05	2.30555E-05	1.7745E+00	1.0379E+00
CCNA_01886	2.52810E-05	1.29117E-04	-2.3525E+00	-1.3156E+00
CCNA_01887	8.86188E-05	9.58093E-05	-1.1262E-01	-3.8300E-02
CCNA_01888	2.60514E-05	1.41402E-04	-2.4403E+00	-1.3657E+00
CCNA_01889	8.79657E-05	9.93366E-05	-1.7544E-01	-7.4130E-02
CCNA_01890	7.13796E-05	8.10168E-05	-1.8278E-01	-7.8315E-02
CCNA_01891	1.43969E-04	1.35616E-04	8.6182E-02	7.5070E-02
CCNA_01892	1.58572E-04	1.03690E-04	6.1279E-01	3.7538E-01
CCNA_01893	3.64708E-05	1.32719E-04	-1.8635E+00	-1.0368E+00
CCNA_01894	1.77478E-05	4.40020E-05	-1.3099E+00	-7.2110E-01
CCNA_01895	1.82986E-05	2.46540E-05	-4.3029E-01	-2.1946E-01
CCNA_01896	6.47388E-04	9.37003E-05	2.7884E+00	1.6161E+00
CCNA_01897	2.78632E-05	6.24794E-05	-1.1650E+00	-6.3847E-01
CCNA_01898	1.09867E-04	3.06355E-05	1.8422E+00	1.0765E+00
CCNA_01899	1.30694E-04	1.35931E-04	-5.6735E-02	-6.4323E-03
CCNA_01900	2.59190E-05	4.22984E-05	-7.0668E-01	-3.7708E-01
CCNA_01901	2.51227E-04	1.23113E-04	1.0289E+00	6.1270E-01
CCNA_01902	1.75754E-04	6.77029E-05	1.3761E+00	8.1070E-01
CCNA_01903	3.28815E-04	4.57957E-05	2.8438E+00	1.6477E+00
CCNA_01904	9.29045E-05	1.74567E-05	2.4114E+00	1.4011E+00
CCNA_01905	7.46059E-05	6.36952E-05	2.2799E-01	1.5594E-01
CCNA_01906	2.03090E-04	1.13701E-05	4.1579E+00	2.3971E+00
CCNA_01907	1.96343E-04	1.31984E-04	5.7296E-01	3.5267E-01

CCNA_01908	8.10104E-05	3.68197E-05	1.1374E+00	6.7455E-01
CCNA_01909	5.38364E-05	9.52239E-05	-8.2277E-01	-4.4329E-01
CCNA_01910	1.14017E-04	5.93723E-05	9.4124E-01	5.6269E-01
CCNA_01911	7.45153E-04	4.65687E-05	3.9999E+00	2.3070E+00
CCNA_01912	3.13869E-04	1.15307E-04	1.4446E+00	8.4974E-01
CCNA_01913	3.82684E-04	1.44637E-04	1.4037E+00	8.2640E-01
CCNA_01914	2.36248E-04	7.27088E-05	1.7000E+00	9.9538E-01
CCNA_01915	2.64999E-05	1.37245E-04	-2.3726E+00	-1.3271E+00
CCNA_04000	9.90923E-05	1.45575E-04	-5.5495E-01	-2.9055E-01
CCNA_01916	1.33477E-04	1.23931E-04	1.0701E-01	8.6947E-02
CCNA_01917	5.96263E-04	1.47099E-04	2.0191E+00	1.1774E+00
CCNA_01918	5.84026E-04	1.47099E-04	1.9892E+00	1.1603E+00
CCNA_01919	1.37119E-05	1.41312E-04	-3.3651E+00	-1.8931E+00
CCNA_01920	1.76717E-04	3.71124E-05	2.2512E+00	1.3097E+00
CCNA_01921	3.00458E-04	1.40945E-04	1.0920E+00	6.4865E-01
CCNA_01922	1.31654E-04	1.40577E-04	-9.4655E-02	-2.8057E-02
CCNA_01923	1.27247E-04	1.21837E-04	6.2634E-02	6.1641E-02
CCNA_01924	3.86588E-05	8.83342E-05	-1.1922E+00	-6.5395E-01
CCNA_01925	2.34896E-04	1.17972E-04	9.9351E-01	5.9250E-01
CCNA_01926	2.44220E-04	1.21476E-04	1.0074E+00	6.0044E-01
CCNA_01927	2.57258E-04	1.24268E-04	1.0497E+00	6.2453E-01
CCNA_01928	2.23014E-05	1.43444E-04	-2.6852E+00	-1.5054E+00
CCNA_01929	5.45376E-05	7.22510E-05	-4.0583E-01	-2.0552E-01
CCNA_01930	3.26937E-05	1.44187E-04	-2.1408E+00	-1.1949E+00
CCNA_01931	3.82134E-05	1.01873E-04	-1.4146E+00	-7.8080E-01
CCNA_01932	4.97373E-05	4.36718E-05	1.8747E-01	1.3283E-01
CCNA_01933	8.94615E-05	6.85435E-05	3.8414E-01	2.4499E-01
CCNA_01934	1.31350E-04	1.08740E-04	2.7246E-01	1.8130E-01
CCNA_01935	4.17377E-05	4.86326E-05	-2.2069E-01	-9.9934E-02
CCNA_01936	9.78193E-05	5.63028E-05	7.9676E-01	4.8030E-01
CCNA_01937	2.05498E-05	1.11607E-04	-2.4411E+00	-1.3662E+00
CCNA_01938	3.57665E-05	8.22101E-05	-1.2007E+00	-6.5882E-01
CCNA_01939	2.99245E-04	1.47099E-04	1.0245E+00	6.1016E-01
CCNA_01940	1.82926E-05	4.70791E-05	-1.3638E+00	-7.5183E-01
CCNA_01941	4.31432E-05	1.44817E-04	-1.7470E+00	-9.7035E-01
CCNA_01942	1.39009E-04	3.05605E-05	2.1851E+00	1.2720E+00
CCNA_01943	1.42214E-04	2.30630E-05	2.6240E+00	1.5223E+00
CCNA_01944	3.63633E-04	7.41572E-05	2.2937E+00	1.3340E+00
CCNA_01945	3.72364E-04	1.37365E-04	1.4386E+00	8.4635E-01
CCNA_01946	3.63995E-04	1.24313E-04	1.5499E+00	9.0977E-01
CCNA_01947	1.18402E-04	9.64997E-05	2.9502E-01	1.9417E-01
CCNA_01948	1.69244E-04	6.78905E-05	1.3177E+00	7.7737E-01
CCNA_01949	3.70005E-05	1.47384E-04	-1.9939E+00	-1.1112E+00
CCNA_01950	6.92548E-05	7.00595E-05	-1.6759E-02	1.6365E-02
CCNA_01951	1.81030E-04	9.33926E-05	9.5475E-01	5.7040E-01
CCNA_01952	1.72753E-04	1.47099E-04	2.3188E-01	1.5816E-01
CCNA_01953	7.74621E-05	5.08016E-05	6.0846E-01	3.7291E-01
CCNA_01954	6.30459E-05	1.88601E-05	1.7406E+00	1.0185E+00
CCNA_01955	8.77309E-05	8.43115E-05	5.7277E-02	5.8586E-02
CCNA_01956	3.65250E-05	7.28364E-05	-9.9581E-01	-5.4196E-01
CCNA_01957	1.49209E-04	3.17163E-05	2.2337E+00	1.2998E+00
CCNA_01958	1.98007E-04	1.46100E-04	4.3854E-01	2.7601E-01
CCNA_01959	1.75221E-04	1.16748E-04	5.8571E-01	3.5994E-01

CCNA_01960	8.05229E-05	3.44556E-05	1.2244E+00	7.2417E-01
CCNA_01961	2.67135E-05	7.34668E-05	-1.4595E+00	-8.0640E-01
CCNA_01962	1.84346E-04	7.23560E-05	1.3491E+00	7.9529E-01
CCNA_01963	1.05407E-04	1.25109E-05	3.0739E+00	1.7789E+00
CCNA_01964	1.66881E-04	1.13514E-04	5.5589E-01	3.4293E-01
CCNA_01965	1.57729E-04	5.86593E-05	1.4269E+00	8.3963E-01
CCNA_01966	1.64149E-04	8.32008E-05	9.8023E-01	5.8493E-01
CCNA_01967	9.39790E-05	1.38926E-04	-5.6394E-01	-2.9568E-01
CCNA_01968	3.88996E-04	1.30145E-04	1.5796E+00	9.2671E-01
CCNA_01969	1.57214E-04	7.40897E-05	1.0853E+00	6.4483E-01
CCNA_01970	9.57306E-05	4.24485E-05	1.1731E+00	6.9489E-01
CCNA_01971	7.72243E-05	2.63952E-05	1.5484E+00	9.0895E-01
CCNA_01972	1.55333E-04	3.68947E-05	2.0736E+00	1.2085E+00
CCNA_01973	1.77087E-04	6.65997E-05	1.4107E+00	8.3043E-01
CCNA_01974	2.08580E-04	9.50212E-05	1.1342E+00	6.7272E-01
CCNA_01975	1.25412E-04	1.37154E-04	-1.2918E-01	-4.7743E-02
CCNA_01976	3.29194E-05	4.71841E-05	-5.1946E-01	-2.7031E-01
CCNA_01977	2.79975E-04	1.47331E-04	9.2617E-01	5.5410E-01
CCNA_01978	1.65804E-04	1.45162E-04	1.9176E-01	1.3528E-01
CCNA_01979	1.34594E-04	1.77344E-05	2.9234E+00	1.6931E+00
CCNA_01980	9.46802E-05	8.07467E-05	2.2957E-01	1.5684E-01
CCNA_01981	2.24399E-05	5.07716E-05	-1.1780E+00	-6.4585E-01
CCNA_01982	2.30057E-05	6.39354E-05	-1.4746E+00	-8.1501E-01
CCNA_01983	2.28907E-04	4.60284E-05	2.3140E+00	1.3455E+00
CCNA_01984	1.22011E-05	3.91012E-05	-1.6801E+00	-9.3222E-01
CCNA_01985	1.98585E-04	9.34752E-05	1.0870E+00	6.4582E-01
CCNA_01986	1.43379E-05	5.93573E-05	-2.0495E+00	-1.1428E+00
CCNA_01987	9.91315E-05	1.46663E-04	-5.6512E-01	-2.9635E-01
CCNA_01988	3.53572E-05	8.30507E-05	-1.2320E+00	-6.7666E-01
CCNA_01989	1.68483E-04	1.44712E-04	2.1936E-01	1.5102E-01
CCNA_01990	1.38798E-04	6.41305E-05	1.1138E+00	6.6108E-01
CCNA_01991	2.35504E-05	8.79515E-05	-1.9009E+00	-1.0581E+00
CCNA_01992	1.54536E-04	8.08817E-05	9.3395E-01	5.5853E-01
CCNA_01993	1.72323E-04	4.50677E-05	1.9347E+00	1.1293E+00
CCNA_01994	2.82752E-04	9.14864E-05	1.6278E+00	9.5422E-01
CCNA_01995	4.72573E-05	3.08157E-05	6.1661E-01	3.7756E-01
CCNA_01996	1.08768E-04	6.41530E-05	7.6154E-01	4.6021E-01
CCNA_01997	7.81302E-05	6.98569E-05	1.6138E-01	1.1795E-01
CCNA_01998	1.58506E-04	1.14752E-04	4.6595E-01	2.9164E-01
CCNA_01999	2.39712E-04	4.40921E-05	2.4425E+00	1.4188E+00
CCNA_02000	4.37601E-05	1.46663E-04	-1.7448E+00	-9.6909E-01
CCNA_02001	1.10779E-04	1.15960E-04	-6.6004E-02	-1.1718E-02
CCNA_02002	2.86186E-05	1.55654E-05	8.7807E-01	5.2666E-01
CCNA_02003	3.73234E-04	1.15923E-04	1.6868E+00	9.8789E-01
CCNA_02004	2.92543E-04	6.02579E-05	2.2793E+00	1.3257E+00
CCNA_02005	3.56531E-04	7.38570E-05	2.2711E+00	1.3211E+00
CCNA_02006	1.89186E-05	8.38462E-05	-2.1478E+00	-1.1989E+00
CCNA_02007	1.33345E-04	6.77029E-05	9.7775E-01	5.8351E-01
CCNA_02008	2.46670E-05	8.48369E-05	-1.7821E+00	-9.9035E-01
CCNA_02009	1.01178E-04	1.43376E-04	-5.0294E-01	-2.6089E-01
CCNA_02010	1.07035E-04	6.81907E-05	6.5031E-01	3.9678E-01
CCNA_02011	1.30046E-04	1.29987E-04	6.0781E-04	2.6269E-02
CCNA_02012	3.11889E-04	1.45808E-04	1.0969E+00	6.5146E-01

CCNA_02013	4.31070E-05	7.51554E-05	-8.0200E-01	-4.3144E-01
CCNA_02014	1.15841E-04	4.32365E-05	1.4216E+00	8.3664E-01
CCNA_02015	4.97764E-05	2.74759E-05	8.5699E-01	5.1464E-01
CCNA_02016	4.71038E-05	1.39489E-04	-1.5662E+00	-8.6726E-01
CCNA_02017	3.81321E-05	4.63886E-05	-2.8288E-01	-1.3540E-01
CCNA_02018	4.89999E-05	1.33747E-04	-1.4486E+00	-8.0021E-01
CCNA_02019	3.08864E-04	1.37950E-04	1.1628E+00	6.8902E-01
CCNA_02020	2.56241E-05	1.17949E-04	-2.2025E+00	-1.2301E+00
CCNA_02021	6.24169E-05	4.01895E-05	6.3492E-01	3.8800E-01
CCNA_02022	4.58789E-05	1.38393E-04	-1.5928E+00	-8.8244E-01
CCNA_02023	5.03723E-05	7.42548E-05	-5.5991E-01	-2.9338E-01
CCNA_02024	1.63661E-04	8.73211E-05	9.0621E-01	5.4271E-01
CCNA_02025	4.87140E-05	1.27150E-04	-1.3841E+00	-7.6341E-01
CCNA_02026	2.00713E-05	3.96566E-05	-9.8249E-01	-5.3437E-01
CCNA_02027	2.12330E-05	8.68032E-05	-2.0314E+00	-1.1325E+00
CCNA_02028	6.78583E-05	9.74904E-05	-5.2278E-01	-2.7221E-01
CCNA_02029	6.02830E-05	8.99178E-05	-5.7690E-01	-3.0307E-01
CCNA_02030	2.36636E-04	1.47099E-04	6.8583E-01	4.1704E-01
CCNA_02031	1.75146E-04	1.46491E-04	2.5770E-01	1.7288E-01
CCNA_02032	1.00534E-04	1.47099E-04	-5.4913E-01	-2.8724E-01
CCNA_02033	2.21961E-04	4.11801E-05	2.4300E+00	1.4117E+00
CCNA_02034	1.48234E-04	1.30603E-04	1.8264E-01	1.3008E-01
CCNA_02035	2.90000E-04	8.90547E-05	1.7032E+00	9.9721E-01
CCNA_02036	8.21721E-05	1.35623E-04	-7.2291E-01	-3.8634E-01
CCNA_02037	1.12440E-04	1.18880E-04	-8.0399E-02	-1.9927E-02
CCNA_02038	1.31976E-04	1.25101E-04	7.7120E-02	6.9902E-02
CCNA_02039	9.76387E-05	9.04657E-05	1.1001E-01	8.8657E-02
CCNA_02040	1.61037E-04	1.44712E-04	1.5416E-01	1.1383E-01
CCNA_02041	1.86200E-04	8.35610E-05	1.1558E+00	6.8508E-01
CCNA_02042	1.02111E-04	5.94399E-05	7.8050E-01	4.7102E-01
CCNA_02043	1.14339E-04	1.46333E-04	-3.5597E-01	-1.7708E-01
CCNA_02044	1.19808E-04	1.13221E-04	8.1520E-02	7.2412E-02
CCNA_02045	3.04115E-04	9.17415E-05	1.7289E+00	1.0119E+00
CCNA_02046	2.97644E-04	7.38495E-05	2.0108E+00	1.1726E+00
CCNA_02047	9.00995E-05	1.42843E-04	-6.6487E-01	-3.5324E-01
CCNA_02048	9.64228E-05	3.40804E-05	1.5002E+00	8.8143E-01
CCNA_02049	1.51150E-04	4.35067E-05	1.7965E+00	1.0504E+00
CCNA_02050	1.52140E-04	6.22543E-05	1.2890E+00	7.6102E-01
CCNA_02051	1.16262E-05	2.76110E-05	-1.2479E+00	-6.8572E-01
CCNA_02052	1.80380E-04	3.73225E-05	2.2727E+00	1.3220E+00
CCNA_02053	1.58758E-05	7.81949E-05	-2.3001E+00	-1.2858E+00
CCNA_02054	1.76214E-05	1.24201E-04	-2.8171E+00	-1.5806E+00
CCNA_02055	1.48405E-05	4.80472E-05	-1.6948E+00	-9.4061E-01
CCNA_02056	1.91353E-05	6.15188E-05	-1.6847E+00	-9.3485E-01
CCNA_02057	1.99870E-05	4.65387E-05	-1.2194E+00	-6.6947E-01
CCNA_02058	1.82565E-05	6.14212E-05	-1.7503E+00	-9.7222E-01
CCNA_02059	1.86628E-05	8.48219E-05	-2.1842E+00	-1.2197E+00
CCNA_02060	4.62190E-05	1.11180E-04	-1.2663E+00	-6.9624E-01
CCNA_02061	3.47222E-05	9.90514E-05	-1.5123E+00	-8.3651E-01
CCNA_02062	2.60550E-04	5.71658E-05	2.1882E+00	1.2738E+00
CCNA_02063	1.68233E-04	1.01543E-04	7.2829E-01	4.4125E-01
CCNA_02064	1.15447E-04	4.86101E-05	1.2477E+00	7.3747E-01
CCNA_02065	1.02857E-04	7.46901E-05	4.6155E-01	2.8914E-01

CCNA_02066	1.03161E-04	8.01763E-05	3.6356E-01	2.3325E-01
CCNA_02067	1.21021E-04	9.19217E-05	3.9669E-01	2.5215E-01
CCNA_02068	1.81090E-05	5.22801E-05	-1.5295E+00	-8.4633E-01
CCNA_02069	1.22583E-04	1.41350E-04	-2.0556E-01	-9.1302E-02
CCNA_02070	2.31893E-05	4.48651E-05	-9.5219E-01	-5.1709E-01
CCNA_02071	7.72393E-05	7.37295E-05	6.7004E-02	6.4134E-02
CCNA_02072	5.72343E-05	1.14850E-04	-1.0048E+00	-5.4710E-01
CCNA_02073	4.06512E-05	9.60644E-06	2.0802E+00	1.2122E+00
CCNA_02074	2.16182E-05	7.95158E-05	-1.8789E+00	-1.0456E+00
CCNA_02075	1.58617E-04	1.07465E-04	5.6161E-01	3.4620E-01
CCNA_02076	7.25744E-05	4.78221E-05	6.0162E-01	3.6901E-01
CCNA_02077	1.57645E-04	5.79464E-05	1.4437E+00	8.4925E-01
CCNA_02078	1.68037E-04	8.95426E-05	9.0804E-01	5.4376E-01
CCNA_02079	6.28172E-05	1.41117E-04	-1.1677E+00	-6.3997E-01
CCNA_02080	1.41661E-04	1.27886E-04	1.4753E-01	1.1006E-01
CCNA_02081	2.96507E-04	7.73394E-05	1.9387E+00	1.1315E+00
CCNA_02082	3.24385E-04	1.03247E-04	1.6515E+00	9.6775E-01
CCNA_02083	1.83136E-05	5.06965E-05	-1.4689E+00	-8.1178E-01
CCNA_02084	1.05945E-04	9.71301E-05	1.2526E-01	9.7354E-02
CCNA_02085	4.08348E-05	5.31582E-05	-3.8059E-01	-1.9112E-01
CCNA_02086	1.16304E-04	5.48543E-05	1.0841E+00	6.4414E-01
CCNA_02087	1.04266E-04	5.43815E-05	9.3892E-01	5.6137E-01
CCNA_02088	1.07751E-04	8.35010E-05	3.6775E-01	2.3564E-01
CCNA_02089	1.42473E-04	1.01551E-04	4.8842E-01	3.0446E-01
CCNA_02090	1.73226E-04	3.07481E-05	2.4938E+00	1.4481E+00
CCNA_02091	2.31968E-04	5.42839E-05	2.0951E+00	1.2207E+00
CCNA_02092	1.67556E-04	1.00117E-04	7.4287E-01	4.4956E-01
CCNA_02093	2.08460E-04	1.36637E-04	6.0936E-01	3.7343E-01
CCNA_02094	2.58937E-04	8.13546E-05	1.6702E+00	9.7839E-01
CCNA_02095	2.68541E-04	1.15367E-04	1.2188E+00	7.2099E-01
CCNA_02096	1.83970E-04	3.91162E-05	2.2334E+00	1.2996E+00
CCNA_02097	1.35747E-04	9.03981E-05	5.8646E-01	3.6037E-01
CCNA_02098	1.58987E-04	1.20478E-04	4.0007E-01	2.5408E-01
CCNA_02099	5.44173E-05	2.64778E-05	1.0390E+00	6.1841E-01
CCNA_02100	2.85470E-04	7.04122E-05	2.0193E+00	1.1775E+00
CCNA_02101	2.37403E-04	9.51863E-05	1.3184E+00	7.7779E-01
CCNA_02102	5.21892E-04	1.47099E-04	1.8269E+00	1.0678E+00
CCNA_02103	1.22080E-04	9.63571E-05	3.4129E-01	2.2055E-01
CCNA_02104	4.54245E-05	7.66114E-05	-7.5413E-01	-4.0414E-01
CCNA_02105	2.64577E-05	1.45538E-04	-2.4595E+00	-1.3767E+00
CCNA_02106	1.30504E-04	1.26400E-04	4.6047E-02	5.2182E-02
CCNA_02107	4.51813E-04	4.06398E-05	3.4745E+00	2.0074E+00
CCNA_02108	9.01928E-05	5.14470E-05	8.0976E-01	4.8771E-01
CCNA_02109	1.19591E-04	3.97992E-05	1.5871E+00	9.3099E-01
CCNA_02110	1.60188E-04	5.18223E-05	1.6279E+00	9.5430E-01
CCNA_02111	4.25150E-04	3.29696E-05	3.6884E+00	2.1294E+00
CCNA_02112	4.06506E-04	3.11384E-05	3.7062E+00	2.1395E+00
CCNA_02113	1.11980E-04	1.20906E-04	-1.1070E-01	-3.7208E-02
CCNA_02114	1.54455E-05	9.79857E-05	-2.6652E+00	-1.4940E+00
CCNA_02115	1.67095E-05	9.42332E-05	-2.4954E+00	-1.3972E+00
CCNA_02116	1.08642E-04	4.27036E-05	1.3469E+00	7.9405E-01
CCNA_02117	1.56080E-05	9.07659E-05	-2.5397E+00	-1.4224E+00
CCNA_02118	2.38424E-05	6.67948E-05	-1.4862E+00	-8.2162E-01

CCNA_02119	1.49769E-04	7.20633E-05	1.0553E+00	6.2772E-01
CCNA_02120	1.35413E-04	1.29762E-04	6.1443E-02	6.0962E-02
CCNA_02121	1.65407E-04	1.47016E-04	1.7000E-01	1.2287E-01
CCNA_02122	1.38835E-05	1.00215E-04	-2.8515E+00	-1.6002E+00
CCNA_02123	4.66494E-06	0.00000E+00	9.2807E+00	5.3185E+00
CCNA_02124	1.08687E-04	1.11397E-04	-3.5590E-02	5.6261E-03
CCNA_02125	7.84110E-04	1.44652E-04	2.4384E+00	1.4165E+00
CCNA_02126	7.78289E-04	1.42558E-04	2.4487E+00	1.4224E+00
CCNA_02127	1.82059E-04	1.44374E-04	3.3454E-01	2.1670E-01
CCNA_02128	1.84644E-04	1.46828E-04	3.3057E-01	2.1444E-01
CCNA_02129	9.35034E-05	1.13349E-04	-2.7772E-01	-1.3246E-01
CCNA_02130	1.49829E-04	1.28681E-04	2.1946E-01	1.5107E-01
CCNA_02131	6.89929E-05	1.20111E-04	-7.9987E-01	-4.3023E-01
CCNA_02132	4.27059E-04	1.48284E-04	1.5260E+00	8.9617E-01
CCNA_02133	2.60484E-05	3.63469E-05	-4.8077E-01	-2.4825E-01
CCNA_02134	1.63342E-04	3.26919E-05	2.3206E+00	1.3493E+00
CCNA_02135	2.59130E-05	3.56564E-05	-4.6062E-01	-2.3676E-01
CCNA_02136	1.55764E-04	1.27668E-04	2.8690E-01	1.8954E-01
CCNA_02137	9.47735E-06	5.78488E-05	-2.6095E+00	-1.4622E+00
CCNA_02138	3.50563E-05	6.77629E-05	-9.5086E-01	-5.1633E-01
CCNA_02139	2.22472E-05	3.21215E-05	-5.3006E-01	-2.7636E-01
CCNA_02140	2.41572E-04	7.17256E-05	1.7518E+00	1.0249E+00
CCNA_02141	5.40344E-04	6.33200E-05	3.0930E+00	1.7898E+00
CCNA_02142	5.44832E-04	6.84459E-05	2.9926E+00	1.7325E+00
CCNA_02143	1.85565E-04	1.24936E-04	5.7067E-01	3.5136E-01
CCNA_02144	4.09311E-05	9.70401E-05	-1.2454E+00	-6.8430E-01
CCNA_02145	1.15961E-04	7.30765E-05	6.6605E-01	4.0576E-01
CCNA_02146	5.32525E-05	3.48534E-05	6.1132E-01	3.7455E-01
CCNA_02147	1.48905E-04	5.15896E-05	1.5291E+00	8.9791E-01
CCNA_02148	2.12029E-05	1.40787E-04	-2.7311E+00	-1.5315E+00
CCNA_02149	1.54963E-04	8.83342E-05	8.1079E-01	4.8830E-01
CCNA_02150	1.10959E-04	1.24666E-04	-1.6809E-01	-6.9933E-02
CCNA_02151	1.45952E-04	8.07091E-05	8.5459E-01	5.1328E-01
CCNA_02152	1.87317E-04	3.84858E-05	2.2828E+00	1.3278E+00
CCNA_02153	2.02446E-04	8.59777E-05	1.2354E+00	7.3044E-01
CCNA_02154	2.60243E-04	1.18054E-05	4.4614E+00	2.5702E+00
CCNA_02155	7.65863E-05	2.81139E-05	1.4455E+00	8.5024E-01
CCNA_02156	1.00329E-04	3.69698E-05	1.4401E+00	8.4716E-01
CCNA_02157	1.38498E-04	1.41117E-04	-2.7079E-02	1.0480E-02
CCNA_02158	1.40710E-04	1.20523E-04	2.2335E-01	1.5329E-01
CCNA_02159	8.55580E-05	9.22744E-05	-1.0909E-01	-3.6292E-02
CCNA_02160	5.88186E-04	1.08335E-04	2.4407E+00	1.4178E+00
CCNA_02161	5.20111E-04	1.37875E-04	1.9154E+00	1.1182E+00
CCNA_02162	3.16521E-04	1.02871E-04	1.6214E+00	9.5055E-01
CCNA_02163	3.54587E-04	7.56507E-05	2.2286E+00	1.2968E+00
CCNA_02164	1.76278E-04	1.25537E-04	4.8968E-01	3.0517E-01
CCNA_02165	1.94856E-04	4.78821E-05	2.0246E+00	1.1805E+00
CCNA_02166	7.58489E-05	4.59233E-05	7.2372E-01	4.3865E-01
CCNA_02167	1.66409E-04	6.07758E-05	1.4530E+00	8.5454E-01
CCNA_04007	5.17929E-05	1.45508E-04	-1.4903E+00	-8.2394E-01
CCNA_02169	6.19715E-05	1.40164E-04	-1.1774E+00	-6.4555E-01
CCNA_02170	9.95979E-05	1.44630E-04	-5.3821E-01	-2.8100E-01
CCNA_02171	2.06500E-04	1.36051E-04	6.0194E-01	3.6919E-01

CCNA_02172	2.00601E-04	1.46648E-04	4.5192E-01	2.8364E-01
CCNA_02173	7.02058E-05	8.17298E-05	-2.1934E-01	-9.9164E-02
CCNA_02174	5.43240E-05	3.24818E-05	7.4170E-01	4.4890E-01
CCNA_02175	2.06491E-05	1.15705E-04	-2.4862E+00	-1.3919E+00
CCNA_02176	7.38505E-05	1.29957E-04	-8.1538E-01	-4.3907E-01
CCNA_02177	1.60925E-05	8.17974E-05	-2.3455E+00	-1.3117E+00
CCNA_02178	4.01094E-05	2.96899E-05	4.3371E-01	2.7326E-01
CCNA_02179	1.36487E-05	7.03447E-05	-2.3655E+00	-1.3231E+00
CCNA_02180	1.32653E-04	9.06833E-05	5.4866E-01	3.3881E-01
CCNA_02181	8.58830E-05	5.42239E-05	6.6330E-01	4.0419E-01
CCNA_02182	1.46371E-04	9.90214E-05	5.6373E-01	3.4741E-01
CCNA_02183	1.70990E-04	4.74018E-05	1.8507E+00	1.0813E+00
CCNA_02184	1.05382E-04	5.77512E-05	8.6757E-01	5.2068E-01
CCNA_02185	1.78168E-04	1.47099E-04	2.7640E-01	1.8355E-01
CCNA_02186	9.16194E-05	1.41695E-04	-6.2909E-01	-3.3284E-01
CCNA_02187	5.91803E-04	6.60218E-05	3.1639E+00	1.8303E+00
CCNA_02188	9.49330E-05	1.18632E-04	-3.2156E-01	-1.5745E-01
CCNA_02189	1.89126E-04	4.86401E-05	1.9589E+00	1.1431E+00
CCNA_02190	4.43440E-05	1.31165E-04	-1.5646E+00	-8.6631E-01
CCNA_02191	2.39146E-05	8.56700E-05	-1.8408E+00	-1.0239E+00
CCNA_02192	2.92543E-04	1.35308E-04	1.1123E+00	6.6026E-01
CCNA_02193	8.32857E-05	1.42198E-04	-7.7178E-01	-4.1421E-01
CCNA_02194	1.15441E-04	7.72718E-05	5.7903E-01	3.5613E-01
CCNA_02195	5.36348E-05	1.47099E-04	-1.4555E+00	-8.0414E-01
CCNA_02196	2.92242E-04	5.86293E-05	2.3173E+00	1.3474E+00
CCNA_02197	2.79565E-05	4.68014E-05	-7.4344E-01	-3.9805E-01
CCNA_02198	6.06382E-05	1.31878E-04	-1.1209E+00	-6.1331E-01
CCNA_02199	4.05669E-05	1.42603E-04	-1.8136E+00	-1.0083E+00
CCNA_02200	2.31992E-04	1.33462E-04	7.9758E-01	4.8077E-01
CCNA_02201	2.71776E-04	1.16456E-04	1.2226E+00	7.2312E-01
CCNA_02202	3.25179E-04	1.44059E-04	1.1745E+00	6.9572E-01
CCNA_02203	1.20208E-04	7.12153E-05	7.5516E-01	4.5657E-01
CCNA_02204	8.31112E-05	1.38363E-04	-7.3537E-01	-3.9344E-01
CCNA_02205	4.24636E-04	1.47099E-04	1.5294E+00	8.9809E-01
CCNA_02206	1.89120E-04	1.18992E-04	6.6836E-01	4.0707E-01
CCNA_02207	4.73991E-04	1.39549E-04	1.7640E+00	1.0319E+00
CCNA_02208	2.49640E-04	4.99685E-05	2.3206E+00	1.3493E+00
CCNA_02209	4.02930E-05	1.20486E-04	-1.5802E+00	-8.7525E-01
CCNA_02210	2.21238E-05	6.23218E-05	-1.4941E+00	-8.2614E-01
CCNA_02211	1.07038E-04	5.25728E-05	1.0256E+00	6.1078E-01
CCNA_02212	2.49421E-04	7.70317E-05	1.6949E+00	9.9251E-01
CCNA_02213	2.92868E-05	6.50161E-05	-1.1506E+00	-6.3022E-01
CCNA_02214	2.40530E-05	8.39363E-05	-1.8030E+00	-1.0023E+00
CCNA_02215	1.92677E-05	1.40322E-04	-2.8643E+00	-1.6075E+00
CCNA_02216	2.09651E-05	5.20324E-05	-1.3114E+00	-7.2195E-01
CCNA_02217	4.58669E-05	9.50888E-05	-1.0518E+00	-5.7392E-01
CCNA_02218	2.12751E-05	3.10934E-05	-5.4758E-01	-2.8635E-01
CCNA_02219	2.88293E-05	5.36085E-05	-8.9498E-01	-4.8446E-01
CCNA_02220	2.50191E-05	7.06449E-05	-1.4975E+00	-8.2809E-01
CCNA_02221	6.16615E-05	9.07809E-05	-5.5807E-01	-2.9233E-01
CCNA_02222	3.75844E-05	1.47339E-04	-1.9709E+00	-1.0980E+00
CCNA_02223	7.76186E-05	1.85449E-05	2.0648E+00	1.2035E+00
CCNA_02224	7.76607E-05	8.98953E-05	-2.1113E-01	-9.4478E-02

CCNA_02225	2.00963E-04	1.03614E-04	9.5562E-01	5.7089E-01
CCNA_02226	7.97253E-05	9.26271E-05	-2.1646E-01	-9.7519E-02
CCNA_02227	2.16393E-04	1.39406E-04	6.3430E-01	3.8765E-01
CCNA_02228	2.04701E-04	1.37425E-04	5.7482E-01	3.5373E-01
CCNA_02229	2.45526E-05	9.02180E-05	-1.8775E+00	-1.0448E+00
CCNA_02230	1.76756E-05	1.32539E-04	-2.9064E+00	-1.6315E+00
CCNA_02231	2.16182E-05	1.13986E-04	-2.3984E+00	-1.3419E+00
CCNA_02232	1.97071E-05	1.23390E-04	-2.6463E+00	-1.4832E+00
CCNA_02233	3.12972E-05	7.57558E-05	-1.2753E+00	-7.0137E-01
CCNA_02234	9.25343E-05	8.18949E-05	1.7613E-01	1.2637E-01
CCNA_02235	9.02229E-05	7.44049E-05	2.7800E-01	1.8446E-01
CCNA_02236	6.28322E-05	7.80523E-05	-3.1301E-01	-1.5258E-01
CCNA_02237	2.03993E-05	1.19743E-04	-2.5532E+00	-1.4301E+00
CCNA_02238	2.92085E-05	9.29799E-05	-1.6705E+00	-9.2672E-01
CCNA_02239	1.71610E-05	1.25724E-04	-2.8729E+00	-1.6124E+00
CCNA_02240	1.10761E-04	8.85969E-05	3.2203E-01	2.0957E-01
CCNA_02241	1.24322E-04	1.47129E-04	-2.4303E-01	-1.1267E-01
CCNA_02242	1.29764E-04	1.45755E-04	-1.6770E-01	-6.9715E-02
CCNA_02243	1.25664E-04	1.43879E-04	-1.9532E-01	-8.5464E-02
CCNA_02244	8.56573E-05	8.27805E-05	4.9205E-02	5.3983E-02
CCNA_02245	3.70276E-05	3.04179E-05	2.8344E-01	1.8756E-01
CCNA_02246	2.76712E-04	1.47099E-04	9.1154E-01	5.4576E-01
CCNA_02247	2.77040E-04	1.47099E-04	9.1325E-01	5.4673E-01
CCNA_02248	2.10317E-04	1.40539E-04	5.8153E-01	3.5756E-01
CCNA_02249	8.66565E-05	5.68356E-05	6.0837E-01	3.7286E-01
CCNA_02250	3.39156E-05	1.12320E-04	-1.7276E+00	-9.5927E-01
CCNA_02251	1.97998E-04	7.33917E-05	1.4317E+00	8.4237E-01
CCNA_02252	2.46531E-04	8.11669E-05	1.6027E+00	9.3990E-01
CCNA_02253	2.30469E-04	1.14046E-04	1.0149E+00	6.0468E-01
CCNA_02254	2.22683E-05	6.25845E-05	-1.4908E+00	-8.2424E-01
CCNA_02255	1.33315E-04	9.00529E-05	5.6591E-01	3.4865E-01
CCNA_02256	4.34134E-04	1.32764E-04	1.7092E+00	1.0006E+00
CCNA_02257	1.27997E-04	1.27293E-04	7.9060E-03	3.0431E-02
CCNA_02258	4.11619E-04	1.18219E-04	1.7998E+00	1.0523E+00
CCNA_02259	1.77629E-05	1.09829E-04	-2.6282E+00	-1.4729E+00
CCNA_02260	1.50954E-04	1.29875E-04	2.1694E-01	1.4964E-01
CCNA_02261	1.68720E-05	7.82625E-05	-2.2136E+00	-1.2364E+00
CCNA_02262	1.71459E-05	1.42130E-04	-3.0511E+00	-1.7141E+00
CCNA_02263	5.62110E-05	9.39930E-05	-7.4174E-01	-3.9707E-01
CCNA_02264	6.91645E-05	6.35826E-05	1.2129E-01	9.5092E-02
CCNA_02265	7.54727E-05	1.34175E-04	-8.3011E-01	-4.4747E-01
CCNA_02266	3.53632E-05	1.43714E-04	-2.0228E+00	-1.1277E+00
CCNA_02267	2.29365E-05	5.35409E-05	-1.2230E+00	-6.7153E-01
CCNA_02268	1.36875E-04	1.38618E-05	3.3029E+00	1.9095E+00
CCNA_02269	2.65679E-04	9.42857E-05	1.4945E+00	8.7819E-01
CCNA_02270	7.34051E-05	5.91322E-05	3.1181E-01	2.0374E-01
CCNA_02271	1.98600E-04	1.47099E-04	4.3303E-01	2.7287E-01
CCNA_02272	2.85196E-04	5.27829E-05	2.4336E+00	1.4138E+00
CCNA_02273	3.24279E-04	1.46611E-04	1.1452E+00	6.7900E-01
CCNA_02274	1.83988E-04	1.46296E-04	3.3068E-01	2.1450E-01
CCNA_02275	9.17578E-05	1.20231E-04	-3.8994E-01	-1.9645E-01
CCNA_02276	4.58518E-05	6.72901E-05	-5.5348E-01	-2.8972E-01
CCNA_02277	9.11860E-05	1.40389E-04	-6.2258E-01	-3.2912E-01

CCNA_02278	6.94835E-05	6.26595E-05	1.4903E-01	1.1091E-01
CCNA_02279	1.41769E-04	3.91763E-05	1.8552E+00	1.0839E+00
CCNA_02280	6.77409E-05	1.63009E-05	2.0545E+00	1.1975E+00
CCNA_02281	2.68080E-04	4.47150E-05	2.5836E+00	1.4993E+00
CCNA_02282	9.96431E-05	7.90505E-05	3.3390E-01	2.1634E-01
CCNA_02283	4.27525E-04	1.16816E-04	1.8717E+00	1.0933E+00
CCNA_02284	1.89156E-05	1.32734E-04	-2.8107E+00	-1.5770E+00
CCNA_02285	3.56552E-05	8.76888E-05	-1.2983E+00	-7.1446E-01
CCNA_02286	1.98826E-04	9.90064E-05	1.0058E+00	5.9952E-01
CCNA_02287	1.63718E-04	1.16343E-04	4.9276E-01	3.0693E-01
CCNA_02288	7.97494E-05	1.46768E-04	-8.8002E-01	-4.7593E-01
CCNA_02289	5.54165E-05	8.58651E-05	-6.3180E-01	-3.3438E-01
CCNA_02290	1.25809E-04	6.85435E-05	8.7602E-01	5.2550E-01
CCNA_02291	2.24775E-04	1.15532E-04	9.6011E-01	5.7345E-01
CCNA_02292	1.68407E-04	9.49762E-05	8.2623E-01	4.9710E-01
CCNA_02293	3.20918E-05	9.69425E-05	-1.5949E+00	-8.8361E-01
CCNA_02294	2.31983E-05	1.25852E-04	-2.4395E+00	-1.3653E+00
CCNA_02295	8.11338E-05	1.36427E-04	-7.4977E-01	-4.0166E-01
CCNA_02296	1.37134E-04	3.76978E-05	1.8628E+00	1.0882E+00
CCNA_02297	1.62776E-04	4.38069E-05	1.8934E+00	1.1057E+00
CCNA_02298	1.69033E-04	1.18197E-04	5.1605E-01	3.2021E-01
CCNA_02299	2.36828E-04	4.63211E-05	2.3539E+00	1.3683E+00
CCNA_02300	5.87602E-05	4.61034E-05	3.4980E-01	2.2541E-01
CCNA_02301	3.74911E-05	5.91622E-05	-6.5820E-01	-3.4943E-01
CCNA_02302	2.10810E-04	4.20882E-05	2.3242E+00	1.3514E+00
CCNA_02303	4.33237E-05	1.05476E-04	-1.2837E+00	-7.0613E-01
CCNA_02304	2.61387E-05	4.57957E-05	-8.0910E-01	-4.3549E-01
CCNA_02305	2.62233E-04	1.04793E-04	1.3232E+00	7.8053E-01
CCNA_02306	1.29812E-04	6.87986E-05	9.1584E-01	5.4821E-01
CCNA_02307	2.56271E-05	1.00620E-04	-1.9731E+00	-1.0993E+00
CCNA_02308	2.43576E-04	5.52446E-05	2.1403E+00	1.2465E+00
CCNA_02309	2.42192E-04	5.26553E-05	2.2013E+00	1.2813E+00
CCNA_02310	6.65341E-05	6.65096E-05	4.3292E-04	2.6169E-02
CCNA_04009	2.19842E-04	5.62652E-05	1.9660E+00	1.1471E+00
CCNA_02312	1.70421E-04	1.05408E-04	6.9304E-01	4.2115E-01
CCNA_02313	3.11407E-05	9.29123E-05	-1.5770E+00	-8.7343E-01
CCNA_02314	1.71378E-04	7.38270E-05	1.2148E+00	7.1872E-01
CCNA_02315	6.43701E-05	8.43115E-05	-3.8940E-01	-1.9614E-01
CCNA_02316	3.10565E-05	1.82597E-05	7.6577E-01	4.6263E-01
CCNA_02317	2.98135E-05	1.14039E-04	-1.9354E+00	-1.0778E+00
CCNA_02318	2.47985E-04	5.83441E-05	2.0874E+00	1.2163E+00
CCNA_02319	5.64307E-05	4.88653E-05	2.0753E-01	1.4427E-01
CCNA_02320	1.04630E-04	7.17406E-05	5.4433E-01	3.3634E-01
CCNA_02321	1.52405E-04	1.17349E-04	3.7705E-01	2.4094E-01
CCNA_02322	1.46900E-05	1.35954E-04	-3.2100E+00	-1.8047E+00
CCNA_02323	1.29652E-04	1.42708E-04	-1.3846E-01	-5.3041E-02
CCNA_02324	3.92135E-04	5.03663E-05	2.9606E+00	1.7143E+00
CCNA_02325	2.09308E-04	4.87752E-05	2.1012E+00	1.2242E+00
CCNA_02326	5.64819E-05	2.91345E-05	9.5476E-01	5.7040E-01
CCNA_02327	2.73739E-04	1.35346E-04	1.0161E+00	6.0537E-01
CCNA_02328	2.75719E-04	1.29822E-04	1.0866E+00	6.4558E-01
CCNA_02329	1.20193E-04	1.29132E-04	-1.0354E-01	-3.3123E-02
CCNA_02330	1.26555E-05	1.10024E-04	-3.1197E+00	-1.7532E+00

CCNA_02331	1.30949E-05	3.62118E-05	-1.4674E+00	-8.1091E-01
CCNA_02332	8.30209E-05	1.44104E-04	-7.9559E-01	-4.2778E-01
CCNA_02333	2.15279E-04	7.79548E-05	1.4654E+00	8.6160E-01
CCNA_02334	2.07063E-04	6.81232E-05	1.6037E+00	9.4048E-01
CCNA_02335	1.54307E-04	8.17899E-05	9.1571E-01	5.4813E-01
CCNA_02336	1.69054E-04	5.95299E-05	1.5056E+00	8.8456E-01
CCNA_02337	1.13388E-04	1.47099E-04	-3.7555E-01	-1.8825E-01
CCNA_02338	9.21461E-05	5.06139E-05	8.6422E-01	5.1877E-01
CCNA_02339	1.46467E-04	1.41155E-04	5.3252E-02	5.6291E-02
CCNA_02340	1.66583E-04	1.04898E-04	6.6718E-01	4.0640E-01
CCNA_02341	1.73755E-04	9.74228E-05	8.3464E-01	5.0190E-01
CCNA_02342	2.61595E-04	6.30123E-05	2.0535E+00	1.1970E+00
CCNA_02343	1.90119E-04	5.12294E-05	1.8917E+00	1.1047E+00
CCNA_02344	3.59739E-04	7.88704E-05	2.1893E+00	1.2744E+00
CCNA_02345	2.40205E-04	5.49444E-05	2.1280E+00	1.2395E+00
CCNA_02346	5.59248E-04	1.46648E-04	1.9311E+00	1.1272E+00
CCNA_02347	8.35957E-05	5.36985E-05	6.3840E-01	3.8999E-01
CCNA_02348	1.90901E-05	1.14767E-04	-2.5877E+00	-1.4498E+00
CCNA_02349	1.58608E-04	1.10189E-04	5.2541E-01	3.2555E-01
CCNA_02350	9.13786E-05	4.51728E-05	1.0162E+00	6.0544E-01
CCNA_02351	2.99128E-05	1.40164E-04	-2.2282E+00	-1.2448E+00
CCNA_02352	3.39487E-05	5.99877E-05	-8.2136E-01	-4.4248E-01
CCNA_02353	1.57425E-04	1.44307E-04	1.2548E-01	9.7480E-02
CCNA_02354	2.20953E-04	6.18565E-05	1.8366E+00	1.0733E+00
CCNA_02355	1.03706E-04	7.70242E-05	4.2902E-01	2.7058E-01
CCNA_02356	1.47511E-04	8.04390E-05	8.7475E-01	5.2478E-01
CCNA_02357	4.29746E-05	6.74327E-05	-6.5002E-01	-3.4477E-01
CCNA_02358	1.76485E-05	6.77930E-05	-1.9415E+00	-1.0813E+00
CCNA_02359	1.80699E-05	1.25754E-04	-2.7988E+00	-1.5702E+00
CCNA_02360	1.38597E-04	1.14527E-04	2.7515E-01	1.8283E-01
CCNA_02361	3.06237E-04	1.46776E-04	1.0610E+00	6.3097E-01
CCNA_02362	1.85902E-04	1.47099E-04	3.3771E-01	2.1851E-01
CCNA_02363	1.82851E-04	1.47099E-04	3.1383E-01	2.0489E-01
CCNA_02364	2.30641E-04	8.88296E-05	1.3764E+00	8.1087E-01
CCNA_02365	2.30087E-05	3.70298E-05	-6.8661E-01	-3.6564E-01
CCNA_02366	8.72313E-05	1.37822E-04	-6.5992E-01	-3.5042E-01
CCNA_02367	1.27609E-04	1.27428E-04	1.9933E-03	2.7059E-02
CCNA_02368	6.93180E-05	4.13227E-05	7.4610E-01	4.5140E-01
CCNA_02369	2.95697E-05	9.39180E-05	-1.6673E+00	-9.2487E-01
CCNA_02370	3.71871E-05	7.42173E-05	-9.9698E-01	-5.4263E-01
CCNA_02371	2.16255E-04	4.42722E-05	2.2880E+00	1.3307E+00
CCNA_02372	1.01666E-05	0.00000E+00	1.0404E+01	5.9592E+00
CCNA_02373	1.49459E-05	0.00000E+00	1.0960E+01	6.2761E+00
CCNA_02374	1.15603E-04	4.99610E-05	1.2101E+00	7.1603E-01
CCNA_02375	1.52125E-04	1.30685E-04	2.1911E-01	1.5088E-01
CCNA_02376	1.62493E-04	9.01805E-05	8.4940E-01	5.1032E-01
CCNA_02377	1.59715E-04	1.44930E-04	1.4010E-01	1.0582E-01
CCNA_02378	1.18276E-04	9.63121E-05	2.9629E-01	1.9489E-01
CCNA_02379	6.86017E-05	4.99460E-05	4.5772E-01	2.8695E-01
CCNA_02380	1.71962E-04	1.34858E-04	3.5059E-01	2.2586E-01
CCNA_02381	1.78499E-04	1.47129E-04	2.7879E-01	1.8491E-01
CCNA_02382	1.27542E-04	1.44277E-04	-1.7790E-01	-7.5531E-02
CCNA_02383	1.10634E-04	1.19465E-04	-1.1084E-01	-3.7288E-02

CCNA_02384	1.64603E-04	1.50221E-04	1.3186E-01	1.0112E-01
CCNA_02385	3.49569E-05	3.22641E-05	1.1544E-01	9.1753E-02
CCNA_02386	3.56094E-04	5.74510E-05	2.6317E+00	1.5267E+00
CCNA_02387	1.69623E-05	1.42273E-04	-3.0681E+00	-1.7237E+00
CCNA_02388	6.54897E-05	1.47099E-04	-1.1675E+00	-6.3985E-01
CCNA_02389	4.59584E-04	1.09911E-04	2.0639E+00	1.2029E+00
CCNA_02390	4.19161E-04	1.47429E-04	1.5074E+00	8.8557E-01
CCNA_02391	2.25934E-05	3.77578E-05	-7.4097E-01	-3.9664E-01
CCNA_02392	1.18884E-04	7.34968E-05	6.9369E-01	4.2152E-01
CCNA_04015	3.56130E-04	1.03577E-04	1.7816E+00	1.0419E+00
CCNA_02394	2.42622E-04	8.52046E-05	1.5096E+00	8.8681E-01
CCNA_02395	2.61342E-04	6.85660E-05	1.9302E+00	1.1267E+00
CCNA_02396	1.77653E-04	1.40209E-04	3.4143E-01	2.2063E-01
CCNA_02397	1.74252E-04	7.17181E-05	1.2806E+00	7.5624E-01
CCNA_02398	5.36128E-04	5.40588E-05	3.3098E+00	1.9134E+00
CCNA_02399	3.86708E-05	1.09431E-04	-1.5007E+00	-8.2989E-01
CCNA_02400	1.40165E-04	9.70476E-05	5.3028E-01	3.2833E-01
CCNA_02401	1.37492E-04	8.64505E-05	6.6931E-01	4.0762E-01
CCNA_02402	3.07293E-04	3.74126E-05	3.0377E+00	1.7583E+00
CCNA_02403	5.11578E-04	9.79032E-05	2.3854E+00	1.3863E+00
CCNA_02404	1.31482E-04	1.46528E-04	-1.5635E-01	-6.3243E-02
CCNA_02405	2.05950E-05	9.05332E-05	-2.1361E+00	-1.1922E+00
CCNA_02406	1.34615E-04	1.18857E-04	1.7955E-01	1.2832E-01
CCNA_02407	2.97154E-04	6.57141E-05	2.1768E+00	1.2673E+00
CCNA_02408	1.99692E-04	4.35967E-05	2.1953E+00	1.2778E+00
CCNA_02409	2.10000E-04	7.03222E-05	1.5782E+00	9.2594E-01
CCNA_02410	9.36720E-05	4.67639E-05	1.0020E+00	5.9736E-01
CCNA_02411	5.51907E-04	1.46378E-04	1.9147E+00	1.1178E+00
CCNA_02412	5.62276E-04	1.45560E-04	1.9496E+00	1.1377E+00
CCNA_02413	1.35042E-04	7.75720E-05	7.9970E-01	4.8197E-01
CCNA_02414	9.90683E-05	1.36111E-04	-4.5833E-01	-2.3545E-01
CCNA_02415	1.13238E-04	1.40915E-04	-3.1551E-01	-1.5400E-01
CCNA_02416	5.14841E-04	1.38738E-04	1.8917E+00	1.1047E+00
CCNA_02417	7.86358E-05	1.03697E-04	-3.9917E-01	-2.0171E-01
CCNA_02418	1.96981E-04	1.47099E-04	4.2122E-01	2.6613E-01
CCNA_02419	2.05649E-05	1.47099E-04	-2.8384E+00	-1.5927E+00
CCNA_02420	6.09271E-05	1.47309E-04	-1.2737E+00	-7.0043E-01
CCNA_02421	7.06813E-05	6.55640E-05	1.0832E-01	8.7696E-02
CCNA_02422	1.84551E-05	1.27616E-04	-2.7896E+00	-1.5649E+00
CCNA_02423	1.92075E-05	6.01303E-05	-1.6464E+00	-9.1297E-01
CCNA_02424	2.05618E-05	6.33500E-05	-1.6233E+00	-8.9983E-01
CCNA_02425	3.53395E-04	6.46108E-05	2.4513E+00	1.4238E+00
CCNA_02426	2.59924E-04	5.49293E-05	2.2423E+00	1.3046E+00
CCNA_02427	2.37292E-04	8.81016E-05	1.4293E+00	8.4103E-01
CCNA_02428	3.76349E-04	8.58276E-05	2.1324E+00	1.2420E+00
CCNA_02429	1.87771E-04	8.86720E-05	1.0823E+00	6.4315E-01
CCNA_02430	8.41555E-05	4.65087E-05	8.5538E-01	5.1372E-01
CCNA_02431	5.29937E-05	5.79989E-05	-1.3031E-01	-4.8390E-02
CCNA_02432	9.27330E-05	1.17161E-04	-3.3738E-01	-1.6648E-01
CCNA_02433	1.35172E-04	8.87695E-05	6.0657E-01	3.7183E-01
CCNA_02434	4.01576E-05	4.50302E-05	-1.6535E-01	-6.8374E-02
CCNA_02435	2.07891E-04	5.43364E-05	1.9357E+00	1.1298E+00
CCNA_02436	2.85446E-04	5.81190E-05	2.2960E+00	1.3353E+00

CCNA_02437	1.98019E-04	9.65372E-05	1.0364E+00	6.1695E-01
CCNA_02438	2.42101E-04	1.27023E-04	9.3046E-01	5.5654E-01
CCNA_02439	1.04744E-04	1.12718E-04	-1.0590E-01	-3.4470E-02
CCNA_02440	3.95740E-04	5.04488E-05	2.9715E+00	1.7205E+00
CCNA_02441	3.45759E-04	6.78605E-05	2.3490E+00	1.3655E+00
CCNA_02442	7.11659E-05	1.45973E-04	-1.0365E+00	-5.6514E-01
CCNA_02443	8.64850E-05	9.22294E-05	-9.2844E-02	-2.7024E-02
CCNA_02444	2.87860E-04	8.44541E-05	1.7690E+00	1.0348E+00
CCNA_02445	2.88790E-04	1.02977E-04	1.4876E+00	8.7428E-01
CCNA_02446	1.83772E-04	6.36802E-05	1.5288E+00	8.9779E-01
CCNA_02447	4.32936E-04	1.35789E-04	1.6727E+00	9.7984E-01
CCNA_02448	2.28350E-04	1.40682E-04	6.9875E-01	4.2441E-01
CCNA_02449	3.81122E-04	7.70842E-05	2.3056E+00	1.3408E+00
CCNA_02450	3.73990E-04	9.60119E-05	1.9616E+00	1.1446E+00
CCNA_02451	4.92708E-05	1.47271E-04	-1.5797E+00	-8.7492E-01
CCNA_02452	5.52389E-05	1.13844E-04	-1.0433E+00	-5.6906E-01
CCNA_02453	5.69514E-05	1.36532E-04	-1.2614E+00	-6.9345E-01
CCNA_02454	7.59482E-05	7.93582E-05	-6.3443E-02	-1.0258E-02
CCNA_02455	3.03853E-05	6.56315E-05	-1.1110E+00	-6.0768E-01
CCNA_02456	4.03562E-05	1.15578E-04	-1.5180E+00	-8.3975E-01
CCNA_02457	4.19423E-05	1.47099E-04	-1.8103E+00	-1.0064E+00
CCNA_02458	4.13193E-05	1.43796E-04	-1.7991E+00	-1.0001E+00
CCNA_02459	1.28141E-04	1.39076E-04	-1.1818E-01	-4.1472E-02
CCNA_02460	2.03424E-04	8.39963E-05	1.2760E+00	7.5359E-01
CCNA_02461	1.08512E-04	1.35233E-04	-3.1763E-01	-1.5521E-01
CCNA_02462	4.91712E-04	1.02376E-04	2.2638E+00	1.3169E+00
CCNA_02463	2.59521E-05	1.40854E-04	-2.4402E+00	-1.3657E+00
CCNA_02464	2.57637E-04	6.26445E-05	2.0399E+00	1.1892E+00
CCNA_02465	3.60392E-04	1.33822E-04	1.4292E+00	8.4095E-01
CCNA_02466	4.37150E-05	4.79572E-05	-1.3374E-01	-5.0349E-02
CCNA_02467	1.02397E-04	9.17791E-05	1.5786E-01	1.1595E-01
CCNA_02468	1.53359E-04	1.08876E-04	4.9416E-01	3.0773E-01
CCNA_02469	1.51445E-04	1.46213E-04	5.0676E-02	5.4822E-02
CCNA_02470	1.81370E-04	7.96359E-05	1.1873E+00	7.0303E-01
CCNA_02471	3.44420E-04	1.21169E-04	1.5071E+00	8.8537E-01
CCNA_02472	1.94152E-04	7.46075E-05	1.3797E+00	8.1272E-01
CCNA_02473	1.96981E-05	1.40457E-04	-2.8339E+00	-1.5902E+00
CCNA_02474	2.16784E-05	1.02736E-04	-2.2445E+00	-1.2541E+00
CCNA_02475	2.70121E-04	1.47099E-04	8.7676E-01	5.2592E-01
CCNA_02476	3.08948E-04	1.46986E-04	1.0716E+00	6.3705E-01
CCNA_02477	4.61648E-05	5.53796E-05	-2.6266E-01	-1.2387E-01
CCNA_02478	1.66553E-05	1.33732E-04	-3.0051E+00	-1.6878E+00
CCNA_02479	2.44112E-05	1.14752E-04	-2.2328E+00	-1.2474E+00
CCNA_02480	3.25703E-05	1.47099E-04	-2.1751E+00	-1.2145E+00
CCNA_02481	1.93339E-05	6.08133E-05	-1.6532E+00	-9.1686E-01
CCNA_02482	1.03911E-04	1.26325E-04	-2.8184E-01	-1.3480E-01
CCNA_02483	2.22111E-05	7.44049E-05	-1.7441E+00	-9.6868E-01
CCNA_02484	5.43661E-05	1.26745E-04	-1.2212E+00	-6.7047E-01
CCNA_02485	3.85866E-05	6.15263E-05	-6.7317E-01	-3.5797E-01
CCNA_02486	2.51756E-05	1.00537E-04	-1.9976E+00	-1.1132E+00
CCNA_02487	2.37430E-05	1.41725E-04	-2.5774E+00	-1.4439E+00
CCNA_02488	2.29936E-05	1.01948E-04	-2.1484E+00	-1.1993E+00
CCNA_02489	2.97111E-05	5.47867E-05	-8.8287E-01	-4.7756E-01

CCNA_02490	5.24610E-05	1.72391E-05	1.6050E+00	9.4123E-01
CCNA_02491	9.51046E-06	3.30221E-07	4.8160E+00	2.7724E+00
CCNA_02492	2.09742E-05	6.55189E-05	-1.6433E+00	-9.1119E-01
CCNA_02493	2.24338E-05	4.50152E-05	-1.0048E+00	-5.4708E-01
CCNA_02494	4.58910E-05	1.19300E-04	-1.3783E+00	-7.6010E-01
CCNA_02495	2.16905E-05	1.21334E-04	-2.4837E+00	-1.3905E+00
CCNA_02496	5.99881E-05	8.82442E-05	-5.5688E-01	-2.9165E-01
CCNA_02497	1.67613E-04	8.89797E-05	9.1349E-01	5.4686E-01
CCNA_02498	1.72853E-04	1.06151E-04	7.0334E-01	4.2702E-01
CCNA_02499	7.58489E-05	1.08958E-04	-5.2262E-01	-2.7211E-01
CCNA_02500	1.78140E-05	7.06074E-05	-1.9867E+00	-1.1071E+00
CCNA_02501	2.59250E-05	9.95543E-05	-1.9411E+00	-1.0810E+00
CCNA_02502	2.35444E-05	5.01561E-05	-1.0911E+00	-5.9629E-01
CCNA_02503	2.72914E-05	5.13494E-05	-9.1195E-01	-4.9414E-01
CCNA_02504	1.00079E-04	4.67413E-05	1.0982E+00	6.5219E-01
CCNA_02505	4.20868E-05	7.73319E-05	-8.7773E-01	-4.7463E-01
CCNA_02506	3.80858E-04	1.11833E-04	1.7678E+00	1.0341E+00
CCNA_02507	2.22439E-04	3.75477E-05	2.5663E+00	1.4895E+00
CCNA_02508	6.36237E-05	1.35789E-04	-1.0937E+00	-5.9781E-01
CCNA_02509	8.87633E-05	6.65321E-05	4.1580E-01	2.6304E-01
CCNA_02510	3.07826E-05	1.39609E-04	-2.1811E+00	-1.2179E+00
CCNA_02511	3.12400E-05	9.21168E-05	-1.5600E+00	-8.6374E-01
CCNA_02512	2.54197E-04	1.30085E-04	9.6643E-01	5.7706E-01
CCNA_02513	2.26084E-04	5.03738E-05	2.1659E+00	1.2611E+00
CCNA_02514	3.14664E-04	1.13866E-04	1.4664E+00	8.6217E-01
CCNA_02515	1.38194E-04	9.96894E-05	4.7110E-01	2.9458E-01
CCNA_02516	5.25934E-05	6.58041E-05	-3.2338E-01	-1.5849E-01
CCNA_02517	6.84632E-05	4.09775E-05	7.4030E-01	4.4810E-01
CCNA_02518	2.07274E-05	7.02621E-05	-1.7612E+00	-9.7842E-01
CCNA_02519	3.57816E-05	9.80533E-05	-1.4543E+00	-8.0345E-01
CCNA_02520	2.02278E-05	6.23818E-05	-1.6247E+00	-9.0063E-01
CCNA_02521	2.04415E-05	5.49969E-05	-1.4278E+00	-7.8834E-01
CCNA_02522	1.84882E-05	5.99127E-05	-1.6962E+00	-9.4138E-01
CCNA_02523	3.10640E-04	3.08457E-05	3.3318E+00	1.9260E+00
CCNA_04001	4.31793E-05	1.26527E-04	-1.5510E+00	-8.5859E-01
CCNA_02525	3.07404E-05	7.33542E-05	-1.2547E+00	-6.8963E-01
CCNA_02526	1.56312E-04	1.30663E-04	2.5852E-01	1.7335E-01
CCNA_02527	1.77719E-05	1.06129E-04	-2.5780E+00	-1.4443E+00
CCNA_02528	1.87209E-04	1.34895E-04	4.7275E-01	2.9552E-01
CCNA_02529	4.17617E-05	1.02564E-04	-1.2963E+00	-7.1331E-01
CCNA_02530	1.50049E-04	1.00928E-04	5.7203E-01	3.5214E-01
CCNA_02531	9.12823E-05	4.18631E-05	1.1244E+00	6.6717E-01
CCNA_02532	5.83840E-05	1.19510E-04	-1.0335E+00	-5.6346E-01
CCNA_02533	8.91726E-05	4.46625E-05	9.9734E-01	5.9469E-01
CCNA_02534	1.59983E-04	6.75078E-05	1.2447E+00	7.3573E-01
CCNA_02535	1.53720E-04	6.38078E-05	1.2684E+00	7.4924E-01
CCNA_04018	1.56862E-05	1.23495E-04	-2.9767E+00	-1.6716E+00
CCNA_02537	8.39990E-05	8.82817E-05	-7.1813E-02	-1.5031E-02
CCNA_02538	1.56435E-04	8.60377E-05	8.6242E-01	5.1774E-01
CCNA_02539	2.16444E-04	7.40597E-05	1.5471E+00	9.0820E-01
CCNA_02540	4.73898E-05	8.50470E-05	-8.4372E-01	-4.5523E-01
CCNA_02541	4.74319E-05	8.61728E-05	-8.6141E-01	-4.6532E-01
CCNA_02542	1.40397E-04	1.47099E-04	-6.7319E-02	-1.2468E-02

CCNA_02543	3.28138E-04	1.40262E-04	1.2261E+00	7.2515E-01
CCNA_02544	4.35495E-05	1.47954E-04	-1.7644E+00	-9.8027E-01
CCNA_02545	4.65109E-05	9.95167E-05	-1.0974E+00	-5.9989E-01
CCNA_02546	2.71906E-04	1.19870E-04	1.1816E+00	6.9974E-01
CCNA_02547	2.10046E-04	1.42295E-04	5.6176E-01	3.4628E-01
CCNA_02548	3.03715E-04	1.02504E-04	1.5669E+00	9.1952E-01
CCNA_02549	1.96797E-04	1.28081E-04	6.1959E-01	3.7926E-01
CCNA_02550	1.70586E-05	1.20141E-04	-2.8160E+00	-1.5800E+00
CCNA_02551	2.59732E-05	1.18542E-04	-2.1902E+00	-1.2231E+00
CCNA_02552	1.04639E-04	6.08358E-05	7.8229E-01	4.7205E-01
CCNA_02553	5.19310E-04	1.47099E-04	1.8197E+00	1.0637E+00
CCNA_02554	1.11128E-04	6.60968E-05	7.4944E-01	4.5331E-01
CCNA_02555	2.87270E-05	1.17018E-04	-2.0262E+00	-1.1296E+00
CCNA_02556	2.67647E-05	1.47099E-04	-2.4583E+00	-1.3760E+00
CCNA_02557	1.19374E-04	1.33725E-04	-1.6382E-01	-6.7500E-02
CCNA_02558	9.35847E-05	1.31443E-04	-4.9013E-01	-2.5359E-01
CCNA_02559	2.39206E-05	4.47300E-06	2.4167E+00	1.4041E+00
CCNA_02560	3.77890E-05	2.80313E-05	4.3066E-01	2.7152E-01
CCNA_02561	3.87912E-05	8.54673E-05	-1.1397E+00	-6.2400E-01
CCNA_02562	4.25436E-04	7.10126E-05	2.5827E+00	1.4988E+00
CCNA_02563	1.70508E-04	6.81757E-05	1.3224E+00	7.8004E-01
CCNA_02564	5.49379E-05	8.37562E-05	-6.0844E-01	-3.2106E-01
CCNA_02565	4.23998E-05	8.58576E-05	-1.0179E+00	-5.5457E-01
CCNA_02566	1.14065E-04	4.89328E-05	1.2208E+00	7.2212E-01
CCNA_02567	5.38885E-04	1.30618E-04	2.0446E+00	1.1919E+00
CCNA_02568	9.32597E-05	1.42566E-04	-6.1233E-01	-3.2328E-01
CCNA_02569	2.79653E-04	8.87996E-05	1.6549E+00	9.6968E-01
CCNA_02570	2.98827E-04	1.18625E-04	1.3328E+00	7.8601E-01
CCNA_02571	8.16786E-05	2.83315E-05	1.5272E+00	8.9686E-01
CCNA_02572	2.37217E-04	1.27608E-04	8.9442E-01	5.3599E-01
CCNA_02573	2.72162E-05	8.33659E-05	-1.6150E+00	-8.9506E-01
CCNA_02574	3.02890E-05	4.18406E-05	-4.6622E-01	-2.3995E-01
CCNA_02575	2.65369E-04	1.38003E-04	9.4324E-01	5.6383E-01
CCNA_02576	3.87057E-04	5.35184E-05	2.8543E+00	1.6536E+00
CCNA_02577	2.34722E-05	1.30310E-04	-2.4728E+00	-1.3843E+00
CCNA_02578	5.55368E-05	1.27135E-04	-1.1949E+00	-6.5548E-01
CCNA_02579	4.97012E-05	1.24869E-04	-1.3291E+00	-7.3201E-01
CCNA_02580	8.10676E-05	1.44847E-04	-8.3735E-01	-4.5160E-01
CCNA_02581	1.49248E-05	1.39616E-04	-3.2255E+00	-1.8135E+00
CCNA_02582	1.42235E-05	5.27604E-05	-1.8911E+00	-1.0525E+00
CCNA_02583	1.70917E-05	1.44157E-04	-3.0761E+00	-1.7283E+00
CCNA_04012	1.12013E-04	9.06383E-05	3.0539E-01	2.0008E-01
CCNA_02585	1.46500E-04	1.42776E-04	3.7105E-02	4.7082E-02
CCNA_02586	1.54722E-04	7.43073E-05	1.0580E+00	6.2927E-01
CCNA_02587	2.04478E-04	1.47099E-04	4.7511E-01	2.9687E-01
CCNA_02588	5.71753E-04	3.29171E-05	4.1182E+00	2.3744E+00
CCNA_02589	5.40459E-04	4.18931E-05	3.6891E+00	2.1298E+00
CCNA_02590	4.48990E-04	9.59519E-05	2.2262E+00	1.2955E+00
CCNA_02591	2.22894E-05	9.47510E-05	-2.0877E+00	-1.1646E+00
CCNA_02592	2.05348E-05	1.45448E-04	-2.8242E+00	-1.5847E+00
CCNA_02593	1.58981E-04	1.40044E-04	1.8293E-01	1.3024E-01
CCNA_02594	5.92417E-05	8.42815E-05	-5.0866E-01	-2.6415E-01
CCNA_02595	2.21148E-05	9.90139E-05	-2.1625E+00	-1.2073E+00

CCNA_02596	2.13118E-04	7.57858E-05	1.4915E+00	8.7651E-01
CCNA_02597	2.50101E-05	6.53688E-05	-1.3861E+00	-7.6453E-01
CCNA_02598	2.84260E-05	1.02391E-04	-1.8488E+00	-1.0284E+00
CCNA_02599	4.08197E-05	6.90463E-05	-7.5835E-01	-4.0655E-01
CCNA_02600	2.30653E-04	1.07127E-04	1.1063E+00	6.5683E-01
CCNA_02601	2.40440E-05	1.44660E-04	-2.5888E+00	-1.4504E+00
CCNA_02602	2.44082E-05	1.21544E-04	-2.3160E+00	-1.2948E+00
CCNA_02603	2.75562E-05	5.13344E-05	-8.9760E-01	-4.8596E-01
CCNA_02604	7.12953E-05	1.27526E-04	-8.3893E-01	-4.5250E-01
CCNA_02605	1.71399E-05	1.04643E-04	-2.6099E+00	-1.4624E+00
CCNA_02606	6.58990E-05	7.46601E-05	-1.8016E-01	-7.6818E-02
CCNA_02607	5.72222E-05	5.20624E-05	1.3620E-01	1.0360E-01
CCNA_02608	1.52751E-04	1.36291E-04	1.6444E-01	1.1970E-01
CCNA_02609	5.92417E-05	1.45898E-04	-1.3003E+00	-7.1559E-01
CCNA_02610	5.72674E-05	8.42515E-05	-5.5704E-01	-2.9175E-01
CCNA_02611	1.36267E-04	9.95543E-05	4.5281E-01	2.8415E-01
CCNA_02612	1.25330E-04	5.59425E-05	1.1636E+00	6.8947E-01
CCNA_02613	1.94994E-05	9.59293E-05	-2.2984E+00	-1.2848E+00
CCNA_02614	1.10120E-04	1.31751E-04	-2.5878E-01	-1.2166E-01
CCNA_02615	4.23426E-05	1.43421E-04	-1.7600E+00	-9.7779E-01
CCNA_02616	4.39046E-05	8.68032E-05	-9.8340E-01	-5.3489E-01
CCNA_02617	2.98707E-05	1.34993E-04	-2.1760E+00	-1.2150E+00
CCNA_02618	1.29797E-04	1.36014E-04	-6.7545E-02	-1.2597E-02
CCNA_02619	1.30955E-04	1.47054E-04	-1.6731E-01	-6.9489E-02
CCNA_02620	1.43677E-04	8.01387E-05	8.4215E-01	5.0618E-01
CCNA_02621	1.92858E-05	6.36127E-05	-1.7217E+00	-9.5594E-01
CCNA_02622	9.70608E-05	8.17073E-05	2.4834E-01	1.6754E-01
CCNA_02623	2.89828E-04	6.91739E-05	2.0668E+00	1.2045E+00
CCNA_02624	5.29952E-04	1.28156E-04	2.0479E+00	1.1938E+00
CCNA_02625	6.50834E-05	4.53304E-05	5.2164E-01	3.2340E-01
CCNA_02626	1.08341E-04	1.47099E-04	-4.4124E-01	-2.2571E-01
CCNA_02627	2.97313E-04	3.10633E-05	3.2584E+00	1.8841E+00
CCNA_02628	9.11078E-05	8.67582E-05	7.0497E-02	6.6125E-02
CCNA_02629	1.47081E-05	5.93423E-05	-2.0123E+00	-1.1217E+00
CCNA_02630	1.75161E-05	5.00961E-05	-1.5160E+00	-8.3861E-01
CCNA_02631	2.70193E-04	4.30264E-05	2.6505E+00	1.5374E+00
CCNA_02632	1.35386E-04	1.12253E-04	2.7026E-01	1.8004E-01
CCNA_02633	6.93481E-05	4.19757E-05	7.2411E-01	4.3886E-01
CCNA_02634	3.87039E-05	7.16656E-05	-8.8884E-01	-4.8096E-01
CCNA_02635	1.97450E-04	1.19007E-04	7.3037E-01	4.4244E-01
CCNA_02636	2.20008E-04	1.29424E-04	7.6538E-01	4.6240E-01
CCNA_02637	1.20244E-04	1.02421E-04	2.3138E-01	1.5787E-01
CCNA_02638	9.82737E-05	1.18497E-04	-2.7002E-01	-1.2806E-01
CCNA_02639	1.53061E-04	1.46821E-04	6.0005E-02	6.0142E-02
CCNA_02640	9.37141E-05	1.43729E-04	-6.1704E-01	-3.2596E-01
CCNA_02641	3.95466E-05	1.46491E-04	-1.8891E+00	-1.0514E+00
CCNA_02642	5.68701E-05	1.02249E-04	-8.4637E-01	-4.5674E-01
CCNA_02643	2.38484E-05	1.46543E-04	-2.6193E+00	-1.4678E+00
CCNA_02644	6.29857E-05	3.78254E-05	7.3545E-01	4.4533E-01
CCNA_02645	2.31227E-04	1.27308E-04	8.6092E-01	5.1689E-01
CCNA_02646	3.91403E-05	4.36268E-05	-1.5669E-01	-6.3437E-02
CCNA_02647	4.44433E-05	9.35352E-05	-1.0736E+00	-5.8631E-01
CCNA_02648	2.86909E-05	5.34659E-05	-8.9808E-01	-4.8623E-01

CCNA_02649	4.11418E-05	3.60992E-05	1.8844E-01	1.3339E-01
CCNA_02650	1.64128E-04	8.25854E-05	9.9075E-01	5.9093E-01
CCNA_02651	2.90430E-05	1.32584E-04	-2.1906E+00	-1.2233E+00
CCNA_02652	2.90340E-05	1.32809E-04	-2.1935E+00	-1.2250E+00
CCNA_02653	2.41042E-05	1.33492E-04	-2.4693E+00	-1.3823E+00
CCNA_02654	2.08809E-05	8.99403E-05	-2.1067E+00	-1.1755E+00
CCNA_02655	1.60986E-05	1.46243E-04	-3.1832E+00	-1.7894E+00
CCNA_02656	1.67246E-05	6.08358E-05	-1.8629E+00	-1.0364E+00
CCNA_02657	2.27047E-05	8.66606E-05	-1.9323E+00	-1.0760E+00
CCNA_02658	1.78923E-04	4.32815E-05	2.0473E+00	1.1934E+00
CCNA_02659	1.87154E-04	4.29813E-05	2.1222E+00	1.2362E+00
CCNA_02660	4.80639E-05	1.09056E-04	-1.1820E+00	-6.4817E-01
CCNA_02661	5.85314E-05	5.20924E-05	1.6800E-01	1.2173E-01
CCNA_02662	1.40186E-04	1.04815E-04	4.1942E-01	2.6511E-01
CCNA_02663	1.20319E-04	1.37222E-04	-1.8969E-01	-8.2252E-02
CCNA_02664	4.89798E-04	9.66498E-05	2.3412E+00	1.3611E+00
CCNA_02665	4.95495E-04	1.11585E-04	2.1506E+00	1.2524E+00
CCNA_02666	2.07003E-05	5.21675E-05	-1.3335E+00	-7.3454E-01
CCNA_02667	9.19444E-05	1.25184E-04	-4.4525E-01	-2.2800E-01
CCNA_02668	8.54436E-05	1.35106E-04	-6.6107E-01	-3.5107E-01
CCNA_02669	1.81069E-04	1.47617E-04	2.9463E-01	1.9395E-01
CCNA_02670	1.55674E-04	7.82775E-05	9.9174E-01	5.9149E-01
CCNA_02671	3.59260E-05	1.47099E-04	-2.0336E+00	-1.1338E+00
CCNA_02672	3.05870E-05	1.47376E-04	-2.2684E+00	-1.2677E+00
CCNA_02673	3.62992E-05	1.46401E-04	-2.0119E+00	-1.1214E+00
CCNA_02674	2.29190E-04	0.00000E+00	1.4898E+01	8.5221E+00
CCNA_02675	0.00000E+00	0.00000E+00	-1.3183E+00	-7.2586E-01
CCNA_02676	3.27957E-04	1.40517E-04	1.2227E+00	7.2320E-01
CCNA_02677	3.79314E-04	1.23938E-04	1.6137E+00	9.4618E-01
CCNA_02678	7.56081E-05	7.74144E-05	-3.4144E-02	6.4512E-03
CCNA_02679	3.14736E-04	1.01731E-04	1.6293E+00	9.5507E-01
CCNA_02680	2.41012E-05	3.67221E-05	-6.0766E-01	-3.2061E-01
CCNA_02681	2.00562E-05	4.93681E-05	-1.2995E+00	-7.1517E-01
CCNA_02682	4.12350E-05	5.47417E-05	-4.0886E-01	-2.0724E-01
CCNA_02683	2.39633E-04	9.37078E-05	1.3545E+00	7.9836E-01
CCNA_02684	2.36229E-04	6.07082E-05	1.9601E+00	1.1437E+00
CCNA_02685	1.77268E-05	7.36844E-05	-2.0553E+00	-1.1462E+00
CCNA_02686	3.79816E-05	7.58909E-05	-9.9865E-01	-5.4359E-01
CCNA_02687	1.53961E-04	9.34227E-05	7.2063E-01	4.3688E-01
CCNA_02688	1.17105E-05	1.13251E-05	4.7695E-02	5.3122E-02
CCNA_02689	2.80649E-05	1.03149E-04	-1.8778E+00	-1.0450E+00
CCNA_02690	2.73365E-05	1.36059E-04	-2.3152E+00	-1.2944E+00
CCNA_02691	1.71926E-04	8.61728E-05	9.9638E-01	5.9414E-01
CCNA_02692	3.81080E-05	4.57732E-05	-2.6453E-01	-1.2493E-01
CCNA_02693	4.56713E-05	1.28764E-04	-1.4954E+00	-8.2685E-01
CCNA_02694	1.85707E-04	9.12837E-05	1.0245E+00	6.1017E-01
CCNA_02695	2.00833E-04	1.24591E-04	6.8873E-01	4.1869E-01
CCNA_02696	1.55767E-04	4.27787E-05	1.8642E+00	1.0890E+00
CCNA_02697	1.62821E-05	8.43416E-05	-2.3728E+00	-1.3272E+00
CCNA_02698	2.32314E-05	6.55940E-05	-1.4975E+00	-8.2805E-01
CCNA_02699	3.63113E-05	1.05686E-04	-1.5413E+00	-8.5303E-01
CCNA_02700	1.34982E-05	7.38195E-05	-2.4511E+00	-1.3719E+00
CCNA_02701	1.28403E-04	8.81691E-05	5.4225E-01	3.3515E-01

CCNA_02702	2.63524E-05	6.03405E-05	-1.1952E+00	-6.5568E-01
CCNA_02703	1.62009E-05	1.04065E-04	-2.6832E+00	-1.5042E+00
CCNA_02704	1.67908E-05	5.37736E-05	-1.6792E+00	-9.3167E-01
CCNA_02705	3.56793E-05	1.34903E-04	-1.9187E+00	-1.0683E+00
CCNA_02706	1.84157E-04	5.95675E-05	1.6282E+00	9.5444E-01
CCNA_02707	1.08555E-04	5.19649E-05	1.0626E+00	6.3192E-01
CCNA_02708	1.10770E-04	7.97035E-05	4.7475E-01	2.9666E-01
CCNA_02709	6.07255E-05	8.17448E-05	-4.2889E-01	-2.1866E-01
CCNA_02710	1.86251E-04	7.46000E-05	1.3199E+00	7.7862E-01
CCNA_02711	1.98871E-04	9.58468E-05	1.0529E+00	6.2639E-01
CCNA_02712	1.19886E-04	1.46889E-04	-2.9310E-01	-1.4122E-01
CCNA_02713	2.04956E-05	6.91289E-05	-1.7539E+00	-9.7430E-01
CCNA_02714	1.66252E-05	4.62085E-05	-1.4748E+00	-8.1510E-01
CCNA_02715	1.59062E-04	1.47099E-04	1.1276E-01	9.0228E-02
CCNA_02716	2.75472E-05	6.42506E-05	-1.2218E+00	-6.7085E-01
CCNA_02717	1.66072E-05	6.97968E-05	-2.0712E+00	-1.1553E+00
CCNA_02718	9.86860E-05	4.99685E-05	9.8165E-01	5.8574E-01
CCNA_02719	6.06129E-04	4.83099E-05	3.6490E+00	2.1069E+00
CCNA_02720	4.63274E-05	7.04047E-05	-6.0387E-01	-3.1845E-01
CCNA_02721	4.85840E-04	7.57258E-05	2.6815E+00	1.5551E+00
CCNA_02722	9.42859E-05	1.10339E-04	-2.2688E-01	-1.0346E-01
CCNA_02723	7.26587E-05	9.84811E-05	-4.3876E-01	-2.2429E-01
CCNA_02724	8.03032E-05	1.47084E-04	-8.7313E-01	-4.7200E-01
CCNA_02725	1.77779E-05	1.08770E-04	-2.6130E+00	-1.4642E+00
CCNA_02726	4.69498E-04	9.52764E-05	2.3008E+00	1.3380E+00
CCNA_02727	4.02027E-04	5.20549E-05	2.9490E+00	1.7077E+00
CCNA_02728	3.89056E-05	1.46318E-04	-1.9110E+00	-1.0639E+00
CCNA_02729	1.85905E-05	6.25545E-05	-1.7505E+00	-9.7234E-01
CCNA_02730	1.04025E-04	6.41080E-05	6.9823E-01	4.2411E-01
CCNA_02731	1.29363E-04	8.04840E-05	6.8455E-01	4.1631E-01
CCNA_02732	6.78433E-05	6.10234E-05	1.5273E-01	1.1302E-01
CCNA_02733	4.42056E-05	5.81190E-05	-3.9487E-01	-1.9926E-01
CCNA_02734	2.99941E-05	1.10286E-04	-1.8785E+00	-1.0453E+00
CCNA_02735	1.49850E-05	1.43391E-04	-3.2582E+00	-1.8321E+00
CCNA_02736	2.79686E-05	8.06716E-05	-1.5282E+00	-8.4559E-01
CCNA_02737	1.72663E-05	7.31741E-05	-2.0833E+00	-1.1621E+00
CCNA_02738	1.73385E-05	4.27111E-05	-1.3006E+00	-7.1580E-01
CCNA_02739	0.00000E+00	0.00000E+00	-1.3183E+00	-7.2586E-01
CCNA_02740	1.93339E-05	1.09799E-04	-2.5055E+00	-1.4029E+00
CCNA_02741	2.00683E-05	1.16996E-04	-2.5433E+00	-1.4245E+00
CCNA_02742	3.63053E-05	7.34518E-05	-1.0166E+00	-5.5385E-01
CCNA_02743	1.28322E-04	6.86560E-05	9.0219E-01	5.4042E-01
CCNA_02744	1.32256E-04	7.99136E-05	7.2671E-01	4.4035E-01
CCNA_02745	1.34410E-04	8.45367E-05	6.6890E-01	4.0738E-01
CCNA_02746	5.88204E-05	6.16539E-05	-6.7977E-02	-1.2843E-02
CCNA_02747	3.74850E-05	1.09386E-04	-1.5450E+00	-8.5517E-01
CCNA_02748	2.82424E-05	1.47879E-04	-2.3884E+00	-1.3361E+00
CCNA_02749	7.60445E-05	5.64979E-05	4.2851E-01	2.7029E-01
CCNA_02750	1.74288E-05	7.07349E-05	-2.0209E+00	-1.1265E+00
CCNA_02751	2.20727E-05	9.57717E-05	-2.1173E+00	-1.1815E+00
CCNA_02752	1.93661E-04	1.11202E-04	8.0028E-01	4.8230E-01
CCNA_02753	5.14528E-05	9.49687E-05	-8.8423E-01	-4.7834E-01
CCNA_02754	3.24776E-04	1.47324E-04	1.1404E+00	6.7626E-01

CCNA_02755	3.81255E-04	8.82967E-05	2.1102E+00	1.2293E+00
CCNA_02756	8.94073E-05	1.09806E-04	-2.9654E-01	-1.4319E-01
CCNA_02757	1.00585E-04	1.20718E-04	-2.6328E-01	-1.2422E-01
CCNA_02758	2.51305E-05	1.17446E-04	-2.2244E+00	-1.2426E+00
CCNA_02759	3.79455E-05	9.77981E-05	-1.3659E+00	-7.5300E-01
CCNA_02760	5.03362E-05	1.13281E-04	-1.1702E+00	-6.4144E-01
CCNA_02761	8.79747E-05	1.45042E-04	-7.2134E-01	-3.8544E-01
CCNA_02762	1.32927E-04	3.17688E-05	2.0646E+00	1.2033E+00
CCNA_02763	9.31874E-05	4.83099E-05	9.4764E-01	5.6634E-01
CCNA_02764	5.36934E-04	9.66498E-05	2.4738E+00	1.4367E+00
CCNA_02765	1.25099E-04	1.15953E-04	1.0947E-01	8.8351E-02
CCNA_02766	1.90561E-04	1.47324E-04	3.7121E-01	2.3762E-01
CCNA_02767	3.23876E-04	9.91640E-05	1.7075E+00	9.9965E-01
CCNA_02768	6.18204E-04	1.47099E-04	2.0712E+00	1.2071E+00
CCNA_02769	1.84130E-05	8.23978E-05	-2.1618E+00	-1.2069E+00
CCNA_02770	4.88013E-05	0.00000E+00	1.2667E+01	7.2495E+00
CCNA_02771	1.00016E-04	0.00000E+00	1.3702E+01	7.8399E+00
CCNA_02772	1.44493E-05	0.00000E+00	1.0911E+01	6.2483E+00
CCNA_02773	2.12631E-05	0.00000E+00	1.1468E+01	6.5661E+00
CCNA_02774	1.05557E-04	6.92264E-05	6.0851E-01	3.7294E-01
CCNA_02775	4.53613E-05	8.47543E-05	-9.0186E-01	-4.8839E-01
CCNA_02776	1.90510E-05	1.40892E-04	-2.8865E+00	-1.6202E+00
CCNA_02777	9.38495E-05	6.70425E-05	4.8516E-01	3.0260E-01
CCNA_02778	2.65119E-05	7.04422E-05	-1.4098E+00	-7.7805E-01
CCNA_02779	2.67939E-04	1.29109E-04	1.0532E+00	6.2657E-01
CCNA_02780	5.88685E-05	1.47099E-04	-1.3212E+00	-7.2754E-01
CCNA_02781	5.01153E-04	5.75486E-05	3.1222E+00	1.8065E+00
CCNA_02782	3.96327E-04	5.51395E-05	2.8453E+00	1.6486E+00
CCNA_02783	3.86570E-04	5.65279E-05	2.7735E+00	1.6076E+00
CCNA_02784	1.84852E-05	3.68497E-05	-9.9534E-01	-5.4170E-01
CCNA_02785	1.99238E-05	9.14638E-05	-2.1986E+00	-1.2279E+00
CCNA_02786	7.39769E-05	1.01220E-04	-4.5240E-01	-2.3207E-01
CCNA_02787	8.52029E-05	1.32606E-04	-6.3821E-01	-3.3803E-01
CCNA_02788	1.28361E-04	1.25034E-04	3.7836E-02	4.7499E-02
CCNA_02789	1.59694E-04	7.15980E-05	1.1572E+00	6.8585E-01
CCNA_02790	1.57220E-04	1.36389E-04	2.0501E-01	1.4284E-01
CCNA_02791	2.79686E-05	1.35361E-04	-2.2749E+00	-1.2714E+00
CCNA_02792	3.23416E-05	8.88071E-05	-1.4573E+00	-8.0513E-01
CCNA_02793	2.50020E-04	1.11517E-04	1.1647E+00	6.9012E-01
CCNA_02794	3.10447E-04	1.07600E-04	1.5286E+00	8.9764E-01
CCNA_02795	7.19665E-05	7.65213E-05	-8.8618E-02	-2.4615E-02
CCNA_02796	1.10445E-04	1.05326E-04	6.8404E-02	6.4932E-02
CCNA_02797	9.25253E-05	5.80965E-05	6.7126E-01	4.0873E-01
CCNA_02798	1.26002E-04	5.63928E-05	1.1597E+00	6.8727E-01
CCNA_02799	1.18556E-04	4.98109E-05	1.2509E+00	7.3925E-01
CCNA_02800	9.79577E-05	9.58768E-05	3.0908E-02	4.3549E-02
CCNA_02801	2.09381E-05	1.35091E-04	-2.6896E+00	-1.5079E+00
CCNA_02802	1.70105E-05	1.13949E-04	-2.7437E+00	-1.5388E+00
CCNA_02803	1.59848E-04	8.85369E-05	8.5225E-01	5.1194E-01
CCNA_02804	3.35695E-05	5.17997E-05	-6.2587E-01	-3.3100E-01
CCNA_02805	1.59848E-04	8.85369E-05	8.5225E-01	5.1194E-01
CCNA_02806	1.55219E-04	4.05797E-05	1.9352E+00	1.1295E+00
CCNA_02807	1.65289E-05	5.37661E-05	-1.7016E+00	-9.4449E-01

CCNA_02808	1.68660E-05	9.10210E-05	-2.4319E+00	-1.3610E+00
CCNA_02809	3.28953E-05	1.46949E-04	-2.1593E+00	-1.2055E+00
CCNA_02810	2.44533E-05	6.85735E-05	-1.4876E+00	-8.2242E-01
CCNA_02811	1.82565E-05	1.04928E-04	-2.5228E+00	-1.4128E+00
CCNA_02812	5.39478E-05	0.00000E+00	1.2811E+01	7.3320E+00
CCNA_02813	4.74800E-05	0.00000E+00	1.2627E+01	7.2270E+00
CCNA_02814	0.00000E+00	0.00000E+00	-1.3183E+00	-7.2586E-01
CCNA_02815	5.93531E-05	3.74651E-05	6.6356E-01	4.0433E-01
CCNA_02816	3.84403E-04	1.17206E-04	1.7135E+00	1.0031E+00
CCNA_02817	1.71269E-04	1.47099E-04	2.1944E-01	1.5106E-01
CCNA_02818	1.91922E-04	1.46858E-04	3.8604E-01	2.4607E-01
CCNA_02819	4.87170E-05	1.03104E-04	-1.0816E+00	-5.9090E-01
CCNA_02820	1.43114E-04	1.37665E-04	5.5960E-02	5.7835E-02
CCNA_02821	1.91594E-05	9.39630E-05	-2.2939E+00	-1.2823E+00
CCNA_02822	1.79441E-04	1.15097E-04	6.4058E-01	3.9123E-01
CCNA_02823	6.42317E-05	5.34959E-05	2.6372E-01	1.7632E-01
CCNA_02824	7.44615E-05	5.54622E-05	4.2485E-01	2.6821E-01
CCNA_02825	6.87040E-05	1.47099E-04	-1.0983E+00	-6.0043E-01
CCNA_02826	4.79523E-04	1.44705E-04	1.7284E+00	1.0116E+00
CCNA_02827	7.68662E-05	0.00000E+00	1.3322E+01	7.6233E+00
CCNA_02828	1.27849E-05	0.00000E+00	1.0735E+01	6.1476E+00
CCNA_02829	1.40731E-05	0.00000E+00	1.0873E+01	6.2266E+00
CCNA_02830	1.00058E-04	6.07908E-07	7.3451E+00	4.2147E+00
CCNA_02831	5.43781E-05	1.30858E-04	-1.2669E+00	-6.9656E-01
CCNA_02832	2.84691E-04	1.47099E-04	9.5255E-01	5.6914E-01
CCNA_02833	1.35816E-04	1.45440E-04	-9.8814E-02	-3.0429E-02
CCNA_02834	2.26415E-05	6.49185E-05	-1.5196E+00	-8.4069E-01
CCNA_02835	2.61468E-04	1.94681E-05	3.7469E+00	2.1627E+00
CCNA_02836	5.66775E-05	1.05476E-04	-8.9609E-01	-4.8510E-01
CCNA_02837	1.75326E-04	1.37410E-04	3.5151E-01	2.2638E-01
CCNA_02838	9.36750E-05	1.37770E-04	-5.5656E-01	-2.9147E-01
CCNA_02839	1.22011E-05	2.37985E-05	-9.6396E-01	-5.2380E-01
CCNA_02840	1.52047E-05	6.00628E-05	-1.9818E+00	-1.1043E+00
CCNA_02841	1.54334E-05	4.31164E-05	-1.4821E+00	-8.1931E-01
CCNA_02842	3.89387E-05	6.39729E-05	-7.1631E-01	-3.8258E-01
CCNA_02843	2.43179E-05	1.47099E-04	-2.5966E+00	-1.4549E+00
CCNA_02844	2.47874E-05	8.67507E-05	-1.8072E+00	-1.0047E+00
CCNA_02845	1.42491E-04	5.88320E-05	1.2760E+00	7.5362E-01
CCNA_02846	1.05419E-04	1.24644E-04	-2.4173E-01	-1.1193E-01
CCNA_02847	7.93310E-05	1.02083E-04	-3.6384E-01	-1.8157E-01
CCNA_02848	7.77630E-05	7.59284E-05	3.4358E-02	4.5516E-02
CCNA_02849	1.25351E-04	8.73586E-05	5.2087E-01	3.2296E-01
CCNA_02850	4.12019E-05	6.34476E-05	-6.2292E-01	-3.2931E-01
CCNA_02851	1.00176E-04	2.46090E-05	2.0249E+00	1.1807E+00
CCNA_02852	3.37835E-04	1.46318E-04	1.2071E+00	7.1433E-01
CCNA_02853	7.78353E-05	8.35761E-05	-1.0274E-01	-3.2668E-02
CCNA_02854	8.60004E-05	1.02219E-04	-2.4930E-01	-1.1625E-01
CCNA_02855	7.55660E-05	6.66447E-05	1.8114E-01	1.2922E-01
CCNA_02856	3.88845E-05	8.66531E-05	-1.1561E+00	-6.3336E-01
CCNA_02857	3.30157E-05	9.16290E-05	-1.4726E+00	-8.1389E-01
CCNA_02858	1.03504E-04	7.01571E-05	5.6092E-01	3.4580E-01
CCNA_02859	1.88283E-04	8.69233E-05	1.1150E+00	6.6177E-01
CCNA_02860	6.42437E-05	1.47849E-04	-1.2025E+00	-6.5984E-01

CCNA_02861	1.42085E-05	4.77170E-05	-1.7477E+00	-9.7073E-01
CCNA_02862	5.52961E-05	1.07022E-04	-9.5268E-01	-5.1737E-01
CCNA_02863	4.84040E-05	1.09664E-04	-1.1799E+00	-6.4695E-01
CCNA_02864	5.22955E-05	1.31691E-04	-1.3324E+00	-7.3391E-01
CCNA_02865	3.00663E-05	1.18925E-04	-1.9838E+00	-1.1054E+00
CCNA_02866	4.05759E-05	6.61869E-05	-7.0598E-01	-3.7668E-01
CCNA_02867	7.34532E-05	4.77395E-05	6.2147E-01	3.8034E-01
CCNA_02868	8.89980E-05	1.05093E-04	-2.3988E-01	-1.1087E-01
CCNA_02869	1.16109E-04	7.74069E-05	5.8484E-01	3.5944E-01
CCNA_02870	7.52560E-05	1.24291E-04	-7.2387E-01	-3.8689E-01
CCNA_02871	4.23185E-05	9.64547E-05	-1.1886E+00	-6.5189E-01
CCNA_02872	1.75342E-05	9.70476E-05	-2.4684E+00	-1.3817E+00
CCNA_02873	1.76064E-05	9.85936E-05	-2.4853E+00	-1.3914E+00
CCNA_02874	5.17808E-05	7.03447E-05	-4.4209E-01	-2.2619E-01
CCNA_02875	1.27819E-05	7.99436E-05	-2.6447E+00	-1.4823E+00
CCNA_02877	1.28129E-04	1.16170E-04	1.4130E-01	1.0650E-01
CCNA_02876	9.43913E-05	9.33851E-05	1.5391E-02	3.4699E-02
CCNA_02878	1.44101E-05	8.72460E-05	-2.5978E+00	-1.4556E+00
CCNA_02879	1.66704E-05	2.78587E-05	-7.4097E-01	-3.9663E-01
CCNA_02880	1.65452E-04	1.28269E-04	3.6719E-01	2.3532E-01
CCNA_02881	1.60823E-04	1.13574E-04	5.0178E-01	3.1207E-01
CCNA_02882	1.39367E-04	1.47234E-04	-7.9259E-02	-1.9277E-02
CCNA_02883	1.77918E-04	1.03539E-04	7.8095E-01	4.7128E-01
CCNA_02884	1.87500E-05	1.41365E-04	-2.9143E+00	-1.6360E+00
CCNA_02885	1.34410E-05	1.39286E-04	-3.3731E+00	-1.8977E+00
CCNA_02886	6.98477E-05	6.43557E-05	1.1804E-01	9.3237E-02
CCNA_02887	1.46268E-05	4.10450E-05	-1.4886E+00	-8.2297E-01
CCNA_02888	2.66624E-05	1.05258E-04	-1.9810E+00	-1.1038E+00
CCNA_02889	2.00315E-04	1.34363E-04	5.7608E-01	3.5445E-01
CCNA_02890	2.32425E-04	3.15437E-05	2.8810E+00	1.6689E+00
CCNA_02891	2.10338E-04	1.01408E-04	1.0524E+00	6.2611E-01
CCNA_02892	6.05509E-05	9.78206E-05	-6.9203E-01	-3.6873E-01
CCNA_02893	3.09240E-05	5.45466E-05	-8.1882E-01	-4.4103E-01
CCNA_02894	1.57133E-05	1.30190E-04	-3.0504E+00	-1.7136E+00
CCNA_02895	1.89700E-04	6.88362E-05	1.4624E+00	8.5987E-01
CCNA_02896	1.70409E-04	8.86795E-05	9.4223E-01	5.6326E-01
CCNA_02897	1.18228E-04	2.86317E-05	2.0455E+00	1.1924E+00
CCNA_02898	6.43340E-05	1.25897E-04	-9.6861E-01	-5.2645E-01
CCNA_02899	1.40354E-04	5.79689E-05	1.2756E+00	7.5335E-01
CCNA_02900	5.01376E-05	1.72841E-05	1.5359E+00	9.0182E-01
CCNA_02901	6.87401E-05	2.33857E-05	1.5551E+00	9.1278E-01
CCNA_02902	1.11480E-04	1.32802E-04	-2.5253E-01	-1.1809E-01
CCNA_02903	1.14008E-04	1.40937E-04	-3.0595E-01	-1.4856E-01
CCNA_02904	1.10421E-04	1.19645E-04	-1.1580E-01	-4.0118E-02
CCNA_02905	1.46807E-04	1.41920E-05	3.3700E+00	1.9478E+00
CCNA_02906	3.10869E-04	9.86312E-05	1.6561E+00	9.7036E-01
CCNA_02907	6.28713E-06	2.85191E-07	4.4256E+00	2.5498E+00
CCNA_02908	3.81321E-05	1.47099E-04	-1.9477E+00	-1.0848E+00
CCNA_02909	4.44313E-05	6.90388E-05	-6.3589E-01	-3.3671E-01
CCNA_02910	2.13925E-05	1.47436E-04	-2.7848E+00	-1.5622E+00
CCNA_02911	2.24332E-04	1.43639E-04	6.4314E-01	3.9269E-01
CCNA_02912	1.85526E-04	4.73643E-05	1.9695E+00	1.1491E+00
CCNA_02913	2.55729E-05	8.93699E-05	-1.8051E+00	-1.0035E+00

CCNA_02914	1.61335E-04	7.93132E-05	1.0243E+00	6.1007E-01
CCNA_02915	1.44556E-04	1.05341E-04	4.5649E-01	2.8625E-01
CCNA_02916	1.27058E-04	1.47662E-04	-2.1685E-01	-9.7741E-02
CCNA_02917	2.64066E-05	7.67690E-05	-1.5396E+00	-8.5208E-01
CCNA_02918	1.99960E-05	1.07224E-04	-2.4227E+00	-1.3557E+00
CCNA_02919	3.39608E-05	1.01798E-04	-1.5838E+00	-8.7726E-01
CCNA_02920	2.88143E-05	3.20615E-05	-1.5425E-01	-6.2040E-02
CCNA_02921	2.61808E-05	3.57014E-05	-4.4761E-01	-2.2934E-01
CCNA_02922	1.97462E-04	1.04072E-04	9.2391E-01	5.5281E-01
CCNA_02923	8.76015E-05	1.47271E-04	-7.4947E-01	-4.0148E-01
CCNA_02924	4.12648E-04	1.34370E-04	1.6186E+00	9.4899E-01
CCNA_02925	1.98459E-04	9.33626E-06	4.4087E+00	2.5401E+00
CCNA_02926	2.32585E-05	1.43789E-04	-2.6280E+00	-1.4728E+00
CCNA_02927	1.06616E-04	9.55691E-05	1.5774E-01	1.1588E-01
CCNA_02928	3.54565E-05	6.94066E-05	-9.6905E-01	-5.2671E-01
CCNA_02929	8.28343E-05	1.45260E-04	-8.1036E-01	-4.3621E-01
CCNA_02930	1.00408E-04	6.38003E-05	6.5411E-01	3.9894E-01
CCNA_02931	1.33905E-04	1.03577E-04	3.7043E-01	2.3717E-01
CCNA_02932	6.47885E-05	3.06431E-05	1.0799E+00	6.4176E-01
CCNA_02933	1.34856E-04	1.16861E-04	2.0657E-01	1.4372E-01
CCNA_02934	1.30805E-04	1.13168E-04	2.0888E-01	1.4504E-01
CCNA_02935	2.39718E-05	1.46904E-04	-2.6154E+00	-1.4656E+00
CCNA_02936	3.52248E-05	9.70251E-05	-1.4618E+00	-8.0768E-01
CCNA_02937	2.03120E-05	1.41642E-04	-2.8017E+00	-1.5718E+00
CCNA_02938	8.70508E-05	1.33912E-04	-6.2139E-01	-3.2844E-01
CCNA_02939	6.21069E-05	8.86870E-05	-5.1402E-01	-2.6721E-01
CCNA_02940	3.81953E-05	1.20606E-04	-1.6588E+00	-9.2006E-01
CCNA_02941	6.10385E-05	8.02063E-05	-3.9406E-01	-1.9880E-01
CCNA_02942	2.44292E-05	1.31113E-04	-2.4240E+00	-1.3564E+00
CCNA_02943	3.32273E-04	1.37342E-04	1.2745E+00	7.5276E-01
CCNA_02944	2.84333E-04	1.39549E-04	1.0267E+00	6.1145E-01
CCNA_02945	2.23466E-05	1.39414E-04	-2.6411E+00	-1.4803E+00
CCNA_02946	2.23436E-05	1.38213E-04	-2.6288E+00	-1.4732E+00
CCNA_02947	2.55699E-05	1.22212E-04	-2.2568E+00	-1.2611E+00
CCNA_02948	2.28010E-05	5.51545E-05	-1.2744E+00	-7.0083E-01
CCNA_02949	1.58126E-04	4.82273E-05	1.7130E+00	1.0028E+00
CCNA_02950	1.82113E-05	4.84975E-05	-1.4131E+00	-7.7991E-01
CCNA_02951	1.59932E-05	5.94849E-05	-1.8950E+00	-1.0547E+00
CCNA_02952	1.67336E-05	8.71485E-05	-2.3806E+00	-1.3317E+00
CCNA_02953	1.58187E-05	1.18602E-04	-2.9062E+00	-1.6314E+00
CCNA_02954	2.53592E-05	1.01813E-04	-2.0053E+00	-1.1176E+00
CCNA_02955	3.87431E-05	1.30603E-04	-1.7531E+00	-9.7386E-01
CCNA_02956	1.40869E-04	1.25995E-04	1.6094E-01	1.1770E-01
CCNA_02957	1.22555E-04	5.77062E-05	1.0865E+00	6.4552E-01
CCNA_02958	1.55550E-04	1.22925E-04	3.3954E-01	2.1956E-01
CCNA_02959	1.58644E-04	1.12560E-04	4.9502E-01	3.0822E-01
CCNA_02960	7.44253E-05	1.12020E-04	-5.8993E-01	-3.1050E-01
CCNA_02961	8.04958E-05	8.31408E-05	-4.6719E-02	-7.2046E-04
CCNA_02962	1.85279E-04	1.47099E-04	3.3287E-01	2.1575E-01
CCNA_02963	3.40095E-04	1.17304E-04	1.5356E+00	9.0165E-01
CCNA_02964	3.34196E-04	1.03194E-04	1.6952E+00	9.9268E-01
CCNA_02965	2.00923E-05	5.80664E-05	-1.5310E+00	-8.4719E-01
CCNA_02966	2.34812E-05	6.53313E-05	-1.4762E+00	-8.1595E-01

CCNA_02967	2.43781E-05	1.34205E-04	-2.4607E+00	-1.3774E+00
CCNA_02968	2.85193E-05	1.44817E-04	-2.3441E+00	-1.3109E+00
CCNA_02969	2.84922E-05	4.35967E-05	-6.1375E-01	-3.2408E-01
CCNA_02970	1.92226E-05	5.08691E-05	-1.4040E+00	-7.7473E-01
CCNA_02971	2.35173E-05	1.43601E-04	-2.6102E+00	-1.4626E+00
CCNA_02972	3.01385E-05	9.76180E-05	-1.6955E+00	-9.4099E-01
CCNA_02973	2.49288E-05	1.47099E-04	-2.5608E+00	-1.4344E+00
CCNA_02974	4.10063E-05	8.65631E-05	-1.0779E+00	-5.8879E-01
CCNA_02975	1.47861E-04	8.95351E-05	7.2362E-01	4.3859E-01
CCNA_02976	1.51189E-04	1.03727E-04	5.4349E-01	3.3586E-01
CCNA_02977	1.43448E-04	8.48294E-05	7.5780E-01	4.5808E-01
CCNA_02978	2.87751E-05	1.46273E-04	-2.3457E+00	-1.3118E+00
CCNA_02979	6.77048E-05	1.29462E-04	-9.3522E-01	-5.0741E-01
CCNA_02980	2.57866E-05	9.90139E-05	-1.9410E+00	-1.0810E+00
CCNA_02981	4.81632E-05	1.47391E-04	-1.6136E+00	-8.9430E-01
CCNA_02982	1.01009E-04	1.25679E-04	-3.1530E-01	-1.5389E-01
CCNA_02983	1.76997E-05	1.47099E-04	-3.0548E+00	-1.7162E+00
CCNA_02984	4.69714E-05	1.05100E-04	-1.1619E+00	-6.3670E-01
CCNA_02985	5.91033E-05	6.08658E-05	-4.2499E-02	1.6863E-03
CCNA_02986	2.37123E-04	6.90013E-05	1.7808E+00	1.0415E+00
CCNA_02987	1.86236E-04	1.19885E-04	6.3541E-01	3.8828E-01
CCNA_02988	2.71235E-04	1.62709E-05	4.0585E+00	2.3404E+00
CCNA_02989	5.66233E-05	9.14638E-05	-6.9185E-01	-3.6862E-01
CCNA_02990	0.00000E+00	0.00000E+00	-1.3183E+00	-7.2586E-01
CCNA_02991	5.26687E-07	0.00000E+00	6.1412E+00	3.5281E+00
CCNA_02992	2.64246E-05	3.63544E-05	-4.6038E-01	-2.3662E-01
CCNA_02993	1.15058E-05	5.09292E-05	-2.1460E+00	-1.1979E+00
CCNA_02994	1.41658E-04	5.52821E-05	1.3574E+00	7.9999E-01
CCNA_02995	8.39328E-05	1.17626E-04	-4.8695E-01	-2.5177E-01
CCNA_02996	1.47954E-05	1.21964E-04	-3.0430E+00	-1.7095E+00
CCNA_02997	8.95789E-05	4.70641E-05	9.2835E-01	5.5534E-01
CCNA_02998	2.37409E-04	4.82649E-05	2.2981E+00	1.3365E+00
CCNA_02999	1.20879E-04	1.45890E-04	-2.7136E-01	-1.2883E-01
CCNA_03000	1.47842E-04	1.17266E-04	3.3421E-01	2.1652E-01
CCNA_03001	4.07625E-05	5.39087E-05	-4.0337E-01	-2.0411E-01
CCNA_03002	1.79889E-04	1.02159E-04	8.1622E-01	4.9139E-01
CCNA_03003	1.52372E-04	8.44466E-05	8.5139E-01	5.1145E-01
CCNA_03004	1.84310E-05	1.09116E-04	-2.5655E+00	-1.4371E+00
CCNA_03005	3.76987E-05	6.46033E-05	-7.7715E-01	-4.1727E-01
CCNA_03006	8.06733E-05	1.04635E-04	-3.7525E-01	-1.8808E-01
CCNA_03007	1.80717E-04	7.49528E-05	1.2696E+00	7.4992E-01
CCNA_03008	4.45818E-05	1.01288E-04	-1.1839E+00	-6.4926E-01
CCNA_03009	1.45621E-04	1.11502E-04	3.8508E-01	2.4553E-01
CCNA_03010	7.96501E-05	1.18467E-04	-5.7277E-01	-3.0072E-01
CCNA_03011	3.08247E-05	1.45958E-04	-2.2433E+00	-1.2534E+00
CCNA_03012	2.91604E-05	1.47519E-04	-2.3387E+00	-1.3078E+00
CCNA_03013	1.29014E-04	8.24353E-05	6.4610E-01	3.9438E-01
CCNA_03014	7.28694E-05	8.17448E-05	-1.6589E-01	-6.8680E-02
CCNA_03015	6.17849E-05	9.36778E-05	-6.0050E-01	-3.1653E-01
CCNA_03016	1.09247E-04	7.39696E-05	5.6248E-01	3.4669E-01
CCNA_03017	8.87091E-05	7.50503E-07	6.8708E+00	3.9442E+00
CCNA_03018	5.84171E-05	5.55372E-07	6.6975E+00	3.8454E+00
CCNA_03019	3.98774E-04	5.85468E-05	2.7677E+00	1.6043E+00

CCNA_03020	4.13085E-04	7.35418E-05	2.4897E+00	1.4457E+00
CCNA_03021	2.66895E-05	1.04680E-04	-1.9716E+00	-1.0984E+00
CCNA_03022	3.21911E-05	7.32641E-05	-1.1865E+00	-6.5069E-01
CCNA_03023	2.54013E-05	1.09986E-04	-2.1143E+00	-1.1798E+00
CCNA_03024	1.58442E-04	1.31969E-04	2.6371E-01	1.7631E-01
CCNA_03025	1.59806E-04	9.64622E-05	7.2820E-01	4.4120E-01
CCNA_03026	2.87571E-05	3.36826E-05	-2.2826E-01	-1.0425E-01
CCNA_03027	1.66583E-05	6.95116E-05	-2.0609E+00	-1.1494E+00
CCNA_03028	3.74489E-05	1.39256E-04	-1.8947E+00	-1.0546E+00
CCNA_03029	2.38234E-04	1.42025E-04	7.4617E-01	4.5145E-01
CCNA_03030	2.33075E-04	1.45012E-04	6.8457E-01	4.1631E-01
CCNA_03031	1.78411E-05	8.75612E-05	-2.2950E+00	-1.2828E+00
CCNA_03032	1.62520E-04	1.07104E-04	6.0153E-01	3.6896E-01
CCNA_03033	2.14819E-04	4.52178E-05	2.2479E+00	1.3079E+00
CCNA_03034	3.15651E-05	8.76363E-05	-1.4732E+00	-8.1420E-01
CCNA_03035	1.26299E-04	5.17472E-05	1.2871E+00	7.5994E-01
CCNA_03036	4.44253E-05	6.37478E-05	-5.2107E-01	-2.7123E-01
CCNA_03037	9.84091E-05	9.85786E-05	-2.5481E-03	2.4469E-02
CCNA_03038	7.91294E-05	6.87611E-05	2.0252E-01	1.4141E-01
CCNA_03039	1.35662E-04	8.90022E-05	6.0802E-01	3.7266E-01
CCNA_03040	1.56191E-04	6.90688E-05	1.1771E+00	6.9718E-01
CCNA_03041	4.09798E-04	1.06106E-04	1.9493E+00	1.1376E+00
CCNA_03042	7.07325E-05	9.43833E-05	-4.1621E-01	-2.1143E-01
CCNA_03043	3.44480E-04	1.03449E-04	1.7354E+00	1.0156E+00
CCNA_03044	2.95989E-04	1.38671E-04	1.0938E+00	6.4970E-01
CCNA_03045	3.67748E-05	9.00079E-05	-1.2913E+00	-7.1050E-01
CCNA_03046	3.15801E-05	8.84543E-05	-1.4859E+00	-8.2145E-01
CCNA_03047	1.09154E-04	1.16793E-04	-9.7652E-02	-2.9766E-02
CCNA_03048	1.17484E-04	1.17334E-04	1.7944E-03	2.6946E-02
CCNA_03049	1.79495E-05	8.73586E-06	1.0379E+00	6.1783E-01
CCNA_03050	6.66575E-05	1.31023E-04	-9.7500E-01	-5.3010E-01
CCNA_03051	1.40788E-04	7.21084E-05	9.6517E-01	5.7633E-01
CCNA_03052	1.14330E-04	1.35601E-04	-2.4620E-01	-1.1448E-01
CCNA_03053	1.30591E-04	1.47099E-04	-1.7177E-01	-7.2032E-02
CCNA_03055	1.13054E-04	8.57450E-05	3.9880E-01	2.5335E-01
CCNA_03054	1.30949E-04	5.42464E-05	1.2712E+00	7.5088E-01
CCNA_03056	3.75242E-05	1.32591E-04	-1.8211E+00	-1.0126E+00
CCNA_03057	1.04781E-04	1.47421E-04	-4.9261E-01	-2.5500E-01
CCNA_03058	2.83117E-05	6.42131E-05	-1.1815E+00	-6.4785E-01
CCNA_03059	2.13946E-04	8.23978E-05	1.3765E+00	8.1089E-01
CCNA_03060	1.82538E-04	7.67915E-05	1.2491E+00	7.3823E-01
CCNA_03061	1.51096E-04	1.47099E-04	3.8635E-02	4.7955E-02
CCNA_03062	5.12078E-04	6.60518E-05	2.9545E+00	1.7108E+00
CCNA_03063	7.57646E-05	1.47046E-04	-9.5669E-01	-5.1966E-01
CCNA_03064	1.26170E-04	2.24025E-05	2.4932E+00	1.4477E+00
CCNA_03065	1.27864E-04	4.39495E-05	1.5405E+00	9.0443E-01
CCNA_03066	2.23601E-04	5.91472E-05	1.9184E+00	1.1199E+00
CCNA_03067	2.09742E-05	6.50461E-05	-1.6328E+00	-9.0523E-01
CCNA_03068	4.77509E-05	1.26933E-04	-1.4105E+00	-7.7843E-01
CCNA_03069	1.92918E-05	1.42010E-04	-2.8798E+00	-1.6164E+00
CCNA_03070	2.16513E-05	7.06149E-05	-1.7055E+00	-9.4667E-01
CCNA_03071	4.94273E-05	6.06332E-05	-2.9489E-01	-1.4225E-01
CCNA_03072	2.18647E-04	6.46784E-05	1.7571E+00	1.0280E+00

CCNA_03073	2.23736E-05	7.73919E-05	-1.7903E+00	-9.9506E-01
CCNA_03074	5.61508E-05	3.78779E-05	5.6774E-01	3.4969E-01
CCNA_03075	6.43701E-05	3.40428E-05	9.1879E-01	5.4989E-01
CCNA_03076	4.44223E-05	5.57549E-05	-3.2791E-01	-1.6108E-01
CCNA_03077	1.44132E-05	1.04793E-04	-2.8619E+00	-1.6061E+00
CCNA_03078	1.81632E-05	5.93123E-05	-1.7073E+00	-9.4769E-01
CCNA_03079	1.61356E-04	8.03714E-05	1.0054E+00	5.9927E-01
CCNA_03080	6.26877E-05	9.51863E-05	-6.0262E-01	-3.1774E-01
CCNA_03081	2.86307E-05	1.24854E-04	-2.1245E+00	-1.1857E+00
CCNA_03082	1.31515E-04	2.91796E-05	2.1719E+00	1.2645E+00
CCNA_03083	5.98436E-05	8.95951E-05	-5.8227E-01	-3.0613E-01
CCNA_03084	4.06090E-05	2.87443E-05	4.9826E-01	3.1007E-01
CCNA_03085	3.07103E-05	6.52338E-05	-1.0869E+00	-5.9392E-01
CCNA_03086	1.46548E-04	7.33317E-05	9.9875E-01	5.9549E-01
CCNA_03087	1.47204E-04	7.33317E-05	1.0052E+00	5.9916E-01
CCNA_03088	3.10896E-05	6.38528E-05	-1.0384E+00	-5.6623E-01
CCNA_03089	2.47422E-05	9.86161E-05	-1.9948E+00	-1.1117E+00
CCNA_03090	1.07234E-03	7.35869E-05	3.8650E+00	2.2301E+00
CCNA_03091	1.14360E-03	4.75444E-05	4.5879E+00	2.6423E+00
CCNA_03092	4.42203E-04	1.46723E-04	1.5915E+00	9.3355E-01
CCNA_03093	9.20317E-05	7.75195E-05	2.4748E-01	1.6705E-01
CCNA_03094	1.31085E-04	1.38010E-04	-7.4319E-02	-1.6460E-02
CCNA_03095	1.85445E-04	1.19705E-04	6.3144E-01	3.8602E-01
CCNA_03096	7.83499E-05	2.68305E-05	1.5457E+00	9.0741E-01
CCNA_03097	9.91676E-05	1.25747E-04	-3.4262E-01	-1.6947E-01
CCNA_03098	1.00853E-04	1.30475E-04	-3.7156E-01	-1.8597E-01
CCNA_03099	1.11736E-04	7.82850E-05	5.1318E-01	3.1858E-01
CCNA_03100	1.18267E-04	1.35098E-04	-1.9201E-01	-8.3575E-02
CCNA_03101	1.25743E-05	2.93072E-05	-1.2208E+00	-6.7027E-01
CCNA_03102	1.49158E-05	7.21009E-05	-2.2730E+00	-1.2703E+00
CCNA_03103	1.35765E-05	4.53004E-05	-1.7383E+00	-9.6541E-01
CCNA_03104	1.95235E-05	7.38495E-05	-1.9193E+00	-1.0686E+00
CCNA_03105	2.00072E-04	1.05176E-04	9.2764E-01	5.5493E-01
CCNA_03106	7.95477E-05	8.13771E-05	-3.2880E-02	7.1717E-03
CCNA_03107	2.78732E-04	3.93789E-05	2.8231E+00	1.6359E+00
CCNA_03108	8.81673E-05	1.43616E-04	-7.0393E-01	-3.7551E-01
CCNA_03109	8.90221E-05	1.44810E-04	-7.0195E-01	-3.7438E-01
CCNA_03110	1.46184E-04	8.78089E-05	7.3525E-01	4.4522E-01
CCNA_03111	1.28087E-04	9.74003E-05	3.9505E-01	2.5121E-01
CCNA_03112	6.01386E-05	7.32566E-05	-2.8474E-01	-1.3646E-01
CCNA_03113	2.19120E-04	1.10639E-04	9.8578E-01	5.8809E-01
CCNA_03114	2.64189E-04	4.92856E-05	2.4221E+00	1.4072E+00
CCNA_03115	1.93739E-04	1.28133E-04	5.9641E-01	3.6604E-01
CCNA_03116	4.22132E-05	6.48210E-05	-6.1883E-01	-3.2698E-01
CCNA_03117	1.07552E-04	1.31263E-05	3.0337E+00	1.7560E+00
CCNA_03118	2.79237E-04	4.63061E-05	2.5920E+00	1.5041E+00
CCNA_03119	2.06645E-04	1.06181E-04	9.6054E-01	5.7370E-01
CCNA_03120	9.02320E-05	8.80115E-05	3.5871E-02	4.6379E-02
CCNA_03121	3.29856E-05	1.46070E-04	-2.1467E+00	-1.1983E+00
CCNA_03122	5.61478E-05	9.72277E-05	-7.9217E-01	-4.2584E-01
CCNA_03123	3.29405E-05	8.73361E-05	-1.4067E+00	-7.7629E-01
CCNA_03124	7.97434E-05	1.26828E-04	-6.6946E-01	-3.5586E-01
CCNA_03125	2.14377E-05	1.26707E-04	-2.5632E+00	-1.4358E+00

CCNA_03126	1.40881E-04	8.50696E-05	7.2767E-01	4.4089E-01
CCNA_03127	1.67209E-04	5.20174E-05	1.6844E+00	9.8650E-01
CCNA_03128	1.84130E-05	1.04538E-04	-2.5051E+00	-1.4027E+00
CCNA_03129	8.70809E-05	4.76420E-05	8.6995E-01	5.2203E-01
CCNA_03130	2.62347E-04	1.45252E-04	8.5286E-01	5.1229E-01
CCNA_03131	3.15031E-04	1.34903E-04	1.2235E+00	7.2366E-01
CCNA_03132	2.17323E-04	1.13986E-04	9.3090E-01	5.5680E-01
CCNA_03133	4.82836E-05	8.94150E-05	-8.8901E-01	-4.8106E-01
CCNA_03134	4.57465E-05	8.24953E-05	-8.5069E-01	-4.5921E-01
CCNA_03135	2.51362E-04	1.47099E-04	7.7293E-01	4.6670E-01
CCNA_03136	1.54148E-04	1.44502E-04	9.3177E-02	7.9059E-02
CCNA_03137	3.99680E-05	6.81232E-05	-7.6935E-01	-4.1282E-01
CCNA_03138	4.14548E-05	1.46581E-04	-1.8221E+00	-1.0132E+00
CCNA_03139	3.94925E-05	6.41455E-05	-6.9983E-01	-3.7317E-01
CCNA_03140	5.06733E-05	3.84858E-05	3.9670E-01	2.5215E-01
CCNA_03141	3.04244E-05	1.47099E-04	-2.2734E+00	-1.2706E+00
CCNA_03142	7.37181E-05	1.47099E-04	-9.9671E-01	-5.4248E-01
CCNA_03143	2.75608E-04	6.31248E-05	2.1262E+00	1.2384E+00
CCNA_03144	1.06451E-04	4.77095E-05	1.1577E+00	6.8611E-01
CCNA_03145	9.75845E-05	6.57591E-05	5.6934E-01	3.5060E-01
CCNA_03146	1.30113E-04	1.37552E-04	-8.0264E-02	-1.9850E-02
CCNA_03147	7.77871E-05	7.85102E-05	-1.3430E-02	1.8263E-02
CCNA_03148	1.38940E-04	1.33244E-04	6.0337E-02	6.0331E-02
CCNA_03149	8.18772E-05	7.68966E-05	9.0455E-02	7.7507E-02
CCNA_03150	2.64938E-05	1.24644E-04	-2.2340E+00	-1.2481E+00
CCNA_03151	2.04084E-05	6.64871E-05	-1.7039E+00	-9.4575E-01
CCNA_03152	1.74336E-04	8.74336E-05	9.9551E-01	5.9364E-01
CCNA_03153	9.46050E-05	1.46153E-04	-6.2752E-01	-3.3194E-01
CCNA_03154	5.45166E-05	8.46718E-05	-6.3523E-01	-3.3634E-01
CCNA_03155	2.33741E-04	1.44712E-04	6.9167E-01	4.2036E-01
CCNA_03156	1.56757E-04	9.68374E-05	6.9481E-01	4.2216E-01
CCNA_03157	7.74169E-05	1.02008E-04	-3.9802E-01	-2.0106E-01
CCNA_03158	2.16423E-05	8.41690E-05	-1.9594E+00	-1.0915E+00
CCNA_03159	2.27920E-05	1.05543E-04	-2.2111E+00	-1.2350E+00
CCNA_03160	1.68407E-04	7.52605E-05	1.1619E+00	6.8851E-01
CCNA_03161	1.31924E-04	1.09356E-04	2.7062E-01	1.8025E-01
CCNA_03162	5.53503E-05	7.43374E-05	-4.2556E-01	-2.1677E-01
CCNA_03163	3.11618E-05	8.39063E-05	-1.4290E+00	-7.8900E-01
CCNA_03164	3.19593E-05	8.01688E-05	-1.3268E+00	-7.3072E-01
CCNA_03165	4.16143E-05	3.99493E-05	5.8741E-02	5.9421E-02
CCNA_03166	4.93641E-05	6.92489E-05	-4.8840E-01	-2.5260E-01
CCNA_03167	4.94348E-04	9.21693E-05	2.4231E+00	1.4077E+00
CCNA_03168	3.56642E-05	1.10662E-04	-1.6336E+00	-9.0567E-01
CCNA_03169	2.70085E-05	1.39939E-04	-2.3732E+00	-1.3275E+00
CCNA_03170	4.39347E-05	5.39687E-05	-2.9686E-01	-1.4337E-01
CCNA_03171	3.40423E-04	1.33882E-04	1.3463E+00	7.9368E-01
CCNA_03172	9.32597E-05	6.71400E-05	4.7397E-01	2.9621E-01
CCNA_03173	9.81292E-05	5.61752E-05	8.0460E-01	4.8477E-01
CCNA_03174	7.57526E-05	8.39138E-05	-1.4768E-01	-5.8299E-02
CCNA_03175	8.46310E-05	1.38288E-04	-7.0844E-01	-3.7809E-01
CCNA_03176	3.75001E-05	9.42407E-05	-1.3295E+00	-7.3224E-01
CCNA_03177	1.84370E-05	1.29462E-04	-2.8117E+00	-1.5775E+00
CCNA_03178	6.12732E-05	1.47594E-04	-1.2683E+00	-6.9737E-01

CCNA_03179	3.57003E-05	1.01325E-04	-1.5050E+00	-8.3233E-01
CCNA_03180	2.94674E-04	9.27772E-05	1.6672E+00	9.7667E-01
CCNA_03181	3.15010E-04	7.95158E-05	1.9860E+00	1.1585E+00
CCNA_03182	5.05288E-05	1.47099E-04	-1.5416E+00	-8.5321E-01
CCNA_03183	1.23091E-04	1.32156E-04	-1.0256E-01	-3.2567E-02
CCNA_03184	2.67123E-04	9.01730E-05	1.5666E+00	9.1934E-01
CCNA_03185	2.18566E-04	8.42665E-05	1.3749E+00	8.1001E-01
CCNA_03186	2.32464E-05	1.22137E-04	-2.3933E+00	-1.3389E+00
CCNA_03187	2.19433E-05	6.86861E-05	-1.6462E+00	-9.1287E-01
CCNA_03188	2.66594E-05	6.53388E-05	-1.2933E+00	-7.1162E-01
CCNA_03189	3.24138E-05	5.71658E-05	-8.1860E-01	-4.4091E-01
CCNA_03190	2.23195E-05	1.20471E-04	-2.4322E+00	-1.3611E+00
CCNA_03191	1.52679E-04	9.44208E-05	6.9324E-01	4.2126E-01
CCNA_03192	2.30809E-05	7.08925E-05	-1.6189E+00	-8.9730E-01
CCNA_03193	2.80468E-05	5.50794E-05	-9.7372E-01	-5.2937E-01
CCNA_03194	2.60334E-05	1.04725E-04	-2.0081E+00	-1.1193E+00
CCNA_03195	2.54465E-05	1.10497E-04	-2.1184E+00	-1.1821E+00
CCNA_03196	7.60716E-05	1.22827E-04	-6.9123E-01	-3.6827E-01
CCNA_03197	1.05292E-04	1.16823E-04	-1.4998E-01	-5.9609E-02
CCNA_03198	3.90750E-04	1.32704E-04	1.5580E+00	9.1439E-01
CCNA_03199	1.56023E-04	3.87785E-05	2.0082E+00	1.1711E+00
CCNA_03200	8.48567E-05	8.32834E-05	2.6922E-02	4.1276E-02
CCNA_03201	1.36325E-04	1.47729E-04	-1.1595E-01	-4.0201E-02
CCNA_03202	6.51677E-05	1.06654E-04	-7.1074E-01	-3.7940E-01
CCNA_03203	8.22624E-05	1.00237E-04	-2.8517E-01	-1.3670E-01
CCNA_03204	2.16333E-05	1.00095E-04	-2.2099E+00	-1.2344E+00
CCNA_03205	2.58886E-04	7.53655E-05	1.7802E+00	1.0411E+00
CCNA_03206	8.53714E-05	1.20283E-04	-4.9465E-01	-2.5617E-01
CCNA_03207	2.64276E-05	4.47225E-05	-7.5903E-01	-4.0694E-01
CCNA_03208	2.42517E-05	9.85036E-05	-2.0220E+00	-1.1272E+00
CCNA_03209	2.52349E-04	6.87611E-05	1.8756E+00	1.0955E+00
CCNA_03210	2.40587E-04	1.02871E-04	1.2256E+00	7.2487E-01
CCNA_03211	1.17466E-04	8.35685E-05	4.9112E-01	3.0600E-01
CCNA_03212	2.16011E-04	1.36096E-04	6.6642E-01	4.0596E-01
CCNA_03213	1.98082E-04	1.47099E-04	4.2926E-01	2.7072E-01
CCNA_03214	1.61943E-04	2.91195E-05	2.4751E+00	1.4374E+00
CCNA_03215	1.43168E-04	1.09656E-04	3.8466E-01	2.4529E-01
CCNA_03216	1.91145E-04	1.39293E-04	4.5649E-01	2.8625E-01
CCNA_03217	1.76302E-04	9.57192E-05	8.8108E-01	5.2838E-01
CCNA_03218	1.57840E-04	1.47099E-04	1.0164E-01	8.3884E-02
CCNA_03219	6.56763E-05	7.06599E-05	-1.0561E-01	-3.4302E-02
CCNA_03220	3.24890E-05	1.29544E-04	-1.9954E+00	-1.1120E+00
CCNA_03221	2.35444E-05	1.12365E-04	-2.2547E+00	-1.2599E+00
CCNA_03222	1.81454E-04	1.09731E-04	7.2556E-01	4.3969E-01
CCNA_03223	3.32776E-05	5.30381E-05	-6.7255E-01	-3.5762E-01
CCNA_03224	7.85546E-05	1.04718E-04	-4.1479E-01	-2.1062E-01
CCNA_03225	6.97664E-05	1.01971E-04	-5.4760E-01	-2.8636E-01
CCNA_03226	4.63484E-05	8.85894E-05	-9.3464E-01	-5.0708E-01
CCNA_03227	1.38413E-04	1.01431E-04	4.4841E-01	2.8164E-01
CCNA_03228	8.87994E-05	1.43113E-04	-6.8857E-01	-3.6675E-01
CCNA_03229	1.18941E-04	9.14113E-05	3.7972E-01	2.4247E-01
CCNA_03230	5.79115E-05	1.21949E-04	-1.0744E+00	-5.8677E-01
CCNA_03231	5.22443E-05	1.47947E-04	-1.5017E+00	-8.3048E-01

CCNA_03232	5.59552E-05	1.19308E-04	-1.0924E+00	-5.9702E-01
CCNA_03233	2.86879E-05	1.44472E-04	-2.3322E+00	-1.3041E+00
CCNA_03234	2.60273E-05	7.50653E-05	-1.5281E+00	-8.4552E-01
CCNA_03235	1.52101E-04	8.46568E-05	8.4523E-01	5.0794E-01
CCNA_03236	1.36132E-04	7.16956E-05	9.2493E-01	5.5339E-01
CCNA_03237	1.29797E-04	6.31023E-05	1.0404E+00	6.1921E-01
CCNA_03238	1.87651E-05	4.29513E-05	-1.1947E+00	-6.5537E-01
CCNA_03239	2.16814E-05	1.24253E-04	-2.5186E+00	-1.4104E+00
CCNA_03240	7.48196E-05	1.03862E-04	-4.7323E-01	-2.4395E-01
CCNA_03241	3.18381E-04	5.27754E-05	2.5926E+00	1.5044E+00
CCNA_03242	6.06954E-05	1.45830E-04	-1.2646E+00	-6.9527E-01
CCNA_03243	4.26646E-05	1.46251E-04	-1.7773E+00	-9.8763E-01
CCNA_03244	1.20509E-04	1.22445E-04	-2.3042E-02	1.2782E-02
CCNA_03245	1.17773E-04	1.47339E-04	-3.2316E-01	-1.5837E-01
CCNA_03246	5.44835E-05	1.02624E-04	-9.1350E-01	-4.9503E-01
CCNA_03247	6.22333E-05	1.43609E-04	-1.2064E+00	-6.6206E-01
CCNA_03248	6.16825E-05	4.45799E-05	4.6830E-01	2.9298E-01
CCNA_03249	5.66023E-05	1.23293E-04	-1.1232E+00	-6.1459E-01
CCNA_03250	9.51076E-05	1.14129E-04	-2.6308E-01	-1.2411E-01
CCNA_04004	8.79175E-05	1.43204E-04	-7.0387E-01	-3.7548E-01
CCNA_03252	6.91043E-05	1.47016E-04	-1.0891E+00	-5.9519E-01
CCNA_03253	1.39662E-04	9.07359E-05	6.2211E-01	3.8070E-01
CCNA_03254	9.18241E-05	1.25109E-04	-4.4628E-01	-2.2858E-01
CCNA_03255	2.95667E-05	1.46378E-04	-2.3076E+00	-1.2900E+00
CCNA_03256	2.71800E-05	1.15855E-04	-2.0916E+00	-1.1669E+00
CCNA_03257	9.84422E-05	1.47399E-04	-5.8241E-01	-3.0621E-01
CCNA_03258	2.49920E-05	1.43038E-04	-2.5168E+00	-1.4093E+00
CCNA_03259	1.31633E-04	1.15668E-04	1.8647E-01	1.3226E-01
CCNA_03260	3.12792E-05	1.02579E-04	-1.7134E+00	-9.5121E-01
CCNA_03261	3.53903E-05	1.19840E-04	-1.7597E+00	-9.7757E-01
CCNA_03262	5.74871E-05	1.40307E-04	-1.2873E+00	-7.0818E-01
CCNA_03263	6.95106E-05	8.83493E-05	-3.4605E-01	-1.7142E-01
CCNA_03264	2.21458E-04	1.24103E-04	8.3543E-01	5.0235E-01
CCNA_03265	1.39268E-04	8.34184E-05	7.3933E-01	4.4754E-01
CCNA_03266	2.41078E-04	1.47099E-04	7.1266E-01	4.3234E-01
CCNA_03267	2.46724E-04	5.12068E-05	2.2683E+00	1.3195E+00
CCNA_03268	2.19373E-05	1.43226E-04	-2.7067E+00	-1.5177E+00
CCNA_03269	2.34481E-05	9.07734E-05	-1.9527E+00	-1.0877E+00
CCNA_03271	1.79618E-04	9.87963E-05	8.6232E-01	5.1768E-01
CCNA_03270	1.10111E-04	1.32449E-04	-2.6652E-01	-1.2607E-01
CCNA_03272	6.04817E-05	1.09596E-04	-8.5765E-01	-4.6318E-01
CCNA_03274	1.13623E-04	7.43899E-05	6.1097E-01	3.7434E-01
CCNA_03275	7.87050E-05	1.15172E-04	-5.4930E-01	-2.8733E-01
CCNA_03276	1.25354E-04	7.01345E-05	8.3770E-01	5.0364E-01
CCNA_03277	1.30089E-04	5.89821E-05	1.1410E+00	6.7661E-01
CCNA_03278	2.70446E-05	7.97110E-05	-1.5594E+00	-8.6338E-01
CCNA_03279	5.48266E-05	5.80890E-05	-8.3496E-02	-2.1693E-02
CCNA_03280	2.60126E-04	1.24606E-04	1.0618E+00	6.3142E-01
CCNA_03281	1.09373E-04	5.85468E-05	9.0145E-01	5.4000E-01
CCNA_03282	2.28251E-05	1.46971E-04	-2.6867E+00	-1.5063E+00
CCNA_03283	8.47273E-05	1.41530E-04	-7.4023E-01	-3.9622E-01
CCNA_03284	1.04913E-04	1.07314E-04	-3.2711E-02	7.2680E-03
CCNA_03285	2.83779E-05	1.06977E-04	-1.9144E+00	-1.0658E+00

CCNA_03286	1.34230E-04	9.61545E-05	4.8120E-01	3.0034E-01
CCNA_03287	1.48775E-04	7.65513E-05	9.5852E-01	5.7255E-01
CCNA_03288	1.38178E-04	8.07091E-05	7.7563E-01	4.6824E-01
CCNA_03289	1.40827E-04	9.59519E-05	5.5346E-01	3.4155E-01
CCNA_03290	1.29180E-04	7.78947E-05	7.2968E-01	4.4204E-01
CCNA_03291	3.59378E-04	6.94441E-05	2.3714E+00	1.3783E+00
CCNA_03292	6.03583E-05	1.15750E-04	-9.3941E-01	-5.0980E-01
CCNA_03293	2.10946E-05	6.66672E-05	-1.6601E+00	-9.2077E-01
CCNA_03294	1.02833E-04	9.14188E-05	1.6967E-01	1.2268E-01
CCNA_03295	2.38523E-04	1.07795E-04	1.1458E+00	6.7932E-01
CCNA_03296	1.23046E-04	7.87878E-05	6.4305E-01	3.9264E-01
CCNA_03297	2.05086E-04	1.45838E-04	4.9181E-01	3.0639E-01
CCNA_03298	3.91434E-05	1.36051E-04	-1.7973E+00	-9.9903E-01
CCNA_03299	2.54194E-05	7.74444E-05	-1.6072E+00	-8.9063E-01
CCNA_03300	2.99941E-05	1.05378E-04	-1.8128E+00	-1.0079E+00
CCNA_03301	1.08723E-04	1.02166E-04	8.9678E-02	7.7064E-02
CCNA_03302	3.54355E-05	0.00000E+00	1.2205E+01	6.9863E+00
CCNA_03303	4.24178E-05	4.08949E-05	5.2587E-02	5.5911E-02
CCNA_03304	1.76515E-05	1.17124E-04	-2.7300E+00	-1.5309E+00
CCNA_03305	2.38153E-05	1.21867E-04	-2.3553E+00	-1.3172E+00
CCNA_03306	2.55819E-05	5.41338E-05	-1.0814E+00	-5.9080E-01
CCNA_03307	2.31471E-05	1.15563E-04	-2.3197E+00	-1.2969E+00
CCNA_03308	2.49950E-05	9.95918E-05	-1.9943E+00	-1.1114E+00
CCNA_03309	2.62943E-04	1.29942E-04	1.0168E+00	6.0579E-01
CCNA_03310	3.85505E-05	1.47091E-04	-1.9319E+00	-1.0758E+00
CCNA_03311	1.21806E-04	3.90787E-05	1.6399E+00	9.6111E-01
CCNA_03312	1.57034E-04	8.62629E-05	8.6417E-01	5.1874E-01
CCNA_03313	2.24218E-05	1.04357E-04	-2.2185E+00	-1.2392E+00
CCNA_03314	1.95386E-05	2.88569E-05	-5.6274E-01	-2.9500E-01
CCNA_03315	1.26360E-04	1.08493E-04	2.1987E-01	1.5131E-01
CCNA_03316	2.42520E-04	4.69965E-05	2.3673E+00	1.3759E+00
CCNA_03317	1.88163E-05	8.00337E-05	-2.0885E+00	-1.1651E+00
CCNA_03318	1.21833E-04	3.96716E-05	1.6185E+00	9.4891E-01
CCNA_03319	5.73670E-04	1.47099E-04	1.9634E+00	1.1456E+00
CCNA_03320	6.69753E-04	1.37192E-04	2.2874E+00	1.3304E+00
CCNA_03321	1.25701E-04	7.30765E-05	7.8240E-01	4.7211E-01
CCNA_03322	2.19854E-05	7.60560E-05	-1.7905E+00	-9.9514E-01
CCNA_03323	4.71941E-05	8.29606E-05	-8.1386E-01	-4.3820E-01
CCNA_03324	2.33969E-05	1.33612E-04	-2.5136E+00	-1.4075E+00
CCNA_03325	1.02081E-04	1.47099E-04	-5.2710E-01	-2.7467E-01
CCNA_03326	6.21581E-05	1.21949E-04	-9.7229E-01	-5.2855E-01
CCNA_03327	1.47099E-04	1.10159E-04	4.1713E-01	2.6380E-01
CCNA_03328	2.62392E-04	1.45755E-04	8.4812E-01	5.0959E-01
CCNA_03329	2.65269E-04	7.69341E-05	1.7856E+00	1.0442E+00
CCNA_03330	1.75974E-05	8.06641E-05	-2.1965E+00	-1.2267E+00
CCNA_03331	3.07465E-04	8.61203E-05	1.8359E+00	1.0729E+00
CCNA_03332	2.94722E-04	1.05206E-04	1.4861E+00	8.7339E-01
CCNA_03333	2.60093E-05	1.47376E-04	-2.5023E+00	-1.4011E+00
CCNA_03334	1.46765E-04	9.88488E-05	5.7013E-01	3.5106E-01
CCNA_03335	8.10285E-05	7.06449E-05	1.9774E-01	1.3869E-01
CCNA_03336	4.15992E-05	1.38716E-04	-1.7375E+00	-9.6492E-01
CCNA_03337	1.56029E-04	1.14152E-04	4.5079E-01	2.8300E-01
CCNA_03338	1.02590E-04	1.47099E-04	-5.1993E-01	-2.7058E-01

CCNA_03339	2.47016E-04	7.02696E-05	1.8135E+00	1.0601E+00
CCNA_03340	5.64397E-05	8.48294E-05	-5.8790E-01	-3.0935E-01
CCNA_03341	7.20176E-05	1.07307E-04	-5.7536E-01	-3.0219E-01
CCNA_03342	2.16859E-04	1.08538E-04	9.9848E-01	5.9533E-01
CCNA_03343	2.17627E-05	1.18069E-04	-2.4396E+00	-1.3653E+00
CCNA_03344	1.70887E-05	8.14821E-05	-2.2533E+00	-1.2591E+00
CCNA_03345	3.24048E-05	1.13896E-04	-1.8134E+00	-1.0082E+00
CCNA_03346	2.09736E-04	1.23720E-04	7.6142E-01	4.6014E-01
CCNA_03347	1.79359E-04	8.27430E-05	1.1160E+00	6.6237E-01
CCNA_03348	4.76275E-05	1.02887E-04	-1.1112E+00	-6.0777E-01
CCNA_03349	2.41075E-04	1.47099E-04	7.1264E-01	4.3233E-01
CCNA_03350	4.23312E-04	1.09701E-04	1.9481E+00	1.1369E+00
CCNA_03351	2.49920E-05	1.07667E-04	-2.1070E+00	-1.1756E+00
CCNA_03352	2.11186E-05	4.99535E-05	-1.2421E+00	-6.8241E-01
CCNA_03353	3.48817E-05	1.16200E-04	-1.7360E+00	-9.6410E-01
CCNA_03354	5.97774E-05	6.58792E-05	-1.4031E-01	-5.4095E-02
CCNA_03355	5.52088E-05	5.87269E-05	-8.9229E-02	-2.4963E-02
CCNA_03356	1.58319E-04	1.27826E-04	3.0860E-01	2.0191E-01
CCNA_03357	6.09361E-05	1.47099E-04	-1.2714E+00	-6.9914E-01
CCNA_03358	6.33649E-05	1.47099E-04	-1.2150E+00	-6.6698E-01
CCNA_03359	6.15561E-05	9.87137E-05	-6.8139E-01	-3.6266E-01
CCNA_03360	1.14186E-04	1.30002E-04	-1.8720E-01	-8.0833E-02
CCNA_03361	3.18871E-05	1.47054E-04	-2.2052E+00	-1.2317E+00
CCNA_03273	1.82414E-05	1.47099E-04	-3.0113E+00	-1.6914E+00
CCNA_03362	1.42567E-04	8.98202E-05	6.6643E-01	4.0597E-01
CCNA_03363	1.13611E-04	1.40141E-04	-3.0282E-01	-1.4677E-01
CCNA_03364	1.21743E-04	7.85177E-05	6.3265E-01	3.8671E-01
CCNA_03365	9.16284E-05	1.43144E-04	-6.4362E-01	-3.4112E-01
CCNA_03366	2.39357E-05	7.51404E-05	-1.6504E+00	-9.1526E-01
CCNA_03367	1.21075E-04	9.78806E-05	3.0673E-01	2.0084E-01
CCNA_03368	1.87236E-04	1.01310E-04	8.8599E-01	5.3118E-01
CCNA_03369	4.34682E-05	1.47099E-04	-1.7587E+00	-9.7704E-01
CCNA_03370	3.34220E-05	1.40975E-04	-2.0765E+00	-1.1583E+00
CCNA_03371	2.28561E-04	1.41162E-04	6.9517E-01	4.2236E-01
CCNA_03372	9.43281E-05	1.09716E-04	-2.1807E-01	-9.8437E-02
CCNA_03373	1.15194E-04	8.53247E-05	4.3294E-01	2.7282E-01
CCNA_03374	4.41424E-05	1.47099E-04	-1.7365E+00	-9.6438E-01
CCNA_03375	8.46762E-05	7.18382E-05	2.3711E-01	1.6114E-01
CCNA_03376	4.34802E-05	6.53163E-05	-5.8715E-01	-3.0892E-01
CCNA_03377	1.35593E-04	9.75429E-05	4.7510E-01	2.9686E-01
CCNA_03378	1.54087E-04	1.42738E-04	1.1033E-01	8.8841E-02
CCNA_03379	3.69072E-05	5.76086E-05	-6.4245E-01	-3.4045E-01
CCNA_03380	4.33960E-05	1.01708E-04	-1.2288E+00	-6.7484E-01
CCNA_03381	5.18440E-05	7.57183E-05	-5.4652E-01	-2.8575E-01
CCNA_03382	9.06413E-05	2.73558E-05	1.7280E+00	1.0113E+00
CCNA_03383	1.34994E-04	1.50318E-04	-1.5516E-01	-6.2563E-02
CCNA_03384	1.29739E-04	1.48299E-04	-1.9294E-01	-8.4104E-02
CCNA_03385	1.24834E-04	1.21169E-04	4.2936E-02	5.0408E-02
CCNA_03386	1.23215E-04	8.66231E-05	5.0826E-01	3.1577E-01
CCNA_03387	4.90752E-05	1.28261E-04	-1.3860E+00	-7.6449E-01
CCNA_03388	6.45868E-05	4.74618E-05	4.4431E-01	2.7930E-01
CCNA_03389	2.63232E-04	9.21768E-05	1.5138E+00	8.8918E-01
CCNA_03390	2.28624E-04	1.32764E-04	7.8405E-01	4.7305E-01

CCNA_03391	5.78693E-05	1.34453E-04	-1.2162E+00	-6.6767E-01
CCNA_03392	2.09441E-05	1.33875E-04	-2.6761E+00	-1.5002E+00
CCNA_03393	2.44963E-04	1.03539E-04	1.2423E+00	7.3438E-01
CCNA_03394	2.25271E-04	8.10243E-05	1.4751E+00	8.6715E-01
CCNA_03395	3.25459E-04	8.61803E-05	1.9169E+00	1.1191E+00
CCNA_03396	7.88766E-05	1.11750E-04	-5.0265E-01	-2.6073E-01
CCNA_03397	1.06008E-04	1.11637E-04	-7.4696E-02	-1.6675E-02
CCNA_03398	2.72478E-04	1.55782E-04	8.0656E-01	4.8588E-01
CCNA_03399	1.22998E-04	1.32426E-04	-1.0660E-01	-3.4871E-02
CCNA_03400	1.19350E-04	6.93465E-05	7.8319E-01	4.7256E-01
CCNA_03401	6.78132E-05	1.47174E-04	-1.1179E+00	-6.1159E-01
CCNA_03402	7.73206E-05	1.32059E-04	-7.7228E-01	-4.1449E-01
CCNA_03403	1.30733E-04	5.33158E-05	1.2938E+00	7.6376E-01
CCNA_03404	6.49329E-05	1.28801E-04	-9.8814E-01	-5.3759E-01
CCNA_03405	3.97408E-04	1.45305E-04	1.4515E+00	8.5366E-01
CCNA_03406	4.24112E-04	1.47099E-04	1.5276E+00	8.9708E-01
CCNA_03407	1.52462E-04	1.01543E-04	5.8628E-01	3.6027E-01
CCNA_03408	2.12631E-04	1.21086E-04	8.1225E-01	4.8913E-01
CCNA_03409	2.24308E-05	1.04680E-04	-2.2223E+00	-1.2414E+00
CCNA_03410	1.52417E-04	6.17514E-05	1.3033E+00	7.6918E-01
CCNA_04013	1.11212E-04	1.21859E-04	-1.3195E-01	-4.9326E-02
CCNA_03412	7.12321E-05	7.57183E-05	-8.8196E-02	-2.4374E-02
CCNA_03413	2.07027E-04	1.13581E-04	8.6602E-01	5.1979E-01
CCNA_03414	5.75924E-05	1.08380E-04	-9.1217E-01	-4.9427E-01
CCNA_03415	9.18662E-05	8.98803E-05	3.1456E-02	4.3861E-02
CCNA_03416	8.65903E-05	1.14744E-04	-4.0619E-01	-2.0572E-01
CCNA_03417	6.70728E-05	1.47099E-04	-1.1330E+00	-6.2020E-01
CCNA_03418	1.41646E-04	1.37725E-04	4.0449E-02	4.8989E-02
CCNA_03419	1.49973E-04	1.15728E-04	3.7391E-01	2.3915E-01
CCNA_03420	1.32048E-04	6.67573E-05	9.8393E-01	5.8704E-01
CCNA_03421	1.92271E-04	5.12744E-05	1.9066E+00	1.1132E+00
CCNA_03422	3.02649E-05	6.78980E-05	-1.1657E+00	-6.3887E-01
CCNA_03423	1.21668E-04	1.03922E-04	2.2737E-01	1.5559E-01
CCNA_03424	1.19865E-04	1.12883E-04	8.6518E-02	7.5262E-02
CCNA_03425	3.64377E-05	8.81391E-05	-1.2744E+00	-7.0081E-01
CCNA_03426	2.02937E-04	1.05018E-04	9.5031E-01	5.6786E-01
CCNA_03427	2.26241E-04	6.73802E-05	1.7473E+00	1.0224E+00
CCNA_03428	1.64016E-04	1.34550E-04	2.8564E-01	1.8882E-01
CCNA_03429	1.46061E-04	6.91063E-05	1.0796E+00	6.4157E-01
CCNA_03430	1.54518E-04	9.32951E-05	7.2781E-01	4.4098E-01
CCNA_03431	1.53913E-04	9.63796E-05	6.7523E-01	4.1099E-01
CCNA_03432	1.37170E-04	1.04035E-04	3.9883E-01	2.5337E-01
CCNA_03433	1.30025E-04	6.87311E-05	9.1963E-01	5.5037E-01
CCNA_03434	2.02488E-04	1.47099E-04	4.6100E-01	2.8882E-01
CCNA_03435	2.05318E-05	1.12853E-04	-2.4584E+00	-1.3760E+00
CCNA_03436	7.93220E-05	3.75477E-05	1.0788E+00	6.4112E-01
CCNA_03437	1.45444E-04	1.42746E-04	2.6968E-02	4.1302E-02
CCNA_03438	9.26186E-05	6.14437E-05	5.9191E-01	3.6347E-01
CCNA_03439	4.67216E-05	1.47189E-04	-1.6555E+00	-9.1816E-01
CCNA_03440	2.47693E-05	0.00000E+00	1.1689E+01	6.6917E+00
CCNA_03441	0.00000E+00	0.00000E+00	-1.3183E+00	-7.2586E-01
CCNA_03442	4.38516E-04	8.42815E-05	2.3792E+00	1.3827E+00
CCNA_03443	2.90972E-05	7.87803E-05	-1.4369E+00	-7.9353E-01

CCNA_03444	2.91995E-05	8.01312E-05	-1.4564E+00	-8.0463E-01
CCNA_03445	1.85063E-04	1.42543E-04	3.7656E-01	2.4067E-01
CCNA_03446	4.65079E-05	9.77531E-05	-1.0717E+00	-5.8523E-01
CCNA_03447	1.12690E-04	1.48652E-04	-3.9962E-01	-2.0197E-01
CCNA_03448	1.67107E-04	1.47556E-04	1.7946E-01	1.2826E-01
CCNA_03449	1.12747E-04	5.80890E-05	9.5661E-01	5.7145E-01
CCNA_03450	9.96642E-05	1.11840E-04	-1.6634E-01	-6.8939E-02
CCNA_03451	2.38902E-04	1.23270E-04	9.5453E-01	5.7027E-01
CCNA_03452	1.14135E-04	6.15413E-07	7.5175E+00	4.3130E+00
CCNA_03453	1.85451E-04	4.86101E-05	1.9315E+00	1.1274E+00
CCNA_03454	1.78159E-04	3.88160E-05	2.1982E+00	1.2795E+00
CCNA_03455	1.23407E-04	1.23638E-04	-2.7472E-03	2.4356E-02
CCNA_03456	1.67125E-05	6.41230E-05	-1.9398E+00	-1.0803E+00
CCNA_03457	1.25475E-04	6.37327E-05	9.7716E-01	5.8317E-01
CCNA_03458	6.49660E-05	1.37680E-04	-1.0836E+00	-5.9201E-01
CCNA_03459	6.87552E-05	1.43579E-04	-1.0623E+00	-5.7989E-01
CCNA_03460	1.08476E-04	1.41365E-04	-3.8208E-01	-1.9197E-01
CCNA_03461	5.67016E-05	1.17792E-04	-1.0548E+00	-5.7560E-01
CCNA_03462	4.83017E-05	1.41275E-04	-1.5483E+00	-8.5706E-01
CCNA_03463	5.10976E-05	1.06121E-04	-1.0544E+00	-5.7538E-01
CCNA_03464	1.49636E-04	1.39391E-04	1.0227E-01	8.4247E-02
CCNA_03465	3.58129E-04	9.24845E-05	1.9531E+00	1.1397E+00
CCNA_03466	3.16370E-04	7.54481E-05	2.0679E+00	1.2052E+00
CCNA_03467	4.61257E-05	9.54940E-05	-1.0499E+00	-5.7279E-01
CCNA_03468	4.56682E-05	9.57492E-05	-1.0681E+00	-5.8318E-01
CCNA_03469	5.46851E-05	7.17256E-05	-3.9141E-01	-1.9729E-01
CCNA_04011	2.06305E-04	1.27413E-04	6.9520E-01	4.2238E-01
CCNA_03471	2.36329E-04	8.77714E-05	1.4289E+00	8.4077E-01
CCNA_03472	1.88012E-04	1.43354E-04	3.9119E-01	2.4901E-01
CCNA_03473	1.87585E-04	1.44757E-04	3.7386E-01	2.3912E-01
CCNA_03474	9.35967E-05	9.79632E-05	-6.5846E-02	-1.1628E-02
CCNA_03475	1.44192E-04	8.75912E-05	7.1904E-01	4.3597E-01
CCNA_03476	1.21054E-04	1.37725E-04	-1.8619E-01	-8.0255E-02
CCNA_03477	4.04977E-05	1.10114E-04	-1.4431E+00	-7.9703E-01
CCNA_03478	5.61749E-05	5.95900E-05	-8.5248E-02	-2.2693E-02
CCNA_03479	3.49449E-05	2.14119E-05	7.0629E-01	4.2870E-01
CCNA_03480	3.36658E-05	8.24578E-05	-1.2924E+00	-7.1109E-01
CCNA_03481	1.43226E-04	1.01431E-04	4.9772E-01	3.0976E-01
CCNA_03482	8.42909E-05	1.31893E-04	-6.4595E-01	-3.4245E-01
CCNA_03483	3.64828E-05	1.23578E-04	-1.7601E+00	-9.7782E-01
CCNA_03484	1.41901E-04	1.32809E-04	9.5484E-02	8.0375E-02
CCNA_03485	2.04466E-04	8.66831E-05	1.2379E+00	7.3189E-01
CCNA_03486	4.88735E-05	9.25371E-05	-9.2101E-01	-4.9931E-01
CCNA_03487	1.12967E-04	9.13363E-05	3.0656E-01	2.0075E-01
CCNA_03488	8.66174E-05	8.71184E-05	-8.3956E-03	2.1135E-02
CCNA_03489	7.53373E-05	1.34460E-04	-8.3577E-01	-4.5070E-01
CCNA_03490	1.07158E-04	5.29255E-05	1.0175E+00	6.0620E-01
CCNA_03491	3.28382E-05	1.47099E-04	-2.1633E+00	-1.2077E+00
CCNA_03492	2.28751E-04	1.12831E-04	1.0195E+00	6.0734E-01
CCNA_03493	2.61658E-05	1.40862E-04	-2.4284E+00	-1.3590E+00
CCNA_03494	3.43346E-04	1.47099E-04	1.2228E+00	7.2327E-01
CCNA_03495	4.48827E-05	1.21064E-04	-1.4315E+00	-7.9045E-01
CCNA_03496	4.74530E-05	6.41530E-05	-4.3510E-01	-2.2220E-01

CCNA_03497	3.51887E-05	6.74778E-05	-9.3934E-01	-5.0976E-01
CCNA_03498	8.16455E-05	1.08778E-04	-4.1399E-01	-2.1017E-01
CCNA_03499	1.60263E-04	1.46228E-04	1.3218E-01	1.0130E-01
CCNA_03500	7.10545E-05	7.79248E-05	-1.3323E-01	-5.0057E-02
CCNA_03501	3.05388E-05	1.25484E-04	-2.0387E+00	-1.1367E+00
CCNA_03502	1.51890E-04	1.46363E-04	5.3433E-02	5.6394E-02
CCNA_03503	1.67875E-04	1.16741E-04	5.2401E-01	3.2475E-01
CCNA_03504	1.89126E-04	9.92391E-05	9.3028E-01	5.5644E-01
CCNA_03505	2.06615E-04	9.37754E-05	1.1396E+00	6.7579E-01
CCNA_03506	1.99389E-04	9.81208E-05	1.0229E+00	6.0924E-01
CCNA_03507	1.50969E-04	1.33807E-04	1.7405E-01	1.2518E-01
CCNA_03508	1.26959E-04	1.47594E-04	-2.1732E-01	-9.8008E-02
CCNA_03509	3.25793E-05	1.39564E-04	-2.0988E+00	-1.1710E+00
CCNA_03510	8.44595E-05	1.41380E-04	-7.4327E-01	-3.9795E-01
CCNA_03511	2.65269E-05	1.40742E-04	-2.4074E+00	-1.3470E+00
CCNA_03512	2.00683E-05	1.07825E-04	-2.4256E+00	-1.3573E+00
CCNA_03513	8.46551E-05	1.46949E-04	-7.9566E-01	-4.2783E-01
CCNA_03514	3.61458E-04	1.12823E-04	1.6797E+00	9.8381E-01
CCNA_03515	2.87992E-04	7.79923E-05	1.8845E+00	1.1006E+00
CCNA_03516	1.63562E-04	4.08499E-05	2.0012E+00	1.1672E+00
CCNA_03517	7.82235E-05	1.46896E-04	-9.0914E-01	-4.9254E-01
CCNA_03518	2.32139E-04	1.20643E-04	9.4417E-01	5.6436E-01
CCNA_03519	1.68784E-04	8.36511E-05	1.0126E+00	6.0339E-01
CCNA_03520	5.26958E-05	1.47099E-04	-1.4810E+00	-8.1867E-01
CCNA_03521	2.61718E-05	1.47384E-04	-2.4934E+00	-1.3960E+00
CCNA_03522	5.12782E-05	1.47099E-04	-1.5204E+00	-8.4110E-01
CCNA_03523	3.22904E-05	1.32854E-04	-2.0406E+00	-1.1378E+00
CCNA_03524	4.54666E-05	1.47061E-04	-1.6935E+00	-9.3985E-01
CCNA_03525	1.16705E-04	9.17791E-05	3.4655E-01	2.2355E-01
CCNA_03526	4.56953E-05	1.07074E-04	-1.2285E+00	-6.7466E-01
CCNA_03527	2.26385E-05	1.13041E-04	-2.3199E+00	-1.2971E+00
CCNA_03528	3.21760E-05	1.45500E-04	-2.1769E+00	-1.2155E+00
CCNA_03529	2.43690E-05	1.40157E-04	-2.5238E+00	-1.4134E+00
CCNA_03530	7.62672E-05	9.24245E-05	-2.7727E-01	-1.3220E-01
CCNA_03531	1.55547E-04	7.38946E-05	1.0737E+00	6.3823E-01
CCNA_03532	1.31792E-04	8.75162E-05	5.9055E-01	3.6270E-01
CCNA_03533	3.10384E-05	1.33229E-04	-2.1017E+00	-1.1726E+00
CCNA_03534	4.87983E-05	1.46281E-04	-1.5838E+00	-8.7730E-01
CCNA_03535	9.79276E-05	1.47264E-04	-5.8864E-01	-3.0977E-01
CCNA_03536	2.65390E-05	7.72943E-05	-1.5422E+00	-8.5357E-01
CCNA_03537	1.60299E-04	1.39789E-04	1.9747E-01	1.3854E-01
CCNA_03538	1.44749E-04	1.05941E-04	4.5021E-01	2.8267E-01
CCNA_03539	7.44554E-05	1.36862E-04	-8.7829E-01	-4.7495E-01
CCNA_03540	2.15021E-04	1.16756E-04	8.8091E-01	5.2828E-01
CCNA_03541	7.30469E-05	1.47099E-04	-1.0099E+00	-5.5000E-01
CCNA_03542	1.67914E-04	1.28914E-04	3.8125E-01	2.4334E-01
CCNA_03543	2.04836E-05	1.42866E-04	-2.8020E+00	-1.5720E+00
CCNA_03544	8.47695E-05	1.46776E-04	-7.9202E-01	-4.2575E-01
CCNA_03545	1.40625E-04	1.45853E-04	-5.2700E-02	-4.1313E-03
CCNA_03546	1.37959E-04	1.23540E-04	1.5920E-01	1.1671E-01
CCNA_03547	9.52550E-05	1.43796E-04	-5.9419E-01	-3.1293E-01
CCNA_03548	1.16094E-04	1.06969E-04	1.1803E-01	9.3233E-02
CCNA_03549	1.45131E-04	8.73436E-05	7.3249E-01	4.4364E-01

CCNA_03550	9.24470E-05	6.35376E-05	5.4089E-01	3.3438E-01
CCNA_03551	1.64299E-04	1.17176E-04	4.8758E-01	3.0398E-01
CCNA_03552	9.41415E-05	6.63670E-05	5.0425E-01	3.1348E-01
CCNA_03553	5.17628E-05	5.10267E-05	2.0533E-02	3.7632E-02
CCNA_03554	3.43174E-04	1.06189E-04	1.6922E+00	9.9096E-01
CCNA_03555	8.83931E-05	6.70049E-05	3.9955E-01	2.5378E-01
CCNA_03556	4.91986E-05	1.47099E-04	-1.5801E+00	-8.7516E-01
CCNA_03557	3.32866E-05	8.56249E-05	-1.3631E+00	-7.5141E-01
CCNA_03558	1.85803E-04	1.06594E-04	8.0157E-01	4.8304E-01
CCNA_03559	1.80759E-04	1.47099E-04	2.9723E-01	1.9543E-01
CCNA_03560	2.56903E-05	1.32216E-04	-2.3635E+00	-1.3219E+00
CCNA_03561	8.17839E-05	1.27676E-04	-6.4263E-01	-3.4055E-01
CCNA_03562	1.04636E-04	1.47099E-04	-4.9144E-01	-2.5433E-01
CCNA_03563	4.66163E-05	1.38971E-04	-1.5759E+00	-8.7275E-01
CCNA_03564	1.11814E-04	1.06301E-04	7.2880E-02	6.7484E-02
CCNA_03565	4.21298E-04	1.28816E-04	1.7095E+00	1.0008E+00
CCNA_03566	3.87602E-04	8.44091E-05	2.1990E+00	1.2800E+00
CCNA_03567	4.39828E-05	1.13656E-04	-1.3697E+00	-7.5516E-01
CCNA_03568	3.62240E-05	1.40809E-04	-1.9587E+00	-1.0911E+00
CCNA_03569	6.81954E-05	9.22143E-05	-4.3537E-01	-2.2236E-01
CCNA_03570	1.82384E-05	6.45508E-05	-1.8234E+00	-1.0139E+00
CCNA_03571	1.93038E-05	3.69623E-05	-9.3724E-01	-5.0856E-01
CCNA_03572	1.03501E-04	1.47414E-04	-5.1025E-01	-2.6506E-01
CCNA_03573	3.36899E-05	1.21206E-04	-1.8470E+00	-1.0274E+00
CCNA_03574	1.87558E-04	9.49612E-05	9.8183E-01	5.8584E-01
CCNA_03575	1.90757E-04	9.43007E-05	1.0163E+00	6.0550E-01
CCNA_03576	1.69262E-05	5.88620E-05	-1.7980E+00	-9.9944E-01
CCNA_03577	1.20578E-04	3.75102E-05	1.6844E+00	9.8648E-01
CCNA_03578	1.29752E-04	1.21514E-04	9.4573E-02	7.9855E-02
CCNA_03579	2.45316E-05	7.94258E-05	-1.6949E+00	-9.4066E-01
CCNA_03580	6.14779E-05	6.81532E-05	-1.4880E-01	-5.8936E-02
CCNA_03581	6.57455E-05	1.25019E-04	-9.2720E-01	-5.0284E-01
CCNA_03582	1.11420E-04	7.16956E-05	6.3594E-01	3.8858E-01
CCNA_03583	7.15752E-05	1.47099E-04	-1.0393E+00	-5.6675E-01
CCNA_03584	1.08756E-04	8.04540E-05	4.3477E-01	2.7386E-01
CCNA_03585	5.97022E-05	1.11180E-04	-8.9706E-01	-4.8565E-01
CCNA_03586	1.28045E-04	1.24876E-04	3.6099E-02	4.6509E-02
CCNA_03587	4.04044E-05	1.47099E-04	-1.8642E+00	-1.0372E+00
CCNA_03588	5.21059E-05	1.42903E-04	-1.4555E+00	-8.0412E-01
CCNA_03589	1.47656E-04	1.40119E-04	7.5538E-02	6.9000E-02
CCNA_03590	3.75154E-04	9.48186E-05	1.9841E+00	1.1574E+00
CCNA_03591	3.74844E-04	9.35202E-05	2.0028E+00	1.1681E+00
CCNA_03592	1.97884E-05	9.06908E-05	-2.1962E+00	-1.2265E+00
CCNA_03593	1.27579E-04	1.13919E-04	1.6332E-01	1.1906E-01
CCNA_03594	1.39518E-04	1.42926E-04	-3.4863E-02	6.0411E-03
CCNA_03595	3.01566E-05	1.24066E-04	-2.0405E+00	-1.1377E+00
CCNA_03596	5.01496E-05	1.32907E-04	-1.4061E+00	-7.7594E-01
CCNA_03597	3.25014E-04	7.18157E-05	2.1780E+00	1.2680E+00
CCNA_03598	3.11555E-04	7.49978E-05	2.0544E+00	1.1975E+00
CCNA_03599	1.55210E-04	1.23240E-04	3.3269E-01	2.1565E-01
CCNA_03600	3.09902E-05	8.91373E-05	-1.5242E+00	-8.4329E-01
CCNA_03601	1.02150E-04	1.47399E-04	-5.2907E-01	-2.7579E-01
CCNA_03602	1.60895E-05	7.80749E-05	-2.2786E+00	-1.2735E+00

CCNA_03603	1.74980E-05	1.03772E-04	-2.5680E+00	-1.4386E+00
CCNA_03604	3.94293E-05	1.05408E-04	-1.4186E+00	-7.8310E-01
CCNA_03605	8.19735E-05	5.96725E-05	4.5796E-01	2.8709E-01
CCNA_03606	3.99018E-05	1.41680E-04	-1.8281E+00	-1.0166E+00
CCNA_03607	1.05331E-04	4.46925E-05	1.2366E+00	7.3114E-01
CCNA_03608	9.28654E-05	8.08292E-05	2.0018E-01	1.4008E-01
CCNA_03609	1.86486E-04	9.93741E-05	9.0804E-01	5.4376E-01
CCNA_03610	4.28813E-05	1.23000E-04	-1.5202E+00	-8.4103E-01
CCNA_03611	5.03543E-05	1.47099E-04	-1.5466E+00	-8.5606E-01
CCNA_03612	2.60153E-05	1.52675E-04	-2.5529E+00	-1.4300E+00
CCNA_03613	2.64246E-05	1.35428E-04	-2.3575E+00	-1.3185E+00
CCNA_03614	2.73847E-05	1.20681E-04	-2.1397E+00	-1.1943E+00
CCNA_03615	5.17598E-05	4.03471E-05	3.5918E-01	2.3076E-01
CCNA_03616	3.08819E-05	6.70425E-05	-1.1183E+00	-6.1184E-01
CCNA_03617	2.04234E-05	1.05116E-04	-2.3636E+00	-1.3220E+00
CCNA_03618	3.46470E-05	5.92522E-05	-7.7420E-01	-4.1559E-01
CCNA_03619	5.35354E-05	1.38708E-04	-1.3735E+00	-7.5734E-01
CCNA_03620	2.13504E-05	9.83159E-05	-2.2031E+00	-1.2304E+00
CCNA_03621	3.28863E-05	9.29348E-05	-1.4987E+00	-8.2876E-01
CCNA_03622	2.62982E-05	1.44750E-04	-2.4604E+00	-1.3772E+00
CCNA_03623	4.85725E-05	1.19210E-04	-1.2953E+00	-7.1275E-01
CCNA_03624	2.14888E-05	1.30407E-04	-2.6012E+00	-1.4575E+00
CCNA_03625	1.97372E-05	1.15547E-04	-2.5494E+00	-1.4279E+00
CCNA_03626	2.47783E-05	1.15015E-04	-2.2146E+00	-1.2370E+00
CCNA_03627	1.35064E-04	5.42239E-05	1.3165E+00	7.7668E-01
CCNA_03628	1.22652E-04	1.39939E-04	-1.9027E-01	-8.2585E-02
CCNA_03629	5.20020E-04	1.01198E-04	2.3613E+00	1.3725E+00
CCNA_03630	4.13645E-04	4.84825E-05	3.0926E+00	1.7896E+00
CCNA_03631	4.15490E-04	4.72217E-05	3.1371E+00	1.8149E+00
CCNA_03632	1.81463E-04	1.14347E-04	6.6619E-01	4.0584E-01
CCNA_03633	5.35174E-05	1.46573E-04	-1.4535E+00	-8.0300E-01
CCNA_03634	4.08619E-05	3.50485E-06	3.5404E+00	2.0449E+00
CCNA_03635	2.57896E-05	8.27655E-05	-1.6822E+00	-9.3340E-01
CCNA_03636	2.45316E-05	6.89337E-05	-1.4906E+00	-8.2411E-01
CCNA_03637	2.66774E-05	1.47099E-04	-2.4630E+00	-1.3787E+00
CCNA_03638	1.69617E-04	7.06824E-05	1.2627E+00	7.4603E-01
CCNA_03639	1.47842E-04	1.00703E-04	5.5388E-01	3.4179E-01
CCNA_03640	9.87853E-05	1.25417E-04	-3.4440E-01	-1.7048E-01
CCNA_03641	1.34561E-04	1.47099E-04	-1.2857E-01	-4.7396E-02
CCNA_03642	3.23867E-05	8.62103E-05	-1.4125E+00	-7.7957E-01
CCNA_03643	2.75773E-05	9.54715E-05	-1.7915E+00	-9.9576E-01
CCNA_03644	2.95035E-05	9.94342E-05	-1.7528E+00	-9.7367E-01
CCNA_03645	2.50014E-04	1.47099E-04	7.6517E-01	4.6228E-01
CCNA_03646	6.67417E-05	1.14880E-04	-7.8349E-01	-4.2088E-01
CCNA_03647	4.82806E-05	1.43714E-04	-1.5737E+00	-8.7151E-01
CCNA_03648	2.88775E-05	8.81466E-05	-1.6099E+00	-8.9219E-01
CCNA_03649	1.72970E-04	1.06361E-04	7.0147E-01	4.2596E-01
CCNA_03650	4.95657E-05	5.90271E-05	-2.5213E-01	-1.1786E-01
CCNA_03651	1.12855E-04	1.41237E-04	-3.2368E-01	-1.5867E-01
CCNA_03652	3.92096E-05	1.47069E-04	-1.9072E+00	-1.0617E+00
CCNA_03653	6.02770E-05	9.80983E-05	-7.0266E-01	-3.7479E-01
CCNA_03654	2.06010E-05	1.46198E-04	-2.8270E+00	-1.5863E+00
CCNA_03655	2.12390E-05	9.18541E-05	-2.1125E+00	-1.1788E+00

CCNA_03656	2.36612E-04	1.21792E-04	9.5804E-01	5.7227E-01
CCNA_03657	1.51162E-04	1.08215E-04	4.8212E-01	3.0087E-01
CCNA_03658	3.78823E-05	1.11555E-04	-1.5581E+00	-8.6265E-01
CCNA_03659	1.44053E-04	1.36314E-04	7.9621E-02	7.1328E-02
CCNA_03660	1.95142E-04	6.98944E-05	1.4811E+00	8.7058E-01
CCNA_03661	7.01456E-05	1.43316E-04	-1.0308E+00	-5.6191E-01
CCNA_03662	3.06411E-05	1.01025E-04	-1.7211E+00	-9.5561E-01
CCNA_03663	1.40929E-04	1.41763E-04	-8.5507E-03	2.1046E-02
CCNA_03664	1.40186E-04	1.43579E-04	-3.4546E-02	6.2218E-03
CCNA_03665	9.51497E-05	6.97518E-05	4.4786E-01	2.8133E-01
CCNA_03666	5.62862E-05	8.84693E-05	-6.5244E-01	-3.4615E-01
CCNA_03667	3.38283E-05	1.34280E-04	-1.9889E+00	-1.1083E+00
CCNA_03668	3.48305E-05	8.94150E-05	-1.3602E+00	-7.4975E-01
CCNA_03669	2.88203E-05	1.40884E-04	-2.2893E+00	-1.2796E+00
CCNA_03670	2.59641E-05	9.83084E-05	-1.9207E+00	-1.0694E+00
CCNA_03671	5.55760E-05	1.16621E-04	-1.0693E+00	-5.8388E-01
CCNA_03672	3.45356E-05	1.47099E-04	-2.0906E+00	-1.1663E+00
CCNA_03673	5.30479E-05	1.38198E-04	-1.3814E+00	-7.6184E-01
CCNA_03674	2.71063E-04	9.76255E-05	1.4732E+00	8.6606E-01
CCNA_03675	2.65736E-04	9.41056E-05	1.4975E+00	8.7994E-01
CCNA_03676	1.48631E-04	1.41470E-04	7.1192E-02	6.6522E-02
CCNA_03677	2.36588E-05	5.83591E-05	-1.3026E+00	-7.1691E-01
CCNA_03678	2.92356E-05	1.47099E-04	-2.3309E+00	-1.3033E+00
CCNA_03679	2.81792E-05	1.47099E-04	-2.3840E+00	-1.3336E+00
CCNA_03680	9.93421E-05	1.48074E-04	-5.7587E-01	-3.0249E-01
CCNA_03681	3.49645E-04	1.30843E-04	1.4180E+00	8.3457E-01
CCNA_03682	2.77997E-04	1.47091E-04	9.1830E-01	5.4961E-01
CCNA_03683	2.89106E-05	1.14542E-04	-1.9861E+00	-1.1067E+00
CCNA_03684	4.88615E-05	1.07104E-04	-1.1323E+00	-6.1978E-01
CCNA_03685	2.25392E-05	2.85942E-05	-3.4347E-01	-1.6995E-01
CCNA_03686	6.61699E-05	7.82700E-05	-2.4236E-01	-1.1229E-01
CCNA_03687	7.33389E-05	1.31999E-04	-8.4789E-01	-4.5761E-01
CCNA_03688	2.02163E-04	8.38913E-05	1.2688E+00	7.4950E-01
CCNA_03689	2.36747E-04	1.24231E-04	9.3026E-01	5.5643E-01
CCNA_03690	2.40046E-04	1.27886E-04	9.0839E-01	5.4396E-01
CCNA_03691	1.33887E-04	1.18902E-04	1.7118E-01	1.2354E-01
CCNA_03692	1.43828E-04	7.70917E-05	8.9958E-01	5.3893E-01
CCNA_03693	2.93079E-05	1.46618E-04	-2.3226E+00	-1.2986E+00
CCNA_03694	2.91574E-05	1.46100E-04	-2.3250E+00	-1.2999E+00
CCNA_03695	1.08350E-04	1.47324E-04	-4.4333E-01	-2.2690E-01
CCNA_03696	2.43720E-05	7.74595E-05	-1.6682E+00	-9.2540E-01
CCNA_03697	2.53261E-05	1.24989E-04	-2.3030E+00	-1.2874E+00
CCNA_03698	2.03692E-05	1.46070E-04	-2.8421E+00	-1.5948E+00
CCNA_03699	1.31789E-04	1.37320E-04	-5.9353E-02	-7.9253E-03
CCNA_03700	1.71477E-04	1.20996E-04	5.0299E-01	3.1277E-01
CCNA_03701	1.68985E-04	1.48412E-04	1.8724E-01	1.3270E-01
CCNA_03702	2.28416E-04	8.35160E-05	1.4514E+00	8.5364E-01
CCNA_03703	5.93651E-05	1.04260E-04	-8.1253E-01	-4.3744E-01
CCNA_03704	4.44012E-05	6.83784E-05	-6.2300E-01	-3.2936E-01
CCNA_03705	2.96991E-05	1.20328E-04	-2.0184E+00	-1.1251E+00
CCNA_03706	9.22003E-05	1.37230E-04	-5.7378E-01	-3.0129E-01
CCNA_03707	2.69453E-05	0.00000E+00	1.1810E+01	6.7609E+00
CCNA_03708	0.00000E+00	0.00000E+00	-1.3183E+00	-7.2586E-01

CCNA_03709	2.82659E-04	0.00000E+00	1.5201E+01	8.6946E+00
CCNA_03710	2.73967E-05	8.44767E-05	-1.6245E+00	-9.0050E-01
CCNA_03711	3.07149E-04	8.02438E-05	1.9364E+00	1.1302E+00
CCNA_03712	1.66165E-04	1.47309E-04	1.7373E-01	1.2499E-01
CCNA_03713	2.88955E-05	1.37425E-04	-2.2497E+00	-1.2570E+00
CCNA_03714	2.30716E-04	1.47099E-04	6.4928E-01	3.9619E-01
CCNA_03715	1.18336E-04	1.26580E-04	-9.7209E-02	-2.9513E-02
CCNA_03716	1.25198E-04	1.09791E-04	1.8939E-01	1.3392E-01
CCNA_03717	3.28469E-04	1.17296E-04	1.4855E+00	8.7308E-01
CCNA_03718	1.12660E-04	1.15623E-04	-3.7505E-02	4.5340E-03
CCNA_03719	1.28454E-04	1.47099E-04	-1.9557E-01	-8.5606E-02
CCNA_03720	1.34714E-04	6.27871E-05	1.1012E+00	6.5393E-01
CCNA_03721	1.22766E-04	1.47076E-04	-2.6069E-01	-1.2274E-01
CCNA_03722	3.70908E-05	1.43271E-04	-1.9496E+00	-1.0859E+00
CCNA_03723	6.36930E-05	1.35353E-04	-1.0875E+00	-5.9427E-01
CCNA_03724	1.07423E-04	1.47406E-04	-4.5653E-01	-2.3443E-01
CCNA_03725	7.53704E-05	1.12576E-04	-5.7886E-01	-3.0419E-01
CCNA_03726	1.57416E-04	6.59017E-05	1.2561E+00	7.4222E-01
CCNA_03727	1.13641E-04	5.71583E-05	9.9130E-01	5.9124E-01
CCNA_03728	6.03553E-05	1.03329E-04	-7.7573E-01	-4.1646E-01
CCNA_03729	3.53783E-05	1.39894E-04	-1.9834E+00	-1.1051E+00
CCNA_03730	2.41283E-05	1.06744E-04	-2.1453E+00	-1.1975E+00
CCNA_03731	9.73227E-05	1.47099E-04	-5.9597E-01	-3.1394E-01
CCNA_03732	1.21246E-04	1.27360E-04	-7.1026E-02	-1.4582E-02
CCNA_03733	3.81682E-05	1.47099E-04	-1.9463E+00	-1.0840E+00
CCNA_03734	9.04065E-05	1.29950E-04	-5.2349E-01	-2.7261E-01
CCNA_03735	7.15060E-05	1.46716E-04	-1.0369E+00	-5.6540E-01
CCNA_03736	3.91253E-04	1.13694E-04	1.7829E+00	1.0426E+00
CCNA_03737	1.01584E-04	1.33132E-04	-3.9022E-01	-1.9661E-01
CCNA_03738	1.00411E-04	7.44724E-05	4.3103E-01	2.7173E-01
CCNA_03739	1.98696E-05	1.47339E-04	-2.8904E+00	-1.6224E+00
CCNA_03740	1.16608E-04	1.47099E-04	-3.3515E-01	-1.6521E-01
CCNA_03741	2.77729E-04	1.47429E-04	9.1360E-01	5.4693E-01
CCNA_03742	2.74235E-04	8.14972E-05	1.7505E+00	1.0242E+00
CCNA_03743	1.39548E-04	9.51338E-05	5.5265E-01	3.4108E-01
CCNA_03744	2.68550E-05	9.48186E-05	-1.8199E+00	-1.0119E+00
CCNA_03745	1.52637E-04	8.79140E-05	7.9584E-01	4.7977E-01
CCNA_03746	1.58788E-04	9.01730E-05	8.1625E-01	4.9141E-01
CCNA_03747	2.19198E-04	1.24546E-04	8.1549E-01	4.9098E-01
CCNA_03748	1.54006E-04	1.45117E-04	8.5722E-02	7.4808E-02
CCNA_03749	5.74510E-05	7.41047E-05	-3.6730E-01	-1.8354E-01
CCNA_03750	6.07706E-05	8.74862E-05	-5.2573E-01	-2.7389E-01
CCNA_03751	3.52519E-05	1.19698E-04	-1.7636E+00	-9.7981E-01
CCNA_03752	4.44072E-05	1.47099E-04	-1.7279E+00	-9.5946E-01
CCNA_03753	5.54526E-05	1.38918E-04	-1.3249E+00	-7.2964E-01
CCNA_03754	1.92466E-04	1.37139E-04	4.8891E-01	3.0473E-01
CCNA_03755	2.32224E-05	7.58083E-05	-1.7068E+00	-9.4743E-01
CCNA_03756	1.19898E-04	1.14347E-04	6.8334E-02	6.4892E-02
CCNA_03757	4.04255E-05	1.33867E-04	-1.7274E+00	-9.5920E-01
CCNA_03758	5.03211E-05	1.44975E-04	-1.5266E+00	-8.4464E-01
CCNA_03759	3.19894E-05	1.47099E-04	-2.2011E+00	-1.2293E+00
CCNA_03760	2.58558E-05	1.15908E-04	-2.1643E+00	-1.2084E+00
CCNA_03761	2.34282E-04	1.46558E-04	6.7672E-01	4.1184E-01

CCNA_03762	6.67628E-05	1.23495E-04	-8.8736E-01	-4.8012E-01
CCNA_03763	3.15591E-05	6.20891E-05	-9.7632E-01	-5.3085E-01
CCNA_03764	3.08668E-05	7.38570E-05	-1.2587E+00	-6.9188E-01
CCNA_03765	3.14823E-04	1.50896E-04	1.0609E+00	6.3095E-01
CCNA_03766	1.54187E-04	1.38228E-04	1.5758E-01	1.1579E-01
CCNA_03767	1.16040E-04	1.47556E-04	-3.4669E-01	-1.7178E-01
CCNA_03768	1.53792E-04	1.13514E-04	4.3805E-01	2.7573E-01
CCNA_03769	1.17599E-04	1.47099E-04	-3.2295E-01	-1.5825E-01
CCNA_03770	1.56462E-04	9.86912E-05	6.6474E-01	4.0501E-01
CCNA_03771	1.72693E-04	1.21972E-04	5.0160E-01	3.1197E-01
CCNA_03772	2.47753E-05	1.06699E-04	-2.1065E+00	-1.1754E+00
CCNA_03773	8.47394E-05	1.32029E-04	-6.3978E-01	-3.3893E-01
CCNA_03774	2.92146E-05	1.28509E-04	-2.1370E+00	-1.1928E+00
CCNA_03775	3.38614E-05	1.46941E-04	-2.1175E+00	-1.1816E+00
CCNA_03776	2.90280E-05	1.09926E-04	-1.9210E+00	-1.0696E+00
CCNA_03777	5.08779E-05	1.13566E-04	-1.1584E+00	-6.3470E-01
CCNA_03778	5.17838E-05	1.13251E-04	-1.1290E+00	-6.1790E-01
CCNA_03779	3.92141E-04	1.47834E-04	1.4073E+00	8.2849E-01
CCNA_03780	3.26576E-05	1.14542E-04	-1.8103E+00	-1.0065E+00
CCNA_03781	1.45426E-04	1.41245E-04	4.2038E-02	4.9896E-02
CCNA_03782	1.44216E-04	1.45673E-04	-1.4545E-02	1.7628E-02
CCNA_03783	2.02236E-04	1.07532E-04	9.1119E-01	5.4555E-01
CCNA_03784	1.24882E-04	1.11180E-04	1.6761E-01	1.2151E-01
CCNA_04010	1.05217E-04	1.16230E-04	-1.4367E-01	-5.6011E-02
CCNA_03786	1.60173E-05	1.46311E-04	-3.1911E+00	-1.7939E+00
CCNA_03787	9.15803E-05	9.43458E-05	-4.2988E-02	1.4071E-03
CCNA_03788	1.54978E-04	9.83760E-05	6.5561E-01	3.9980E-01
CCNA_03789	2.74948E-04	1.35541E-04	1.0204E+00	6.0782E-01
CCNA_03790	2.81091E-04	1.13424E-04	1.3092E+00	7.7255E-01
CCNA_03791	4.51536E-05	1.09551E-04	-1.2787E+00	-7.0329E-01
CCNA_03792	3.01265E-05	1.39556E-04	-2.2117E+00	-1.2353E+00
CCNA_03793	2.62892E-05	1.33214E-04	-2.3411E+00	-1.3092E+00
CCNA_03794	2.69724E-05	1.47099E-04	-2.4471E+00	-1.3696E+00
CCNA_03795	4.10605E-05	6.27496E-05	-6.1192E-01	-3.2304E-01
CCNA_03796	7.37662E-05	1.25462E-04	-7.6624E-01	-4.1105E-01
CCNA_03797	6.90772E-05	1.34318E-04	-9.5938E-01	-5.2119E-01
CCNA_03798	2.03542E-05	1.20756E-04	-2.5686E+00	-1.4389E+00
CCNA_03799	4.40701E-05	7.95158E-05	-8.5148E-01	-4.5966E-01
CCNA_03800	4.69744E-05	1.47099E-04	-1.6468E+00	-9.1322E-01
CCNA_03801	5.24279E-05	9.20793E-05	-8.1258E-01	-4.3747E-01
CCNA_03802	1.67700E-04	1.43654E-04	2.2324E-01	1.5323E-01
CCNA_03803	1.56059E-04	1.37852E-04	1.7891E-01	1.2795E-01
CCNA_03804	1.42991E-04	9.59143E-05	5.7602E-01	3.5442E-01
CCNA_03805	9.32807E-05	4.37994E-05	1.0905E+00	6.4779E-01
CCNA_03806	9.59021E-05	5.84417E-05	7.1442E-01	4.3334E-01
CCNA_03807	1.33294E-04	1.47091E-04	-1.4214E-01	-5.5138E-02
CCNA_03808	1.76789E-04	1.15180E-04	6.1807E-01	3.7840E-01
CCNA_03809	1.71546E-04	7.06374E-05	1.2800E+00	7.5586E-01
CCNA_03810	5.65661E-05	1.47099E-04	-1.3788E+00	-7.6036E-01
CCNA_03811	5.82846E-05	9.41657E-05	-6.9213E-01	-3.6878E-01
CCNA_03812	6.85776E-05	1.06977E-04	-6.4153E-01	-3.3993E-01
CCNA_03813	2.98469E-04	1.47099E-04	1.0207E+00	6.0803E-01
CCNA_03814	2.24218E-05	1.37597E-04	-2.6174E+00	-1.4667E+00

CCNA_03815	1.89758E-05	7.61085E-05	-2.0038E+00	-1.1168E+00
CCNA_03816	2.41966E-04	1.10872E-04	1.1258E+00	6.6796E-01
CCNA_03817	2.53965E-04	1.19600E-04	1.0863E+00	6.4544E-01
CCNA_03818	4.85816E-05	0.00000E+00	1.2660E+01	7.2458E+00
CCNA_03819	1.89186E-05	1.34933E-04	-2.8342E+00	-1.5904E+00
CCNA_03820	1.44210E-04	1.27518E-04	1.7741E-01	1.2710E-01
CCNA_03821	8.24671E-05	6.74027E-05	2.9090E-01	1.9182E-01
CCNA_03822	2.10735E-05	5.56573E-05	-1.4011E+00	-7.7311E-01
CCNA_03823	2.03301E-05	8.77864E-05	-2.1103E+00	-1.1775E+00
CCNA_03824	1.28033E-04	7.25286E-05	8.1978E-01	4.9342E-01
CCNA_03825	5.45437E-05	1.28088E-04	-1.2317E+00	-6.7647E-01
CCNA_03826	2.10250E-04	6.61269E-05	1.6687E+00	9.7752E-01
CCNA_03827	1.99151E-04	6.84234E-05	1.5412E+00	9.0481E-01
CCNA_03828	9.85777E-05	1.47099E-04	-5.7748E-01	-3.0340E-01
CCNA_03829	1.64669E-04	1.31563E-04	3.2376E-01	2.1056E-01
CCNA_03830	2.76315E-04	1.24373E-04	1.1516E+00	6.8263E-01
CCNA_03831	3.37350E-05	1.49605E-04	-2.1488E+00	-1.1995E+00
CCNA_03832	1.19383E-04	9.50362E-05	3.2897E-01	2.1353E-01
CCNA_03833	2.39718E-05	1.47346E-04	-2.6197E+00	-1.4680E+00
CCNA_03834	3.89628E-05	8.20525E-05	-1.0745E+00	-5.8682E-01
CCNA_03835	1.29258E-04	1.28141E-04	1.2470E-02	3.3034E-02
CCNA_03836	4.59843E-05	1.45838E-04	-1.6651E+00	-9.2367E-01
CCNA_03837	1.29300E-04	1.43234E-04	-1.4769E-01	-5.8301E-02
CCNA_03838	5.16845E-05	1.12741E-04	-1.1252E+00	-6.1576E-01
CCNA_03839	7.46029E-05	1.31496E-04	-8.1774E-01	-4.4041E-01
CCNA_03840	3.53572E-05	1.45875E-04	-2.0446E+00	-1.1401E+00
CCNA_03841	8.30931E-05	1.15645E-04	-4.7694E-01	-2.4607E-01
CCNA_03842	5.45888E-05	1.08230E-04	-9.8744E-01	-5.3720E-01
CCNA_03843	6.07887E-05	1.47391E-04	-1.2778E+00	-7.0277E-01
CCNA_03844	4.82505E-05	1.41462E-04	-1.5518E+00	-8.5903E-01
CCNA_03845	7.09552E-05	1.47069E-04	-1.0515E+00	-5.7374E-01
CCNA_03846	4.19363E-04	1.47099E-04	1.5114E+00	8.8781E-01
CCNA_03847	2.87986E-04	1.04643E-04	1.4604E+00	8.5878E-01
CCNA_03848	4.25593E-05	1.47099E-04	-1.7892E+00	-9.9442E-01
CCNA_03849	7.31011E-05	9.73703E-05	-4.1364E-01	-2.0997E-01
CCNA_03850	2.33488E-05	1.07089E-04	-2.1973E+00	-1.2272E+00
CCNA_03851	3.29917E-05	1.47099E-04	-2.1566E+00	-1.2039E+00
CCNA_03852	2.00204E-04	1.23045E-04	7.0222E-01	4.2638E-01
CCNA_03853	3.29706E-05	1.47099E-04	-2.1575E+00	-1.2044E+00
CCNA_03854	4.67457E-05	7.34743E-05	-6.5246E-01	-3.4616E-01
CCNA_03855	1.05163E-04	1.25402E-04	-2.5398E-01	-1.1891E-01
CCNA_03856	2.04264E-05	9.49462E-05	-2.2166E+00	-1.2381E+00
CCNA_03857	2.07214E-04	3.29021E-05	2.6546E+00	1.5398E+00
CCNA_03858	2.01733E-04	8.49270E-05	1.2480E+00	7.3766E-01
CCNA_03859	1.55355E-04	1.46881E-04	8.0870E-02	7.2041E-02
CCNA_03860	1.95386E-04	8.36286E-05	1.2241E+00	7.2403E-01
CCNA_03861	3.05237E-05	9.59669E-05	-1.6526E+00	-9.1650E-01
CCNA_03862	2.57324E-05	1.38911E-04	-2.4324E+00	-1.3612E+00
CCNA_03863	3.89559E-04	7.92006E-05	2.2981E+00	1.3365E+00
CCNA_03864	3.12051E-04	1.08553E-04	1.5233E+00	8.9463E-01
CCNA_03865	4.52439E-05	1.39804E-04	-1.6276E+00	-9.0226E-01
CCNA_03866	2.95065E-05	7.98160E-05	-1.4356E+00	-7.9279E-01
CCNA_03867	3.20087E-04	1.28404E-04	1.3177E+00	7.7738E-01

CCNA_03868	1.92433E-04	1.20688E-04	6.7300E-01	4.0972E-01
CCNA_03869	1.44180E-04	1.32089E-04	1.2631E-01	9.7954E-02
CCNA_03870	1.33423E-04	8.26379E-05	6.9104E-01	4.2000E-01
CCNA_03871	1.91572E-04	8.08217E-05	1.2450E+00	7.3590E-01
CCNA_03872	1.13310E-04	9.59293E-05	2.4015E-01	1.6288E-01
CCNA_03873	1.49161E-04	8.14221E-05	8.7327E-01	5.2393E-01
CCNA_03874	1.25586E-04	7.42548E-05	7.5801E-01	4.5820E-01
CCNA_03875	5.85977E-05	1.47099E-04	-1.3279E+00	-7.3133E-01
CCNA_03876	1.20199E-04	9.15389E-05	3.9289E-01	2.4998E-01
CCNA_03877	1.60007E-04	1.02564E-04	6.4154E-01	3.9178E-01
CCNA_03878	3.23957E-05	9.22068E-05	-1.5091E+00	-8.3466E-01
CCNA_03879	1.65608E-04	4.64562E-06	5.1535E+00	2.9648E+00
